# Predicting gene regulatory networks of soybean nodulation from RNA-Seq transcriptome data

**DOI:** 10.1186/1471-2105-14-278

**Published:** 2013-09-22

**Authors:** Mingzhu Zhu, Jeremy L Dahmen, Gary Stacey, Jianlin Cheng

**Affiliations:** 1 Department of Computer Science, University of Missouri, Columbia, MO 65211, USA; 2 Informatics Institute, University of Missouri, Columbia, MO, USA; 3 C.S. Bond Life Science Center, University of Missouri, Columbia, MO, USA; 4 Divisions of Plant Science and Biochemistry, Columbia, MO, USA; 5 Current address: Department of Genetics, Geisel School of Medicine at Dartmouth, Hanover, NH 03755, USA

## Abstract

**Background:**

High-throughput RNA sequencing (RNA-Seq) is a revolutionary technique to study the transcriptome of a cell under various conditions at a systems level. Despite the wide application of RNA-Seq techniques to generate experimental data in the last few years, few computational methods are available to analyze this huge amount of transcription data. The computational methods for constructing gene regulatory networks from RNA-Seq expression data of hundreds or even thousands of genes are particularly lacking and urgently needed.

**Results:**

We developed an automated bioinformatics method to predict gene regulatory networks from the quantitative expression values of differentially expressed genes based on RNA-Seq transcriptome data of a cell in different stages and conditions, integrating transcriptional, genomic and gene function data. We applied the method to the RNA-Seq transcriptome data generated for soybean root hair cells in three different development stages of nodulation after rhizobium infection. The method predicted a soybean nodulation-related gene regulatory network consisting of 10 regulatory modules common for all three stages, and 24, 49 and 70 modules separately for the first, second and third stage, each containing both a group of co-expressed genes and several transcription factors collaboratively controlling their expression under different conditions. 8 of 10 common regulatory modules were validated by at least two kinds of validations, such as independent DNA binding motif analysis, gene function enrichment test, and previous experimental data in the literature.

**Conclusions:**

We developed a computational method to reliably reconstruct gene regulatory networks from RNA-Seq transcriptome data. The method can generate valuable hypotheses for interpreting biological data and designing biological experiments such as ChIP-Seq, RNA interference, and yeast two hybrid experiments.

## Background

Gene expression information has been widely used to elucidate complex biological mechanisms, including the prediction of protein functions, the precise classification of phenotypes at the modular level, the study of expression modes under certain experimental conditions, and the reduction of experimental noise, with the ultimate aim of affecting the direction of biological research. RNA-Seq is a revolutionary DNA sequencing technology recently developed that provides a high throughput method for cDNA sequencing, generating information about mRNA content and quantifying gene expression. This kind of novel sequencing technology when contrasted with traditional microarray hybridization technology, reduces background noise and is sensitive enough to detect a wider range (>90%) of the transcriptome, even mRNA that are expressed at very low levels or that are rapidly degraded [1]. Not only can RNA-Seq more accurately measure gene expression levels [2], but this new technology promises to deliver more advantages, such as investigation of alternative splicing [3] and allele specific expression [4]. In addition, the combination of strand-specific array data and sequencing data reveals information on new, non-coding transcripts and gene structures distinct to each case [1], which largely benefits the study of condition specific sub-networks or modules in biological applications.

The widespread and growing application of RNA-Seq techniques to the study of various biological systems emphasize the need for computational methods to analyze the huge amount of RNA-Seq data, with the ultimate goal of obtaining a greater understanding of biological mechanisms at a systems level. In order to partially address this challenge, we developed and applied an array of bioinformatics methods to analyze the RNA-Seq transcriptome data obtained through studies of soybean nodulation. Soybean (*Glycine max* L. merr.), a major crop providing an important source of protein and oil, is very important in biological nitrogen fixation research. The symbiosis between leguminous plants and rhizobia leads to the formation of a novel root organ, the nodule. In mature nodules, rhizobia provide the host plant with ammonium, which is produced through bacterial nitrogen fixation. In recent years, research progress on understanding nodule formation has accelerated through the application of modern molecular methods. For example, using high-throughput sequencing technologies, we obtained gene expression data derived from different conditions (tissues) in soybean. With these data we constructed nodule-related gene regulatory networks as a tool to aid biologists to formulate testable hypothesis about how nodule development is regulated.

Several algorithms exist to infer regulatory networks from microarray gene expression data [5-8]. Among of them, the method based on the Bayesian probabilistic network [7] to infer co-regulated genes and their putative regulators, transcription factors, was successfully applied to the microarray data of a model species: *Saccharomyces cerevisiae*. However, the application of computational methods to predict plant gene regulatory networks is still at an early stage [8]. Specifically, there is a lack of bioinformatic tools or integration methods to combine RNA-Seq data with other data sources to study gene modules and their regulatory relationships. In the case of soybean, the availability of the complete genome sequence [9,10] and numerous annotation resources (e.g. SoyDB, a functional annotation database of all putative transcription factors [10]), makes it now possible to develop and integrate a set of bioinformatic methods to reliably construct gene regulatory modules by integrating the vast soybean RNA-Seq data with functional genomics data [8].

In line with an integrative bioinformatics framework for predicting gene regulatory networks from microarray gene expression data [8], here we developed and applied an integrated protocol for differential expression analysis, gene clustering, co-regulated gene module and regulator construction, DNA binding motif identification, and gene function prediction to construct and verify gene regulatory modules from RNA-Seq data. Although the basic framework of constructing regulatory module network is the same as our previous method [8] developed for microarray data, the preprocessing and normalization of RNA-Seq data, the filtering of differentially expressed genes, and the biological application are quite different. Here, considering the nodulation may have three separate stages, we separately selected the differentially expressed genes for each stage and also studied the differentially expressed genes commonly present in all the three stages. Thus, this work is a new application and adaptation of the previous framework for increasingly important RNA-Seq data analysis in a new biological context. Furthermore, we added a new random computational method to evaluate the predicted network models.

For the 10 regulatory modules constructed based on genes which respond at all the three stages of nodulation formation, we validated them from different aspects, such as, by existing literature, function enrichment and binding site analysis. The results demonstrated that we can obtain reliable results about regulatory mechanisms in the process of soybean nodulation formation by constructing regulatory networks and modules from RNA-Seq data. In addition, a series of condition specific regulatory networks and modules separately based on the different stages of nodulation were produced by our method. The experiments demonstrated that our computational methods can effectively integrate RNA-Seq transcriptome data with other data sources to construct gene regulatory networks for a cell responding to different biological conditions.

## Methods

### Data

#### RNA-Seq dataset

In this work we used the data set [11] generated for root hairs cell tissues in different stages of nodulation (12 hours, 24 hours and 48 hours) upon rhizobium infection to predict and analyze gene regulatory logic. Our differential expression analysis identified 354 genes differentially expressed in all three developmental stages. In order to better discover the transcription regulatory networks controlling the expression of these 354 genes, we augmented their expression data in the data set [11] with the only other RNA-Seq data set [12] of soybean nodulation in Soybean Knowledge Base (SoyKB) [13]. The data set [12] contains the expression data of different tissues, such as nodule, leave and seed. The combined data set contains 64,788 soybean genes and 34 experimental conditions in total. Both original data sets are available in the SoyKB [13]. The accession number of the data set [11] in the SRA repository is SRA012188.

For the two data sets, 36mer reads were aligned to all contigs of the Glyma1n8x Soybean genome assembly by using the program GSNAP [14]. Processing of the alignments was performed using the Alpheus Pipeline retaining only alignments which had a minimum of 34 out of 36 identities [15]. The raw count of each gene in each dataset was normalized by both the length of gene in terms of kilobase (KB) and the total number of reads in the dataset in terms of megabase (MB), resulting in the normalized gene expression value in terms of number of mapped reads per KB per MB. The normalized expression data of the two datasets were combined together for gene regulatory network construction.

#### Transcription factors

The set of transcription factors used in this study come from SoyDB [9]. SoyDB provides an automatic classification of predicted soybean transcription factors into one of 63 annotated transcription factor families using hidden Markov models. The number of overlapped genes between the RNA-Seq gene profile and the set of transcription factors from SoyDB is 5,474.

For each group of selected differentially expressed genes (see Methods section for details), we only considered differentially expressed TFs as possible gene regulators. The number of TFs used in gene regulatory network construction is 19 for common differentially expressed genes, and 87, 126 and 205 for the 12 hours, 24 hours and 48 hours development stage, respectively.

#### Soybean genomic and proteomic sequence data

We used genomic sequence data [9] for DNA binding analysis and protein sequence data for function prediction. We extracted 500 bps of genomic sequence located upstream to the start codon of each of the genes, and then used these sequences to further analyze for transcription factor binding sites.

#### Methods

The computational framework for RNA-Seq data analysis contains a filter for differentially expressed genes, the construction of regulatory module networks and validation of regulatory modules. In order to predict regulatory networks and their modules most relevant to specific experimental conditions, we only focus on the differentially expressed genes, which are induced or repressed under particular biological conditions. This approach reduces the complexity of modeling and increases the chance that the predicted regulatory networks will be relevant to the specific biological question under investigation. However, one potential limitation of the approach is that some relevant genes and transcription factors, whose expressions do not change significantly under the experimental conditions, will be missed from the analysis. This problem may be alleviated by incorporating prior knowledge (e.g. known relevant transcription factors) into the automated modeling process [8]. The following sections describe the detailed techniques used in the process.

#### Differential gene expression selection

Nodulation is the result of a mutualistic interaction between legumes and symbiotic soil bacteria (e.g., soybean [*Glycine max*] and *Bradyrhizobium japonicum*) initiated by the infection of plant root hair cells by the symbiont [16]. In order to identify the genes directly related with nodulation, we selected genes differentially expressed when soybean roots were inoculated with *B. japonicum.* These genes are referred to as differentially expressed genes (DEGs). Using the edgeR [17] package, we set the adjusted p value to 0.05 as the threshold to select the DEGs based on comparisons of expression values with three time points. We also used the DEGseq [18] package to select the DEGs, and used the default value 0.001 as the threshold.

#### Regulatory module network construction

A model-based strategy was used for inferring regulatory modules from RNA-Seq data. A regulatory module contains two parts: a regulatory network represented by a decision tree and its target genes as in [7,8]. In the decision tree, transcription factors were composed as a hierarchical structure predicted to collaboratively regulate their target genes. Each regulator (i.e., transcription factor) is denoted as a node of the hierarchical tree, and its expression status was separated into three situations: highly expressed (1), normally expressed (0), and lowly expressed (−1). As published previously [7,19], our strategy was based on the hypothesis: the regulators are themselves transcriptionally regulated, so that their expression profiles provide information about their activity level [20]. The expression status of a regulator was separated into the three activity levels (1, 0, -1) based on its expression values under all of the experimental conditions [7,21]. In contrast to Joshi et al.’s method [19] that classified gene expression status as either high or low, our method added one category to represent the normal expression level, considering that genes may be normally expressed in some conditions. With the expression status/activity levels of regulators, the expression values of target genes were modeled by a mixture of probability distributions [8]. In order for gene expression values to approximate normal distributions, here the logarithm values of gene expression values were used in the further analysis.

In order to construct the gene regulatory modules, our method initially clustered all the differentially expressed genes into a number of groups based on the similarity of their expression profiles under the various treatment conditions using the K-means algorithm [22]. Here, for the overlap DEG gene group, the number of experimental conditions used in clustering is 34, while for each of the other three DEG gene groups in which genes are selected separately based on different stages of nodulation, the number of experimental conditions used is 14. The number of initial clusters was determined automatically by balancing correlation coefficients of gene expression values in clusters and sizes of clusters. Generally speaking, the higher the number of clusters, the higher the correlation coefficients and the smaller the cluster sizes. Similarly as in [8], we obtained a series of average correlation coefficients and their corresponding average cluster sizes by varying the number of clusters, and then selected the range with the most drastic change on correlation coefficients and cluster sizes as the cluster number changes.

After the initial clustering, our method repeated two steps: (1) regulatory tree construction and (2) gene re-assignment to iteratively construct gene regulatory modules. In the tree construction step, a transcription factor (TF) was selected from the TF list to divide the genes in each cluster into two sub-set of conditions according to the expression status of the TF in these conditions, i.e., the conditions in which the TF has the same expression level (e.g., high versus normal/low) were grouped into the same sub-set. Based on the assumption that the expression values in each sub-set of conditions obey the normal distribution as in [8], the probability that a gene i (g_i_) is regulated by a TF can be calculated as $\frac{1}{\sqrt{2\pi }\;\sigma }e^{\frac{{\left(x-\mu \right)}^{2}}{2\sigma^{2}}}$, where μ is the mean expression value in the sub-set, σ the standard deviation of expression values in the sub-set, and *x* the expression value of the gene g_i_ in a condition assigned to the sub-set. The likelihood of the division by the TF is the multiplication of the probability of all the gene expression values in the two sub-sets of conditions. The division and the TF that produced the highest likelihood were selected. After the first division, each sub-set of conditions could be furthered divided into sub-sub-groups by incorporating another TF in the same way, resulting in a hierarchical, multi-level binary tree. The first TF selected forms the root of the tree and other TFs the internal nodes of the tree. A leaf node contains expression values of the genes in the conditions represented by the leaf node. After a tree was constructed for each cluster, our method entered into the second step to re-assign each gene into a tree to produce the highest likelihood of its expression values in all the conditions. A gene was re-assigned to a tree that generated the highest likelihood for its expression values in all the conditions. The likelihood of the expression values of a gene is the product of the probability of its expression value in each condition calculated according to the formula above. The genes assigned to the same tree formed a new cluster. The new clusters can be used to construct a new set of regulatory trees as described above. This process will iterate until the assignment of genes did not change. The detailed process can be found in the method [8] developed for constructing this kind of regulatory modules from microarray gene expression data.

#### Function prediction

A software MULTICOM-PDCN [23-25], for protein structure and function prediction, was used for the analysis of functional coherence for the referred regulatory modules. With MULTICOM-PDCN three categories of functions were predicted for the differentially expressed genes based on the sub-ontologies (i.e. biological processes (P), molecular function (F) and cellular component (C)) [25,26].

## Results and discussion

### Differential expression gene analysis

With the EdgeR [17] package, we set the adjusted p-value to 0.05 as the threshold to select the different expression genes (DEGs) based on three comparisons as follows.

• Group 1: In the comparison between root hairs at 12 hours after rhizobium inoculation vs mock inoculation, we identified 1101 DEGs.

• Group 2: In the comparison between root hairs at 24 hours after rhizobium inoculation vs mock inoculation, we identified 2168 DEGs.

• Group 3: In the comparison between root hairs at 48 hours after rhizobium inoculation vs mock inoculation, we identified 3081 DEGs.

**Figure 1:**
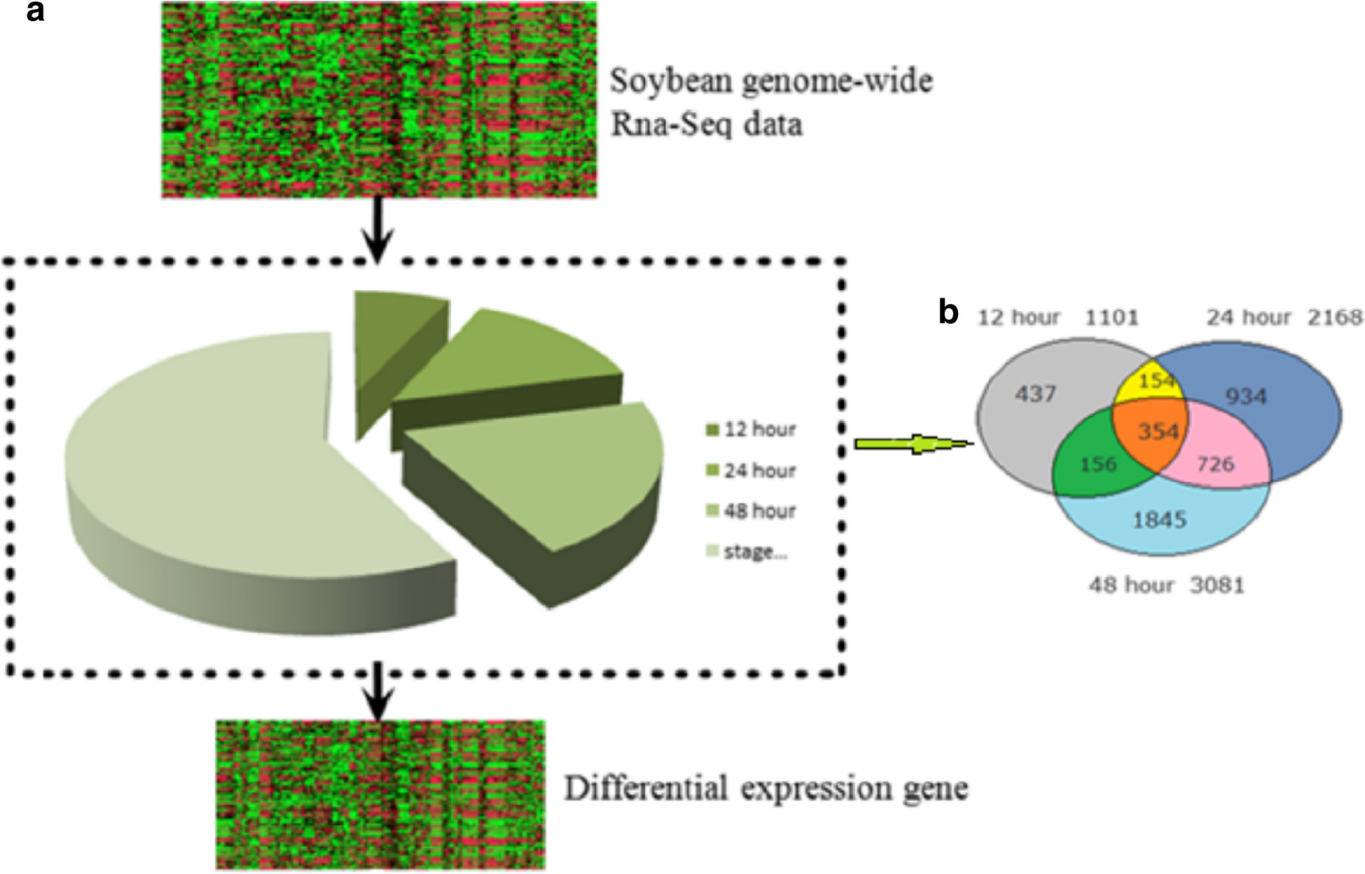
**Selection of differentially expressed genes (DEGs). a**: DEGs selection process; **b**: with p value threshold of 0.05, the DEG numbers of each set.

The total number of DEGs identified regardless of inoculation time was 4606. The number of DEGs consistently found at all the time points was 354. Figure 1 shows the numbers of each gene set.

**Figure 2:**
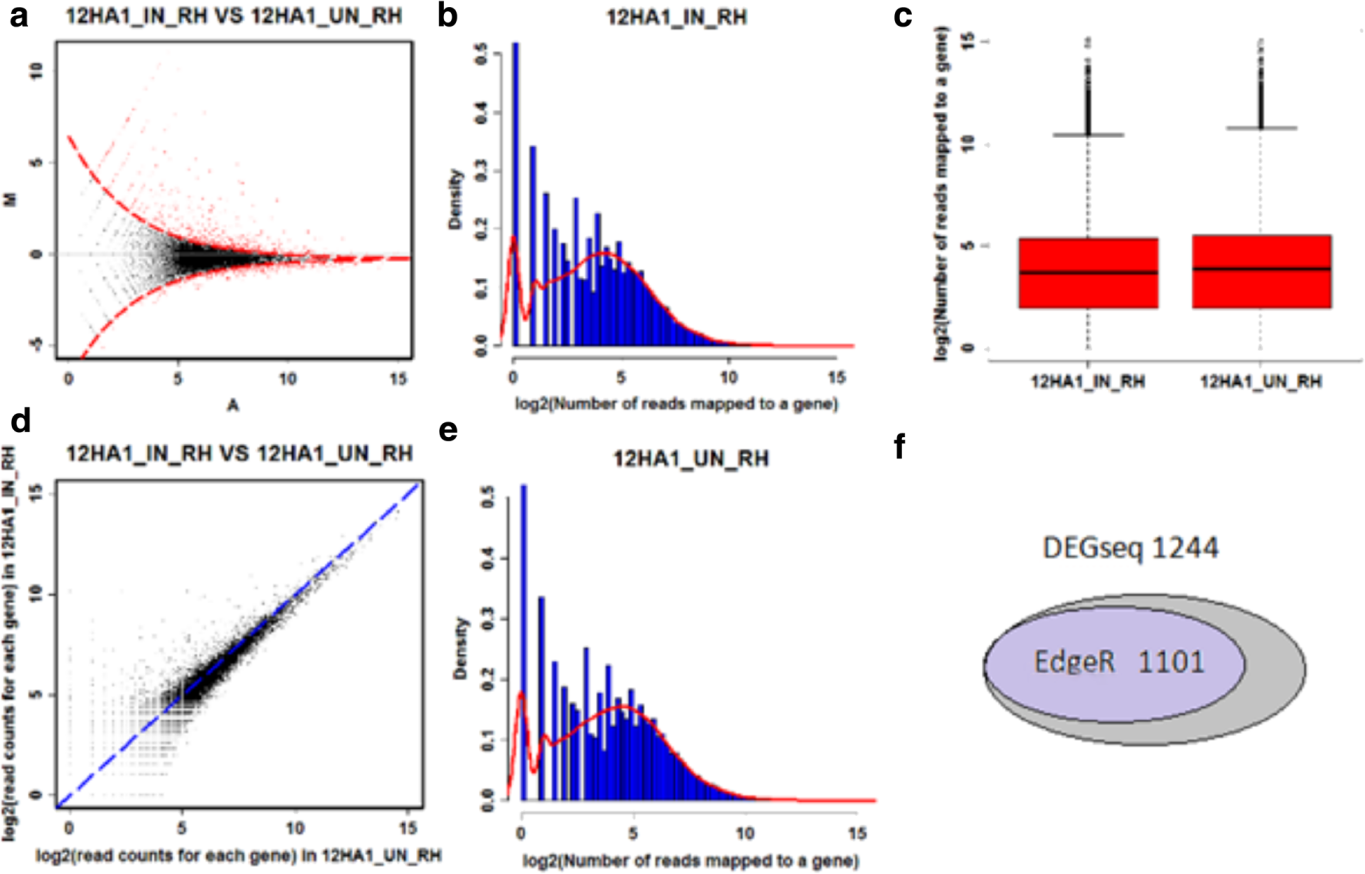
**Overview of the genes differentially expressed when comparing roots mock inoculated with those inoculated with Bradyrhizobium japonicum. (a)** Differentially expressed genes on the MA-plot. **(b)** Histogram of the number of reads for inoculated genes after 12 hours. **(c)** Boxplot of read counts for each group. **(d)** Scatterplot comparing the number of reads for each gene for inoculated and un-inoculated after 12 hours. **(e)** Histogram of the number of reads for un-inoculated genes after 12 hours. **(f)** Differentially expressed genes separately chosen by DEGseq and EdgeR.

In order to test the stability of identifying DEGs, we also used DEGseq to select the DEGs. Taking the genes in the comparison between 12 hours after rhizobium inoculation to mock inoculation, we compared the results of selected DEGs with DEGseq and EdgeR (Figure 2). All the DEGs selected with EdgeR are included in those with DEGseq. Therefore, for our further analyses we used the DEGs selected with EdgeR.

### Initial gene clustering

**Figure 3:**
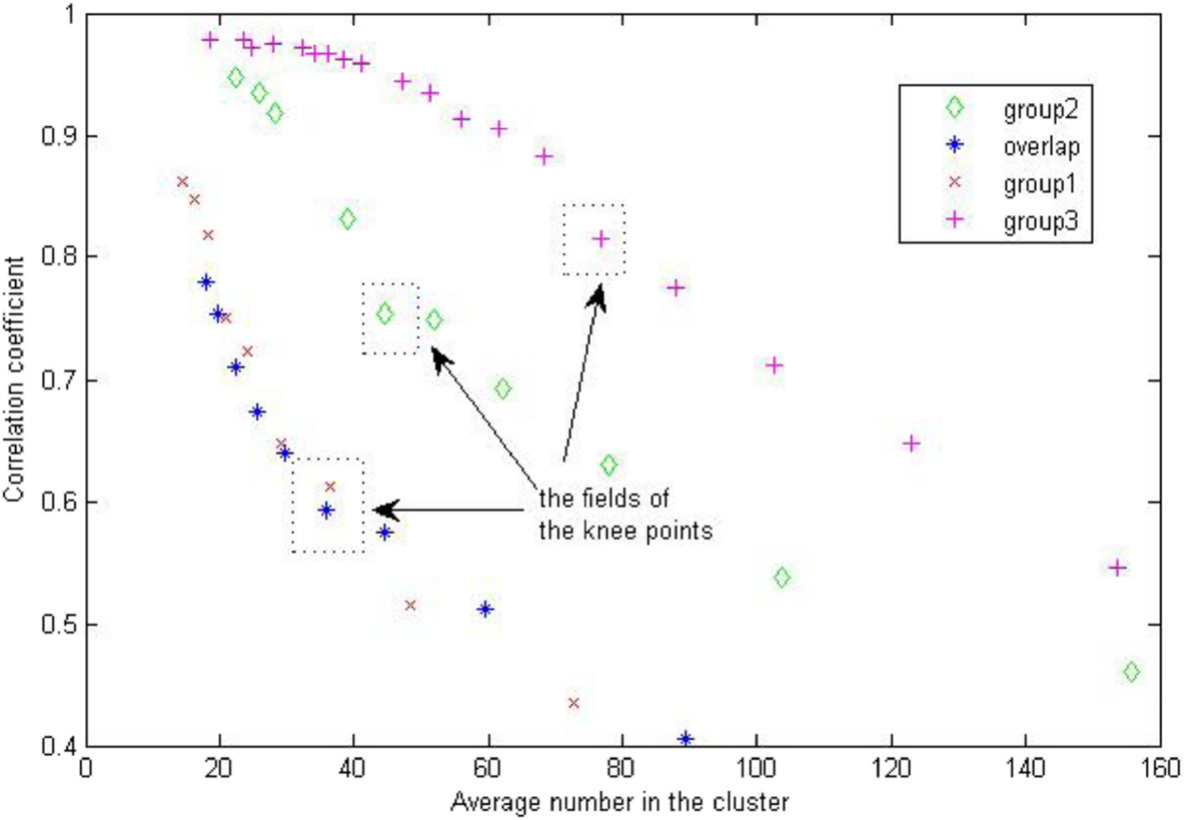
**Determination of number of clusters.** The initial cluster number for each group is determined based on the change trend between average correlation coefficient of expression values of genes in the same clusters and average size of clusters (i.e. number of genes in the cluster). The rectangle fields show the drastic change on correlation coefficients and cluster sizes. Here, group 1 represents the DEGs based on the comparison at 12 hours; group 2 represents the DEGs based on the comparison at 24 hours; group 3 represents the DEGs based on the comparison at 48 hours; overlap represents the genes differentially expressed based on the comparisons at all three time points.

In order to construct gene regulatory modules, we clustered each group of genes or their overlap described above into clusters. The key issues are to determine how many clusters there should be and what average size (number of genes) the clusters should have. We tested different numbers of clusters to do clustering and obtained a series of average correlation coefficients and average cluster sizes. Figure 3 illustrates the plots of average correlation coefficients against average cluster sizes for clustering on each list of genes. The number of clusters balancing both correlation coefficients and cluster sizes eventually chosen were denoted in the plots as well.

### Regulatory network prediction

**Figure 4:**
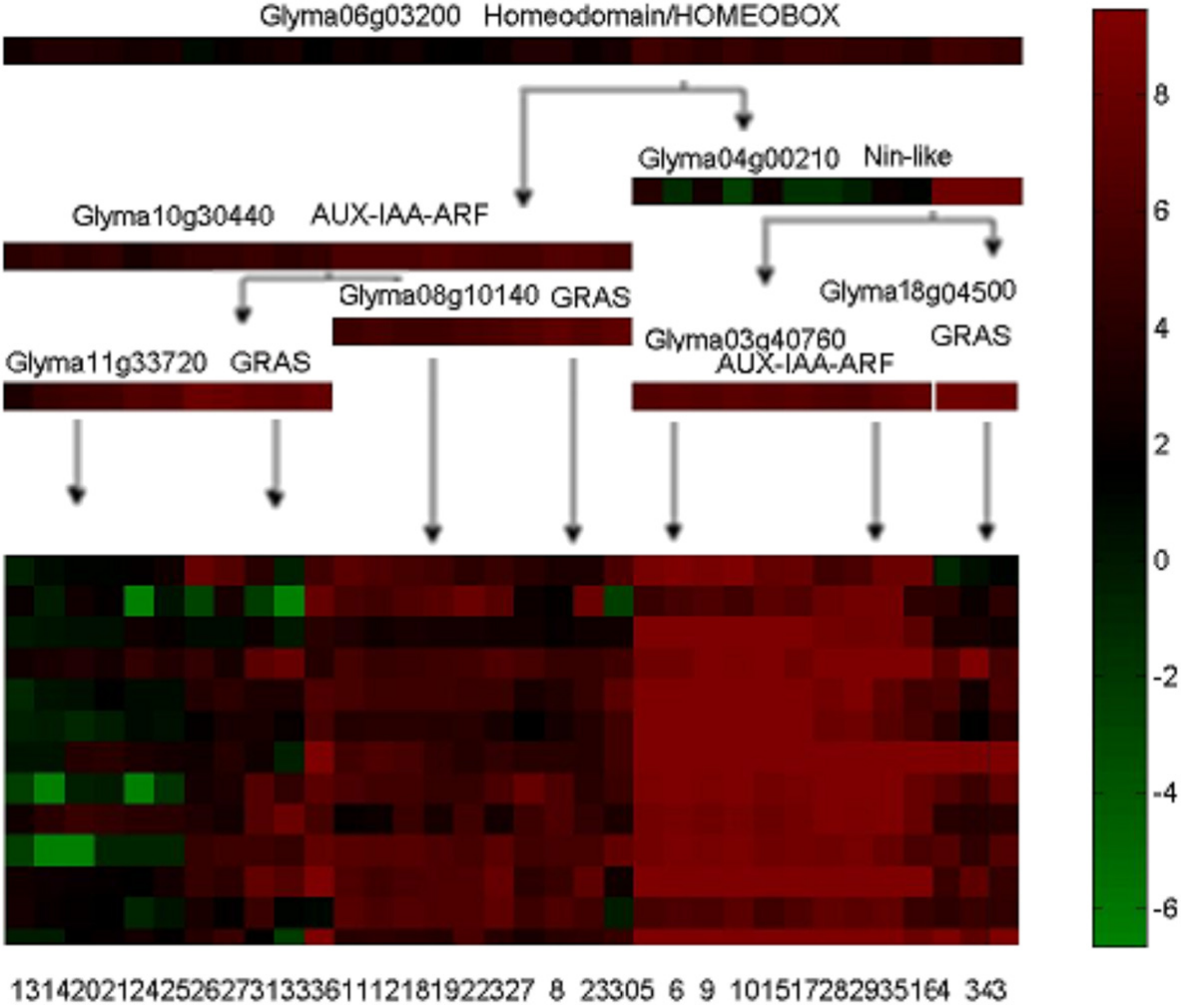
**Module 9 generated based on overlapped DEGs.** The tree-like upper part visualizes the predicted regulatory decision tree of the module, where a tree node (i.e. color bar) represents a query on the expression level of a predicted TF and a branch (i.e. arrowed edge) denotes a yes / no answer (highly / lowly expressed or not) to the query. The family names and gene IDs of the predicted TFs are listed above the nodes. A path from the top to a sub-set of conditions shown in the square box at the bottom predicts that the TFs on the path collaboratively regulate the expression of the genes in these conditions. For example, the right most path suggests that, if Homeodomain is highly expressed, Nin-like highly expressed, and GRAS highly expressed, the genes in the modules will be regulated in the nodulation conditions (i.e., conditions 34 and 3). The colored square box visualizes the expression levels of all genes in the module across all the conditions, where a row denotes a gene and a column denotes an experimental condition. The color bar on the right illustrates a specific color with an expression value, from the lowest (green) to the highest (red). The numbers at the bottom of the figure corresponds to the experimental conditions, which are described in details in the Additional file 1.

We chose the DEGs (354 genes) that were differentially expressed in all three time points of the rhizobial inoculation study to predict regulatory network modules. These genes were likely to be important since their expression predicts that they play a role through the nodulation formation process. Although chosen from the rhizobial inoculation experiment, the expression values of these genes under all 34 of the experimental conditions available were used to construct the regulatory network. Based on these DEGs, 10 modules were generated (see Part A in the Additional file 1). In the module 9 (see Figure 4), we found the regulator 'Glyma04g00210', a known transcriptional factor [10] that functions in nodulation. Another transcription factor, Glyma08g10140, significantly matches LOC_Os03g15680.1 (E-value = 3e-45) according to sequence alignment, which is a nodulation-signaling pathway 2 (NSP2) protein in the rice genome, in the transcription factor database [27]. The de novo prediction that these specific TF genes are involved in nodulation from our cluster analysis matches closely with the literature and gave us confidence that our methods did identify physiologically relevant regulatory modules.

We conducted functional analyses on the genes in the module to further validate the predicted relationships between TFs and their targets. We used MULTICOM-PDCN [23,25] to predict the functions of 13 genes in the module and then identified significantly enriched functions with p-values less than 0.01. Some functions, such as response to biotic stimulus, defense response, cis-zeatin O-beta-D-glucosyltransferase activity, and trans-zeatin O-beta-D-glucosyltransferase activity, were significantly enriched for this module (Table 1). These two glucosyl transferase enzymes are responsible for conjugating a glucosyl moiety to the cytokinin, zeatin [28]. Glucosyl- zeatin has been hypothesized to be significant in storage or transport [28]. This form of zeatin has also been shown to be resistant to degradation [29]. Cytokinins such as zeatin are involved in cortical cell division, which is vital in the formation of nodule organs in legume plant roots [30,31]. They have also been found to bind proteins including Lotus histidine kinase (LHK1) and *Medicago truncatula* cytokinin response (MtCRE) in the cortex inducing nodule organogenesis [32,33].

**Table 1:** The enriched functions of genes within the gene regulatory module 9

**GO term**	**Functions**	**P-value**
GO:0009607	P:response to biotic stimulus	6.74236E-04
GO:0010224	P:response to UV-B	2.32667E-02
GO:0006952	P:defense response	1.16894E-02
GO:0050502	F:cis-zeatin O-beta-D-glucosyltransferase activity	4.00410E-02
GO:0050403	F:trans-zeatin O-beta-D-glucosyltransferase activity	4.09650E-02
GO:0005199	F:structural constituent of cell wall	6.22214E-03
GO:0005618	C:cell wall	2.05644E-02

P-value is calculated based on the hypergeometric distribution. 27089 soybean genes were annotated by MULTICOM-PDCN [25] according to Gene Ontology function terms. The prefix *P*: biological process; *F*: molecular function; *C*: cellular component.

Moreover, we used MEME [34] and TomTom [35] to predict the TF binding sites of genes in the module. Two domain classes that were predicted are the BetabetaAlpha-zinc finger and the Leucine Zipper. The leucine zipper is a super-secondary structure that functions as a dimerization domain, which consists of multiple leucine residues at approximately 7-residue intervals. Interestingly, some TFs predicted for this module are in the GRAS family, which often possess the leucine heptad repeat (LHR) domains [36]. The matching between predicted TFs and DNA binding sites might indirectly support the prediction of the gene regulatory module.

Nodulation signaling protein (NSP) is a GRAS-like transcription factor comprised of a variable N- terminal domain and a highly conserved C-terminal domain [37]. NSP1 and NSP2 were first identified in the legume plant Medicago and found to be vital for changes in gene expression induced by Nod factor signaling [38,39]. The NSP transcription factors have also been identified as essential for nodule formation in another legume, Lotus japonicus [40]. Recently, soybean homologues of NSP1 and NSP2 have been shown to be regulated by root hair infection by Bradyrhizobium infection [11].

Additional file 2: Figure S1 illustrates the fifth gene regulatory module. This module contains 37 genes. According to the MULTICOM-PDCN function predictions, the significantly enriched functions include nodulation, gibberellic acid mediated signaling pathway, gibberellin 3-beta-dioxygenase activity, glutamate dehydrogenase [NAD (P)+] activity, and flavanone 4-reductase activity, which are directly or strongly related to nodular formation and development [41,42]. The DNA binding site analysis on the genes in the module predicted BetabetaAlpha-zinc finger, Stat, Homeo, and Helix-Loop-Helix leucine zipper domains. Among them, Stat and Leucine zipper are the typical domains of GRAS family [36,43,44] predicted for the module. Homeo domain is also consistent with the TF (MYB/HD-like, Homeodomain) predictions.

The supporting evidences of all the 10 modules are listed in the Table 2. These modules were assessed from four aspects: (1) function enrichment of genes in a module, (2) interaction potentials between TFs regulating a module predicted by STRING [45], (3) the goodness of fit between the motifs extracted from upstream of genes in a module and the annotated target motifs of the TFs regulating the module, and (4) literature confirmation of the regulatory function of TFs and the genes in corresponding experimental conditions. Most modules can be partially supported by the potential interactions among the predicted TFs within these modules predicted by STRING or by the possible match between the DNA binding motifs of the putative TFs and the conserved motifs in the upstream sequences of the genes in the modules. Some relationships between the nodulation development and the gene function of modules based on biological experiments were reported in the previous work [41,46-51].

**Table 2:** The 10 modules based on overlapping genes and some of their evidence supports

**Module**	Representative enriched biological process^*^	**Gene number**	**Coherence (%)**^**+**^	**I**^**$**^	**M&**	**L**^**#**^
1	gibberellic acid mediated signaling pathway	57	7		√	√ [32]
2	polyamine biosynthetic process	52	4	√		√ [33]
3	flavonoid biosynthetic process	48	6		√	√ [34]
4	cytokinin biosynthetic process	35	3		√	√ [35]
5	Nodulation	28	7	√	√	√
6	regulation of cytoskeleton organization	32	3	√		
7	pattern specification process	11	9	√		
8	response to stress	34	9		√	√ [36]
9	response to UV-B	13	8	√	√	√ [37]
10	nitrile biosynthetic process	44	2		√	√ [38]

Column 1 lists id of the module. Column 2* reports the representative enriched GO biological processes in these modules. Column 3 is the number of genes in the corresponding GO lists. Column 4 is the percent of genes in each module participating in the biological process, and ^**+**^ presents all of the GO biological processes in Table 2 with the significance *p* < 0.05. Column 5 (I^$^) shows if there are at least two predicted TFs in the module existing the interaction relation according to the STRING prediction and the BLAST homology search. Column 6 (M&) reports if the DNA binding motifs of some predicted TF families matched the motifs extracted from the upstream sequences of the regulated genes in the modules. Column 7 (L^#^) lists if previous literature had reported the relationship between the nodule development or formation and the gene function of the module.

### Incorporation of prior biological knowledge into network construction

Previous studies [52] implicated specific transcription factors as key regulators of nodule formation and development. According to [52], these key regulators belong to NIN-like, Bzip, GRAS, C2H2 (Zn), HomeoDomain and CCAAT families. In order to specifically incorporate this prior information into the gene regulatory network construction, we constructed gene regulatory modules based on these six pre-selected transcription factor families, resulting in 10 modules (see F1-F10 in Part B in the Additional file 1). For example, in module 9, 5 regulators belonging to these 6 family (i.e., Glyma06g03200, Glyma04g00210, Glyma11g33720, Glyma08g10140, Glyma18g04500) were predicted to regulate the module. This experiment demonstrated that prior biological knowledge could be incorporated into our gene regulatory network construction framework if necessary.

### Incorporation of non-differentially expressed TFs into network construction

Thus far, we have focused on constructing gene regulatory networks from differentially expressed genes. However, this approach may miss some TFs, critical to the biological process, but whose expression level does not change appreciably in the datasets available. To address this issue, we incorporated different percent of the non-differentially expressed transcription factors into the network construction. We then compared the modules constructed in these different scenarios in order to check how well the same TF-TF relation, TF-target relation, and gene-gene relation were preserved in these modules, i.e., how many pairs of these three relations mentioned above still can be predicted together. Here we use Rand index [45] to calculate the overlap of the three relations. Given a set of n elements *S* = {*O*_1_, *O*_2_, … *O*_*n*_} and two partitions of S to compare, *X* = {*x*_1_, *x*_2_, … *x*_*r*_} and *Y* = {*y*_1_, *y*_2_, … *y*_*s*_}, the Rand index is calculated as $R\;=\frac{a+b}{a+b+c+d}$, a, the number of pairs of relations in S that are in the same set in X and in the same set in Y; b, the number of pairs of relations in S that are in different sets in X and in different sets in Y; c, the number of pairs of relations in S that are in the same set in X and in different sets in Y; d, the number of pairs of relations in S that are in different sets in X and in the same set in Y. Additional file 2: Figure S2 reports how Rand indices change with different portions of non-differentially expressed TFs incorporated. It is shown that when the percent of non-differential expression transcription factors is under 10%, the predicted relations are relatively stable, i.e., most TFs and genes predicted for a module under random perturbation by introducing a small fraction of non-differentially expressed TFs into candidate TF lists are the same.

### Gene regulatory modules predicted for each stage of nodulation

The gene regulatory modules described above were constructed for the genes differentially expressed in all of the three different time points sampled during nodule formation. Similarly, we also constructed the gene regulatory modules for genes differentially expressed in a certain stage (12, 24, or 48 hours) in nodulation formation. In this case, the TF expression status (up, down, norm) was determined by comparing the expression level at the selected specific condition with the other conditions. We used the RNA-Seq data [11] generated under 14 experimental condition (corresponding to the number from 3 to 16: 12HA1_IN_RH, 12HA1_UN_RH, 24HA1_IN_RH, 24HA1_UN_RH, 48HA1_IN_RH, 48HA1_Scripped_Root, 48HA1_UN_RH, Green_Pods, Leaves, Nodule, Root, Root_Tip) to construct the modules for the genes differentially expressed in each of the three nodulation stages. All the modules predicted for the three stages are listed in Additional files 3, 4, and 5, respectively. By way of example, we describe one module in detail below.

**Figure 5:**
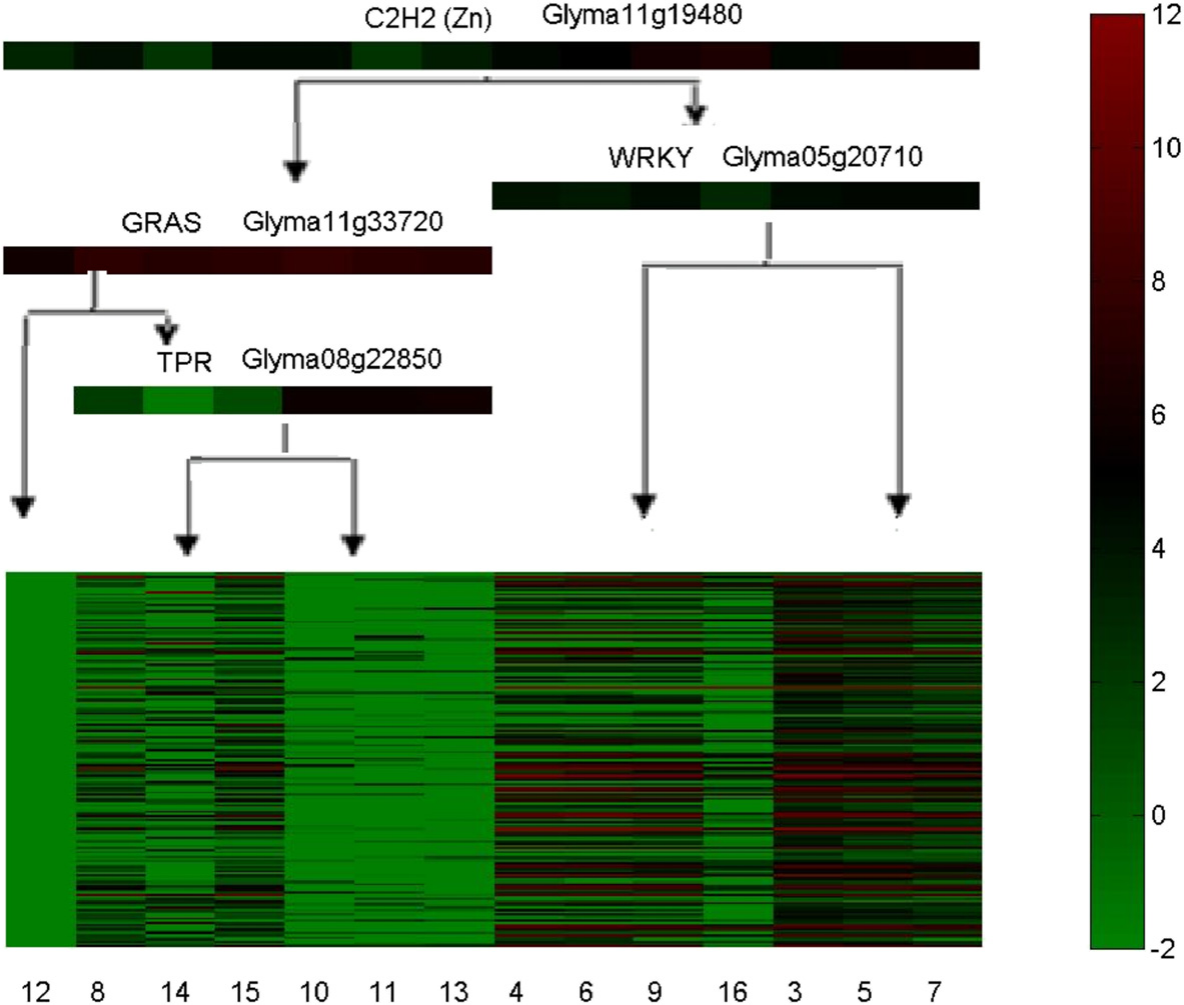
Module 41 generated based on DEGs identified from roots 24 hours after rhizobia inoculation.

Module 41 (Figure 5) is one module predicted from the data derived 24 hours after inoculation. This module contains 6 nodulation related genes: Glyma16g01020, Glyma18g02230, Glyma17g08110, Glyma02g36580, Glyma04g00210, Glyma07g04430 [10]. We predicted 4 transcription factors: Glyma11g33720, Glyma11g19480, Glyma05g20710, Glyma08g22850, which separately belong to GRAS, C2H2(ZN), WRKY and TPR families. With the binding site analysis, the four most significant TFs are, separately, the High Mobility Group, BetaBetaAlpha-zinc finger, TATA-binding and Leucine Zipper. Leucine Zipper domain is one of typical domains of GRAS family [36], and BetaBetaAlpha-zinc finger superfamily contains C2H2(ZN) family [53] (Table 3). The enriched functions include response to gibberellin stimulus, gibberellin biosynthetic process, and nodulation (Table 4). The prediction of GRAS family TFs for the module is largely consistent with their reported role in root and shoot development and gibberellic acid signaling [54], and in nodulation [55,56].

**Table 3:** The binding site analysis for the gene regulatory module 41 at nodulation stage of 24 hours

**TF family or domain by TOMTOM**	**p-value**	**Motif based on the genes**
High Mobility Group	3.29431E-05	CTTTTTTTCTCTTTTTTT
BetaBetaAlpha-zinc finger	4.82488E-05	CACCCACACACACAAACA
BetaBetaAlpha-zinc finger	4.89364E-05	CCCCCTCCACC
TATA-binding	7.80973E-05	TATATATATATATATATA
Leucine Zipper	9.65926E-04	GGGGGGCATCACGGTGGC

**Table 4:** The enriched functions of genes in the gene regulatory module 41 at nodulation stage of 24 hours

**GO term**	**Functions**	**P-value**
GO:0042545	P:cell wall modification	7.76836E-07
GO:0009639	P:response to red or far red light	1.46641E-03
GO:0006032	P:chitin catabolic process	7.70352E-05
GO:0042744	P:hydrogen peroxide catabolic process	2.11711E-05
GO:0007047	P:cellular cell wall organization	2.64477E-06
GO:0009739	P:response to gibberellin stimulus	2.07483E-03
GO:0006949	P:syncytium formation	8.69996E-04
GO:0009686	P:gibberellin biosynthetic process	7.38468E-03
GO:0006073	P:cellular glucan metabolic process	3.89541E-02
GO:0009607	P:response to biotic stimulus	1.94115E-03
GO:0009877	P:nodulation	4.04420E-02
GO:0006952	P:defense response	7.89696E-03
GO:0009820	P:alkaloid metabolic process	1.51887E-02

P-value is calculated based on the hypergeometric distribution. The prefix *P*: biological process; *F*: molecular function; *C*: cellular component.

### Module evaluation

Similar with Joshi et al. [57], we used a random experiment and probability distribution to assess the reliability of the modules. For a predicted module, we randomly re-assigned the same number of genes to form the TF regulatory tree of the same topology to generate a random tree and repeated the process1000 times.

**Figure 6:**
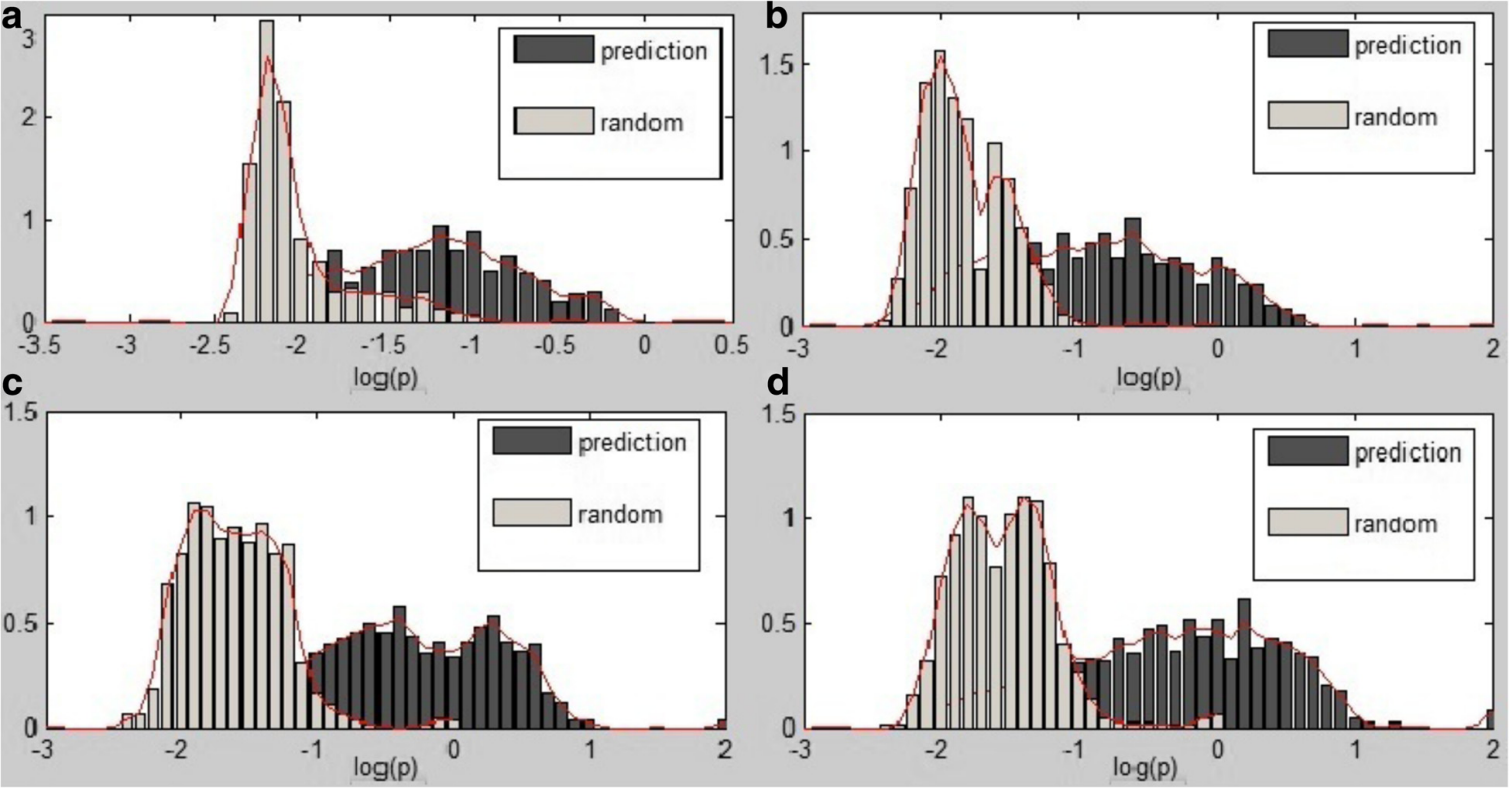
**Evaluation of Predicted Modules (or Models). (a)** Histogram of log(*p*) (i.e. log-likelihood) of predicted modules and random modules based on overlap DEGs; **(b)** Histogram of log(*p*) of predicted modules and random modules based on group1 DEGs; **(c)** Histogram of log(*p*) of predicted modules and random modules based on group2 DEGs; **(d)** Histogram of log(*p*) of predicted modules and random modules based on group3 DEGs. All histograms are normalized to have an area equal to 1.

Assuming that a regulatory tree divided experimental conditions into a set of sub-groups - *S* = {*S*_1_, *S*_2_, … *S*_*s*_} and the mean and standard deviation of the gene expression values in a sub-group *S*_*k*_ were μ_k_ and σ_k_, respectively. The genes within a module *M* = {*g*_1_, *g*_2_, … *g*_*n*_} under certain condition were assigned to the sub-group yielding a probability score: $\text{log}\;\left(p_{j}\right)\sim \sum_{i=1}^{n}-\frac{{\left(x_{\text{ij}}-\mu_{k}\right)}^{2}}{2\sigma_{k}{}^{2}}-\text{ln}\left(\sigma_{k}\right)$, where x_ij_ was the expression value of *g*_*i*_ under condition j. The *log*(*p*_*j*_) values (a measure of likelihood of data under the module) of modules predicted by our algorithm and generated by random experiments have the different distributions in Figure 6 (a). A higher value of *log*(*p*_*j*_) suggests that a module predict the expression values of the genes better.

The average of *log*(*p*_*j*_) for 10 predicted modules under all conditions is −1.2062 and the range of averages for the random modules generated by 1000 random experiments is [−2.0703, -1.9758]. The range of the standard deviations of *log*(*p*_*j*_) for the random modules generated by 1000 random experiments is [0.2575, 0.3533], whereas the standard deviation for the predicted modules is 0.4667. The data show that our method reconstructs the gene regulatory modules with substantially high *log*(*p*_*j*_) (i.e. likelihood), which suggests the more accurate prediction of the outcome of experiments [57].

In order to investigate the robustness of the method with respect to the thresholds of selecting differentially expressed genes, we used 0.0001 p-value threshold rather than the common threshold 0.05 to select overlap DEG genes in all three nodulation stages for gene regulatory network construction. The results showed that the gene regulatory modules constructed under 0.0001 threshold were smaller due to a small size of DEG genes, but almost completely overlapped with the large modules constructed under threshold 0.05 (data not shown). The experiment suggests that our gene regulatory construction process is rather stable against the threshold of selecting differentially expressed genes.

## Conclusions

In this work, we focus on inferring the gene regulatory modules related to soybean nodule development and formation from RNA-Seq transcriptome data. Our method was able to construct gene regulatory networks for differentially expressed genes in a number of biological conditions. The method can also incorporate non-differentially expressed TFs or pre-selected TFs into network construction and predict their target genes. Some of predicted TF-gene relationships were validated by DNA binding site analysis, gene function enrichment analysis and previous research. Furthermore, the gene regulatory network prediction clearly also identified TFs not previously shown to play a role in nodulation and, therefore, should stimulate research to explore their function. For example, in some modules, TFs belonging to the AUX-IAA-ARF family were predicted, which may be consistent with previous work [58] reporting that hormones (e.g. auxin and cytokinin) play a role in nodule formation. NODCON1GM (sequence pattern: AAAGAT) and NODCON2GM (sequence pattern: CTCTT) were two putative nodulin consensus sequences investigated in [59]. We searched these two patterns in the up-stream sequences of all predicted gene modules and the results were reported in the Additional files 1, 3, 4 and 5.

In addition to being applied to the soybean RNA-Seq data in this case study, the method can be similarly employed to analyzing the RNA-Seq data of any other species. With the large amount of RNA-Seq data being produced for many species under various biological conditions, our method should become a useful tool to infer gene regulatory logic from these data at a systems level. The predicted regulatory relationships can be used to generate hypotheses for designing biological experiments.

## Competing interests

The authors declare that they have no competing interests.

## Authors’ contributions

JC and MZ conceived the project. JC and MZ designed the method and experiment. MZ implemented the methods and carried out the experiment. MZ and JC analyzed the data. JD contributed to the biological discussion of the results. GS and JD contributed to the generation of some datasets and aided in providing a biological context for the results. MZ and JC wrote the manuscript. All of the authors edited and approved the manuscript.

## Supplementary Material

Additional file 1 **Modules based on overlapping DEGs.** Part A: Module 1–10 generated based on overlapped genes with all included TF families. Part B: Module F1-F10 generated based on overlapped genes with pre-selected six families (NIN like, Bzip, GRAS, C2H2 (Zn), HomeoDomain and CCAAT). Click here for file

Additional file 2 Figure S1 Module 5 generated based on the overlapped DEGs; **Figure S2.** Module stability after incorporating different percent of non-differential expressed TFs. Click here for file

Additional file 3 Modules generated based on the 12-hour DEGs. Click here for file

Additional file 4 Modules generated based on the 24-hour DEGs. Click here for file

Additional file 5 Modules generated based on the 48-hour DEGs. Click here for file
