# Supplementary material for: Predicting gene regulatory networks of soybean nodulation from RNA-Seq transcriptome data
Source: BMC Bioinformatics. 2013 Sep 22;14:278. doi: 10.1186/1471-2105-14-278 (PMC3854569; doi:10.1186/1471-2105-14-278)
Supplement: Additional file 1 — Modules based on overlapping DEGs. Part A: Module 1–10 generated based on overlapped genes with all included TF families. Part B: Module F1-F10 generated based on overlapped genes with pre-selected six families (NIN like, Bzip, GRAS, C2H2 (Zn), HomeoDomain and CCAAT). [file 1471-2105-14-278-S1.doc]

**Supplemental Materials**

**Part A**

**Module 1-10 are generated based on overlapping genes with all included TF families.** The numbers at the bottom of each figure correspond to the experimental conditions:

3)10A_nodule

4)10B_nodule

5)12HA1_IN_RH

6)12HA1_UN_RH

7)1A_flower

8)1B_flower

9)24HA1_IN_RH

10)24HA1_UN_RH

11)2A_cm_pod

12)2B_cm_pod

13)3A_-2_seed

14)3B_-2_seed

15)48HA1_IN_RH

16)48HA1_Scrip_Root

17)48HA1_UN_RH

18)4A_-2_shell

19)4B_-2_shell

20)5A_-1_seed

21)5B_-1_seed

22)6A_-1_shell

23)6B_-1_shell

24)7A_0_seed

25)7B_0_seed

26)8A_young_leaf

27)8B_young_leaf

28)9A_root

29)9B_root

30)Apical_Meristem_Stacey

31)Flower_Stacey

32)Green_Pods_Stacey

33)Leaves_Stacey

34)Nodule_Stacey

35)Root_Stacey

36)Root_Tip_Stacey

The modules are listed one by one in the following order: the figure of the module, the enrichment functions in Biological Process, the genes in the module.

**Module 1**


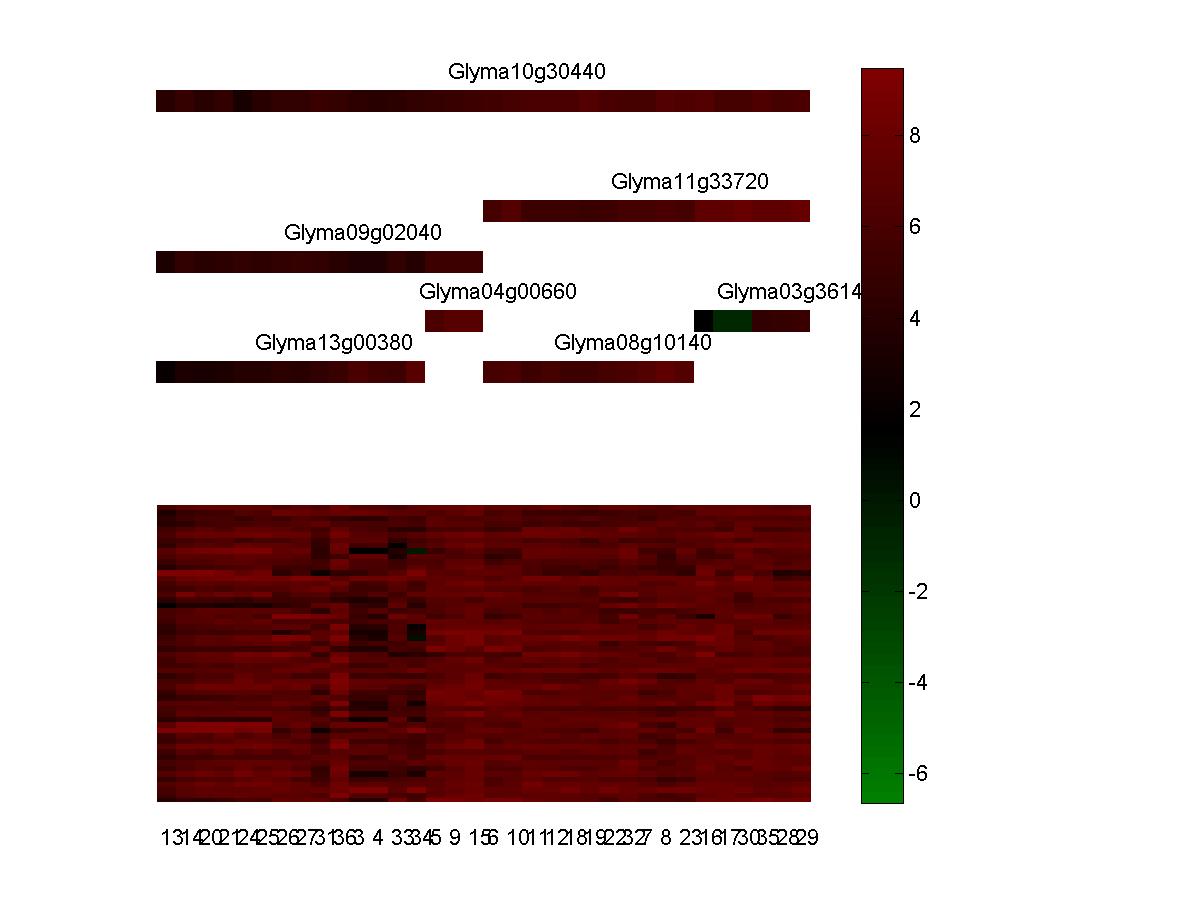


| GO ACC | FUNCTION |
| --- | --- |
| GO:0007018 | P:microtubule-based movement |
| GO:0009740 | P:gibberellic acid mediated signaling pathway |
| GO:0006412 | P:translation |
| GO:0051258 | P:protein polymerization |

Glyma08g03150 Glyma11g33720 Glyma05g08670 Glyma08g10140 Glyma08g05820 Glyma06g20540

Glyma12g04020 Glyma04g09350 Glyma04g00450 Glyma01g01310 Glyma07g15320 Glyma04g09820

Glyma02g43470 Glyma11g12500 Glyma13g06390 Glyma05g27180 Glyma20g05560 Glyma13g17220

Glyma18g00500 Glyma19g06460 Glyma14g09510 Glyma20g29660 Glyma06g02650 Glyma12g29510

Glyma13g22940 Glyma04g06700 Glyma10g40870 Glyma17g11940 Glyma14g06170 Glyma05g27190

Glyma08g14130 Glyma03g37340 Glyma17g34920 Glyma16g24120 Glyma19g32990 Glyma02g40290

Glyma10g35520 Glyma04g03110 Glyma05g01180 Glyma03g40760 Glyma17g17850 Glyma14g05510

Glyma04g00660 Glyma02g43080 Glyma14g06630 Glyma04g00650 Glyma0169s00210Glyma05g36420

Glyma05g21820 Glyma10g40150 Glyma04g40430 Glyma07g04890 Glyma06g00990 Glyma05g34570

Glyma10g06600

**Module 2**


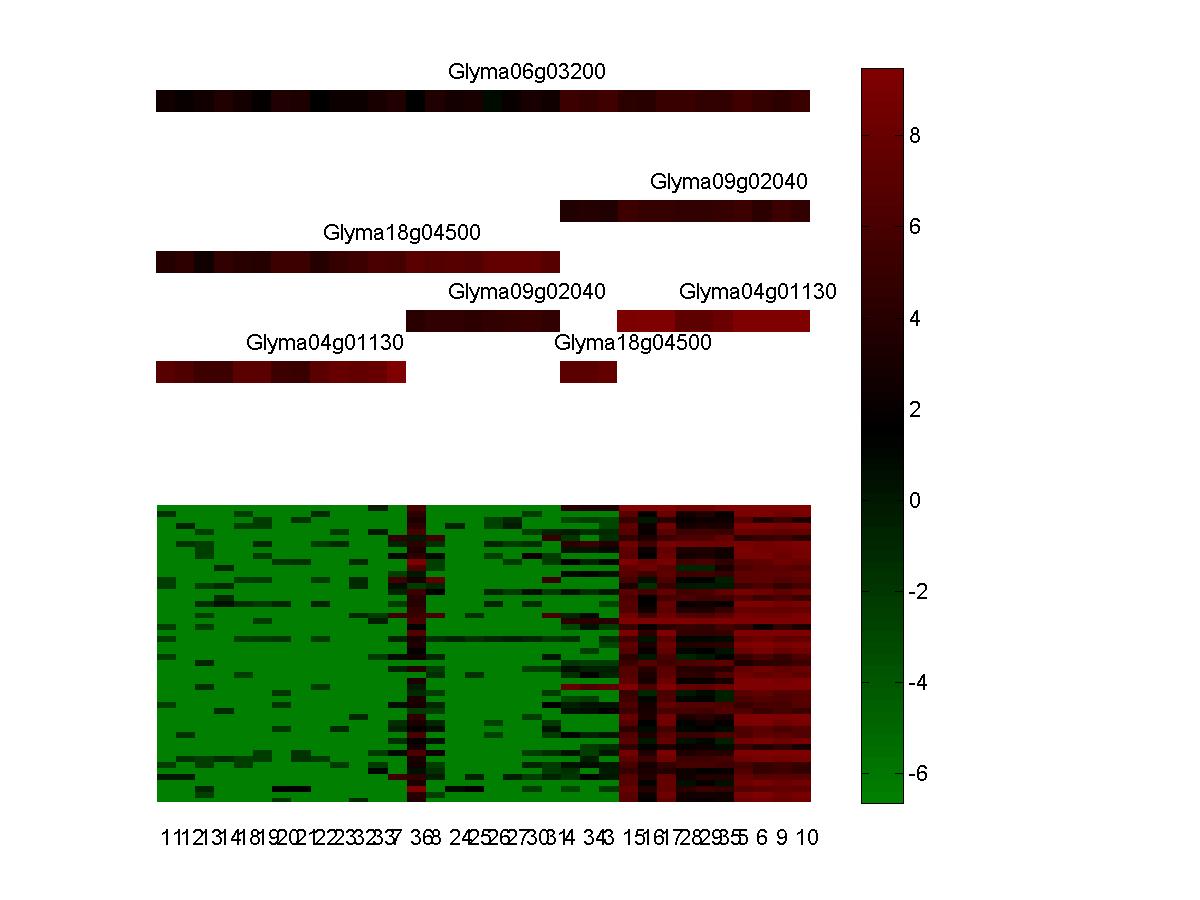


| GO ACC | FUNCTION |
| --- | --- |
| GO:0007047 | P:cellular cell wall organization |
| GO:0055114 | P:oxidation reduction |
| GO:0009664 | P:plant-type cell wall organization |
| GO:0042744 | P:hydrogen peroxide catabolic process |
| GO:0042545 | P:cell wall modification |
| GO:0006596 | P:polyamine biosynthetic process |

Glyma08g24770 Glyma02g01970 Glyma13g22350 Glyma18g02870 Glyma19g40960 Glyma02g09210 Glyma19g45260 Glyma11g29920 Glyma12g03050 Glyma06g02290 Glyma07g04340 Glyma05g25450 Glyma13g27130 Glyma15g11700 Glyma09g02610 Glyma10g02730 Glyma11g35560 Glyma08g43550 Glyma10g05800 Glyma08g24760 Glyma09g28800 Glyma03g04880 Glyma15g02380 Glyma01g42370 Glyma09g32630 Glyma15g35410 Glyma16g07830 Glyma11g03310 Glyma02g40010 Glyma15g35390 Glyma04g35130 Glyma01g34770 Glyma11g06070 Glyma15g07700 Glyma16g06520 Glyma17g14230 Glyma02g16800 Glyma06g47190 Glyma18g53740 Glyma10g29150 Glyma10g31280 Glyma10g02090 Glyma15g05760 Glyma07g17170 Glyma03g37390 Glyma17g15690 Glyma09g00850 Glyma04g02230 Glyma03g37400 Glyma08g17300

**Module 3**


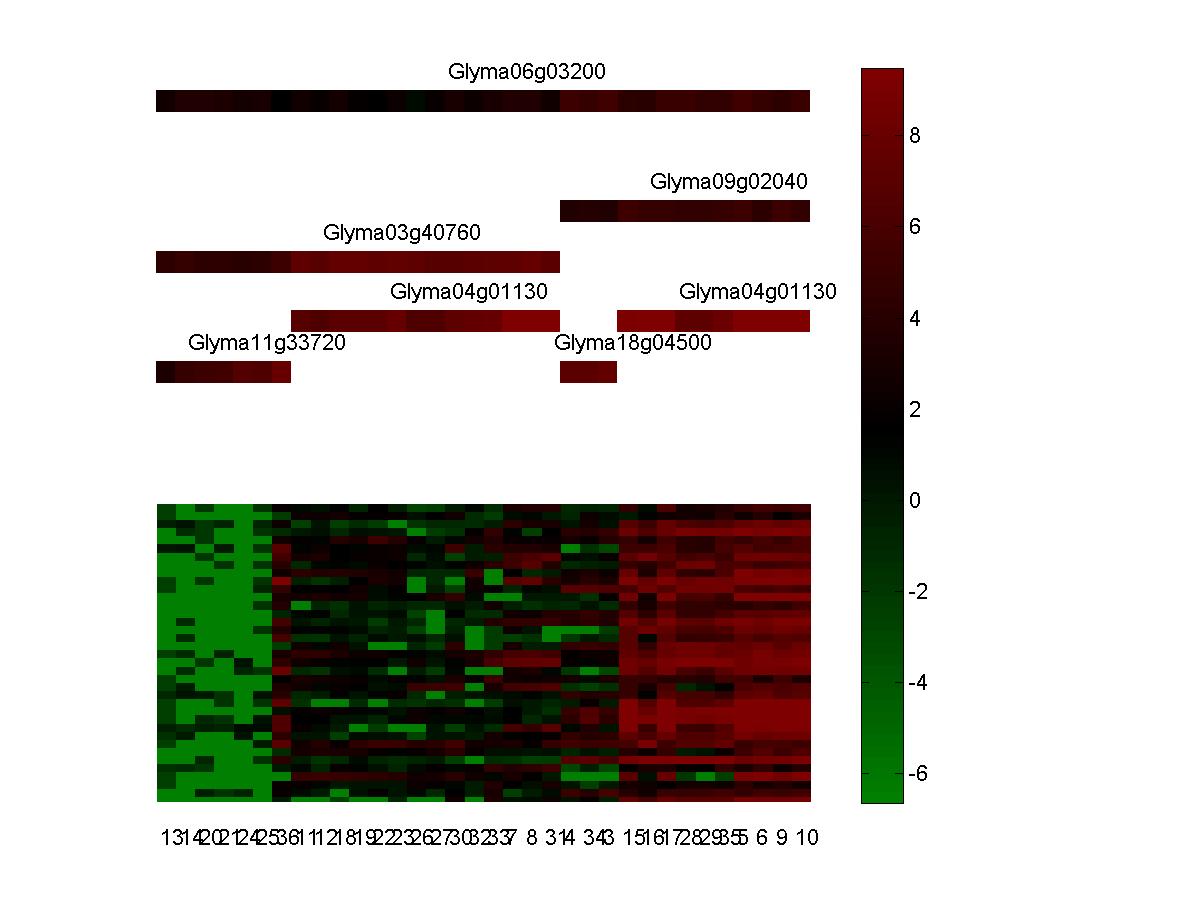


| GO ACC | FUNCTION |
| --- | --- |
| GO:0006952 | P:defense response |
| GO:0005975 | P:carbohydrate metabolic process |
| GO:0055114 | P:oxidation reduction |
| GO:0009813 | P:flavonoid biosynthetic process |

Glyma07g38580 Glyma12g36360 Glyma10g33650 Glyma01g31750 Glyma15g41700 Glyma01g42670 Glyma09g37910 Glyma11g03430 Glyma11g05800 Glyma16g27900 Glyma09g04530 Glyma12g06110 Glyma17g34590 Glyma03g02410 Glyma20g35630 Glyma15g15200 Glyma20g38590 Glyma03g03460 Glyma16g28590 Glyma02g09220 Glyma08g24720 Glyma18g50180 Glyma20g11610 Glyma16g01960 Glyma07g37270 Glyma09g05440 Glyma13g32300 Glyma17g02600 Glyma17g03340 Glyma03g28850 Glyma20g28490 Glyma12g02240 Glyma15g09540 Glyma03g24020 Glyma04g02750 Glyma01g04380 Glyma09g31110

**Module 4**


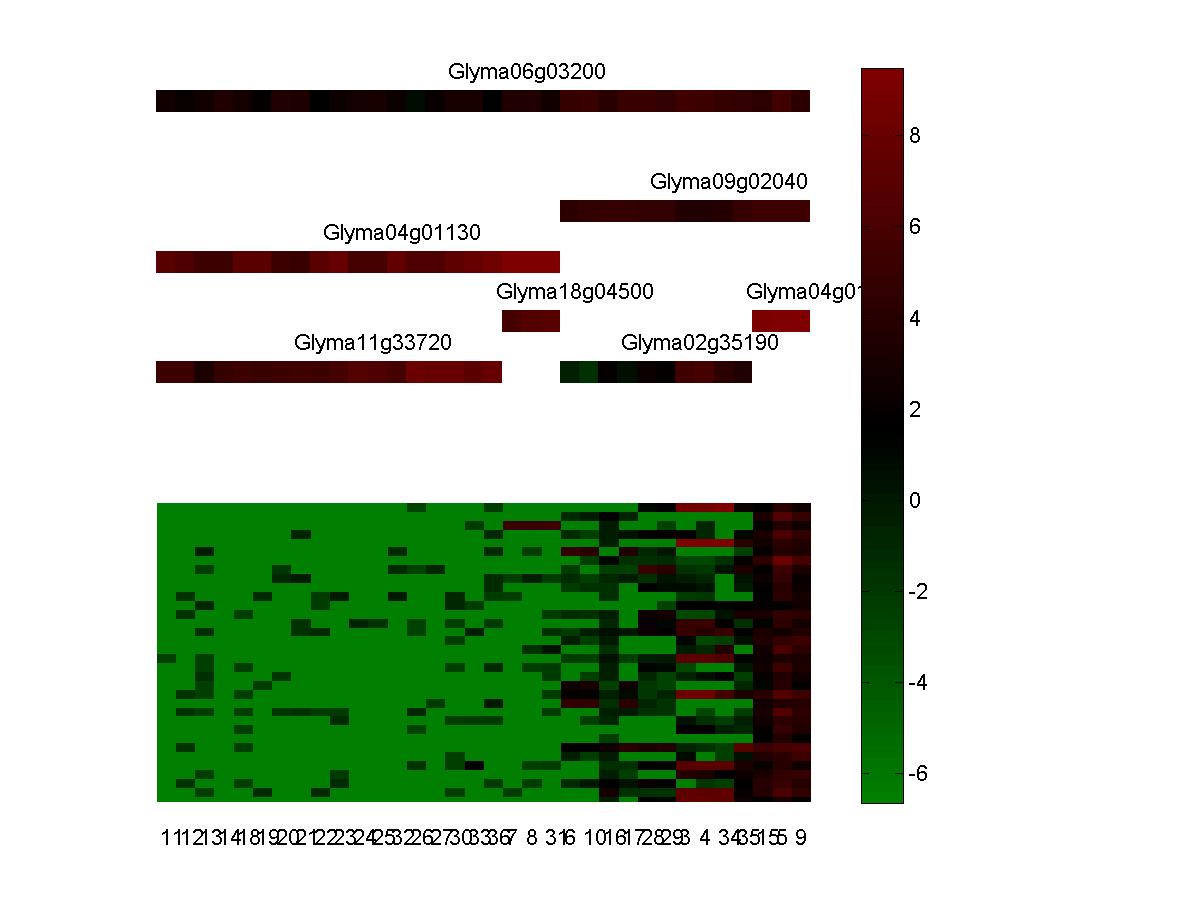


| GO ACC | FUNCTION |
| --- | --- |
| GO:0009877 | P:nodulation |
| GO:0006949 | P:syncytium formation |
| GO:0010114 | P:response to red light |
| GO:0009686 | P:gibberellin biosynthetic process |
| GO:0009567 | P:double fertilization forming a zygote and e... |
| GO:0007096 | P:regulation of exit from mitosis |
| GO:0000753 | P:cell morphogenesis involved in conjugation ... |
| GO:0045184 | P:establishment of protein localization |
| GO:0007118 | P:budding cell apical bud growth |
| GO:0009739 | P:response to gibberellin stimulus |
| GO:0009639 | P:response to red or far red light |
| GO:0030010 | P:establishment of cell polarity |
| GO:0009691 | P:cytokinin biosynthetic process |
| GO:0007124 | P:pseudohyphal growth |
| GO:0000750 | P:pheromone-dependent signal transduction inv... |
| GO:0001403 | P:invasive growth in response to glucose limi... |
| GO:0007119 | P:budding cell isotropic bud growth |
| GO:0006033 | P:chitin localization |

Glyma08g08170 Glyma01g45280 Glyma08g19250 Glyma05g12090 Glyma09g31910 Glyma07g37260

Glyma20g34830 Glyma06g29670 Glyma10g42840 Glyma05g12100 Glyma11g37620 Glyma08g17270

Glyma18g06350 Glyma20g29210 Glyma07g38620 Glyma10g25120 Glyma10g32070 Glyma13g27300

Glyma17g07440 Glyma09g27600 Glyma18g16790 Glyma02g36580 Glyma05g03720 Glyma08g05850

Glyma06g47690 Glyma01g38040 Glyma04g42300 Glyma16g06500 Glyma10g25130 Glyma10g10240

Glyma13g23770 Glyma17g02080 Glyma02g48080 Glyma08g24680

**Module 5**


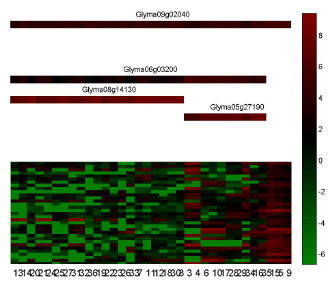


| GO ACC | FUNCTION |
| --- | --- |
| GO:0006548 | P:histidine catabolic process |
| GO:0006325 | P:chromatin organization |
| GO:0010114 | P:response to red light |
| GO:0009686 | P:gibberellin biosynthetic process |
| GO:0009646 | P:response to absence of light |
| GO:0009567 | P:double fertilization forming a zygote and e… |
| GO:0042744 | P:hydrogen peroxide catabolic process |
| GO:0009877 | P:nodulation |
| GO:0042631 | P:cellular response to water deprivation |
| GO:0009642 | P:response to light intensity |
| GO:0070896 | P:positive regulation of transposon integration |
| GO:0070898 | P:RNA polymerase III transcriptional preiniti… |

Glyma02g04180 Glyma08g48030 Glyma07g09710 Glyma10g28610 Glyma20g30450 Glyma09g37910 Glyma15g01500 Glyma13g38710 Glyma16g26940 Glyma08g48240 Glyma04g00210 Glyma08g03330 Glyma20g29200 Glyma08g23310 Glyma16g06740 Glyma16g28610 Glyma20g32470 Glyma02g35190 Glyma14g05840 Glyma19g38800 Glyma03g36140 Glyma03g28080 Glyma02g40890 Glyma16g04980 Glyma01g42800 Glyma09g05340 Glyma16g27440 Glyma01g03470 Glyma16g22920 Glyma05g03750 Glyma08g12650 Glyma03g02580 Glyma11g14060 Glyma02g42730 Glyma05g28810 Glyma06g12010 Glyma19g44060

**Module 6**


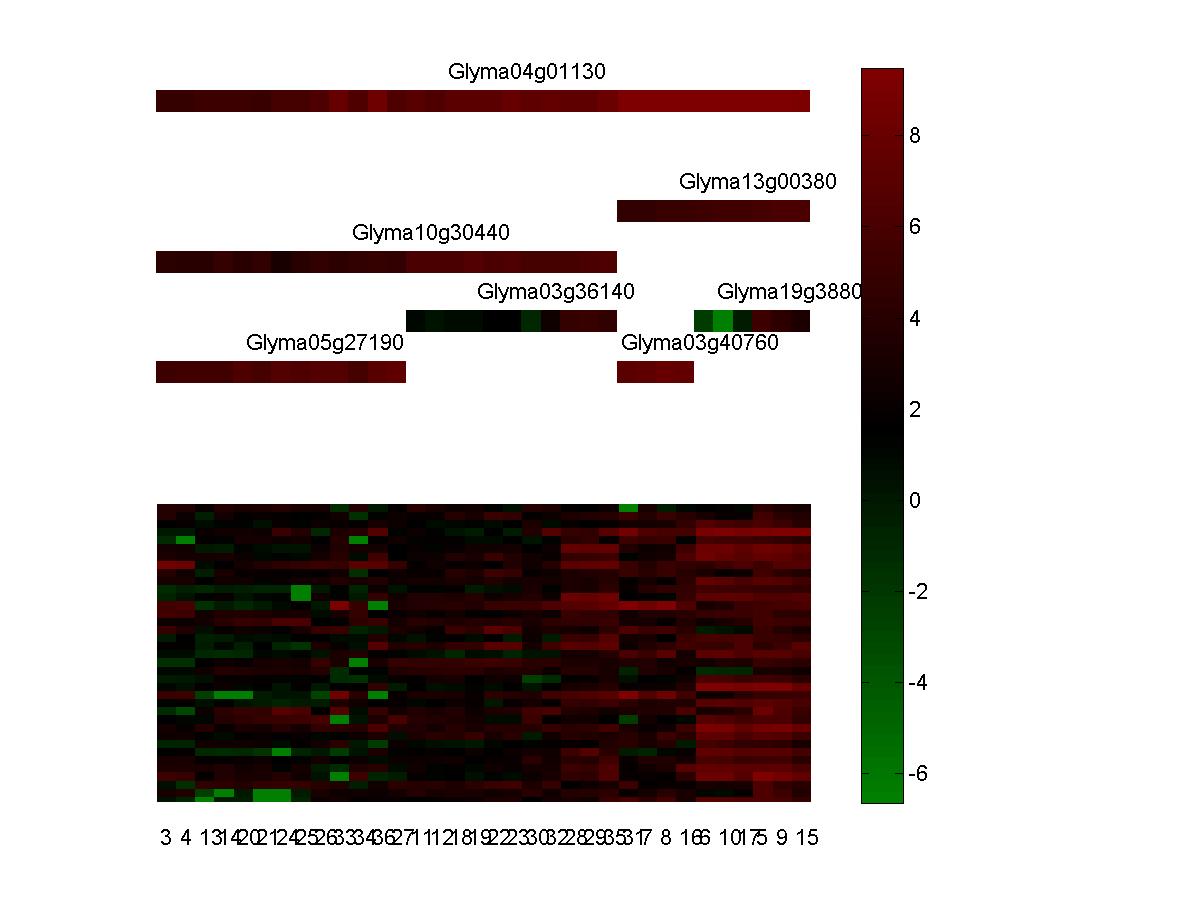


| GO ACC | FUNCTION |
| --- | --- |
| GO:0007186 | P:G-protein coupled receptor protein signalin... |
| GO:0050665 | P:hydrogen peroxide biosynthetic process |
| GO:0050896 | P:response to stimulus |
| GO:0048511 | P:rhythmic process |
| GO:0007050 | P:cell cycle arrest |
| GO:0007608 | P:sensory perception of smell |
| GO:0008202 | P:steroid metabolic process |
| GO:0009698 | P:phenylpropanoid metabolic process |
| GO:0006559 | P:L-phenylalanine catabolic process |
| GO:0030334 | P:regulation of cell migration |
| GO:0015696 | P:ammonium transport |
| GO:0009236 | P:cobalamin biosynthetic process |
| GO:0007548 | P:sex differentiation |
| GO:0019953 | P:sexual reproduction |
| GO:0006725 | P:cellular aromatic compound metabolic process |
| GO:0045815 | P:positive regulation of gene expression, epi... |
| GO:0051493 | P:regulation of cytoskeleton organization |

Glyma12g02590 Glyma14g06650 Glyma15g14790 Glyma07g38110 Glyma12g36300 Glyma08g11960

Glyma09g28490 Glyma15g42780 Glyma16g29370 Glyma14g06640 Glyma08g45920 Glyma04g33010

Glyma19g01940 Glyma08g43330 Glyma06g03200 Glyma13g44870 Glyma02g42250 Glyma07g16850

Glyma12g32160 Glyma03g19260 Glyma02g11720 Glyma10g07500 Glyma08g46610 Glyma19g37240

Glyma08g43340 Glyma04g42240 Glyma17g04940 Glyma06g05530 Glyma10g29260 Glyma15g41970

Glyma10g38080 Glyma11g14130 Glyma20g32140 Glyma02g08950 Glyma01g39460 Glyma12g33070

Glyma17g17310

**Module 7**


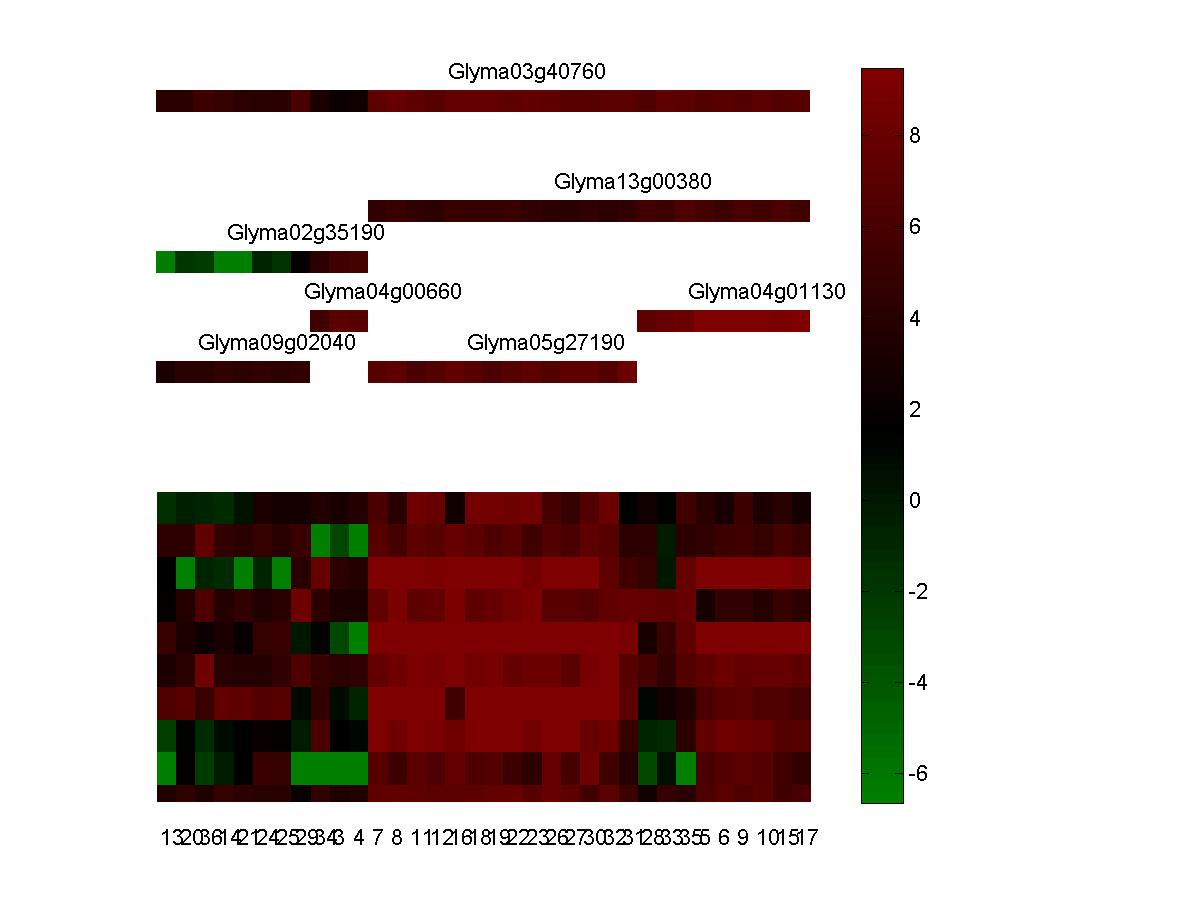


| GO ACC | FUNCTION |
| --- | --- |
| GO:0007047 | P:cellular cell wall organization |
| GO:0030245 | P:cellulose catabolic process |
| GO:0006811 | P:ion transport |
| GO:0009624 | P:response to nematode |
| GO:0042744 | P:hydrogen peroxide catabolic process |
| GO:0007389 | P:pattern specification process |
| GO:0031408 | P:oxylipin biosynthetic process |

Glyma14g05850 Glyma02g01990 Glyma08g21410 Glyma09g33750 Glyma07g01730 Glyma08g14550

Glyma13g42340 Glyma02g18090 Glyma08g45610 Glyma13g39600

**Module 8**


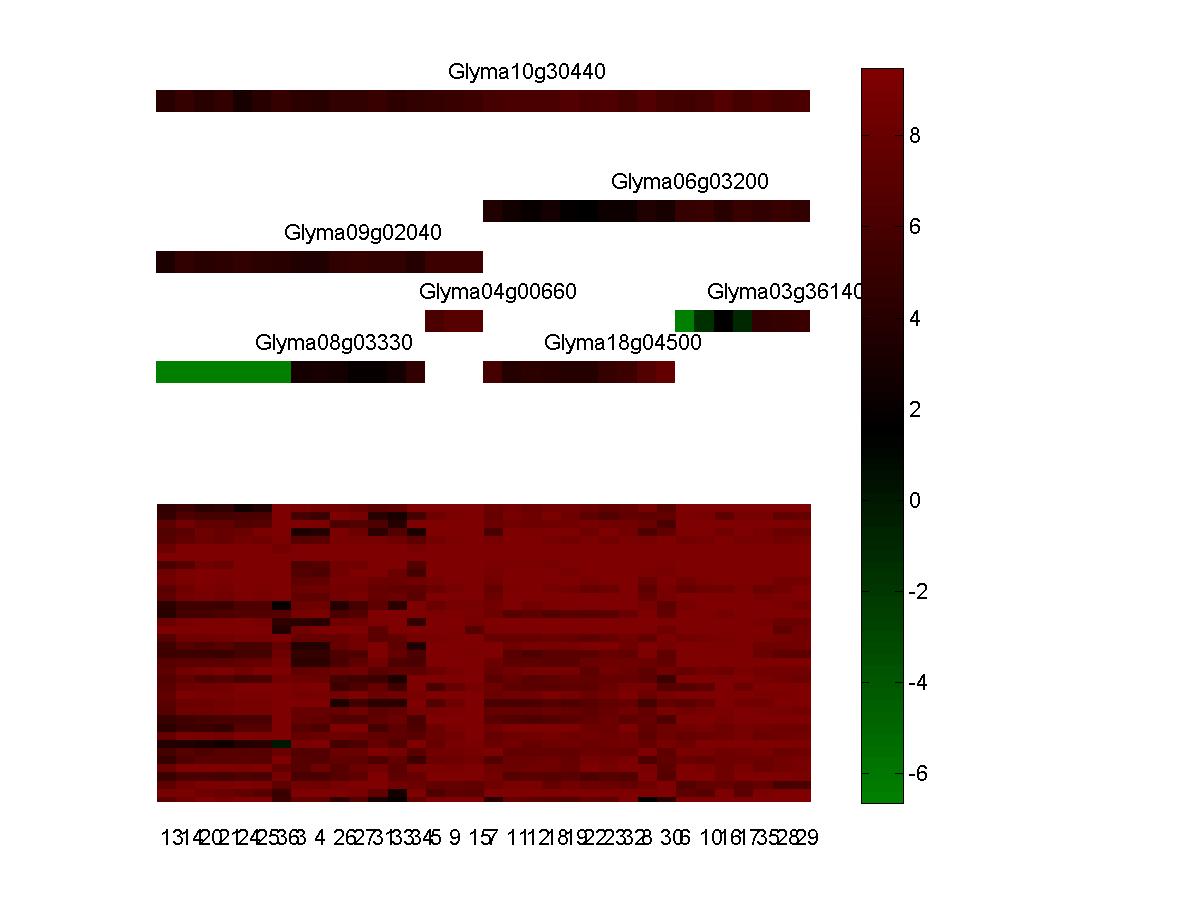


| GO ACC | FUNCTION |
| --- | --- |
| GO:0006950 | P:response to stress |
| GO:0055085 | P:transmembrane transport |
| GO:0006414 | P:translational elongation |

Glyma13g42330 Glyma16g33710 Glyma07g00900 Glyma05g37730 Glyma12g02790 Glyma10g39780

Glyma17g23900 Glyma03g34310 Glyma19g37000 Glyma05g11630 Glyma10g05580 Glyma08g11480

Glyma12g34550 Glyma15g19580 Glyma19g29210 Glyma19g36440 Glyma19g35570 Glyma16g04190

Glyma04g01130 Glyma13g40100 Glyma07g34440 Glyma07g00910 Glyma09g01320 Glyma03g32850

Glyma15g12170 Glyma19g35560 Glyma02g04510 Glyma08g46520 Glyma05g24110 Glyma12g34570

Glyma14g09440 Glyma08g11490 Glyma17g23870 Glyma12g00390 Glyma11g12510 Glyma10g35870

Glyma09g12200

**Module 9**


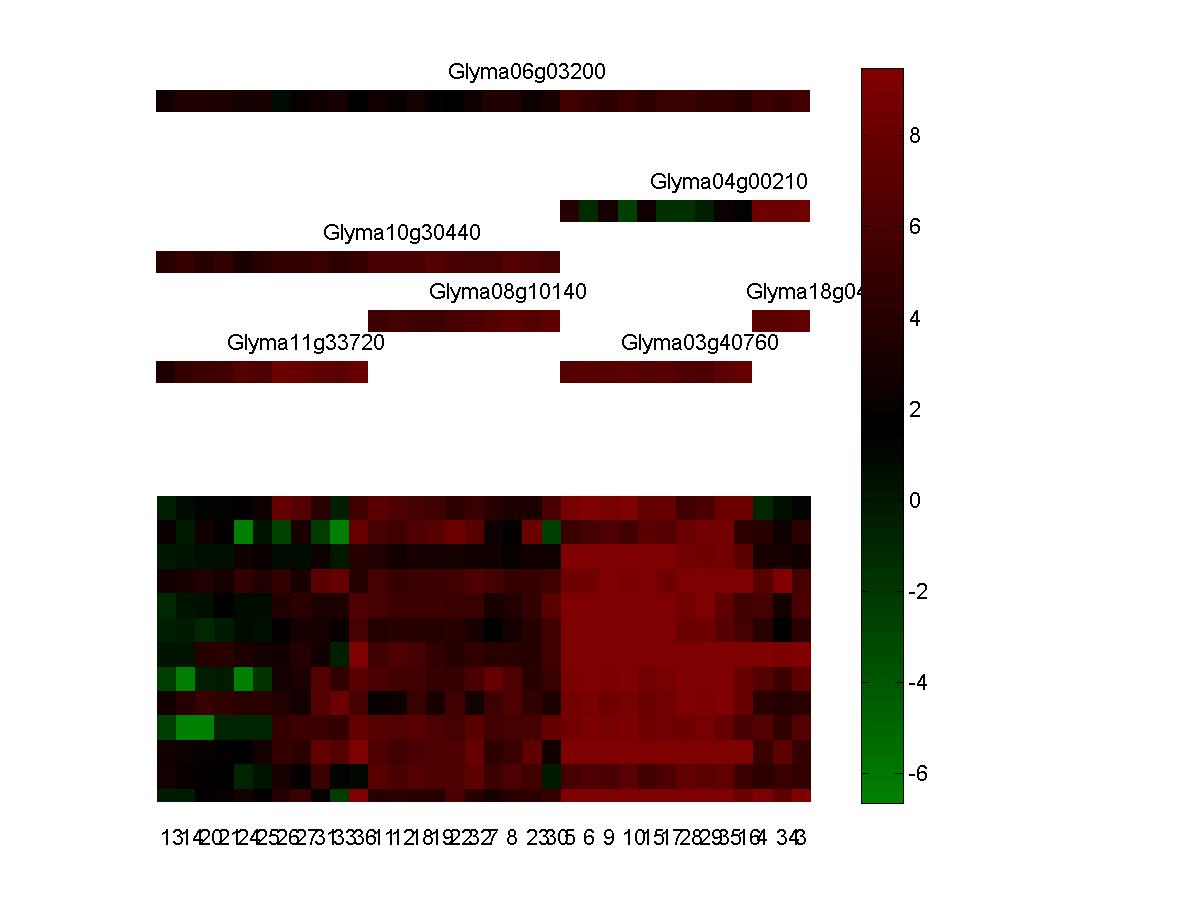


| GO ACC | FUNCTION |
| --- | --- |
| GO:0007275 | P:multicellular organismal development |
| GO:0008152 | P:metabolic process |
| GO:0010224 | P:response to UV-B |
| GO:0006869 | P:lipid transport |
| GO:0042744 | P:hydrogen peroxide catabolic process |
| GO:0009607 | P:response to biotic stimulus |

Glyma09g02600 Glyma05g04440 Glyma17g03350 Glyma17g34870 Glyma12g06100 Glyma11g14140

Glyma10g35080 Glyma16g28600 Glyma11g00230 Glyma02g09200 Glyma11g03690 Glyma15g30110

Glyma10g35090

**Module 10**


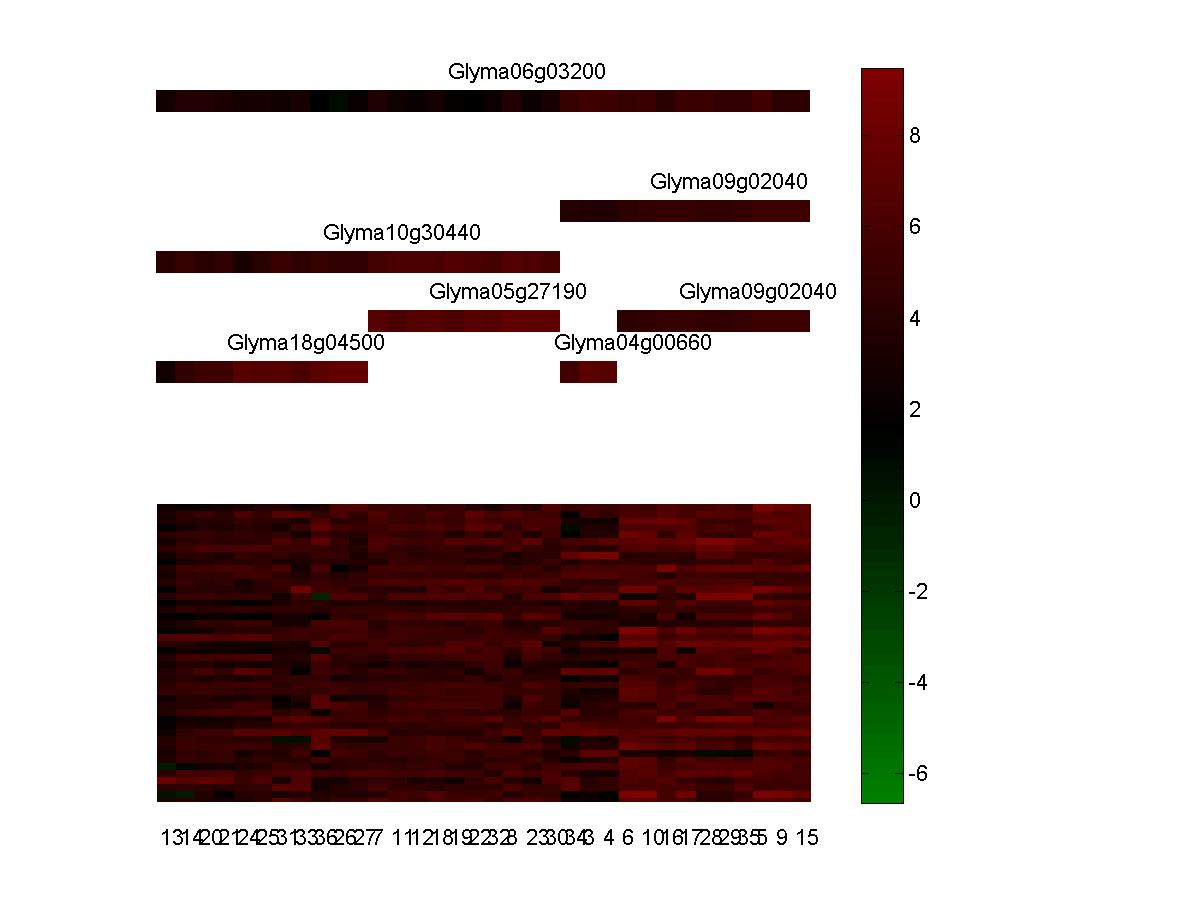


| GO ACC | FUNCTION |
| --- | --- |
| GO:0048765 | P:root hair cell differentiation |
| GO:0010089 | P:xylem development |
| GO:0007109 | P:cytokinesis, completion of separation |
| GO:0010088 | P:phloem development |
| GO:0006071 | P:glycerol metabolic process |
| GO:0009932 | P:cell tip growth |
| GO:0010026 | P:trichome differentiation |
| GO:0008615 | P:pyridoxine biosynthetic process |
| GO:0006564 | P:L-serine biosynthetic process |
| GO:0006537 | P:glutamate biosynthetic process |
| GO:0006796 | P:phosphate metabolic process |
| GO:0007369 | P:gastrulation |
| GO:0006885 | P:regulation of Ph |
| GO:0010102 | P:lateral root morphogenesis |
| GO:0010442 | P:guard cell morphogenesis |
| GO:0052541 | P:plant-type cell wall cellulose metabolic pr… |
| GO:0019762 | P:glucosinolate catabolic process |
| GO:0080028 | P:nitrile biosynthetic process |

Glyma20g30910 Glyma01g02950 Glyma13g01870 Glyma11g05470 Glyma13g19500 Glyma10g44370

Glyma10g35700 Glyma05g37590 Glyma02g04760 Glyma09g04630 Glyma19g16450 Glyma10g30440

Glyma17g03910 Glyma08g18080 Glyma18g50760 Glyma09g02040 Glyma13g24380 Glyma09g36740

Glyma20g26440 Glyma09g12320 Glyma10g44360 Glyma07g32150 Glyma20g29190 Glyma20g00760

Glyma06g05460 Glyma10g30020 Glyma07g05230 Glyma13g19830 Glyma19g03500 Glyma11g25650

Glyma08g29130 Glyma02g00340 Glyma13g00380 Glyma18g04500 Glyma08g00320 Glyma03g30110

Glyma06g36590 Glyma14g02070 Glyma19g30600 Glyma03g40860 Glyma06g03100 Glyma13g05120

Glyma01g26840 Glyma15g09820

**Part B**

**Module F1-F10 are generated based on overlapping genes with pre-selected six families (NIN like, Bzip, GRAS, C2H2 (Zn), HomeoDomain and CCAAT).** The numbers at the bottom of each figure correspond to the experimental conditions:

3)10A_nodule

4)10B_nodule

5)12HA1_IN_RH

6)12HA1_UN_RH

7)1A_flower

8)1B_flower

9)24HA1_IN_RH

10)24HA1_UN_RH

11)2A_cm_pod

12)2B_cm_pod

13)3A_-2_seed

14)3B_-2_seed

15)48HA1_IN_RH

16)48HA1_Scrip_Root

17)48HA1_UN_RH

18)4A_-2_shell

19)4B_-2_shell

20)5A_-1_seed

21)5B_-1_seed

22)6A_-1_shell

23)6B_-1_shell

24)7A_0_seed

25)7B_0_seed

26)8A_young_leaf

27)8B_young_leaf

28)9A_root

29)9B_root

30)Apical_Meristem_Stacey

31)Flower_Stacey

32)Green_Pods_Stacey

33)Leaves_Stacey

34)Nodule_Stacey

35)Root_Stacey

36)Root_Tip_Stacey

The modules are listed one by one in the following order: the figure of the module, the genes of the module.

**Module F1**


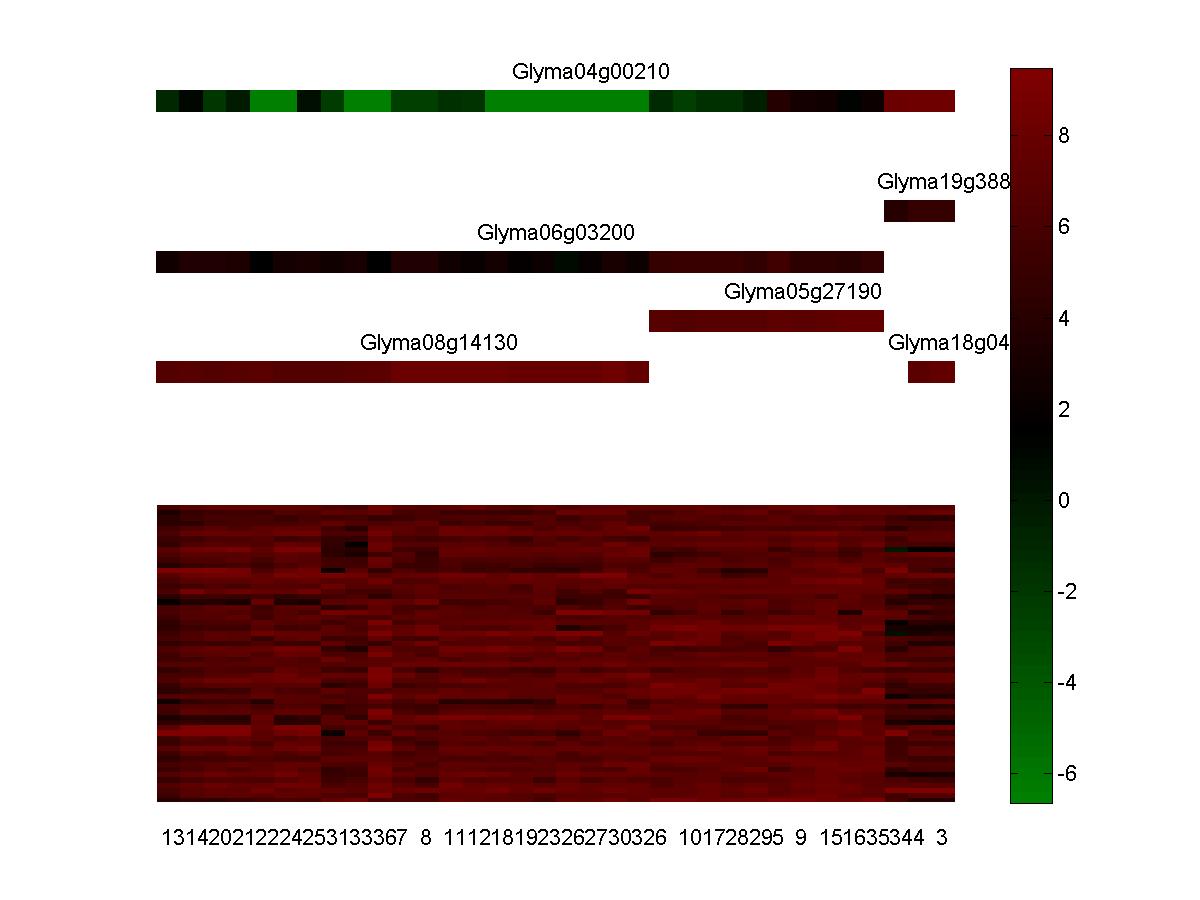


Glyma08g03150 Glyma11g33720 Glyma05g08670 Glyma08g10140 Glyma08g05820 Glyma06g20540

Glyma12g04020 Glyma04g09350 Glyma04g00450 Glyma01g01310 Glyma07g15320 Glyma04g09820

Glyma02g43470 Glyma11g12500 Glyma13g06390 Glyma05g27180 Glyma20g05560 Glyma13g17220

Glyma18g00500 Glyma19g06460 Glyma14g09510 Glyma20g29660 Glyma06g02650 Glyma12g29510

Glyma13g22940 Glyma04g06700 Glyma10g40870 Glyma17g11940 Glyma14g06170 Glyma05g27190

Glyma08g14130 Glyma03g37340 Glyma17g34920 Glyma16g24120 Glyma19g32990 Glyma02g40290

Glyma10g35520 Glyma18g04500 Glyma04g03110 Glyma05g01180 Glyma08g14550 Glyma03g40760

Glyma17g17850 Glyma14g05510 Glyma04g00660 Glyma02g43080 Glyma14g06630 Glyma04g00650

Glyma0169s00210 Glyma05g36420 Glyma05g21820 Glyma10g40150 Glyma04g40430 Glyma07g04890

Glyma06g00990 Glyma05g34570 Glyma10g06600

**Module F2**


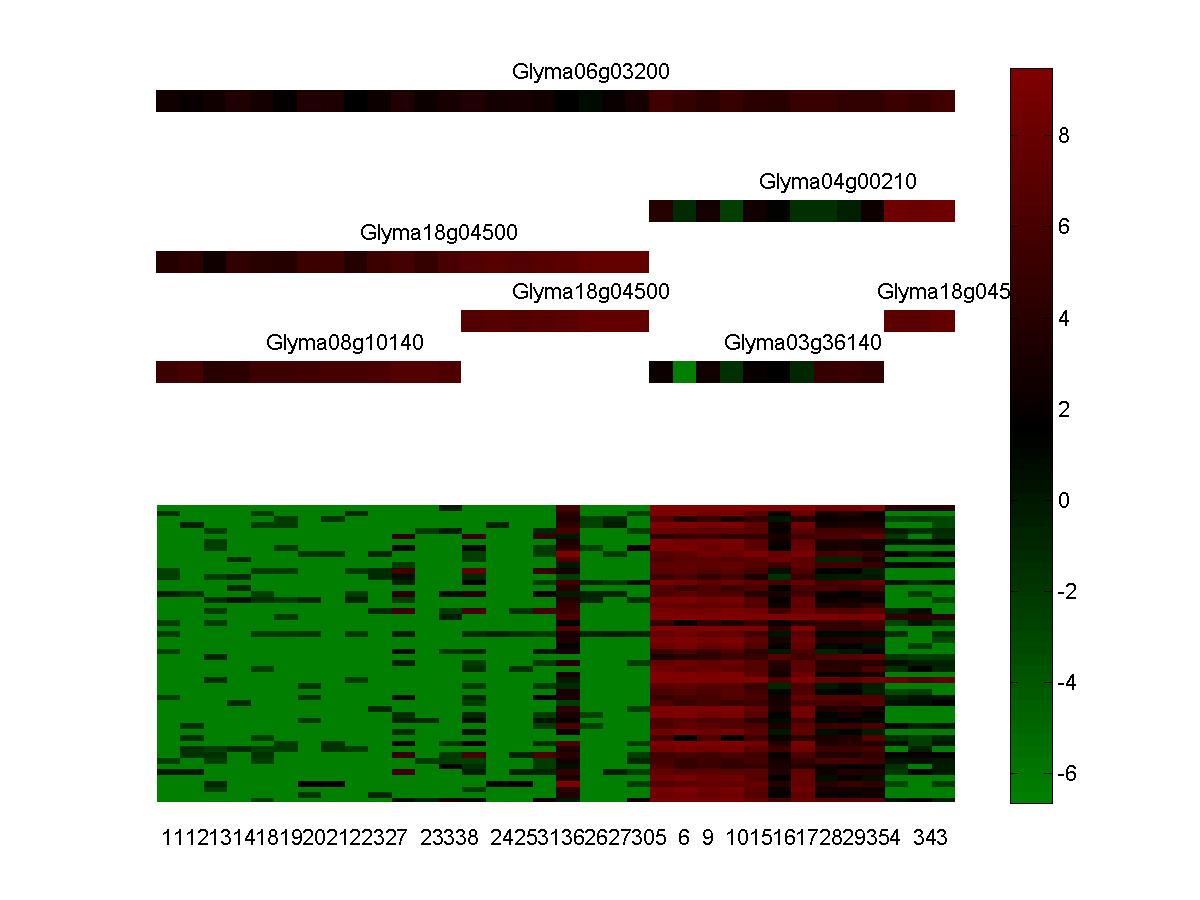


Glyma08g24770 Glyma02g01970 Glyma13g22350 Glyma18g02870 Glyma19g40960 Glyma02g09210

Glyma11g29920 Glyma12g03050 Glyma06g02290 Glyma07g04340 Glyma05g25450 Glyma13g27130

Glyma15g11700 Glyma09g02610 Glyma10g02730 Glyma16g28610 Glyma11g35560 Glyma08g43550

Glyma10g05800 Glyma08g24760 Glyma09g28800 Glyma03g04880 Glyma15g02380 Glyma01g42370

Glyma09g32630 Glyma15g35410 Glyma16g07830 Glyma11g03310 Glyma02g40010 Glyma15g35390

Glyma04g35130 Glyma01g34770 Glyma11g06070 Glyma15g07700 Glyma16g06520 Glyma17g14230

Glyma02g16800 Glyma06g47190 Glyma18g53740 Glyma10g29150 Glyma16g06500 Glyma10g02090

Glyma15g05760 Glyma06g12010 Glyma07g17170 Glyma03g37390 Glyma17g15690 Glyma09g00850

Glyma04g02230 Glyma03g37400 Glyma08g17300 Glyma09g31110

**Module F3**


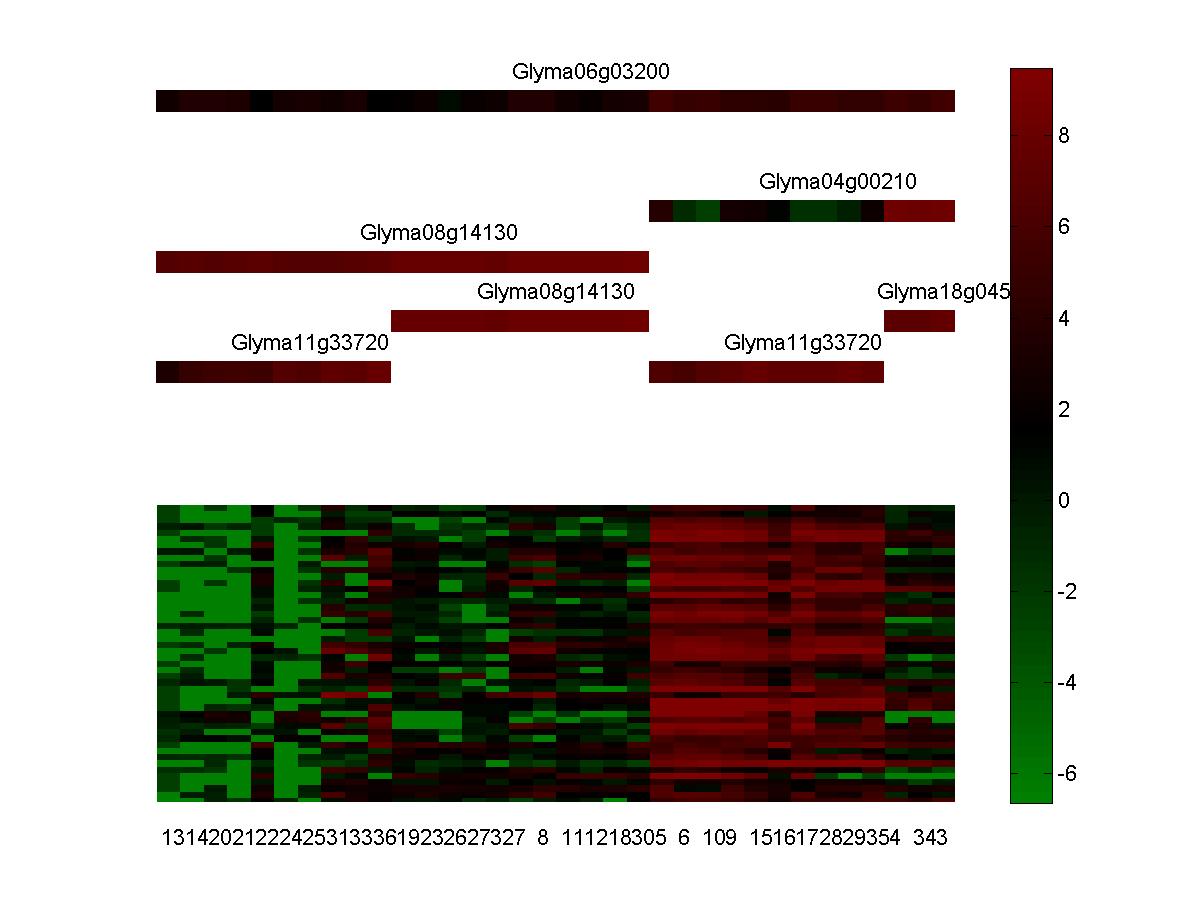


Glyma07g38580 Glyma12g36360 Glyma10g28610 Glyma10g33650 Glyma19g45260 Glyma01g31750

Glyma15g41700 Glyma04g42460 Glyma01g42670 Glyma09g37910 Glyma11g03430 Glyma11g05800

Glyma16g27900 Glyma09g04530 Glyma12g06110 Glyma17g34590 Glyma03g02410 Glyma20g35630

Glyma15g15200 Glyma04g33010 Glyma20g38590 Glyma03g03460 Glyma16g28590 Glyma02g09220

Glyma08g24720 Glyma18g50180 Glyma16g04980 Glyma20g11610 Glyma16g01960 Glyma07g37270

Glyma08g43340 Glyma09g05440 Glyma13g32300 Glyma09g05340 Glyma16g27440 Glyma17g02600

Glyma17g03340 Glyma03g02580 Glyma03g28850 Glyma20g28490 Glyma11g14130 Glyma12g02240

Glyma03g24020 Glyma15g09540 Glyma04g02750 Glyma17g17310 Glyma08g11960 Glyma01g04380

**Module F4**


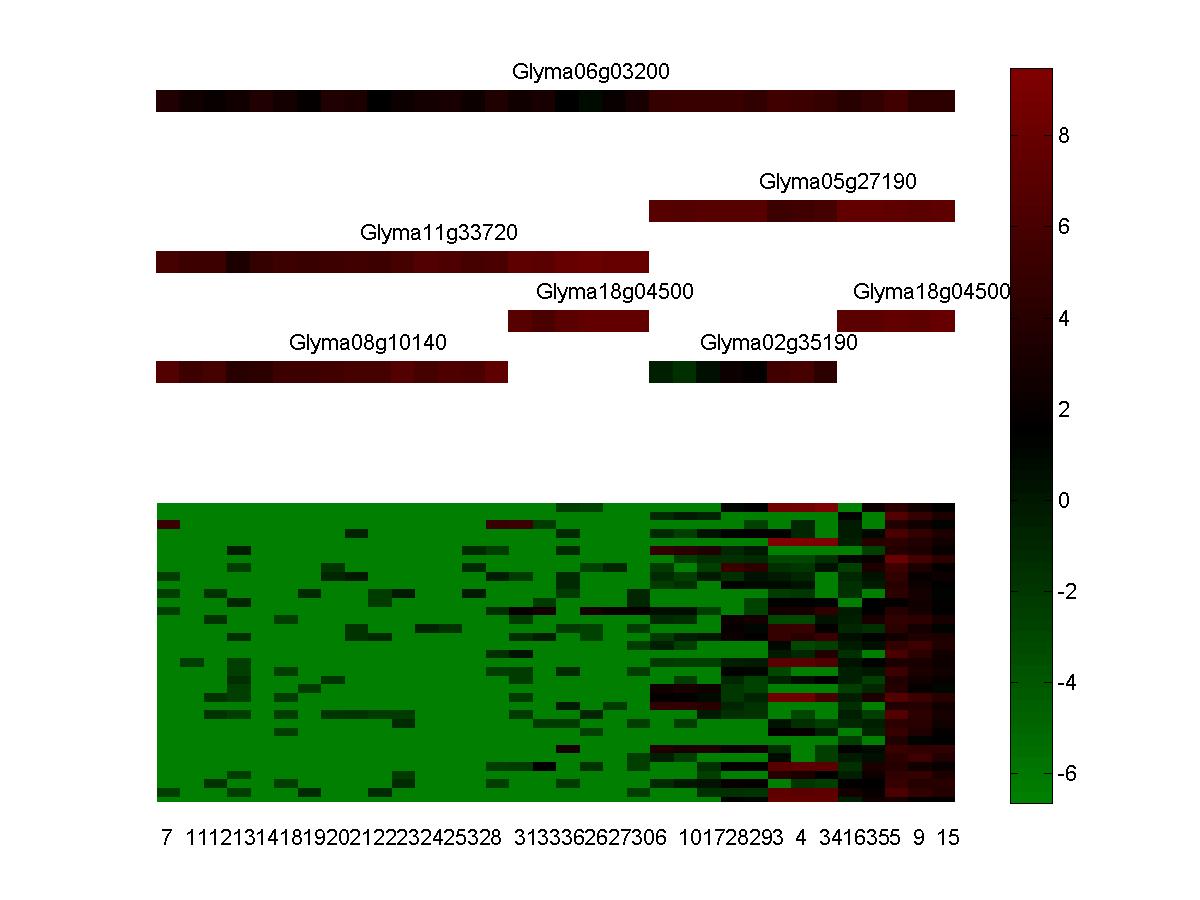


Glyma08g08170 Glyma01g45280 Glyma08g19250 Glyma05g12090 Glyma09g31910 Glyma07g37260

Glyma20g34830 Glyma06g29670 Glyma10g42840 Glyma05g12100 Glyma11g37620 Glyma08g17270

Glyma08g03330 Glyma18g06350 Glyma20g29210 Glyma07g38620 Glyma10g25120 Glyma10g32070

Glyma13g27300 Glyma17g07440 Glyma09g27600 Glyma18g16790 Glyma02g36580 Glyma05g03720

Glyma08g05850 Glyma06g47690 Glyma01g38040 Glyma04g42300 Glyma10g31280 Glyma10g25130

Glyma10g10240 Glyma13g23770 Glyma17g02080 Glyma02g48080 Glyma08g24680

**Module F5**


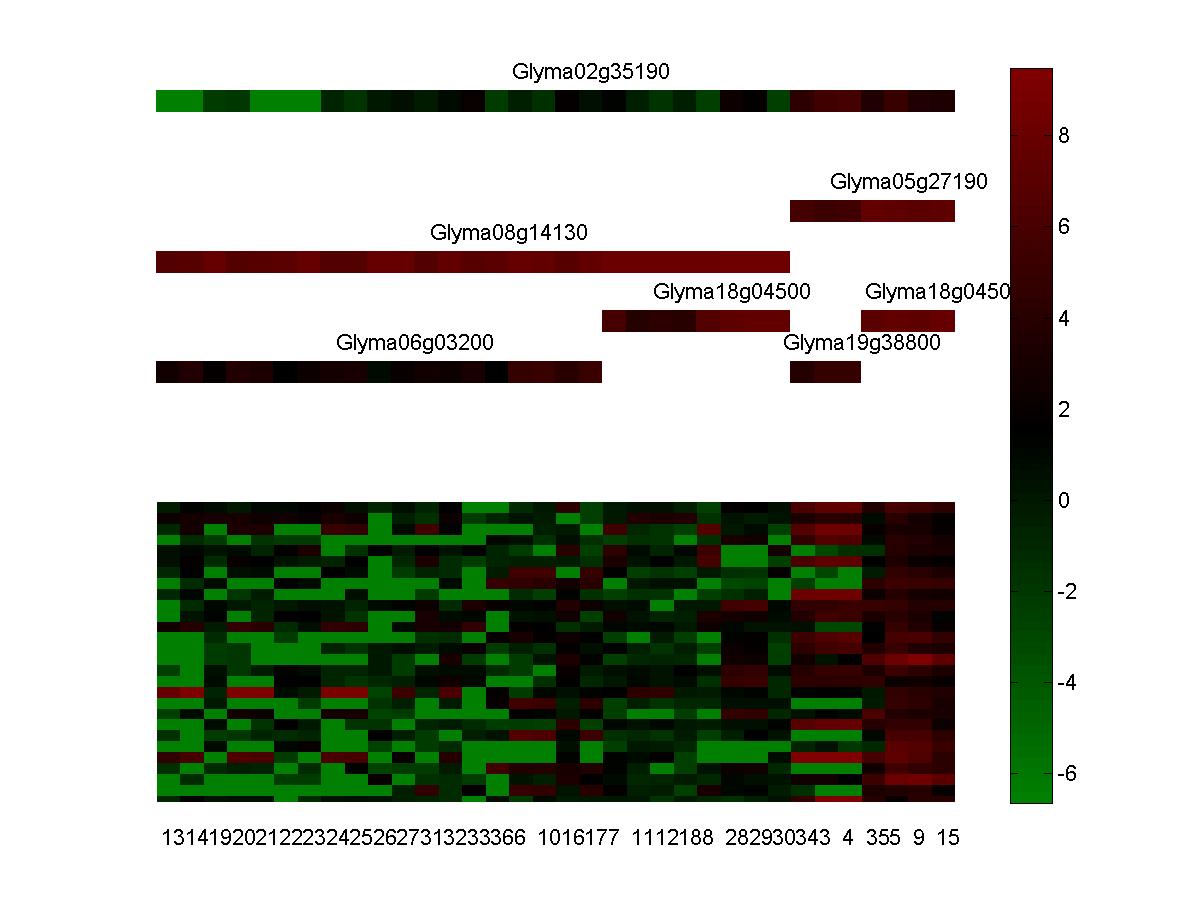


Glyma02g04180 Glyma08g48030 Glyma07g09710 Glyma20g30450 Glyma15g01500 Glyma13g38710

Glyma16g26940 Glyma08g48240 Glyma04g00210 Glyma20g29200 Glyma08g23310 Glyma16g06740

Glyma20g32470 Glyma02g35190 Glyma14g05840 Glyma19g38800 Glyma03g36140 Glyma03g28080

Glyma02g40890 Glyma01g42800 Glyma01g03470 Glyma16g22920 Glyma05g03750 Glyma08g12650

Glyma11g14060 Glyma02g42730 Glyma05g28810 Glyma19g44060

**Module F6**


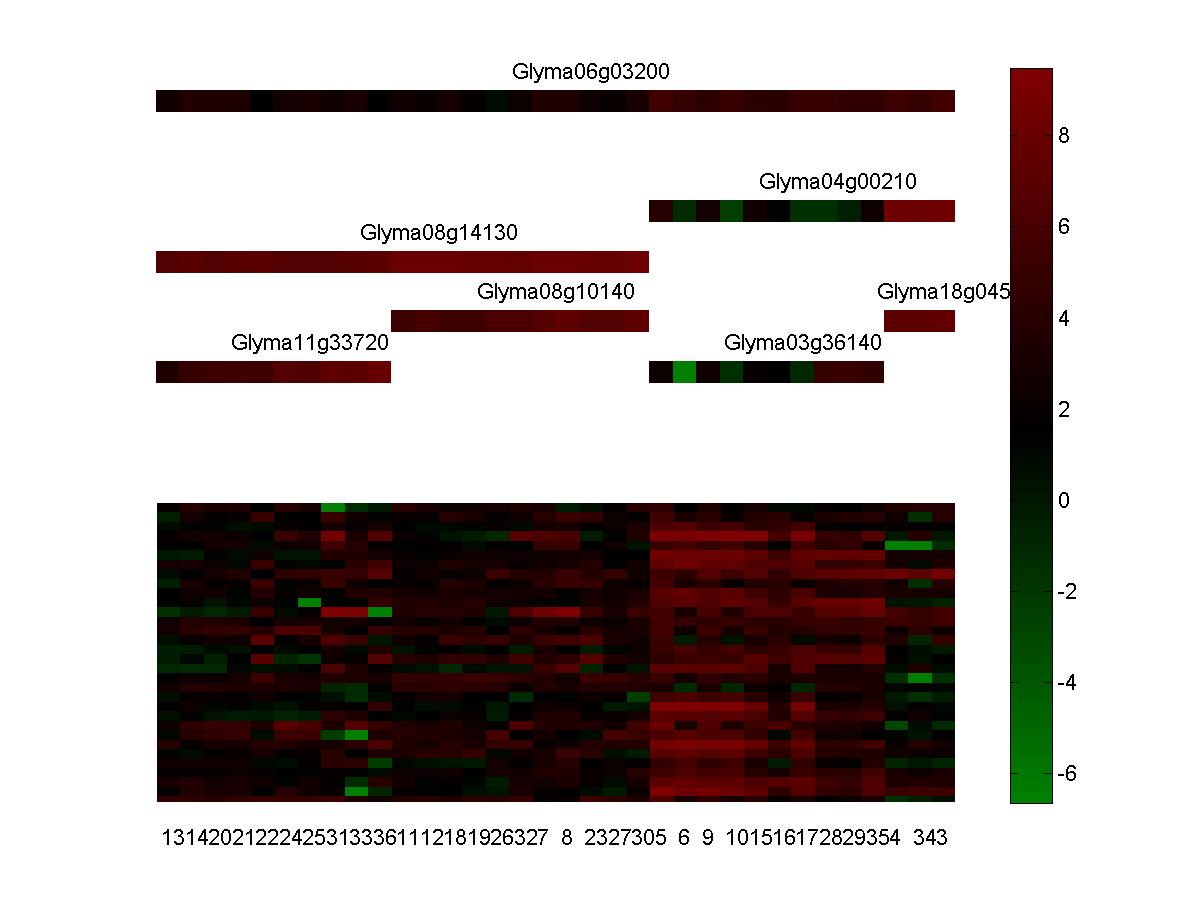


Glyma12g02590 Glyma14g06650 Glyma15g14790 Glyma07g38110 Glyma12g36300 Glyma09g28490

Glyma15g42780 Glyma16g29370 Glyma14g06640 Glyma08g45920 Glyma19g01940 Glyma08g43330

Glyma06g03200 Glyma13g44870 Glyma02g42250 Glyma07g16850 Glyma12g32160 Glyma03g19260

Glyma02g11720 Glyma10g07500 Glyma08g46610 Glyma19g37240 Glyma04g42240 Glyma17g04940

Glyma06g05530 Glyma10g29260 Glyma15g41970 Glyma10g38080 Glyma20g32140 Glyma02g08950

Glyma01g39460 Glyma12g33070

**Module F7**


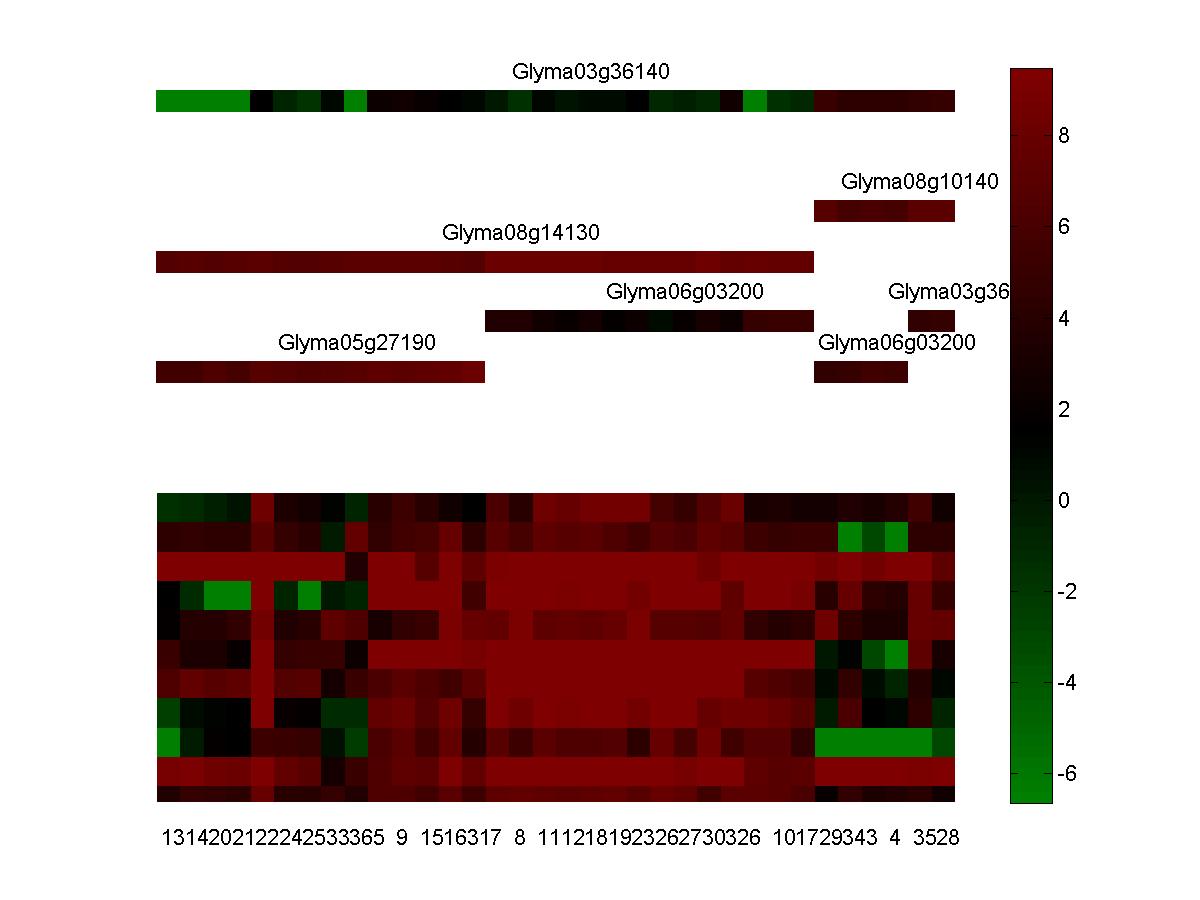


Glyma14g05850 Glyma02g01990 Glyma19g36440 Glyma08g21410 Glyma09g33750 Glyma07g01730

Glyma13g42340 Glyma02g18090 Glyma08g45610 Glyma10g35870 Glyma13g39600

**Module F8**


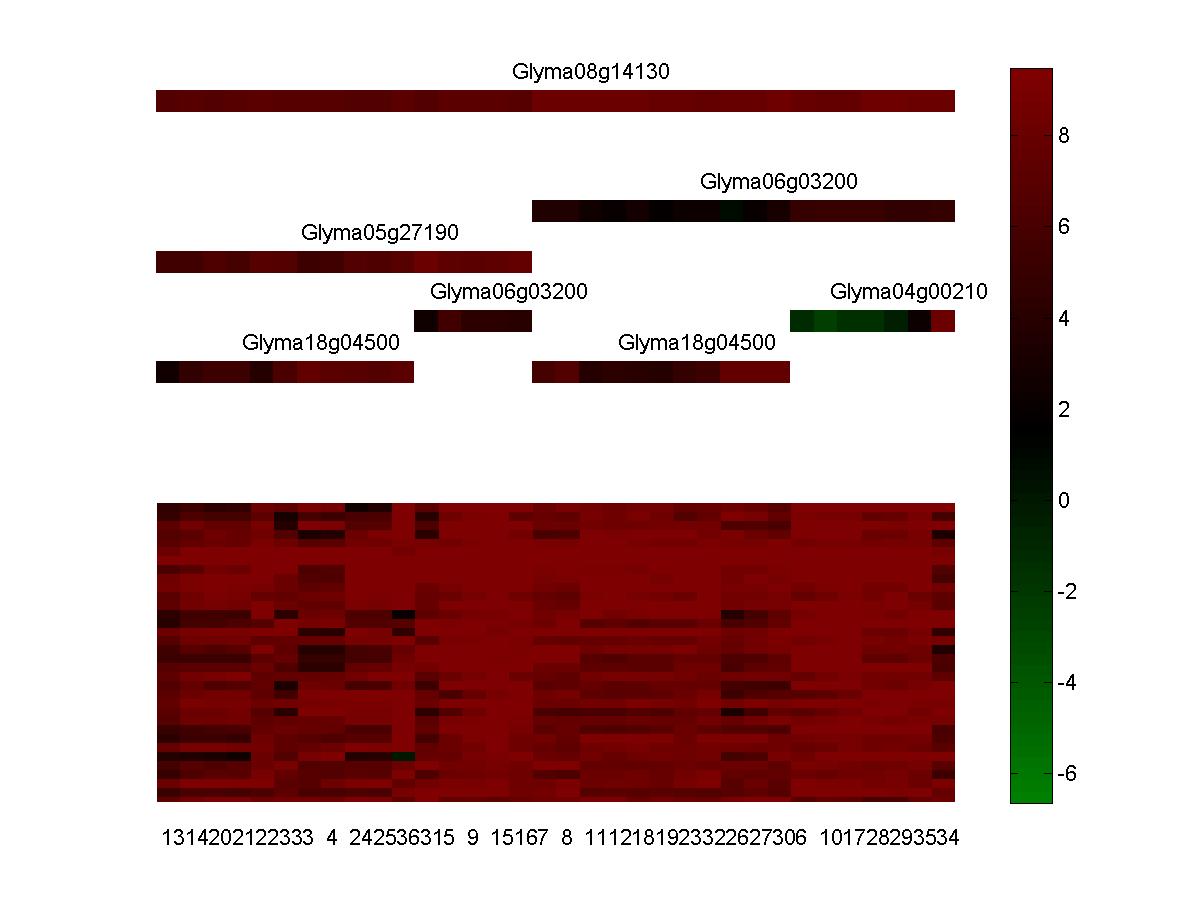


Glyma13g42330 Glyma16g33710 Glyma07g00900 Glyma05g37730 Glyma12g02790 Glyma10g39780

Glyma17g23900 Glyma03g34310 Glyma19g37000 Glyma05g11630 Glyma10g05580 Glyma08g11480

Glyma12g34550 Glyma15g19580 Glyma19g29210 Glyma19g35570 Glyma16g04190 Glyma04g01130

Glyma13g40100 Glyma07g34440 Glyma07g00910 Glyma09g01320 Glyma03g32850 Glyma15g12170

Glyma19g35560 Glyma02g04510 Glyma08g46520 Glyma05g24110 Glyma12g34570 Glyma14g09440

Glyma08g11490 Glyma17g23870 Glyma12g00390 Glyma11g12510

**Module F9**


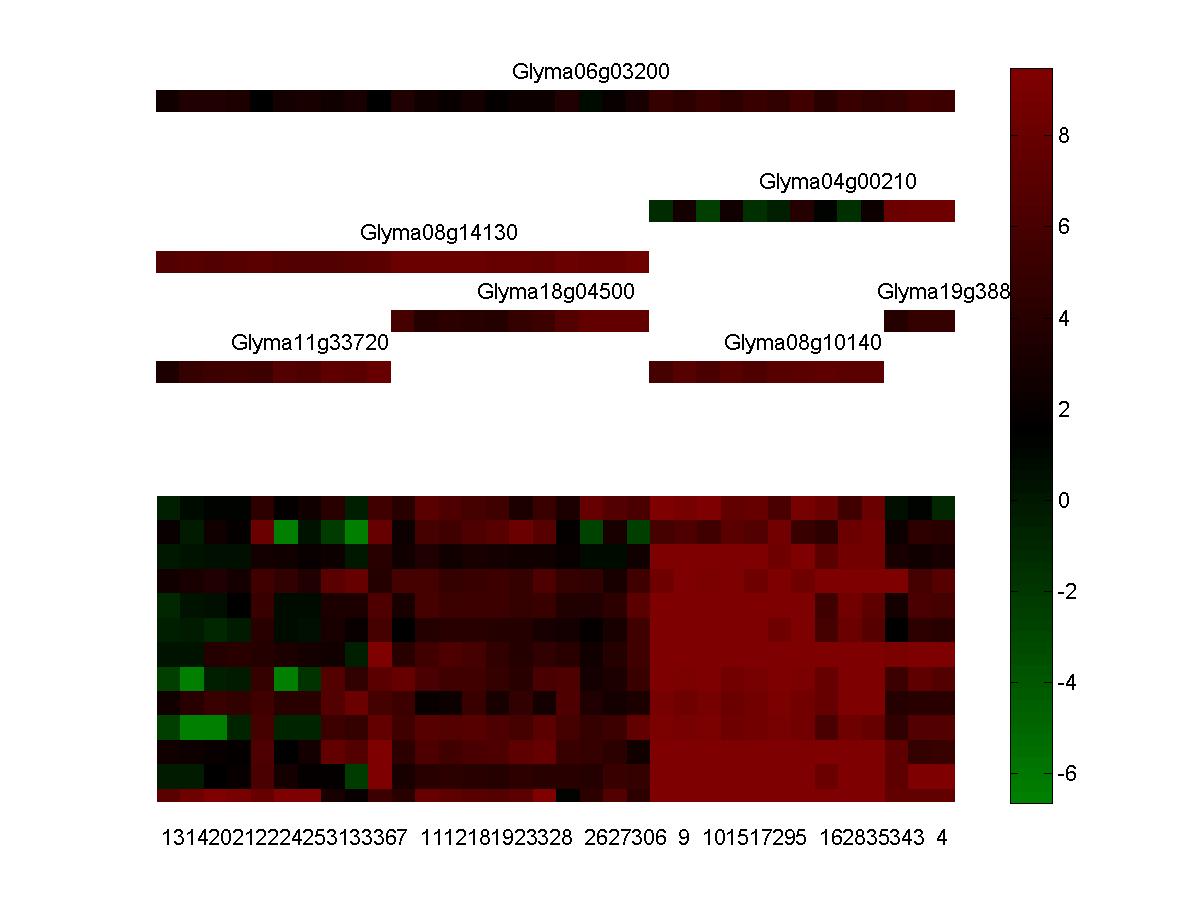


Glyma09g02600 Glyma05g04440 Glyma17g03350 Glyma17g34870 Glyma12g06100 Glyma11g14140

Glyma10g35080 Glyma16g28600 Glyma11g00230 Glyma02g09200 Glyma11g03690 Glyma10g35090

Glyma09g12200

**Module F10**


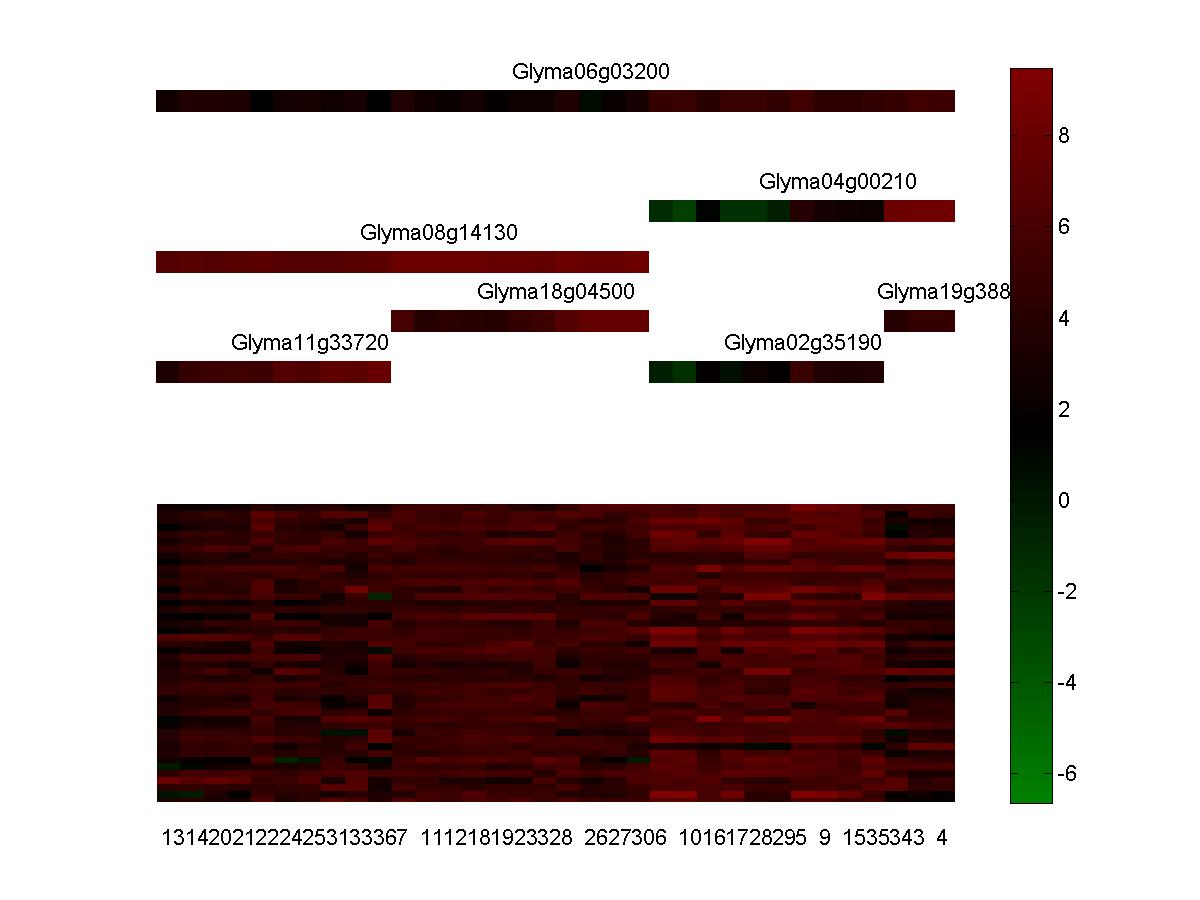


Glyma20g30910 Glyma01g02950 Glyma13g01870 Glyma11g05470 Glyma13g19500 Glyma10g44370

Glyma10g35700 Glyma05g37590 Glyma02g04760 Glyma09g04630 Glyma19g16450 Glyma10g30440

Glyma17g03910 Glyma08g18080 Glyma18g50760 Glyma09g02040 Glyma13g24380 Glyma09g36740

Glyma20g26440 Glyma09g12320 Glyma10g44360 Glyma07g32150 Glyma20g29190 Glyma20g00760

Glyma06g05460 Glyma10g30020 Glyma07g05230 Glyma13g19830 Glyma19g03500 Glyma11g25650

Glyma08g29130 Glyma02g00340 Glyma13g00380 Glyma08g00320 Glyma03g30110 Glyma06g36590

Glyma14g02070 Glyma15g30110 Glyma19g30600 Glyma03g40860 Glyma06g03100 Glyma13g05120

Glyma01g26840 Glyma15g09820

Genes with sequence AAAGAT

Module1

Glyma01g01310

Glyma02g40290

Glyma03g37340

Glyma04g00450

Glyma04g00660

Glyma04g09820

Glyma04g40430

Glyma05g21820

Glyma05g36420

Glyma08g03150

Glyma10g35520

Glyma10g40150

Glyma12g29510

Glyma13g06390

Glyma14g06630

Glyma14g09510

Glyma16g24120

Glyma17g11940

Glyma17g17850

Glyma19g32990

Module2

Glyma01g42370

Glyma04g02230

Glyma09g32630

Glyma10g02090

Glyma10g31280

Glyma11g03310

Glyma13g22350

Glyma13g27130

Glyma15g35390

Glyma15g35410

Glyma17g15690

Module3

Glyma03g24020

Glyma03g28850

Glyma07g37270

Glyma08g24720

Glyma10g28610

Glyma12g02240

Glyma16g28590

Glyma19g45260

Glyma20g38590

Module4

Glyma01g38040

Glyma08g08170

Glyma08g17270

Glyma08g24680

Glyma13g23770

Glyma17g02080

Glyma17g07440

Glyma20g34830

Module5

Glyma01g03470

Glyma01g42800

Glyma03g28080

Glyma05g28810

Glyma08g12650

Glyma08g48240

Glyma19g38800

Module6

Glyma03g19260

Glyma04g33010

Glyma06g03200

Glyma06g05530

Glyma07g38110

Glyma08g46610

Glyma09g28490

Glyma10g07500

Glyma13g44870

Glyma14g06650

Module7

Glyma08g45610

Module8

Glyma05g24110

Glyma07g00900

Glyma09g01320

Glyma12g34550

Glyma13g40100

Glyma15g12170

Glyma16g04190

Module9

Glyma12g06100

Glyma15g30110

Glyma17g34870

Module10

Glyma01g02950

Glyma01g26840

Glyma02g00340

Glyma03g40860

Glyma06g03100

Glyma07g05230

Glyma08g00320

Glyma08g18080

Glyma09g02040

Glyma09g04630

Glyma09g12320

Glyma10g30440

Glyma13g05120

Glyma13g19500

Glyma13g19830

Glyma20g29190

Glyma20g30910

Genes with sequence CTCTT

Module1

Glyma01g01310

Glyma02g40290

Glyma03g37340

Glyma03g40760

Glyma04g00660

Glyma04g03110

Glyma04g06700

Glyma04g09350

Glyma05g01180

Glyma05g27190

Glyma05g34570

Glyma07g15320

Glyma08g03150

Glyma08g14130

Glyma10g06600

Glyma10g40150

Glyma11g33720

Glyma12g04020

Glyma12g29510

Glyma13g22940

Glyma19g06460

Glyma20g29660

Glyma0169s00210

Module2

Glyma02g16800

Glyma02g40010

Glyma03g37390

Glyma03g37400

Glyma06g02290

Glyma06g47190

Glyma07g04340

Glyma07g17170

Glyma08g43550

Glyma09g00850

Glyma09g32630

Glyma10g05800

Glyma10g31280

Glyma11g03310

Glyma11g29920

Glyma11g35560

Glyma12g03050

Glyma13g22350

Glyma15g02380

Glyma15g07700

Glyma15g11700

Glyma17g15690

Glyma18g02870

Glyma19g40960

Module3

Glyma01g04380

Glyma01g42670

Glyma03g24020

Glyma06g12010

Glyma07g37270

Glyma07g38580

Glyma09g04530

Glyma09g37910

Glyma16g01960

Glyma16g04980

Glyma16g27900

Glyma18g50180

Glyma20g35630

Module4

Glyma01g38040

Glyma02g36580

Glyma04g42300

Glyma06g47690

Glyma07g38620

Glyma08g08170

Glyma10g10240

Glyma10g32070

Glyma11g37620

Glyma13g23770

Glyma13g27300

Glyma16g06500

Glyma17g07440

Glyma18g06350

Glyma20g29210

Module5

Glyma02g35190

Glyma02g40890

Glyma03g02580

Glyma03g28080

Glyma03g36140

Glyma04g00210

Glyma07g09710

Glyma08g12650

Glyma08g48030

Glyma09g05340

Glyma13g38710

Glyma14g05840

Glyma15g01500

Glyma16g06740

Glyma16g22920

Glyma16g26940

Glyma19g44060

Glyma20g29200

Glyma20g30450

Module6

Glyma01g39460

Glyma02g08950

Glyma02g11720

Glyma02g42250

Glyma06g03200

Glyma06g05530

Glyma07g16850

Glyma08g11960

Glyma08g43330

Glyma08g46610

Glyma09g28490

Glyma10g07500

Glyma12g02590

Glyma14g06640

Glyma14g06650

Glyma15g41970

Glyma16g29370

Glyma19g01940

Glyma19g37240

Glyma20g32140

Module7

Glyma02g01990

Glyma08g45610

Glyma13g39600

Glyma13g42340

Glyma14g05850

Module8

Glyma04g01130

Glyma05g37730

Glyma09g01320

Glyma10g35870

Glyma12g00390

Glyma13g40100

Glyma15g12170

Glyma15g19580

Glyma16g33710

Glyma17g23870

Glyma19g35560

Module9

Glyma11g03690

Glyma11g14140

Module10

Glyma02g04760

Glyma06g03100

Glyma06g05460

Glyma06g36590

Glyma08g00320

Glyma08g18080

Glyma08g29130

Glyma09g02040

Glyma09g04630

Glyma09g12320

Glyma10g30020

Glyma11g25650

Glyma13g00380

Glyma13g05120

Glyma17g03910

Glyma18g04500

Glyma18g50760

Glyma19g30600

Glyma20g00760

Glyma20g29190

Glyma20g30910
