# Supplementary material for: Predicting gene regulatory networks of soybean nodulation from RNA-Seq transcriptome data
Source: BMC Bioinformatics. 2013 Sep 22;14:278. doi: 10.1186/1471-2105-14-278 (PMC3854569; doi:10.1186/1471-2105-14-278)

Supplementary Figure 1 - Module 5 generated based on the overlapped DEGs


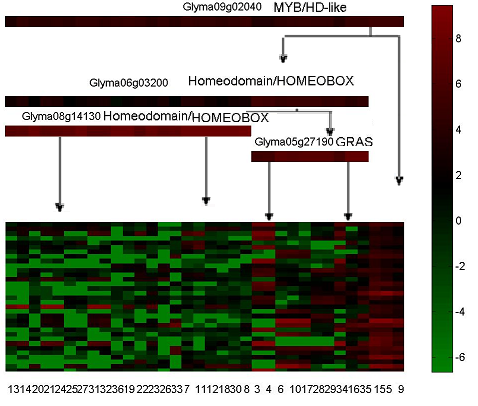


Supplementary Figure 2 - Module stability after incorporating different percent of non-differential expressed TFs.


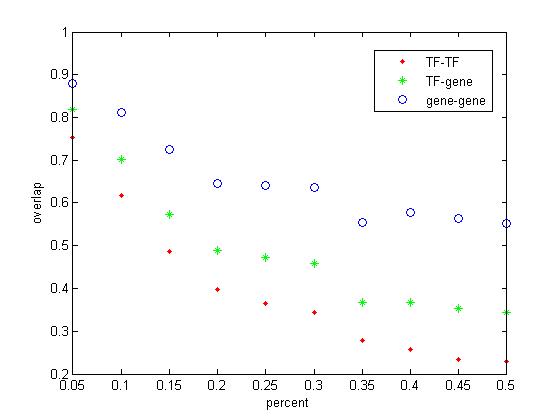

Supplement: Additional file 2 Figure S1 — Module 5 generated based on the overlapped DEGs; Figure S2. Module stability after incorporating different percent of non-differential expressed TFs. [file 1471-2105-14-278-S2.docx]
