# Supplementary material for: Predicting gene regulatory networks of soybean nodulation from RNA-Seq transcriptome data
Source: BMC Bioinformatics. 2013 Sep 22;14:278. doi: 10.1186/1471-2105-14-278 (PMC3854569; doi:10.1186/1471-2105-14-278)
Supplement: Additional file 3 — Modules generated based on the 12-hour DEGs. [file 1471-2105-14-278-S3.doc]

**Supplemental Materials**

**The module 5,9,12,18 and 19 are predicted with GRAS family TFs. Among of them moduel 19 is with Leucine Zipper domain, module 12 is with** bHLH-Zip domain **based on binding site analysis.**

**Module 1-24 are generated based on 12 hour DEGs with all included TF families.**

1


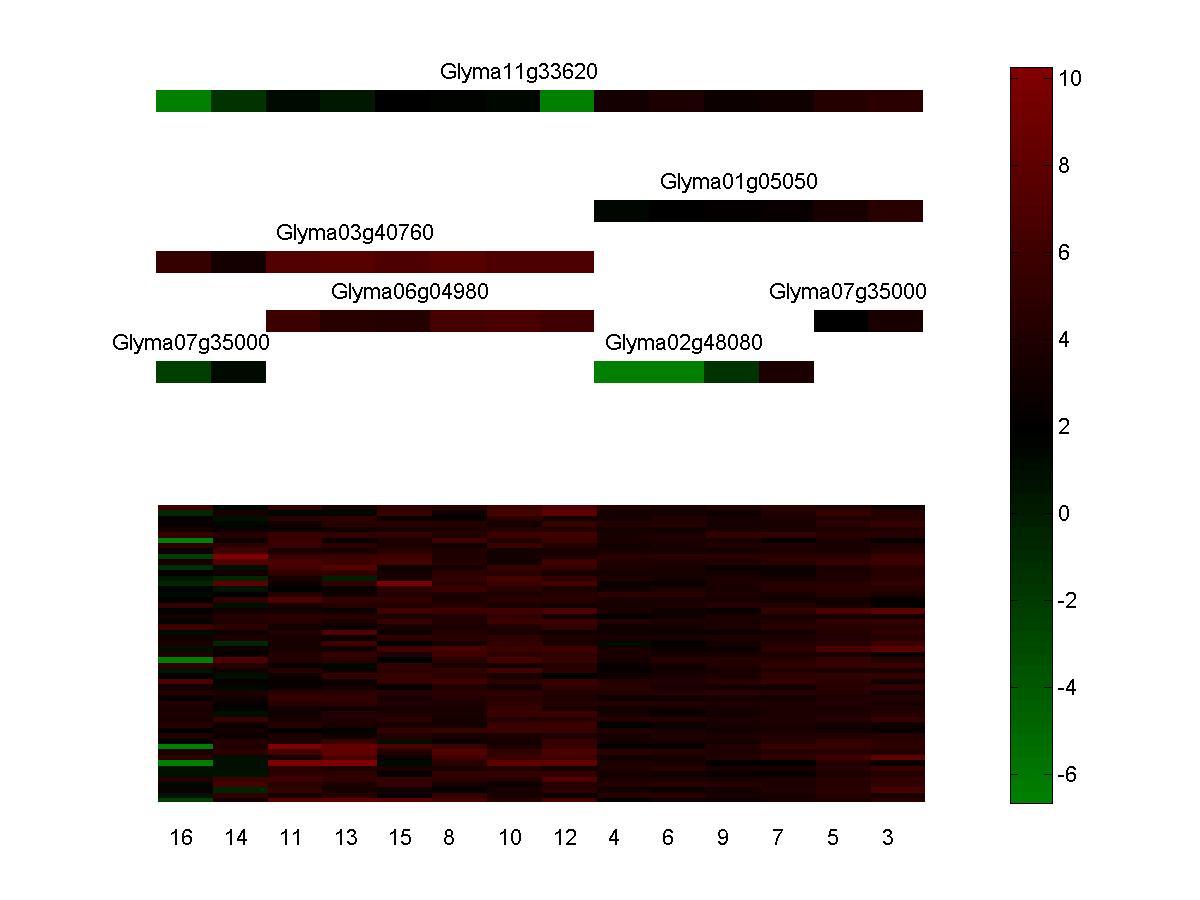


1 Glyma11g33620 MYB/HD-like

1 Glyma01g05050 WRKY

1 Glyma06g04980 LIM

1 Glyma07g35000 AS2

1 Glyma02g48080 Nin-like

1 Glyma03g40760 AUX-IAA-ARF

Glyma19g44540 Glyma14g05850 Glyma13g40030 Glyma05g08950 Glyma09g37920

Glyma08g18310 Glyma11g21640 Glyma07g36060 Glyma20g33120 Glyma05g01360

Glyma04g40000 Glyma07g39720 Glyma17g08950 Glyma10g36680 Glyma08g18080

Glyma06g14640 Glyma08g47020 Glyma15g10490 Glyma13g31620 Glyma13g24380

Glyma13g29430 Glyma02g46330 Glyma09g36740 Glyma18g47930 Glyma15g01510

Glyma13g17570 Glyma07g32150 Glyma18g14640 Glyma18g42580 Glyma13g44870

Glyma15g00390 Glyma18g50180 Glyma11g25650 Glyma04g03260 Glyma08g15430

Glyma10g30220 Glyma11g13670 Glyma11g18290 Glyma13g24340 Glyma03g32940

Glyma08g01720 Glyma05g00740 Glyma06g13850 Glyma06g36590 Glyma08g43340

Glyma05g36710 Glyma17g04940 Glyma03g08280 Glyma08g22690 Glyma19g42020

Glyma06g14850 Glyma17g15040 Glyma12g33070 Glyma19g41020 Glyma18g10200

2


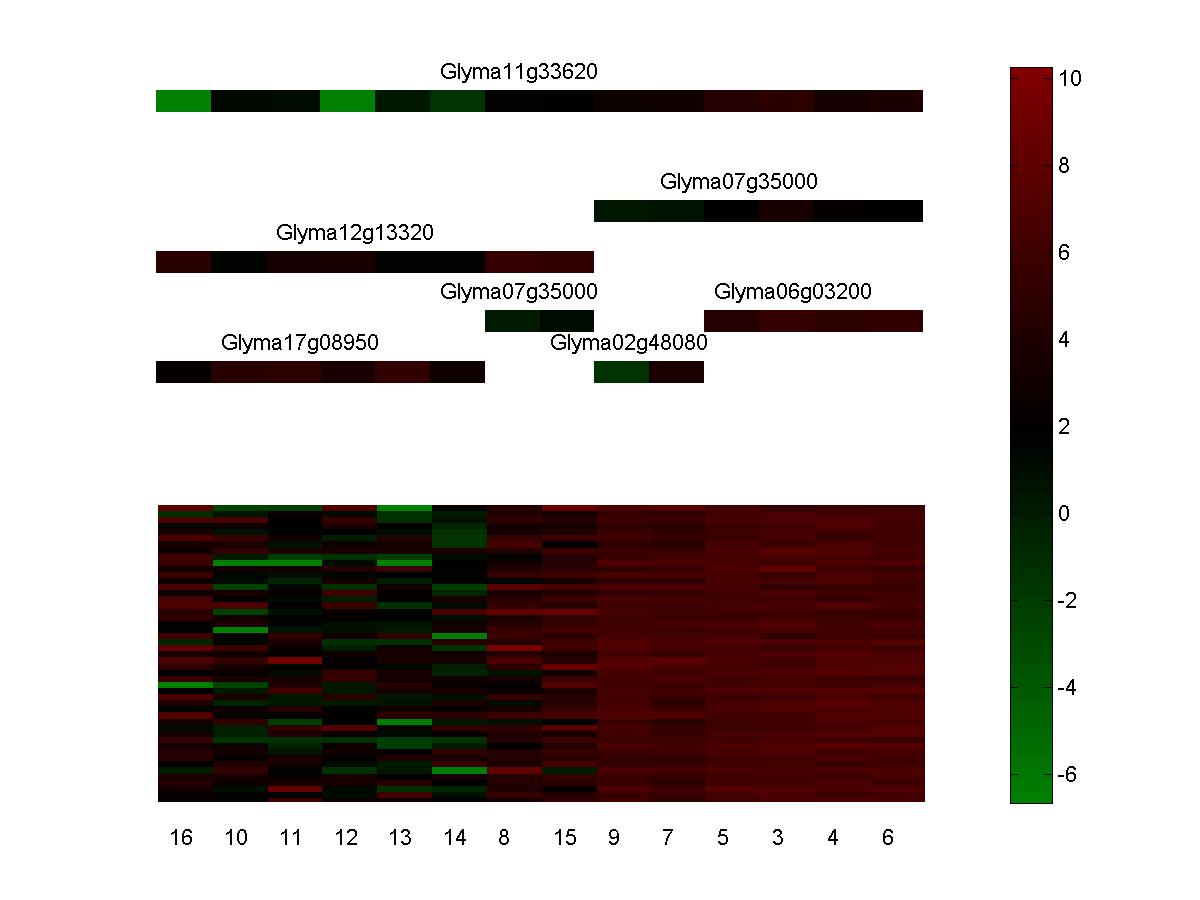


2 Glyma12g13320 AP2-EREBP

2 Glyma06g03200 Homeodomain/HOMEOBOX

2 Glyma11g33620 MYB/HD-like

2 Glyma17g08950 C2C2 (Zn) Dof

2 Glyma07g35000 AS2

2 Glyma02g48080 Nin-like

Glyma05g04440 Glyma10g28610 Glyma08g04400 Glyma05g08210 Glyma09g40090

Glyma04g42460 Glyma07g09700 Glyma15g03460 Glyma08g20230 Glyma09g37910

Glyma17g12150 Glyma02g07960 Glyma08g18880 Glyma17g14920 Glyma06g23590

Glyma20g28350 Glyma08g04390 Glyma09g04530 Glyma14g17730 Glyma04g40170

Glyma01g36590 Glyma08g01740 Glyma03g02410 Glyma17g01220 Glyma08g45920

Glyma09g04340 Glyma19g01940 Glyma15g38060 Glyma20g00760 Glyma19g25980

Glyma03g19260 Glyma08g00320 Glyma16g01960 Glyma04g42240 Glyma16g27440

Glyma06g05530 Glyma15g30110 Glyma15g41970 Glyma15g06000 Glyma11g14130

Glyma02g15150 Glyma07g07420 Glyma19g29590 Glyma03g03270 Glyma08g27560

Glyma08g16300 Glyma12g36330 Glyma13g30770 Glyma08g11960

3


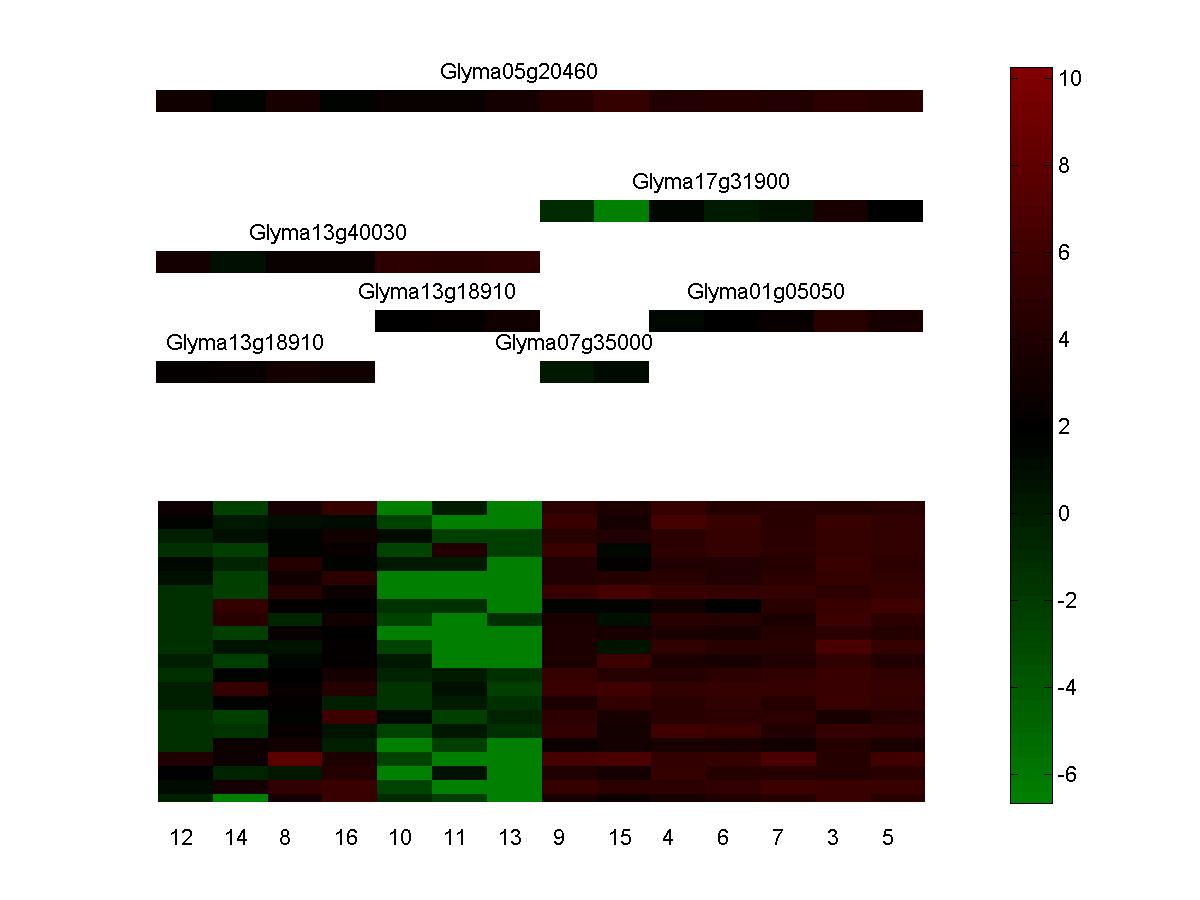


3 Glyma13g18910 AUX-IAA-ARF

3 Glyma05g20460 HSF

3 Glyma13g40030 AUX-IAA-ARF

3 Glyma01g05050 WRKY

3 Glyma17g31900 AP2-EREBP

3 Glyma07g35000 AS2

Glyma20g21440 Glyma08g05490 Glyma20g31040 Glyma17g13530 Glyma19g28770

Glyma08g48240 Glyma13g26070 Glyma20g32470 Glyma17g13500 Glyma03g05480

Glyma17g11170 Glyma19g37620 Glyma05g33470 Glyma06g08630 Glyma03g03870

Glyma15g39090 Glyma08g46610 Glyma15g35070 Glyma18g50500 Glyma10g15280

Glyma03g02580 Glyma11g14060

4


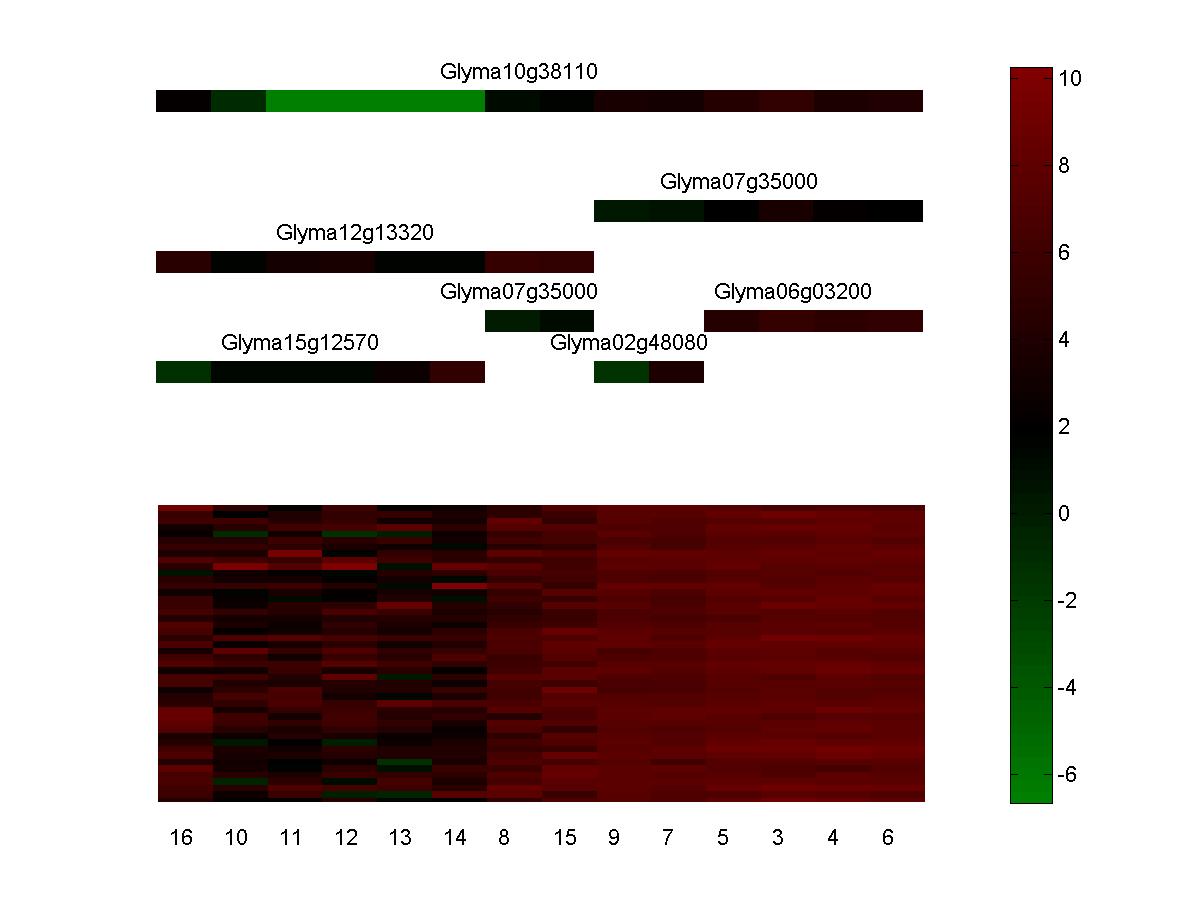


4 Glyma06g03200 Homeodomain/HOMEOBOX

4 Glyma10g38110 MYB/HD-like

4 Glyma15g12570 CCAAT

4 Glyma12g13320 AP2-EREBP

4 Glyma07g35000 AS2

4 Glyma02g48080 Nin-like

Glyma18g49240 Glyma13g22650 Glyma13g01870 Glyma04g40810 Glyma10g33650

Glyma11g09190 Glyma13g19500 Glyma09g07100 Glyma09g32080 Glyma15g03040

Glyma07g05480 Glyma03g38630 Glyma20g28320 Glyma09g28490 Glyma15g42780

Glyma17g03910 Glyma09g32090 Glyma18g50760 Glyma01g07930 Glyma09g06250

Glyma10g40870 Glyma10g44360 Glyma13g44950 Glyma06g07290 Glyma07g09730

Glyma16g28590 Glyma10g37080 Glyma04g12480 Glyma08g06470 Glyma20g38140

Glyma16g20780 Glyma12g33530 Glyma02g11640 Glyma19g40810 Glyma03g30110

Glyma19g00550 Glyma17g03340 Glyma10g29260 Glyma11g01350 Glyma02g08950

Glyma11g37360 Glyma07g16910 Glyma13g24200 Glyma12g05150 Glyma18g06840

Glyma13g01230

5


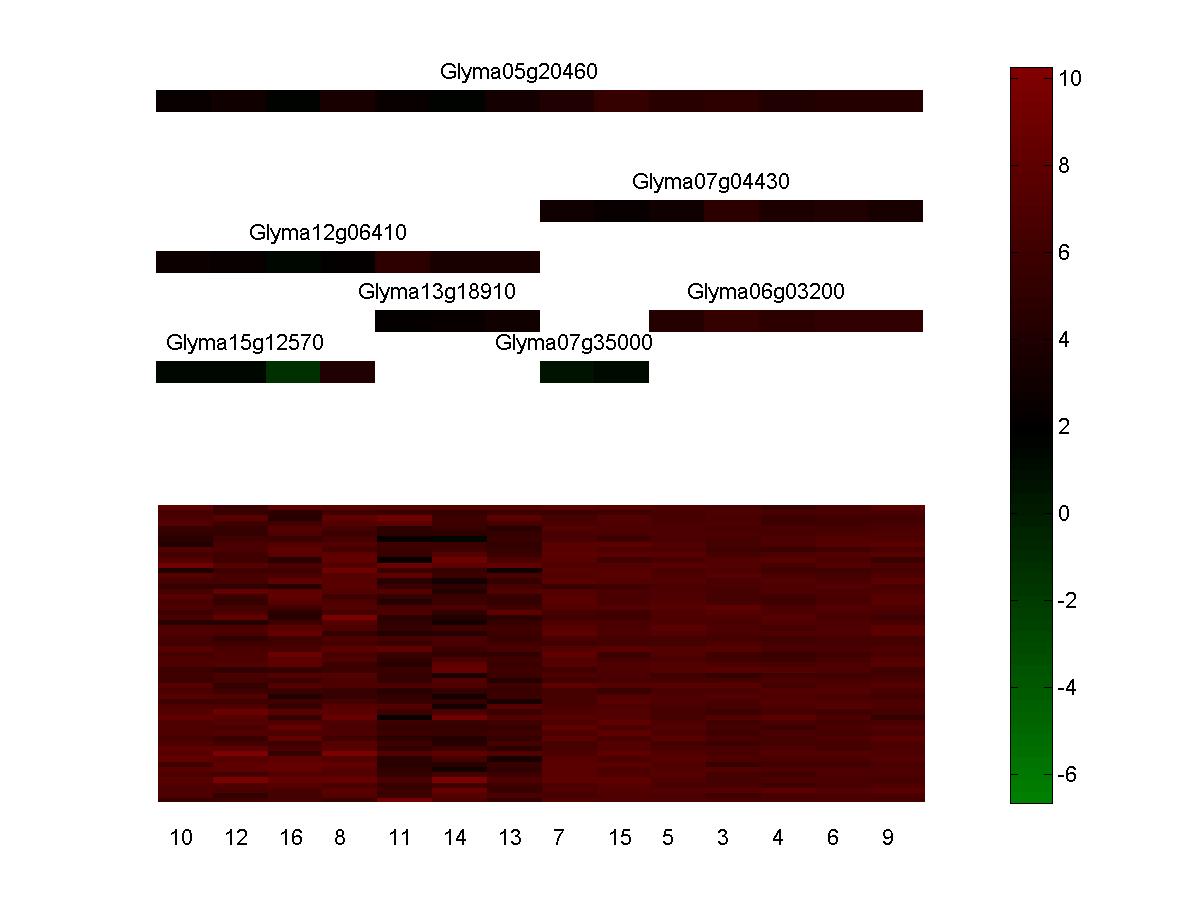


5 Glyma12g06410 MYB/HD-like

5 Glyma15g12570 CCAAT

5 Glyma05g20460 HSF

5 Glyma13g18910 AUX-IAA-ARF

5 Glyma07g04430 GRAS

5 Glyma06g03200 Homeodomain/HOMEOBOX

5 Glyma07g35000 AS2

Glyma11g33720 Glyma05g08670 Glyma06g00710 Glyma08g10140 Glyma12g04020

Glyma08g07150 Glyma16g28080 Glyma10g44370 Glyma07g15320 Glyma04g09820

Glyma02g43470 Glyma11g12500 Glyma09g04630 Glyma05g27180 Glyma20g32000

Glyma13g17220 Glyma18g00500 Glyma20g28780 Glyma19g06460 Glyma20g29660

Glyma11g29350 Glyma05g30380 Glyma01g44600 Glyma01g03180 Glyma07g00330

Glyma08g06010 Glyma05g37300 Glyma05g27190 Glyma03g37340 Glyma06g34190

Glyma08g44590 Glyma08g47500 Glyma11g19490 Glyma19g36080 Glyma18g04500

Glyma04g03110 Glyma13g41930 Glyma08g47790 Glyma03g40760 Glyma17g17850

Glyma14g05510 Glyma04g00660 Glyma02g43080 Glyma04g00650 Glyma02g42260

Glyma14g07150 Glyma20g38440 Glyma07g39020 Glyma09g36560 Glyma05g21820

Glyma10g40150 Glyma04g40430 Glyma14g10590 Glyma20g10820 Glyma08g11040

Glyma05g30950 Glyma08g06420

6


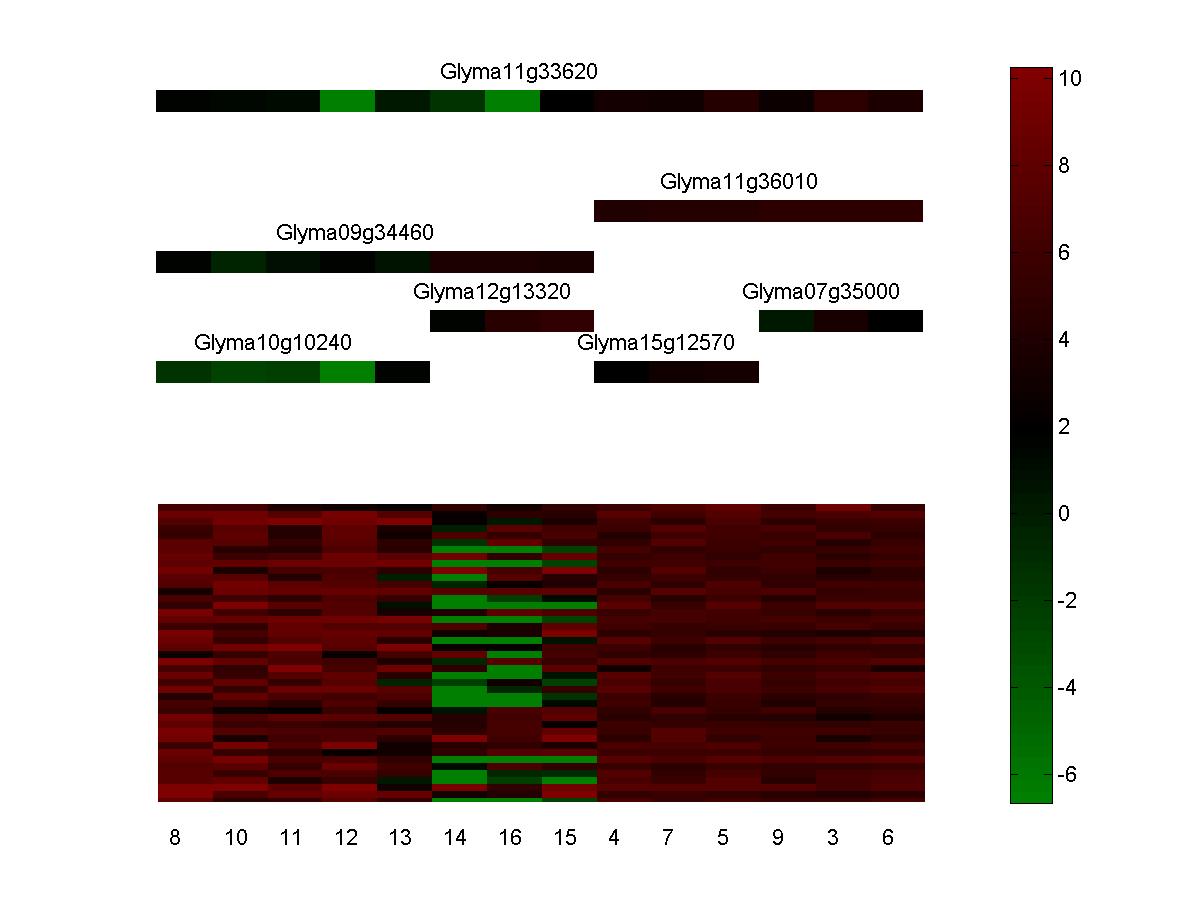


6 Glyma12g13320 AP2-EREBP

6 Glyma15g12570 CCAAT

6 Glyma10g10240 CCAAT

6 Glyma11g36010 BZIP

6 Glyma07g35000 AS2

6 Glyma09g34460 MYB/HD-like

6 Glyma11g33620 MYB/HD-like

Glyma20g30910 Glyma08g15960 Glyma05g00620 Glyma04g00450 Glyma01g01310

Glyma05g04290 Glyma18g10690 Glyma02g13490 Glyma15g38090 Glyma08g36680

Glyma02g01990 Glyma13g34290 Glyma14g09510 Glyma08g43160 Glyma03g32830

Glyma17g11940 Glyma15g38080 Glyma13g20560 Glyma01g02220 Glyma07g36810

Glyma07g35310 Glyma01g38650 Glyma09g37290 Glyma08g43330 Glyma07g36800

Glyma02g45690 Glyma02g09780 Glyma09g24900 Glyma08g43150 Glyma12g32160

Glyma09g33750 Glyma03g31530 Glyma02g00340 Glyma08g36690 Glyma13g42340

Glyma03g28850 Glyma14g20450 Glyma06g46350 Glyma20g34900 Glyma08g45610

Glyma10g35870 Glyma01g02230 Glyma18g10680

7


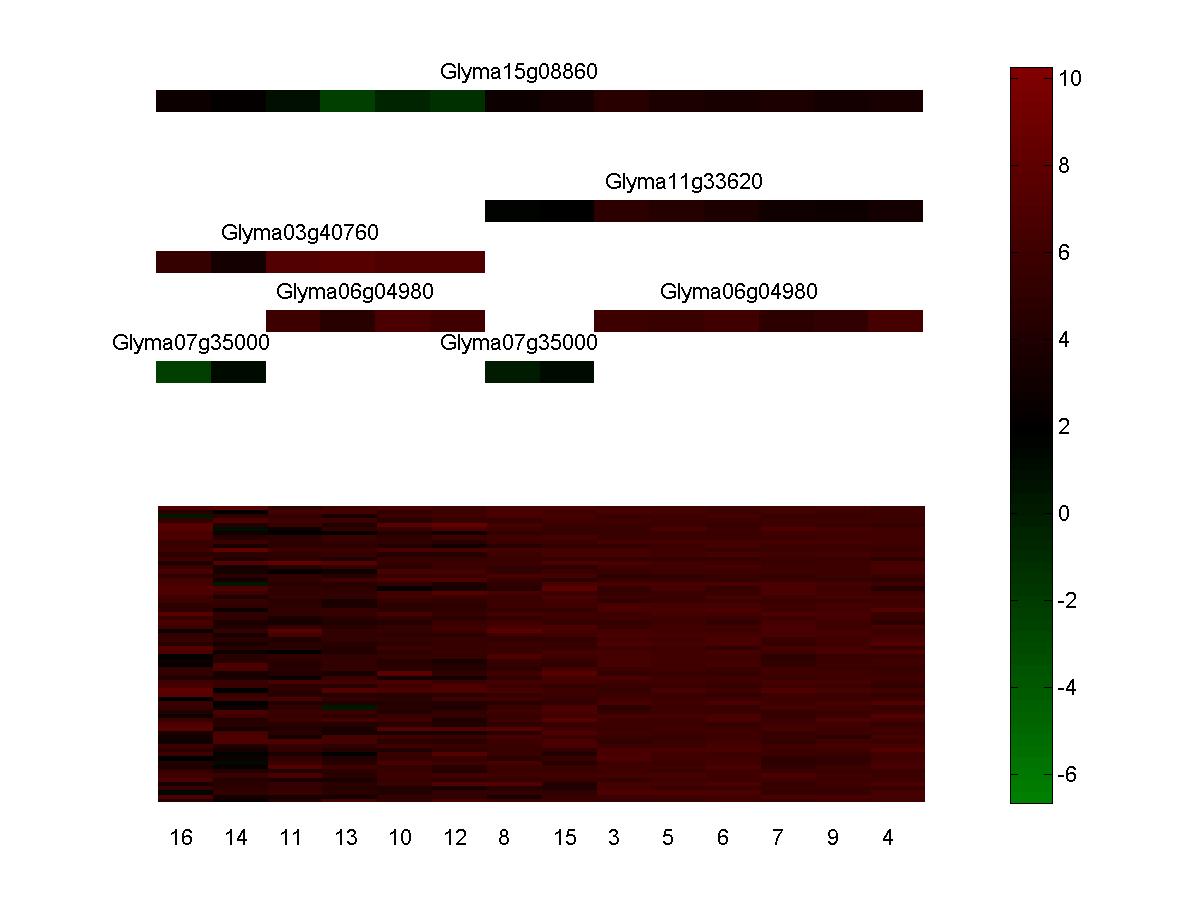


7 Glyma03g40760 AUX-IAA-ARF

7 Glyma15g08860 C2C2 (Zn) Dof

7 Glyma06g04980 LIM

7 Glyma11g33620 MYB/HD-like

7 Glyma07g35000 AS2

Glyma18g32830 Glyma01g02950 Glyma08g03540 Glyma13g31390 Glyma08g05820

Glyma11g05470 Glyma02g12930 Glyma12g04090 Glyma04g09500 Glyma15g41960

Glyma06g13840 Glyma10g35700 Glyma09g05810 Glyma10g06250 Glyma09g40980

Glyma11g21010 Glyma10g30440 Glyma04g37270 Glyma06g33380 Glyma16g29370

Glyma09g29960 Glyma03g37790 Glyma05g37900 Glyma10g42910 Glyma09g12320

Glyma08g18240 Glyma12g02030 Glyma20g29190 Glyma19g38600 Glyma08g07590

Glyma07g03230 Glyma07g05230 Glyma13g19830 Glyma05g03850 Glyma19g03500

Glyma07g02460 Glyma13g24500 Glyma08g29130 Glyma13g00380 Glyma04g40580

Glyma15g12240 Glyma15g06560 Glyma10g39750 Glyma17g14750 Glyma11g18980

Glyma15g01610 Glyma14g02070 Glyma03g36000 Glyma20g28860 Glyma19g30600

Glyma03g40860 Glyma01g45390 Glyma11g05900 Glyma04g05250 Glyma06g03100

Glyma13g05120 Glyma03g37780 Glyma03g16510 Glyma05g33860 Glyma13g44700

Glyma06g04980 Glyma15g09820 Glyma16g32290 Glyma08g01990 Glyma19g44780

Glyma13g39600 Glyma06g06420 Glyma08g06570 Glyma20g00910 Glyma09g00670

8


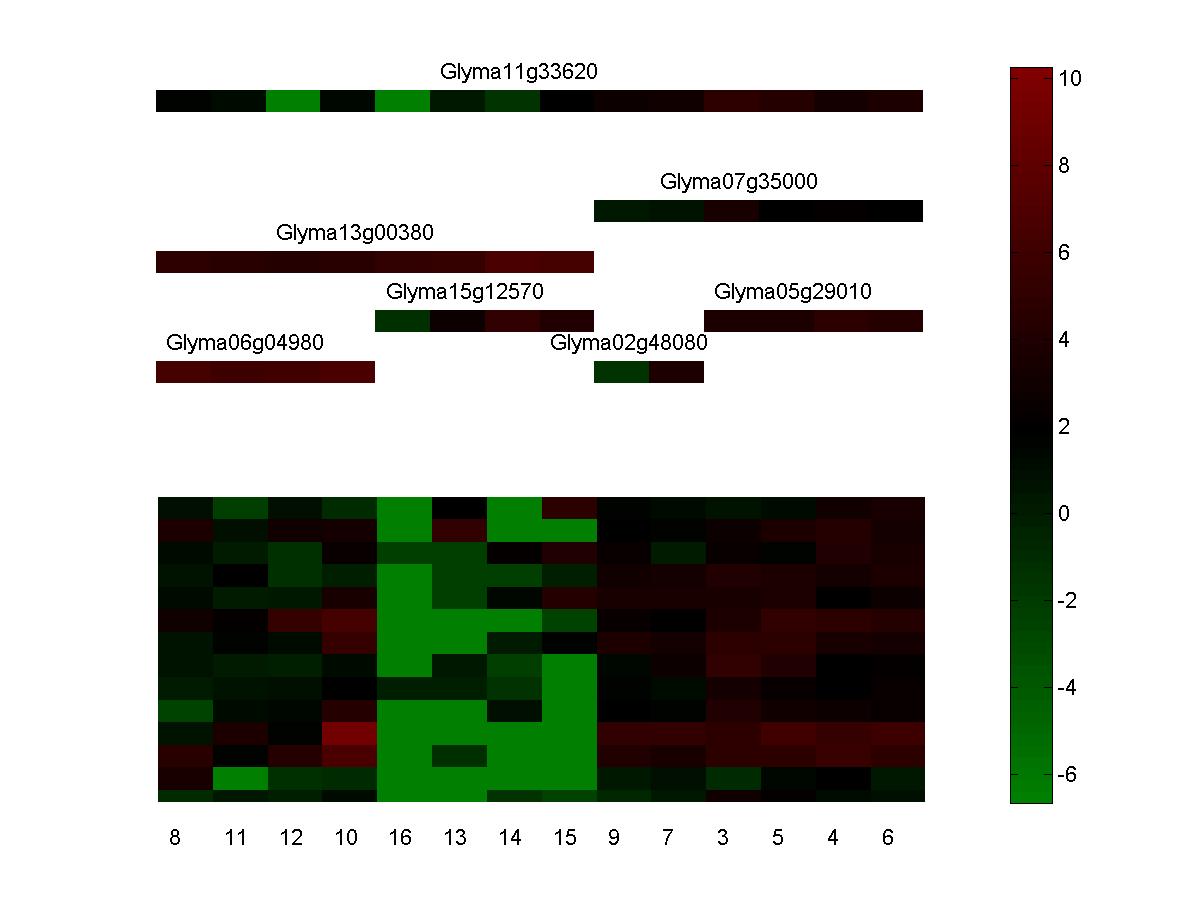


8 Glyma02g48080 Nin-like

8 Glyma15g12570 CCAAT

8 Glyma11g33620 MYB/HD-like

8 Glyma05g29010 AP2-EREBP

8 Glyma06g04980 LIM

8 Glyma07g35000 AS2

8 Glyma13g00380 WRKY

Glyma06g07780 Glyma09g32120 Glyma12g36360 Glyma15g26370 Glyma20g04130

Glyma04g08370 Glyma15g21830 Glyma08g09450 Glyma08g44030 Glyma04g10860

Glyma04g00710 Glyma08g45600 Glyma16g30040 Glyma18g53340

9


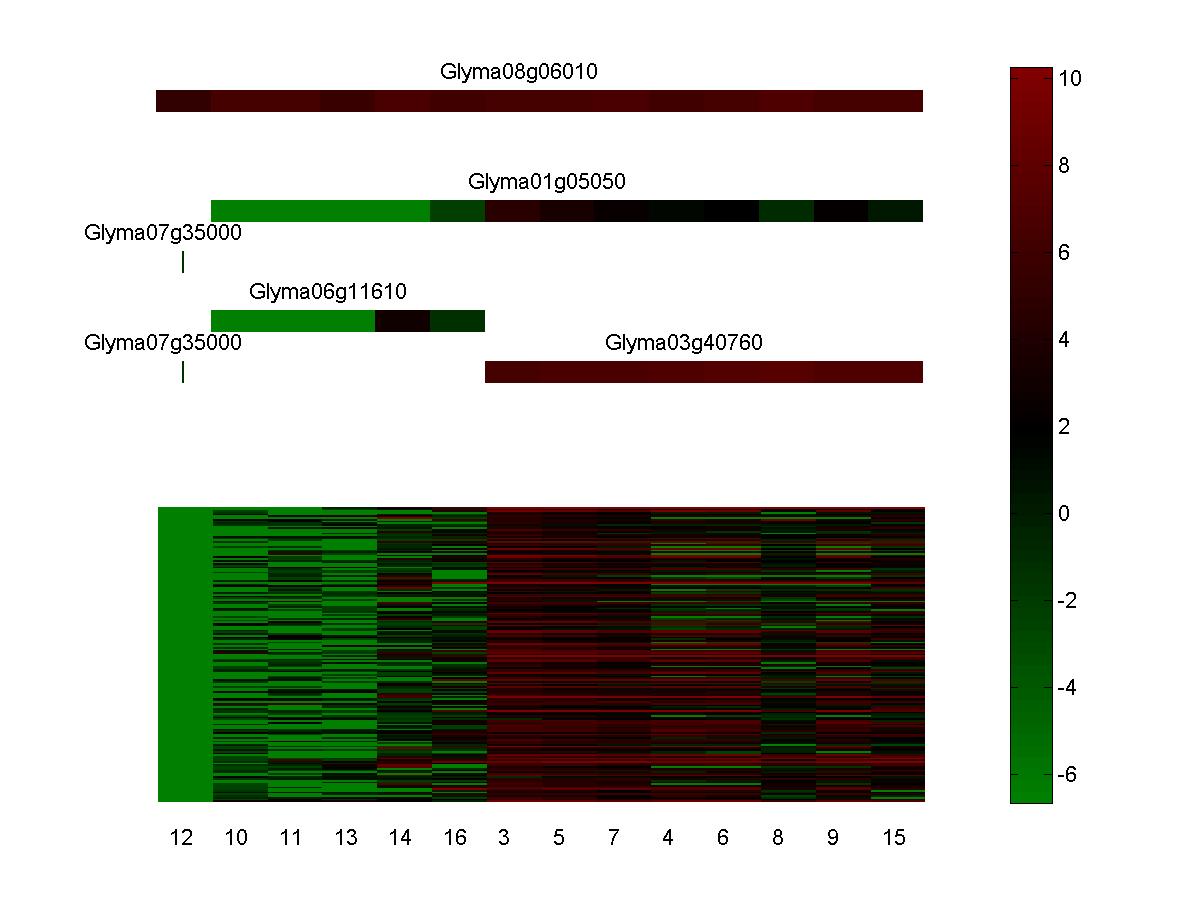


9 Glyma07g35000 AS2

9 Glyma03g40760 AUX-IAA-ARF

9 Glyma01g05050 WRKY

9 Glyma08g06010 TPR

9 Glyma06g11610 GRAS

Glyma08g24770 Glyma19g29000 Glyma02g01970 Glyma10g04150 Glyma16g04230

Glyma03g25650 Glyma08g08170 Glyma02g03280 Glyma11g13620 Glyma15g15600

Glyma05g25920 Glyma07g16940 Glyma18g48910 Glyma13g22350 Glyma20g30450

Glyma09g05150 Glyma19g38370 Glyma11g13270 Glyma19g40960 Glyma19g28760

Glyma19g45260 Glyma10g36300 Glyma15g13100 Glyma17g14260 Glyma11g29920

Glyma06g42040 Glyma07g16810 Glyma08g19250 Glyma18g06220 Glyma12g03050

Glyma07g39710 Glyma06g19870 Glyma20g06820 Glyma16g04220 Glyma02g43190

Glyma06g43630 Glyma13g23680 Glyma12g33750 Glyma13g16870 Glyma06g05370

Glyma01g42560 Glyma04g00210 Glyma11g08620 Glyma09g40590 Glyma17g02260

Glyma08g17270 Glyma08g03330 Glyma11g34380 Glyma16g32300 Glyma10g07410

Glyma06g43560 Glyma09g21250 Glyma11g34490 Glyma08g18030 Glyma07g32590

Glyma20g35570 Glyma09g33510 Glyma18g06350 Glyma04g03200 Glyma03g34440

Glyma01g40440 Glyma18g02100 Glyma05g25450 Glyma02g27090 Glyma20g29210

Glyma07g38620 Glyma10g25120 Glyma16g33790 Glyma10g05170 Glyma09g02610

Glyma11g22090 Glyma11g38150 Glyma15g15200 Glyma17g03360 Glyma07g04430

Glyma16g28610 Glyma09g07770 Glyma01g41930 Glyma20g24710 Glyma09g36620

Glyma11g35560 Glyma18g46500 Glyma10g38110 Glyma10g32070 Glyma20g38590

Glyma03g03460 Glyma15g15620 Glyma08g24760 Glyma09g28800 Glyma04g42130

Glyma15g02380 Glyma16g32470 Glyma15g13510 Glyma17g07440 Glyma01g42830

Glyma02g35350 Glyma03g33340 Glyma09g32630 Glyma16g22760 Glyma09g27600

Glyma15g35410 Glyma08g24720 Glyma13g42280 Glyma03g05510 Glyma06g11610

Glyma11g03310 Glyma02g09540 Glyma07g11080 Glyma18g44030 Glyma02g37340

Glyma02g36580 Glyma04g35130 Glyma11g33620 Glyma16g04980 Glyma05g33010

Glyma20g11610 Glyma07g04940 Glyma18g02090 Glyma01g42800 Glyma07g37270

Glyma08g37670 Glyma12g17680 Glyma06g47690 Glyma11g12060 Glyma15g42680

Glyma11g06070 Glyma15g07700 Glyma15g42760 Glyma16g06520 Glyma09g40580

Glyma16g22920 Glyma08g43040 Glyma20g28100 Glyma18g53740 Glyma16g03710

Glyma09g00800 Glyma05g02830 Glyma09g40740 Glyma10g31280 Glyma20g35010

Glyma10g02090 Glyma06g06930 Glyma16g34380 Glyma10g25130 Glyma10g38600

Glyma15g05760 Glyma08g04380 Glyma12g02240 Glyma06g12010 Glyma11g35030

Glyma07g34010 Glyma05g37420 Glyma10g10240 Glyma01g32070 Glyma07g17170

Glyma06g09000 Glyma03g37390 Glyma06g12670 Glyma19g03670 Glyma13g32310

Glyma17g02080 Glyma20g26140 Glyma02g48080 Glyma01g34580 Glyma17g15690

Glyma04g02230 Glyma05g29010 Glyma06g07160 Glyma18g43040 Glyma14g38370

Glyma06g06490 Glyma18g50320 Glyma09g31110

10


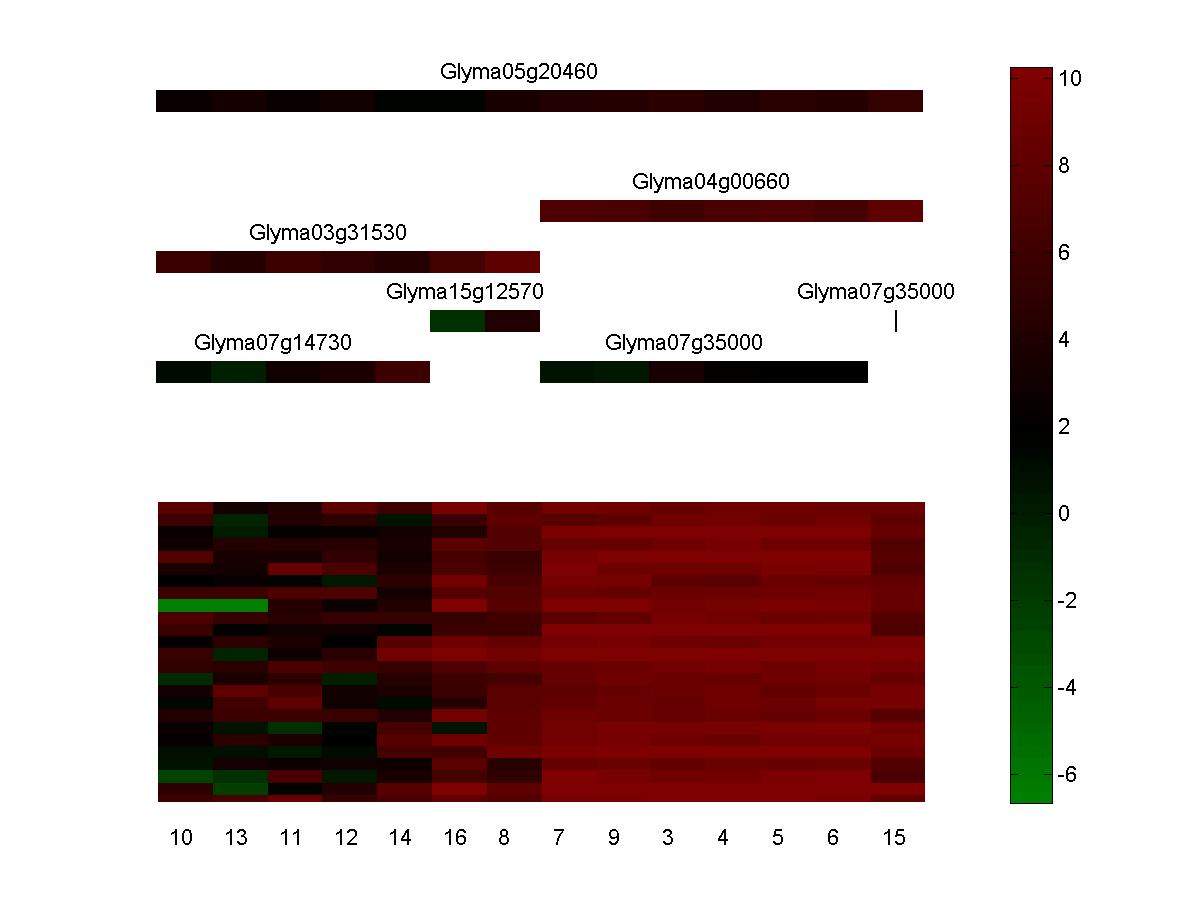


10 Glyma04g00660 CSD

10 Glyma05g20460 HSF

10 Glyma03g31530 AUX-IAA-ARF

10 Glyma15g12570 CCAAT

10 Glyma07g14730 PHD

10 Glyma07g35000 AS2

Glyma16g33710 Glyma09g02600 Glyma17g03350 Glyma03g26060 Glyma12g06100

Glyma07g38110 Glyma18g52250 Glyma12g29510 Glyma16g27900 Glyma20g26440

Glyma11g14140 Glyma07g32340 Glyma10g35080 Glyma16g28600 Glyma20g35630

Glyma11g00230 Glyma02g09220 Glyma11g33280 Glyma09g05440 Glyma07g32330

Glyma13g32300 Glyma08g47750 Glyma17g02600 Glyma10g35090 Glyma12g00400

11


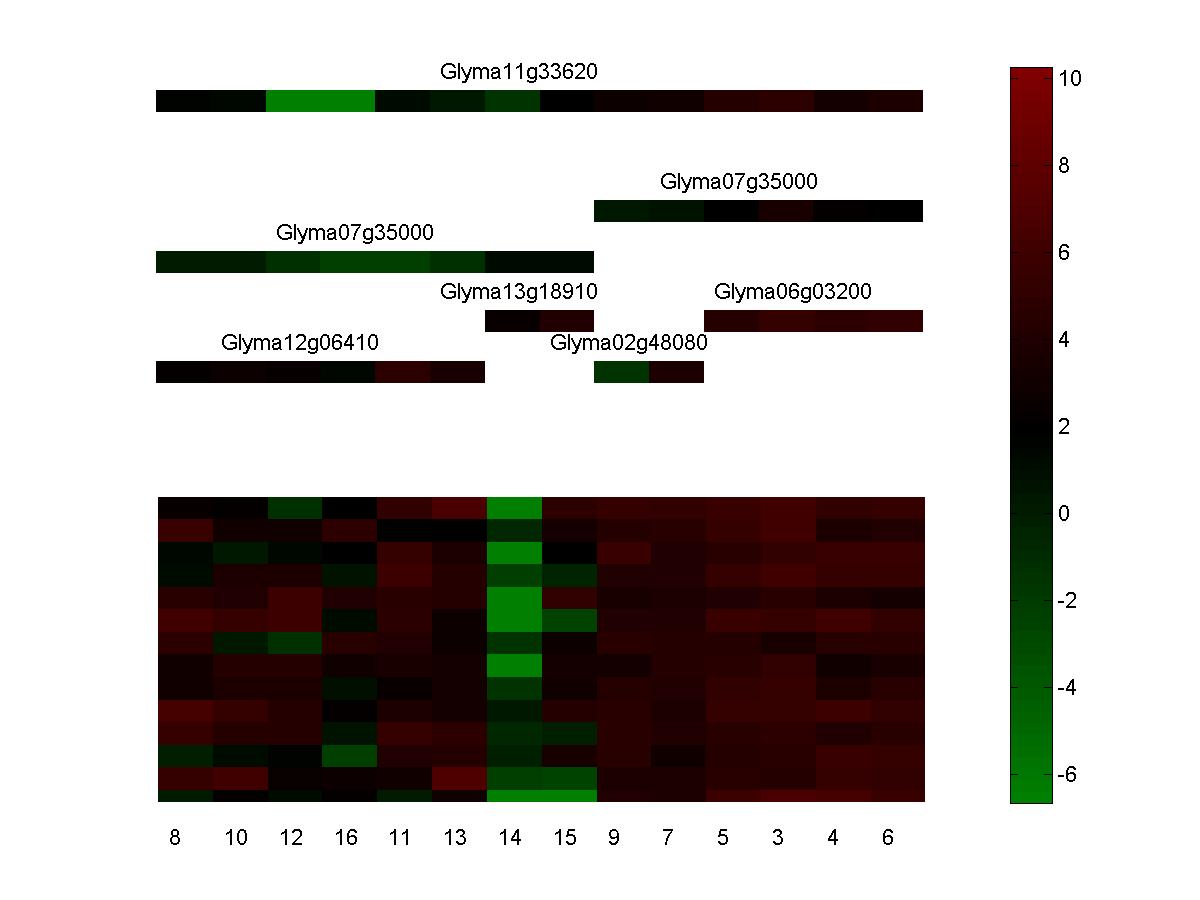


11 Glyma12g06410 MYB/HD-like

11 Glyma02g48080 Nin-like

11 Glyma11g33620 MYB/HD-like

11 Glyma13g18910 AUX-IAA-ARF

11 Glyma06g03200 Homeodomain/HOMEOBOX

11 Glyma07g35000 AS2

Glyma19g44360 Glyma15g14330 Glyma12g36300 Glyma01g27900 Glyma20g19200

Glyma01g04070 Glyma19g34370 Glyma02g11720 Glyma03g34800 Glyma13g31840

Glyma06g02380 Glyma10g38080 Glyma18g13840 Glyma12g02410

12


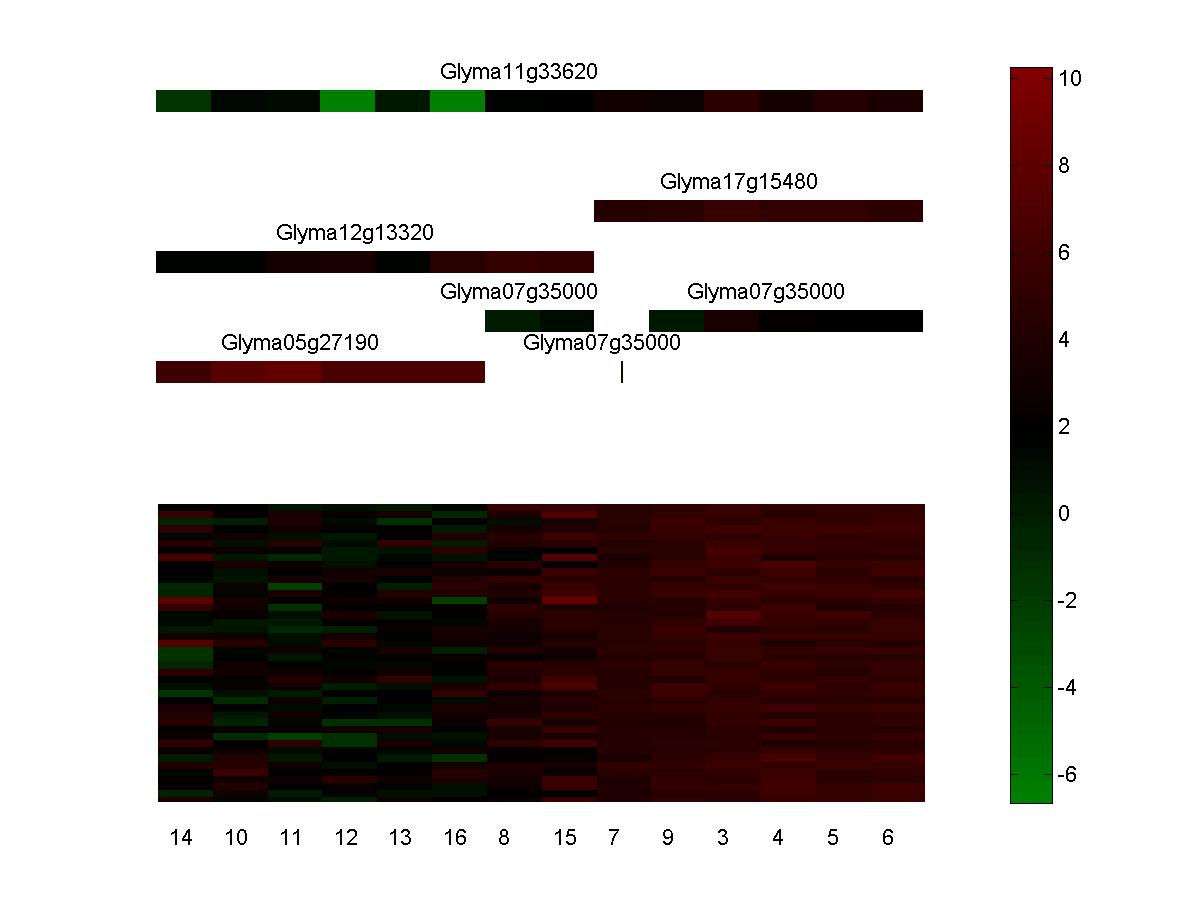


12 Glyma05g27190 GRAS

12 Glyma07g35000 AS2

12 Glyma17g15480 AP2-EREBP

12 Glyma12g13320 AP2-EREBP

12 Glyma11g33620 MYB/HD-like

Glyma18g45250 Glyma17g13390 Glyma07g38580 Glyma16g03960 Glyma06g08990

Glyma19g38940 Glyma07g05140 Glyma05g02690 Glyma15g14790 Glyma15g41700

Glyma12g13310 Glyma03g29770 Glyma11g03430 Glyma10g07710 Glyma01g34400

Glyma12g33420 Glyma19g42200 Glyma17g34590 Glyma02g41520 Glyma19g36800

Glyma06g02770 Glyma06g09220 Glyma04g33010 Glyma06g03200 Glyma07g30810

Glyma07g16850 Glyma12g11210 Glyma11g08770 Glyma15g24130 Glyma02g45940

Glyma06g42850 Glyma05g20460 Glyma13g01120 Glyma11g36010 Glyma13g35690

Glyma20g28490 Glyma13g36070 Glyma07g01220 Glyma12g12570 Glyma20g32140

Glyma02g46600 Glyma01g04380

13


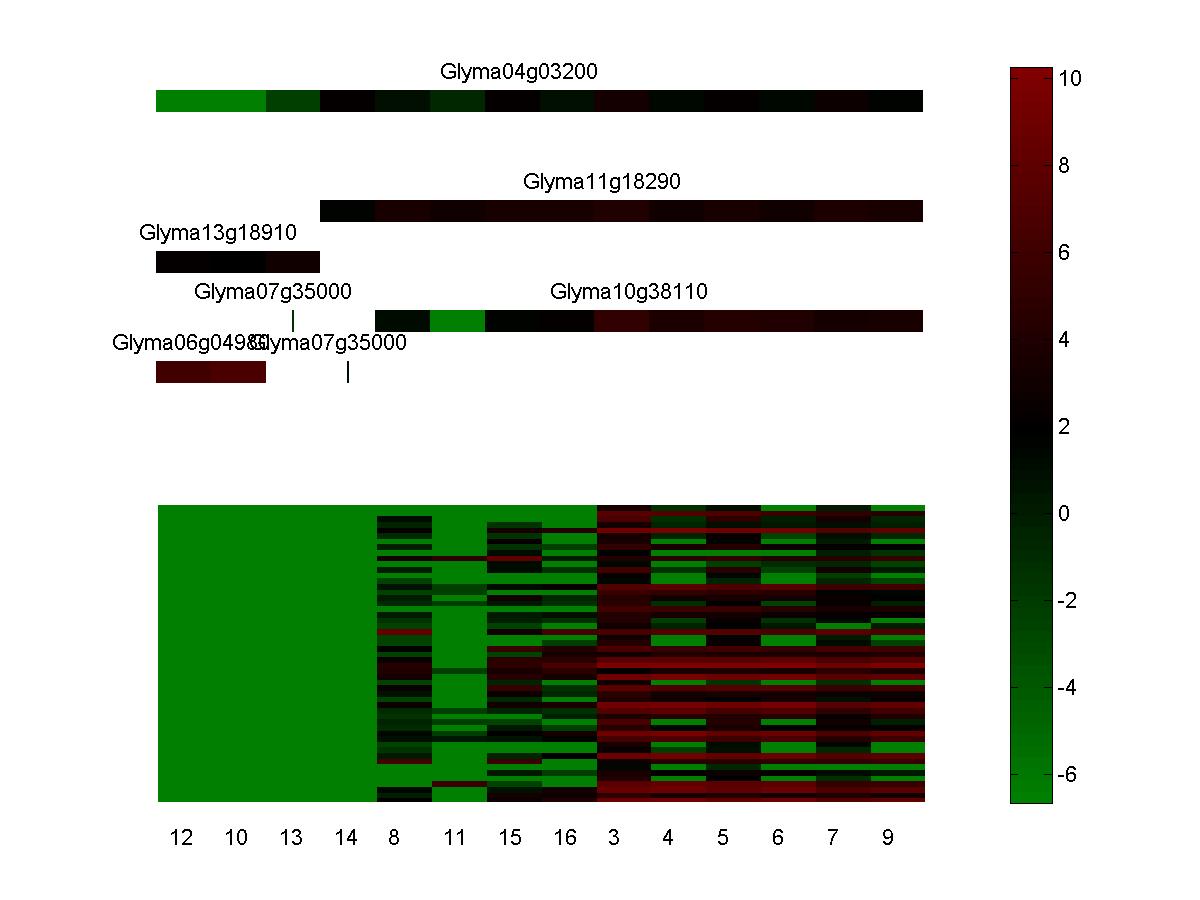


13 Glyma13g18910 AUX-IAA-ARF

13 Glyma04g03200 bHLH

13 Glyma11g18290 bHLH

13 Glyma07g35000 AS2

13 Glyma06g04980 LIM

13 Glyma10g38110 MYB/HD-like

Glyma11g34500 Glyma20g01420 Glyma01g45280 Glyma20g31820 Glyma18g02870

Glyma09g05290 Glyma06g29660 Glyma16g04410 Glyma08g37400 Glyma02g09210

Glyma18g50260 Glyma05g12090 Glyma08g09460 Glyma01g38600 Glyma11g03000

Glyma06g26610 Glyma03g05500 Glyma10g42840 Glyma14g05760 Glyma17g16990

Glyma05g12100 Glyma15g16610 Glyma07g04340 Glyma06g12510 Glyma11g03940

Glyma10g02730 Glyma13g35710 Glyma08g43550 Glyma03g04880 Glyma15g11140

Glyma01g42370 Glyma12g34510 Glyma01g31660 Glyma11g06740 Glyma18g16790

Glyma15g35390 Glyma01g34770 Glyma05g03720 Glyma08g05850 Glyma01g05050

Glyma02g16800 Glyma06g47190 Glyma04g42300 Glyma18g03150 Glyma10g29150

Glyma19g35270 Glyma11g34510 Glyma04g01730 Glyma15g36290 Glyma11g02350

Glyma09g00850 Glyma02g17060 Glyma03g37400

14


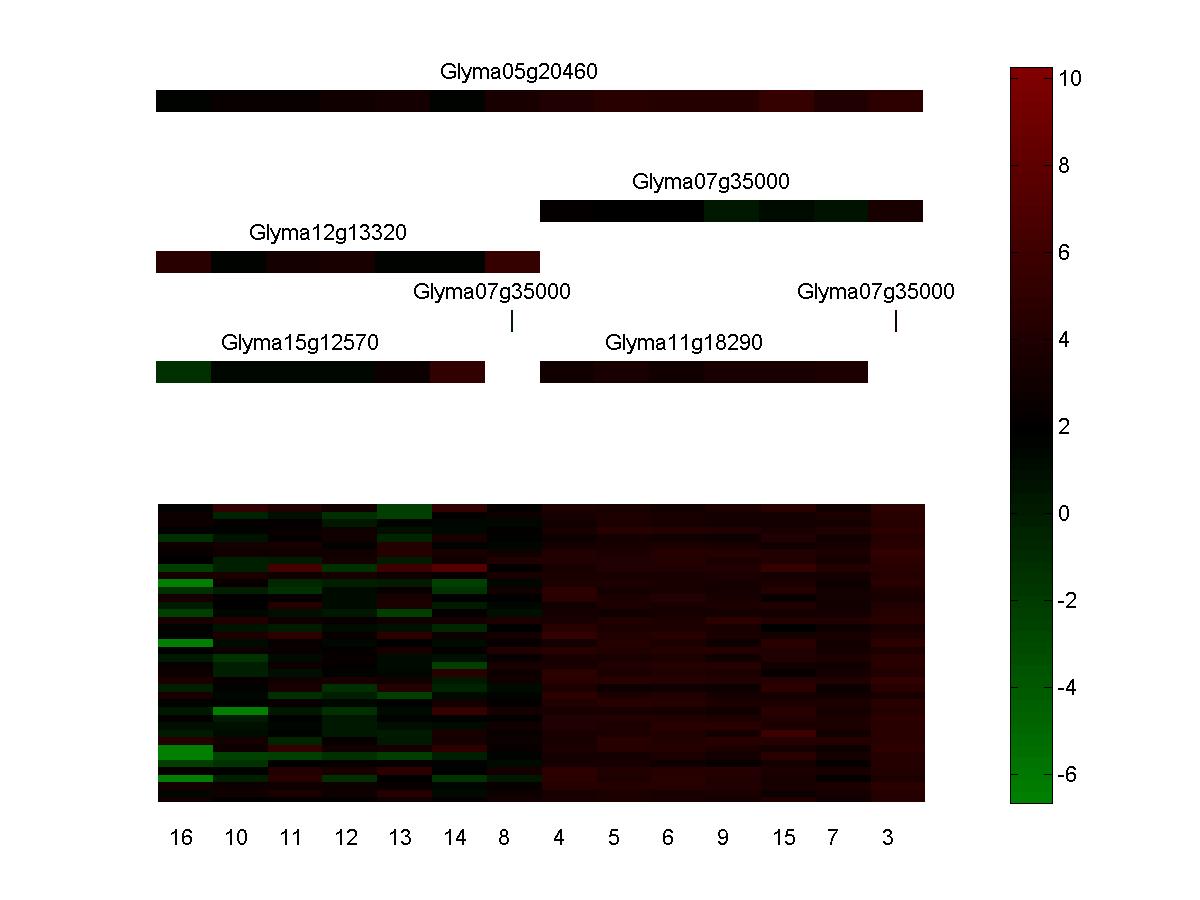


14 Glyma05g20460 HSF

14 Glyma12g13320 AP2-EREBP

14 Glyma07g35000 AS2

14 Glyma11g18290 bHLH

14 Glyma15g12570 CCAAT

Glyma13g31010 Glyma15g08860 Glyma13g24420 Glyma06g14370 Glyma10g42680

Glyma19g31770 Glyma13g29780 Glyma13g39690 Glyma08g02020 Glyma05g00570

Glyma20g03840 Glyma11g19920 Glyma12g00790 Glyma18g50310 Glyma13g36740

Glyma19g38130 Glyma17g07270 Glyma17g02570 Glyma10g39040 Glyma13g34340

Glyma18g19080 Glyma20g22290 Glyma07g34300 Glyma20g35260 Glyma17g33190

Glyma01g32130 Glyma08g12610 Glyma20g01670 Glyma01g43280 Glyma18g05710

Glyma16g34840 Glyma11g09920 Glyma03g18410 Glyma20g28790 Glyma08g40360

Glyma16g34770 Glyma05g28810 Glyma19g43390 Glyma19g34250 Glyma13g18910

15


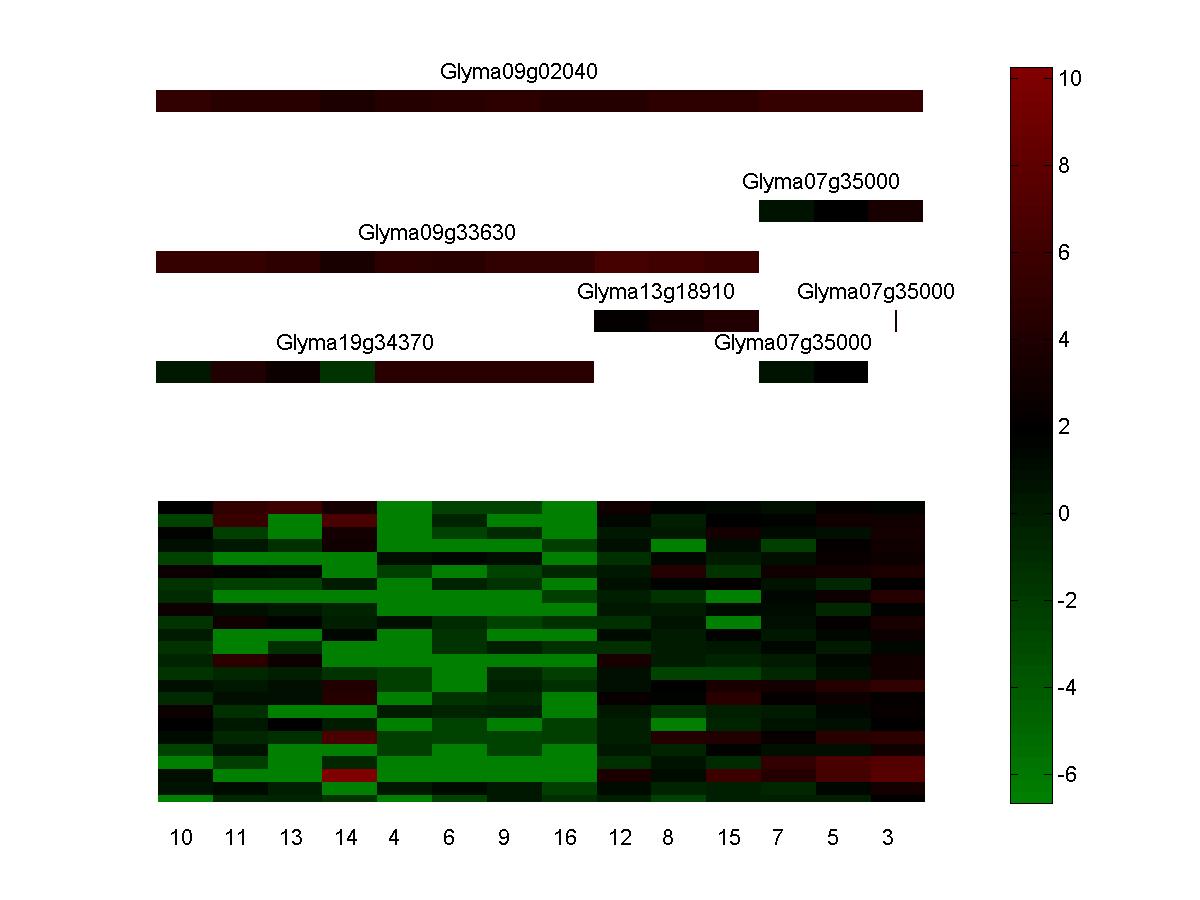


15 Glyma07g35000 AS2

15 Glyma09g33630 AUX-IAA-ARF

15 Glyma13g18910 AUX-IAA-ARF

15 Glyma09g02040 MYB/HD-like

15 Glyma19g34370 AUX-IAA-ARF

Glyma17g09200 Glyma07g09710 Glyma17g07830 Glyma17g35230 Glyma12g10760

Glyma15g01500 Glyma15g11040 Glyma11g37620 Glyma14g11320 Glyma13g28970

Glyma18g53450 Glyma07g09970 Glyma09g28750 Glyma18g52730 Glyma19g38800

Glyma03g36140 Glyma18g46690 Glyma07g35770 Glyma01g03470 Glyma07g00840

Glyma05g03750 Glyma08g12650 Glyma08g48160 Glyma10g43520

16


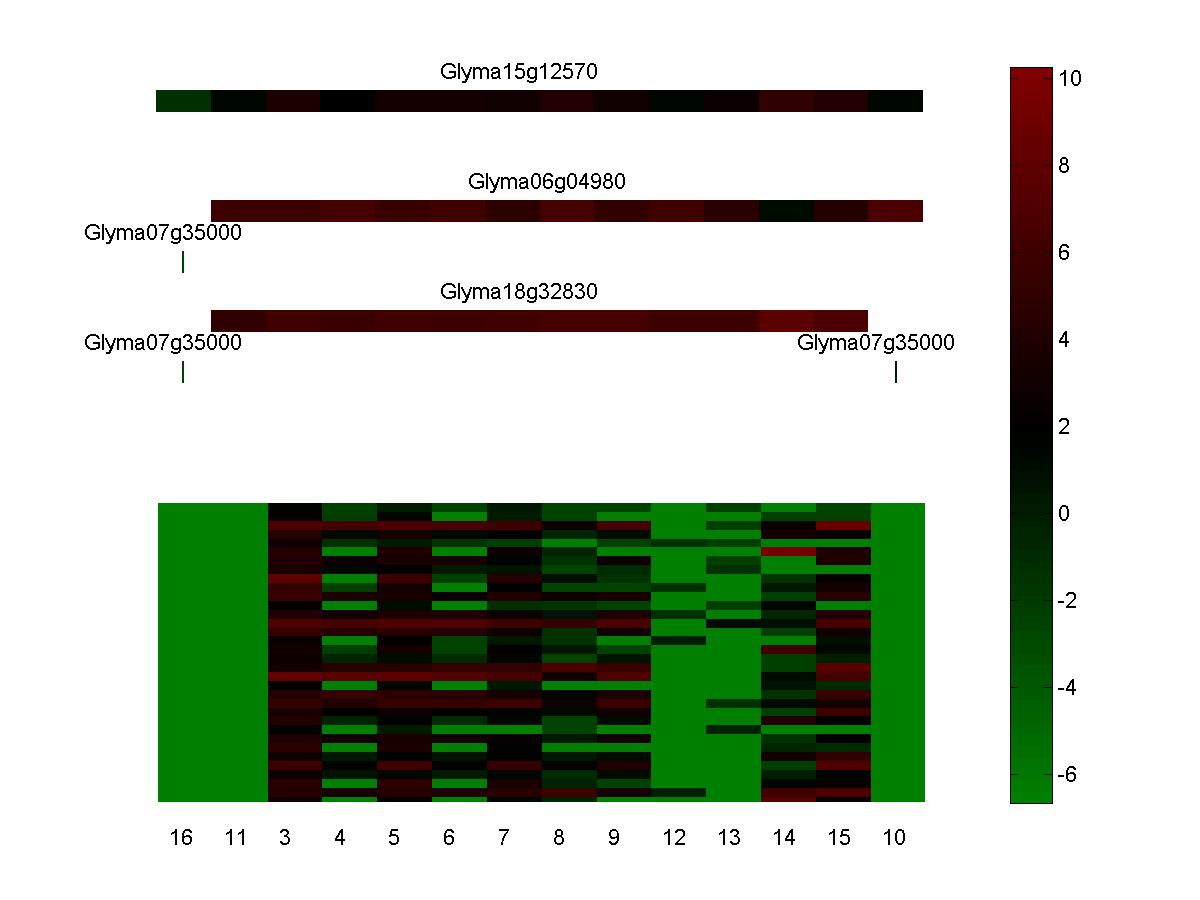


16 Glyma18g32830 TPR

16 Glyma15g12570 CCAAT

16 Glyma06g04980 LIM

16 Glyma07g35000 AS2

Glyma19g38140 Glyma09g24130 Glyma11g14120 Glyma16g01020 Glyma19g29160

Glyma09g31910 Glyma18g44010 Glyma17g31900 Glyma20g34830 Glyma06g29670

Glyma18g06230 Glyma12g16410 Glyma09g02190 Glyma08g27070 Glyma20g08560

Glyma18g00350 Glyma13g27300 Glyma02g02430 Glyma16g07830 Glyma02g40010

Glyma06g19620 Glyma09g02910 Glyma01g26750 Glyma16g06530 Glyma04g05230

Glyma10g34060 Glyma14g12710 Glyma01g38040 Glyma17g34880 Glyma16g06500

Glyma09g37470 Glyma13g23770 Glyma02g25950 Glyma08g24680

17


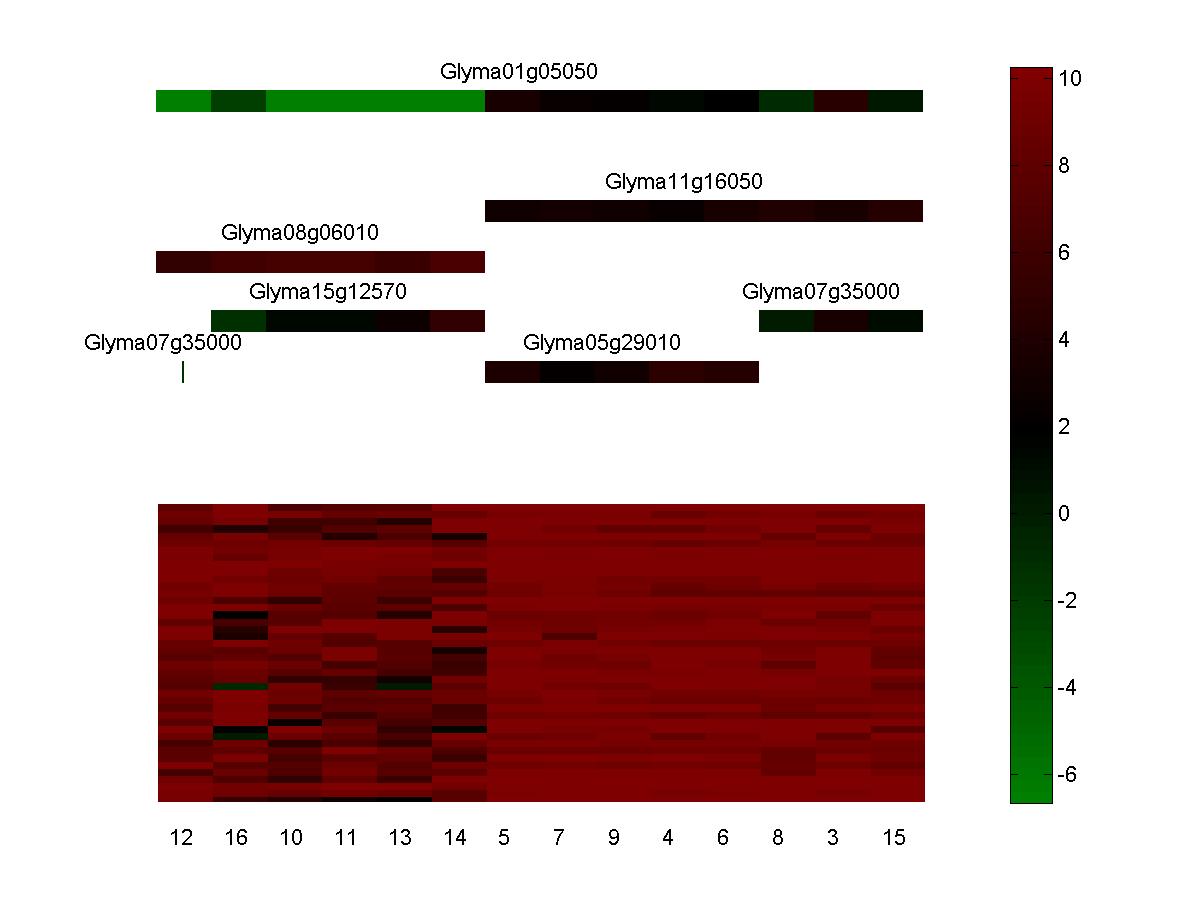


17 Glyma08g06010 TPR

17 Glyma07g35000 AS2

17 Glyma01g05050 WRKY

17 Glyma15g12570 CCAAT

17 Glyma11g16050 BZIP

17 Glyma05g29010 AP2-EREBP

Glyma13g42330 Glyma08g18110 Glyma07g00900 Glyma17g34870 Glyma05g37730

Glyma12g02790 Glyma20g27940 Glyma10g39780 Glyma17g23900 Glyma03g34310

Glyma19g37000 Glyma05g11630 Glyma10g05580 Glyma09g12250 Glyma08g11480

Glyma12g34550 Glyma15g19580 Glyma19g29210 Glyma19g36440 Glyma19g35570

Glyma16g04190 Glyma04g01130 Glyma19g29180 Glyma13g40100 Glyma07g00910

Glyma08g21410 Glyma03g32850 Glyma19g35560 Glyma02g04510 Glyma08g46520

Glyma11g03690 Glyma07g01730 Glyma12g34570 Glyma06g19820 Glyma14g09440

Glyma01g03070 Glyma17g23870 Glyma12g00390 Glyma09g12260 Glyma20g27950

Glyma02g42220 Glyma09g12200

18


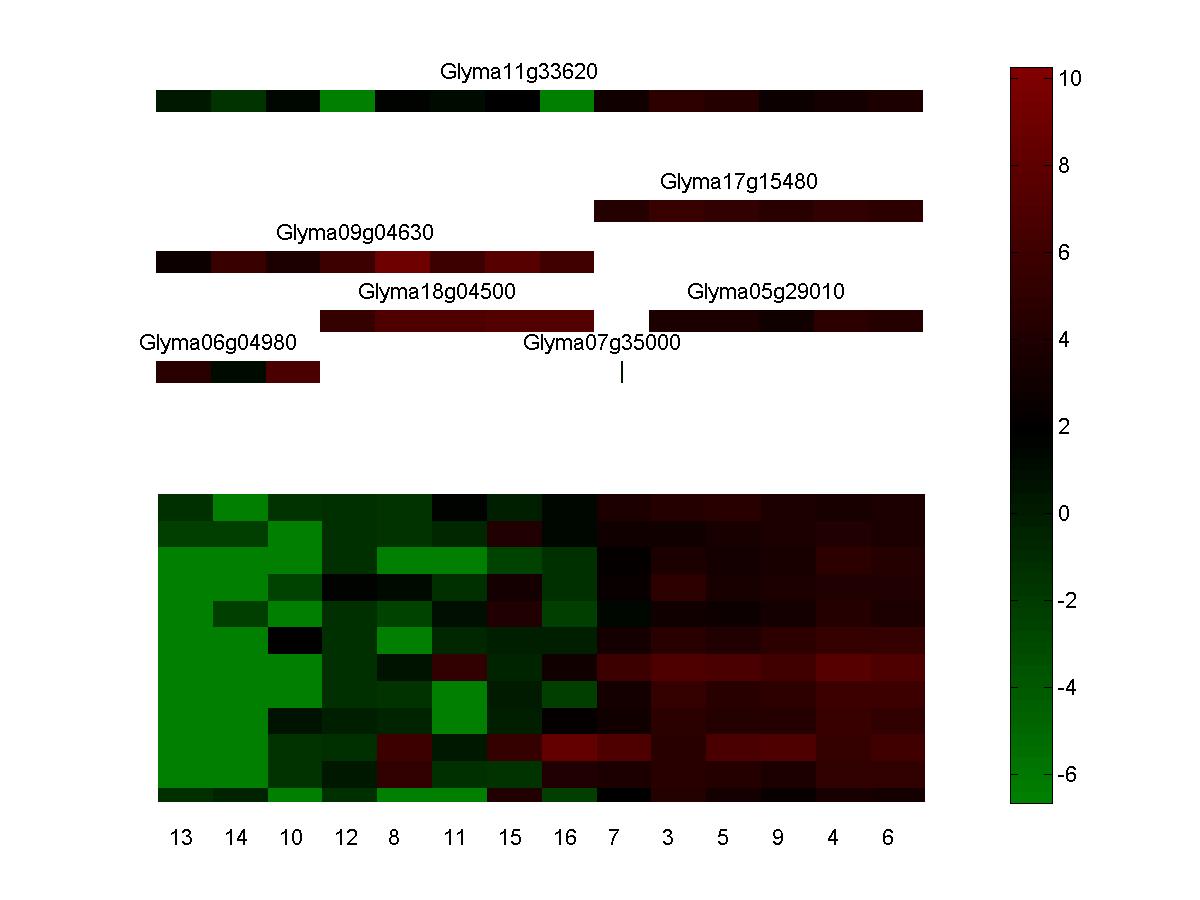


18 Glyma18g04500 GRAS

18 Glyma17g15480 AP2-EREBP

18 Glyma09g04630 AP2-EREBP

18 Glyma07g35000 AS2

18 Glyma11g33620 MYB/HD-like

18 Glyma05g29010 AP2-EREBP

18 Glyma06g04980 LIM

Glyma03g37310 Glyma10g05210 Glyma07g37260 Glyma17g07350 Glyma12g04850

Glyma16g26940 Glyma13g27130 Glyma15g11700 Glyma02g40890 Glyma06g16810

Glyma17g16620 Glyma07g36860

19


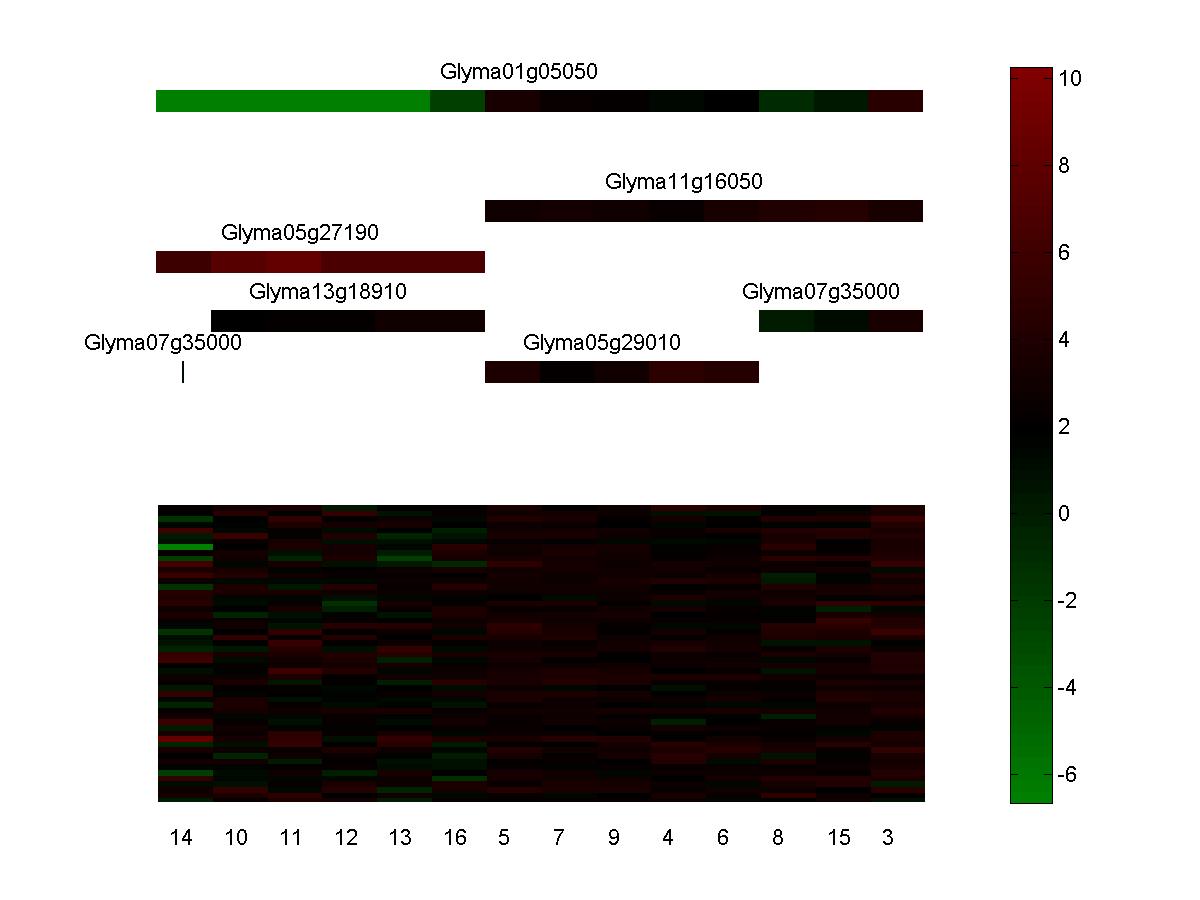


19 Glyma05g29010 AP2-EREBP

19 Glyma13g18910 AUX-IAA-ARF

19 Glyma11g16050 BZIP

19 Glyma07g35000 AS2

19 Glyma01g05050 WRKY

19 Glyma05g27190 GRAS

Glyma18g06140 Glyma01g01320 Glyma14g06650 Glyma02g03530 Glyma11g16050

Glyma04g34160 Glyma15g19390 Glyma15g30150 Glyma07g04090 Glyma12g06030

Glyma11g07750 Glyma03g00420 Glyma16g25190 Glyma06g34940 Glyma09g11990

Glyma16g24890 Glyma12g32810 Glyma20g29200 Glyma05g32920 Glyma09g34460

Glyma04g02560 Glyma03g05460 Glyma14g06640 Glyma12g04460 Glyma20g29930

Glyma02g11610 Glyma16g06410 Glyma07g14730 Glyma15g04790 Glyma04g40400

Glyma04g38830 Glyma18g22780 Glyma0092s00200 Glyma03g33290 Glyma02g09240

Glyma02g13910 Glyma07g04520 Glyma09g34420 Glyma03g22930 Glyma13g21660

Glyma12g06410 Glyma13g43920 Glyma03g24020 Glyma20g22700 Glyma09g07010

Glyma09g01430 Glyma12g10400 Glyma04g02750 Glyma15g12570 Glyma11g36730

Glyma09g03400 Glyma15g06220 Glyma11g19430

20


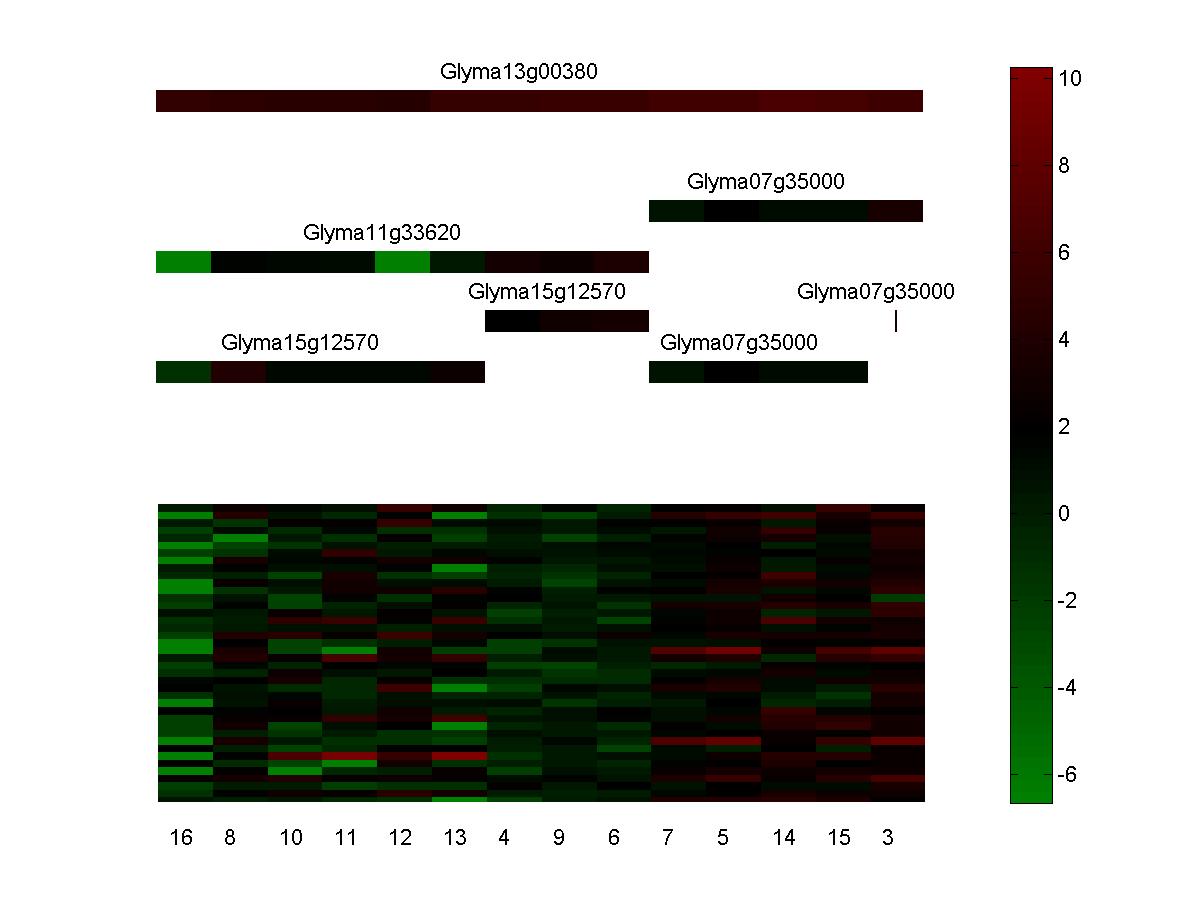


20 Glyma11g33620 MYB/HD-like

20 Glyma13g00380 WRKY

20 Glyma15g12570 CCAAT

20 Glyma07g35000 AS2

Glyma05g15700 Glyma02g04180 Glyma12g34610 Glyma11g04620 Glyma08g48030

Glyma17g08550 Glyma02g26160 Glyma18g15530 Glyma15g15610 Glyma13g38710

Glyma08g23310 Glyma16g06740 Glyma19g11560 Glyma02g35190 Glyma08g17200

Glyma02g06730 Glyma04g11000 Glyma11g05010 Glyma13g35260 Glyma14g05840

Glyma02g42250 Glyma01g04190 Glyma17g14800 Glyma10g07500 Glyma03g28080

Glyma08g48140 Glyma04g06450 Glyma08g19140 Glyma18g47820 Glyma02g13800

Glyma03g22960 Glyma02g42730 Glyma10g08860 Glyma20g27990 Glyma15g06140

Glyma10g11060 Glyma17g17310 Glyma07g35000 Glyma19g22460 Glyma19g44060

21


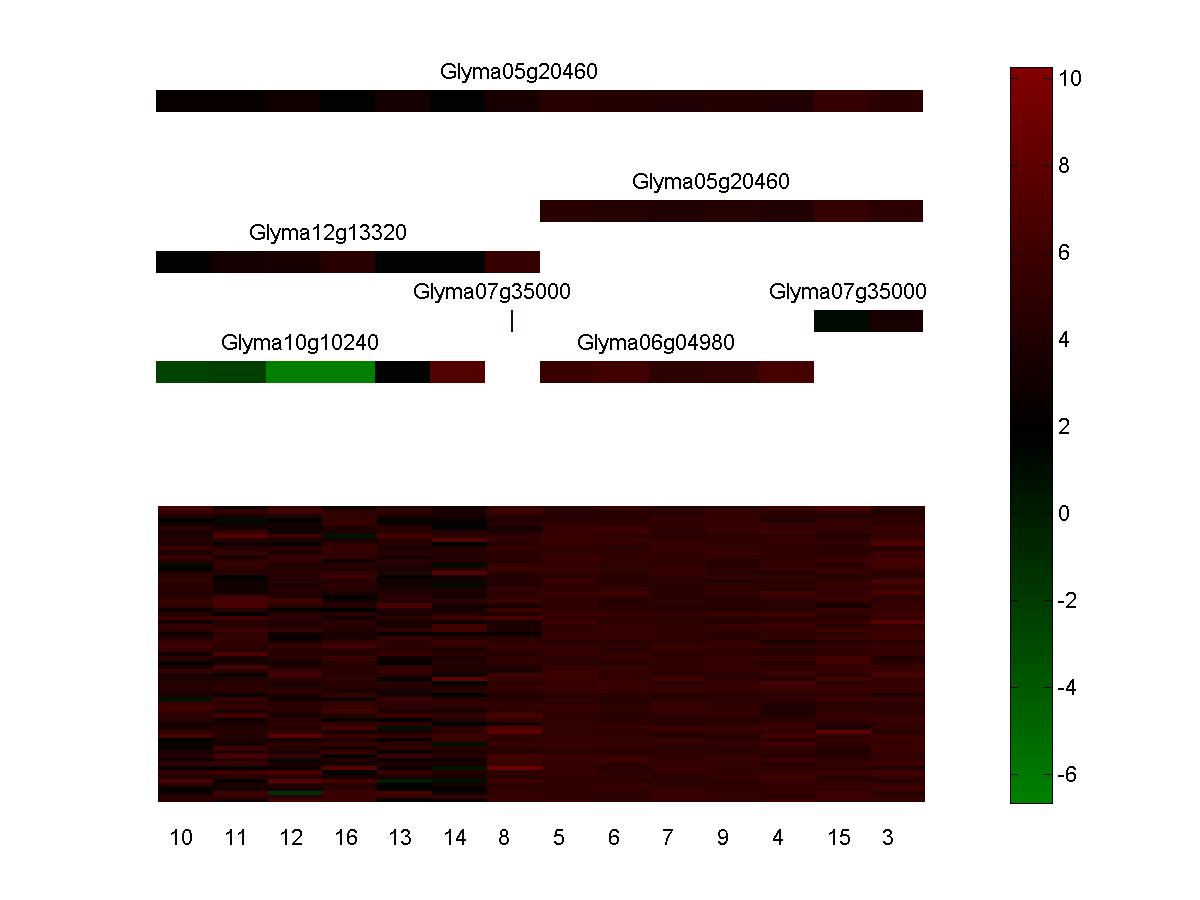


21 Glyma12g13320 AP2-EREBP

21 Glyma10g10240 CCAAT

21 Glyma07g35000 AS2

21 Glyma06g04980 LIM

21 Glyma05g20460 HSF

Glyma06g14200 Glyma09g33630 Glyma08g03880 Glyma03g38120 Glyma06g05320

Glyma16g32280 Glyma10g05480 Glyma11g00260 Glyma18g53990 Glyma18g47360

Glyma20g35990 Glyma01g05340 Glyma11g12260 Glyma12g30660 Glyma06g44430

Glyma16g08360 Glyma05g37590 Glyma06g05470 Glyma02g04760 Glyma19g36620

Glyma03g03190 Glyma15g00600 Glyma18g53950 Glyma08g47310 Glyma09g29840

Glyma12g30620 Glyma16g04800 Glyma04g42160 Glyma12g01970 Glyma19g16450

Glyma16g29900 Glyma05g37690 Glyma04g35600 Glyma13g28330 Glyma04g38870

Glyma09g02040 Glyma12g29630 Glyma07g29670 Glyma17g14520 Glyma13g40180

Glyma03g35000 Glyma04g03270 Glyma06g05460 Glyma10g30020 Glyma07g07970

Glyma18g52590 Glyma02g47690 Glyma17g15480 Glyma04g02520 Glyma08g13320

Glyma12g32850 Glyma13g28390 Glyma06g14220 Glyma20g32050 Glyma13g07110

Glyma08g41960 Glyma06g17860 Glyma07g17250 Glyma08g16310 Glyma02g11740

Glyma16g01640 Glyma16g04420 Glyma17g28450 Glyma16g04560 Glyma07g39540

Glyma13g07900 Glyma20g37370 Glyma13g26600 Glyma18g51260 Glyma12g13320

Glyma14g40170 Glyma16g24140 Glyma08g07490

22


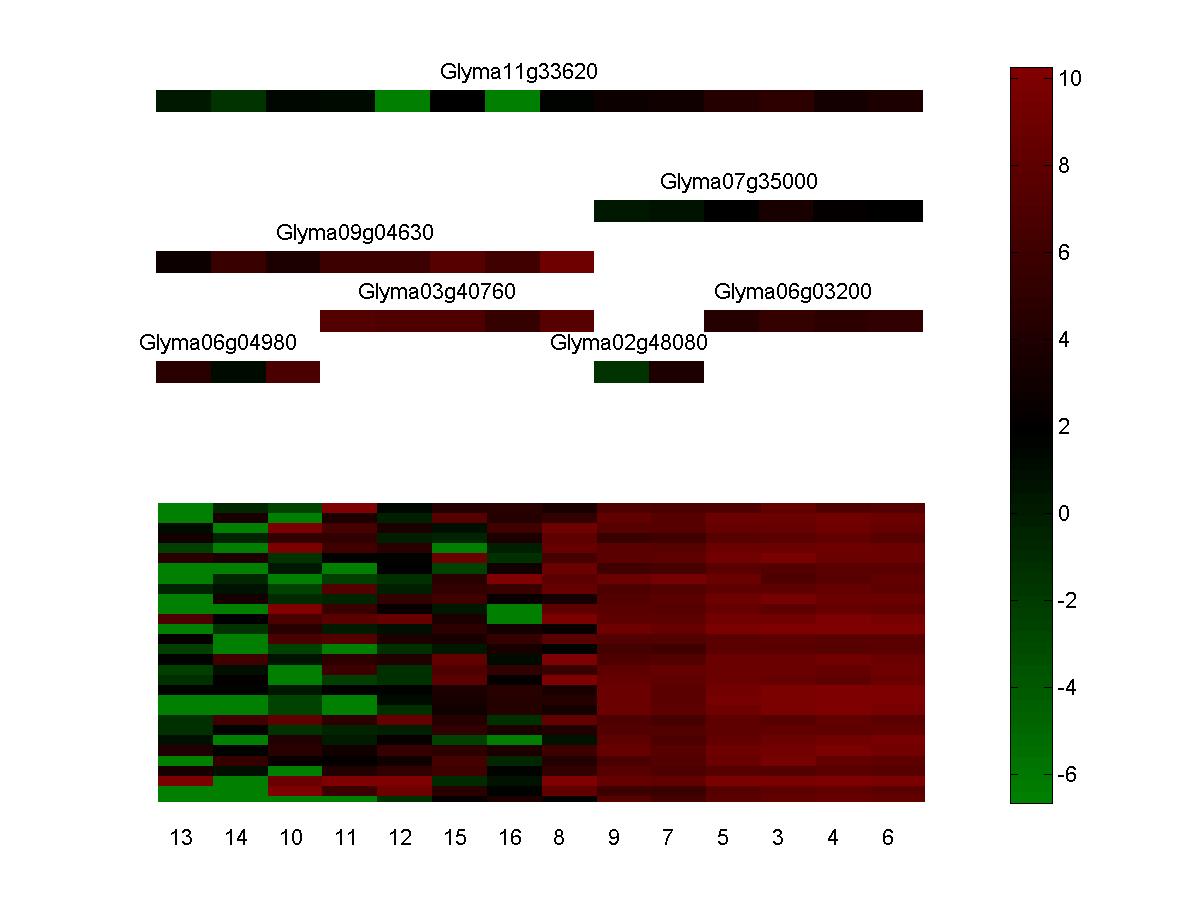


22 Glyma06g03200 Homeodomain/HOMEOBOX

22 Glyma06g04980 LIM

22 Glyma07g35000 AS2

22 Glyma02g48080 Nin-like

22 Glyma09g04630 AP2-EREBP

22 Glyma03g40760 AUX-IAA-ARF

22 Glyma11g33620 MYB/HD-like

Glyma13g20170 Glyma08g45520 Glyma18g44300 Glyma03g04920 Glyma14g05650

Glyma01g31750 Glyma03g04960 Glyma06g02290 Glyma01g42670 Glyma11g05800

Glyma14g05640 Glyma17g03740 Glyma12g06110 Glyma19g43460 Glyma06g47730

Glyma15g23830 Glyma10g05800 Glyma15g31520 Glyma19g37240 Glyma09g05340

Glyma17g14230 Glyma02g18090 Glyma18g41910 Glyma15g09540 Glyma01g26840

Glyma01g39460 Glyma08g24750 Glyma01g32750 Glyma08g21390 Glyma08g17300

23


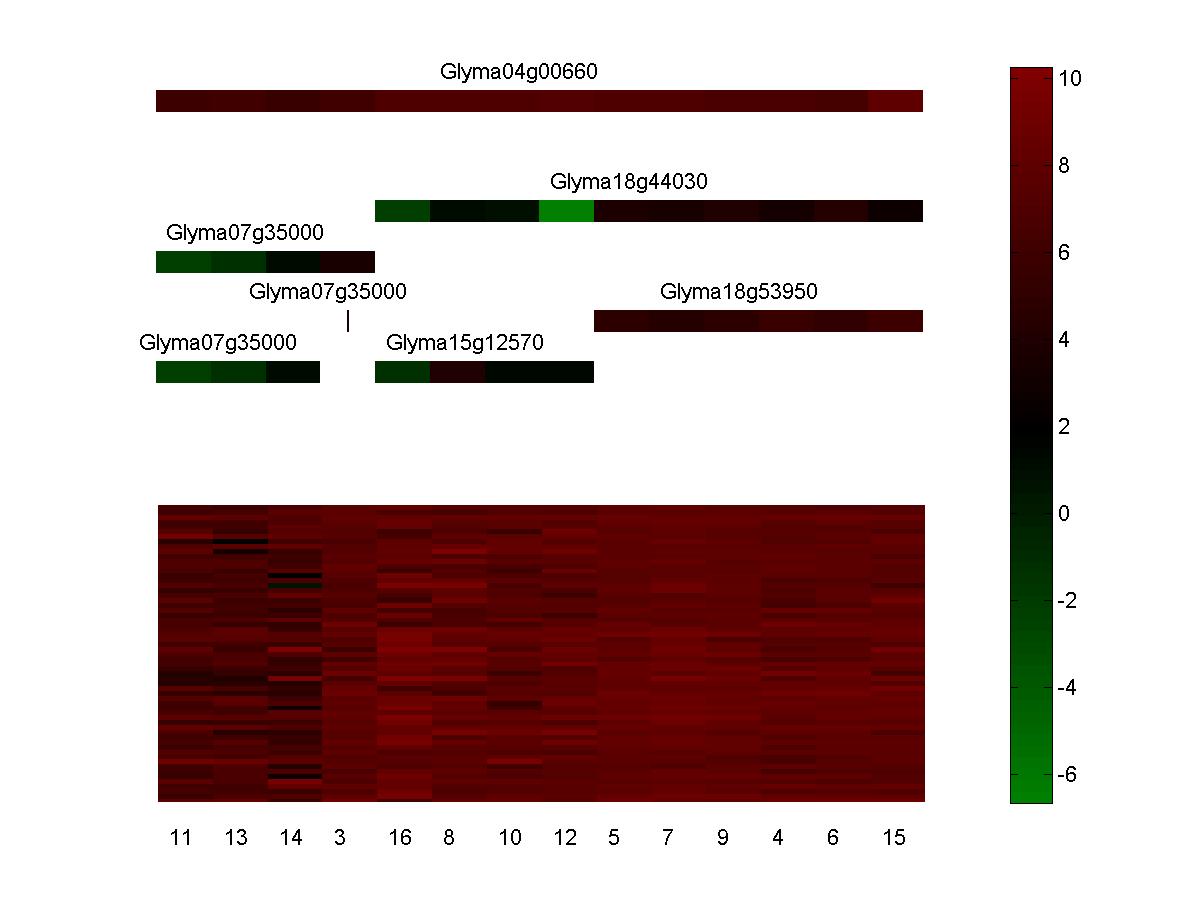


23 Glyma15g12570 CCAAT

23 Glyma18g53950 NAC

23 Glyma04g00660 CSD

23 Glyma18g44030 WRKY

23 Glyma07g35000 AS2

Glyma08g03150 Glyma06g03150 Glyma19g03040 Glyma06g20540 Glyma02g47210

Glyma17g23860 Glyma06g03050 Glyma04g09350 Glyma05g32100 Glyma10g31590

Glyma08g04740 Glyma13g06390 Glyma19g44160 Glyma20g05560 Glyma06g02650

Glyma20g30970 Glyma13g22940 Glyma04g06700 Glyma07g00760 Glyma12g08040

Glyma14g06170 Glyma02g37080 Glyma08g09200 Glyma08g14130 Glyma13g00450

Glyma19g03960 Glyma07g34440 Glyma17g34920 Glyma16g24120 Glyma09g01320

Glyma17g09280 Glyma12g08990 Glyma06g11080 Glyma03g36560 Glyma19g32990

Glyma15g12170 Glyma11g21020 Glyma02g40290 Glyma02g09200 Glyma19g03950

Glyma14g00720 Glyma10g35520 Glyma20g24280 Glyma05g24110 Glyma05g01180

Glyma19g03030 Glyma08g14550 Glyma14g06630 Glyma08g11490 Glyma20g02170

Glyma0169s00210 Glyma05g36420 Glyma11g12510 Glyma06g13760 Glyma07g04890

Glyma04g02610 Glyma06g00990 Glyma11g15680 Glyma05g34570 Glyma08g25950

Glyma10g06600

24


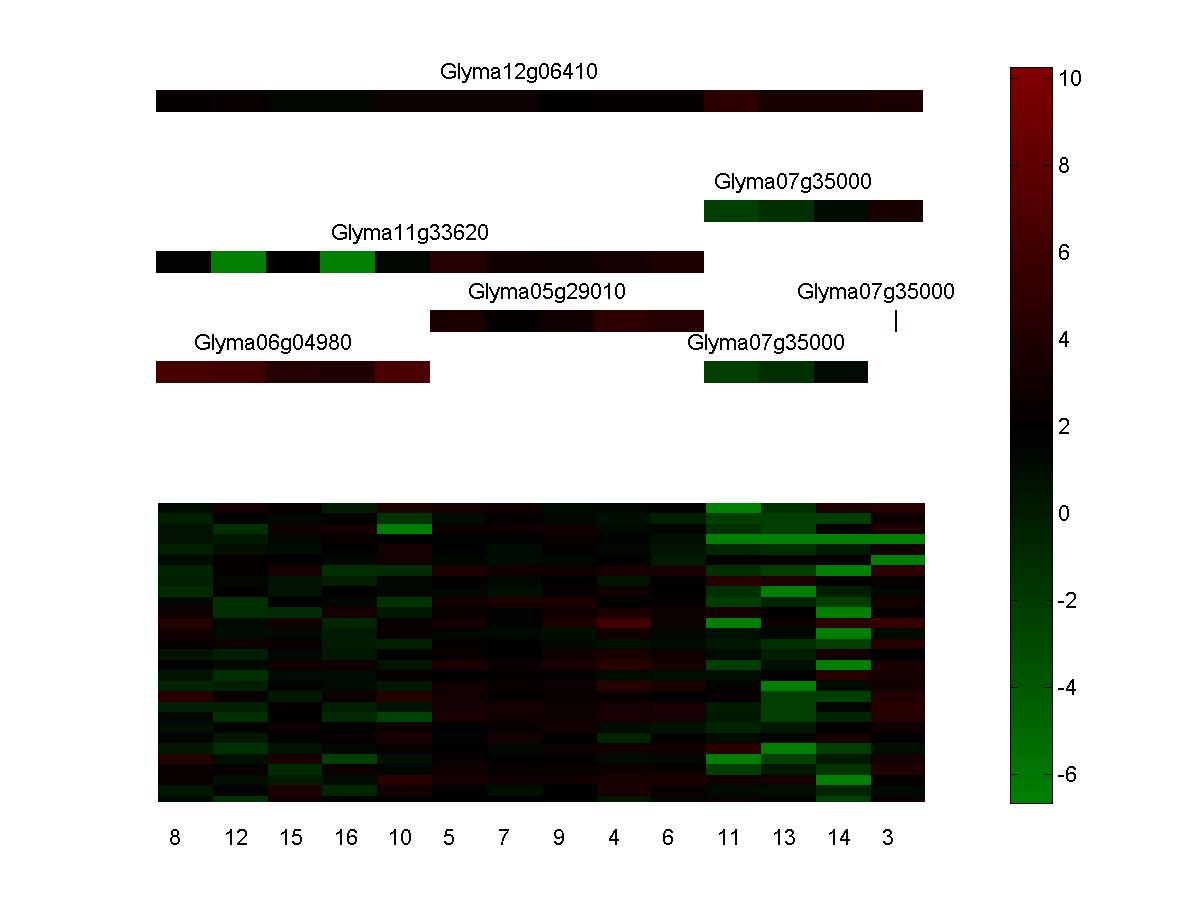


24 Glyma12g06410 MYB/HD-like

24 Glyma06g04980 LIM

24 Glyma11g33620 MYB/HD-like

24 Glyma05g29010 AP2-EREBP

24 Glyma07g35000 AS2

Glyma12g02590 Glyma13g08490 Glyma13g38470 Glyma06g43710 Glyma17g08930

Glyma13g26170 Glyma04g43090 Glyma04g41510 Glyma05g30410 Glyma13g32030

Glyma03g31520 Glyma04g08090 Glyma13g21970 Glyma03g28640 Glyma19g16460

Glyma02g12860 Glyma09g28890 Glyma15g03130 Glyma17g03090 Glyma17g18040

Glyma08g13180 Glyma13g19620 Glyma04g42790 Glyma08g21460 Glyma07g13840

Glyma17g10720 Glyma19g38870 Glyma13g43990 Glyma04g41820

DEG genes List

Glyma20g30910 Glyma13g20170 Glyma18g45250 Glyma17g09200 Glyma18g32830 Glyma13g31010 Glyma18g06140 Glyma06g07780 Glyma01g02950 Glyma05g15700 Glyma01g01320 Glyma02g04180 Glyma08g03150 Glyma15g08860 Glyma13g42330 Glyma08g18110 Glyma03g37310 Glyma09g32120 Glyma06g14200 Glyma08g03540 Glyma09g33630 Glyma20g21440 Glyma12g02590 Glyma19g44540 Glyma08g24770 Glyma18g49240 Glyma13g22650 Glyma17g13390 Glyma16g33710 Glyma19g29000 Glyma07g38580 Glyma16g03960 Glyma06g03150 Glyma02g01970 Glyma13g01870 Glyma08g03880 Glyma11g33720 Glyma14g05850 Glyma08g45520 Glyma13g40030 Glyma10g04150 Glyma03g38120 Glyma11g34500 Glyma07g00900 Glyma12g34610 Glyma19g03040 Glyma09g02600 Glyma06g08990 Glyma16g04230 Glyma08g05490 Glyma05g04440 Glyma13g31390 Glyma03g25650 Glyma08g08170 Glyma18g44300 Glyma19g38940 Glyma05g08670 Glyma04g40810 Glyma19g38140 Glyma13g24420 Glyma06g00710 Glyma11g04620 Glyma09g24130 Glyma08g10140 Glyma02g03280 Glyma08g05820 Glyma08g48030 Glyma11g13620 Glyma06g05320 Glyma06g14370 Glyma06g20540 Glyma16g32280 Glyma07g05140 Glyma07g09710 Glyma11g14120 Glyma20g01420 Glyma15g15600 Glyma05g25920 Glyma10g42680 Glyma13g08490 Glyma12g36360 Glyma07g16940 Glyma08g15960 Glyma01g45280 Glyma10g05480 Glyma20g31820 Glyma10g28610 Glyma14g06650 Glyma17g03350 Glyma18g48910 Glyma03g04920 Glyma17g34870 Glyma05g02690 Glyma12g04020 Glyma16g01020 Glyma10g33650 Glyma15g26370 Glyma13g22350 Glyma13g38470 Glyma20g30450 Glyma11g05470 Glyma05g08950 Glyma11g00260 Glyma05g37730 Glyma09g05150 Glyma20g31040 Glyma06g43710 Glyma17g08550 Glyma19g38370 Glyma17g08930 Glyma18g53990 Glyma18g02870 Glyma12g02790 Glyma02g47210 Glyma15g14790 Glyma20g04130 Glyma09g05290 Glyma11g09190 Glyma06g29660 Glyma19g31770 Glyma11g13270 Glyma18g47360 Glyma20g35990 Glyma19g40960 Glyma01g05340 Glyma02g12930 Glyma16g04410 Glyma05g00620 Glyma10g05210 Glyma08g37400 Glyma08g07150 Glyma02g09210 Glyma17g07830 Glyma18g50260 Glyma13g19500 Glyma11g12260 Glyma02g03530 Glyma13g26170 Glyma19g44360 Glyma19g28760 Glyma17g13530 Glyma12g04090 Glyma16g28080 Glyma19g45260 Glyma12g30660 Glyma17g23860 Glyma09g07100 Glyma06g03050 Glyma14g05650 Glyma17g35230 Glyma10g36300 Glyma04g09500 Glyma04g08370 Glyma06g44430 Glyma04g09350 Glyma01g31750 Glyma15g13100 Glyma17g14260 Glyma04g00450 Glyma09g37920 Glyma01g01310 Glyma13g29780 Glyma10g44370 Glyma11g29920 Glyma08g04400 Glyma07g15320 Glyma03g32970 Glyma15g27740 Glyma17g14180 Glyma03g14900 Glyma11g13520 Glyma11g13520 Glyma15g42430 Glyma07g02170 Glyma16g02550 Glyma20g34500 Glyma09g32560 Glyma10g07550 Glyma15g18710 Glyma02g29980 Glyma03g24510 Glyma09g09690 Glyma14g25510 Glyma11g23820 Glyma01g06300 Glyma13g42330 Glyma02g06120 Glyma05g30190 Glyma01g01050 Glyma13g09880 Glyma12g35170 Glyma12g07100 Glyma08g29070 Glyma14g02260 Glyma02g29550 Glyma10g27170 Glyma13g11930 Glyma12g20160 Glyma10g00870 Glyma10g33360 Glyma04g12180 Glyma04g12180 Glyma10g08850 Glyma20g36920 Glyma04g01930 Glyma10g15860 Glyma12g00340 Glyma01g45020 Glyma04g33670 Glyma18g14830 Glyma16g29380 Glyma11g14500 Glyma16g09840 Glyma09g39920 Glyma07g34870 Glyma17g04590 Glyma09g07870 Glyma06g13810 Glyma17g38110 Glyma15g06280 Glyma06g43040 Glyma08g13210 Glyma08g23190 Glyma09g03660 Glyma05g27210 Glyma16g17440 Glyma13g29580 Glyma07g11860 Glyma17g37210 Glyma10g38490 Glyma17g37780 Glyma18g07390 Glyma18g07390 Glyma18g06460 Glyma18g06460 Glyma08g12730 Glyma02g21820 Glyma10g11840 Glyma13g02450 Glyma12g16050 Glyma18g00230 Glyma11g18270 Glyma10g35970 Glyma06g36260 Glyma15g35180 Glyma15g08360 Glyma17g08150 Glyma03g33050 Glyma18g49240 Glyma08g38480 Glyma04g41310 Glyma04g40220 Glyma05g38180 Glyma14g06980 Glyma04g34150 Glyma16g33650 Glyma08g38070 Glyma17g25180 Glyma06g01380 Glyma16g02180 Glyma03g33360 Glyma19g26830 Glyma02g47170 Glyma15g03860 Glyma18g52570 Glyma10g04760 Glyma20g04200 Glyma19g40340 Glyma12g35010 Glyma12g32670 Glyma10g06560 Glyma07g31270 Glyma03g39080 Glyma12g33360 Glyma12g33360 Glyma20g28120 Glyma17g12070 Glyma11g31270 Glyma01g36720 Glyma19g29800 Glyma10g32080 Glyma13g00370 Glyma18g52560 Glyma16g17380 Glyma16g17380 Glyma04g06900 Glyma08g21940 Glyma06g12860 Glyma06g12860 Glyma08g07400 Glyma20g04220 Glyma15g12090 Glyma13g21500 Glyma11g02600 Glyma12g31710 Glyma02g09640 Glyma02g09640 Glyma18g44270 Glyma11g05350 Glyma16g03960 Glyma18g32850 Glyma18g06850 Glyma18g06850 Glyma04g01300 Glyma04g02770 Glyma13g32680 Glyma09g24540 Glyma01g14780 Glyma10g32480 Glyma15g13060 Glyma19g04080 Glyma18g01900 Glyma06g07460 Glyma20g35890 Glyma09g31630 Glyma01g28970 Glyma06g38550 Glyma02g43520 Glyma02g43520 Glyma11g07810 Glyma03g28730 Glyma20g00700 Glyma15g17310 Glyma20g00370 Glyma20g22150 Glyma11g13830 Glyma09g33590 Glyma20g02980 Glyma08g21910 Glyma08g21910 Glyma06g13730 Glyma10g43160 Glyma01g02730 Glyma15g14250 Glyma02g00320 Glyma01g23340 Glyma05g33730 Glyma18g11130 Glyma1533s00200 Glyma1533s00200 Glyma05g25190 Glyma05g34640 Glyma13g01870 Glyma15g18040 Glyma18g42800 Glyma08g28180 Glyma11g13710 Glyma20g08210 Glyma02g28960 Glyma12g22950 Glyma09g28180 Glyma12g13580 Glyma01g03960 Glyma17g00630 Glyma14g02950 Glyma08g25620 Glyma02g31550 Glyma01g31690 Glyma01g08650 Glyma14g28720 Glyma20g28180 Glyma10g34340 Glyma20g33860 Glyma20g33860 Glyma06g21880 Glyma17g33070 Glyma18g45910 Glyma18g53940 Glyma20g30820 Glyma19g39510 Glyma19g39510 Glyma13g07870 Glyma18g45120 Glyma20g25150 Glyma08g17520 Glyma04g42620 Glyma05g09930 Glyma20g01570 Glyma01g26330 Glyma04g02990 Glyma20g04470 Glyma04g13660 Glyma04g13660 Glyma10g35070 Glyma07g05470 Glyma17g34710 Glyma06g43170 Glyma10g23820 Glyma08g30150 Glyma08g30150 Glyma11g03450 Glyma12g03100 Glyma02g06590 Glyma02g06590 Glyma11g30470 Glyma10g04150 Glyma09g21670 Glyma05g21240 Glyma03g38120 Glyma03g35590 Glyma20g02730 Glyma14g09130 Glyma11g11300 Glyma05g32650 Glyma01g36940 Glyma05g02730 Glyma05g04220 Glyma05g18820 Glyma08g13970 Glyma08g13970 Glyma18g39450 Glyma04g35200 Glyma16g27770 Glyma09g35810 Glyma10g05030 Glyma18g46380 Glyma14g27340 Glyma01g26900 Glyma03g37180 Glyma03g37180 Glyma03g14440 Glyma14g35790 Glyma13g38080 Glyma05g08120 Glyma05g08300 Glyma03g07220 Glyma10g28000 Glyma01g23850 Glyma16g32860 Glyma16g26580 Glyma03g06970 Glyma08g27900 Glyma02g08730 Glyma15g16030 Glyma03g34280 Glyma01g29110 Glyma20g26450 Glyma06g36010 Glyma04g10660 Glyma04g10660 Glyma01g24820 Glyma03g02630 Glyma08g23370 Glyma06g35680 Glyma04g33520 Glyma06g12490 Glyma02g16580 Glyma20g30390 Glyma10g00930 Glyma10g39330 Glyma18g08220 Glyma14g22870 Glyma12g07900 Glyma16g13440 Glyma14g13240 Glyma04g10620 Glyma04g03960 Glyma01g28070 Glyma08g45070 Glyma18g18250 Glyma08g37530 Glyma08g00550 Glyma18g50470 Glyma08g21970 Glyma08g37550 Glyma19g33170 Glyma10g12070 Glyma15g30620 Glyma07g09600 Glyma08g39250 Glyma10g10900 Glyma16g08130 Glyma16g17260 Glyma16g04680 Glyma03g03150 Glyma05g36630 Glyma02g43750 Glyma02g39700 Glyma14g10660 Glyma14g10660 Glyma07g35440 Glyma02g04590 Glyma09g38830 Glyma12g05030 Glyma14g32180 Glyma14g15210 Glyma14g35910 Glyma03g41910 Glyma14g07960 Glyma06g36350 Glyma08g44520 Glyma07g26890 Glyma01g05820 Glyma09g29720 Glyma06g45730 Glyma16g08380 Glyma12g35100 Glyma16g17180 Glyma19g40440 Glyma02g15210 Glyma03g31110 Glyma13g44350 Glyma10g12780 Glyma01g31800 Glyma11g02250 Glyma20g08930 Glyma01g39550 Glyma07g06400 Glyma20g31240 Glyma14g02960 Glyma02g33880 Glyma03g16460 Glyma03g37560 Glyma02g18610 Glyma19g42790 Glyma08g22550 Glyma02g28860 Glyma04g03510 Glyma18g18200 Glyma16g28560 Glyma12g07300 Glyma17g31380 Glyma09g29930 Glyma20g21990 Glyma04g33880 Glyma04g06430 Glyma04g06430 Glyma04g06430 Glyma01g37660 Glyma10g35580 Glyma17g17670 Glyma17g23760 Glyma18g38060 Glyma15g20510 Glyma15g21370 Glyma15g21370 Glyma01g35110 Glyma09g34130 Glyma03g22090 Glyma03g22090 Glyma08g29500 Glyma19g11050 Glyma03g42390 Glyma03g42390 Glyma09g06520 Glyma11g16350 Glyma18g36390 Glyma03g17040 Glyma20g17680 Glyma20g28960 Glyma18g03100 Glyma07g04050 Glyma14g24300 Glyma06g02850 Glyma18g43600 Glyma08g23120 Glyma09g10190 Glyma06g42010 Glyma17g04080 Glyma06g05660 Glyma14g14310 Glyma14g14310 Glyma11g36660 Glyma08g06130 Glyma06g01660 Glyma01g04450 Glyma07g39920 Glyma18g33430 Glyma10g31510 Glyma05g11190 Glyma01g38960 Glyma18g38230 Glyma01g06410 Glyma10g34910 Glyma09g25110 Glyma13g28110 Glyma08g16610 Glyma18g28360 Glyma13g18460 Glyma17g28950 Glyma14g34630 Glyma13g19140 Glyma06g43350 Glyma19g24520 Glyma10g02930 Glyma03g02520 Glyma16g17170 Glyma06g07420 Glyma07g31570 Glyma04g19020 Glyma05g07600 Glyma12g22310 Glyma17g16410 Glyma06g00710 Glyma01g31700 Glyma20g04810 Glyma08g26050 Glyma13g23730 Glyma17g22120 Glyma02g07930 Glyma10g33090 Glyma18g48100 Glyma04g06290 Glyma04g06290 Glyma15g03500 Glyma06g10110 Glyma14g07820 Glyma15g00780 Glyma15g00780 Glyma10g39690 Glyma15g08090 Glyma15g08090 Glyma16g04020 Glyma16g04020 Glyma13g18040 Glyma08g01630 Glyma18g47050 Glyma14g38960 Glyma12g34420 Glyma06g05420 Glyma08g01440 Glyma03g30380 Glyma08g48030 Glyma16g13400 Glyma16g17010 Glyma17g10040 Glyma03g03620 Glyma03g03580 Glyma10g03990 Glyma06g05680 Glyma10g06700 Glyma20g33050 Glyma20g08250 Glyma20g08250 Glyma09g27820 Glyma13g30620 Glyma03g12100 Glyma12g06960 Glyma19g29860 Glyma14g17210 Glyma13g19980 Glyma14g16060 Glyma09g34090 Glyma16g30340 Glyma12g10700 Glyma20g05440 Glyma06g43510 Glyma03g30460 Glyma02g29530 Glyma09g33830 Glyma05g01570 Glyma09g29870 Glyma05g03610 Glyma08g01230 Glyma14g18450 Glyma17g22370 Glyma08g09690 Glyma08g09690 Glyma18g20850 Glyma12g23220 Glyma14g22240 Glyma06g05320 Glyma07g07360 Glyma02g01830 Glyma11g05030 Glyma13g23160 Glyma13g23160 Glyma20g37660 Glyma11g09990 Glyma04g11300 Glyma14g33820 Glyma11g31510 Glyma13g34670 Glyma02g19430 Glyma13g26280 Glyma06g00860 Glyma11g12550 Glyma07g06470 Glyma08g06090 Glyma08g06090 Glyma13g17620 Glyma06g20540 Glyma19g33060 Glyma06g16220 Glyma05g27840 Glyma17g07850 Glyma05g35120 Glyma16g02650 Glyma05g38140 Glyma15g05050 Glyma01g24780 Glyma02g39840 Glyma18g05470 Glyma18g05470 Glyma15g08670 Glyma09g31850 Glyma08g39520 Glyma18g15240 Glyma01g44180 Glyma13g04940 Glyma03g35150 Glyma19g27430 Glyma12g03130 Glyma02g41650 Glyma16g24720 Glyma20g22970 Glyma16g08940 Glyma10g32520 Glyma17g14150 Glyma17g14150 Glyma07g37050 Glyma07g37050 Glyma07g14480 Glyma06g39440 Glyma13g36910 Glyma07g02790 Glyma01g42880 Glyma20g21190 Glyma13g27430 Glyma05g29720 Glyma11g06190 Glyma17g11900 Glyma08g09020 Glyma18g13180 Glyma07g09710 Glyma05g30290 Glyma17g32730 Glyma06g17600 Glyma02g17030 Glyma08g19270 Glyma16g34510 Glyma16g34510 Glyma17g19950 Glyma20g01420 Glyma16g04530 Glyma18g07360 Glyma18g37680 Glyma17g35060 Glyma20g30350 Glyma03g35970 Glyma13g35390 Glyma0893s00200 Glyma07g10050 Glyma12g13510 Glyma01g07610 Glyma10g25790 Glyma19g29660 Glyma18g01100 Glyma18g01100 Glyma05g08780 Glyma18g07160 Glyma12g29740 Glyma09g05210 Glyma14g39470 Glyma05g34030 Glyma20g04190 Glyma06g17990 Glyma14g04220 Glyma12g32070 Glyma02g00750 Glyma13g33950 Glyma03g39680 Glyma18g08260 Glyma16g33950 Glyma09g27750 Glyma09g27750 Glyma07g34740 Glyma20g15460 Glyma10g31770 Glyma07g01960 Glyma15g39750 Glyma15g39750 Glyma06g37200 Glyma07g13550 Glyma20g03690 Glyma10g00280 Glyma14g02220 Glyma12g07150 Glyma10g04710 Glyma04g00800 Glyma04g00800 Glyma03g42460 Glyma03g42460 Glyma05g08810 Glyma05g03620 Glyma13g40850 Glyma12g33560 Glyma02g41710 Glyma13g09390 Glyma20g36610 Glyma09g37300 Glyma09g37300 Glyma08g11620 Glyma11g09500 Glyma17g13370 Glyma02g05120 Glyma11g12990 Glyma02g05300 Glyma12g36360 Glyma12g36360 Glyma13g21210 Glyma08g47070 Glyma07g37800 Glyma10g00420 Glyma03g28950 Glyma10g34810 Glyma10g34810 Glyma19g43500 Glyma13g34310 Glyma11g16310 Glyma10g10020 Glyma14g39220 Glyma19g06450 Glyma09g30580 Glyma19g28060 Glyma06g06510 Glyma18g02140 Glyma01g44640 Glyma02g03630 Glyma02g03630 Glyma16g08020 Glyma04g14230 Glyma14g04240 Glyma09g12040 Glyma13g44550 Glyma07g05280 Glyma15g13890 Glyma05g35330 Glyma08g26490 Glyma08g26490 Glyma08g08830 Glyma14g01000 Glyma03g30890 Glyma08g19370 Glyma01g09630 Glyma11g00730 Glyma12g12290 Glyma12g12290 Glyma0022s00220 Glyma16g08600 Glyma15g24680 Glyma08g40640 Glyma15g10930 Glyma19g00300 Glyma04g05000 Glyma16g14710 Glyma16g05310 Glyma05g30120 Glyma11g14790 Glyma19g07140 Glyma18g47310 Glyma20g32990 Glyma07g14080 Glyma20g16140 Glyma11g02830 Glyma06g01980 Glyma13g32070 Glyma14g35260 Glyma07g13040 Glyma02g03030 Glyma12g34490 Glyma05g14180 Glyma20g23650 Glyma07g22980 Glyma19g12430 Glyma19g12430 Glyma15g12900 Glyma15g14470 Glyma06g46670 Glyma13g31360 Glyma09g26290 Glyma13g09370 Glyma14g04180 Glyma12g19000 Glyma09g29950 Glyma09g05120 Glyma15g15120 Glyma15g16510 Glyma19g29850 Glyma13g11130 Glyma07g17180 Glyma17g18690 Glyma16g01020 Glyma20g35930 Glyma09g02470 Glyma09g02470 Glyma18g09780 Glyma03g40420 Glyma14g07090 Glyma18g40070 Glyma17g13920 Glyma20g07760 Glyma15g26370 Glyma15g26370 Glyma15g04240 Glyma16g02640 Glyma16g02640 Glyma02g02490 Glyma15g13240 Glyma19g36530 Glyma13g25500 Glyma04g33920 Glyma19g27980 Glyma01g36330 Glyma19g37640 Glyma11g21070 Glyma01g34640 Glyma09g04890 Glyma06g42610 Glyma08g09080 Glyma13g01380 Glyma13g01380 Glyma09g37890 Glyma09g37890 Glyma01g44310 Glyma05g03590 Glyma06g24140 Glyma01g33460 Glyma02g17120 Glyma17g00950 Glyma17g35380 Glyma03g23960 Glyma12g25450 Glyma17g29950 Glyma08g22340 Glyma01g43430 Glyma06g14120 Glyma14g40220 Glyma17g33820 Glyma11g00910 Glyma20g27760 Glyma08g19850 Glyma17g11570 Glyma09g41710 Glyma09g41710 Glyma06g03810 Glyma19g39890 Glyma18g51600 Glyma19g02650 Glyma18g47450 Glyma18g47450 Glyma03g29510 Glyma06g04520 Glyma10g37310 Glyma03g33560 Glyma06g42280 Glyma15g15070 Glyma15g15070 Glyma15g15070 Glyma04g41630 Glyma04g41630 Glyma09g35770 Glyma09g35770 Glyma17g25680 Glyma16g23380 Glyma16g23380 Glyma12g35610 Glyma08g33830 Glyma08g34720 Glyma10g36790 Glyma16g10770 Glyma18g16870 Glyma18g16870 Glyma01g10890 Glyma02g06860 Glyma07g38500 Glyma17g30480 Glyma14g20350 Glyma04g09250 Glyma08g41000 Glyma17g17130 Glyma19g32800 Glyma19g32800 Glyma16g01760 Glyma08g23410 Glyma09g02210 Glyma04g34070 Glyma13g07690 Glyma15g35880 Glyma17g37040 Glyma14g18340 Glyma11g07620 Glyma17g17700 Glyma02g44670 Glyma20g17430 Glyma15g22750 Glyma04g11760 Glyma01g38270 Glyma08g38310 Glyma15g10120 Glyma19g36860 Glyma08g04500 Glyma13g17870 Glyma14g13160 Glyma14g33120 Glyma13g09010 Glyma07g01280 Glyma18g53990 Glyma07g15770 Glyma08g04060 Glyma16g04820 Glyma13g25780 Glyma06g00220 Glyma07g37560 Glyma07g37560 Glyma08g42310 Glyma17g01480 Glyma04g12650 Glyma03g35550 Glyma02g38120 Glyma13g37350 Glyma16g04580 Glyma05g31800 Glyma13g22270 Glyma12g17720 Glyma12g22970 Glyma03g03210 Glyma16g25570 Glyma08g07310 Glyma15g40660 Glyma17g34680 Glyma12g12100 Glyma12g02790 Glyma15g25440 Glyma03g38330 Glyma03g38330 Glyma18g14850 Glyma04g07480 Glyma04g07480 Glyma05g35840 Glyma06g23960 Glyma13g19860 Glyma12g22780 Glyma17g37080 Glyma04g15180 Glyma19g33410 Glyma06g03190 Glyma06g03190 Glyma06g43020 Glyma06g43020 Glyma05g32390 Glyma06g17810 Glyma08g19170 Glyma02g45560 Glyma12g01440 Glyma06g33890 Glyma02g41440 Glyma20g26350 Glyma02g01660 Glyma10g03230 Glyma11g32950 Glyma06g16740 Glyma17g20530 Glyma10g26650 Glyma12g31550 Glyma14g12940 Glyma02g07130 Glyma09g30960 Glyma16g01390 Glyma09g25570 Glyma09g25570 Glyma03g26230 Glyma01g22760 Glyma01g06190 Glyma01g06190 Glyma01g37290 Glyma06g15200 Glyma19g06780 Glyma17g03130 Glyma19g37820 Glyma04g15230 Glyma08g18540 Glyma03g04640 Glyma03g36100 Glyma18g36440 Glyma13g11990 Glyma13g11990 Glyma15g05780 Glyma11g11240 Glyma08g28940 Glyma13g17170 Glyma01g01350 Glyma17g03920 Glyma11g30030 Glyma15g10530 Glyma16g13330 Glyma11g08560 Glyma10g11580 Glyma18g26140 Glyma11g13550 Glyma08g35500 Glyma12g34210 Glyma05g01700 Glyma11g11410 Glyma07g35620 Glyma17g29570 Glyma12g10450 Glyma13g37640 Glyma08g17120 Glyma15g05570 Glyma14g10880

Genes with sequence AAAGAT

Module1

Glyma04g40000

Glyma05g00740

Glyma05g01360

Glyma05g36710

Glyma06g14640

Glyma08g18080

Glyma09g37920

Glyma10g30220

Glyma10g36680

Glyma11g18290

Glyma13g44870

Glyma18g10200

Glyma19g44540

Glyma20g33120

Module2

Glyma03g19260

Glyma04g40170

Glyma06g05530

Glyma06g23590

Glyma07g07420

Glyma07g09700

Glyma08g00320

Glyma09g04340

Glyma10g28610

Glyma15g30110

Glyma17g12150

Glyma19g25980

Glyma19g29590

Glyma20g28350

Module3

Glyma08g46610

Glyma08g48240

Glyma15g35070

Glyma15g39090

Glyma17g11170

Module4

Glyma01g07930

Glyma04g12480

Glyma08g06470

Glyma09g06250

Glyma09g28490

Glyma11g37360

Glyma12g33530

Glyma13g19500

Glyma13g22650

Glyma16g28590

Module5

Glyma01g03180

Glyma03g37340

Glyma04g00660

Glyma04g09820

Glyma04g40430

Glyma05g21820

Glyma05g30380

Glyma06g34190

Glyma07g00330

Glyma07g39020

Glyma08g06010

Glyma08g44590

Glyma08g47500

Glyma09g04630

Glyma10g40150

Glyma11g29350

Glyma14g07150

Glyma17g17850

Glyma20g28780

Module6

Glyma01g01310

Glyma02g00340

Glyma02g45690

Glyma03g28850

Glyma03g31530

Glyma03g32830

Glyma04g00450

Glyma05g00620

Glyma08g15960

Glyma08g45610

Glyma14g09510

Glyma14g20450

Glyma17g11940

Glyma20g30910

Module7

Glyma01g02950

Glyma01g45390

Glyma03g16510

Glyma03g36000

Glyma03g40860

Glyma04g09500

Glyma04g37270

Glyma05g37900

Glyma06g03100

Glyma06g04980

Glyma06g06420

Glyma07g03230

Glyma07g05230

Glyma08g03540

Glyma09g05810

Glyma09g12320

Glyma09g29960

Glyma10g30440

Glyma10g42910

Glyma11g05900

Glyma11g18980

Glyma13g05120

Glyma13g19830

Glyma15g12240

Glyma18g32830

Glyma19g38600

Glyma20g00910

Glyma20g28860

Glyma20g29190

Module8

Glyma04g00710

Glyma06g07780

Glyma08g45600

Glyma09g32120

Glyma15g26370

Module9

Glyma01g42800

Glyma02g37340

Glyma02g43190

Glyma03g34440

Glyma04g02230

Glyma05g25920

Glyma05g33010

Glyma06g11610

Glyma06g19870

Glyma06g43630

Glyma07g04940

Glyma07g16810

Glyma07g32590

Glyma07g37270

Glyma08g08170

Glyma08g17270

Glyma08g24720

Glyma08g37670

Glyma09g32630

Glyma09g36620

Glyma10g02090

Glyma10g31280

Glyma11g03310

Glyma11g34380

Glyma11g34490

Glyma12g02240

Glyma13g16870

Glyma13g22350

Glyma13g32310

Glyma15g15600

Glyma15g15620

Glyma15g35410

Glyma16g33790

Glyma17g02080

Glyma17g07440

Glyma17g15690

Glyma18g02090

Glyma18g43040

Glyma18g44030

Glyma18g48910

Glyma19g29000

Glyma19g45260

Glyma20g24710

Glyma20g38590

Module10

Glyma07g32330

Glyma07g32340

Glyma07g38110

Glyma08g47750

Glyma12g06100

Glyma12g29510

Module11

Glyma01g04070

Glyma01g27900

Glyma06g02380

Glyma12g02410

Glyma15g14330

Glyma19g44360

Module12

Glyma02g45940

Glyma04g33010

Glyma06g03200

Glyma06g08990

Glyma07g30810

Glyma10g07710

Glyma12g11210

Glyma15g24130

Glyma19g36800

Glyma19g38940

Module13

Glyma01g42370

Glyma02g17060

Glyma08g37400

Glyma11g06740

Glyma12g34510

Glyma15g35390

Glyma16g04410

Glyma18g50260

Glyma20g01420

Module14

Glyma01g32130

Glyma01g43280

Glyma03g18410

Glyma05g28810

Glyma07g34300

Glyma13g34340

Glyma16g34770

Glyma16g34840

Glyma18g05710

Glyma18g50310

Glyma20g22290

Module15

Glyma01g03470

Glyma08g12650

Glyma09g28750

Glyma13g28970

Glyma17g07830

Glyma17g35230

Glyma18g53450

Glyma19g38800

Module16

Glyma01g38040

Glyma08g24680

Glyma09g37470

Glyma10g34060

Glyma13g23770

Glyma16g06530

Glyma18g06230

Glyma20g34830

Module17

Glyma06g19820

Glyma07g00900

Glyma08g18110

Glyma12g34550

Glyma13g40100

Glyma16g04190

Glyma17g34870

Glyma19g29180

Glyma20g27950

Module18

Glyma13g27130

Glyma17g16620

Module19

Glyma03g22930

Glyma03g24020

Glyma04g02560

Glyma04g38830

Glyma05g32920

Glyma07g04520

Glyma09g03400

Glyma09g11990

Glyma11g07750

Glyma11g16050

Glyma12g04460

Glyma12g06410

Glyma13g43920

Glyma14g06650

Glyma16g06410

Glyma16g24890

Glyma20g22700

Glyma0092s00200

Module20

Glyma02g06730

Glyma03g28080

Glyma07g35000

Glyma08g17200

Glyma10g07500

Glyma19g11560

Module21

Glyma03g03190

Glyma03g38120

Glyma04g35600

Glyma05g37690

Glyma06g14220

Glyma07g07970

Glyma07g29670

Glyma07g39540

Glyma08g41960

Glyma08g47310

Glyma09g02040

Glyma11g00260

Glyma12g13320

Glyma12g29630

Glyma12g30620

Glyma12g30660

Glyma13g07900

Glyma13g26600

Glyma13g28390

Glyma13g40180

Glyma14g40170

Glyma16g04560

Glyma16g08360

Glyma16g24140

Glyma18g47360

Glyma20g35990

Glyma20g37370

Module22

Glyma01g26840

Glyma03g04960

Glyma06g47730

Glyma15g23830

Module23

Glyma02g37080

Glyma02g40290

Glyma02g47210

Glyma03g36560

Glyma05g24110

Glyma05g36420

Glyma06g03150

Glyma08g03150

Glyma08g09200

Glyma08g25950

Glyma09g01320

Glyma10g31590

Glyma10g35520

Glyma12g08040

Glyma13g06390

Glyma14g06630

Glyma15g12170

Glyma16g24120

Glyma19g32990

Glyma19g44160

Module24

Glyma04g41820

Glyma08g13180

Glyma08g21460

Glyma13g43990

Genes with sequence CTCTT

Module1

Glyma02g46330

Glyma03g08280

Glyma03g32940

Glyma04g40000

Glyma05g00740

Glyma05g08950

Glyma06g13850

Glyma06g14850

Glyma06g36590

Glyma08g01720

Glyma08g15430

Glyma08g18080

Glyma08g18310

Glyma08g22690

Glyma11g13670

Glyma11g18290

Glyma11g25650

Glyma13g17570

Glyma13g24340

Glyma13g31620

Glyma13g40030

Glyma14g05850

Glyma15g01510

Glyma15g10490

Glyma18g10200

Glyma18g14640

Glyma18g47930

Glyma18g50180

Glyma19g44540

Module2

Glyma02g07960

Glyma02g15150

Glyma04g40170

Glyma06g05530

Glyma06g23590

Glyma07g07420

Glyma08g00320

Glyma08g04400

Glyma08g11960

Glyma08g18880

Glyma08g27560

Glyma09g04340

Glyma09g04530

Glyma09g37910

Glyma09g40090

Glyma14g17730

Glyma15g03460

Glyma15g06000

Glyma15g41970

Glyma16g01960

Glyma17g12150

Glyma19g01940

Glyma19g29590

Glyma20g00760

Module3

Glyma03g02580

Glyma03g03870

Glyma05g33470

Glyma06g08630

Glyma08g46610

Glyma13g26070

Glyma15g35070

Glyma17g11170

Glyma17g13500

Glyma17g13530

Glyma19g28770

Glyma19g37620

Glyma20g31040

Module4

Glyma02g08950

Glyma02g11640

Glyma07g09730

Glyma07g16910

Glyma09g06250

Glyma09g28490

Glyma09g32080

Glyma12g33530

Glyma13g22650

Glyma13g24200

Glyma16g20780

Glyma17g03910

Glyma18g49240

Glyma18g50760

Glyma19g40810

Glyma20g38140

Module5

Glyma01g03180

Glyma03g37340

Glyma03g40760

Glyma04g00660

Glyma04g03110

Glyma05g27190

Glyma07g15320

Glyma07g39020

Glyma08g06420

Glyma08g47790

Glyma09g04630

Glyma10g40150

Glyma11g19490

Glyma11g33720

Glyma12g04020

Glyma13g41930

Glyma14g07150

Glyma16g28080

Glyma18g04500

Glyma19g06460

Glyma20g29660

Glyma20g32000

Glyma20g38440

Module6

Glyma01g01310

Glyma01g38650

Glyma02g01990

Glyma02g45690

Glyma03g32830

Glyma05g00620

Glyma05g04290

Glyma06g46350

Glyma08g15960

Glyma08g43330

Glyma08g45610

Glyma10g35870

Glyma13g42340

Glyma15g38090

Glyma20g30910

Module7

Glyma01g45390

Glyma04g37270

Glyma04g40580

Glyma05g03850

Glyma06g03100

Glyma07g02460

Glyma08g01990

Glyma08g29130

Glyma09g00670

Glyma09g05810

Glyma09g12320

Glyma09g40980

Glyma10g06250

Glyma11g18980

Glyma11g21010

Glyma12g04090

Glyma13g00380

Glyma13g05120

Glyma13g39600

Glyma15g41960

Glyma16g29370

Glyma18g32830

Glyma19g30600

Glyma19g44780

Glyma20g00910

Glyma20g29190

Module8

Glyma18g53340

Glyma20g04130

Module9

Glyma01g34580

Glyma01g41930

Glyma01g42560

Glyma01g42830

Glyma02g09540

Glyma02g27090

Glyma02g36580

Glyma02g37340

Glyma02g43190

Glyma03g33340

Glyma03g34440

Glyma03g37390

Glyma04g00210

Glyma04g03200

Glyma04g42130

Glyma05g02830

Glyma05g25920

Glyma05g33010

Glyma05g37420

Glyma06g06930

Glyma06g07160

Glyma06g11610

Glyma06g12010

Glyma06g19870

Glyma06g42040

Glyma06g43560

Glyma06g43630

Glyma06g47690

Glyma07g16810

Glyma07g16940

Glyma07g17170

Glyma07g34010

Glyma07g37270

Glyma07g38620

Glyma07g39710

Glyma08g08170

Glyma08g37670

Glyma08g43040

Glyma09g00800

Glyma09g05150

Glyma09g07770

Glyma09g32630

Glyma09g33510

Glyma09g40580

Glyma09g40740

Glyma10g04150

Glyma10g05170

Glyma10g07410

Glyma10g10240

Glyma10g31280

Glyma10g32070

Glyma10g38110

Glyma11g03310

Glyma11g13270

Glyma11g13620

Glyma11g22090

Glyma11g29920

Glyma11g33620

Glyma11g34490

Glyma11g35560

Glyma12g03050

Glyma12g33750

Glyma13g16870

Glyma13g22350

Glyma13g32310

Glyma13g42280

Glyma14g38370

Glyma15g02380

Glyma15g07700

Glyma15g13100

Glyma15g15600

Glyma15g15620

Glyma15g42680

Glyma16g04980

Glyma16g22920

Glyma16g32300

Glyma17g03360

Glyma17g07440

Glyma17g15690

Glyma18g06220

Glyma18g06350

Glyma18g43040

Glyma18g44030

Glyma18g46500

Glyma18g50320

Glyma19g03670

Glyma19g29000

Glyma19g40960

Glyma20g28100

Glyma20g29210

Glyma20g30450

Glyma20g35570

Module10

Glyma03g26060

Glyma07g32330

Glyma07g32340

Glyma11g14140

Glyma12g29510

Glyma16g27900

Glyma16g33710

Glyma18g52250

Glyma20g35630

Module11

Glyma01g27900

Glyma02g11720

Glyma19g34370

Module12

Glyma01g04380

Glyma02g46600

Glyma05g20460

Glyma06g03200

Glyma06g08990

Glyma06g09220

Glyma06g42850

Glyma07g05140

Glyma07g16850

Glyma07g38580

Glyma10g07710

Glyma13g01120

Glyma15g24130

Glyma16g03960

Glyma19g36800

Glyma19g38940

Glyma19g42200

Glyma20g32140

Module13

Glyma01g05050

Glyma01g31660

Glyma01g38600

Glyma02g16800

Glyma03g05500

Glyma03g37400

Glyma04g42300

Glyma06g12510

Glyma06g47190

Glyma07g04340

Glyma08g37400

Glyma08g43550

Glyma09g00850

Glyma11g02350

Glyma11g03940

Glyma11g06740

Glyma11g34500

Glyma11g34510

Glyma12g34510

Glyma16g04410

Glyma17g16990

Glyma18g02870

Glyma18g50260

Glyma19g35270

Glyma20g31820

Module14

Glyma01g43280

Glyma05g00570

Glyma06g14370

Glyma08g02020

Glyma08g12610

Glyma08g40360

Glyma10g39040

Glyma11g19920

Glyma13g29780

Glyma13g31010

Glyma13g36740

Glyma15g08860

Glyma17g07270

Glyma18g19080

Glyma19g31770

Glyma19g38130

Glyma19g43390

Glyma20g01670

Glyma20g03840

Glyma20g35260

Module15

Glyma03g36140

Glyma07g00840

Glyma07g09710

Glyma07g35770

Glyma08g12650

Glyma08g48160

Glyma11g37620

Glyma12g10760

Glyma13g28970

Glyma15g01500

Glyma15g11040

Glyma17g07830

Glyma17g35230

Glyma18g53450

Module16

Glyma01g26750

Glyma01g38040

Glyma02g02430

Glyma02g40010

Glyma09g02190

Glyma09g02910

Glyma09g24130

Glyma13g23770

Glyma13g27300

Glyma16g01020

Glyma16g06500

Glyma16g06530

Glyma17g31900

Glyma17g34880

Glyma18g44010

Glyma20g08560

Module17

Glyma02g42220

Glyma04g01130

Glyma05g37730

Glyma06g19820

Glyma08g18110

Glyma11g03690

Glyma12g00390

Glyma13g40100

Glyma15g19580

Glyma17g23870

Glyma19g29180

Glyma19g35560

Glyma20g27940

Module18

Glyma02g40890

Glyma06g16810

Glyma12g04850

Glyma15g11700

Glyma16g26940

Glyma17g16620

Module19

Glyma02g09240

Glyma02g13910

Glyma03g24020

Glyma04g34160

Glyma04g40400

Glyma05g32920

Glyma06g34940

Glyma07g04520

Glyma07g14730

Glyma09g07010

Glyma09g11990

Glyma09g34420

Glyma11g16050

Glyma11g19430

Glyma11g36730

Glyma12g04460

Glyma12g06030

Glyma12g06410

Glyma13g21660

Glyma14g06640

Glyma14g06650

Glyma15g06220

Glyma15g30150

Glyma16g06410

Glyma16g25190

Glyma18g22780

Glyma20g29200

Glyma20g29930

Module20

Glyma01g04190

Glyma02g06730

Glyma02g13800

Glyma02g26160

Glyma02g35190

Glyma02g42250

Glyma03g22960

Glyma03g28080

Glyma04g06450

Glyma05g15700

Glyma07g35000

Glyma08g17200

Glyma08g19140

Glyma08g48030

Glyma10g07500

Glyma10g08860

Glyma10g11060

Glyma11g04620

Glyma13g38710

Glyma14g05840

Glyma15g06140

Glyma15g15610

Glyma16g06740

Glyma17g08550

Glyma17g14800

Glyma18g15530

Glyma18g47820

Glyma19g11560

Glyma19g44060

Module21

Glyma02g04760

Glyma02g47690

Glyma03g35000

Glyma03g38120

Glyma04g35600

Glyma04g38870

Glyma04g42160

Glyma05g37690

Glyma06g05460

Glyma06g05470

Glyma06g14200

Glyma06g44430

Glyma07g17250

Glyma07g29670

Glyma07g39540

Glyma08g07490

Glyma08g47310

Glyma09g02040

Glyma09g33630

Glyma10g30020

Glyma12g13320

Glyma12g30620

Glyma13g07900

Glyma13g26600

Glyma13g28330

Glyma16g01640

Glyma16g04420

Glyma16g08360

Glyma16g24140

Glyma18g52590

Glyma18g53990

Glyma20g32050

Module22

Glyma01g32750

Glyma01g39460

Glyma01g42670

Glyma06g02290

Glyma06g47730

Glyma08g24750

Glyma08g45520

Glyma09g05340

Glyma10g05800

Glyma13g20170

Glyma15g23830

Glyma17g03740

Glyma19g37240

Glyma19g43460

Module23

Glyma02g37080

Glyma02g40290

Glyma02g47210

Glyma04g06700

Glyma04g09350

Glyma05g01180

Glyma05g32100

Glyma05g34570

Glyma06g03050

Glyma06g11080

Glyma08g03150

Glyma08g09200

Glyma08g14130

Glyma08g25950

Glyma09g01320

Glyma10g06600

Glyma11g15680

Glyma12g08990

Glyma13g22940

Glyma14g00720

Glyma15g12170

Glyma17g09280

Glyma17g23860

Glyma20g30970

Glyma0169s00210

Module24

Glyma03g28640

Glyma04g41820

Glyma04g43090

Glyma05g30410

Glyma07g13840

Glyma08g13180

Glyma08g21460

Glyma09g28890

Glyma12g02590

Glyma13g08490

Glyma13g26170

Glyma13g32030

Glyma15g03130

Glyma17g03090

Glyma17g10720

Glyma17g18040

Glyma19g16460
