# Supplementary material for: Predicting gene regulatory networks of soybean nodulation from RNA-Seq transcriptome data
Source: BMC Bioinformatics. 2013 Sep 22;14:278. doi: 10.1186/1471-2105-14-278 (PMC3854569; doi:10.1186/1471-2105-14-278)
Supplement: Additional file 4 — Modules generated based on the 24-hour DEGs. [file 1471-2105-14-278-S4.doc]

**Supplemental Materials**

**The module 15,16,20,25,26,30,32 and 41 are predicted with GRAS family TFs. Among of them moduel 32 and 41 are with Leucine Zipper domain based on binding site analysis. The module 15 and 25 are with** bHLH-Zip domain**.**

**Module 1-49 are generated based on 24 hour DEGs with all included TF families.**

1


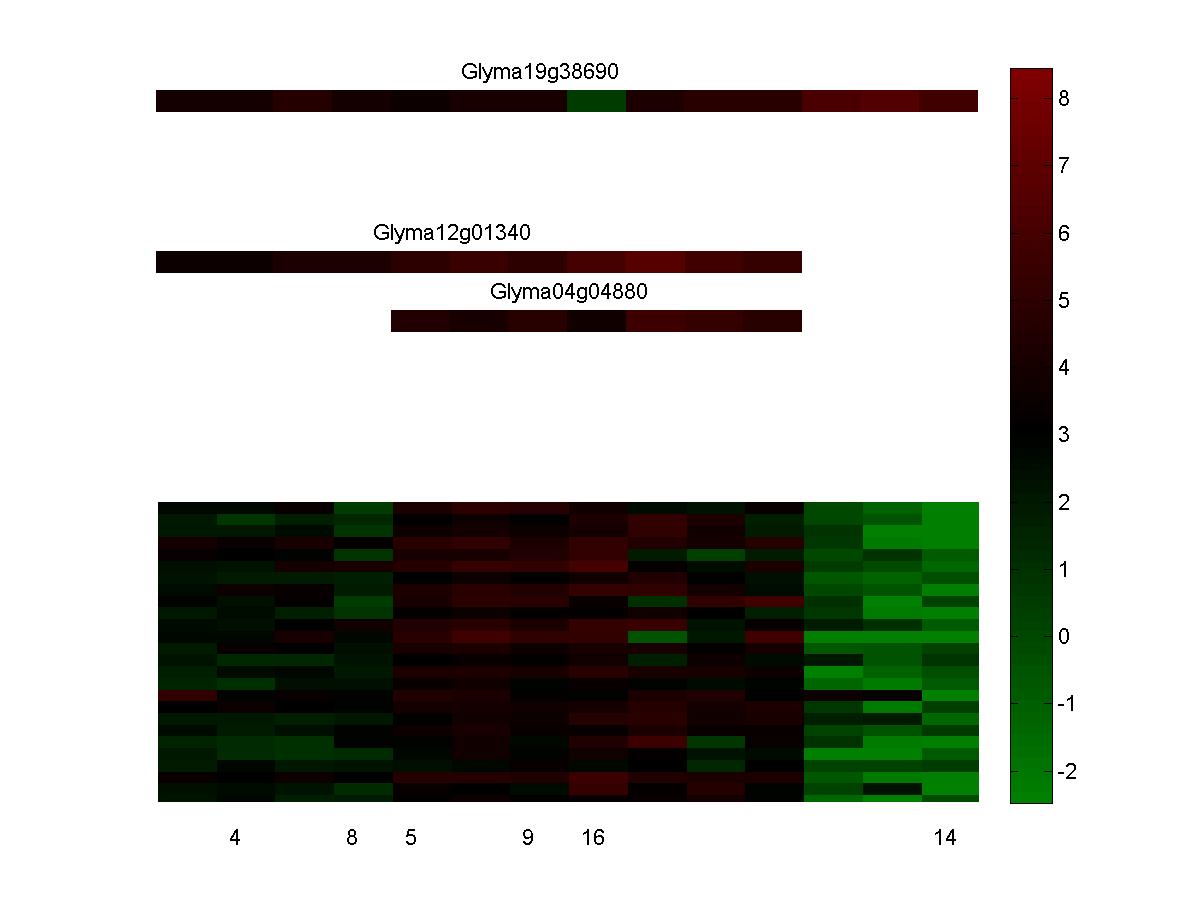


1 Glyma12g01340 C3H-type1(Zn)

1 Glyma19g38690 Homeodomain/HOMEOBOX

1 Glyma04g04880 LIM

Glyma10g07550 Glyma04g07270 Glyma17g03050 Glyma15g03710 Glyma07g16490

Glyma08g21630 Glyma17g13240 Glyma17g18260 Glyma12g04960 Glyma11g18370

Glyma11g11310 Glyma03g21540 Glyma07g35640 Glyma07g11390 Glyma08g44180

Glyma18g05500 Glyma02g11720 Glyma04g06410 Glyma20g31250 Glyma06g09340

Glyma12g03490 Glyma19g32610 Glyma11g24340 Glyma03g34760 Glyma19g03770

Glyma18g08560

2


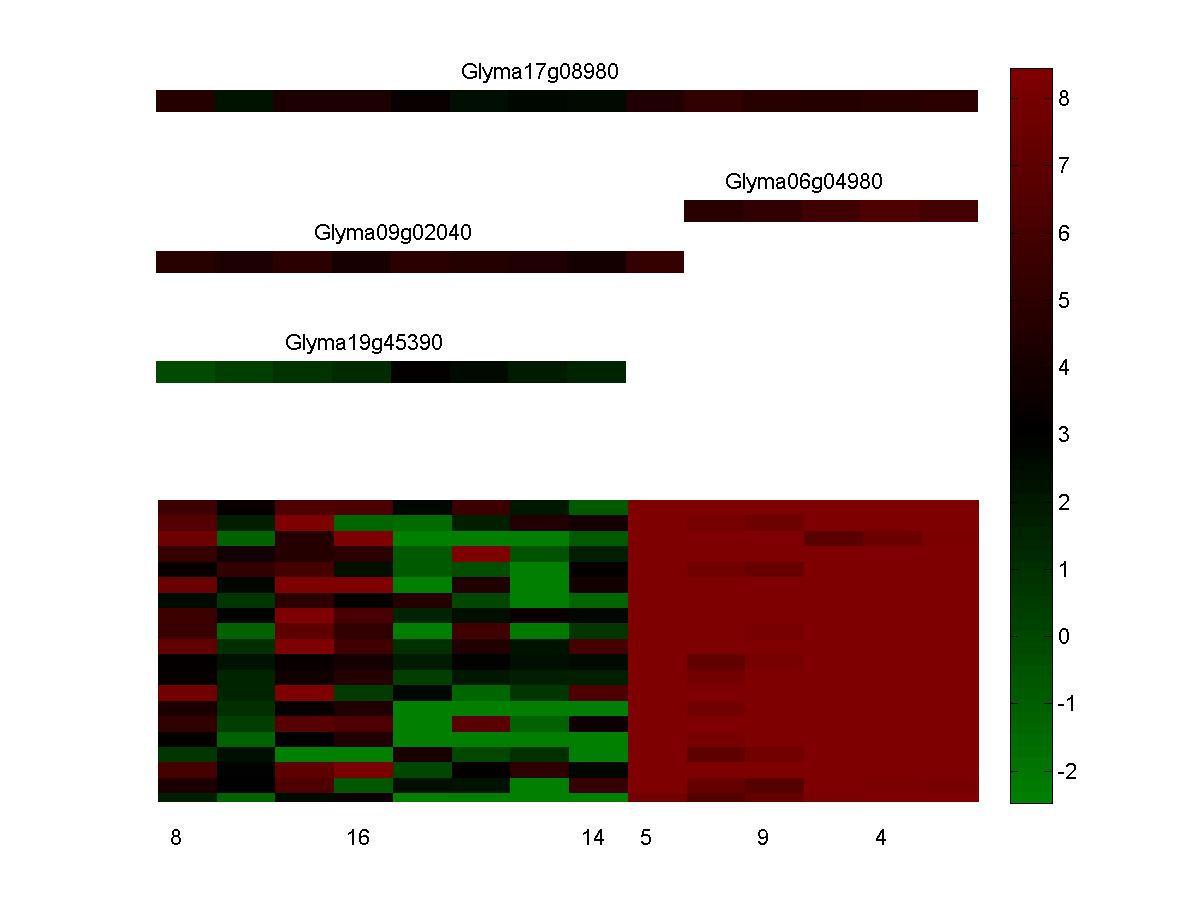


2 Glyma06g04980 LIM

2 Glyma09g02040 MYB/HD-like

2 Glyma17g08980 bHLH

2 Glyma19g45390 Homeodomain/HOMEOBOX

Glyma17g14620 Glyma01g31750 Glyma06g02290 Glyma20g34820 Glyma11g05800

Glyma16g27900 Glyma12g06110 Glyma04g08830 Glyma10g05800 Glyma11g18320

Glyma19g37230 Glyma19g37240 Glyma09g05440 Glyma09g05340 Glyma17g02600

Glyma17g14230 Glyma15g09540 Glyma08g38740 Glyma01g39460 Glyma08g17300

3


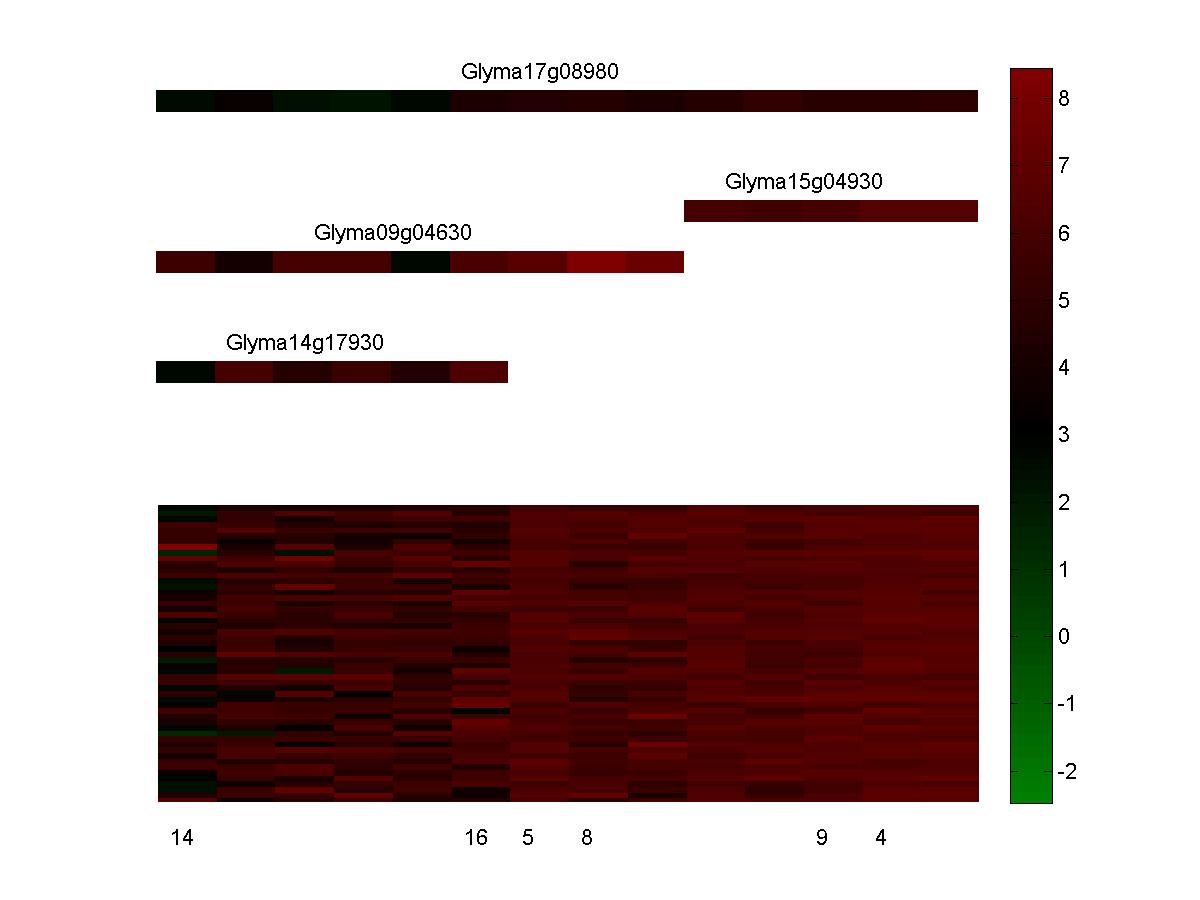


3 Glyma17g08980 bHLH

3 Glyma15g04930 AP2-EREBP

3 Glyma09g04630 AP2-EREBP

3 Glyma14g17930 CCHC (Zn)

Glyma09g27100 Glyma01g02950 Glyma02g08920 Glyma14g36610 Glyma05g08670

Glyma10g34880 Glyma13g10640 Glyma03g40490 Glyma16g28080 Glyma03g38520

Glyma20g30590 Glyma10g35700 Glyma17g00320 Glyma16g06020 Glyma09g36000

Glyma11g00360 Glyma09g03020 Glyma03g41940 Glyma03g37790 Glyma05g28730

Glyma09g12320 Glyma09g32430 Glyma11g06870 Glyma09g14090 Glyma07g05230

Glyma14g07690 Glyma02g11530 Glyma02g46590 Glyma13g19830 Glyma19g03500

Glyma05g36590 Glyma07g00390 Glyma02g12870 Glyma19g02180 Glyma13g03650

Glyma08g41220 Glyma19g30600 Glyma03g40860 Glyma18g52430 Glyma01g06970

Glyma09g34640 Glyma16g27210 Glyma14g38580 Glyma13g19950 Glyma03g37780

Glyma06g47890 Glyma18g44190 Glyma06g11120 Glyma12g10240 Glyma01g35220

Glyma15g09820 Glyma13g39600 Glyma01g39350

4


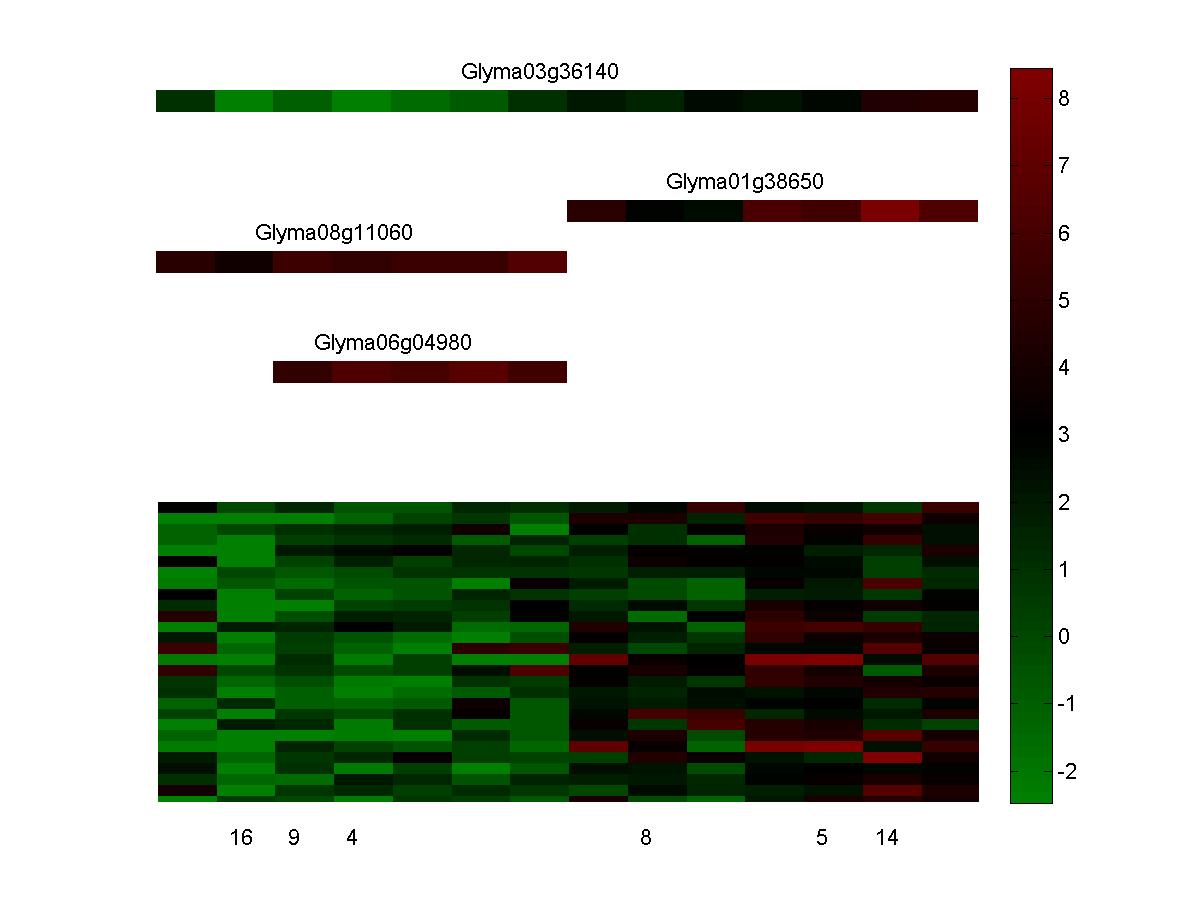


4 Glyma08g11060 HTH-FIS

4 Glyma03g36140 CCAAT

4 Glyma01g38650 Homeodomain/HOMEOBOX

4 Glyma06g04980 LIM

Glyma05g15700 Glyma02g04180 Glyma12g02590 Glyma11g04620 Glyma16g05770

Glyma18g15530 Glyma15g15610 Glyma13g38710 Glyma11g14300 Glyma08g23310

Glyma16g06740 Glyma20g32470 Glyma02g35190 Glyma02g06730 Glyma14g05840

Glyma02g42250 Glyma19g38800 Glyma03g36140 Glyma10g07500 Glyma04g42120

Glyma03g28080 Glyma01g03470 Glyma02g42730 Glyma19g01440 Glyma10g11060

Glyma09g21820 Glyma02g15520 Glyma19g44060

5


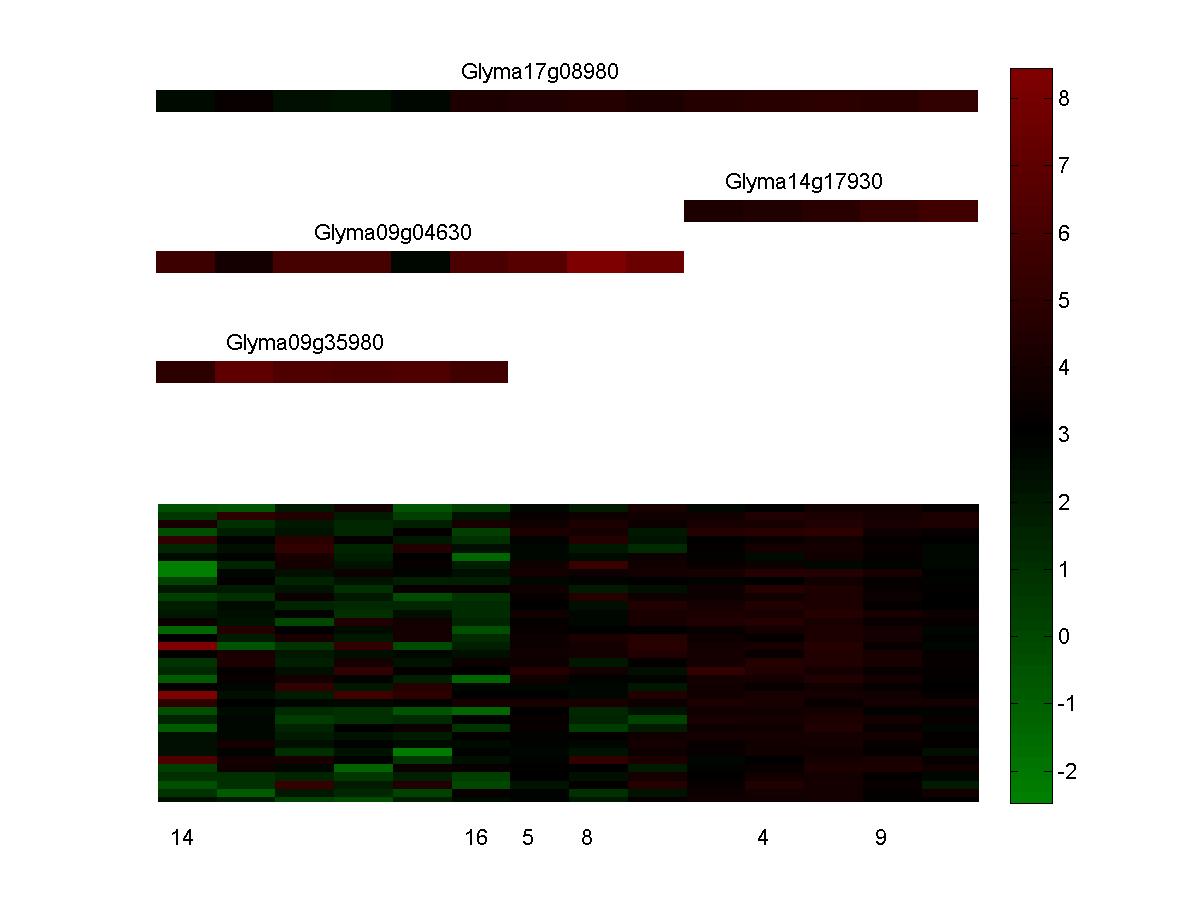


5 Glyma09g04630 AP2-EREBP

5 Glyma14g17930 CCHC (Zn)

5 Glyma09g35980 C3H-type1(Zn)

5 Glyma17g08980 bHLH

Glyma17g11870 Glyma15g08360 Glyma14g04950 Glyma07g09520 Glyma02g15400

Glyma17g36860 Glyma02g40440 Glyma15g13560 Glyma14g01960 Glyma07g09530

Glyma12g00790 Glyma17g35860 Glyma02g17020 Glyma10g35220 Glyma13g11980

Glyma05g08530 Glyma17g36400 Glyma17g08020 Glyma17g04440 Glyma17g08250

Glyma04g03260 Glyma18g53860 Glyma20g32970 Glyma09g24410 Glyma11g02000

Glyma03g20630 Glyma10g31180 Glyma11g37990 Glyma08g44820 Glyma04g40920

Glyma20g23930 Glyma17g13880 Glyma02g35550 Glyma17g36100 Glyma03g24020

Glyma03g31620 Glyma05g00640

6


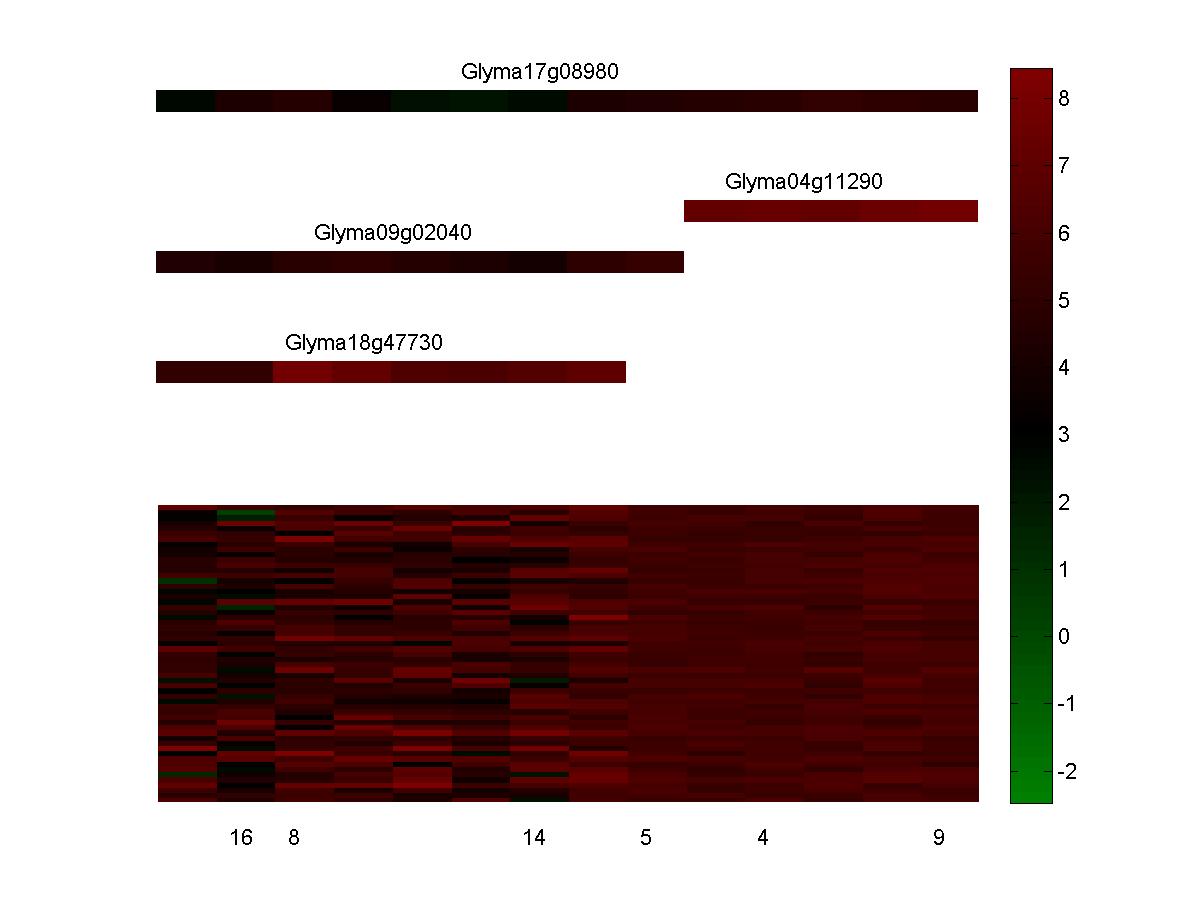


6 Glyma18g47730 C2H2 (Zn)

6 Glyma09g02040 MYB/HD-like

6 Glyma04g11290 AP2-EREBP

6 Glyma17g08980 bHLH

Glyma05g24850 Glyma08g03540 Glyma02g16740 Glyma08g05820 Glyma11g05030

Glyma18g08640 Glyma08g39390 Glyma02g40940 Glyma11g15090 Glyma08g09680

Glyma15g41960 Glyma10g29250 Glyma01g25270 Glyma04g10880 Glyma05g36100

Glyma01g38410 Glyma09g39530 Glyma05g31390 Glyma13g26990 Glyma02g12970

Glyma15g17680 Glyma09g23600 Glyma01g31600 Glyma16g03250 Glyma17g09500

Glyma18g47730 Glyma08g23870 Glyma05g24860 Glyma07g34730 Glyma20g38980

Glyma04g38140 Glyma08g07590 Glyma08g20060 Glyma15g38010 Glyma09g39230

Glyma13g37960 Glyma08g29130 Glyma05g04960 Glyma13g00380 Glyma18g04530

Glyma08g44130 Glyma11g18980 Glyma18g08630 Glyma15g06020 Glyma07g11890

Glyma05g03310 Glyma04g01920 Glyma03g28850 Glyma20g39380 Glyma06g03100

Glyma13g05120 Glyma12g17510 Glyma01g02410 Glyma02g42860 Glyma07g37100

Glyma16g32290 Glyma09g00670

7


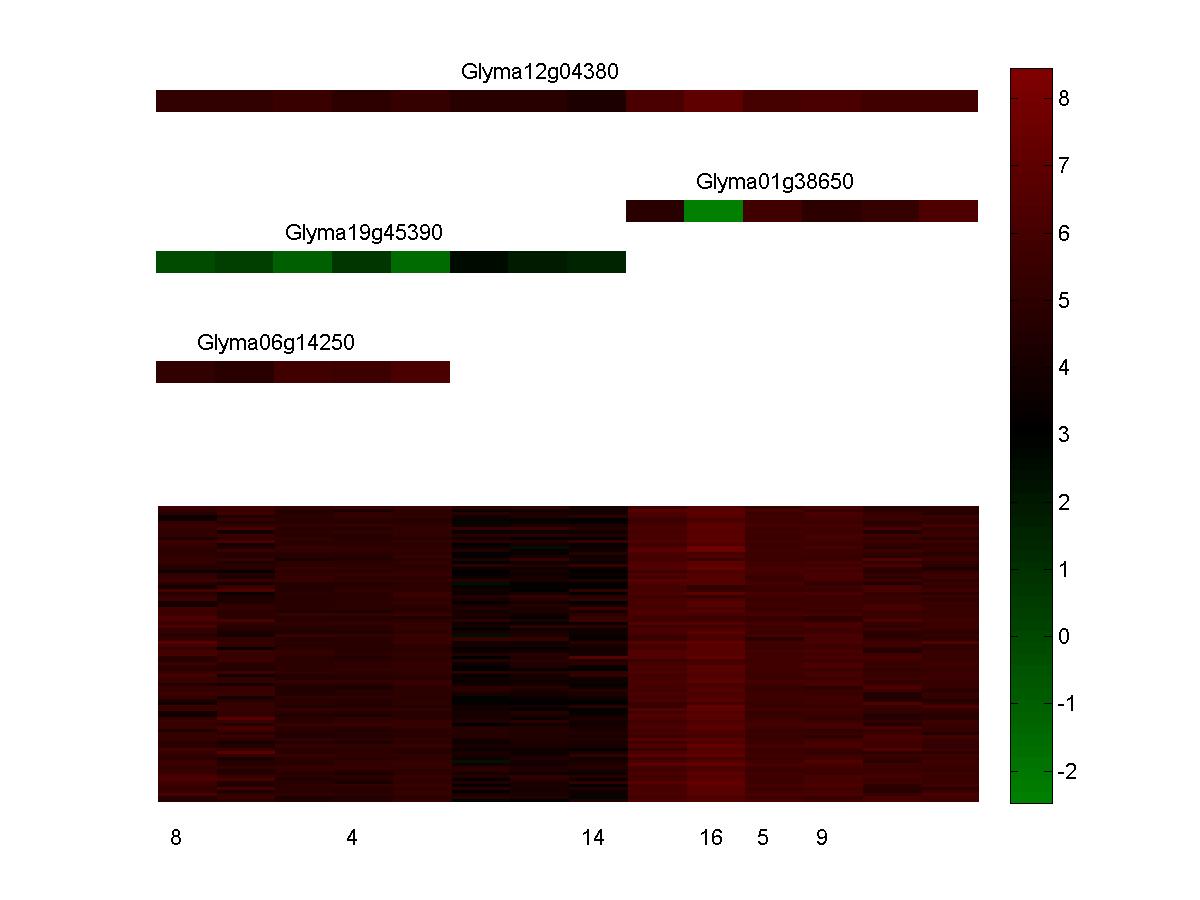


7 Glyma12g04380 NAC

7 Glyma06g14250 NAC

7 Glyma01g38650 Homeodomain/HOMEOBOX

7 Glyma19g45390 Homeodomain/HOMEOBOX

Glyma10g42940 Glyma02g45430 Glyma20g24830 Glyma17g11750 Glyma12g07040

Glyma02g00490 Glyma08g28800 Glyma13g34670 Glyma09g28440 Glyma02g02690

Glyma08g17000 Glyma08g24950 Glyma15g02610 Glyma08g04990 Glyma15g13650

Glyma18g02050 Glyma14g38950 Glyma03g31460 Glyma05g34680 Glyma19g35660

Glyma19g39940 Glyma16g04570 Glyma13g39490 Glyma09g38400 Glyma18g18050

Glyma03g35080 Glyma19g25930 Glyma17g23830 Glyma11g12200 Glyma11g02900

Glyma10g29600 Glyma18g02340 Glyma06g20700 Glyma06g24600 Glyma13g40780

Glyma17g05030 Glyma13g37600 Glyma04g43560 Glyma03g29810 Glyma02g39510

Glyma06g47510 Glyma07g31840 Glyma10g00500 Glyma12g32000 Glyma02g44460

Glyma20g22090 Glyma03g39480 Glyma20g29190 Glyma20g09810 Glyma10g40750

Glyma19g10300 Glyma05g27600 Glyma18g02970 Glyma15g04670 Glyma18g47920

Glyma18g49340 Glyma08g02070 Glyma16g05530 Glyma13g23200 Glyma13g16570

Glyma06g08680 Glyma06g46180 Glyma19g36250 Glyma15g42150 Glyma12g07030

Glyma05g04520 Glyma06g13870 Glyma15g40520 Glyma06g47520 Glyma09g06930

Glyma19g42320 Glyma01g40150 Glyma08g05470 Glyma16g07660 Glyma04g08570

Glyma19g39250 Glyma05g03900 Glyma19g42030 Glyma12g04400 Glyma13g18230

Glyma09g06940 Glyma13g31650 Glyma08g12040 Glyma11g34890 Glyma13g21520

Glyma11g16210 Glyma19g35980 Glyma20g35000 Glyma03g33830 Glyma10g24620

Glyma18g26190 Glyma12g04510 Glyma15g03120 Glyma07g38520 Glyma16g05090

Glyma14g17930 Glyma19g28740

8


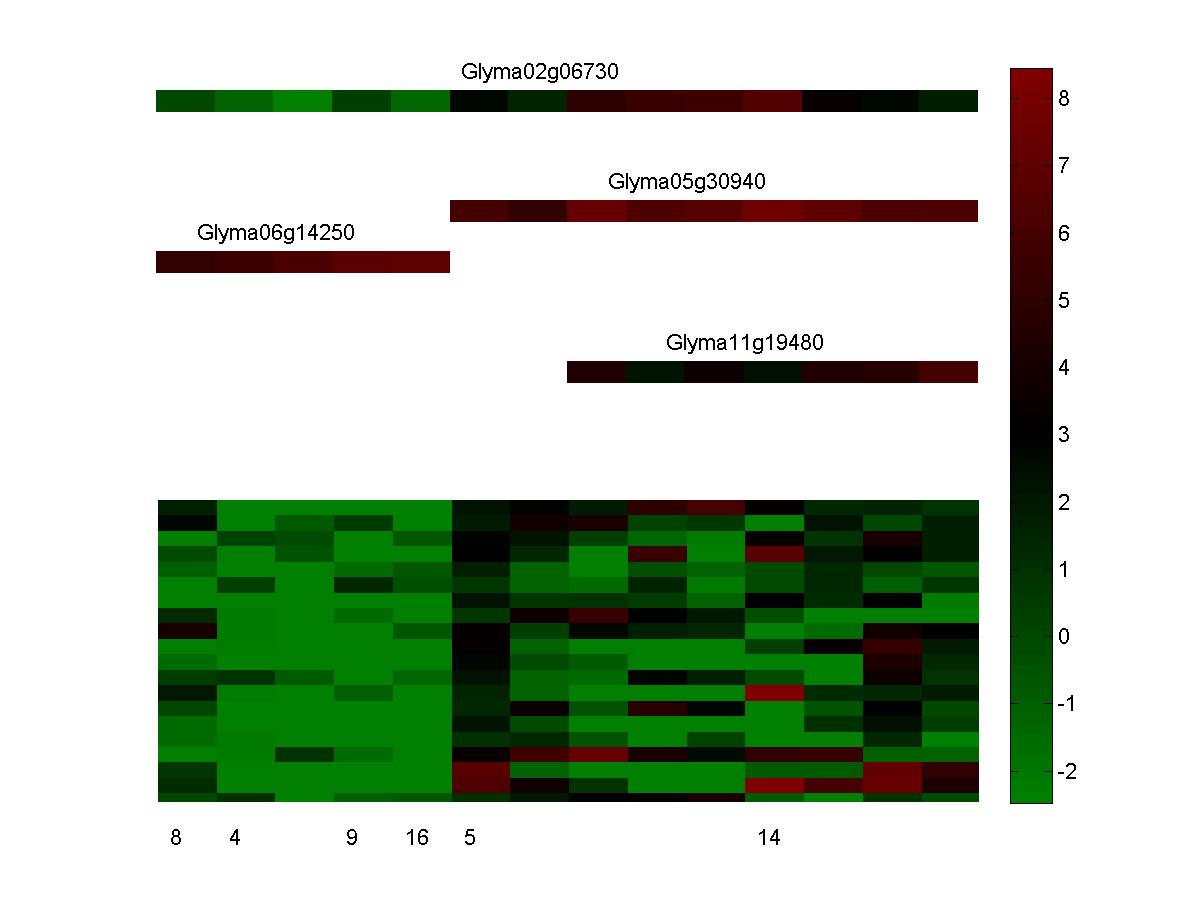


8 Glyma02g06730 Homeodomain/HOMEOBOX

8 Glyma05g30940 Homeodomain/HOMEOBOX

8 Glyma11g19480 C2H2 (Zn)

8 Glyma06g14250 NAC

Glyma17g09200 Glyma02g47170 Glyma08g48030 Glyma07g09710 Glyma11g19130

Glyma12g01130 Glyma17g35230 Glyma16g04760 Glyma15g01500 Glyma06g29670

Glyma11g37620 Glyma13g28970 Glyma14g00470 Glyma09g28750 Glyma18g00350

Glyma03g30930 Glyma06g08860 Glyma05g03750 Glyma08g12650 Glyma13g23850

9


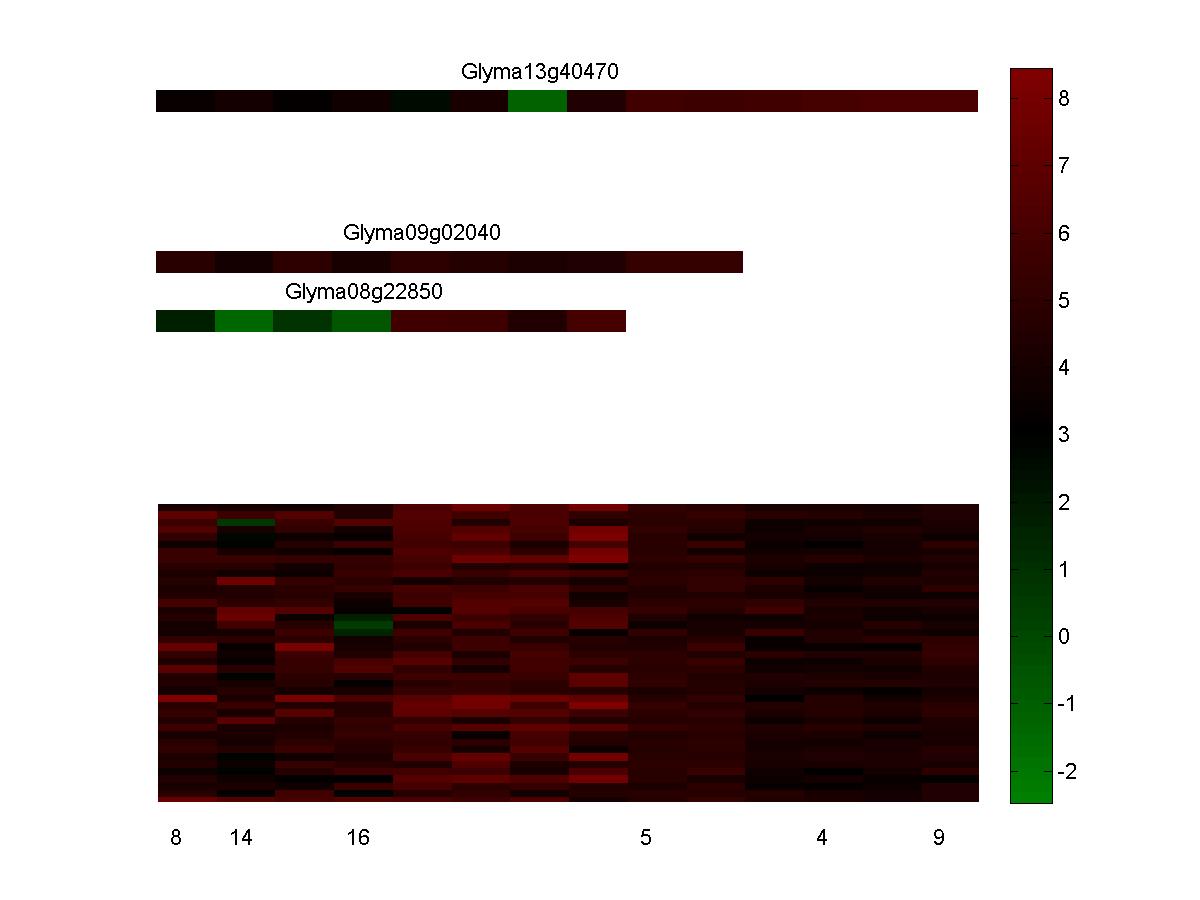


9 Glyma13g40470 AP2-EREBP

9 Glyma09g02040 MYB/HD-like

9 Glyma08g22850 TPR

Glyma06g18470 Glyma04g04540 Glyma08g01410 Glyma17g07900 Glyma09g36120

Glyma07g09060 Glyma10g00520 Glyma03g40640 Glyma04g00350 Glyma05g02670

Glyma05g37590 Glyma08g18310 Glyma02g09190 Glyma08g47310 Glyma04g40000

Glyma04g00420 Glyma19g38690 Glyma02g46330 Glyma05g05820 Glyma11g20600

Glyma07g16970 Glyma12g01340 Glyma04g17600 Glyma08g47990 Glyma19g40620

Glyma19g42260 Glyma09g33750 Glyma13g20830 Glyma09g31690 Glyma10g15250

Glyma15g01950 Glyma13g38730 Glyma02g44710 Glyma12g28970 Glyma05g02800

Glyma08g10160 Glyma07g09050 Glyma13g43360 Glyma20g38460 Glyma05g20710

Glyma09g04940

10


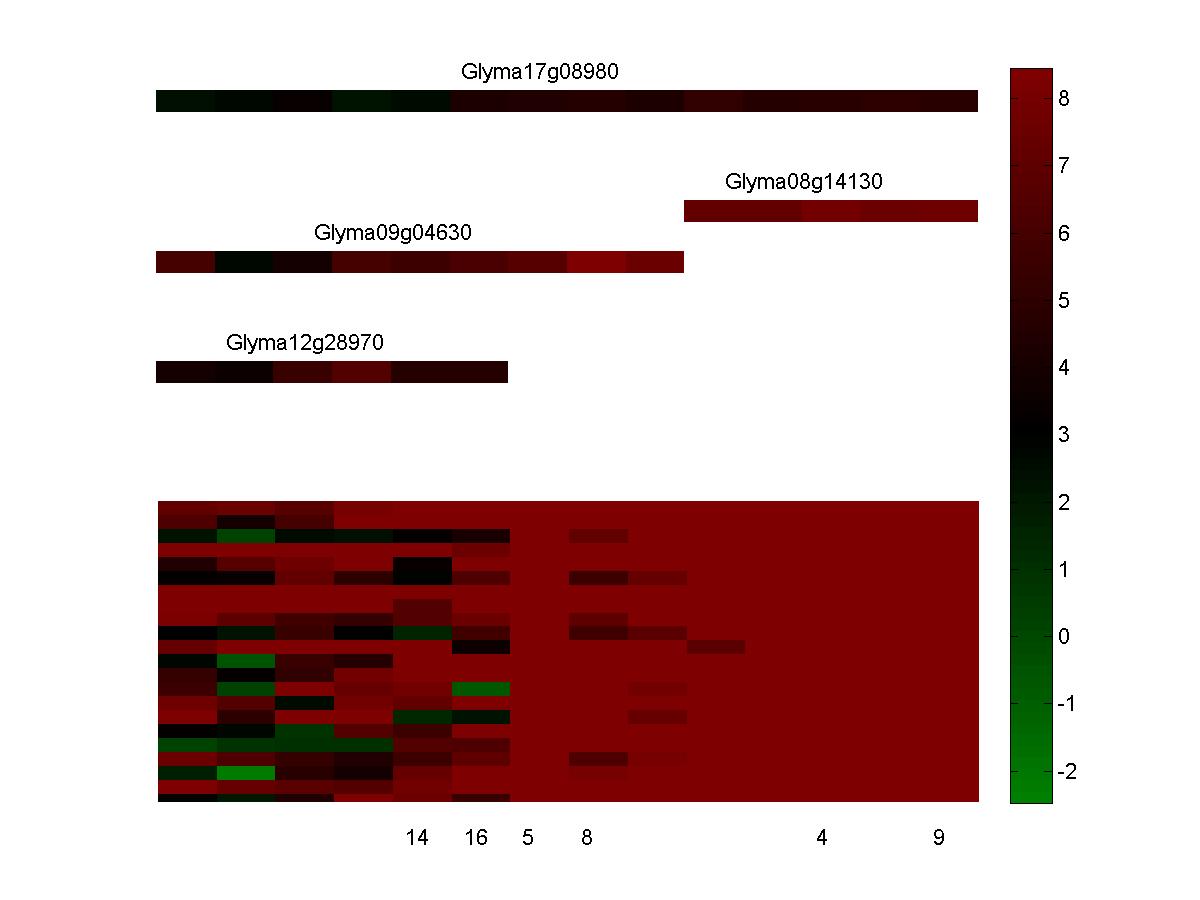


10 Glyma09g04630 AP2-EREBP

10 Glyma08g14130 Homeodomain/HOMEOBOX

10 Glyma17g08980 bHLH

10 Glyma12g28970 TCP

Glyma13g42330 Glyma07g00900 Glyma17g03350 Glyma17g37400 Glyma05g37730

Glyma12g06100 Glyma17g23900 Glyma03g34310 Glyma08g26150 Glyma11g14140

Glyma19g36440 Glyma10g35080 Glyma07g00910 Glyma08g21410 Glyma11g03690

Glyma07g01730 Glyma01g41670 Glyma13g32300 Glyma08g26140 Glyma10g35090

Glyma12g00390 Glyma09g12200

11


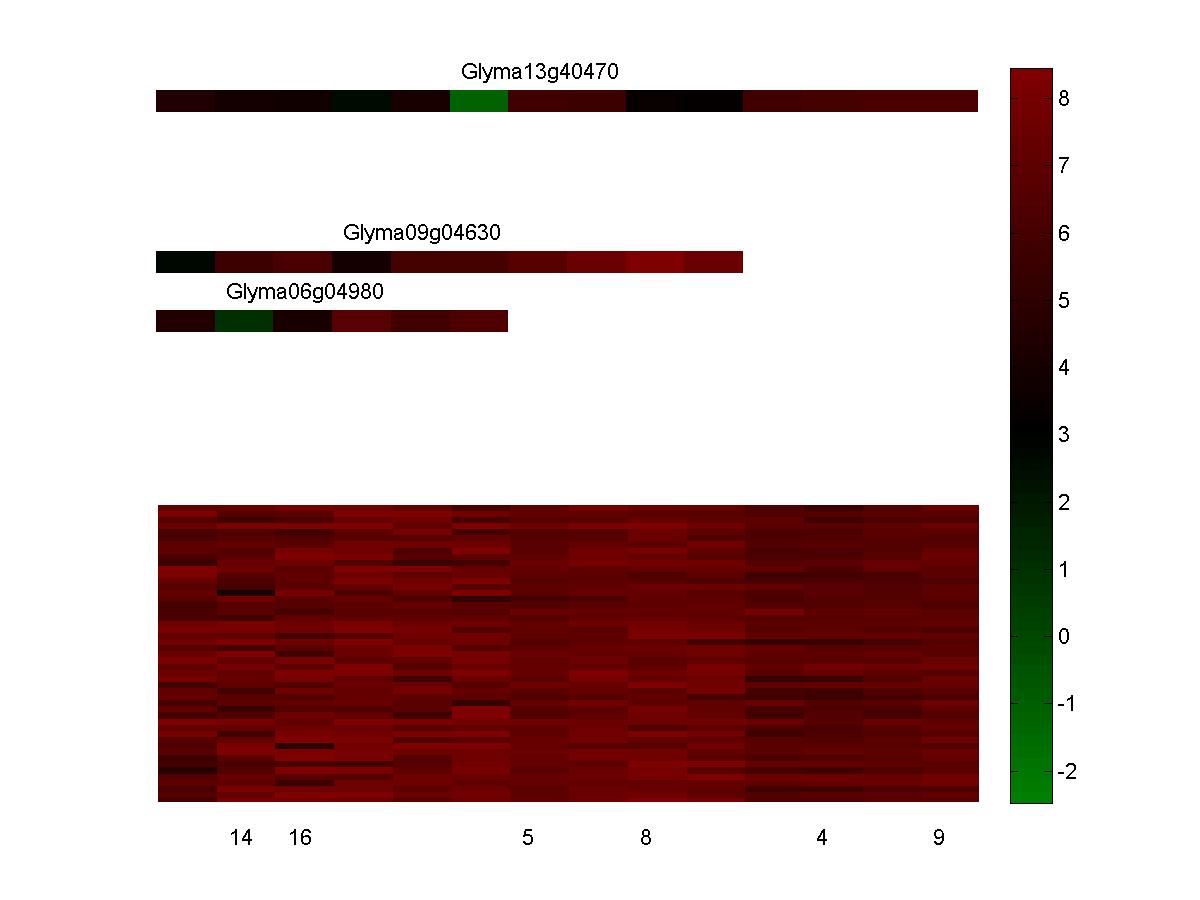


11 Glyma06g04980 LIM

11 Glyma09g04630 AP2-EREBP

11 Glyma13g40470 AP2-EREBP

Glyma11g33720 Glyma06g09420 Glyma08g10140 Glyma12g19520 Glyma20g12250

Glyma09g14860 Glyma18g01870 Glyma18g52860 Glyma17g06170 Glyma04g09820

Glyma11g12500 Glyma19g37500 Glyma17g12840 Glyma05g27180 Glyma18g00500

Glyma05g30940 Glyma11g08440 Glyma20g29660 Glyma06g07140 Glyma07g38940

Glyma06g44380 Glyma07g04950 Glyma08g18760 Glyma05g27190 Glyma04g03020

Glyma04g02270 Glyma08g14130 Glyma17g34920 Glyma18g02210 Glyma06g42070

Glyma05g37170 Glyma20g34880 Glyma18g04500 Glyma15g12530 Glyma17g17850

Glyma11g12510 Glyma05g00400 Glyma04g00500 Glyma03g36470 Glyma01g44040

Glyma06g00990 Glyma16g23730 Glyma04g13490 Glyma02g15190 Glyma16g01500

Glyma04g11290 Glyma13g17660 Glyma02g11580 Glyma15g15910

12


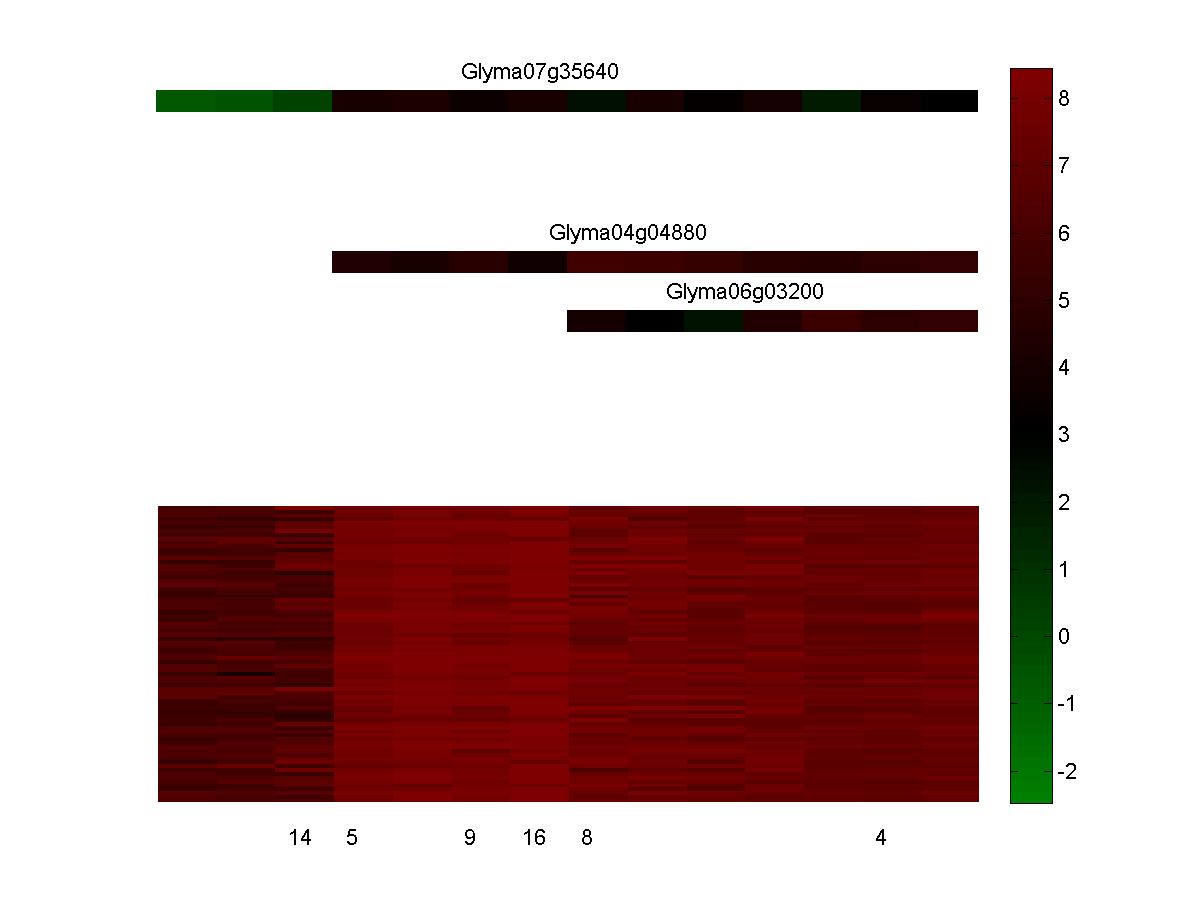


12 Glyma07g35640 TPR

12 Glyma04g04880 LIM

12 Glyma06g03200 Homeodomain/HOMEOBOX

Glyma19g39800 Glyma05g26290 Glyma08g03150 Glyma16g08460 Glyma06g20540

Glyma20g38480 Glyma02g47210 Glyma08g46850 Glyma11g37970 Glyma06g18120

Glyma04g33900 Glyma04g36140 Glyma20g23080 Glyma13g19330 Glyma10g28890

Glyma15g10950 Glyma09g24070 Glyma04g11400 Glyma13g28830 Glyma02g05540

Glyma13g06390 Glyma05g31760 Glyma03g40280 Glyma02g43790 Glyma18g53610

Glyma18g32680 Glyma20g30970 Glyma04g06700 Glyma10g36780 Glyma03g32380

Glyma01g02720 Glyma14g06170 Glyma16g10700 Glyma06g05410 Glyma14g36970

Glyma08g02410 Glyma16g24120 Glyma20g30810 Glyma10g04560 Glyma04g40470

Glyma03g36560 Glyma01g37250 Glyma13g19930 Glyma19g35600 Glyma04g36860

Glyma20g31070 Glyma03g21710 Glyma05g34350 Glyma04g07220 Glyma13g19470

Glyma13g23400 Glyma05g01180 Glyma04g39380 Glyma10g37960 Glyma01g26950

Glyma15g13080 Glyma19g39240 Glyma08g05290 Glyma20g25920 Glyma14g06630

Glyma19g38170 Glyma20g02170 Glyma15g42620 Glyma05g36420 Glyma10g41330

Glyma19g39070 Glyma10g40460 Glyma06g18110 Glyma03g37190 Glyma08g05610

Glyma02g11540 Glyma07g04890 Glyma18g14980 Glyma02g38450 Glyma05g34070

Glyma05g34570 Glyma04g16660

13


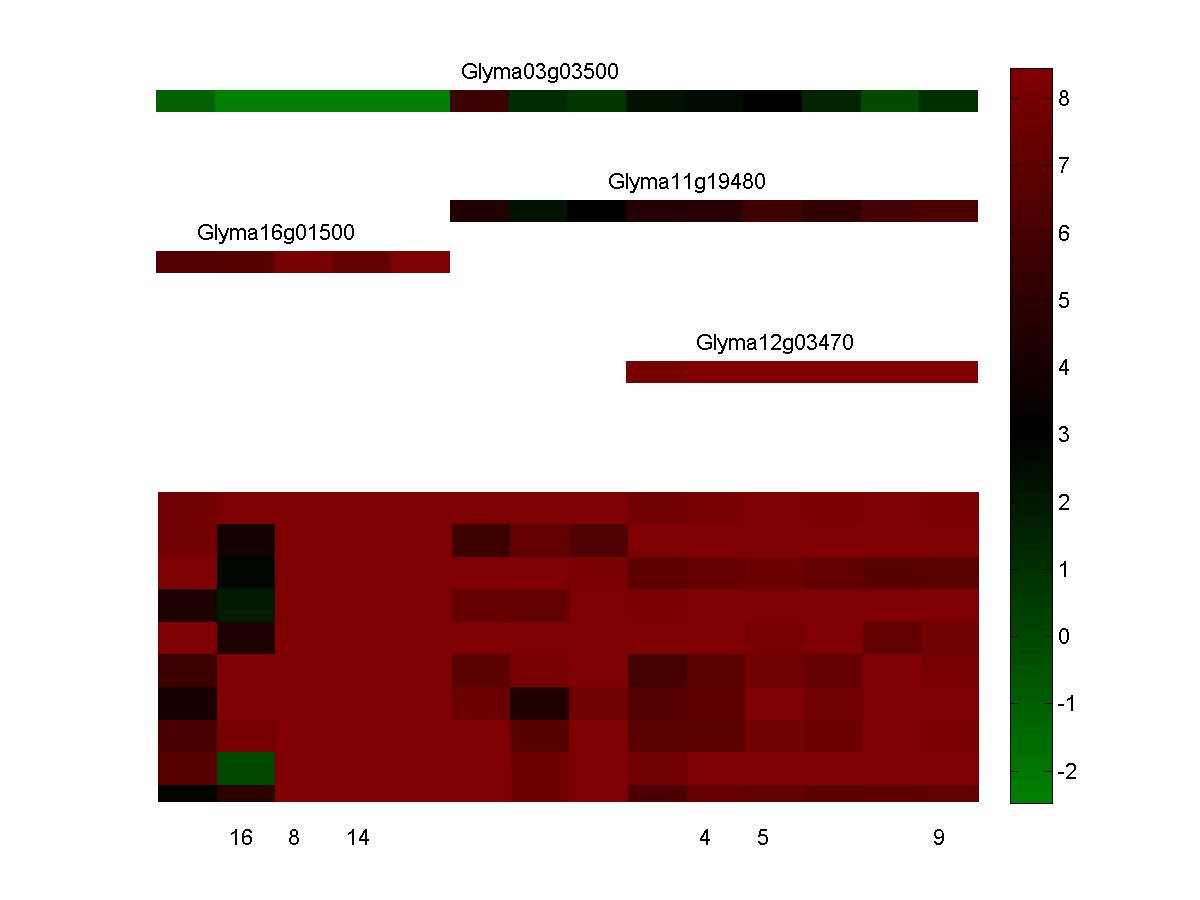


13 Glyma03g03500 C2C2 (Zn) YABBY

13 Glyma11g19480 C2H2 (Zn)

13 Glyma16g01500 AP2-EREBP

13 Glyma12g03470 CSD

Glyma09g04950 Glyma17g34870 Glyma18g39690 Glyma12g34550 Glyma07g15800

Glyma09g01320 Glyma15g12170 Glyma17g01720 Glyma12g34570 Glyma10g35870

14


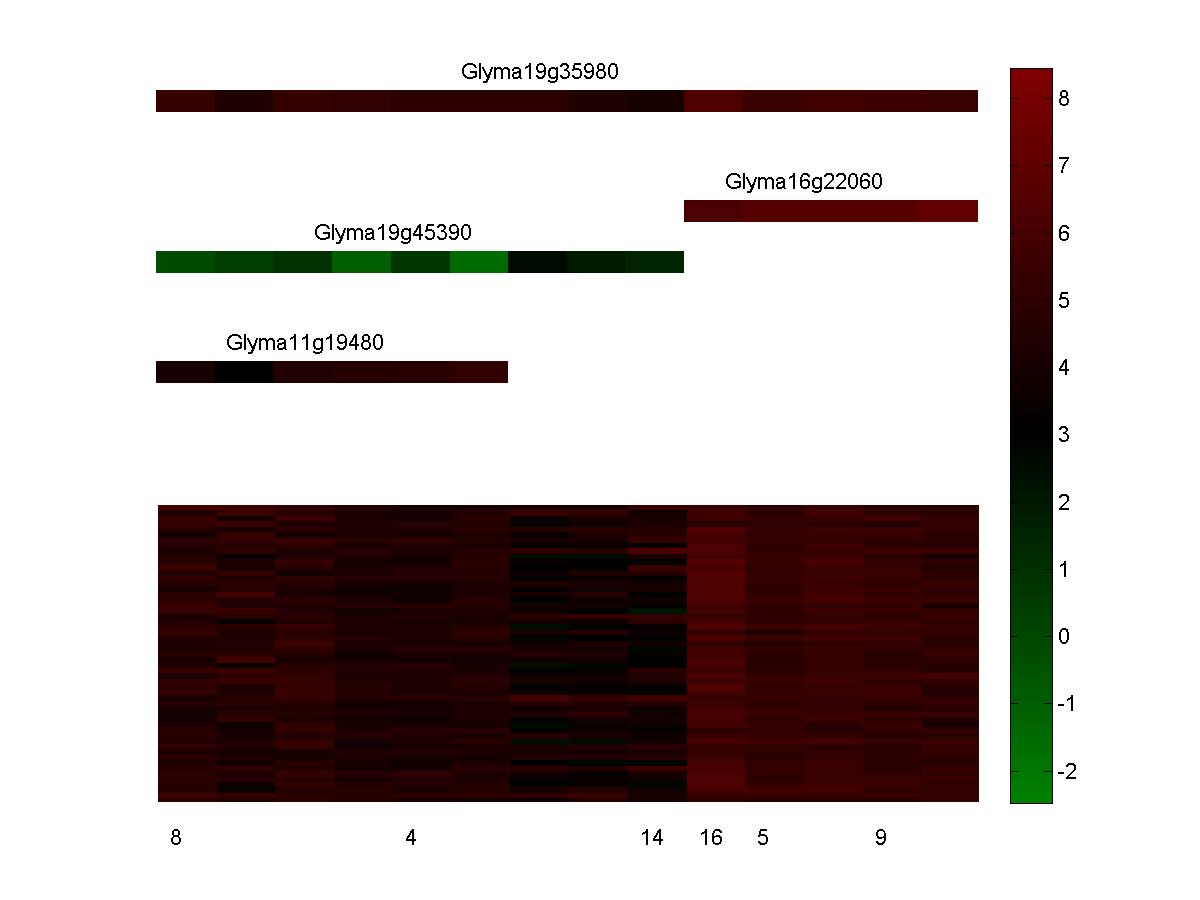


14 Glyma11g19480 C2H2 (Zn)

14 Glyma19g35980 CCHC (Zn)

14 Glyma19g45390 Homeodomain/HOMEOBOX

14 Glyma16g22060 HMG

Glyma20g22850 Glyma15g06890 Glyma17g07060 Glyma02g06120 Glyma19g29460

Glyma20g26530 Glyma02g40430 Glyma19g32690 Glyma13g00430 Glyma03g33530

Glyma05g24930 Glyma14g04350 Glyma08g08910 Glyma08g12030 Glyma11g36580

Glyma17g36970 Glyma14g02940 Glyma08g47920 Glyma02g46200 Glyma10g31550

Glyma05g09040 Glyma16g04040 Glyma19g37780 Glyma05g28790 Glyma15g10210

Glyma01g29950 Glyma17g29080 Glyma15g12050 Glyma02g45830 Glyma09g04280

Glyma06g13860 Glyma04g33750 Glyma04g42830 Glyma08g23260 Glyma19g28070

Glyma05g00440 Glyma14g36670 Glyma06g20870 Glyma08g18460 Glyma15g11340

Glyma03g32150 Glyma11g19780 Glyma13g24750 Glyma19g28750 Glyma19g20320

Glyma04g41530 Glyma01g01390 Glyma13g28840 Glyma06g11680 Glyma05g31830

Glyma02g44080 Glyma06g48130 Glyma04g33570 Glyma04g03290 Glyma12g13100

15


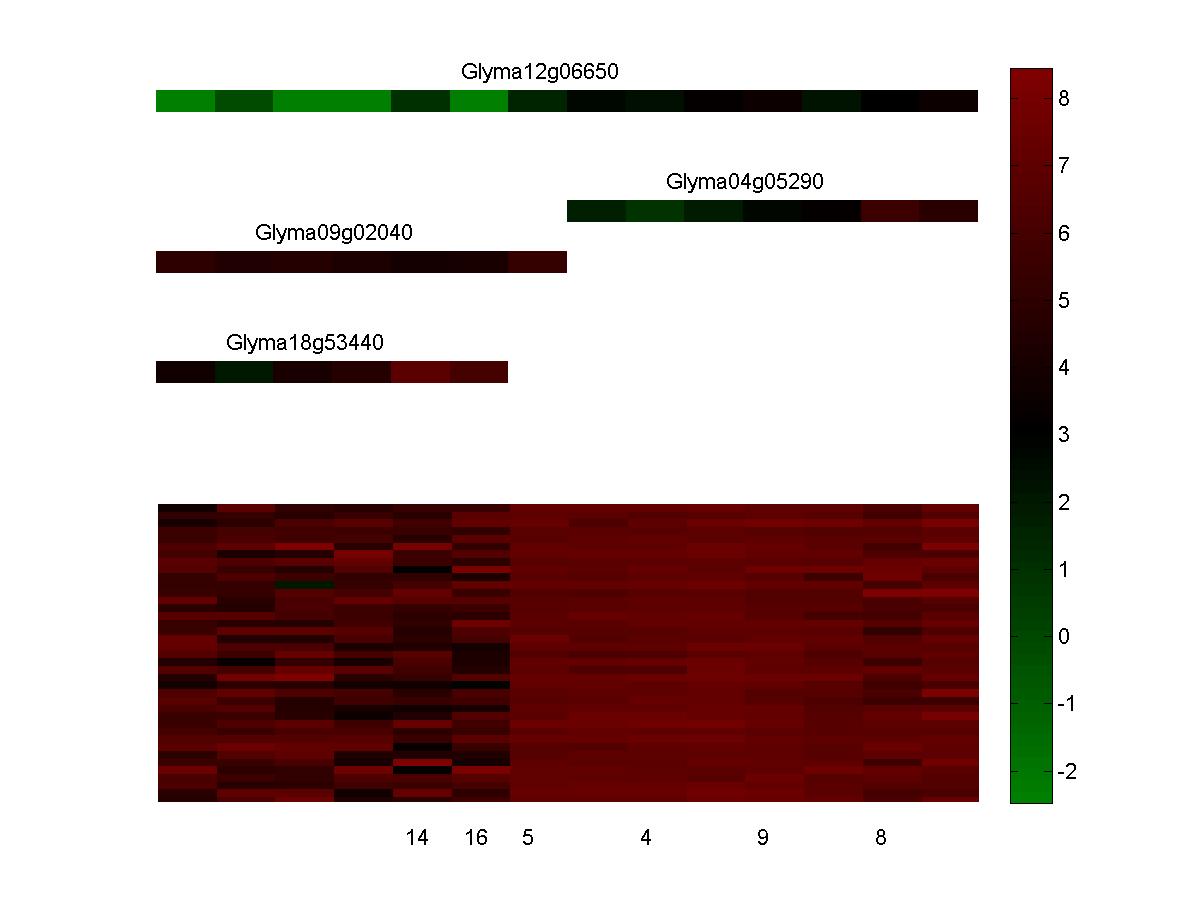


15 Glyma12g06650 GRAS

15 Glyma09g02040 MYB/HD-like

15 Glyma18g53440 AS2

15 Glyma04g05290 C3H-type1(Zn)

Glyma06g11970 Glyma08g07150 Glyma10g44370 Glyma04g42250 Glyma10g07820

Glyma01g02400 Glyma08g23860 Glyma03g36040 Glyma20g32000 Glyma13g17220

Glyma18g12210 Glyma10g38760 Glyma08g11850 Glyma12g00380 Glyma03g05620

Glyma03g34950 Glyma07g04500 Glyma13g40940 Glyma17g10490 Glyma19g41320

Glyma13g33590 Glyma20g26400 Glyma10g02370 Glyma16g22650 Glyma13g20800

Glyma04g03110 Glyma05g01390 Glyma01g24950 Glyma07g32020 Glyma08g25170

Glyma15g13970 Glyma03g40760 Glyma11g37920 Glyma04g19030 Glyma10g40150

Glyma06g07410 Glyma15g31750 Glyma19g02370 Glyma13g17820

16


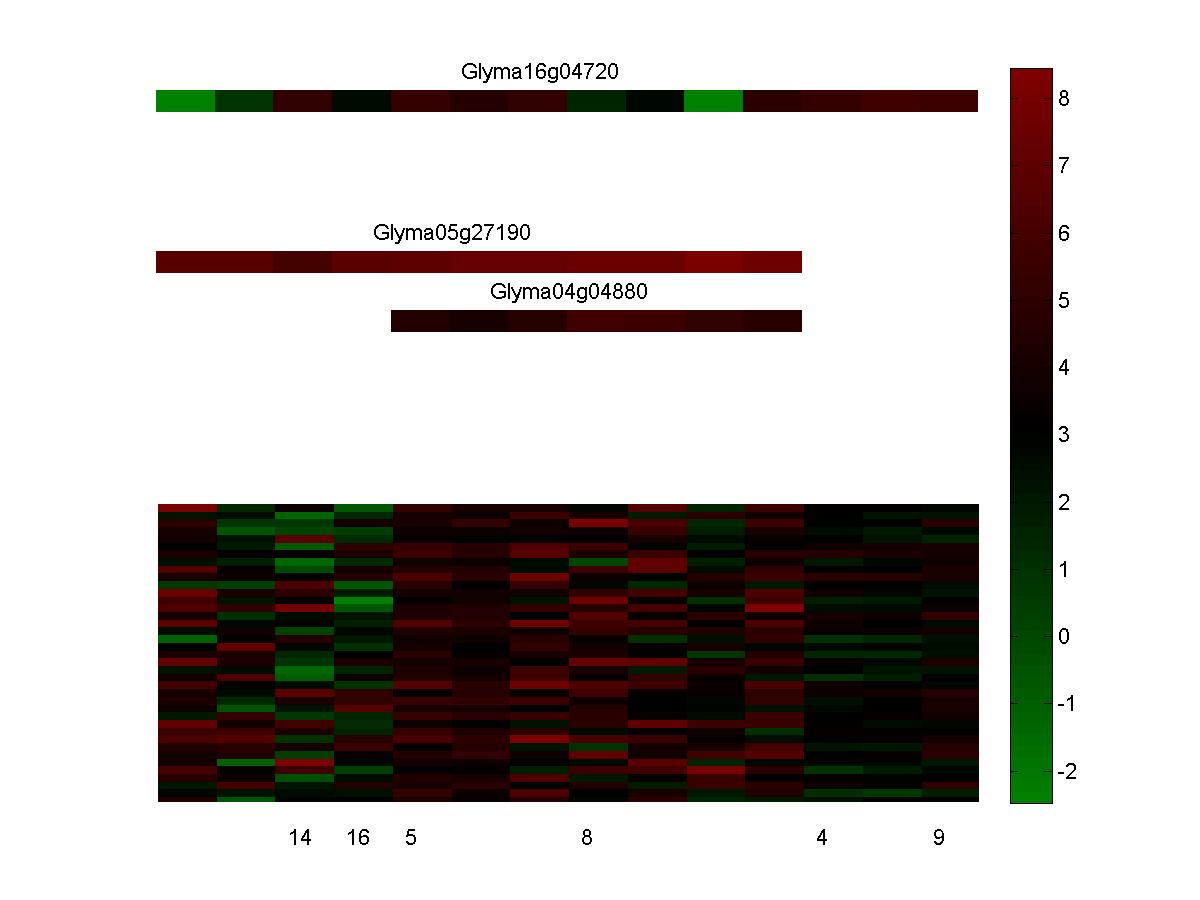


16 Glyma16g04720 NAC

16 Glyma05g27190 GRAS

16 Glyma04g04880 LIM

Glyma14g05850 Glyma14g06650 Glyma14g40680 Glyma04g34160 Glyma02g41590

Glyma15g14330 Glyma02g04760 Glyma02g14410 Glyma11g00290 Glyma12g01970

Glyma11g07750 Glyma09g36720 Glyma06g12680 Glyma08g18080 Glyma16g26630

Glyma13g24380 Glyma09g02330 Glyma20g29200 Glyma18g47930 Glyma03g05460

Glyma14g05250 Glyma14g06640 Glyma13g17570 Glyma07g32150 Glyma13g30490

Glyma13g44870 Glyma02g38750 Glyma18g50180 Glyma06g45860 Glyma06g36590

Glyma17g04940 Glyma17g35650 Glyma03g14210 Glyma19g44270 Glyma04g05290

Glyma12g33070 Glyma15g16560 Glyma17g17310 Glyma09g03400

17


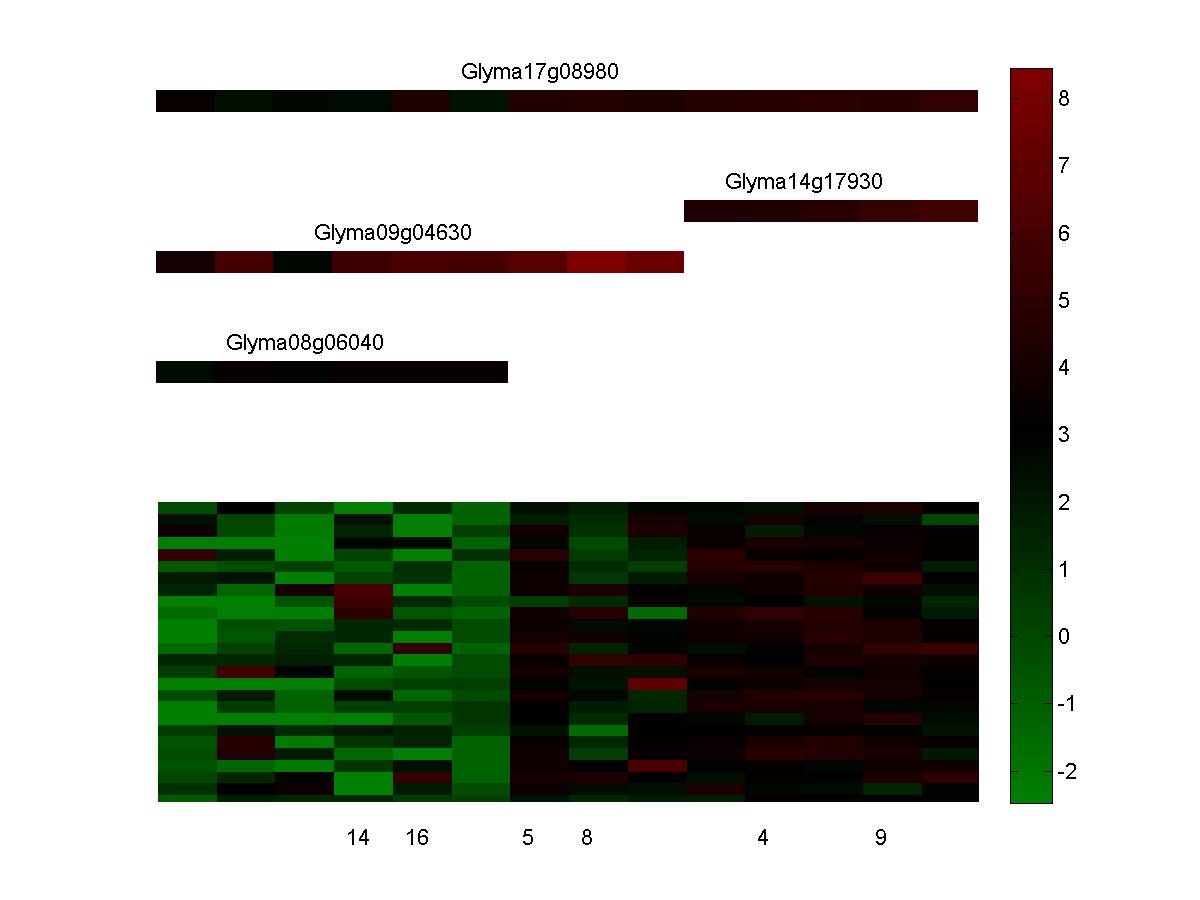


17 Glyma17g08980 bHLH

17 Glyma09g04630 AP2-EREBP

17 Glyma08g06040 PHD

17 Glyma14g17930 CCHC (Zn)

Glyma13g08060 Glyma12g36360 Glyma20g04130 Glyma02g43860 Glyma15g21830

Glyma09g40300 Glyma13g23740 Glyma10g43340 Glyma10g02080 Glyma01g26570

Glyma09g34380 Glyma13g37830 Glyma19g03730 Glyma02g44690 Glyma20g26600

Glyma03g31690 Glyma07g37280 Glyma05g29510 Glyma15g13880 Glyma06g13420

Glyma13g39240 Glyma05g28810 Glyma09g27510 Glyma12g34440 Glyma04g02750

Glyma20g29490

18


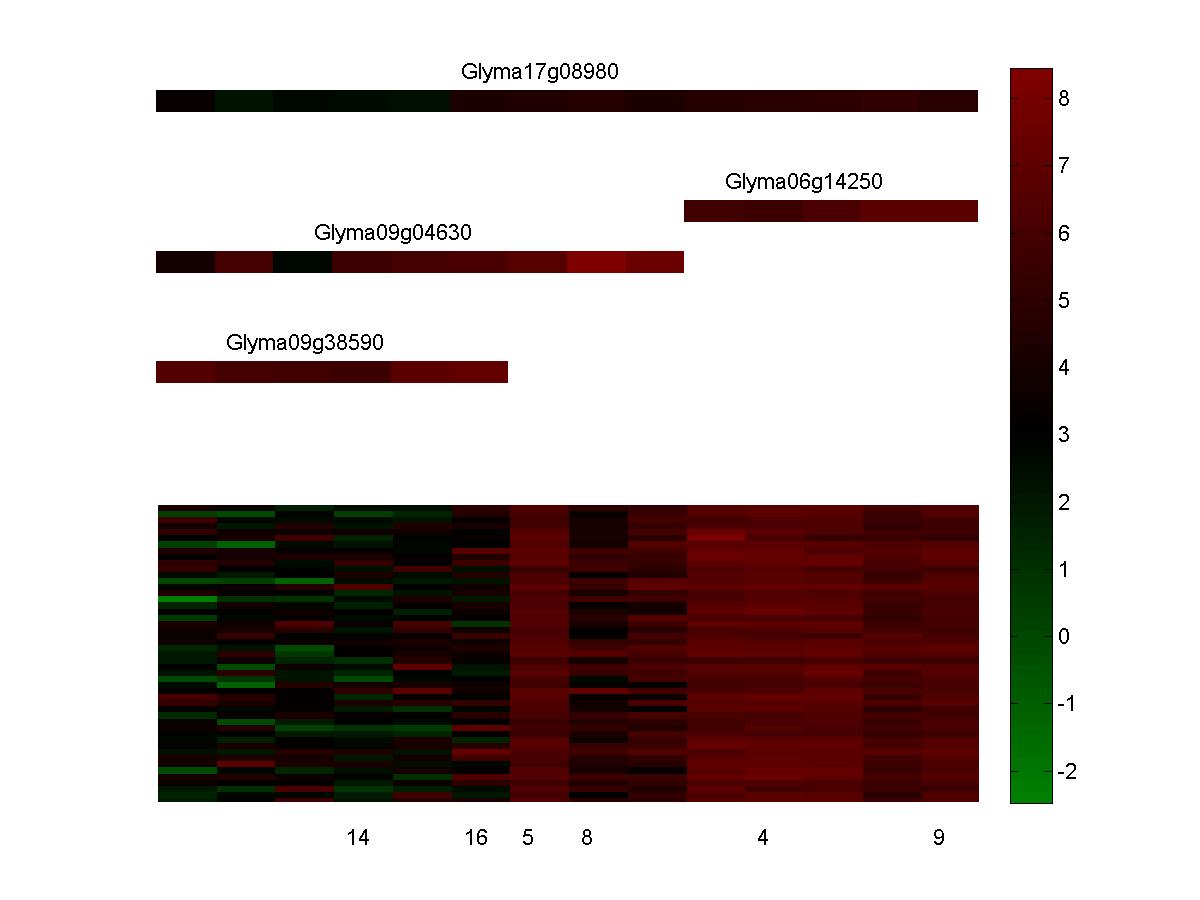


18 Glyma09g04630 AP2-EREBP

18 Glyma17g08980 bHLH

18 Glyma09g38590 C2H2 (Zn)

18 Glyma06g14250 NAC

Glyma08g25150 Glyma18g05700 Glyma07g39630 Glyma04g27810 Glyma15g03460

Glyma17g12150 Glyma05g29310 Glyma01g07070 Glyma18g50760 Glyma16g01790

Glyma07g01250 Glyma19g33730 Glyma01g37810 Glyma06g04140 Glyma19g01100

Glyma03g31950 Glyma08g17230 Glyma08g45920 Glyma20g39190 Glyma17g07190

Glyma04g40350 Glyma20g00760 Glyma05g14760 Glyma02g18380 Glyma01g45000

Glyma08g13640 Glyma15g18430 Glyma13g33890 Glyma06g01570 Glyma13g40470

Glyma02g02560 Glyma03g15130 Glyma09g14380 Glyma09g40970 Glyma09g01390

Glyma04g34600 Glyma08g00320 Glyma06g20070 Glyma04g42240 Glyma15g02700

Glyma16g27440 Glyma14g02070 Glyma16g29220 Glyma15g41970 Glyma13g06050

Glyma09g38990 Glyma13g30770 Glyma08g11960 Glyma17g33420

19


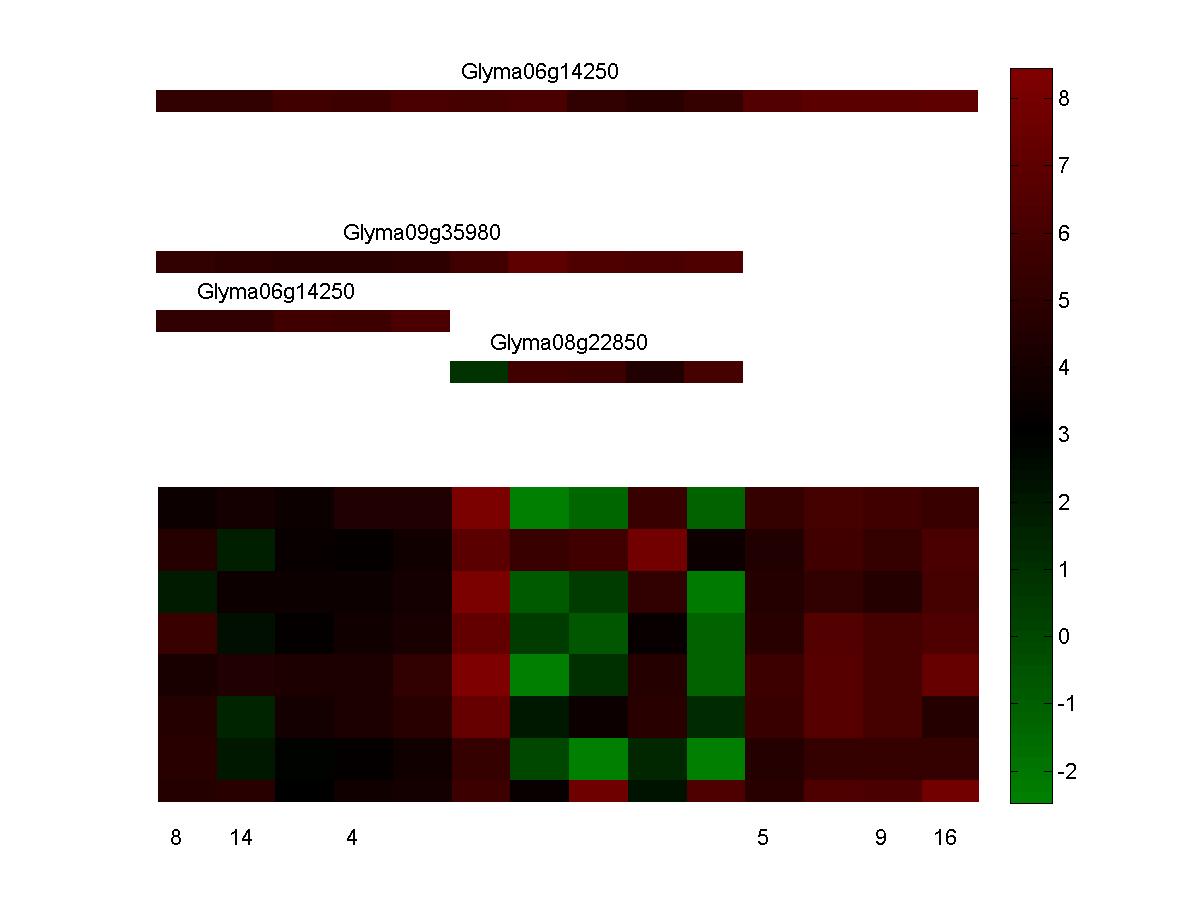


19 Glyma06g14250 NAC

19 Glyma09g35980 C3H-type1(Zn)

19 Glyma08g22850 TPR

Glyma17g14890 Glyma10g36690 Glyma05g04390 Glyma13g38310 Glyma17g14850

Glyma17g14910 Glyma06g38540 Glyma16g07750

20


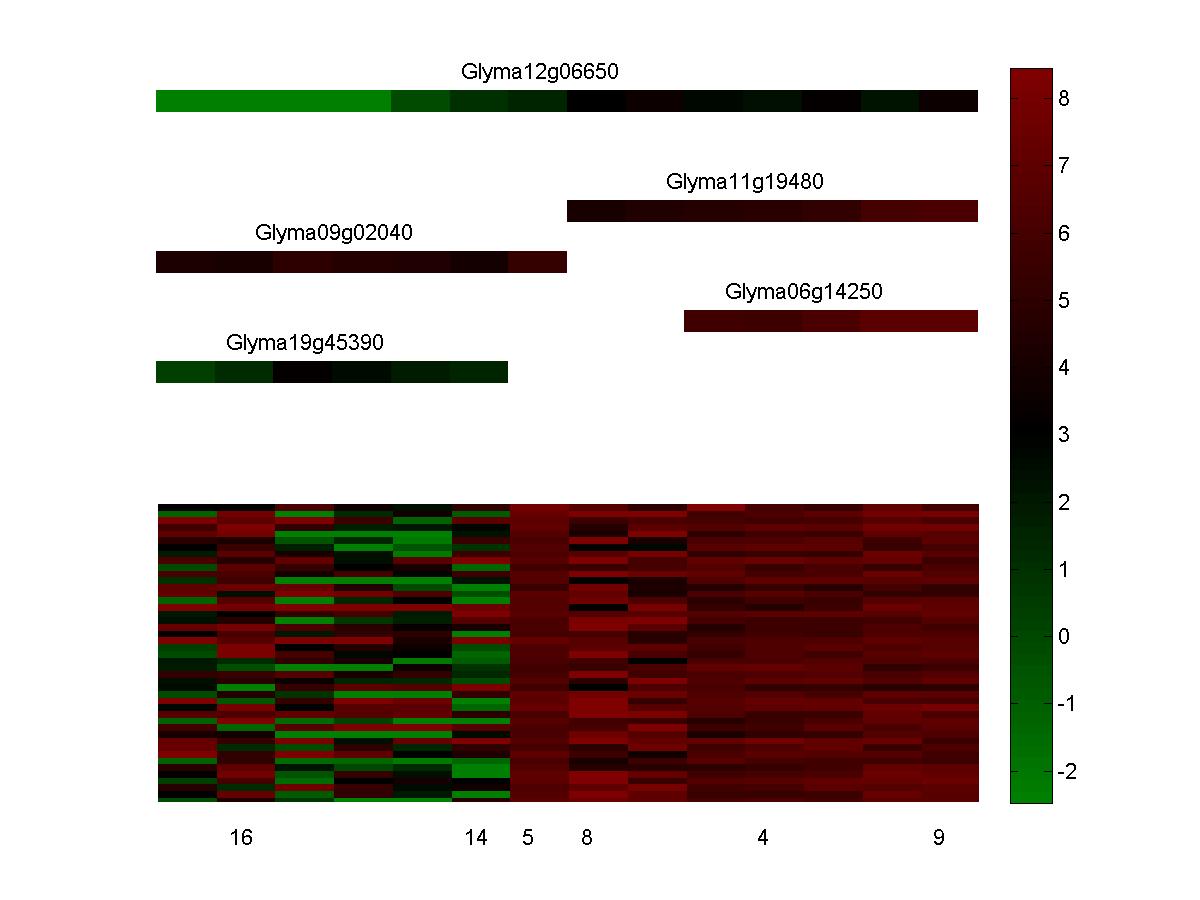


20 Glyma12g06650 GRAS

20 Glyma19g45390 Homeodomain/HOMEOBOX

20 Glyma06g14250 NAC

20 Glyma09g02040 MYB/HD-like

20 Glyma11g19480 C2H2 (Zn)

Glyma20g30910 Glyma17g14930 Glyma12g06950 Glyma18g49240 Glyma05g04440

Glyma17g03040 Glyma08g42440 Glyma16g27880 Glyma02g43470 Glyma04g42460

Glyma09g04630 Glyma09g37910 Glyma02g01990 Glyma13g34290 Glyma17g14920

Glyma14g09510 Glyma10g39450 Glyma09g04530 Glyma17g11940 Glyma08g01740

Glyma07g03910 Glyma06g12340 Glyma17g01220 Glyma07g09110 Glyma12g08520

Glyma07g04470 Glyma19g01940 Glyma01g38650 Glyma14g38170 Glyma02g09780

Glyma10g32340 Glyma02g00340 Glyma06g16810 Glyma17g34900 Glyma18g50500

Glyma14g05510 Glyma15g30110 Glyma13g42340 Glyma15g06000 Glyma17g02000

Glyma05g04490 Glyma03g04990 Glyma16g28150 Glyma05g04500 Glyma14g38180

21


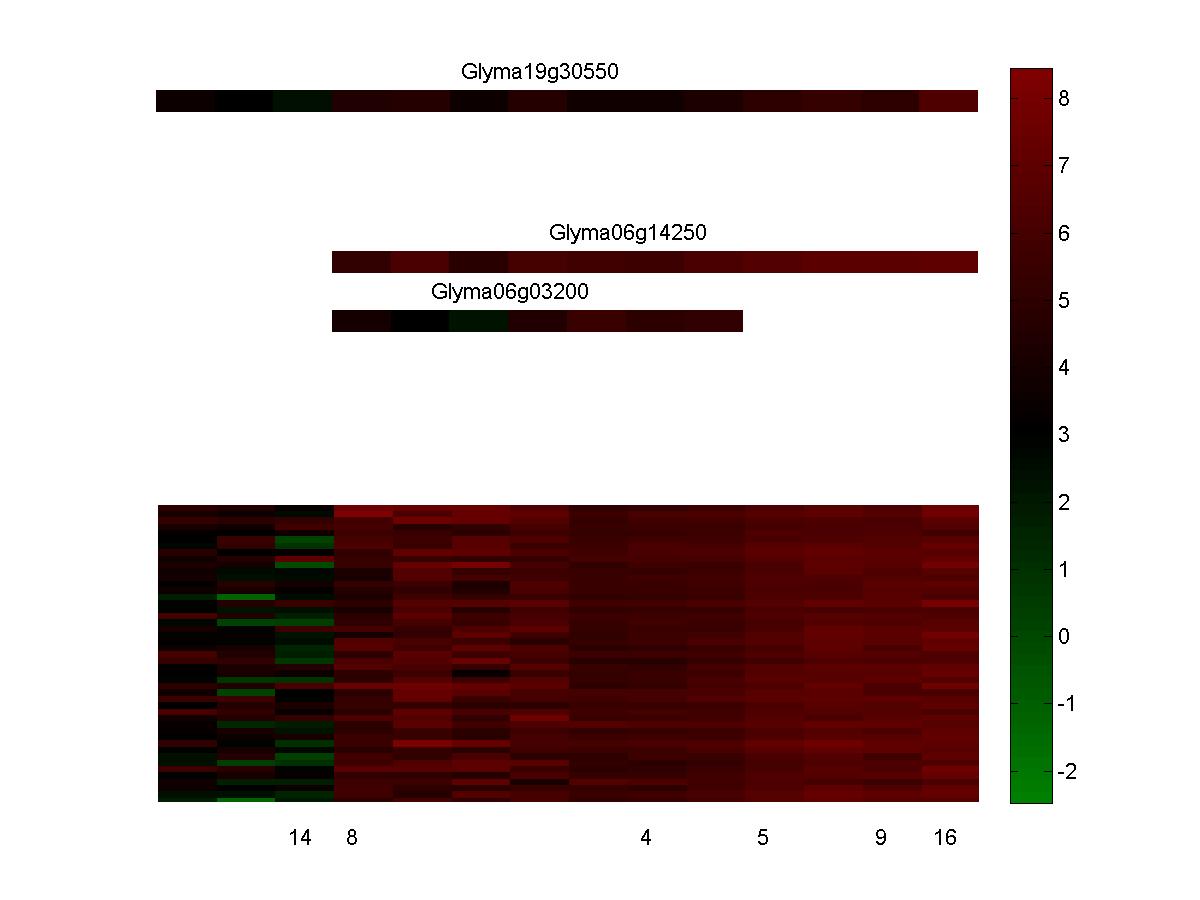


21 Glyma19g30550 NAC

21 Glyma06g14250 NAC

21 Glyma06g03200 Homeodomain/HOMEOBOX

Glyma06g15520 Glyma10g11620 Glyma17g08570 Glyma20g02250 Glyma19g42760

Glyma03g22260 Glyma11g05470 Glyma17g07070 Glyma10g02060 Glyma04g00450

Glyma13g21050 Glyma12g07050 Glyma10g37840 Glyma12g30800 Glyma08g24380

Glyma08g19290 Glyma15g04520 Glyma20g36890 Glyma05g32210 Glyma16g01650

Glyma07g38460 Glyma07g03220 Glyma17g17970 Glyma20g36880 Glyma08g08970

Glyma13g39270 Glyma17g13770 Glyma11g13940 Glyma08g15480 Glyma19g42240

Glyma09g33070 Glyma02g01950 Glyma10g30560 Glyma14g06310 Glyma02g38920

Glyma02g36070 Glyma10g00680 Glyma08g45810 Glyma13g19650 Glyma11g05480

Glyma13g24720 Glyma01g20460 Glyma10g05660 Glyma05g33860 Glyma03g40130

Glyma16g09760 Glyma16g23900

22


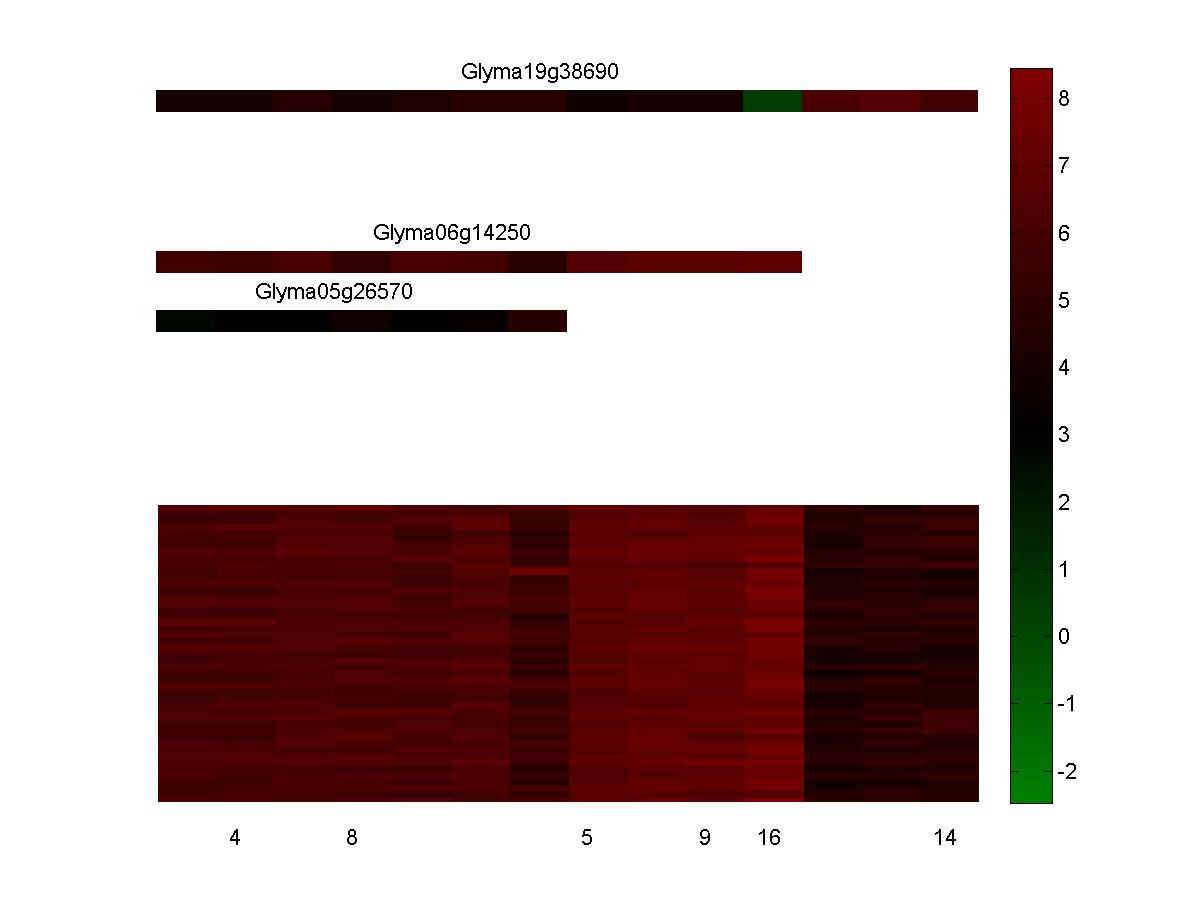


22 Glyma05g26570 TPR

22 Glyma19g38690 Homeodomain/HOMEOBOX

22 Glyma06g14250 NAC

Glyma02g13180 Glyma11g37160 Glyma10g07850 Glyma12g04020 Glyma06g01310

Glyma04g37120 Glyma02g11920 Glyma20g26870 Glyma10g39050 Glyma08g10970

Glyma08g44210 Glyma05g35030 Glyma07g00700 Glyma09g34760 Glyma12g08050

Glyma20g38970 Glyma16g01460 Glyma17g03550 Glyma03g40530 Glyma11g15230

Glyma16g23580 Glyma08g45770 Glyma13g42830 Glyma15g23220 Glyma20g37190

Glyma10g02270 Glyma15g17010 Glyma03g42180 Glyma08g09230 Glyma11g11040

Glyma12g03230 Glyma17g15970 Glyma14g06820 Glyma15g01860 Glyma03g40110

Glyma12g07160 Glyma15g05670 Glyma0169s00200 Glyma07g06580 Glyma08g04690

Glyma05g05670 Glyma08g20960 Glyma19g43190 Glyma01g44700 Glyma11g05160

Glyma11g09590 Glyma20g38130

23


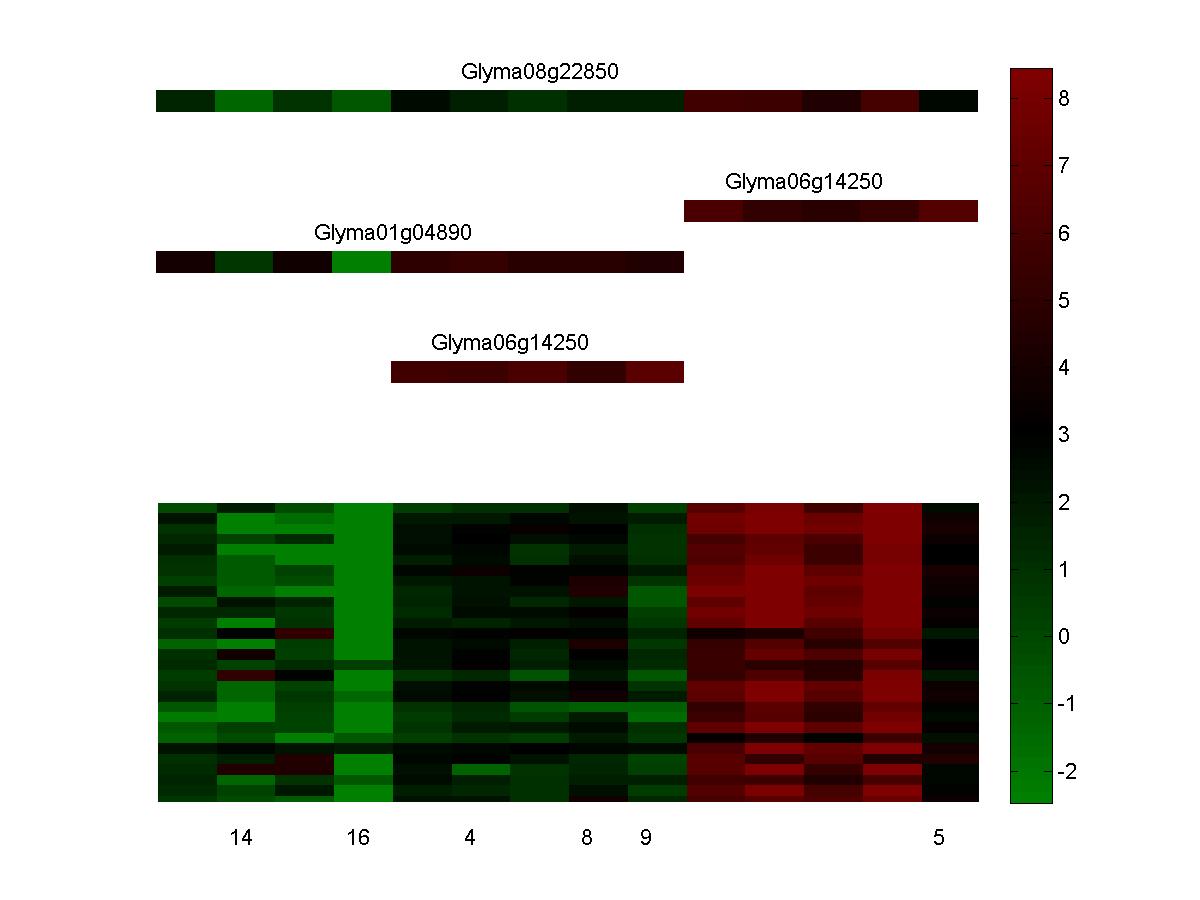


23 Glyma01g04890 Homeodomain/HOMEOBOX

23 Glyma08g22850 TPR

23 Glyma06g14250 NAC

Glyma12g32580 Glyma10g32080 Glyma02g47560 Glyma18g14410 Glyma20g38720

Glyma09g08630 Glyma14g00640 Glyma13g35800 Glyma12g09730 Glyma04g01020

Glyma04g33360 Glyma09g28200 Glyma06g08540 Glyma20g21100 Glyma08g13790

Glyma19g25720 Glyma15g10890 Glyma13g07610 Glyma04g12510 Glyma15g19510

Glyma08g21900 Glyma06g20960 Glyma03g31470 Glyma17g13930 Glyma17g05760

Glyma20g27990 Glyma08g22850 Glyma06g15540 Glyma15g43100

24


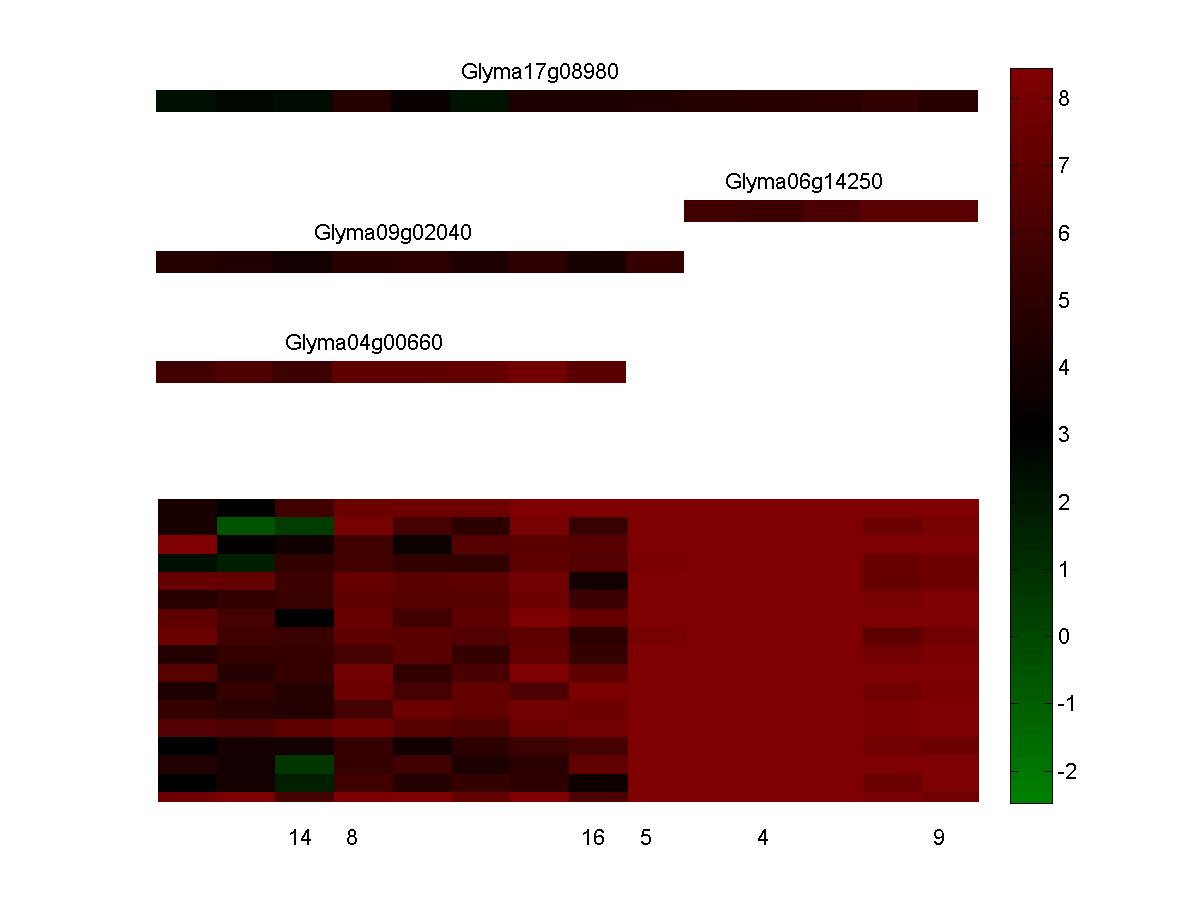


24 Glyma09g02040 MYB/HD-like

24 Glyma06g14250 NAC

24 Glyma04g00660 CSD

24 Glyma17g08980 bHLH

Glyma16g33710 Glyma09g02600 Glyma07g38110 Glyma08g00780 Glyma08g23560

Glyma07g33780 Glyma12g29510 Glyma10g40870 Glyma20g26440 Glyma16g28600

Glyma19g32990 Glyma02g09200 Glyma08g11070 Glyma10g29260 Glyma10g15980

Glyma01g26840 Glyma10g06600

25


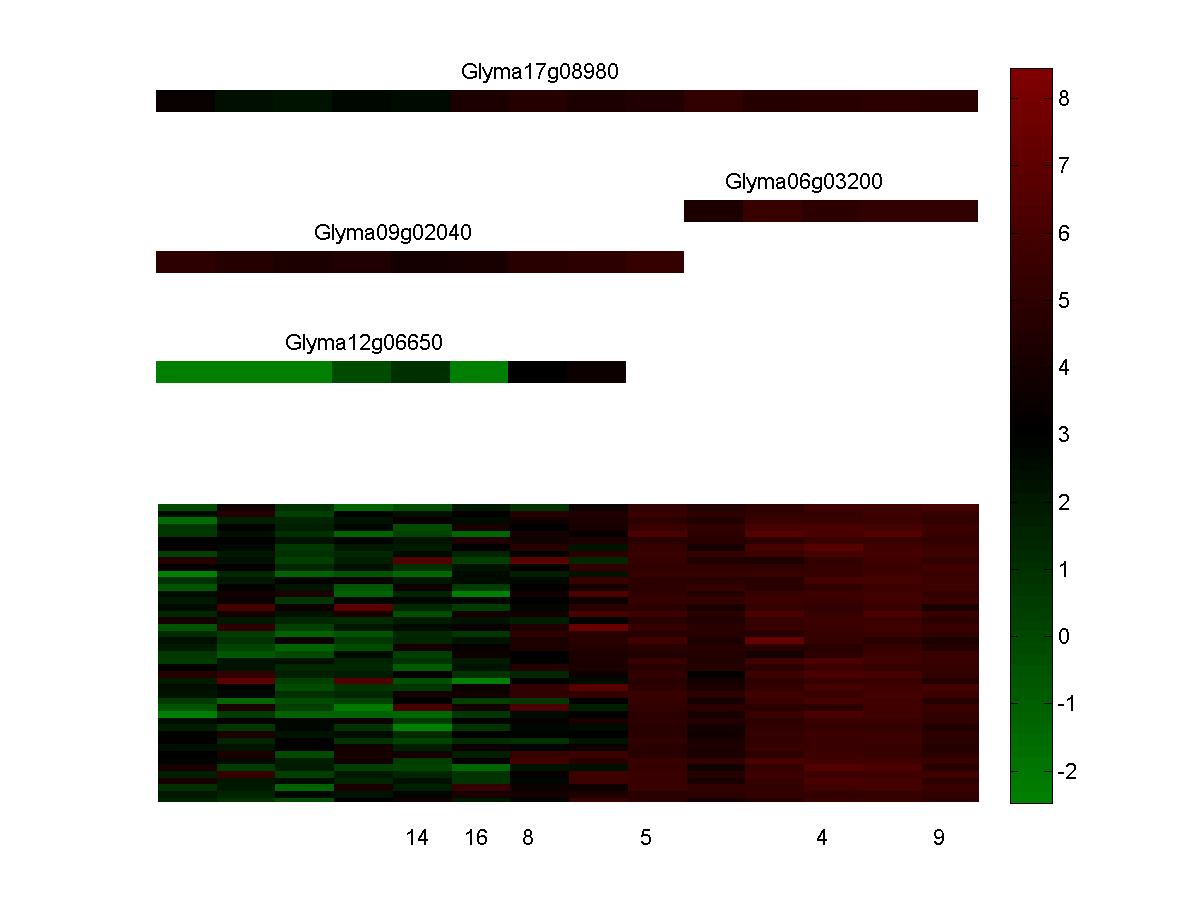


25 Glyma12g06650 GRAS

25 Glyma06g03200 Homeodomain/HOMEOBOX

25 Glyma17g08980 bHLH

25 Glyma09g02040 MYB/HD-like

Glyma07g38580 Glyma18g07220 Glyma08g14600 Glyma16g02490 Glyma10g28610

Glyma03g33560 Glyma15g14790 Glyma02g15780 Glyma03g38130 Glyma20g07060

Glyma09g15090 Glyma15g41700 Glyma15g12200 Glyma09g02590 Glyma01g36170

Glyma04g37320 Glyma11g03430 Glyma13g30590 Glyma04g04240 Glyma08g44320

Glyma12g33420 Glyma02g37310 Glyma17g34590 Glyma18g44950 Glyma04g33010

Glyma07g05620 Glyma14g05390 Glyma07g16850 Glyma17g15860 Glyma14g35660

Glyma18g44250 Glyma08g46610 Glyma07g39130 Glyma04g21810 Glyma11g30110

Glyma15g38070 Glyma17g03390 Glyma11g10340 Glyma18g16720 Glyma20g28490

Glyma05g30690 Glyma20g32140 Glyma10g32190 Glyma01g41270 Glyma01g04380

26


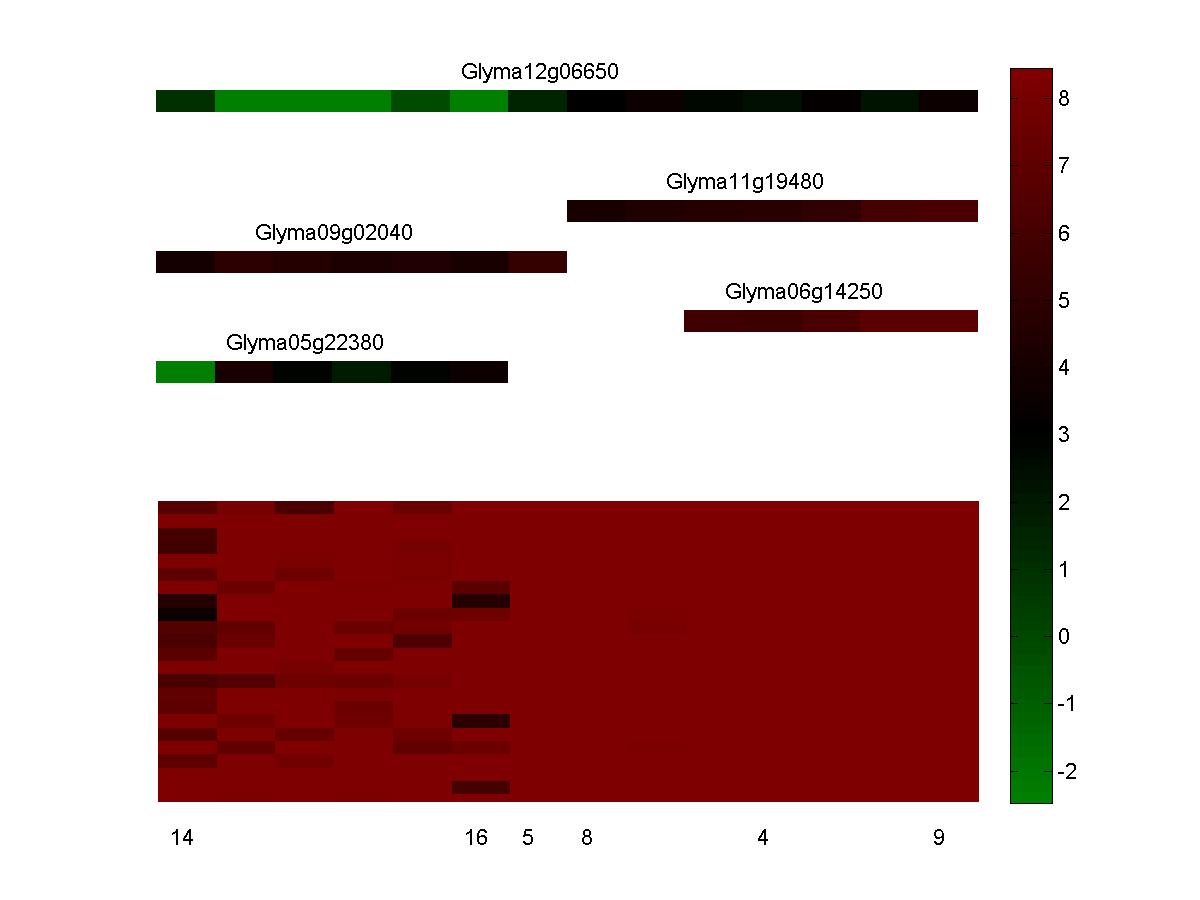


26 Glyma12g06650 GRAS

26 Glyma09g02040 MYB/HD-like

26 Glyma05g22380 BTB/POZ

26 Glyma11g19480 C2H2 (Zn)

26 Glyma06g14250 NAC

Glyma16g04240 Glyma10g39780 Glyma08g29090 Glyma19g37000 Glyma05g11630

Glyma08g11480 Glyma15g19580 Glyma19g29210 Glyma16g04190 Glyma04g01130

Glyma13g40100 Glyma12g04700 Glyma03g32850 Glyma02g04510 Glyma11g12470

Glyma12g04710 Glyma09g08100 Glyma05g28480 Glyma17g23870 Glyma15g21890

Glyma14g06680 Glyma14g40670 Glyma17g35720

27


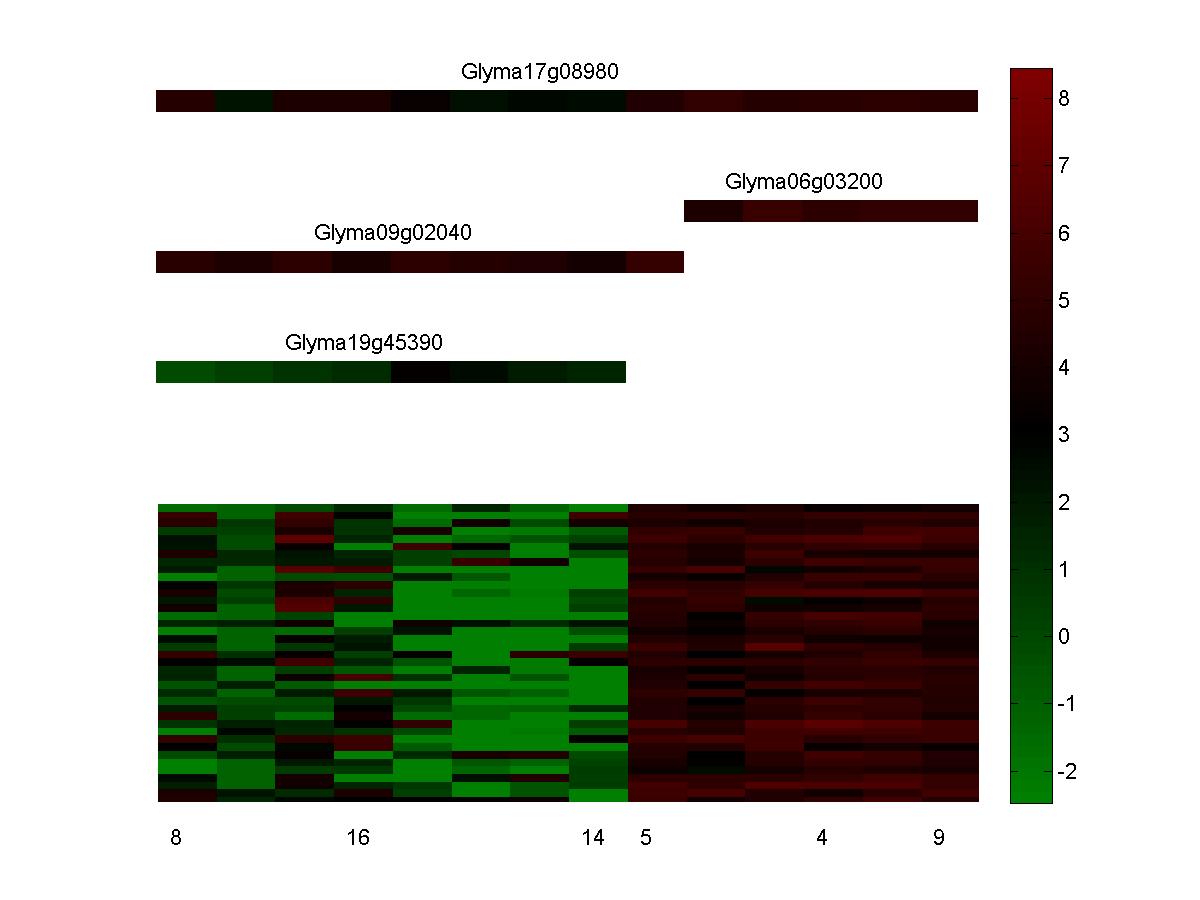


27 Glyma06g03200 Homeodomain/HOMEOBOX

27 Glyma09g02040 MYB/HD-like

27 Glyma19g45390 Homeodomain/HOMEOBOX

27 Glyma17g08980 bHLH

Glyma03g37310 Glyma20g34430 Glyma07g05110 Glyma10g08300 Glyma18g20510

Glyma08g20190 Glyma19g28770 Glyma12g36300 Glyma17g14860 Glyma16g26940

Glyma08g48240 Glyma12g04940 Glyma05g04400 Glyma04g11130 Glyma15g11700

Glyma20g32130 Glyma04g01230 Glyma03g05480 Glyma17g11170 Glyma07g32810

Glyma07g31660 Glyma01g33170 Glyma15g39090 Glyma05g34170 Glyma01g04420

Glyma02g40890 Glyma10g35310 Glyma17g16620 Glyma06g05530 Glyma13g27010

Glyma03g02580 Glyma11g14060 Glyma10g38080 Glyma09g08470 Glyma07g15690

Glyma11g32600 Glyma17g33470 Glyma14g00850 Glyma10g01300

28


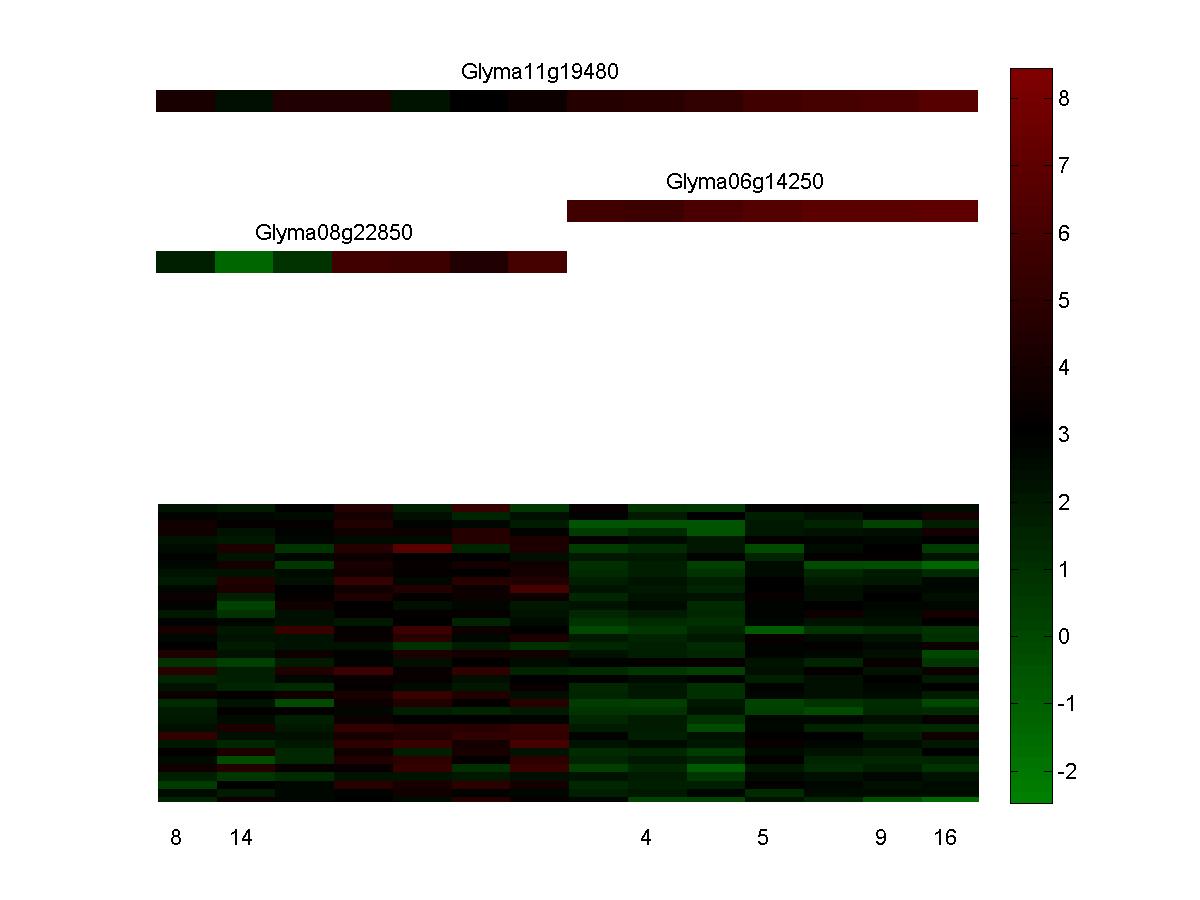


28 Glyma08g22850 TPR

28 Glyma11g19480 C2H2 (Zn)

28 Glyma06g14250 NAC

Glyma01g01320 Glyma11g15840 Glyma13g44790 Glyma05g06570 Glyma13g31460

Glyma07g08010 Glyma07g03820 Glyma06g17100 Glyma19g39330 Glyma15g00730

Glyma11g38040 Glyma04g34880 Glyma11g05980 Glyma13g44440 Glyma06g48170

Glyma13g01310 Glyma03g35870 Glyma04g33930 Glyma02g37370 Glyma18g53370

Glyma17g21540 Glyma15g04350 Glyma05g03010 Glyma13g23310 Glyma04g00280

Glyma10g06630 Glyma08g25830 Glyma01g28500 Glyma19g35710 Glyma05g23860

Glyma09g19790 Glyma08g43950 Glyma02g13850 Glyma06g17550 Glyma07g32620

Glyma02g17410 Glyma19g22460

29


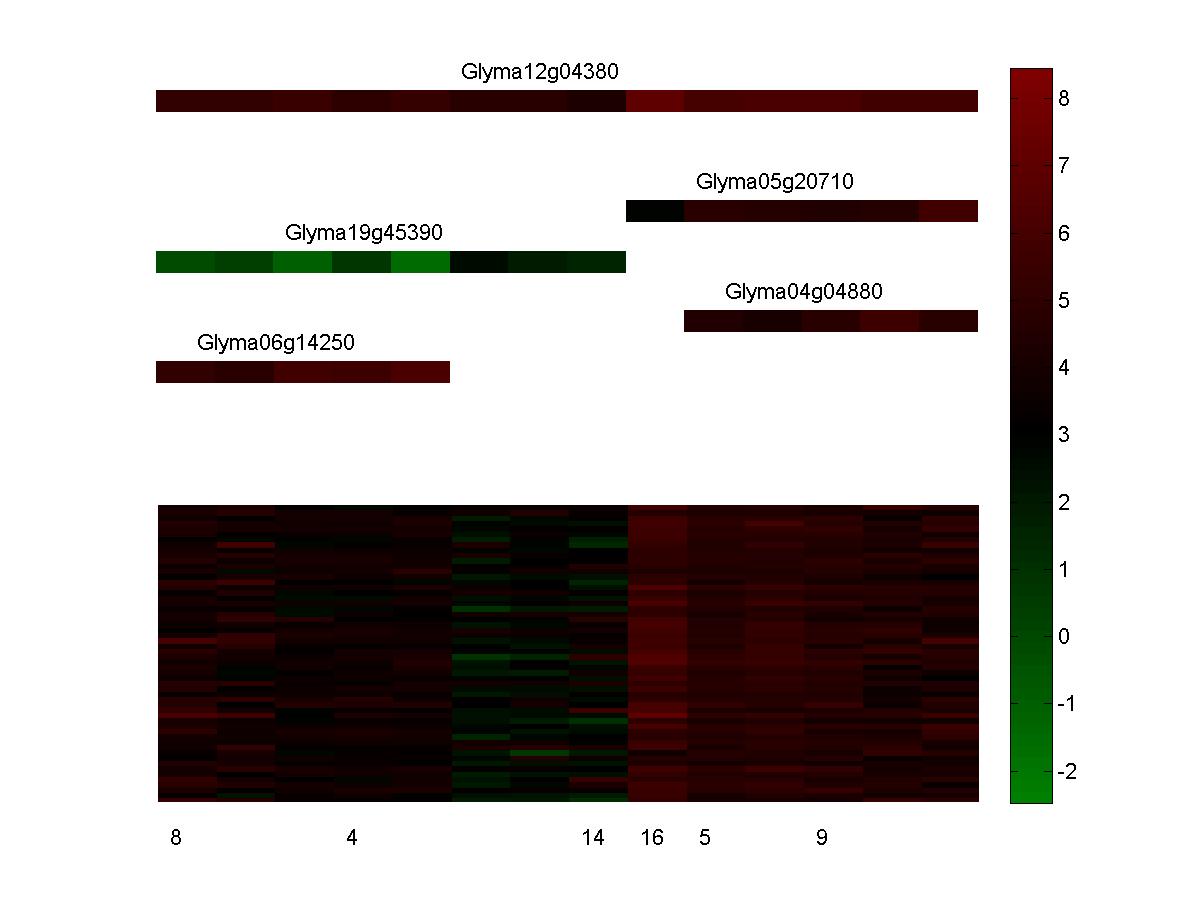


29 Glyma19g45390 Homeodomain/HOMEOBOX

29 Glyma12g04380 NAC

29 Glyma04g04880 LIM

29 Glyma06g14250 NAC

29 Glyma05g20710 WRKY

Glyma19g33700 Glyma20g26500 Glyma19g30860 Glyma08g08030 Glyma09g37570

Glyma13g44090 Glyma11g00590 Glyma01g38520 Glyma05g27690 Glyma17g06340

Glyma12g07130 Glyma08g46190 Glyma06g23580 Glyma17g10340 Glyma15g16630

Glyma19g30550 Glyma13g44430 Glyma01g41210 Glyma03g30720 Glyma19g34910

Glyma13g02870 Glyma09g36740 Glyma06g13260 Glyma17g06540 Glyma05g07840

Glyma08g07890 Glyma02g03950 Glyma04g12320 Glyma20g02110 Glyma17g15100

Glyma07g13290 Glyma01g31270 Glyma08g06950 Glyma08g2045 Glyma05g28880

Glyma01g39770 Glyma01g01380 Glyma08g48290 Glyma11g02130 Glyma11g25650

Glyma17g03420 Glyma03g36580 Glyma08g18350 Glyma17g08700 Glyma13g35400

Glyma13g40380 Glyma02g41220 Glyma12g17150 Glyma02g02180 Glyma10g04590

Glyma12g15800 Glyma11g21140 Glyma20g04230 Glyma13g35580 Glyma20g29980

Glyma04g38260

30


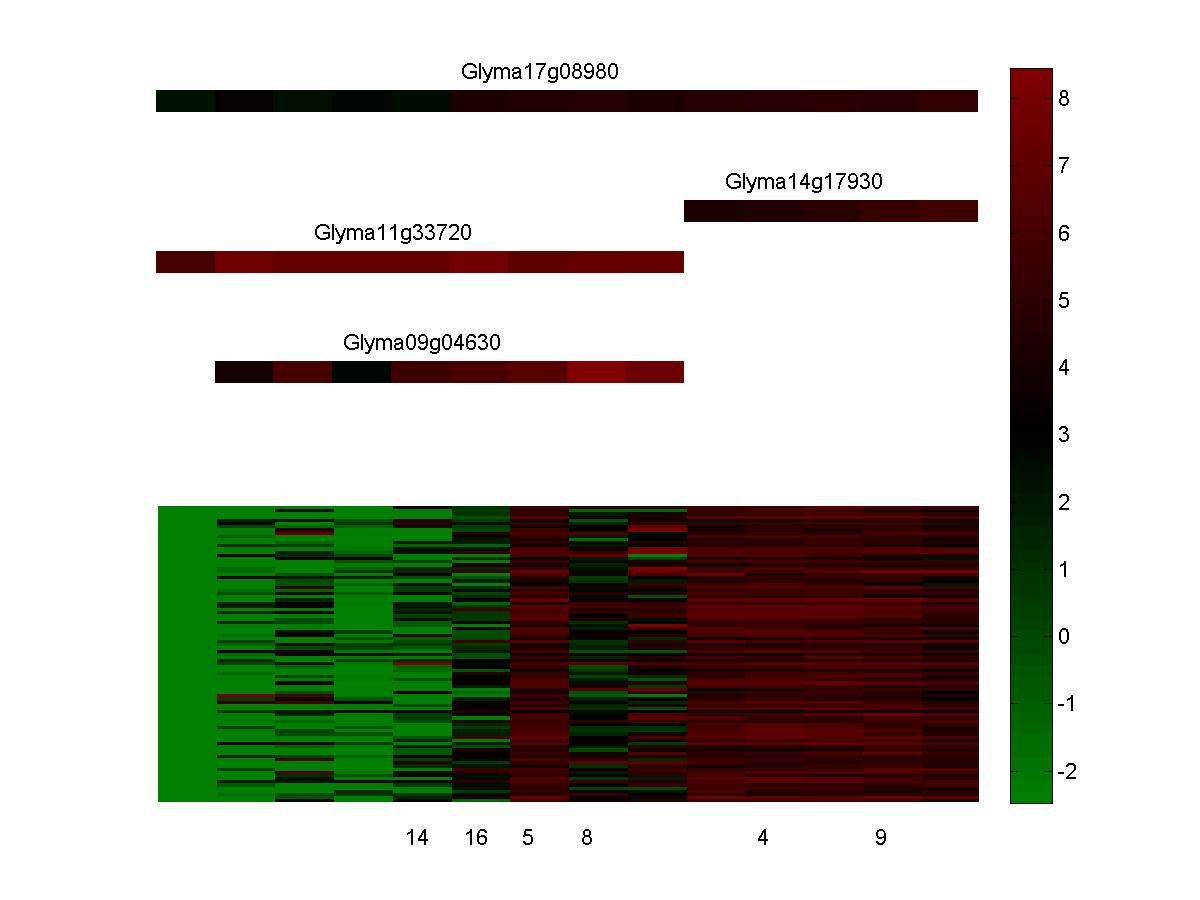


30 Glyma09g04630 AP2-EREBP

30 Glyma11g33720 GRAS

30 Glyma14g17930 CCHC (Zn)

30 Glyma17g08980 bHLH

Glyma06g18560 Glyma19g26830 Glyma09g16930 Glyma05g30290 Glyma19g28520

Glyma16g04720 Glyma03g24060 Glyma02g09210 Glyma16g04960 Glyma09g08490

Glyma16g31280 Glyma08g46620 Glyma03g29190 Glyma12g00970 Glyma03g30420

Glyma06g15410 Glyma08g12470 Glyma09g09400 Glyma06g37930 Glyma10g00950

Glyma12g04880 Glyma06g05370 Glyma04g37040 Glyma01g28510 Glyma08g19180

Glyma03g15800 Glyma06g40620 Glyma09g40590 Glyma10g07410 Glyma15g14210

Glyma11g27480 Glyma01g33440 Glyma18g12180 Glyma01g33450 Glyma12g02040

Glyma07g37250 Glyma09g03450 Glyma02g00840 Glyma10g02730 Glyma16g28610

Glyma11g27720 Glyma20g24720 Glyma20g38590 Glyma13g23760 Glyma10g08010

Glyma06g01270 Glyma06g47470 Glyma03g36620 Glyma14g11670 Glyma09g09430

Glyma13g01900 Glyma12g00980 Glyma10g08210 Glyma15g35410 Glyma04g12600

Glyma03g37410 Glyma07g37240 Glyma16g07830 Glyma04g05510 Glyma14g40200

Glyma16g04980 Glyma20g11610 Glyma20g01370 Glyma11g06070 Glyma15g24760

Glyma15g07700 Glyma16g06520 Glyma01g42230 Glyma11g04970 Glyma16g22920

Glyma06g47190 Glyma17g12160 Glyma18g53740 Glyma12g34310 Glyma12g06300

Glyma08g20220 Glyma12g35370 Glyma15g13870 Glyma02g37320 Glyma02g16710

Glyma08g04380 Glyma08g19240 Glyma02g47750 Glyma06g12010 Glyma07g34010

Glyma16g05710 Glyma15g04930 Glyma07g17170 Glyma11g02640 Glyma03g37390

Glyma13g32310 Glyma17g15690 Glyma14g36700

31


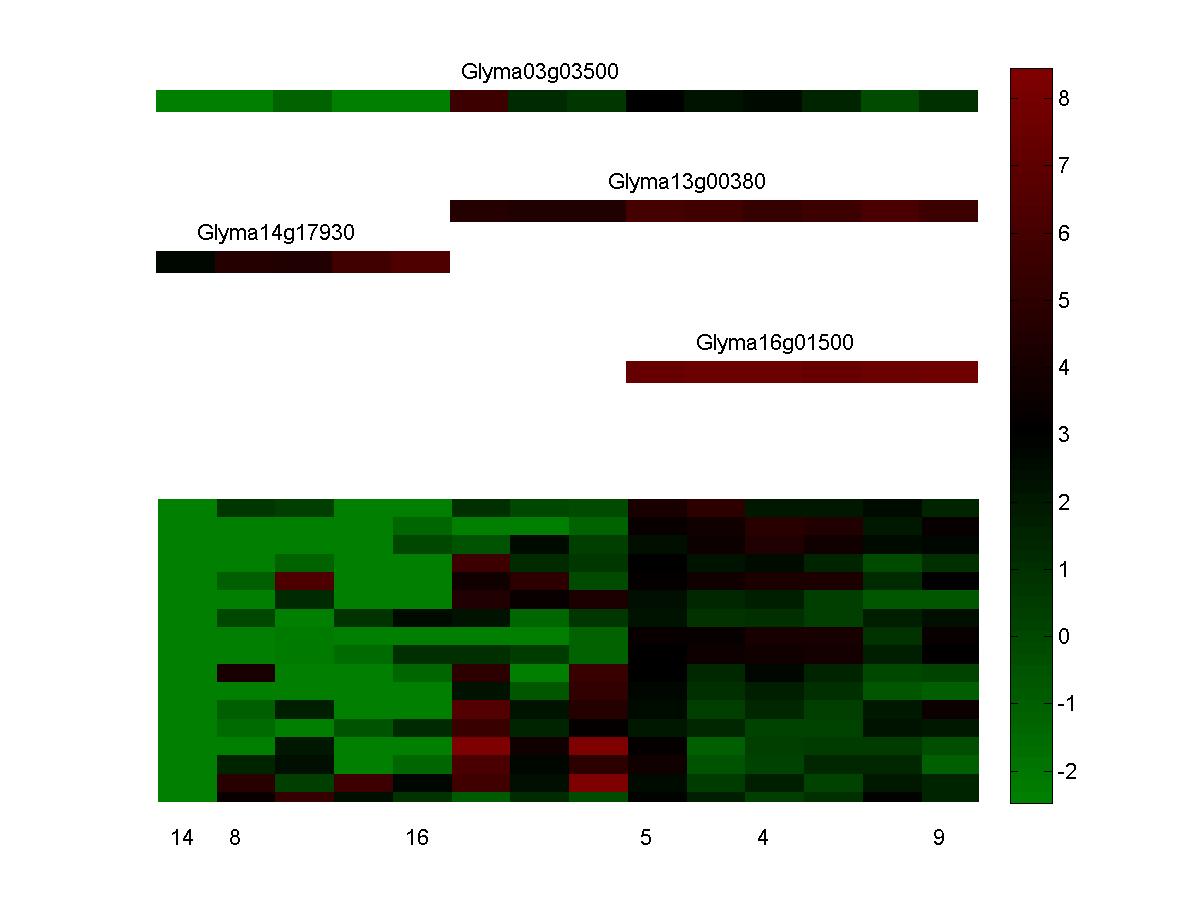


31 Glyma03g03500 C2C2 (Zn) YABBY

31 Glyma14g17930 CCHC (Zn)

31 Glyma16g01500 AP2-EREBP

31 Glyma13g00380 WRKY

Glyma08g09450 Glyma07g37260 Glyma01g02580 Glyma03g03500 Glyma07g09840

Glyma09g24450 Glyma18g02880 Glyma19g38000 Glyma06g02540 Glyma09g33480

Glyma19g42940 Glyma15g38220 Glyma11g37680 Glyma02g03250 Glyma07g13780

Glyma03g34750 Glyma06g47560

32


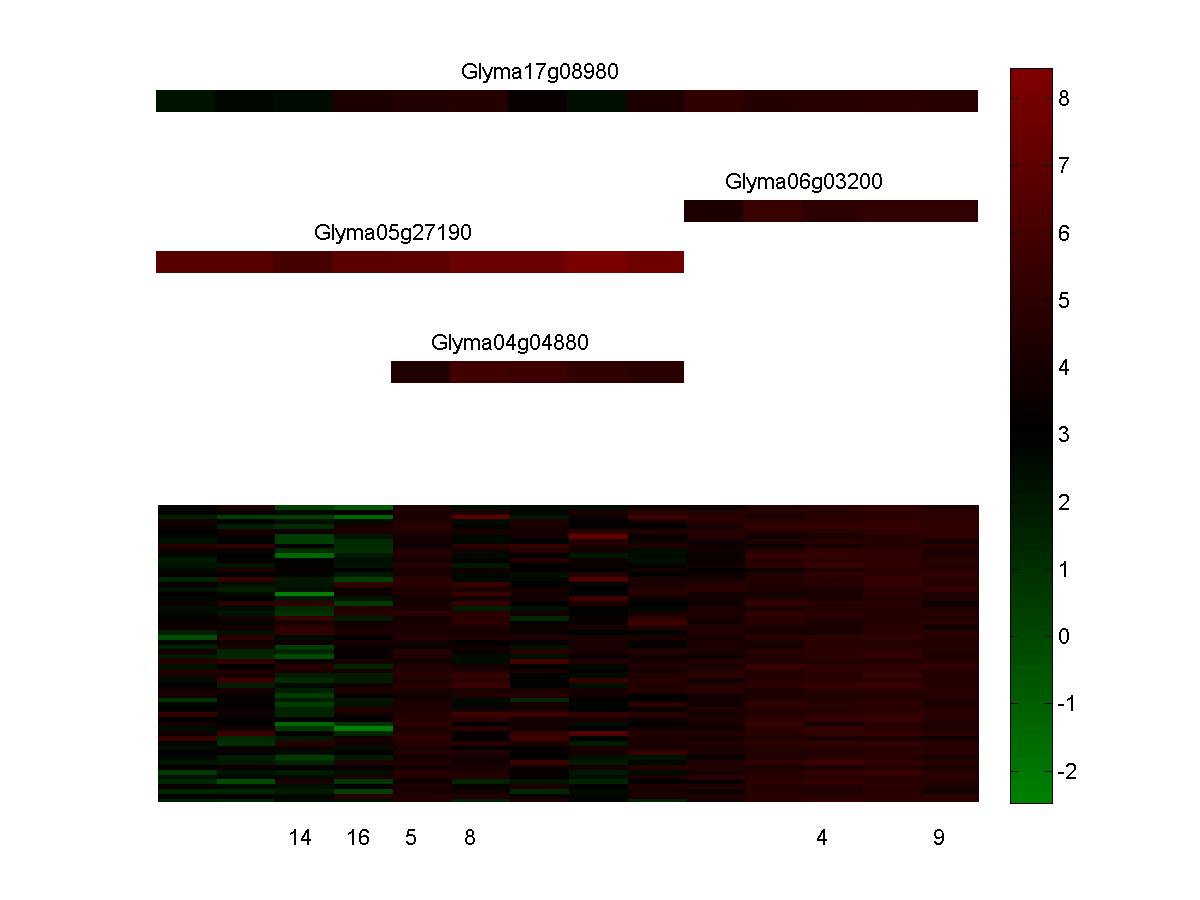


32 Glyma06g03200 Homeodomain/HOMEOBOX

32 Glyma05g27190 GRAS

32 Glyma17g08980 bHLH

32 Glyma04g04880 LIM

Glyma06g10450 Glyma08g19270 Glyma12g09810 Glyma11g00910 Glyma13g37280

Glyma08g25410 Glyma17g07530 Glyma04g08010 Glyma18g01890 Glyma02g14140

Glyma02g00830 Glyma09g32790 Glyma13g05830 Glyma13g44390 Glyma06g20360

Glyma11g02980 Glyma02g01910 Glyma08g09730 Glyma02g01250 Glyma05g28120

Glyma15g2282 Glyma02g46580 Glyma03g31870 Glyma05g36700 Glyma04g40030

Glyma15g19000 Glyma10g43060 Glyma11g15650 Glyma13g19080 Glyma06g16440

Glyma10g05130 Glyma03g04330 Glyma15g09900 Glyma06g03200 Glyma18g00610

Glyma11g36230 Glyma03g34480 Glyma04g43070 Glyma06g09880 Glyma02g37510

Glyma16g27380 Glyma08g14980 Glyma09g02860 Glyma04g04880 Glyma19g32550

Glyma03g34800 Glyma01g04890 Glyma05g34530 Glyma10g30570 Glyma06g45180

Glyma08g07660 Glyma02g42830 Glyma07g34800 Glyma07g01220 Glyma05g30180

Glyma20g02500 Glyma17g08980 Glyma06g41510 Glyma17g0443 Glyma08g07470

Glyma07g15160 Glyma02g06160

33


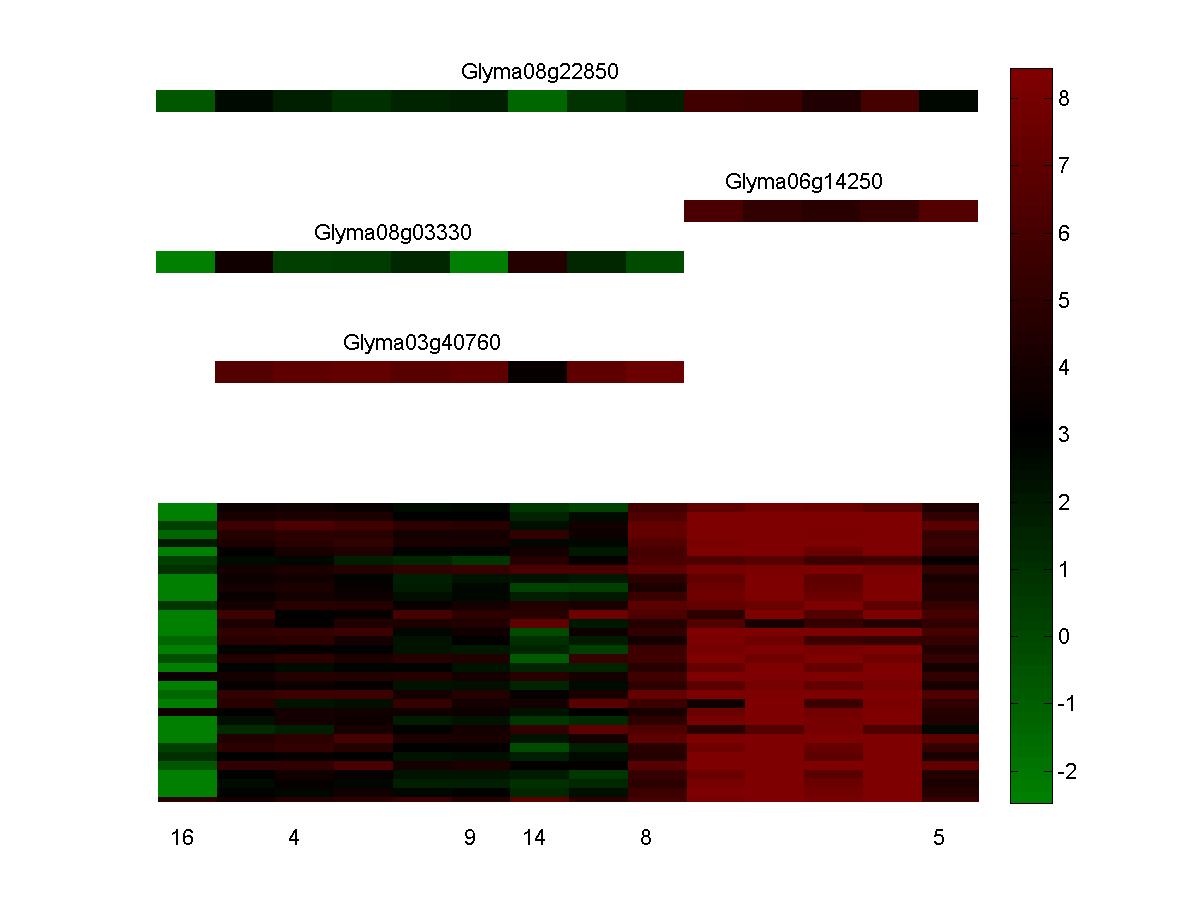


33 Glyma06g14250 NAC

33 Glyma03g40760 AUX-IAA-ARF

33 Glyma08g22850 TPR

33 Glyma08g03330 MYB/HD-like

Glyma0048s00300 Glyma01g28810 Glyma05g00620 Glyma03g42310 Glyma20g28300

Glyma03g26740 Glyma13g23090 Glyma15g03050 Glyma02g16000 Glyma06g02300

Glyma01g38750 Glyma07g00890 Glyma08g43330 Glyma18g42580 Glyma14g01130

Glyma17g10010 Glyma04g05800 Glyma10g34460 Glyma04g03820 Glyma15g41540

Glyma06g05810 Glyma10g39460 Glyma08g43340 Glyma08g19210 Glyma03g08280

Glyma04g08410 Glyma05g25810 Glyma04g02240 Glyma07g06660 Glyma08g08770

Glyma02g45190 Glyma11g18640 Glyma16g25860 Glyma08g17610

34


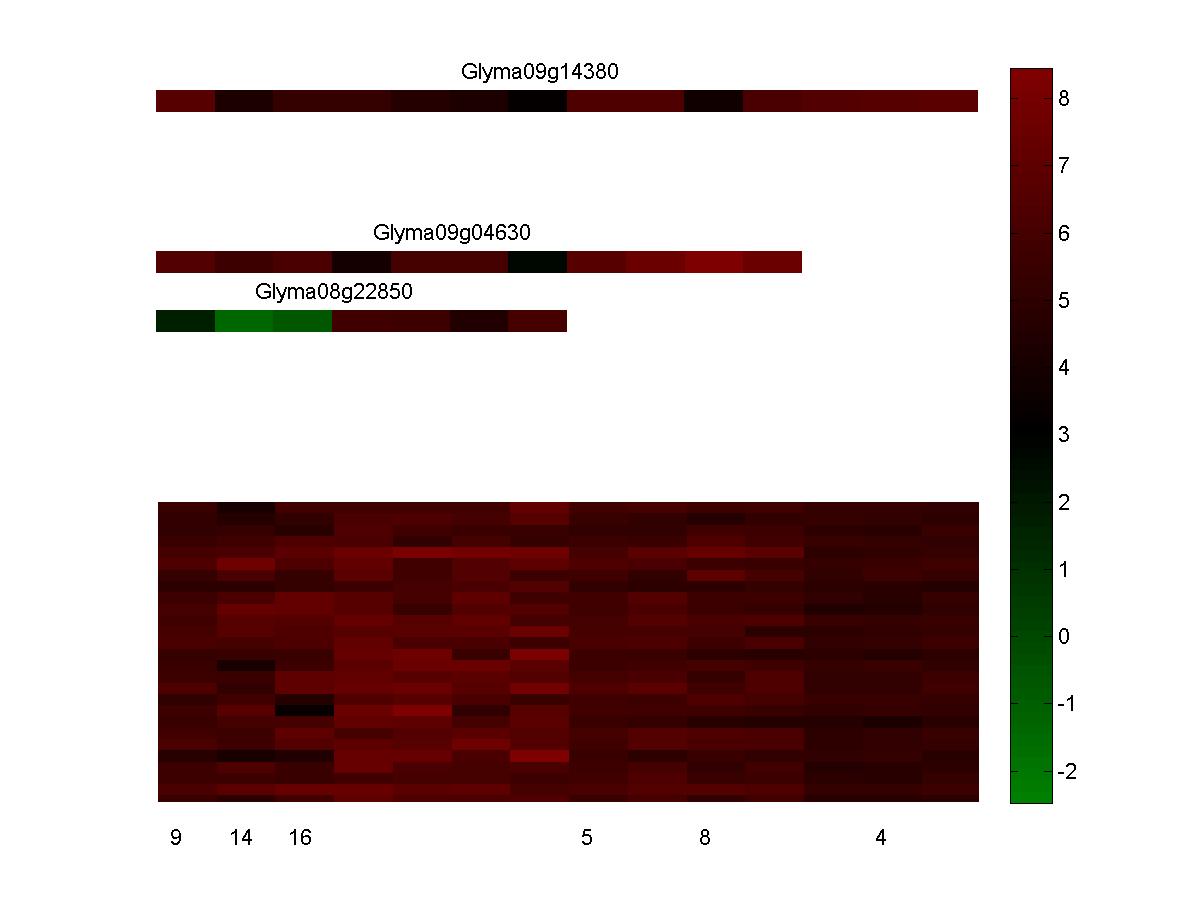


34 Glyma08g22850 TPR

34 Glyma09g04630 AP2-EREBP

34 Glyma09g14380 bHLH

Glyma02g00320 Glyma08g23940 Glyma11g13500 Glyma06g07420 Glyma19g22730

Glyma12g08310 Glyma15g06210 Glyma10g02240 Glyma06g33940 Glyma15g40110

Glyma12g19050 Glyma16g00410 Glyma09g07120 Glyma10g06620 Glyma17g18800

Glyma18g06560 Glyma20g39340 Glyma04g12240 Glyma14g05980 Glyma08g13100

Glyma08g23970 Glyma17g00710 Glyma11g10550 Glyma19g34300 Glyma19g28430

Glyma05g36140 Glyma09g35980

35


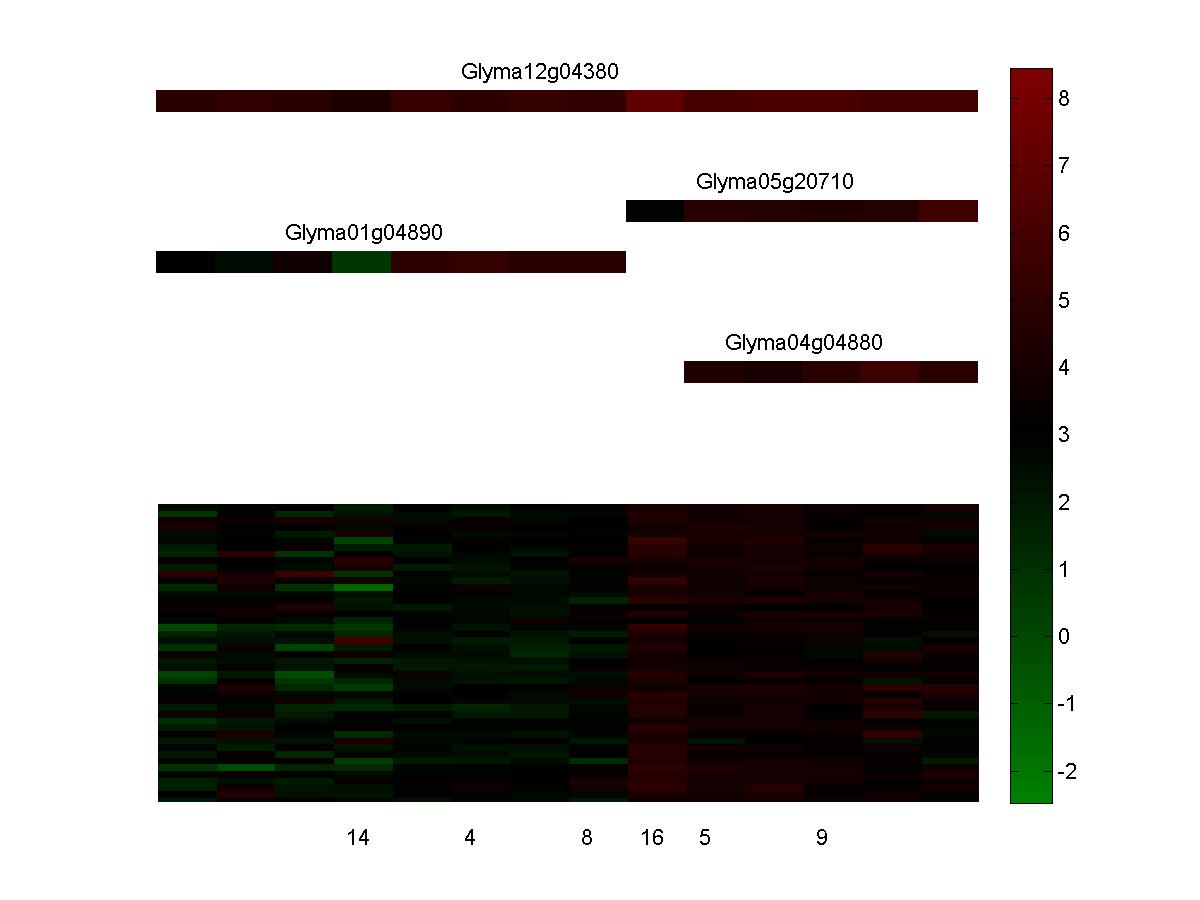


35 Glyma12g04380 NAC

35 Glyma05g20710 WRKY

35 Glyma01g04890 Homeodomain/HOMEOBOX

35 Glyma04g04880 LIM

Glyma17g10770 Glyma06g09060 Glyma20g02760 Glyma07g12190 Glyma02g17440

Glyma11g38010 Glyma05g31290 Glyma04g42930 Glyma18g29500 Glyma11g06440

Glyma03g39570 Glyma16g04090 Glyma13g35750 Glyma03g41400 Glyma05g02340

Glyma09g41100 Glyma15g01270 Glyma06g09560 Glyma02g10550 Glyma17g13230

Glyma02g42130 Glyma02g01560 Glyma19g04910 Glyma03g01310 Glyma02g42270

Glyma18g22780 Glyma13g41450 Glyma02g41230 Glyma06g17490 Glyma03g35450

Glyma11g00600 Glyma10g01720 Glyma11g12540 Glyma17g05270 Glyma06g46540

Glyma10g03920 Glyma08g14450 Glyma09g40560 Glyma10g37980 Glyma12g06770

Glyma10g33010 Glyma14g04360 Glyma10g26790 Glyma17g00230 Glyma09g40370

36


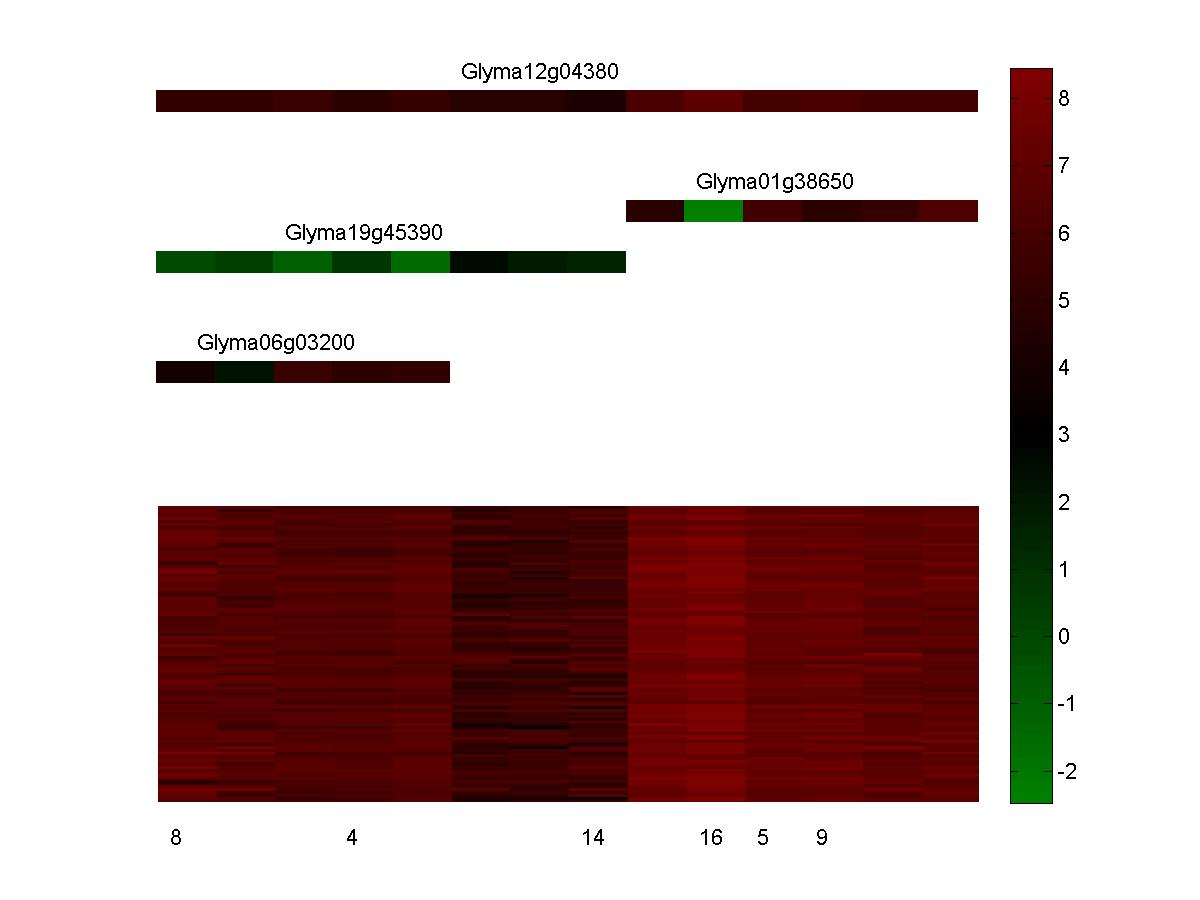


36 Glyma12g04380 NAC

36 Glyma01g38650 Homeodomain/HOMEOBOX

36 Glyma06g03200 Homeodomain/HOMEOBOX

36 Glyma19g45390 Homeodomain/HOMEOBOX

Glyma16g00360 Glyma06g17930 Glyma17g11130 Glyma08g13970 Glyma15g15800

Glyma02g15910 Glyma04g32950 Glyma10g07680 Glyma10g36610 Glyma12g13240

Glyma07g13710 Glyma01g43110 Glyma02g42080 Glyma02g43260 Glyma08g15000

Glyma07g15320 Glyma06g06790 Glyma08g19320 Glyma06g14080 Glyma10g32580

Glyma16g03760 Glyma08g23750 Glyma04g40720 Glyma02g10170 Glyma01g00740

Glyma13g41960 Glyma14g36620 Glyma07g02270 Glyma17g02200 Glyma19g35690

Glyma20g28780 Glyma20g21230 Glyma19g06460 Glyma05g00780 Glyma11g25910

Glyma08g21960 Glyma05g04870 Glyma01g03180 Glyma09g38590 Glyma14g40320

Glyma17g10710 Glyma03g26100 Glyma17g08630 Glyma02g04400 Glyma03g32980

Glyma14g0153 Glyma02g00810 Glyma07g13900 Glyma13g20010 Glyma08g03480

Glyma01g03570 Glyma03g37340 Glyma13g16500 Glyma12g03070 Glyma10g03840

Glyma08g45710 Glyma16g17370 Glyma10g38680 Glyma11g08050 Glyma19g37370

Glyma11g20570 Glyma05g26320 Glyma10g36500 Glyma01g4162 Glyma08g17600

Glyma02g00540 Glyma05g30780 Glyma09g37850 Glyma10g00920 Glyma02g44330

Glyma08g46070 Glyma05g02570 Glyma18g48620 Glyma02g04090 Glyma15g07420

Glyma10g29170 Glyma11g03570 Glyma09g05720 Glyma10g30200 Glyma08g16130

Glyma04g00660 Glyma02g43080 Glyma14g09300 Glyma04g00650 Glyma02g42260

Glyma09g36560 Glyma05g03880 Glyma05g21820 Glyma12g35990 Glyma04g40430

Glyma17g15230 Glyma13g43450 Glyma02g07430 Glyma10g43770 Glyma13g33410

Glyma19g37520 Glyma06g18800 Glyma11g10790 Glyma05g04670 Glyma11g02410

Glyma07g33300 Glyma03g25820 Glyma08g03410 Glyma11g11020 Glyma19g40080

37


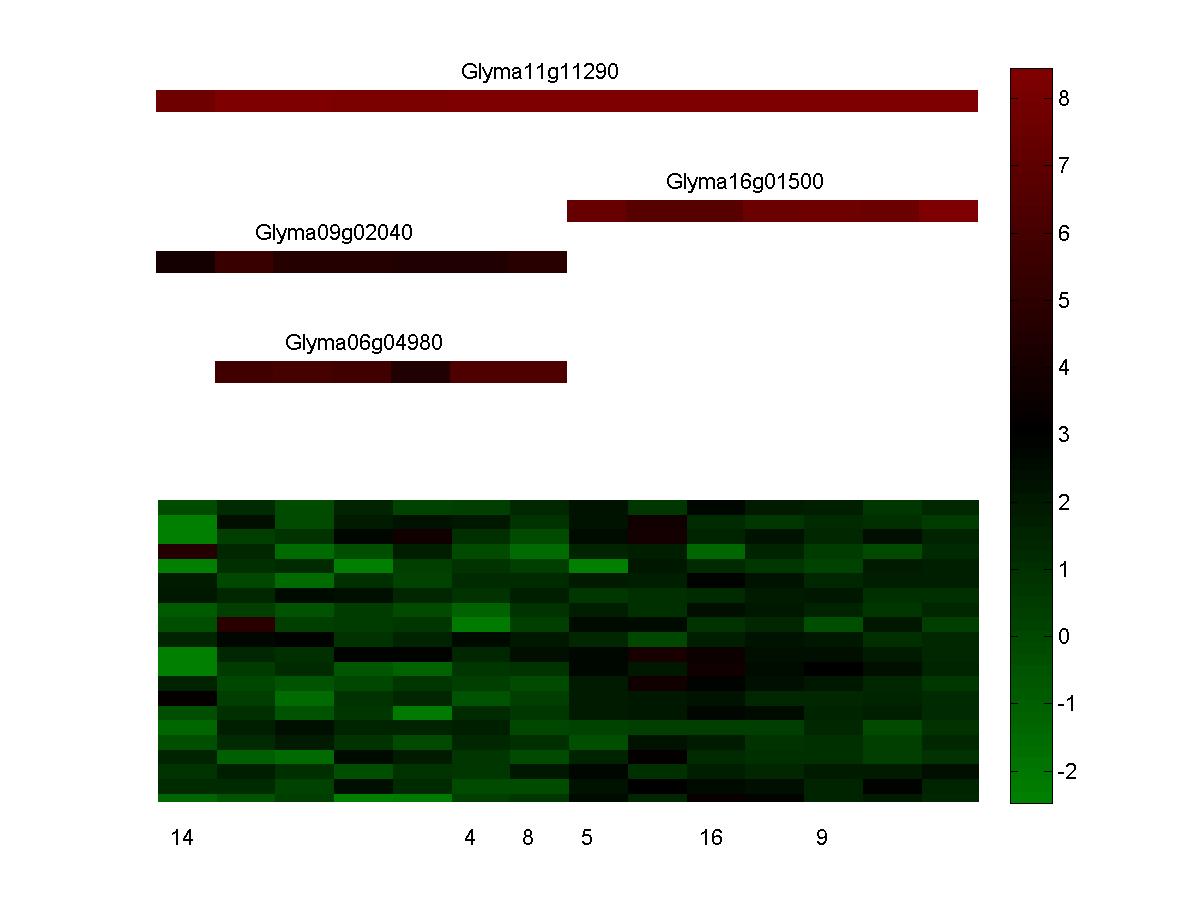


37 Glyma06g04980 LIM

37 Glyma16g01500 AP2-EREBP

37 Glyma09g02040 MYB/HD-like

37 Glyma11g11290 CSD

Glyma13g30820 Glyma11g18900 Glyma06g34330 Glyma04g07070 Glyma17g01920

Glyma07g02600 Glyma03g22130 Glyma06g10570 Glyma08g17200 Glyma08g05220

Glyma05g22380 Glyma12g31990 Glyma02g07730 Glyma17g15300 Glyma04g00380

Glyma06g46750 Glyma06g46810 Glyma19g45390 Glyma09g20040 Glyma01g39410

Glyma13g38490

38


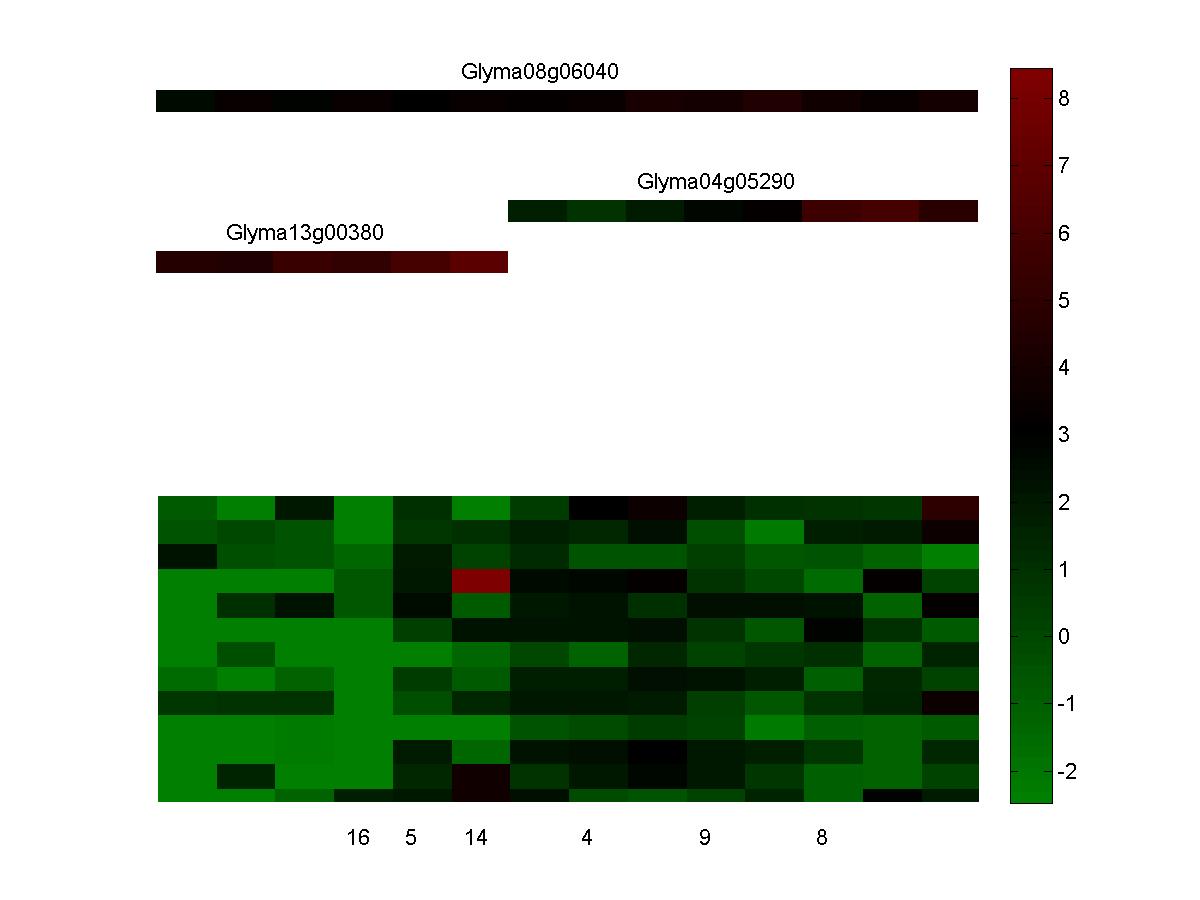


38 Glyma08g06040 PHD

38 Glyma13g00380 WRKY

38 Glyma04g05290 C3H-type1(Zn)

Glyma06g07780 Glyma02g41660 Glyma02g43280 Glyma02g36700 Glyma11g18680

Glyma05g33340 Glyma11g19980 Glyma06g20370 Glyma14g07290 Glyma12g34560

Glyma12g05180 Glyma13g31580 Glyma15g06140

39


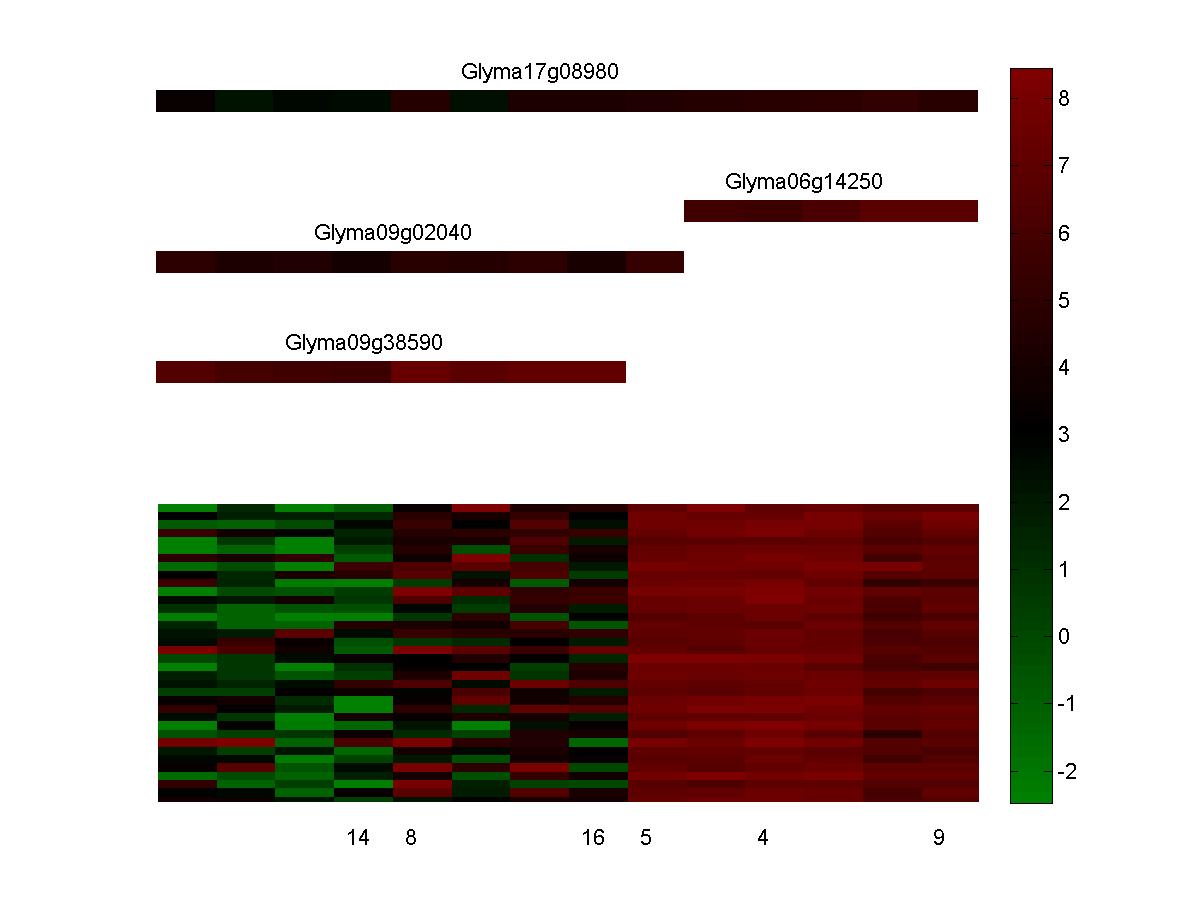


39 Glyma09g02040 MYB/HD-like

39 Glyma09g38590 C2H2 (Zn)

39 Glyma17g08980 bHLH

39 Glyma06g14250 NAC

Glyma13g20170 Glyma01g02330 Glyma10g33650 Glyma13g19500 Glyma11g02770

Glyma11g12790 Glyma09g22310 Glyma14g36690 Glyma07g05480 Glyma13g39890

Glyma01g42670 Glyma15g42780 Glyma16g33270 Glyma13g27130 Glyma03g02410

Glyma14g35340 Glyma15g38060 Glyma09g37290 Glyma15g06790 Glyma02g04820

Glyma08g08380 Glyma15g17530 Glyma03g19260 Glyma15g37520 Glyma06g08910

Glyma06g36520 Glyma17g08900 Glyma16g01960 Glyma02g18090 Glyma10g37200

Glyma11g14130 Glyma19g33330 Glyma18g41910 Glyma03g03270 Glyma02g08950

Glyma05g32160

40


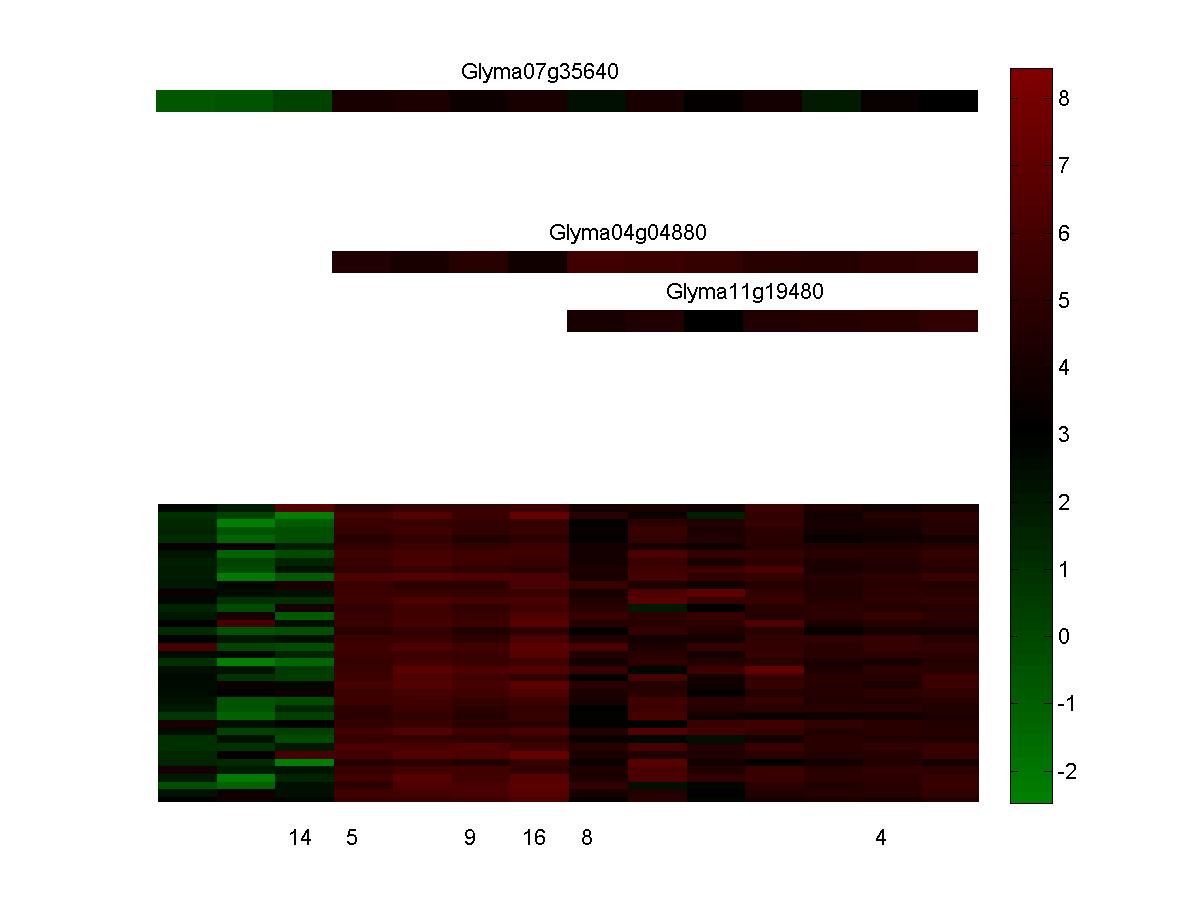


40 Glyma07g35640 TPR

40 Glyma11g19480 C2H2 (Zn)

40 Glyma04g04880 LIM

Glyma16g09020 Glyma15g15990 Glyma15g34870 Glyma18g07150 Glyma13g41710

Glyma10g02050 Glyma19g33360 Glyma11g24400 Glyma09g38320 Glyma12g05910

Glyma08g04240 Glyma15g01160 Glyma03g30440 Glyma18g03950 Glyma01g39810

Glyma19g05980 Glyma13g41700 Glyma04g40140 Glyma19g28220 Glyma18g51660

Glyma07g32360 Glyma12g32160 Glyma12g07930 Glyma18g52310 Glyma08g43690

Glyma08g26880 Glyma11g15500 Glyma08g02550 Glyma05g36320 Glyma13g24180

Glyma06g37940 Glyma13g39590 Glyma10g33680 Glyma10g30650 Glyma11g00670

Glyma02g05480 Glyma04g04400 Glyma11g19480 Glyma11g01660

41


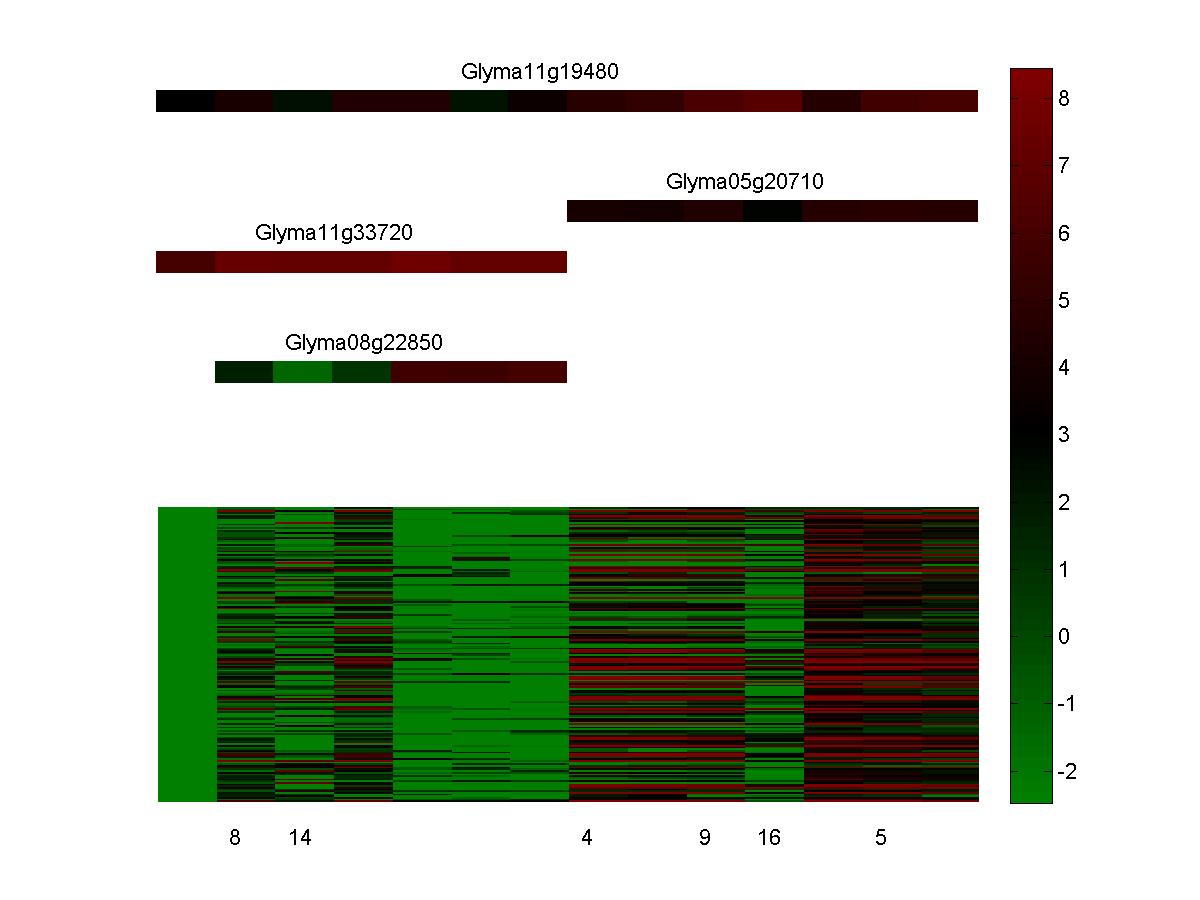


41 Glyma11g33720 GRAS

41 Glyma11g19480 C2H2 (Zn)

41 Glyma05g20710 WRKY

41 Glyma08g22850 TPR

Glyma02g02800 Glyma06g18660 Glyma08g24770 Glyma13g00370 Glyma03g31980

Glyma02g01970 Glyma11g20710 Glyma10g04150 Glyma08g43240 Glyma08g08170

Glyma10g03000 Glyma09g24130 Glyma18g09290 Glyma20g01420 Glyma01g45280

Glyma16g01020 Glyma13g22350 Glyma12g09240 Glyma20g30450 Glyma18g02870

Glyma09g05290 Glyma06g29660 Glyma19g40960 Glyma19g43370 Glyma16g04410

Glyma19g28760 Glyma19g45260 Glyma17g14260 Glyma11g29920 Glyma06g42040

Glyma08g19250 Glyma15g06780 Glyma05g12090 Glyma09g31910 Glyma08g09460

Glyma12g03050 Glyma02g08400 Glyma11g12650 Glyma15g13550 Glyma01g34570

Glyma12g05310 Glyma17g04420 Glyma11g03000 Glyma18g02230 Glyma07g28610

Glyma09g05450 Glyma02g40200 Glyma20g34830 Glyma10g42840 Glyma14g05760

Glyma04g00210 Glyma18g06230 Glyma05g12100 Glyma12g16410 Glyma17g02260

Glyma08g17270 Glyma08g03330 Glyma11g34380 Glyma15g36740 Glyma12g06650

Glyma07g04340 Glyma07g32590 Glyma06g12510 Glyma18g06350 Glyma03g34440

Glyma18g02100 Glyma05g25450 Glyma07g08280 Glyma20g29210 Glyma07g38620

Glyma11g03940 Glyma03g08740 Glyma03g01190 Glyma10g25120 Glyma09g02610

Glyma11g22090 Glyma20g01430 Glyma14g35720 Glyma03g35950 Glyma15g15200

Glyma07g04430 Glyma19g37630 Glyma09g36620 Glyma17g08110 Glyma13g21570

Glyma11g35560 Glyma08g43550 Glyma04g12030 Glyma10g32070 Glyma07g04320

Glyma03g03460 Glyma11g10760 Glyma08g24760 Glyma09g28800 Glyma06g03390

Glyma03g04880 Glyma15g02380 Glyma07g35240 Glyma13g27300 Glyma01g39470

Glyma17g07440 Glyma01g42370 Glyma09g32630 Glyma09g27600 Glyma08g24720

Glyma20g38150 Glyma11g03310 Glyma02g40010 Glyma06g19620 Glyma12g30050

Glyma18g16790 Glyma02g09540 Glyma02g36580 Glyma15g35390 Glyma04g35130

Glyma01g34770 Glyma18g02090 Glyma05g03720 Glyma08g05850 Glyma01g42800

Glyma07g37270 Glyma01g05050 Glyma13g25560 Glyma10g40350 Glyma04g05230

Glyma06g47690 Glyma13g39070 Glyma04g13990 Glyma01g38040 Glyma02g16800

Glyma13g24560 Glyma16g04360 Glyma04g42300 Glyma10g38360 Glyma08g21190

Glyma09g00800 Glyma18g39500 Glyma10g29150 Glyma15g16710 Glyma10g31280

Glyma16g06500 Glyma03g30260 Glyma10g02090 Glyma03g40910 Glyma10g25130

Glyma10g38600 Glyma05g25460 Glyma01g37820 Glyma15g05760 Glyma07g00870

Glyma12g35710 Glyma12g02240 Glyma15g36290 Glyma05g36290 Glyma05g00990

Glyma08g04370 Glyma15g11220 Glyma10g10240 Glyma11g10080 Glyma13g23770

Glyma05g02490 Glyma17g02080 Glyma02g48080 Glyma16g11370 Glyma08g24680

Glyma03g34560 Glyma09g00850 Glyma04g02230 Glyma07g03120 Glyma06g07160

Glyma03g37400 Glyma08g22630 Glyma14g06900 Glyma10g44170 Glyma06g06490

Glyma09g31110

42


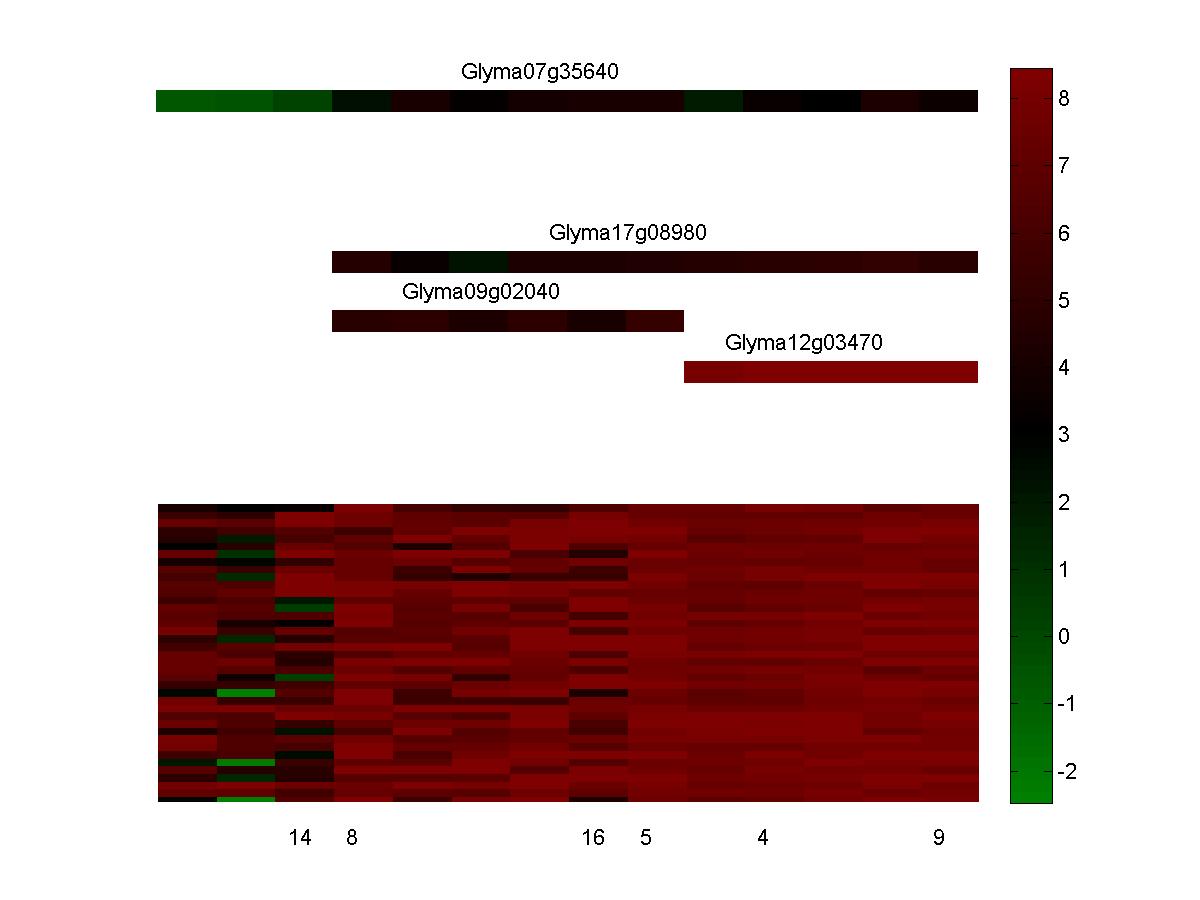


42 Glyma09g02040 MYB/HD-like

42 Glyma17g08980 bHLH

42 Glyma07g35640 TPR

42 Glyma12g03470 CSD

Glyma13g01870 Glyma04g00960 Glyma09g04480 Glyma11g05680 Glyma04g09350

Glyma16g04630 Glyma15g03040 Glyma20g29840 Glyma20g05560 Glyma20g28320

Glyma06g02330 Glyma12g06910 Glyma06g02650 Glyma13g22940 Glyma13g44170

Glyma16g04950 Glyma11g01520 Glyma09g28320 Glyma02g43270 Glyma02g37080

Glyma19g30770 Glyma12g1016 Glyma18g49400 Glyma14g36850 Glyma10g30110

Glyma18g00590 Glyma05g31610 Glyma18g35300 Glyma02g40290 Glyma11g36620

Glyma06g04760 Glyma01g01180 Glyma10g35520 Glyma08g13440 Glyma08g14550

Glyma09g28310 Glyma18g01860 Glyma0169s00210 Glyma10g30120

43


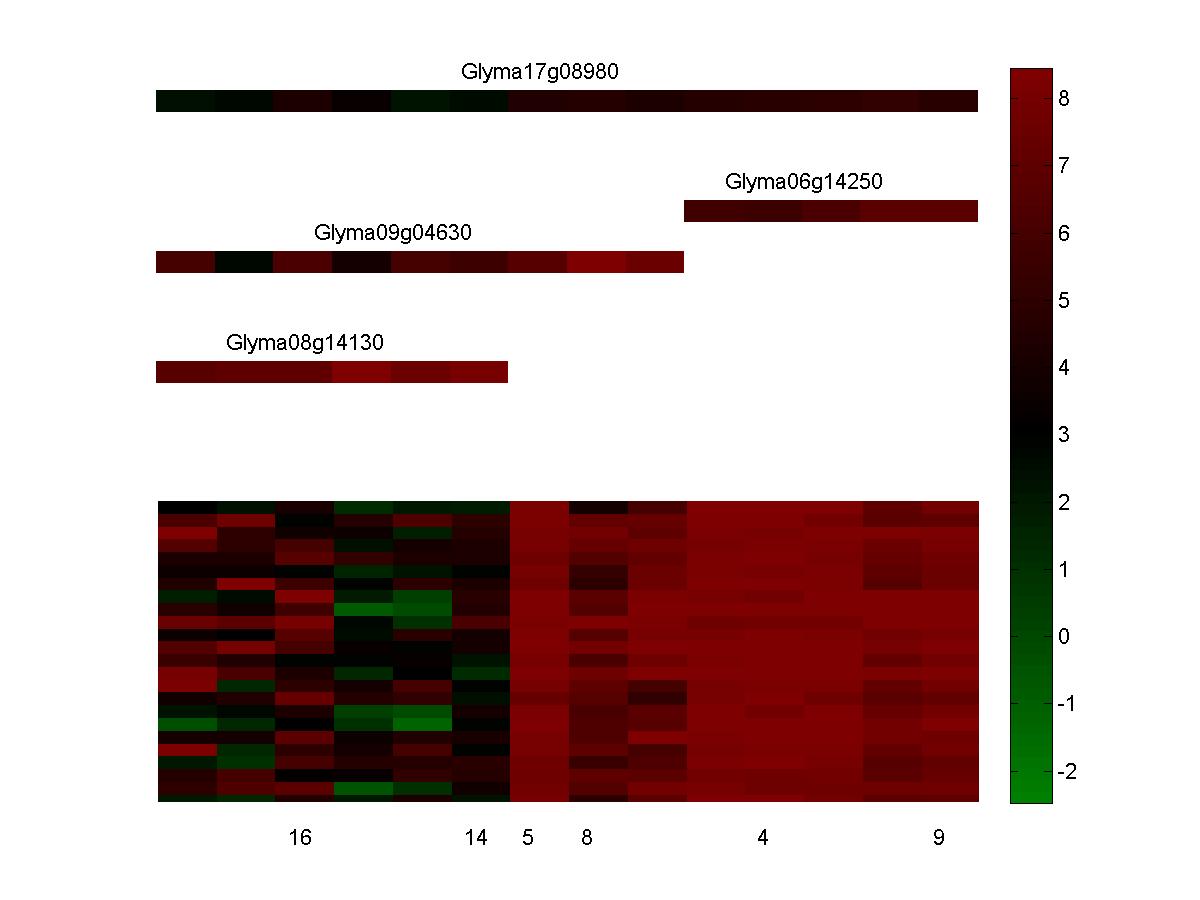


43 Glyma06g14250 NAC

43 Glyma09g04630 AP2-EREBP

43 Glyma17g08980 bHLH

43 Glyma08g14130 Homeodomain/HOMEOBO

Glyma11g07490 Glyma04g40810 Glyma09g07100 Glyma10g38070 Glyma04g34840

Glyma09g28490 Glyma17g03910 Glyma18g52250 Glyma20g35630 Glyma05g22180

Glyma10g44360 Glyma11g00230 Glyma16g28590 Glyma02g09220 Glyma13g34520

Glyma03g30110 Glyma17g03340 Glyma15g07040 Glyma11g01350 Glyma13g34530

Glyma08g00790 Glyma18g02220 Glyma13g24200 Glyma13g01230

44


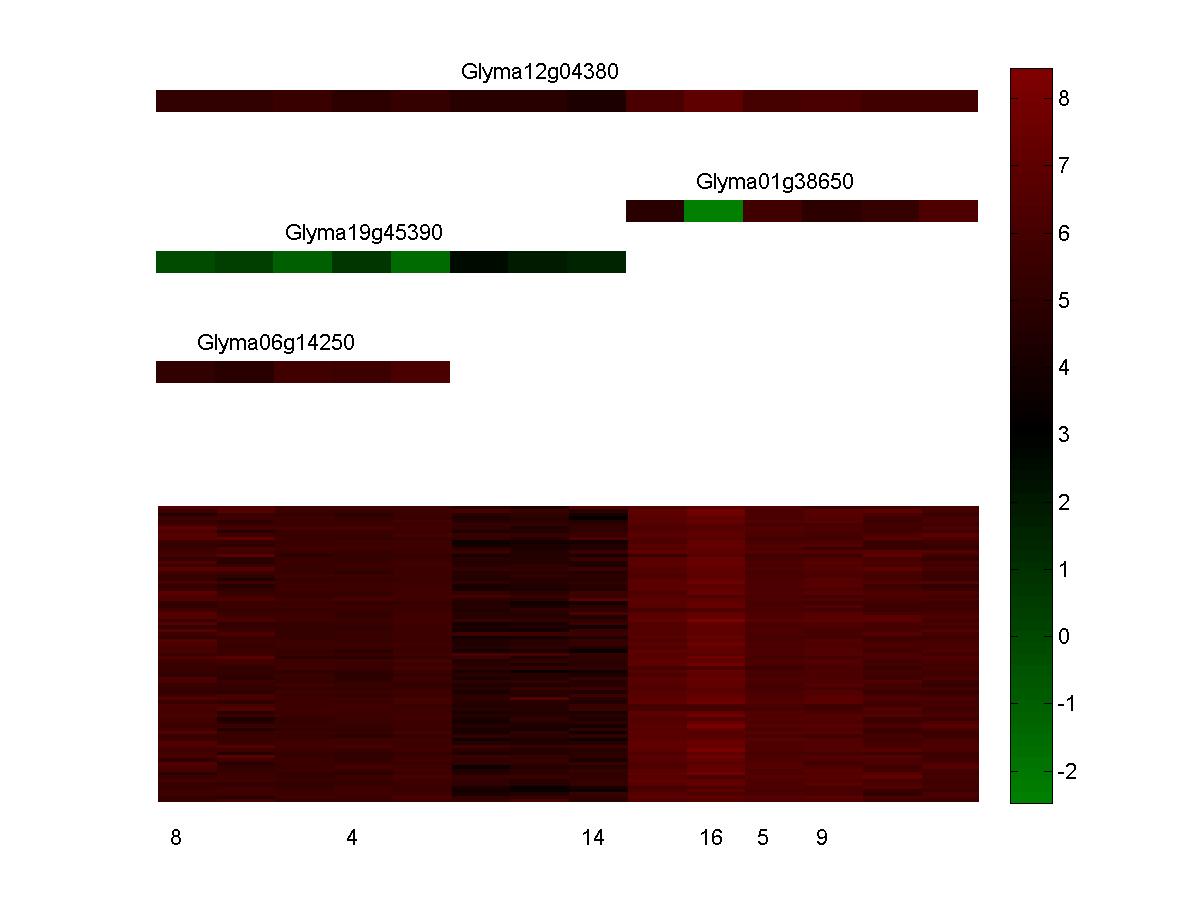


44 Glyma06g14250 NAC

44 Glyma01g38650 Homeodomain/HOMEOBOX

44 Glyma19g45390 Homeodomain/HOMEOBOX

44 Glyma12g04380 NAC

Glyma02g29430 Glyma17g35890 Glyma15g06160 Glyma13g44690 Glyma15g18170

Glyma08g22550 Glyma15g40650 Glyma07g37060 Glyma15g42940 Glyma17g04830

Glyma04g01270 Glyma07g01540 Glyma05g34980 Glyma18g00480 Glyma05g00470

Glyma11g00890 Glyma11g35450 Glyma03g37460 Glyma03g39420 Glyma11g37630

Glyma09g07410 Glyma05g03150 Glyma04g14640 Glyma12g30600 Glyma04g40980

Glyma07g10330 Glyma19g35780 Glyma08g22410 Glyma02g02140 Glyma20g03060

Glyma11g12180 Glyma19g42700 Glyma13g20330 Glyma08g18860 Glyma13g06070

Glyma10g06040 Glyma07g02720 Glyma20g34330 Glyma07g33800 Glyma09g31560

Glyma19g03520 Glyma13g35590 Glyma15g00610 Glyma11g10860 Glyma11g02190

Glyma16g33230 Glyma13g31900 Glyma01g36410 Glyma13g42090 Glyma12g04380

Glyma05g27940 Glyma18g01580 Glyma05g03850 Glyma18g50150 Glyma16g13580

Glyma13g36730 Glyma08g12200 Glyma09g15680 Glyma06g45220 Glyma04g40990

Glyma03g34700 Glyma13g37610 Glyma18g43390 Glyma05g36160 Glyma19g44920

Glyma03g36420 Glyma11g00450 Glyma08g40110 Glyma10g08910 Glyma17g11430

Glyma19g23570 Glyma18g01110 Glyma08g10540 Glyma15g08150 Glyma11g36090

Glyma08g46240 Glyma09g05030 Glyma16g03170 Glyma14g40120 Glyma16g22060

Glyma07g39980 Glyma03g00470 Glyma11g15050 Glyma03g29800 Glyma09g35650

Glyma06g14250 Glyma08g20130

45


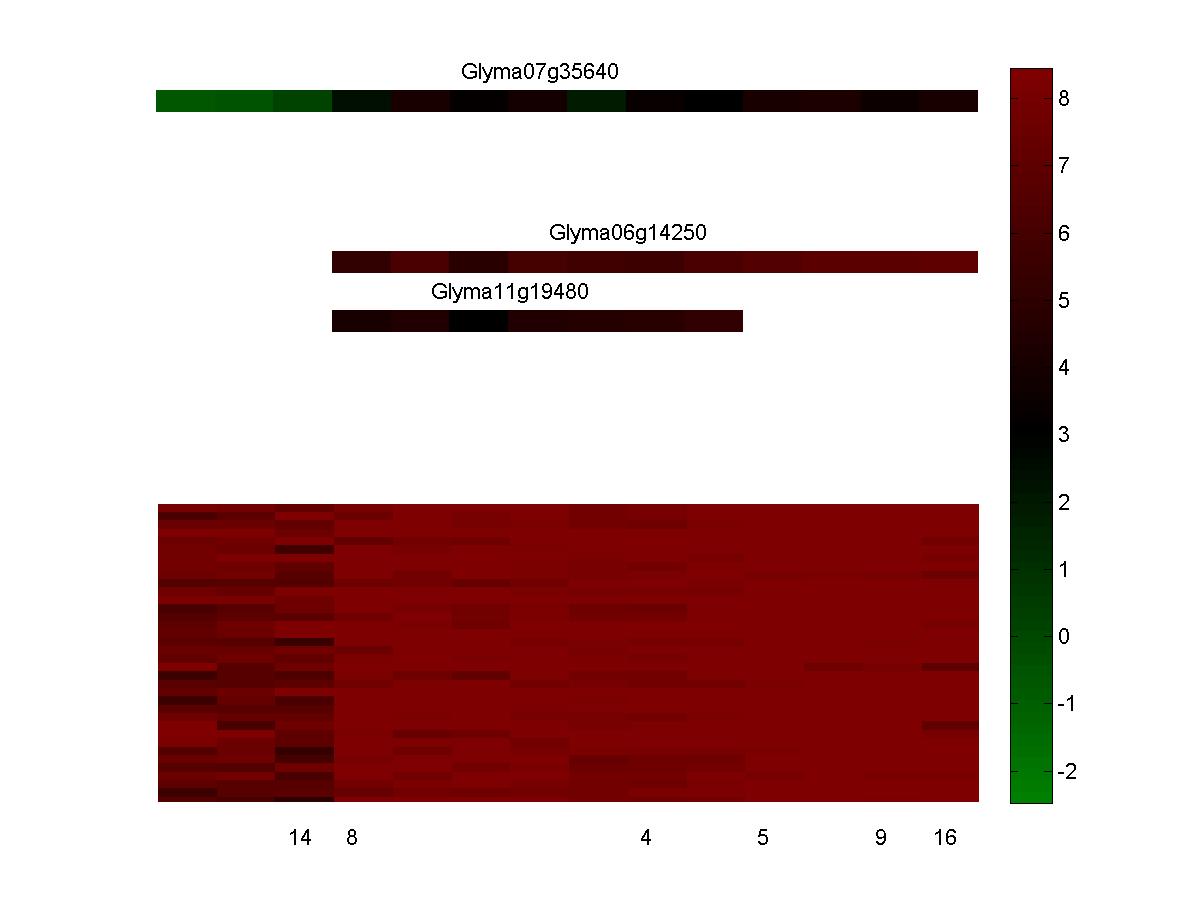


45 Glyma06g14250 NAC

45 Glyma11g19480 C2H2 (Zn)

45 Glyma07g35640 TPR

Glyma11g11300 Glyma18g08220 Glyma15g40860 Glyma12g02790 Glyma11g33560

Glyma05g29000 Glyma08g43290 Glyma10g05580 Glyma18g51980 Glyma09g02790

Glyma17g00600 Glyma11g11290 Glyma15g10220 Glyma08g46860 Glyma07g15610

Glyma19g35570 Glyma17g16830 Glyma12g03470 Glyma07g34440 Glyma17g04340

Glyma13g18830 Glyma17g09280 Glyma19g35560 Glyma08g46520 Glyma05g23230

Glyma05g24110 Glyma04g01380 Glyma14g09440 Glyma19g07240 Glyma08g11490

Glyma11g10480 Glyma03g35540 Glyma09g02160 Glyma02g09370 Glyma08g25950

Glyma20g27280

46


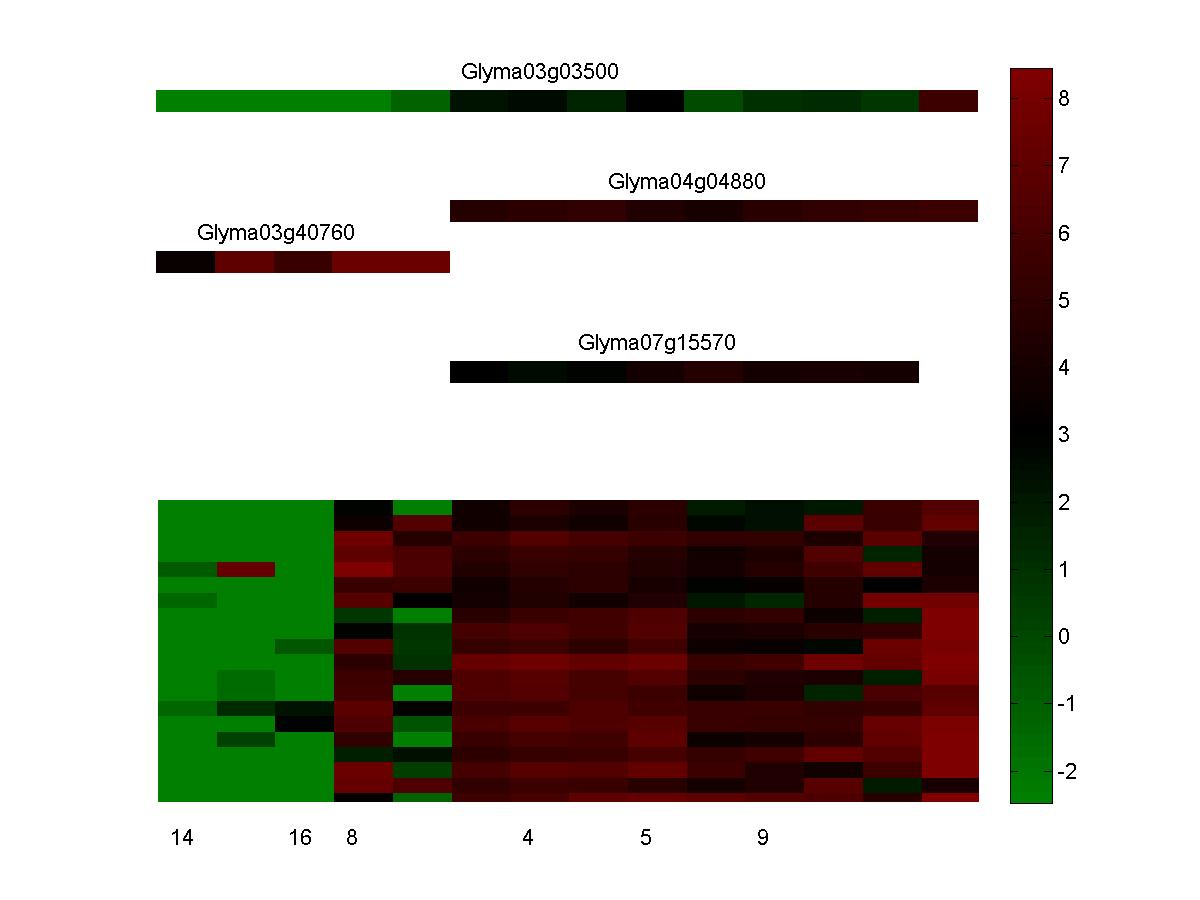


46 Glyma04g04880 LIM

46 Glyma07g15570 SSB protein

46 Glyma03g40760 AUX-IAA-ARF

46 Glyma03g03500 C2C2 (Zn) YABBY

Glyma04g08370 Glyma12g29100 Glyma18g10690 Glyma08g36360 Glyma18g53150

Glyma01g35620 Glyma20g05700 Glyma04g00710 Glyma18g12670 Glyma15g09530

Glyma03g32830 Glyma06g46360 Glyma07g31310 Glyma17g13720 Glyma02g45690

Glyma07g01740 Glyma20g29790 Glyma08g45610 Glyma08g36350 Glyma09g41460

47


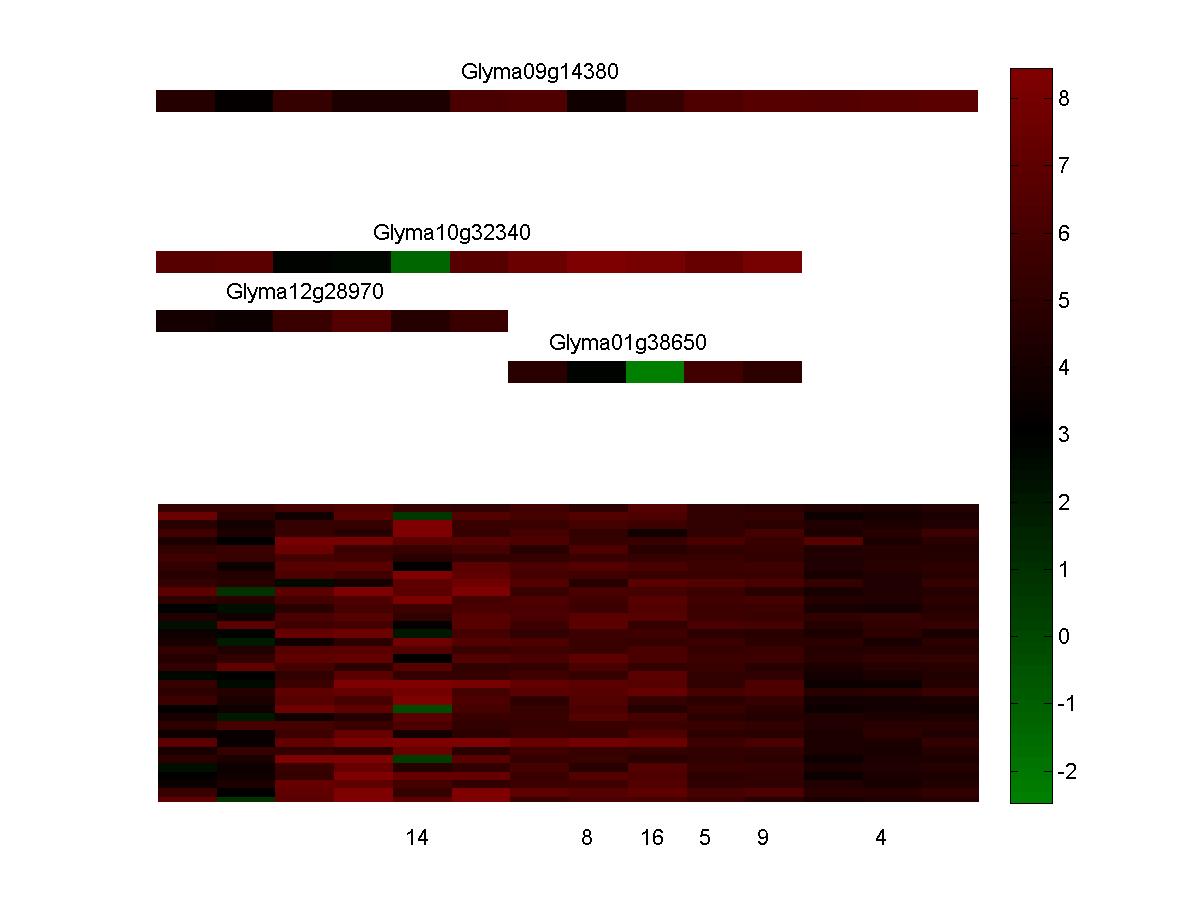


47 Glyma09g14380 bHLH

47 Glyma12g28970 TCP

47 Glyma10g32340 AUX-IAA-ARF

47 Glyma01g38650 Homeodomain/HOMEOBOX

Glyma06g33950 Glyma01g27810 Glyma15g41640 Glyma14g16700 Glyma01g01310

Glyma15g00350 Glyma05g28770 Glyma07g05150 Glyma02g39090 Glyma16g29370

Glyma13g11100 Glyma05g03770 Glyma15g03430 Glyma20g24670 Glyma08g01860

Glyma12g03510 Glyma06g05460 Glyma06g03470 Glyma07g02500 Glyma15g06670

Glyma09g40420 Glyma13g17420 Glyma07g00540 Glyma08g17490 Glyma11g00280

Glyma18g53440 Glyma09g08340 Glyma18g45420 Glyma17g05060 Glyma16g03020

Glyma01g45440 Glyma18g08530 Glyma04g09740 Glyma02g43640 Glyma07g21100

Glyma13g11090

48


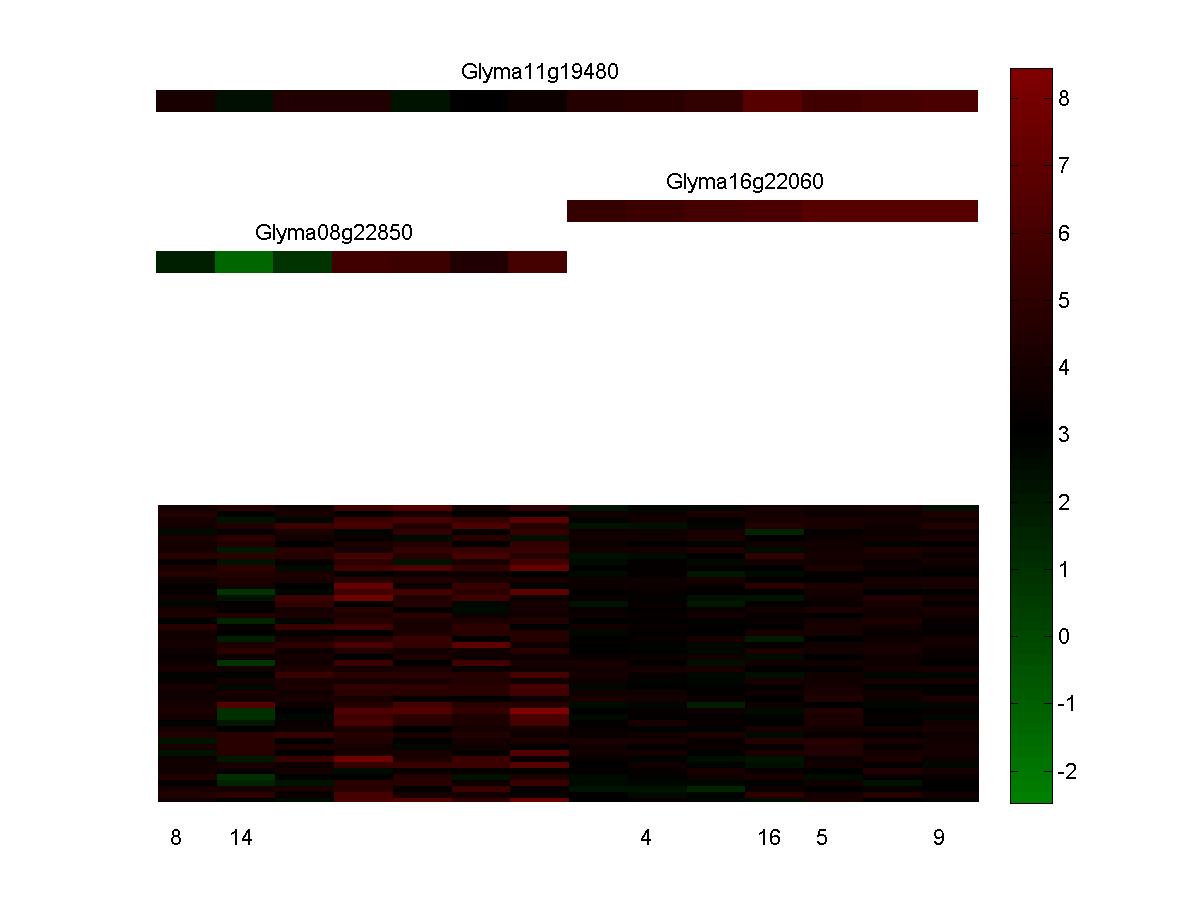


48 Glyma08g22850 TPR

48 Glyma11g19480 C2H2 (Zn)

48 Glyma16g22060 HMG

Glyma09g03660 Glyma11g09790 Glyma05g05450 Glyma17g15820 Glyma05g37840

Glyma14g39800 Glyma03g00840 Glyma03g42140 Glyma07g06440 Glyma13g20490

Glyma19g41150 Glyma06g44810 Glyma12g28710 Glyma17g01010 Glyma12g02850

Glyma01g23040 Glyma02g41430 Glyma07g11560 Glyma09g41070 Glyma01g45430

Glyma17g04330 Glyma05g26570 Glyma09g05820 Glyma17g16780 Glyma02g04600

Glyma13g03170 Glyma13g24340 Glyma11g13750 Glyma01g06030 Glyma12g04010

Glyma07g13880 Glyma07g00750 Glyma11g26970 Glyma18g18880 Glyma11g21150

Glyma09g09110 Glyma11g00300 Glyma05g34420 Glyma13g26550 Glyma19g29030

Glyma15g40060 Glyma16g04380 Glyma01g23050 Glyma11g10310 Glyma08g06040

Glyma17g15420 Glyma15g20680 Glyma13g22680 Glyma07g15570 Glyma13g24640

49


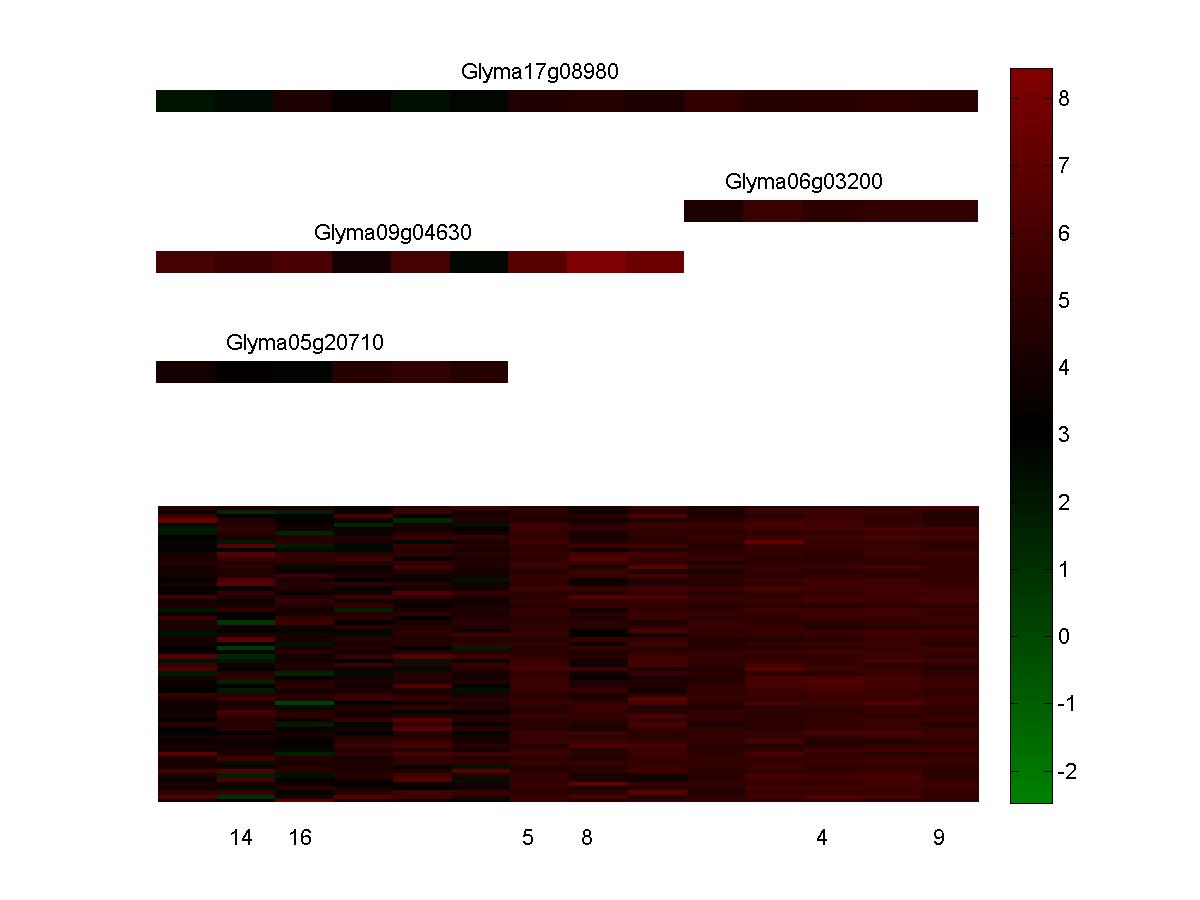


49 Glyma17g08980 bHLH

49 Glyma09g04630 AP2-EREBP

49 Glyma05g20710 WRKY

49 Glyma06g03200 Homeodomain/HOMEOBOX

Glyma10g40250 Glyma04g08000 Glyma06g14200 Glyma13g12070 Glyma01g02290

Glyma03g24180 Glyma10g23450 Glyma08g17240 Glyma18g47360 Glyma18g10930

Glyma14g27290 Glyma05g02080 Glyma18g10270 Glyma20g26580 Glyma18g51220

Glyma11g37760 Glyma11g19300 Glyma08g29920 Glyma19g16450 Glyma14g12220

Glyma18g46750 Glyma10g30440 Glyma12g06280 Glyma15g12930 Glyma12g10500

Glyma18g50940 Glyma08g26670 Glyma12g08820 Glyma09g02040 Glyma11g14840

Glyma19g43150 Glyma04g39340 Glyma16g33280 Glyma05g01680 Glyma04g06890

Glyma20g16480 Glyma04g35710 Glyma03g35000 Glyma04g03270 Glyma10g23440

Glyma15g31290 Glyma10g30020 Glyma18g15570 Glyma19g26070 Glyma15g10360

Glyma03g17450 Glyma08g08550 Glyma13g17650 Glyma16g04910 Glyma02g01570

Glyma08g11060 Glyma09g35760 Glyma14g24140 Glyma17g28450 Glyma18g10060

Glyma16g04560 Glyma13g33620 Glyma20g34750 Glyma13g07900 Glyma20g14390

Glyma17g09700 Glyma18g51260 Glyma15g11540 Glyma19g01400 Glyma13g27060

Glyma15g06870 Glyma17g08970 Glyma01g07120 Glyma06g04980 Glyma06g14710

DEG genes List

Glyma10g42940 Glyma09g27100 Glyma06g10450 Glyma20g30910 Glyma17g14620 Glyma20g22850 Glyma13g20170 Glyma02g02800 Glyma06g15520 Glyma02g45430 Glyma06g18560 Glyma06g18660 Glyma17g09200 Glyma06g18470 Glyma19g33700 Glyma08g25150 Glyma05g24850 Glyma19g39800 Glyma02g29430 Glyma10g40250 Glyma17g35890 Glyma05g26290 Glyma04g04540 Glyma06g07780 Glyma12g32580 Glyma0048s00300 Glyma16g00360 Glyma01g02950 Glyma20g24830 Glyma05g15700 Glyma15g06890 Glyma17g07060 Glyma08g17190 Glyma08g17190 Glyma08g17190 Glyma08g17190 Glyma11g27460 Glyma19g31480 Glyma19g42900 Glyma11g09420 Glyma11g09420 Glyma02g05570 Glyma03g41820 Glyma04g41590 Glyma08g43850 Glyma12g09020 Glyma12g09020 Glyma12g09020 Glyma15g03920 Glyma20g11220 Glyma06g08400 Glyma09g40050 Glyma07g25480 Glyma07g25480 Glyma02g00390 Glyma02g00390 Glyma17g07700 Glyma12g35060 Glyma02g40460 Glyma14g11040 Glyma08g11020 Glyma06g01040 Glyma02g43330 Glyma02g43330 Glyma06g06560 Glyma12g11250 Glyma15g43210 Glyma15g43210 Glyma08g26540 Glyma15g08480 Glyma15g08480 Glyma09g12440 Glyma12g36880 Glyma04g06560 Glyma0844s00200 Glyma0844s00200 Glyma03g40960 Glyma14g08260 Glyma14g08260 Glyma10g41960 Glyma07g10790 Glyma07g10790 Glyma08g36280 Glyma01g37040 Glyma02g11400 Glyma06g45170 Glyma10g01520 Glyma05g30740 Glyma08g44060 Glyma16g24450 Glyma16g24450 Glyma20g21090 Glyma19g12410 Glyma15g14410 Glyma08g15330 Glyma08g40420 Glyma15g19490 Glyma13g26100 Glyma06g22760 Glyma13g03270 Glyma06g15520 Glyma06g15520 Glyma05g27890 Glyma05g27890 Glyma11g05750 Glyma05g37370 Glyma02g12730 Glyma19g29230 Glyma08g44350 Glyma04g21390 Glyma07g29170 Glyma06g18560 Glyma15g16270 Glyma20g29770 Glyma20g29770 Glyma20g29770 Glyma05g25800 Glyma05g05530 Glyma02g22320 Glyma02g02720 Glyma13g21290 Glyma17g16930 Glyma05g29860 Glyma18g18840 Glyma18g18840 Glyma09g26340 Glyma03g03970 Glyma05g31600 Glyma01g16360 Glyma06g36090 Glyma17g05360 Glyma04g09980 Glyma12g13800 Glyma07g40060 Glyma20g17300 Glyma08g28140 Glyma20g25880 Glyma08g23890 Glyma09g10010 Glyma15g16090 Glyma07g05930 Glyma07g05930 Glyma12g12940 Glyma08g41770 Glyma02g46090 Glyma14g39970 Glyma11g30040 Glyma17g02400 Glyma17g09200 Glyma17g09200 Glyma08g03220 Glyma11g10850 Glyma09g34000 Glyma08g45420 Glyma18g32830 Glyma07g01030 Glyma19g40660 Glyma07g36130 Glyma17g14600 Glyma02g45030 Glyma17g32270 Glyma09g01180 Glyma14g17110 Glyma17g34570 Glyma06g18470 Glyma08g27890 Glyma08g25540 Glyma06g31610 Glyma19g34990 Glyma18g34800 Glyma13g16040 Glyma01g31650 Glyma01g31650 Glyma07g19710 Glyma09g02280 Glyma09g02280 Glyma07g01400 Glyma05g28490 Glyma18g46410 Glyma20g14940 Glyma05g30810 Glyma19g25860 Glyma19g25860 Glyma03g34940 Glyma09g03270 Glyma03g35370 Glyma20g34790 Glyma15g06080 Glyma10g14900 Glyma10g14900 Glyma11g37590 Glyma09g21310 Glyma13g23000 Glyma13g23000 Glyma15g35740 Glyma04g42680 Glyma14g09600 Glyma12g30740 Glyma17g27150 Glyma06g25350 Glyma06g25350 Glyma02g39870 Glyma13g31010 Glyma16g32750 Glyma05g29600 Glyma20g12270 Glyma13g35000 Glyma16g32610 Glyma09g03160 Glyma05g24850 Glyma18g35680 Glyma06g29730 Glyma18g01670 Glyma19g39800 Glyma02g29430 Glyma01g08010 Glyma03g15910 Glyma02g43710 Glyma15g22280 Glyma07g17280 Glyma04g38730 Glyma04g38730 Glyma07g39450 Glyma01g42810 Glyma01g42810 Glyma08g05500 Glyma01g02890 Glyma01g02890 Glyma13g02250 Glyma12g29760 Glyma12g29760 Glyma04g22520 Glyma10g40960 Glyma18g10460 Glyma14g03960 Glyma11g26480 Glyma18g06900 Glyma08g05480 Glyma08g05480 Glyma08g05480 Glyma01g01610 Glyma20g25120 Glyma10g40250 Glyma10g41430 Glyma10g41430 Glyma08g11120 Glyma06g36230 Glyma11g10500 Glyma17g32660 Glyma07g18200 Glyma17g13470 Glyma18g12470 Glyma01g35830 Glyma12g22240 Glyma03g29830 Glyma05g31250 Glyma16g31270 Glyma06g36210 Glyma06g36210 Glyma16g06660 Glyma18g04800 Glyma11g09760 Glyma13g00670 Glyma03g39880 Glyma04g06520 Glyma12g32540 Glyma12g32540 Glyma07g18440 Glyma07g18440 Glyma16g21400 Glyma08g16410 Glyma05g00510 Glyma04g27570 Glyma16g07210 Glyma10g42710 Glyma02g12520 Glyma12g19010 Glyma19g27410 Glyma09g33870 Glyma16g17210 Glyma19g09650 Glyma13g26310 Glyma01g34050 Glyma18g14540 Glyma07g07760 Glyma10g28770 Glyma20g31960 Glyma15g07370 Glyma14g38930 Glyma09g32340 Glyma06g06990 Glyma06g06990 Glyma03g42520 Glyma16g03720 Glyma10g32330 Glyma12g07140 Glyma02g41410 Glyma19g05930 Glyma01g30920 Glyma08g14710 Glyma20g01450 Glyma18g10280 Glyma10g13870 Glyma10g13870 Glyma20g13140 Glyma12g07490 Glyma18g32700 Glyma14g37880 Glyma01g07750 Glyma09g04990 Glyma08g07970 Glyma04g11860 Glyma19g42160 Glyma08g46030 Glyma16g27330 Glyma03g29200 Glyma06g37490 Glyma13g32790 Glyma05g05730 Glyma18g40870 Glyma07g08530 Glyma07g08530 Glyma20g26180 Glyma05g19350 Glyma13g37380 Glyma11g27230 Glyma11g27230 Glyma10g39010 Glyma10g39010 Glyma10g39010 Glyma10g39010 Glyma03g24710 Glyma03g24710 Glyma14g12550 Glyma14g12550 Glyma14g12550 Glyma14g12550 Glyma14g12550 Glyma08g46290 Glyma08g46290 Glyma08g46290 Glyma08g46290 Glyma17g22990 Glyma17g22990 Glyma17g22990 Glyma08g17190 Glyma08g17190 Glyma08g17190 Glyma08g17190 Glyma11g27460 Glyma11g27460 Glyma11g27460 Glyma19g31480 Glyma19g31480 Glyma19g31480 Glyma19g31480 Glyma20g18430 Glyma20g18430 Glyma20g18430 Glyma20g18430 Glyma20g18430 Glyma20g18430 Glyma20g30910 Glyma20g30910 Glyma20g30910 Glyma20g30910 Glyma19g42900 Glyma19g42900 Glyma19g42900 Glyma19g42900 Glyma19g42900 Glyma11g09420 Glyma16g21780 Glyma16g21780 Glyma01g30900 Glyma01g30900 Glyma01g30900 Glyma01g30900 Glyma01g30900 Glyma01g30900 Glyma05g17460 Glyma05g17460 Glyma05g17460 Glyma05g17460 Glyma10g23830 Glyma10g23830 Glyma02g05570 Glyma02g05570 Glyma02g05570 Glyma18g36070 Glyma18g36070 Glyma18g36070 Glyma03g41820 Glyma03g41820 Glyma03g41820 Glyma04g41590 Glyma04g41590 Glyma04g41590 Glyma04g41590 Glyma04g41590 Glyma05g38400 Glyma05g38400 Glyma05g38400 Glyma08g43850 Glyma08g43850 Glyma08g43850 Glyma08g43850 Glyma08g43850 Glyma06g17610 Glyma06g17610 Glyma06g17610 Glyma20g16070 Glyma20g16070 Glyma04g32420 Glyma04g32420 Glyma04g32420 Glyma12g09020 Glyma12g09020 Glyma12g09020 Glyma15g03920 Glyma15g03920 Glyma15g03920 Glyma15g03920 Glyma15g03920 Glyma15g03920 Glyma15g03920 Glyma15g03920 Glyma10g04170 Glyma10g04170 Glyma10g04170 Glyma20g11220 Glyma20g11220 Glyma20g11220 Glyma20g11220 Glyma20g11220 Glyma17g14620 Glyma17g14620 Glyma17g14620 Glyma09g29500 Glyma09g29500 Glyma09g29500 Glyma09g29500 Glyma02g18620 Glyma06g07520 Glyma02g36510 Glyma02g36510 Glyma06g08400 Glyma06g08400 Glyma16g30100 Glyma16g30100 Glyma16g30100 Glyma13g31210 Glyma09g40050 Glyma09g40050 Glyma09g40050 Glyma09g40050 Glyma09g40050 Glyma09g40050 Glyma08g26830 Glyma18g36600 Glyma18g36600 Glyma18g36600 Glyma07g25480 Glyma07g25480 Glyma07g25480 Glyma07g25480 Glyma07g25480 Glyma02g00390 Glyma02g00390 Glyma17g07700 Glyma17g07700 Glyma02g06040 Glyma02g06040 Glyma02g06040 Glyma02g06040 Glyma13g26530 Glyma13g26530 Glyma04g09810 Glyma04g09810 Glyma15g24350 Glyma15g24350 Glyma15g24350 Glyma15g24350 Glyma15g24350 Glyma15g24350 Glyma15g24350 Glyma14g38560 Glyma14g38560 Glyma14g38560 Glyma14g38560 Glyma14g38560 Glyma14g38560 Glyma14g38560 Glyma02g33990 Glyma02g33990 Glyma07g33350 Glyma07g33350 Glyma07g33350 Glyma12g35060 Glyma12g35060 Glyma12g35060 Glyma12g35060 Glyma12g35060 Glyma12g35060 Glyma12g35060 Glyma12g35060 Glyma20g18020 Glyma20g18020 Glyma20g18020 Glyma02g40460 Glyma02g40460 Glyma02g40460 Glyma02g40460 Glyma14g11040 Glyma14g11040 Glyma14g11040 Glyma09g30670 Glyma09g30670 Glyma08g11020 Glyma06g01040 Glyma06g01040 Glyma06g01040 Glyma09g30550 Glyma09g30550 Glyma09g30550 Glyma02g43330 Glyma02g43330 Glyma02g43330 Glyma02g43330 Glyma02g43330 Glyma06g06560 Glyma19g37810 Glyma19g37810 Glyma20g22850 Glyma20g22850 Glyma20g22850 Glyma07g05300 Glyma09g32960 Glyma09g32960 Glyma09g32960 Glyma09g32960 Glyma09g32960 Glyma04g32960 Glyma04g32960 Glyma04g32960 Glyma04g32960 Glyma04g32960 Glyma04g32960 Glyma15g43210 Glyma15g43210 Glyma15g43210 Glyma15g43210 Glyma15g43210 Glyma08g26540 Glyma08g26540 Glyma08g26540 Glyma08g26540 Glyma08g26540 Glyma08g26540 Glyma20g28550 Glyma20g28550 Glyma20g28550 Glyma20g28550 Glyma20g28550 Glyma20g28550 Glyma14g01300 Glyma14g01300 Glyma18g41350 Glyma18g41350 Glyma18g41350 Glyma18g41350 Glyma18g41350 Glyma06g21710 Glyma06g21710 Glyma06g21710 Glyma03g23990 Glyma18g52550 Glyma18g52550 Glyma18g52550 Glyma18g52550 Glyma18g52550 Glyma20g29040 Glyma12g18260 Glyma15g08480 Glyma15g08480 Glyma15g08480 Glyma15g08480 Glyma15g08480 Glyma09g12440 Glyma18g46880 Glyma18g46880 Glyma08g08620 Glyma08g08620 Glyma12g36880 Glyma12g36880 Glyma12g36880 Glyma13g20170 Glyma13g20170 Glyma13g20170 Glyma13g20170 Glyma13g20170 Glyma04g06560 Glyma0844s00200 Glyma0844s00200 Glyma0844s00200 Glyma0844s00200 Glyma08g03670 Glyma03g40960 Glyma03g40960 Glyma03g40960 Glyma03g40960 Glyma18g46420 Glyma18g46420 Glyma14g08260 Glyma14g08260 Glyma14g08260 Glyma14g08260 Glyma14g08260 Glyma17g01690 Glyma17g01690 Glyma17g01690 Glyma17g01690 Glyma18g46730 Glyma18g46730 Glyma18g46730 Glyma18g46730 Glyma18g46730 Glyma03g00980 Glyma14g10480 Glyma14g10480 Glyma14g10480 Glyma18g44730 Glyma18g44730 Glyma18g44730 Glyma18g44730 Glyma18g44730 Glyma05g27060 Glyma05g27060 Glyma05g27060 Glyma05g27060 Glyma05g27060 Glyma18g46710 Glyma18g46710 Glyma18g46710 Glyma18g46710 Glyma02g02800 Glyma03g26250 Glyma03g26250 Glyma03g26250 Glyma11g35610 Glyma11g35610 Glyma11g35610 Glyma11g35610 Glyma19g41360 Glyma19g41360 Glyma19g41360 Glyma12g14810 Glyma12g14810 Glyma12g14810 Glyma10g41960 Glyma10g41960 Glyma10g41960 Glyma10g41960 Glyma10g41960 Glyma07g34380 Glyma07g34380 Glyma07g34380 Glyma07g34380 Glyma07g34380 Glyma07g10790 Glyma07g10790 Glyma07g10790 Glyma07g10790 Glyma08g36280 Glyma08g36280 Glyma08g36280 Glyma08g36280 Glyma08g36280 Glyma11g17090 Glyma11g17090 Glyma11g17090 Glyma11g17090 Glyma11g17090 Glyma01g37040 Glyma01g37040 Glyma01g37040 Glyma02g11400 Glyma02g11400 Glyma02g40920 Glyma02g40920 Glyma02g40920 Glyma02g40920 Glyma06g45170 Glyma06g45170 Glyma06g45170 Glyma06g36290 Glyma06g36290 Glyma06g36290 Glyma06g36290 Glyma03g36840 Glyma03g36840 Glyma03g36840 Glyma03g36840 Glyma03g36840 Glyma14g10650 Glyma14g10650 Glyma14g10650 Glyma18g05590 Glyma18g05590 Glyma17g08410 Glyma10g09220 Glyma10g09220 Glyma06g16130 Glyma06g40810 Glyma10g01520 Glyma06g12620 Glyma06g12620 Glyma06g12620 Glyma06g12620 Glyma06g12620 Glyma02g27040 Glyma02g27040 Glyma02g27040 Glyma02g27040 Glyma02g27040 Glyma02g27040 Glyma13g20470 Glyma13g20470 Glyma13g20470 Glyma19g01650 Glyma19g01650 Glyma19g01650 Glyma05g30740 Glyma05g30740 Glyma05g30740 Glyma05g30740 Glyma03g33440 Glyma03g08730 Glyma03g08730 Glyma03g08730 Glyma03g08730 Glyma03g08730 Glyma08g44060 Glyma08g44060 Glyma08g44060 Glyma08g44060 Glyma08g44060 Glyma16g24450 Glyma16g24450 Glyma16g24450 Glyma16g24450 Glyma13g01090 Glyma13g01090 Glyma13g01090 Glyma13g01090 Glyma13g01090 Glyma04g02430 Glyma04g02430 Glyma20g21090 Glyma20g21090 Glyma20g21090 Glyma07g39000 Glyma07g39000 Glyma07g39000 Glyma04g05790 Glyma19g12410 Glyma19g12410 Glyma19g12410 Glyma15g14410 Glyma15g14410 Glyma15g14410 Glyma08g15330 Glyma08g15330 Glyma08g10000 Glyma08g10000 Glyma08g10000 Glyma08g40420 Glyma08g40420 Glyma07g08350 Glyma07g08350 Glyma07g08350 Glyma07g08350 Glyma18g51250 Glyma18g51250 Glyma15g19490 Glyma15g19490 Glyma15g19490 Glyma15g19490 Glyma09g05320 Glyma09g05320 Glyma09g05320 Glyma09g21170 Glyma02g26550 Glyma02g26550 Glyma05g25890 Glyma05g25890 Glyma05g25890 Glyma05g25890 Glyma13g26100 Glyma13g26100 Glyma13g26100 Glyma13g26100 Glyma13g26100 Glyma13g17790 Glyma13g17790 Glyma13g17790 Glyma13g17790 Glyma06g22760 Glyma12g06860 Glyma12g06860 Glyma12g06860 Glyma10g23650 Glyma10g23650 Glyma19g27610 Glyma19g27610 Glyma13g43190 Glyma13g43190 Glyma13g43190 Glyma13g43190 Glyma13g43190 Glyma05g08180 Glyma05g08180 Glyma13g03270 Glyma13g03270 Glyma13g03270 Glyma13g03270 Glyma13g03270 Glyma06g15520 Glyma06g15520 Glyma06g15520 Glyma13g42140 Glyma02g45430 Glyma02g45430 Glyma05g27890 Glyma05g27890 Glyma05g27890 Glyma05g27890 Glyma05g27890 Glyma18g14990 Glyma18g14990 Glyma18g14990 Glyma08g42800 Glyma08g42800 Glyma08g42800 Glyma08g42800 Glyma08g42800 Glyma08g42800 Glyma11g25950 Glyma11g25950 Glyma11g25950 Glyma11g25950 Glyma11g25950 Glyma11g25950 Glyma11g25950 Glyma11g25950 Glyma11g25950 Glyma11g05750 Glyma11g05750 Glyma11g05750 Glyma11g05750 Glyma11g05750 Glyma11g34080 Glyma11g34080 Glyma11g34080 Glyma01g44910 Glyma01g44910 Glyma01g44910 Glyma01g44910 Glyma01g44910 Glyma07g21000 Glyma09g29700 Glyma09g29700 Glyma09g29700 Glyma09g29700 Glyma01g15020 Glyma01g15020 Glyma11g35570 Glyma11g35570 Glyma11g35570 Glyma11g35570 Glyma10g36280 Glyma10g36280 Glyma10g36280 Glyma10g36280 Glyma10g36280 Glyma15g42280 Glyma15g42280 Glyma15g42280 Glyma15g42280 Glyma15g42280 Glyma18g50580 Glyma18g50580 Glyma18g50580 Glyma18g50580 Glyma18g50580 Glyma10g37030 Glyma10g37030 Glyma08g47710 Glyma08g47710 Glyma18g37390 Glyma18g37390 Glyma05g37370 Glyma05g37370 Glyma05g37370 Glyma08g43480 Glyma08g43480 Glyma08g14760 Glyma08g14760 Glyma08g14760 Glyma08g14760 Glyma08g14760 Glyma08g14760 Glyma04g11100 Glyma04g11100 Glyma04g11100 Glyma08g39500 Glyma08g39500 Glyma08g39500 Glyma08g39500 Glyma12g09370 Glyma12g09370 Glyma11g12130 Glyma11g12130 Glyma19g43630 Glyma19g43630 Glyma19g43630 Glyma19g43630 Glyma19g43630 Glyma10g00290 Glyma10g00290 Glyma10g00290 Glyma10g00290 Glyma18g28580 Glyma18g28580 Glyma18g28580 Glyma18g28580 Glyma18g28580 Glyma18g28580 Glyma10g40300 Glyma10g40300 Glyma10g40300 Glyma10g40300 Glyma10g40300 Glyma10g40300 Glyma10g40300 Glyma10g40300 Glyma10g40300 Glyma02g12730 Glyma02g12730 Glyma02g12730 Glyma02g12730 Glyma06g36580 Glyma06g36580 Glyma06g36580 Glyma06g36580 Glyma07g35060 Glyma07g35060 Glyma07g35060 Glyma06g04000 Glyma06g04000 Glyma06g04000 Glyma06g04000 Glyma06g04000 Glyma19g23230 Glyma19g23230 Glyma19g23230 Glyma19g23230 Glyma17g35620 Glyma17g35620 Glyma17g35620 Glyma17g35620 Glyma17g35620 Glyma17g35620 Glyma13g05390 Glyma13g05390 Glyma13g05390 Glyma08g04220 Glyma08g04220 Glyma08g04220 Glyma17g14790 Glyma17g14790 Glyma17g14790 Glyma19g29230 Glyma19g29230 Glyma19g29230 Glyma04g38070 Glyma04g38070 Glyma04g38070 Glyma04g38070 Glyma04g38070 Glyma04g38070 Glyma18g28150 Glyma18g28150 Glyma18g28150 Glyma18g28150 Glyma16g12130 Glyma16g12130 Glyma16g12130 Glyma16g12130 Glyma16g03150 Glyma16g03150 Glyma16g03150 Glyma15g07830 Glyma15g07830 Glyma15g07830 Glyma15g07830 Glyma06g14290 Glyma08g44350 Glyma08g44350 Glyma08g44350 Glyma08g44350 Glyma08g44350 Glyma09g26560 Glyma09g26560 Glyma09g26560 Glyma09g26560 Glyma08g45880 Glyma08g45880 Glyma08g45880 Glyma08g45880 Glyma03g07230 Glyma03g07230 Glyma03g07230 Glyma03g07230 Glyma04g14710 Glyma04g14710 Glyma04g14710 Glyma04g14710 Glyma04g21390 Glyma04g21390 Glyma04g21390 Glyma04g21390 Glyma04g21390 Glyma04g21390 Glyma16g09320 Glyma16g09320 Glyma08g08740 Glyma08g08740 Glyma08g08740 Glyma07g29170 Glyma07g29170 Glyma06g18560 Glyma06g18560 Glyma06g18560 Glyma05g00550 Glyma05g00550 Glyma05g00550 Glyma05g00550 Glyma15g16270 Glyma15g16270 Glyma15g16270 Glyma15g16270 Glyma15g16270 Glyma15g16270 Glyma01g33880 Glyma01g33880 Glyma01g33880 Glyma01g33880 Glyma01g33880 Glyma01g33880 Glyma01g33880 Glyma01g33880 Glyma13g17730 Glyma13g17730 Glyma13g17730 Glyma13g17730 Glyma08g07740 Glyma08g07740 Glyma08g07740 Glyma08g07740 Glyma11g17530 Glyma11g17530 Glyma11g17530 Glyma10g38970 Glyma10g38970 Glyma10g38970 Glyma10g38970 Glyma10g38970 Glyma10g38970 Glyma10g38970 Glyma10g38970 Glyma10g38970 Glyma10g38970 Glyma10g38970 Glyma20g29770 Glyma05g25800 Glyma05g25800 Glyma05g25800 Glyma05g05530 Glyma05g05530 Glyma05g05530 Glyma05g05530 Glyma05g05530 Glyma02g22320 Glyma02g22320 Glyma02g22320 Glyma02g02720 Glyma02g02720 Glyma02g02720 Glyma02g02720 Glyma02g02720 Glyma02g02720 Glyma02g02720 Glyma19g30640 Glyma19g30640 Glyma19g30640 Glyma19g30640 Glyma19g30640 Glyma07g26240 Glyma07g26240 Glyma07g26240 Glyma07g26240 Glyma07g26240 Glyma07g26240 Glyma07g26240 Glyma02g11520 Glyma02g11520 Glyma02g11520 Glyma02g11520 Glyma02g11520 Glyma13g21290 Glyma13g21290 Glyma13g21290 Glyma13g21290 Glyma03g29750 Glyma03g29750 Glyma19g28840 Glyma19g28840 Glyma19g28840 Glyma17g16930 Glyma17g16930 Glyma08g29120 Glyma08g29120 Glyma08g29120 Glyma08g29120 Glyma08g29120 Glyma08g29120 Glyma08g29120 Glyma18g06440 Glyma18g06440 Glyma17g07880 Glyma17g07880 Glyma17g07880 Glyma17g07880 Glyma05g29860 Glyma05g29860 Glyma05g29860 Glyma18g18840 Glyma18g18840 Glyma18g18840 Glyma09g26340 Glyma09g26340 Glyma09g26340 Glyma02g03800 Glyma02g03800 Glyma02g03800 Glyma02g03800 Glyma02g03800 Glyma18g34340 Glyma18g34340 Glyma18g34340 Glyma18g34340 Glyma18g34340 Glyma18g34340 Glyma18g34340 Glyma18g34340 Glyma18g34340 Glyma18g34340 Glyma03g03970 Glyma03g03970 Glyma18g11540 Glyma18g11540 Glyma18g11540 Glyma05g31600 Glyma05g31600 Glyma19g31620 Glyma19g31620 Glyma19g31620 Glyma19g31620 Glyma19g31620 Glyma19g31620 Glyma20g25160 Glyma20g25160 Glyma20g25160 Glyma05g23570 Glyma05g23570 Glyma04g03530 Glyma04g03530 Glyma01g16360 Glyma01g16360 Glyma01g16360 Glyma01g16360 Glyma18g51070 Glyma18g51070 Glyma18g51070 Glyma18g51070 Glyma18g51070 Glyma06g36090 Glyma06g36090 Glyma06g36090 Glyma06g36090 Glyma06g36090 Glyma19g02440 Glyma19g02440 Glyma19g02440 Glyma17g05360 Glyma17g05360 Glyma17g05360 Glyma07g07010 Glyma07g07010 Glyma19g02890 Glyma19g02890 Glyma19g02890 Glyma19g02890 Glyma04g11940 Glyma04g11940 Glyma04g11940 Glyma04g11940 Glyma15g05510 Glyma15g05510 Glyma15g05510 Glyma20g22510 Glyma20g22510 Glyma04g09980 Glyma04g09980 Glyma04g09980 Glyma14g23940 Glyma14g23940 Glyma14g23940 Glyma14g23940 Glyma12g13800 Glyma12g13800 Glyma07g40060 Glyma07g40060 Glyma20g26370 Glyma20g26370 Glyma03g20900 Glyma03g20900 Glyma03g20900 Glyma15g12080 Glyma15g12080 Glyma15g12080 Glyma12g16450 Glyma12g16450 Glyma12g16450 Glyma16g27300 Glyma16g27300 Glyma16g27300 Glyma16g27300 Glyma16g27300 Glyma16g27300 Glyma16g27300 Glyma16g27300 Glyma20g17300 Glyma20g17300 Glyma20g17300 Glyma15g28320 Glyma15g28320 Glyma15g28320 Glyma15g28320 Glyma09g30360 Glyma07g08880 Glyma07g08880 Glyma07g08880 Glyma17g33600 Glyma17g33600 Glyma17g33600 Glyma17g33600 Glyma02g00550 Glyma02g00550 Glyma02g00550 Glyma02g00550 Glyma02g00550 Glyma02g00550 Glyma19g22780 Glyma19g22780 Glyma19g22780 Glyma14g05860 Glyma14g05860 Glyma20g25880 Glyma20g25880 Glyma20g25880 Glyma20g25880 Glyma20g25880 Glyma20g25880 Glyma06g18660 Glyma06g18660 Glyma06g18660 Glyma06g18660 Glyma08g23890 Glyma08g23890 Glyma08g23890 Glyma06g43210 Glyma06g43210 Glyma06g43210 Glyma15g12010 Glyma15g12010 Glyma15g12010 Glyma18g45250 Glyma18g45250 Glyma18g45250 Glyma09g10010 Glyma09g10010 Glyma16g03650 Glyma16g03650 Glyma15g16090 Glyma15g16090 Glyma15g16090 Glyma15g16090 Glyma15g16090 Glyma04g05100 Glyma06g11830 Glyma06g11830 Glyma20g21470 Glyma20g21470 Glyma20g21470 Glyma20g21470 Glyma20g21470 Glyma07g05930 Glyma07g05930 Glyma07g05930 Glyma09g29490 Glyma09g29490 Glyma09g29490 Glyma09g29490 Glyma09g29490 Glyma05g35970 Glyma05g35970 Glyma05g35970 Glyma05g35970 Glyma05g35970 Glyma05g35970 Glyma05g35690 Glyma03g36270 Glyma03g36270 Glyma07g17930 Glyma07g17930 Glyma14g32700 Glyma14g32700 Glyma14g32700 Glyma12g12940 Glyma12g12940 Glyma12g12940 Glyma12g12940 Glyma12g12940 Glyma05g25110 Glyma05g25110 Glyma05g25110 Glyma08g41770 Glyma08g41770 Glyma08g41770 Glyma08g41770 Glyma08g41770 Glyma05g34920 Glyma05g34920 Glyma09g03470 Glyma09g03470 Glyma09g03470 Glyma14g05070 Glyma14g05070 Glyma14g05070 Glyma14g05070 Glyma19g09030 Glyma19g09030 Glyma19g09030 Glyma19g09030 Glyma19g09030 Glyma19g09030 Glyma01g09230 Glyma01g09230 Glyma01g09230 Glyma04g41950 Glyma09g12510 Glyma09g12510 Glyma09g12510 Glyma09g12510 Glyma09g12510 Glyma09g12510 Glyma09g12510 Glyma09g12510 Glyma09g12510 Glyma11g21200 Glyma11g21200 Glyma04g01900 Glyma04g01900 Glyma04g01900 Glyma04g01900 Glyma12g01370 Glyma12g01370 Glyma13g43640 Glyma13g43640 Glyma13g43640 Glyma13g43640 Glyma13g43640 Glyma13g43640 Glyma20g17500 Glyma20g17500 Glyma20g17500 Glyma02g46090 Glyma14g39970 Glyma14g39970 Glyma14g39970 Glyma13g24780 Glyma13g24780 Glyma13g24780 Glyma13g24780 Glyma11g30040 Glyma11g30040 Glyma11g30040 Glyma11g30040 Glyma11g30040 Glyma18g10570 Glyma18g10570 Glyma06g44170 Glyma08g17760 Glyma08g17760 Glyma08g17760 Glyma17g02400 Glyma17g02400 Glyma17g09200 Glyma08g03220 Glyma08g03220 Glyma08g03220 Glyma11g10850 Glyma11g10850 Glyma12g23390 Glyma09g34000 Glyma16g03390 Glyma16g03390 Glyma16g03390 Glyma18g25870 Glyma18g25870 Glyma18g25870 Glyma08g45420 Glyma08g45420 Glyma08g45420 Glyma08g45420 Glyma18g32830 Glyma18g32830 Glyma18g32830 Glyma19g32470 Glyma19g32470 Glyma19g32470 Glyma06g08740 Glyma06g08740 Glyma06g08740 Glyma06g08740 Glyma07g02830 Glyma07g02830 Glyma07g02830 Glyma07g02830 Glyma07g02830 Glyma07g02830 Glyma07g01030 Glyma07g01030 Glyma07g01030 Glyma07g01030 Glyma02g27240 Glyma02g03560 Glyma02g03560 Glyma02g03560 Glyma02g03560 Glyma02g03560 Glyma15g20010 Glyma19g25690 Glyma19g25690 Glyma19g25690 Glyma19g25690 Glyma05g14670 Glyma05g14670 Glyma05g14670 Glyma05g14670 Glyma05g14670 Glyma19g40660 Glyma19g40660 Glyma19g40660 Glyma19g40660 Glyma19g40660 Glyma07g33060 Glyma07g33060 Glyma07g33060 Glyma07g33060 Glyma18g02640 Glyma07g36130 Glyma07g36130 Glyma07g36130 Glyma07g36130 Glyma02g45030 Glyma02g45030 Glyma11g17010 Glyma02g31560 Glyma02g31560 Glyma02g31560 Glyma02g31560 Glyma02g31560 Glyma02g31560 Glyma13g36110 Glyma13g36110 Glyma13g36110 Glyma13g36110 Glyma13g36110 Glyma13g36110 Glyma13g36110 Glyma01g45090 Glyma01g45090 Glyma01g45090 Glyma01g45090 Glyma01g45090 Glyma04g14750 Glyma04g14750 Glyma04g14750 Glyma04g14750 Glyma04g14750 Glyma19g38960 Glyma19g38960 Glyma17g32270 Glyma17g32270 Glyma17g32270 Glyma17g32270 Glyma15g23680 Glyma15g23680 Glyma15g23680 Glyma15g23680 Glyma03g40380 Glyma03g40380 Glyma03g40380 Glyma09g01180 Glyma09g01180 Glyma09g01180 Glyma09g01180 Glyma09g01180 Glyma09g01180 Glyma09g12220 Glyma09g12220 Glyma09g12220 Glyma18g29680 Glyma18g29680 Glyma18g29680 Glyma18g29680 Glyma18g29680 Glyma18g29680 Glyma08g27730 Glyma08g27730 Glyma08g27730 Glyma14g17110 Glyma14g17110 Glyma14g17110 Glyma14g17110 Glyma14g17110 Glyma14g17110 Glyma14g17110 Glyma14g17110 Glyma14g39710 Glyma14g39710 Glyma14g39710 Glyma14g39710 Glyma19g22860 Glyma19g22860 Glyma19g22860 Glyma19g22860 Glyma17g34570 Glyma17g34570 Glyma06g18470 Glyma06g18470 Glyma06g18470 Glyma15g41300 Glyma15g41300 Glyma15g41300 Glyma15g41300 Glyma07g01090 Glyma08g27890 Glyma08g27890 Glyma08g25540 Glyma08g25540 Glyma08g25540 Glyma06g31610 Glyma09g02000 Glyma10g15260 Glyma10g15260 Glyma17g07080 Glyma17g07080 Glyma17g07080 Glyma17g07080 Glyma17g07080 Glyma05g33560 Glyma05g33560 Glyma05g33560 Glyma05g33560 Glyma06g00670 Glyma06g00670 Glyma06g00670 Glyma19g34990 Glyma19g34990 Glyma19g34990 Glyma19g34990 Glyma18g34800 Glyma18g34800 Glyma18g34800 Glyma18g34800 Glyma18g34800 Glyma06g19160 Glyma06g19160 Glyma13g16040 Glyma13g16040 Glyma13g16040 Glyma05g34300 Glyma05g34300 Glyma16g01920 Glyma16g01920 Glyma16g01920 Glyma16g01920 Glyma16g01920 Glyma18g15940 Glyma18g15940 Glyma18g15940 Glyma18g15940 Glyma18g15940 Glyma18g15940 Glyma18g15940 Glyma18g15940 Glyma18g15940 Glyma17g37430 Glyma17g37430 Glyma17g37430 Glyma15g09610 Glyma15g09610 Glyma15g09610 Glyma15g09610 Glyma15g09610 Glyma04g11260 Glyma04g11260 Glyma04g11260 #number reads mapped uniquely = 4693603 #number reads mapped uniquely = 4693603 #number reads mapped uniquely = 4693603 #number reads mapped uniquely = 4693603 Glyma07g19710 Glyma07g19710 Glyma07g19710 Glyma07g19710 Glyma07g19710 Glyma09g02280 Glyma09g02280 Glyma09g02280 Glyma09g02280 Glyma06g23450 Glyma06g23450 Glyma20g36760 Glyma20g36760 Glyma20g36760 Glyma20g36760 Glyma05g05130 Glyma05g05130 Glyma12g29380 Glyma12g29380 Glyma20g25440 Glyma20g25440 Glyma20g25440 Glyma07g01400 Glyma06g08790 Glyma06g08790 Glyma17g31960 Glyma17g31960 Glyma13g20760 Glyma13g20760 Glyma13g20760 Glyma13g20760 Glyma13g20760 Glyma07g09420 Glyma07g09420 Glyma07g09420 Glyma07g09420 Glyma05g28800 Glyma05g28800 Glyma05g28800 Glyma03g29360 Glyma03g29360 Glyma03g29360 Glyma03g29360 Glyma03g29360 Glyma05g28490 Glyma05g28490 Glyma05g28490 Glyma05g28490 Glyma18g46410 Glyma18g46410 Glyma18g46410 Glyma20g14940 Glyma20g14940 Glyma20g14940 Glyma08g08850 Glyma08g08850 Glyma08g08850 Glyma05g30810 Glyma05g30810 Glyma05g30810 Glyma05g30810 Glyma05g30810 Glyma05g30810 Glyma02g11150 Glyma02g11150 Glyma02g11150 Glyma02g11150 Glyma02g11150 Glyma18g33800 Glyma18g33800 Glyma18g33800 Glyma18g33800 Glyma18g33800 Glyma18g33800 Glyma18g33800 Glyma06g38420 Glyma06g38420 Glyma06g38420 Glyma11g36800 Glyma11g36800 Glyma11g36800 Glyma11g36800 Glyma15g07150 Glyma15g07150 Glyma15g07150 Glyma15g07150 Glyma15g07150 Glyma19g25860 Glyma19g25860 Glyma03g34940 Glyma03g34940 Glyma03g34940 Glyma03g34940 Glyma09g03270 Glyma09g03270 Glyma09g03270 Glyma15g36680 Glyma15g36680 Glyma15g36680 Glyma15g36680 Glyma03g01620 Glyma03g01620 Glyma03g35370 Glyma03g35370 Glyma03g35370 Glyma03g35370 Glyma03g35370 Glyma10g40360 Glyma10g40360 Glyma09g30280 Glyma09g30280 Glyma19g28440 Glyma19g28440 Glyma19g28440 Glyma20g34790 Glyma20g34790 Glyma20g34790 Glyma20g34790 Glyma20g34790 Glyma20g34790 Glyma20g34790 Glyma12g01240 Glyma12g01240 Glyma15g06080 Glyma15g06080 Glyma15g06080 Glyma17g23180 Glyma17g23180 Glyma17g23180 Glyma05g23560 Glyma05g23560 Glyma05g23560 Glyma05g23560 Glyma05g23560 Glyma05g23560 Glyma01g32460 Glyma01g32460 Glyma01g32460 Glyma01g32460 Glyma01g32460 Glyma10g14900 Glyma10g14900 Glyma10g14900 Glyma04g09020 Glyma18g08170 Glyma18g08170 Glyma18g08170 Glyma04g32270 Glyma04g32270 Glyma04g32270 Glyma04g32270 Glyma04g32270 Glyma02g07380 Glyma02g07380 Glyma02g07380 Glyma02g07380 Glyma11g37590 Glyma11g37590 Glyma11g37590 Glyma11g37590 Glyma11g37590 Glyma03g41770 Glyma03g41770 Glyma03g41770 Glyma14g08050 Glyma0859s00200 Glyma0859s00200 Glyma0859s00200 Glyma0859s00200 Glyma0859s00200 Glyma20g01620 Glyma09g21310 Glyma09g21310 Glyma09g21310 Glyma09g21310 Glyma12g29800 Glyma12g29800 Glyma12g29800 Glyma13g23000 Glyma13g23000 Glyma13g23000 Glyma13g23000 Glyma15g35740 Glyma15g35740 Glyma05g30900 Glyma05g30900 Glyma05g30900 Glyma11g30450 Glyma11g30450 Glyma11g30450 Glyma11g30450 Glyma19g27600 Glyma19g27600 Glyma19g27600 Glyma19g27600 Glyma19g27600 Glyma19g27600 Glyma04g42680 Glyma04g42680 Glyma04g42680 Glyma04g42680 Glyma17g27150 Glyma07g03020 Glyma07g03020 Glyma07g03020 Glyma07g03020 Glyma19g24490 Glyma19g24490 Glyma19g24490 Glyma10g09470 Glyma10g09470 Glyma10g09470 Glyma04g05610 Glyma04g05610 Glyma04g05610 Glyma04g05610 Glyma19g33700 Glyma07g19090 Glyma07g19090 Glyma07g19090 Glyma07g19090 Glyma07g00740 Glyma07g00740 Glyma07g00740 Glyma10g33320 Glyma10g33320 Glyma20g13340 Glyma20g13340 Glyma20g13340 Glyma20g13340 Glyma20g13340 Glyma20g13340 Glyma20g13340 Glyma20g13340 Glyma20g13340 Glyma06g25350 Glyma16g34170 Glyma16g34170 Glyma16g34170 Glyma16g34170 Glyma02g39870 Glyma02g39870 Glyma02g39870 Glyma05g28050 Glyma05g28050 Glyma05g28050 Glyma05g28050 Glyma02g42540 Glyma02g42540 Glyma02g42540 Glyma02g42540 Glyma02g42540 Glyma02g42540 Glyma10g43070 Glyma10g43070 Glyma10g43070 Glyma10g43070 Glyma10g01630 Glyma10g01630 Glyma12g22200 Glyma12g22200 Glyma12g22200 Glyma19g32880 Glyma19g32880 Glyma19g32880 Glyma19g32880 Glyma19g37550 Glyma19g37550 Glyma19g37550 Glyma13g31010 Glyma13g31010 Glyma13g31010 Glyma13g31010 Glyma13g31010 Glyma13g31010 Glyma13g31010 Glyma16g32750 Glyma03g38650 Glyma03g38650 Glyma05g29600 Glyma18g38550 Glyma18g38550 Glyma18g38550 Glyma18g38550 Glyma08g25150 Glyma13g00410 Glyma13g00410 Glyma12g33650 Glyma12g33650 Glyma12g33650 Glyma08g18620 Glyma08g18620 Glyma08g18620 Glyma20g12270 Glyma20g12270 Glyma20g12270 Glyma07g06140 Glyma07g06140 Glyma07g06140 Glyma18g34400 Glyma18g34400 Glyma18g34400 Glyma11g06530 Glyma11g06530 Glyma14g40000 Glyma14g40000 Glyma13g31490 Glyma13g31490 Glyma13g31490 Glyma13g31490 Glyma13g35000 Glyma13g35000 Glyma13g35000 Glyma13g35000 Glyma05g17080 Glyma05g17080 Glyma05g17080 Glyma05g17080 Glyma04g04650 Glyma04g04650 Glyma04g04650 Glyma04g04650 Glyma04g04650 Glyma04g04650 Glyma05g14810 Glyma05g14810 Glyma05g14810 Glyma05g14810 Glyma05g14810 Glyma10g30710 Glyma10g30710 Glyma10g30710 Glyma10g30710 Glyma04g34390 Glyma04g34390 Glyma04g34390 Glyma04g34390 Glyma16g32610 Glyma14g19000 Glyma14g19000 Glyma14g19000 Glyma14g19000 Glyma09g03160 Glyma09g03160 Glyma09g03160 Glyma09g03160 Glyma09g03160 Glyma08g35450 Glyma01g24970 Glyma01g24970 Glyma01g24970 Glyma01g24970 Glyma01g24970 Glyma14g40100 Glyma14g40100 Glyma13g01130 Glyma13g01130 Glyma13g01130 Glyma13g01130 Glyma13g01130 Glyma03g02620 Glyma03g02620 Glyma03g02620 Glyma03g02620 Glyma03g02620 Glyma03g04180 Glyma06g22250 Glyma06g22250 Glyma06g22250 Glyma05g24850 Glyma17g10090 Glyma17g10090 Glyma17g10090 Glyma17g10090 Glyma17g10090 Glyma17g10090 Glyma17g10090 Glyma18g35680 Glyma18g35680 Glyma18g35680 Glyma18g35680 Glyma18g35680 Glyma06g29730 Glyma06g29730 Glyma18g01670 Glyma06g45690 Glyma06g45690 Glyma06g45690 Glyma14g34060 Glyma14g34060 Glyma14g34060 Glyma14g34060 Glyma19g39800 Glyma19g39800 Glyma09g36920 Glyma09g36920 Glyma09g36920 Glyma09g36920 Glyma14g00970 Glyma14g00970 Glyma14g00970 Glyma06g03670 Glyma06g03670 Glyma06g03670 Glyma06g03670 Glyma02g29430 Glyma02g29430 Glyma12g04800 Glyma12g04800 Glyma12g04800 Glyma12g04800 Glyma12g04800 Glyma12g04800 Glyma06g36690 Glyma06g36690 Glyma05g16420 Glyma05g16420 Glyma05g16420 Glyma05g16420 Glyma05g16420 Glyma10g42810 Glyma10g42810 Glyma0021s00420 Glyma0021s00420 Glyma0021s00420 Glyma0021s00420 Glyma0021s00420 Glyma0021s00420 Glyma18g07290 Glyma18g07290 Glyma18g07290 Glyma18g07290 Glyma18g07290 Glyma11g27120 Glyma11g27120 Glyma11g27120 Glyma11g27120 Glyma11g27120 Glyma15g38140 Glyma15g38140 Glyma01g08010 Glyma01g08010 Glyma03g15910 Glyma03g15910 Glyma03g37540 Glyma03g37540 Glyma03g37540 Glyma03g37540 Glyma16g29020 Glyma16g29020 Glyma16g29020 Glyma16g29020 Glyma02g43710 Glyma02g43710 Glyma02g43710 Glyma02g43710 Glyma08g18730 Glyma08g18730 Glyma13g25000 Glyma13g25000 Glyma13g25000 Glyma13g25000 Glyma13g25000 Glyma13g25000 Glyma13g25000 Glyma15g22280 Glyma15g05750 Glyma15g05750 Glyma15g05750 Glyma15g05750 Glyma15g05750 Glyma05g14350 Glyma05g14350 Glyma05g14350 Glyma05g14350 Glyma05g14350 Glyma05g14350 Glyma07g17280 Glyma07g17280 Glyma07g17280 Glyma07g17280 Glyma07g17280 Glyma04g12130 Glyma04g12130 Glyma04g12130 Glyma08g28790 Glyma08g28790 Glyma08g28790 Glyma08g28790 Glyma08g28790 Glyma12g01690 Glyma12g01690 Glyma04g38730 Glyma16g21450 Glyma16g21450 Glyma16g21450 Glyma16g21450 Glyma16g21450 Glyma18g14610 Glyma18g14610 Glyma18g14610 Glyma03g35300 Glyma03g35300 Glyma07g39450 Glyma07g39450 Glyma07g39450 Glyma10g25700 Glyma10g25700 Glyma03g22000 Glyma03g22000 Glyma15g17790 Glyma15g17790 Glyma15g17790 Glyma15g17790 Glyma15g17790 Glyma15g17790 Glyma02g19330 Glyma02g19330 Glyma02g19330 Glyma09g35000 Glyma09g35000 Glyma09g35000 Glyma09g35000 Glyma09g35000 Glyma01g38420 Glyma01g38420 Glyma01g38420 Glyma01g38420 Glyma07g09210 Glyma07g09210 Glyma07g09210 Glyma07g09210

Genes with sequence AAAGAT

Module1

Glyma06g09340

Glyma07g16490

Glyma07g35640

Glyma08g21630

Glyma10g07550

Glyma11g18370

Module2

Glyma19g37230

Glyma20g34820

Module3

Glyma01g02950

Glyma01g06970

Glyma02g12870

Glyma03g40860

Glyma07g05230

Glyma09g12320

Glyma09g27100

Glyma09g36000

Glyma13g10640

Glyma13g19830

Glyma13g19950

Glyma14g07690

Glyma14g36610

Module4

Glyma01g03470

Glyma02g06730

Glyma02g15520

Glyma03g28080

Glyma09g21820

Glyma10g07500

Glyma16g05770

Glyma19g38800

Module5

Glyma03g24020

Glyma04g40920

Glyma07g09520

Glyma08g44820

Glyma09g24410

Glyma11g37990

Glyma14g04950

Glyma17g08250

Module6

Glyma01g38410

Glyma02g40940

Glyma03g28850

Glyma05g04960

Glyma05g24850

Glyma06g03100

Glyma07g37100

Glyma08g03540

Glyma08g09680

Glyma09g39230

Glyma11g15090

Glyma11g18980

Glyma13g05120

Glyma15g06020

Glyma18g47730

Module7

Glyma01g40150

Glyma02g44460

Glyma02g45430

Glyma03g31460

Glyma05g04520

Glyma05g27600

Glyma05g34680

Glyma06g08680

Glyma06g13870

Glyma07g38520

Glyma09g06930

Glyma10g42940

Glyma11g02900

Glyma11g16210

Glyma12g04510

Glyma12g07040

Glyma12g32000

Glyma13g31650

Glyma13g34670

Glyma13g39490

Glyma15g02610

Glyma15g03120

Glyma15g04670

Glyma16g05530

Glyma17g05030

Glyma17g23830

Glyma18g02970

Glyma18g49340

Glyma19g36250

Glyma19g39940

Glyma19g42320

Glyma20g22090

Glyma20g29190

Module8

Glyma08g12650

Glyma09g28750

Glyma13g28970

Glyma16g04760

Glyma17g35230

Module9

Glyma02g44710

Glyma04g00420

Glyma04g40000

Glyma08g01410

Glyma08g10160

Glyma08g47310

Glyma09g04940

Glyma10g00520

Glyma11g20600

Glyma15g01950

Glyma17g07900

Module10

Glyma07g00900

Glyma12g06100

Module11

Glyma01g44040

Glyma02g15190

Glyma03g36470

Glyma04g09820

Glyma04g11290

Glyma05g00400

Glyma06g09420

Glyma07g38940

Glyma16g23730

Glyma17g17850

Glyma18g02210

Glyma18g52860

Glyma20g34880

Module12

Glyma02g11540

Glyma02g47210

Glyma03g36560

Glyma03g40280

Glyma04g11400

Glyma04g40470

Glyma05g36420

Glyma06g05410

Glyma08g03150

Glyma10g04560

Glyma10g40460

Glyma11g37970

Glyma13g06390

Glyma14g06630

Glyma15g13080

Glyma16g24120

Glyma19g39240

Module13

Glyma09g01320

Glyma12g34550

Glyma15g12170

Glyma17g34870

Module14

Glyma01g01390

Glyma02g06120

Glyma02g46200

Glyma04g03290

Glyma04g33570

Glyma04g41530

Glyma04g42830

Glyma05g09040

Glyma05g24930

Glyma05g28790

Glyma06g11680

Glyma06g13860

Glyma06g20870

Glyma09g04280

Glyma12g13100

Glyma14g02940

Glyma14g04350

Module15

Glyma03g34950

Glyma07g04500

Glyma07g32020

Glyma08g11850

Glyma08g25170

Glyma10g40150

Glyma13g40940

Glyma17g10490

Glyma19g02370

Module16

Glyma02g14410

Glyma04g05290

Glyma08g18080

Glyma09g02330

Glyma09g03400

Glyma11g07750

Glyma13g44870

Glyma14g06650

Glyma14g40680

Glyma15g14330

Glyma16g26630

Glyma19g44270

Module17

Glyma05g28810

Glyma12g34440

Glyma13g37830

Glyma19g03730

Glyma20g26600

Module18

Glyma01g45000

Glyma02g02560

Glyma03g15130

Glyma03g31950

Glyma04g27810

Glyma05g14760

Glyma06g01570

Glyma06g04140

Glyma08g00320

Glyma08g17230

Glyma09g14380

Glyma09g38990

Glyma17g07190

Glyma17g12150

Module19

Glyma16g07750

Module20

Glyma02g00340

Glyma03g04990

Glyma05g04500

Glyma07g04470

Glyma09g04630

Glyma14g09510

Glyma15g30110

Glyma17g02000

Glyma17g11940

Glyma20g30910

Module21

Glyma02g01950

Glyma03g40130

Glyma04g00450

Glyma05g32210

Glyma07g03220

Glyma07g38460

Glyma08g15480

Glyma08g45810

Glyma12g07050

Glyma12g30800

Glyma13g21050

Glyma17g08570

Glyma20g36880

Module22

Glyma02g11920

Glyma05g35030

Glyma08g04690

Glyma09g34760

Glyma13g42830

Glyma16g23580

Glyma20g38970

Module23

Glyma03g31470

Glyma06g15540

Glyma08g13790

Glyma08g21900

Glyma12g09730

Glyma13g35800

Glyma15g19510

Glyma17g05760

Glyma18g14410

Glyma20g21100

Glyma20g38720

Module24

Glyma01g26840

Glyma07g38110

Glyma08g11070

Glyma12g29510

Glyma19g32990

Module25

Glyma02g15780

Glyma03g38130

Glyma04g04240

Glyma04g21810

Glyma04g33010

Glyma05g30690

Glyma08g46610

Glyma10g28610

Glyma15g12200

Glyma15g38070

Module26

Glyma08g29090

Glyma09g08100

Glyma13g40100

Glyma16g04190

Glyma17g35720

Module27

Glyma01g04420

Glyma04g11130

Glyma06g05530

Glyma07g15690

Glyma07g31660

Glyma08g20190

Glyma08g48240

Glyma11g32600

Glyma14g00850

Glyma15g39090

Glyma17g11170

Glyma17g16620

Module28

Glyma01g28500

Glyma02g13850

Glyma02g17410

Glyma05g03010

Glyma06g17100

Glyma07g03820

Glyma07g32620

Glyma09g19790

Glyma11g05980

Glyma17g21540

Glyma19g39330

Module29

Glyma01g38520

Glyma02g02180

Glyma02g41220

Glyma03g36580

Glyma05g27690

Glyma08g06950

Glyma08g07890

Glyma08g48290

Glyma09g37570

Glyma11g02130

Glyma12g07130

Glyma13g35400

Glyma17g08700

Glyma20g02110

Module30

Glyma01g42230

Glyma02g00840

Glyma02g16710

Glyma03g29190

Glyma03g30420

Glyma04g05510

Glyma05g30290

Glyma06g47470

Glyma08g19180

Glyma10g08210

Glyma11g02640

Glyma12g02040

Glyma12g06300

Glyma12g34310

Glyma12g35370

Glyma13g32310

Glyma14g40200

Glyma15g35410

Glyma16g04960

Glyma16g31280

Glyma17g15690

Glyma20g38590

Module31

Glyma01g02580

Glyma02g03250

Glyma06g02540

Glyma06g47560

Glyma11g37680

Module32

Glyma01g04890

Glyma02g01250

Glyma02g14140

Glyma02g37510

Glyma02g42830

Glyma04g04880

Glyma04g40030

Glyma05g30180

Glyma06g03200

Glyma06g09880

Glyma07g15160

Glyma08g07470

Glyma08g25410

Glyma09g02860

Glyma09g32790

Glyma10g43060

Glyma11g00910

Glyma13g19080

Glyma13g44390

Glyma15g22820

Glyma18g00610

Glyma19g32550

Module33

Glyma03g42310

Glyma04g02240

Glyma05g00620

Glyma05g25810

Glyma06g02300

Glyma07g06660

Glyma08g19210

Glyma10g39460

Glyma11g18640

Glyma13g23090

Glyma15g03050

Glyma16g25860

Glyma17g10010

Glyma20g28300

Module34

Glyma08g13100

Glyma09g07120

Glyma15g06210

Glyma17g00710

Glyma17g18800

Glyma19g22730

Module35

Glyma02g10550

Glyma02g41230

Glyma02g42130

Glyma03g39570

Glyma04g42930

Glyma06g17490

Glyma07g12190

Glyma10g37980

Glyma15g01270

Glyma17g00230

Glyma18g29500

Module36

Glyma01g03180

Glyma01g03570

Glyma02g42080

Glyma02g44330

Glyma03g32980

Glyma03g37340

Glyma04g00660

Glyma04g32950

Glyma04g40430

Glyma05g02570

Glyma05g03880

Glyma05g21820

Glyma06g06790

Glyma06g14080

Glyma08g15000

Glyma08g17600

Glyma08g19320

Glyma10g43770

Glyma12g13240

Glyma13g33410

Glyma13g41960

Glyma16g00360

Glyma17g15230

Glyma18g48620

Glyma19g35690

Glyma20g28780

Module37

Glyma04g07070

Glyma06g46750

Glyma08g05220

Glyma08g17200

Glyma12g31990

Glyma13g30820

Glyma13g38490

Module38

Glyma02g36700

Glyma06g07780

Glyma06g20370

Glyma14g07290

Module39

Glyma02g04820

Glyma03g19260

Glyma05g32160

Glyma06g36520

Glyma09g22310

Glyma11g12790

Glyma13g19500

Glyma13g27130

Glyma14g35340

Glyma15g06790

Glyma19g33330

Module40

Glyma01g39810

Glyma04g04400

Glyma11g00670

Glyma11g01660

Glyma11g19480

Glyma13g39590

Glyma15g01160

Glyma15g15990

Glyma16g09020

Glyma19g05980

Glyma19g28220

Module41

Glyma01g38040

Glyma01g42370

Glyma01g42800

Glyma03g34440

Glyma03g35950

Glyma04g02230

Glyma05g02490

Glyma05g25460

Glyma07g32590

Glyma07g37270

Glyma08g08170

Glyma08g17270

Glyma08g24680

Glyma08g24720

Glyma08g43240

Glyma09g32630

Glyma09g36620

Glyma10g02090

Glyma10g31280

Glyma10g44170

Glyma11g03310

Glyma11g34380

Glyma12g02240

Glyma13g00370

Glyma13g22350

Glyma13g23770

Glyma14g06900

Glyma15g06780

Glyma15g35390

Glyma16g04410

Glyma17g02080

Glyma17g07440

Glyma18g02090

Glyma18g06230

Glyma19g45260

Glyma20g01420

Glyma20g34830

Module42

Glyma02g37080

Glyma02g40290

Glyma06g02330

Glyma06g04760

Glyma10g35520

Glyma11g01520

Glyma12g06910

Glyma13g44170

Module43

Glyma05g22180

Glyma09g28490

Glyma10g38070

Glyma11g07490

Glyma13g34520

Glyma16g28590

Module44

Glyma01g36410

Glyma03g00470

Glyma03g34700

Glyma04g01270

Glyma04g40980

Glyma04g40990

Glyma06g14250

Glyma06g45220

Glyma07g33800

Glyma08g10540

Glyma08g18860

Glyma08g20130

Glyma08g22550

Glyma09g07410

Glyma09g31560

Glyma11g10860

Glyma11g12180

Glyma11g35450

Glyma12g04380

Glyma13g20330

Glyma13g31900

Glyma13g36730

Glyma14g40120

Glyma15g42940

Glyma16g03170

Glyma16g22060

Glyma17g04830

Glyma18g50150

Glyma19g35780

Module45

Glyma04g01380

Glyma05g24110

Glyma05g29000

Glyma08g25950

Glyma08g46860

Glyma11g11290

Glyma11g33560

Glyma12g03470

Glyma18g08220

Glyma18g51980

Glyma20g27280

Module46

Glyma02g45690

Glyma03g32830

Glyma04g00710

Glyma07g31310

Glyma08g36350

Glyma08g45610

Glyma09g41460

Glyma12g29100

Glyma15g09530

Glyma20g05700

Module47

Glyma01g01310

Glyma01g45440

Glyma02g39090

Glyma05g28770

Glyma06g03470

Glyma07g02500

Glyma13g17420

Glyma15g41640

Glyma16g03020

Glyma18g45420

Module48

Glyma01g06030

Glyma02g04600

Glyma05g34420

Glyma06g44810

Glyma07g00750

Glyma07g13880

Glyma08g06040

Glyma09g41070

Glyma11g00300

Glyma11g10310

Glyma11g21150

Glyma13g03170

Glyma13g20490

Glyma13g26550

Glyma15g20680

Glyma17g01010

Glyma17g15420

Glyma18g18880

Module49

Glyma04g06890

Glyma05g01680

Glyma06g04980

Glyma08g17240

Glyma09g02040

Glyma10g30440

Glyma10g40250

Glyma13g07900

Glyma13g17650

Glyma13g27060

Glyma15g11540

Glyma16g04560

Glyma18g10270

Glyma18g10930

Glyma18g47360

Glyma18g50940

Glyma19g43150

Glyma20g14390

Glyma20g26580

Genes with sequence CTCTT

Module1

Glyma02g11720

Glyma03g21540

Glyma03g34760

Glyma04g06410

Glyma04g07270

Glyma07g11390

Glyma07g16490

Glyma07g35640

Glyma10g07550

Glyma11g11310

Glyma12g04960

Glyma15g03710

Glyma17g03050

Glyma20g31250

Module2

Glyma01g39460

Glyma06g02290

Glyma08g38740

Glyma09g05340

Glyma10g05800

Glyma16g27900

Glyma17g14620

Glyma19g37230

Glyma19g37240

Glyma20g34820

Module3

Glyma01g39350

Glyma03g38520

Glyma05g28730

Glyma06g47890

Glyma08g41220

Glyma09g03020

Glyma09g12320

Glyma09g14090

Glyma09g27100

Glyma10g34880

Glyma11g06870

Glyma12g10240

Glyma13g10640

Glyma13g39600

Glyma14g07690

Glyma16g27210

Glyma16g28080

Glyma18g52430

Glyma19g30600

Glyma20g30590

Module4

Glyma02g06730

Glyma02g15520

Glyma02g35190

Glyma02g42250

Glyma03g28080

Glyma03g36140

Glyma04g42120

Glyma05g15700

Glyma09g21820

Glyma10g07500

Glyma10g11060

Glyma11g04620

Glyma11g14300

Glyma12g02590

Glyma13g38710

Glyma14g05840

Glyma15g15610

Glyma16g05770

Glyma16g06740

Glyma18g15530

Glyma19g44060

Module5

Glyma02g35550

Glyma02g40440

Glyma03g24020

Glyma03g31620

Glyma04g40920

Glyma05g00640

Glyma05g08530

Glyma07g09530

Glyma08g44820

Glyma10g35220

Glyma11g02000

Glyma11g37990

Glyma14g01960

Glyma15g13560

Glyma17g08020

Glyma17g08250

Glyma17g35860

Glyma17g36100

Glyma17g36400

Glyma18g53860

Glyma20g23930

Module6

Glyma02g12970

Glyma04g01920

Glyma04g10880

Glyma05g03310

Glyma05g24850

Glyma05g36100

Glyma06g03100

Glyma08g20060

Glyma08g23870

Glyma08g29130

Glyma08g39390

Glyma08g44130

Glyma09g00670

Glyma09g23600

Glyma09g39230

Glyma09g39530

Glyma11g18980

Glyma13g00380

Glyma13g05120

Glyma15g17680

Glyma15g41960

Glyma18g08630

Glyma18g47730

Glyma20g38980

Module7

Glyma01g40150

Glyma02g02690

Glyma02g44460

Glyma03g29810

Glyma03g31460

Glyma03g39480

Glyma04g08570

Glyma05g04520

Glyma05g27600

Glyma06g46180

Glyma06g47520

Glyma07g38520

Glyma08g04990

Glyma08g17000

Glyma08g24950

Glyma09g06930

Glyma09g28440

Glyma10g24620

Glyma10g29600

Glyma10g42940

Glyma13g18230

Glyma13g21520

Glyma14g17930

Glyma15g40520

Glyma17g23830

Glyma18g02970

Glyma18g18050

Glyma18g26190

Glyma18g49340

Glyma19g25930

Glyma19g39940

Glyma20g09810

Glyma20g29190

Module8

Glyma02g47170

Glyma07g09710

Glyma08g12650

Glyma08g48030

Glyma11g19130

Glyma11g37620

Glyma12g01130

Glyma13g23850

Glyma13g28970

Glyma15g01500

Glyma17g35230

Module9

Glyma02g09190

Glyma02g44710

Glyma02g46330

Glyma04g00350

Glyma04g04540

Glyma04g40000

Glyma05g02670

Glyma05g05820

Glyma06g18470

Glyma07g16970

Glyma08g01410

Glyma08g18310

Glyma08g47310

Glyma08g47990

Glyma09g31690

Glyma09g36120

Glyma11g20600

Glyma12g28970

Glyma13g38730

Glyma15g01950

Glyma19g38690

Glyma19g42260

Module10

Glyma01g41670

Glyma05g37730

Glyma11g03690

Glyma11g14140

Glyma12g00390

Glyma17g37400

Module11

Glyma04g03020

Glyma05g00400

Glyma05g27190

Glyma06g07140

Glyma06g09420

Glyma08g14130

Glyma11g08440

Glyma11g33720

Glyma15g12530

Glyma16g23730

Glyma18g04500

Glyma18g52860

Glyma19g37500

Glyma20g12250

Glyma20g29660

Glyma20g34880

Module12

Glyma01g26950

Glyma02g05540

Glyma02g11540

Glyma02g38450

Glyma02g43790

Glyma02g47210

Glyma03g37190

Glyma03g40280

Glyma04g06700

Glyma04g07220

Glyma04g11400

Glyma04g36860

Glyma04g39380

Glyma05g01180

Glyma05g31760

Glyma05g34070

Glyma05g34570

Glyma06g18120

Glyma08g03150

Glyma08g05610

Glyma09g24070

Glyma13g19330

Glyma13g19470

Glyma13g28830

Glyma14g36970

Glyma15g10950

Glyma16g08460

Glyma18g14980

Glyma19g39070

Glyma19g39800

Glyma20g30970

Glyma20g38480

Module13

Glyma09g01320

Glyma09g04950

Glyma10g35870

Glyma15g12170

Glyma17g01720

Module14

Glyma01g01390

Glyma01g29950

Glyma02g40430

Glyma02g44080

Glyma03g32150

Glyma04g03290

Glyma04g33570

Glyma04g33750

Glyma05g00440

Glyma06g11680

Glyma06g20870

Glyma08g08910

Glyma08g18460

Glyma09g04280

Glyma10g31550

Glyma11g19780

Glyma12g13100

Glyma13g28840

Glyma15g06890

Glyma17g07060

Glyma17g29080

Glyma17g36970

Glyma20g22850

Glyma20g26530

Module15

Glyma01g02400

Glyma01g24950

Glyma03g34950

Glyma03g40760

Glyma04g03110

Glyma05g01390

Glyma07g04500

Glyma08g23860

Glyma10g02370

Glyma10g38760

Glyma10g40150

Glyma13g20800

Glyma13g40940

Glyma15g13970

Glyma19g02370

Glyma20g32000

Module16

Glyma02g04760

Glyma02g38750

Glyma02g41590

Glyma03g14210

Glyma04g34160

Glyma06g36590

Glyma08g18080

Glyma09g02330

Glyma09g36720

Glyma13g17570

Glyma14g05850

Glyma14g06640

Glyma14g06650

Glyma14g40680

Glyma15g16560

Glyma17g35650

Glyma18g47930

Glyma18g50180

Glyma20g29200

Module17

Glyma07g37280

Glyma10g02080

Glyma13g39240

Glyma15g13880

Glyma19g03730

Glyma20g04130

Glyma20g26600

Glyma20g29490

Module18

Glyma01g07070

Glyma01g45000

Glyma02g02560

Glyma03g15130

Glyma03g31950

Glyma04g40350

Glyma06g04140

Glyma07g39630

Glyma08g00320

Glyma08g11960

Glyma08g25150

Glyma09g01390

Glyma09g38990

Glyma13g06050

Glyma13g33890

Glyma13g40470

Glyma15g02700

Glyma15g03460

Glyma15g41970

Glyma17g07190

Glyma17g12150

Glyma18g50760

Glyma19g33730

Glyma20g00760

Module19

Glyma10g36690

Glyma17g14850

Module20

Glyma01g38650

Glyma02g01990

Glyma06g16810

Glyma07g03910

Glyma09g04530

Glyma09g04630

Glyma09g37910

Glyma10g32340

Glyma10g39450

Glyma12g06950

Glyma12g08520

Glyma13g42340

Glyma15g06000

Glyma16g27880

Glyma18g49240

Glyma19g01940

Glyma20g30910

Module21

Glyma02g01950

Glyma03g22260

Glyma03g40130

Glyma05g32210

Glyma07g38460

Glyma08g15480

Glyma08g19290

Glyma08g24380

Glyma08g45810

Glyma10g11620

Glyma11g13940

Glyma16g01650

Glyma16g09760

Glyma17g07070

Glyma17g17970

Module22

Glyma07g00700

Glyma07g06580

Glyma08g04690

Glyma08g10970

Glyma08g44210

Glyma11g15230

Glyma12g03230

Glyma12g04020

Glyma12g07160

Glyma12g08050

Glyma13g42830

Glyma14g06820

Glyma15g23220

Glyma16g01460

Glyma17g03550

Module23

Glyma04g01020

Glyma04g12510

Glyma04g33360

Glyma06g15540

Glyma06g20960

Glyma08g22850

Glyma10g32080

Glyma12g09730

Glyma15g10890

Glyma15g19510

Glyma15g43100

Glyma17g13930

Glyma19g25720

Glyma20g38720

Module24

Glyma08g00780

Glyma10g06600

Glyma10g15980

Glyma12g29510

Glyma16g33710

Module25

Glyma01g04380

Glyma01g41270

Glyma02g37310

Glyma03g33560

Glyma03g38130

Glyma04g21810

Glyma05g30690

Glyma07g05620

Glyma07g16850

Glyma07g38580

Glyma08g46610

Glyma09g02590

Glyma09g15090

Glyma11g10340

Glyma13g30590

Glyma14g35660

Glyma16g02490

Glyma17g03390

Glyma17g15860

Glyma18g16720

Glyma18g44250

Glyma20g07060

Glyma20g32140

Module26

Glyma04g01130

Glyma09g08100

Glyma13g40100

Glyma15g19580

Glyma15g21890

Glyma17g23870

Module27

Glyma01g04420

Glyma01g33170

Glyma02g40890

Glyma03g02580

Glyma05g04400

Glyma06g05530

Glyma07g31660

Glyma08g20190

Glyma09g08470

Glyma10g08300

Glyma10g35310

Glyma11g32600

Glyma12g04940

Glyma13g27010

Glyma15g11700

Glyma16g26940

Glyma17g11170

Glyma17g16620

Glyma19g28770

Glyma20g34430

Module28

Glyma01g28500

Glyma02g13850

Glyma02g17410

Glyma04g00280

Glyma05g06570

Glyma06g48170

Glyma07g32620

Glyma08g25830

Glyma08g43950

Glyma11g05980

Glyma11g15840

Glyma13g23310

Glyma13g31460

Glyma13g44790

Glyma15g04350

Glyma17g21540

Glyma18g53370

Glyma19g39330

Module29

Glyma01g39770

Glyma02g02180

Glyma02g41220

Glyma03g30720

Glyma04g38260

Glyma08g46190

Glyma10g04590

Glyma11g25650

Glyma12g07130

Glyma12g15800

Glyma12g17150

Glyma13g02870

Glyma15g16630

Glyma17g10340

Glyma19g33700

Glyma20g04230

Glyma20g29980

Module30

Glyma01g42230

Glyma03g15800

Glyma03g36620

Glyma03g37390

Glyma04g05510

Glyma04g12600

Glyma05g30290

Glyma06g01270

Glyma06g12010

Glyma06g15410

Glyma06g18560

Glyma06g40620

Glyma06g47190

Glyma06g47470

Glyma07g17170

Glyma07g34010

Glyma08g19180

Glyma08g20220

Glyma09g03450

Glyma09g09430

Glyma10g07410

Glyma10g08010

Glyma11g27480

Glyma11g27720

Glyma12g00980

Glyma12g02040

Glyma12g06300

Glyma13g01900

Glyma13g23760

Glyma13g32310

Glyma15g04930

Glyma15g07700

Glyma15g13870

Glyma15g14210

Glyma15g24760

Glyma16g04960

Glyma16g04980

Glyma16g05710

Glyma16g22920

Glyma16g31280

Glyma17g12160

Glyma17g15690

Glyma19g28520

Glyma20g01370

Module31

Glyma01g02580

Glyma02g03250

Glyma06g47560

Glyma07g09840

Glyma09g24450

Glyma19g42940

Module32

Glyma02g01910

Glyma02g42830

Glyma02g46580

Glyma03g04330

Glyma03g34480

Glyma04g40030

Glyma05g34530

Glyma06g03200

Glyma06g20360

Glyma06g45180

Glyma07g15160

Glyma08g07470

Glyma08g07660

Glyma08g09730

Glyma08g19270

Glyma09g02860

Glyma09g32790

Glyma11g00910

Glyma11g15650

Glyma12g09810

Glyma13g05830

Glyma15g19000

Glyma15g22820

Glyma17g04430

Glyma17g07530

Glyma20g02500

Module33

Glyma01g28810

Glyma01g38750

Glyma02g16000

Glyma02g45190

Glyma03g08280

Glyma03g42310

Glyma04g02240

Glyma04g05800

Glyma05g00620

Glyma05g25810

Glyma06g05810

Glyma07g00890

Glyma08g08770

Glyma08g43330

Glyma13g23090

Glyma0048s00300

Module34

Glyma08g13100

Glyma08g23940

Glyma11g13500

Glyma12g19050

Glyma14g05980

Glyma17g00710

Glyma18g06560

Glyma19g28430

Glyma19g34300

Module35

Glyma02g01560

Glyma02g41230

Glyma02g42270

Glyma04g42930

Glyma05g31290

Glyma06g09560

Glyma06g17490

Glyma07g12190

Glyma08g14450

Glyma10g01720

Glyma10g26790

Glyma11g06440

Glyma11g12540

Glyma12g06770

Glyma13g35750

Glyma13g41450

Glyma16g04090

Glyma17g00230

Glyma18g22780

Glyma18g29500

Glyma20g02760

Module36

Glyma01g00740

Glyma01g03180

Glyma01g41620

Glyma02g00540

Glyma02g04400

Glyma02g10170

Glyma02g42080

Glyma03g32980

Glyma03g37340

Glyma04g00660

Glyma04g32950

Glyma05g30780

Glyma06g06790

Glyma07g15320

Glyma08g03480

Glyma08g17600

Glyma08g21960

Glyma09g38590

Glyma10g36610

Glyma11g11020

Glyma11g20570

Glyma12g13240

Glyma13g16500

Glyma14g01530

Glyma14g09300

Glyma17g08630

Glyma17g15230

Glyma18g48620

Glyma19g06460

Glyma19g35690

Glyma19g37520

Glyma19g40080

Module37

Glyma01g39410

Glyma05g22380

Glyma06g10570

Glyma06g34330

Glyma06g46810

Glyma08g05220

Glyma08g17200

Glyma12g31990

Glyma13g30820

Glyma13g38490

Glyma17g01920

Module38

Glyma02g36700

Glyma05g33340

Glyma06g20370

Glyma11g19980

Glyma12g05180

Glyma13g31580

Glyma14g07290

Glyma15g06140

Module39

Glyma01g42670

Glyma02g04820

Glyma02g08950

Glyma06g36520

Glyma10g37200

Glyma11g12790

Glyma13g20170

Glyma13g39890

Glyma14g35340

Glyma15g06790

Glyma15g17530

Glyma16g01960

Glyma16g33270

Glyma17g08900

Glyma19g33330

Module40

Glyma03g30440

Glyma08g02550

Glyma08g04240

Glyma08g26880

Glyma08g43690

Glyma10g30650

Glyma13g24180

Glyma13g39590

Glyma13g41700

Glyma13g41710

Glyma15g15990

Glyma15g34870

Glyma16g09020

Glyma19g05980

Glyma19g28220

Module41

Glyma01g05050

Glyma01g38040

Glyma02g09540

Glyma02g16800

Glyma02g36580

Glyma02g40010

Glyma02g40200

Glyma03g34440

Glyma03g34560

Glyma03g37400

Glyma03g40910

Glyma04g00210

Glyma04g13990

Glyma04g42300

Glyma05g25460

Glyma05g36290

Glyma06g07160

Glyma06g12510

Glyma06g18660

Glyma06g42040

Glyma06g47690

Glyma07g00870

Glyma07g03120

Glyma07g04340

Glyma07g08280

Glyma07g37270

Glyma07g38620

Glyma08g04370

Glyma08g08170

Glyma08g21190

Glyma08g43240

Glyma08g43550

Glyma09g00800

Glyma09g00850

Glyma09g24130

Glyma09g32630

Glyma10g03000

Glyma10g04150

Glyma10g10240

Glyma10g31280

Glyma10g32070

Glyma10g38360

Glyma10g40350

Glyma11g03310

Glyma11g03940

Glyma11g10080

Glyma11g10760

Glyma11g20710

Glyma11g22090

Glyma11g29920

Glyma11g35560

Glyma12g03050

Glyma12g05310

Glyma12g30050

Glyma12g35710

Glyma13g00370

Glyma13g22350

Glyma13g23770

Glyma13g24560

Glyma13g25560

Glyma13g27300

Glyma15g02380

Glyma15g11220

Glyma15g13550

Glyma15g16710

Glyma16g01020

Glyma16g04410

Glyma16g06500

Glyma16g11370

Glyma17g04420

Glyma17g07440

Glyma18g02230

Glyma18g02870

Glyma18g06350

Glyma18g09290

Glyma18g39500

Glyma19g37630

Glyma19g40960

Glyma19g43370

Glyma20g01430

Glyma20g29210

Glyma20g30450

Module42

Glyma01g01180

Glyma02g37080

Glyma02g40290

Glyma04g00960

Glyma04g09350

Glyma05g31610

Glyma09g04480

Glyma10g30110

Glyma11g01520

Glyma12g06910

Glyma13g22940

Glyma18g00590

Glyma18g49400

Glyma19g30770

Glyma0169s00210

Module43

Glyma04g34840

Glyma08g00790

Glyma09g28490

Glyma13g24200

Glyma15g07040

Glyma17g03910

Glyma18g02220

Glyma18g52250

Glyma20g35630

Module44

Glyma03g34700

Glyma04g01270

Glyma05g00470

Glyma05g03850

Glyma06g14250

Glyma07g01540

Glyma07g02720

Glyma07g39980

Glyma08g18860

Glyma08g20130

Glyma09g31560

Glyma09g35650

Glyma11g02190

Glyma11g36090

Glyma12g30600

Glyma13g20330

Glyma16g03170

Glyma16g33230

Glyma17g04830

Glyma17g11430

Glyma18g01110

Glyma19g44920

Glyma20g03060

Glyma20g34330

Module45

Glyma02g09370

Glyma04g01380

Glyma08g25950

Glyma09g02790

Glyma11g10480

Glyma12g03470

Glyma17g09280

Glyma17g16830

Glyma19g35560

Glyma20g27280

Module46

Glyma01g35620

Glyma02g45690

Glyma03g32830

Glyma07g01740

Glyma08g45610

Glyma09g41460

Glyma18g12670

Module47

Glyma01g01310

Glyma01g27810

Glyma02g43640

Glyma06g05460

Glyma07g00540

Glyma08g01860

Glyma09g08340

Glyma09g40420

Glyma12g03510

Glyma13g11090

Glyma13g17420

Glyma14g16700

Glyma15g03430

Glyma16g03020

Glyma16g29370

Glyma18g08530

Glyma18g45420

Glyma18g53440

Glyma20g24670

Module48

Glyma01g06030

Glyma02g41430

Glyma03g00840

Glyma03g42140

Glyma05g37840

Glyma07g11560

Glyma11g10310

Glyma11g13750

Glyma12g04010

Glyma13g20490

Glyma13g22680

Glyma13g24340

Glyma15g40060

Glyma17g15420

Module49

Glyma01g07120

Glyma02g01570

Glyma03g24180

Glyma03g35000

Glyma04g06890

Glyma04g08000

Glyma04g35710

Glyma05g01680

Glyma05g02080

Glyma06g14200

Glyma08g08550

Glyma08g17240

Glyma08g26670

Glyma08g29920

Glyma09g02040

Glyma09g35760

Glyma10g23440

Glyma10g30020

Glyma11g14840

Glyma11g19300

Glyma11g37760

Glyma12g08820

Glyma12g10500

Glyma13g07900

Glyma13g17650

Glyma14g24140

Glyma14g27290

Glyma15g12930

Glyma16g04910

Glyma17g08970

Glyma18g10060

Glyma18g10930

Glyma18g50940

Glyma19g01400

Glyma19g26070

Glyma20g26580
