# Supplementary material for: Predicting gene regulatory networks of soybean nodulation from RNA-Seq transcriptome data
Source: BMC Bioinformatics. 2013 Sep 22;14:278. doi: 10.1186/1471-2105-14-278 (PMC3854569; doi:10.1186/1471-2105-14-278)
Supplement: Additional file 5 — Modules generated based on the 48-hour DEGs. [file 1471-2105-14-278-S5.doc]

**Supplemental Materials**

**The module 5,6,48,60 and 64 are predicted with GRAS family TFs. Among of them moduel 48 is with Leucine Zipper domain based on binding site analysis. The module 64 is with** bHLH-Zip domain**.**

**Module 1-70 are generated based on 48 hour DEGs with all included TF families.**

1


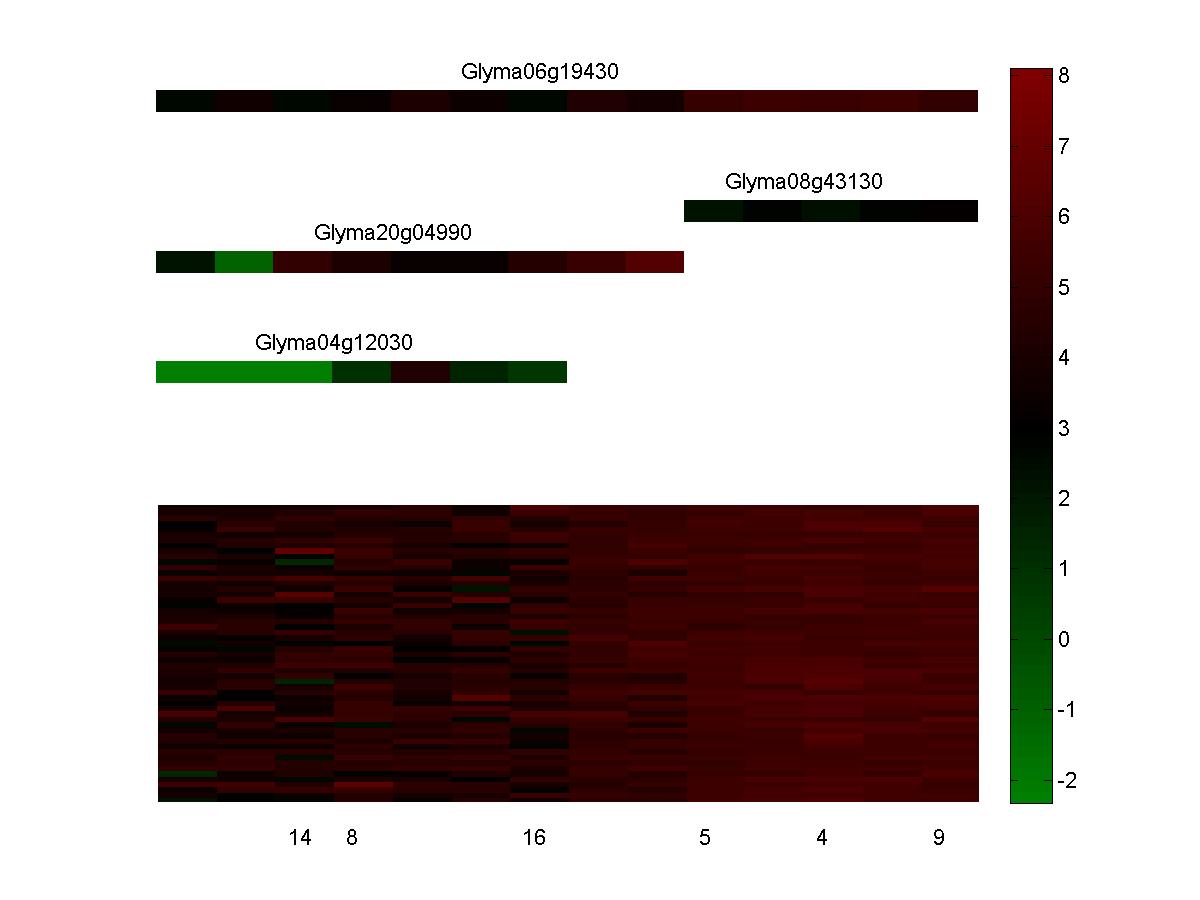


1 Glyma06g19430 TPR

1 Glyma20g04990 SRS

1 Glyma08g43130 C2H2 (Zn)

1 Glyma04g12030 AS2

Glyma01g39930 Glyma09g07040 Glyma15g14970 Glyma07g02180 Glyma05g24230

Glyma18g52590 Glyma08g16290 Glyma03g26090 Glyma10g05480 Glyma13g18640

Glyma01g40830 Glyma11g14840 Glyma14g04520 Glyma08g17240 Glyma18g42940

Glyma14g03970 Glyma18g15570 Glyma02g18320 Glyma06g21850 Glyma10g41560

Glyma11g36960 Glyma20g38980 Glyma13g01590 Glyma10g42480 Glyma07g07970

Glyma17g13760 Glyma10g30020 Glyma05g07480 Glyma06g48350 Glyma19g16450

Glyma18g46750 Glyma17g08970 Glyma15g31290 Glyma11g04040 Glyma17g05520

Glyma19g42050 Glyma02g11960 Glyma06g09600 Glyma12g06280 Glyma16g17500

Glyma12g04300 Glyma10g21350 Glyma20g34750 Glyma08g26670 Glyma20g24540

Glyma13g39390 Glyma14g05340 Glyma17g09700 Glyma20g37370 Glyma15g01520

Glyma12g29160 Glyma02g19870 Glyma16g02730 Glyma10g35890 Glyma01g38470

2


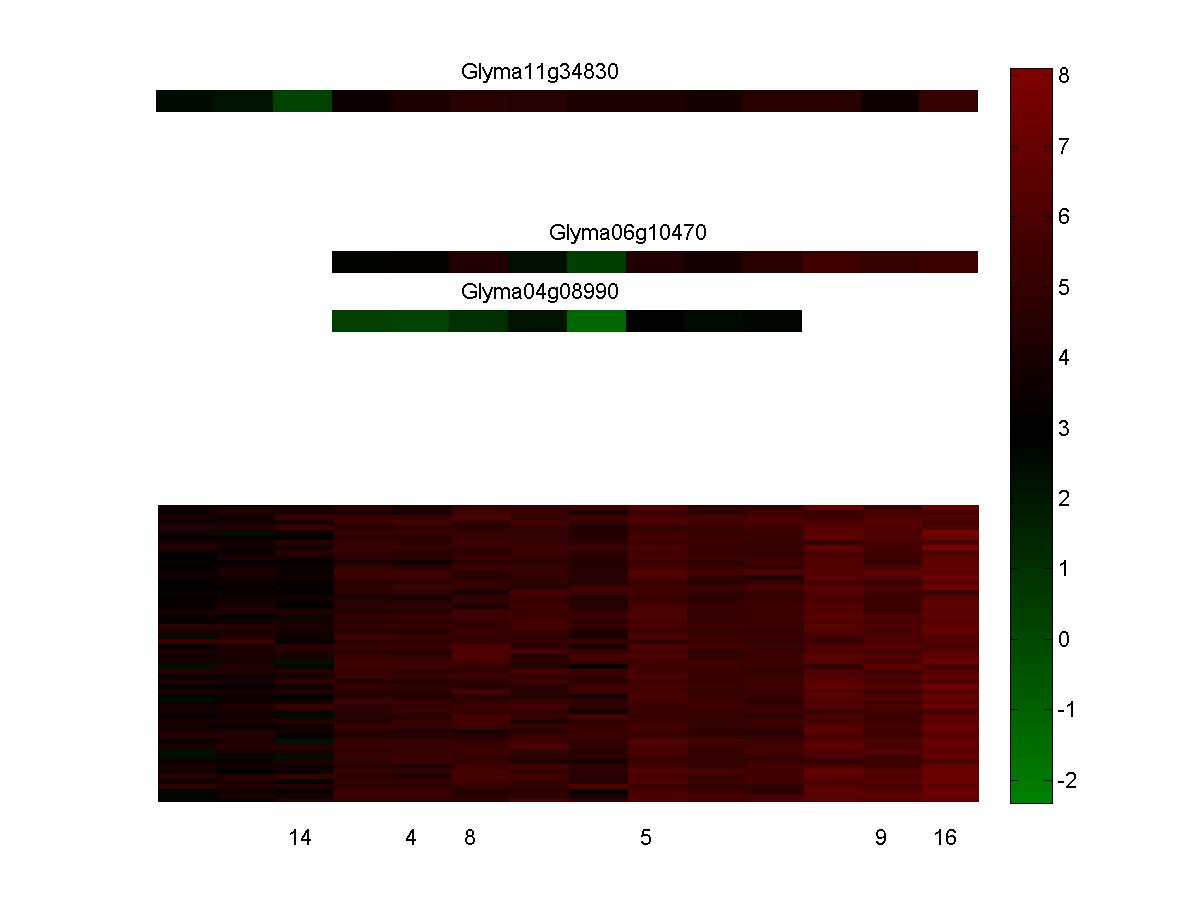


2 Glyma04g08990 C2C2 (Zn) GATA

2 Glyma11g34830 LIM

2 Glyma06g10470 bHLH

Glyma20g29190 Glyma04g37770 Glyma03g29810 Glyma15g03120 Glyma11g15090

Glyma11g35740 Glyma19g25930 Glyma10g01310 Glyma18g26190 Glyma12g04510

Glyma08g04990 Glyma15g13650 Glyma03g40130 Glyma10g01060 Glyma06g47520

Glyma11g37250 Glyma03g27570 Glyma18g52310 Glyma13g20310 Glyma06g46180

Glyma05g36600 Glyma08g02630 Glyma11g12080 Glyma10g31070 Glyma11g02950

Glyma16g05530 Glyma18g02340 Glyma05g03900 Glyma06g13870 Glyma10g29200

Glyma08g12030 Glyma04g40750 Glyma07g06830 Glyma20g35000 Glyma11g34890

Glyma13g40780 Glyma04g24380 Glyma18g02820 Glyma08g47920 Glyma19g35510

Glyma09g38230 Glyma06g17310 Glyma12g30800 Glyma17g07060 Glyma09g03480

Glyma10g00500 Glyma14g38950 Glyma06g47510 Glyma19g39940 Glyma20g22090

Glyma07g31840 Glyma02g36070 Glyma18g02050 Glyma10g36880 Glyma19g42090

Glyma05g24930 Glyma05g34680 Glyma02g45430 Glyma12g32000 Glyma19g28740

3


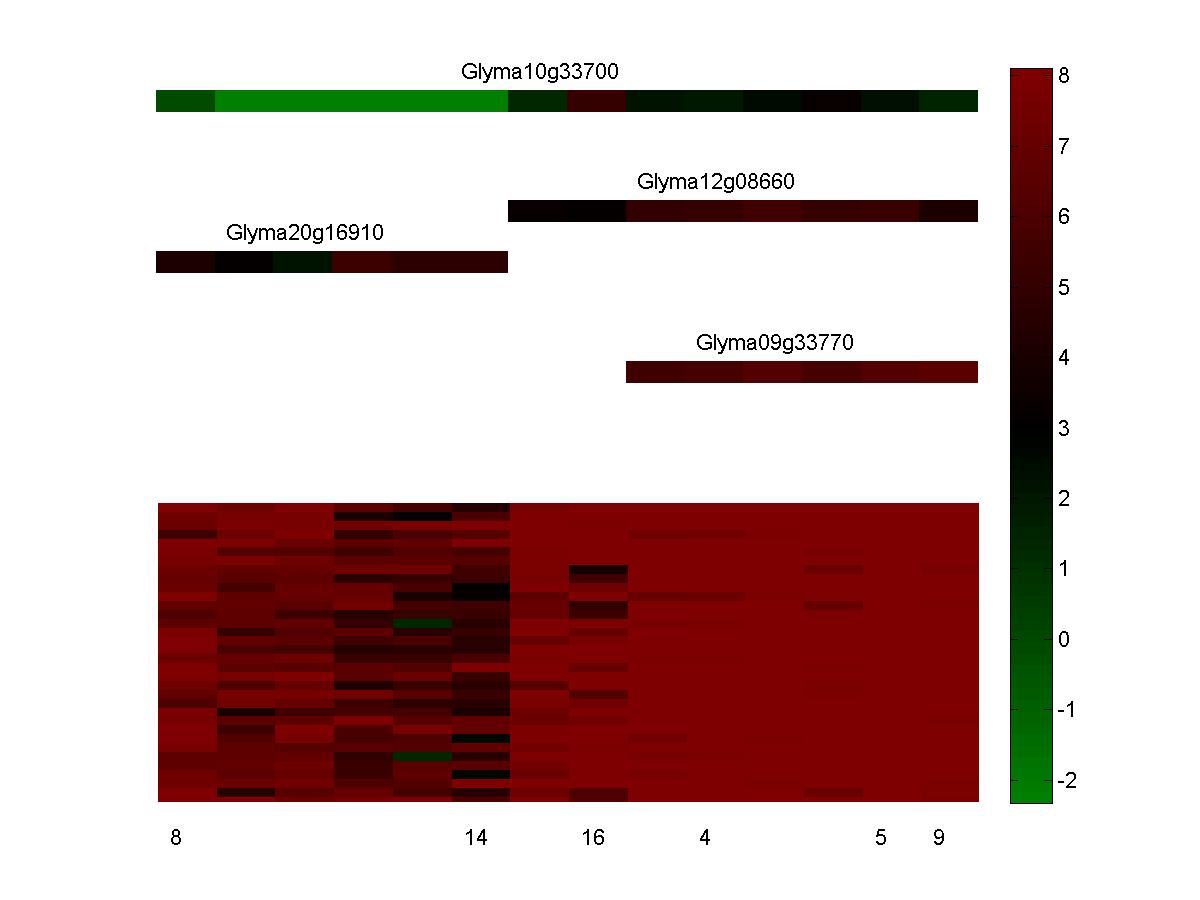


3 Glyma20g16910 AP2-EREBP

3 Glyma10g33700 AP2-EREBP

3 Glyma09g33770 PLATZ

3 Glyma12g08660 C2H2 (Zn)

Glyma10g40870 Glyma20g26440 Glyma11g05680 Glyma19g32990 Glyma14g04460

Glyma16g33710 Glyma14g00720 Glyma11g15680 Glyma07g33780 Glyma10g06600

Glyma18g35300 Glyma14g36850 Glyma08g11070 Glyma08g02940 Glyma08g23830

Glyma09g28310 Glyma04g02610 Glyma09g28320 Glyma10g35520 Glyma12g05150

Glyma06g11080 Glyma20g38560 Glyma02g40290 Glyma03g38190 Glyma12g29510

Glyma08g23560 Glyma11g33280 Glyma11g33560 Glyma09g02790 Glyma05g28490

Glyma06g04760 Glyma02g09200 Glyma16g28600 Glyma16g04950

4


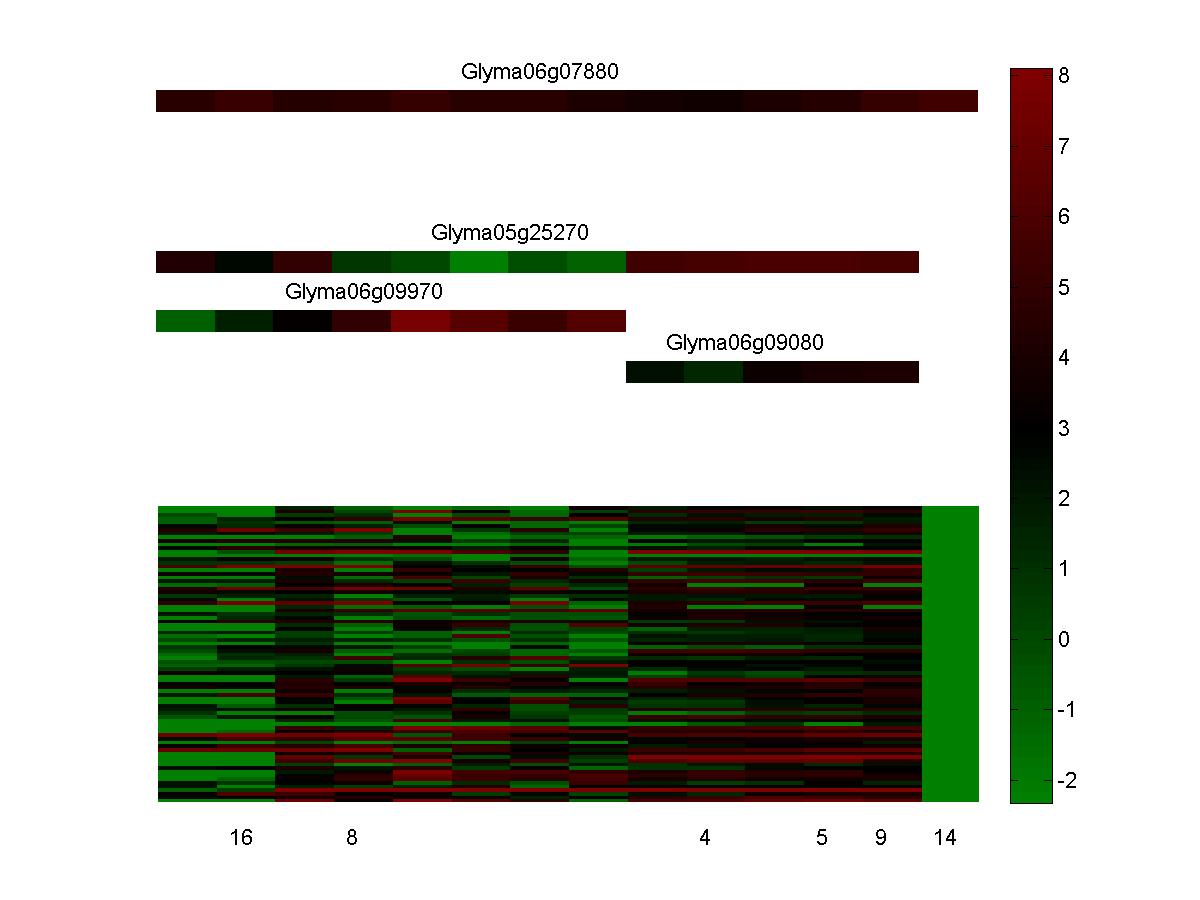


4 Glyma05g25270 WRKY

4 Glyma06g09080 C2C2 (Zn) GATA

4 Glyma06g07880 HTH-ARAC

4 Glyma06g09970 ZF-HD

Glyma15g15690 Glyma05g03580 Glyma20g24930 Glyma09g30420 Glyma12g09660

Glyma19g41730 Glyma11g18810 Glyma11g15020 Glyma12g06310 Glyma06g02810

Glyma08g23520 Glyma19g07080 Glyma18g06060 Glyma10g31780 Glyma05g26130

Glyma10g42100 Glyma15g38220 Glyma04g02380 Glyma02g43360 Glyma13g23250

Glyma13g07890 Glyma14g01960 Glyma15g13460 Glyma02g12020 Glyma02g11720

Glyma08g11100 Glyma07g37410 Glyma03g26660 Glyma13g39230 Glyma06g08170

Glyma05g04500 Glyma01g04420 Glyma04g02780 Glyma01g32140 Glyma13g19290

Glyma15g41940 Glyma20g32990 Glyma14g05680 Glyma15g09540 Glyma13g04850

Glyma06g01990 Glyma13g43970 Glyma06g03290 Glyma08g25090 Glyma07g09840

Glyma20g29790 Glyma13g34730 Glyma06g39810 Glyma07g18080 Glyma15g01370

Glyma08g45610 Glyma14g05650 Glyma10g43800 Glyma10g01300 Glyma15g38210

Glyma12g36300 Glyma20g34670 Glyma13g42640 Glyma02g01990 Glyma11g37620

Glyma02g40520 Glyma04g06430 Glyma06g09080 Glyma13g02230 Glyma05g04490

Glyma06g02540 Glyma06g09970 Glyma06g47560 Glyma09g01750 Glyma13g08060

Glyma01g32750 Glyma05g26120 Glyma03g29330 Glyma15g01100 Glyma15g01500

Glyma15g02790 Glyma04g40130 Glyma09g41460 Glyma12g08160 Glyma16g06640

Glyma19g38000

5


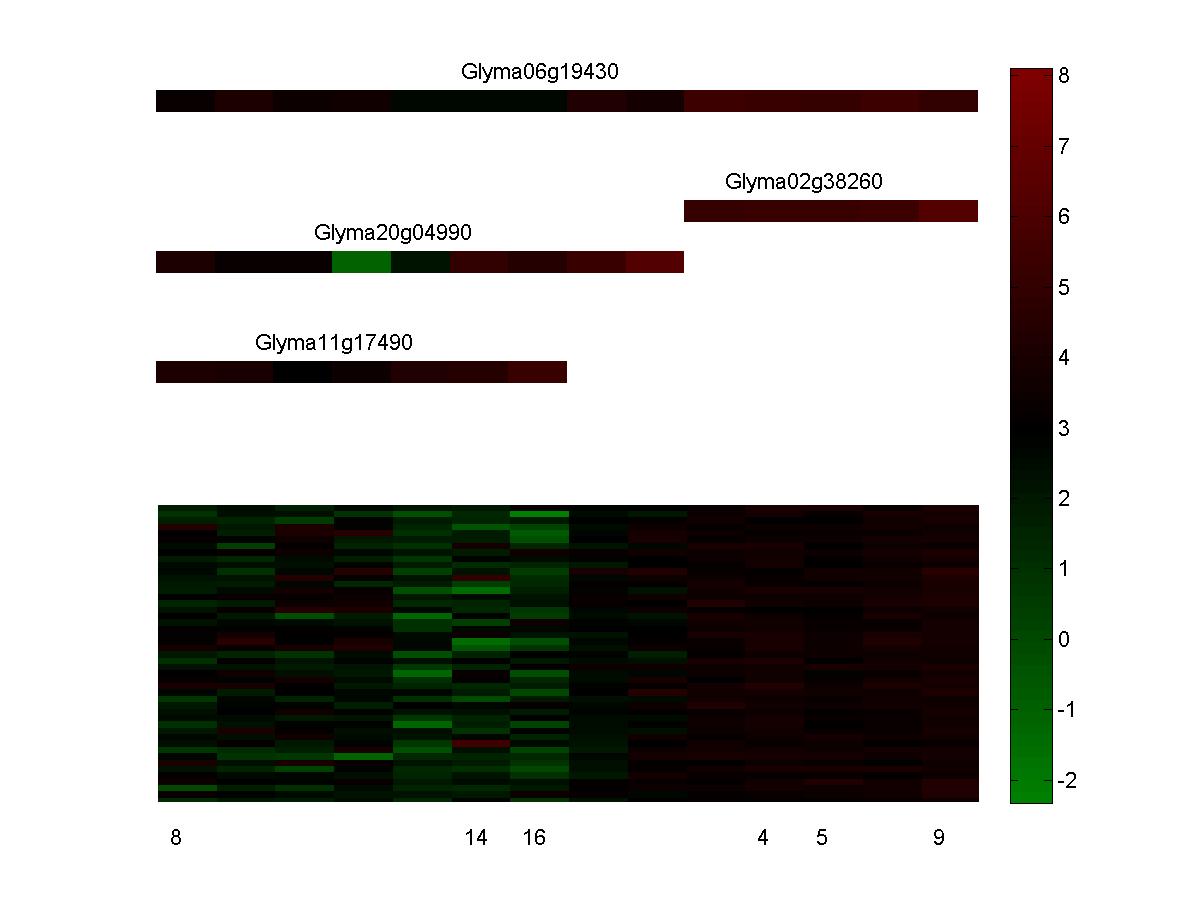


5 Glyma06g19430 TPR

5 Glyma20g04990 SRS

5 Glyma11g17490 GRAS

5 Glyma02g38260 AUX-IAA-ARF

Glyma11g03460 Glyma11g04520 Glyma11g04150 Glyma08g22820 Glyma05g08970

Glyma20g26770 Glyma05g08530 Glyma04g43270 Glyma15g13700 Glyma14g05000

Glyma17g11000 Glyma10g34710 Glyma16g26640 Glyma11g11430 Glyma19g31420

Glyma05g36430 Glyma04g38020 Glyma17g07770 Glyma06g00290 Glyma18g18230

Glyma04g07000 Glyma08g02470 Glyma06g23570 Glyma02g02630 Glyma15g24060

Glyma10g13700 Glyma09g05200 Glyma04g35580 Glyma07g39090 Glyma05g24130

Glyma02g38940 Glyma20g34920 Glyma12g05760 Glyma02g07620 Glyma03g16440

Glyma10g29980 Glyma16g34560 Glyma18g50410 Glyma16g05700 Glyma01g32450

Glyma01g08540 Glyma03g00410 Glyma20g01260 Glyma20g20280 Glyma02g33780

Glyma12g30430 Glyma08g47120

6


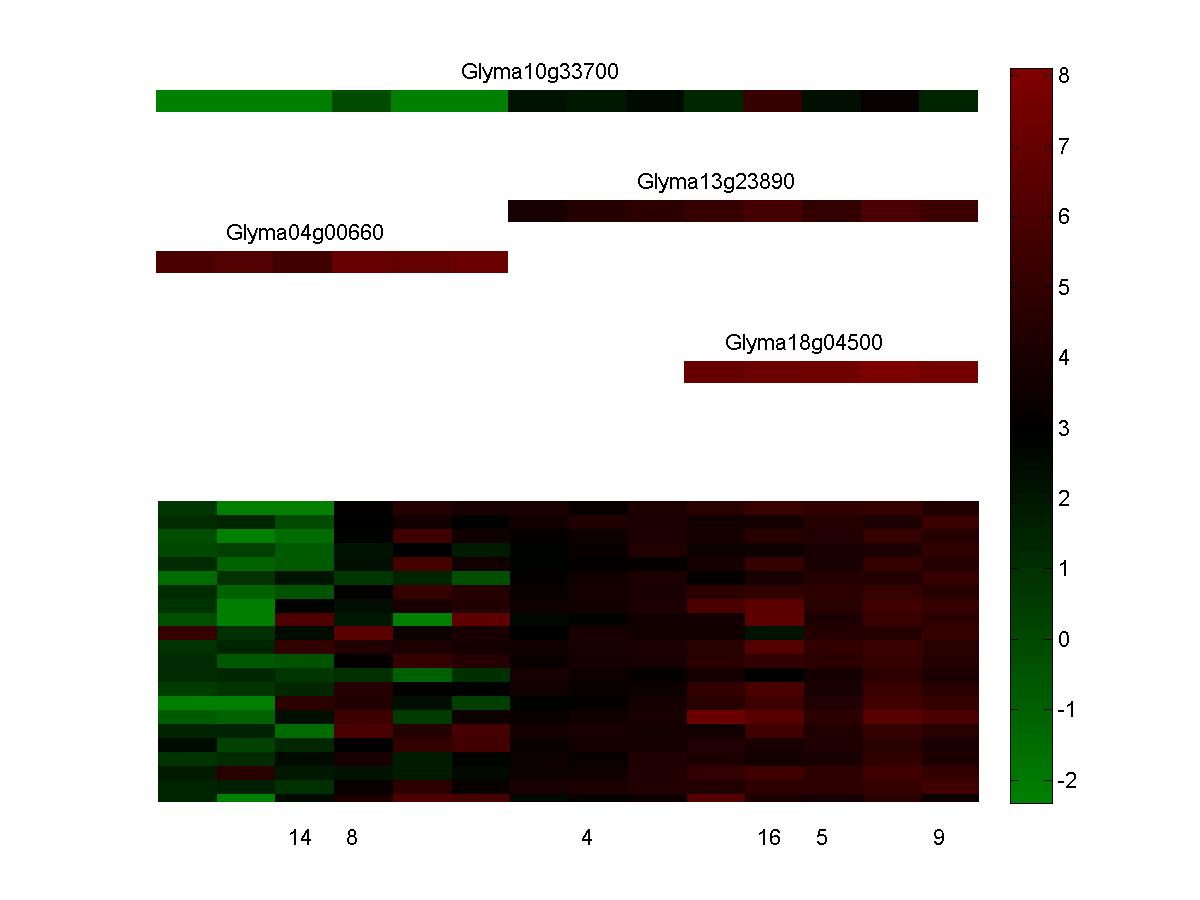


6 Glyma10g33700 AP2-EREBP

6 Glyma13g23890 Homeodomain/HOMEOBOX

6 Glyma04g00660 CSD

6 Glyma18g04500 GRAS

Glyma08g17010 Glyma13g38310 Glyma20g02110 Glyma06g10470 Glyma16g23380

Glyma07g39160 Glyma11g21480 Glyma13g41700 Glyma15g43170 Glyma14g00780

Glyma11g24140 Glyma13g42150 Glyma18g07160 Glyma01g03190 Glyma13g41710

Glyma18g07550 Glyma15g03710 Glyma16g29350 Glyma15g06130 Glyma01g03650

Glyma16g26630 Glyma11g00710

7


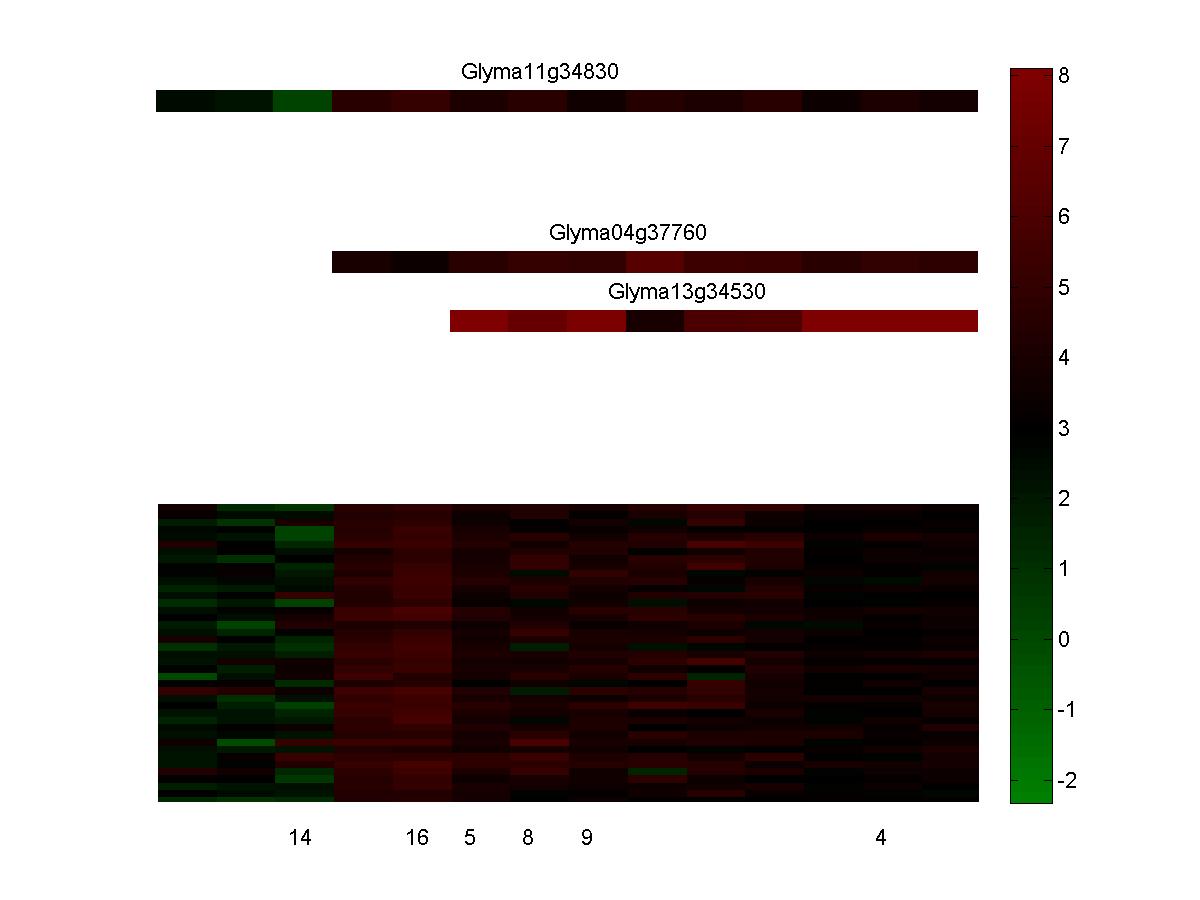


7 Glyma04g37760 AUX-IAA-ARF

7 Glyma11g34830 LIM

7 Glyma13g34530 C2H2 (Zn)

Glyma01g45480 Glyma03g39390 Glyma13g29330 Glyma11g21140 Glyma09g40260

Glyma01g33650 Glyma04g09260 Glyma18g52050 Glyma03g25520 Glyma11g34830

Glyma15g16630 Glyma09g05310 Glyma17g26600 Glyma05g27260 Glyma01g41210

Glyma03g37280 Glyma15g10980 Glyma01g38520 Glyma07g37600 Glyma02g41470

Glyma10g40670 Glyma17g00230 Glyma06g13260 Glyma11g38010 Glyma18g13270

Glyma09g38100 Glyma12g32390 Glyma07g12840 Glyma20g04230 Glyma20g31890

Glyma03g29860 Glyma16g26140 Glyma04g35980 Glyma10g26790 Glyma01g38070

Glyma01g4416 Glyma03g38710 Glyma12g29340 Glyma11g1066 Glyma08g48040

Glyma02g03950

8


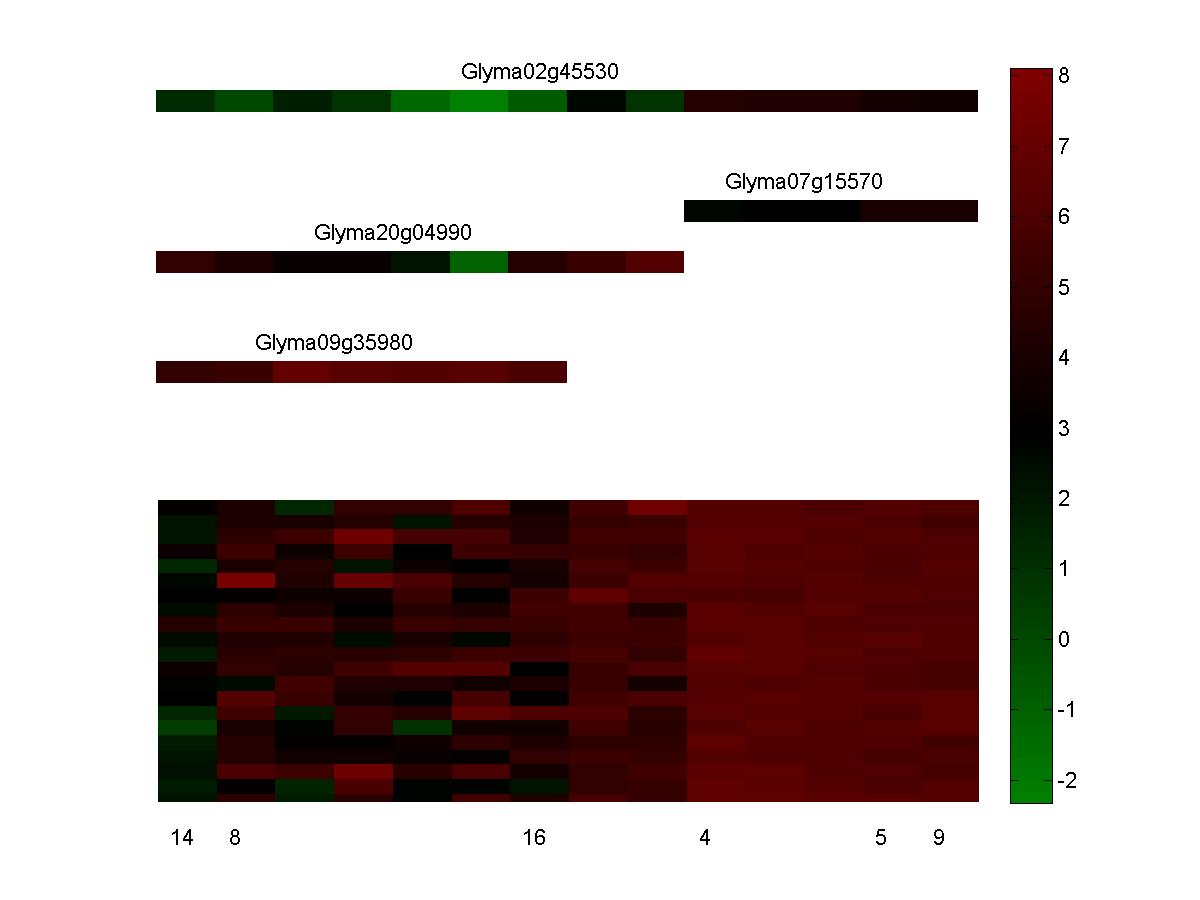


8 Glyma02g45530 WRKY

8 Glyma20g04990 SRS

8 Glyma09g35980 C3H-type1(Zn)

8 Glyma07g15570 SSB protein

Glyma10g26690 Glyma09g38990 Glyma16g29330 Glyma05g01400 Glyma02g40300

Glyma08g16300 Glyma09g36000 Glyma17g33420 Glyma02g46590 Glyma12g06540

Glyma10g43760 Glyma15g09820 Glyma09g34640 Glyma07g05230 Glyma08g11960

Glyma19g01100 Glyma18g44830 Glyma04g27810 Glyma09g39230 Glyma20g00760

Glyma08g41120

9


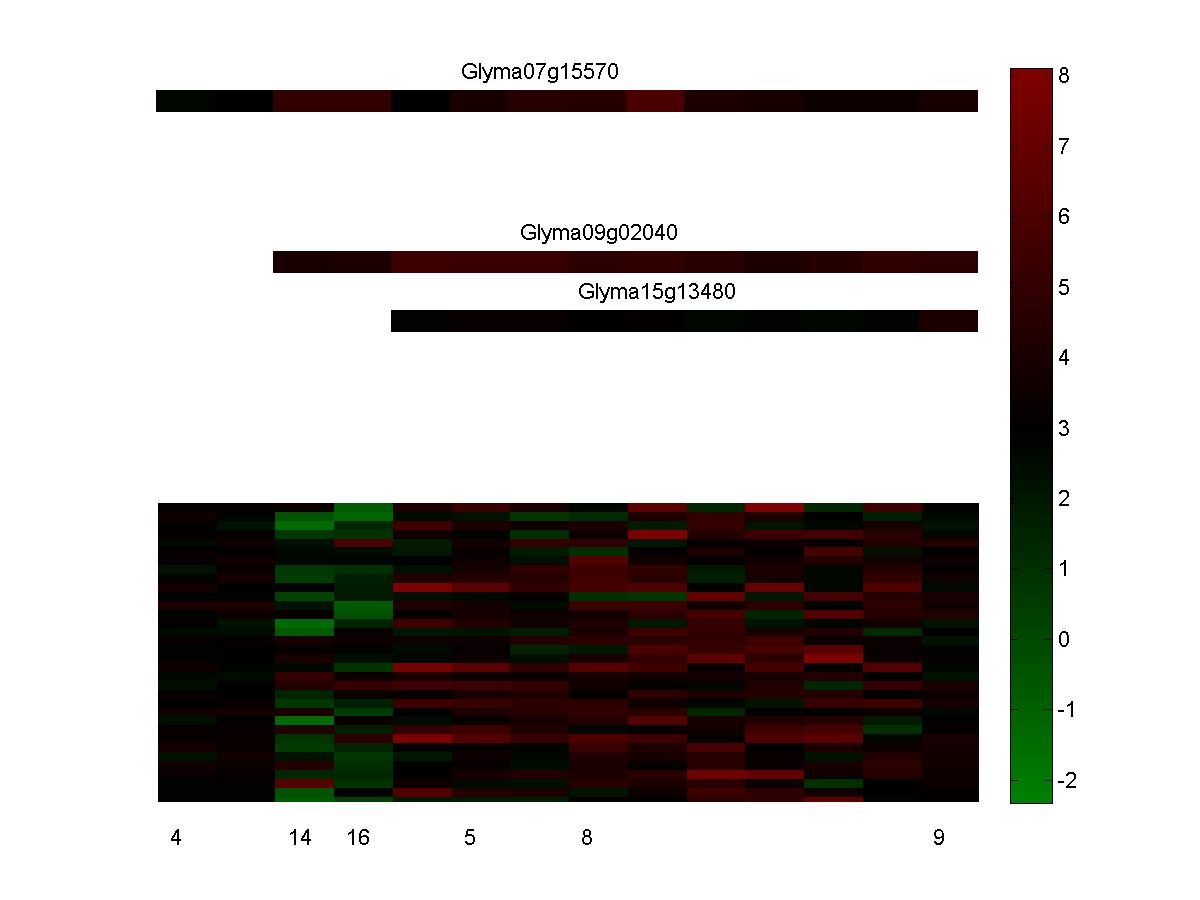


9 Glyma09g02040 MYB/HD-like

9 Glyma07g15570 SSB protein

9 Glyma15g13480 C3H-type1(Zn)

Glyma16g29790 Glyma04g42740 Glyma13g24380 Glyma14g06650 Glyma18g50180

Glyma11g03500 Glyma15g43200 Glyma01g45430 Glyma17g04940 Glyma07g05820

Glyma06g36590 Glyma12g15170 Glyma20g30140 Glyma14g06640 Glyma06g45320

Glyma12g33070 Glyma07g32150 Glyma08g22710 Glyma14g05850 Glyma12g04890

Glyma13g23790 Glyma16g31290 Glyma06g43060 Glyma01g31300 Glyma14g26660

Glyma06g14640 Glyma04g28560 Glyma03g28490 Glyma04g0569 Glyma14g39050

Glyma13g44870 Glyma07g37080 Glyma16g06410 Glyma09g17130

10


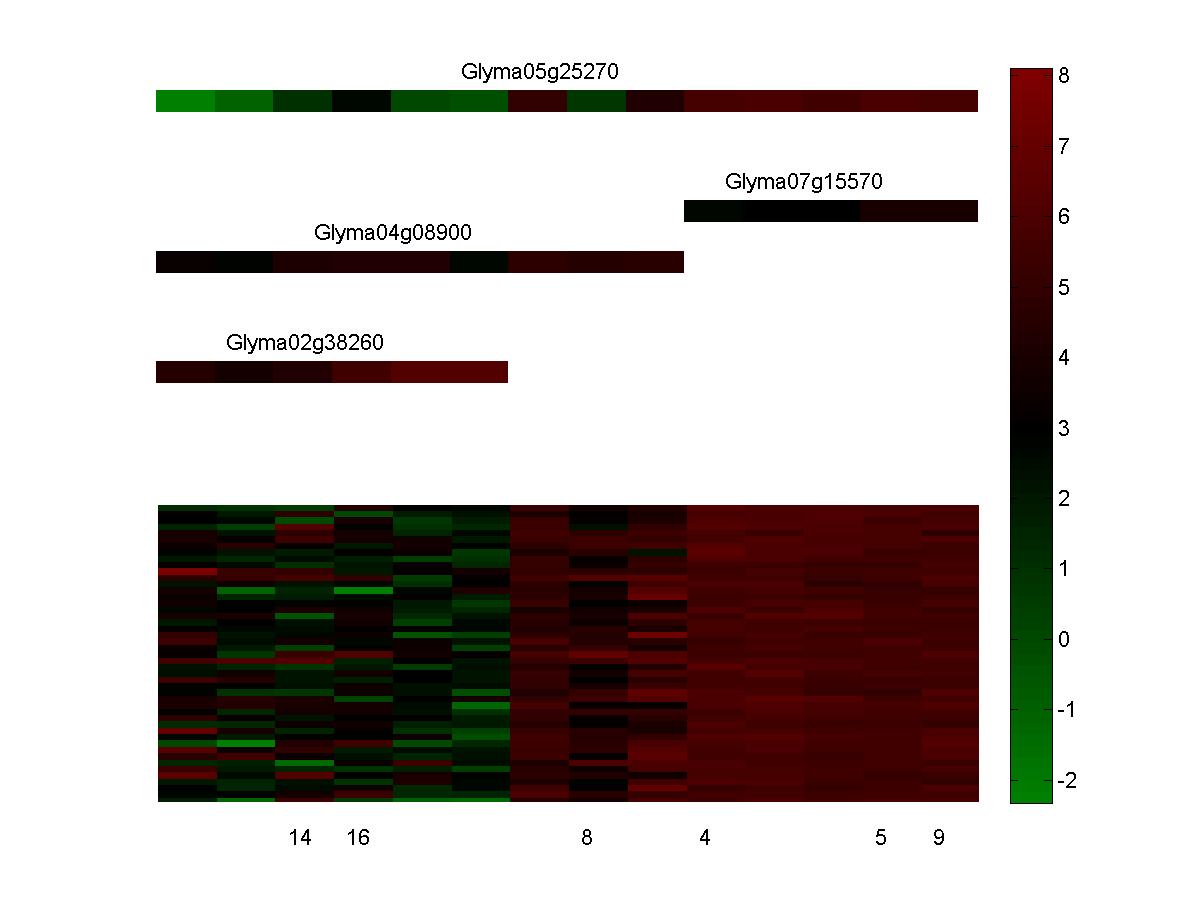


10 Glyma05g25270 WRKY

10 Glyma07g15570 SSB protein

10 Glyma02g38260 AUX-IAA-ARF

10 Glyma04g08900 AP2-EREBP

Glyma20g07060 Glyma04g04240 Glyma13g35550 Glyma07g25390 Glyma15g24130

Glyma05g37680 Glyma04g37220 Glyma07g37790 Glyma09g02590 Glyma04g34600

Glyma08g05200 Glyma18g18910 Glyma16g02490 Glyma01g42790 Glyma15g41700

Glyma14g35220 Glyma04g35710 Glyma03g24180 Glyma04g09040 Glyma06g21560

Glyma08g36660 Glyma01g02290 Glyma15g18070 Glyma05g25700 Glyma07g38530

Glyma18g40650 Glyma11g03430 Glyma07g30090 Glyma17g36950 Glyma01g03820

Glyma17g15860 Glyma08g41650 Glyma17g11330 Glyma15g14790 Glyma08g08550

Glyma18g44950 Glyma16g03960 Glyma14g39230 Glyma07g05390 Glyma07g16850

Glyma01g36170 Glyma13g40470 Glyma05g30690 Glyma08g18690 Glyma20g32140

Glyma02g15780 Glyma13g27060

11


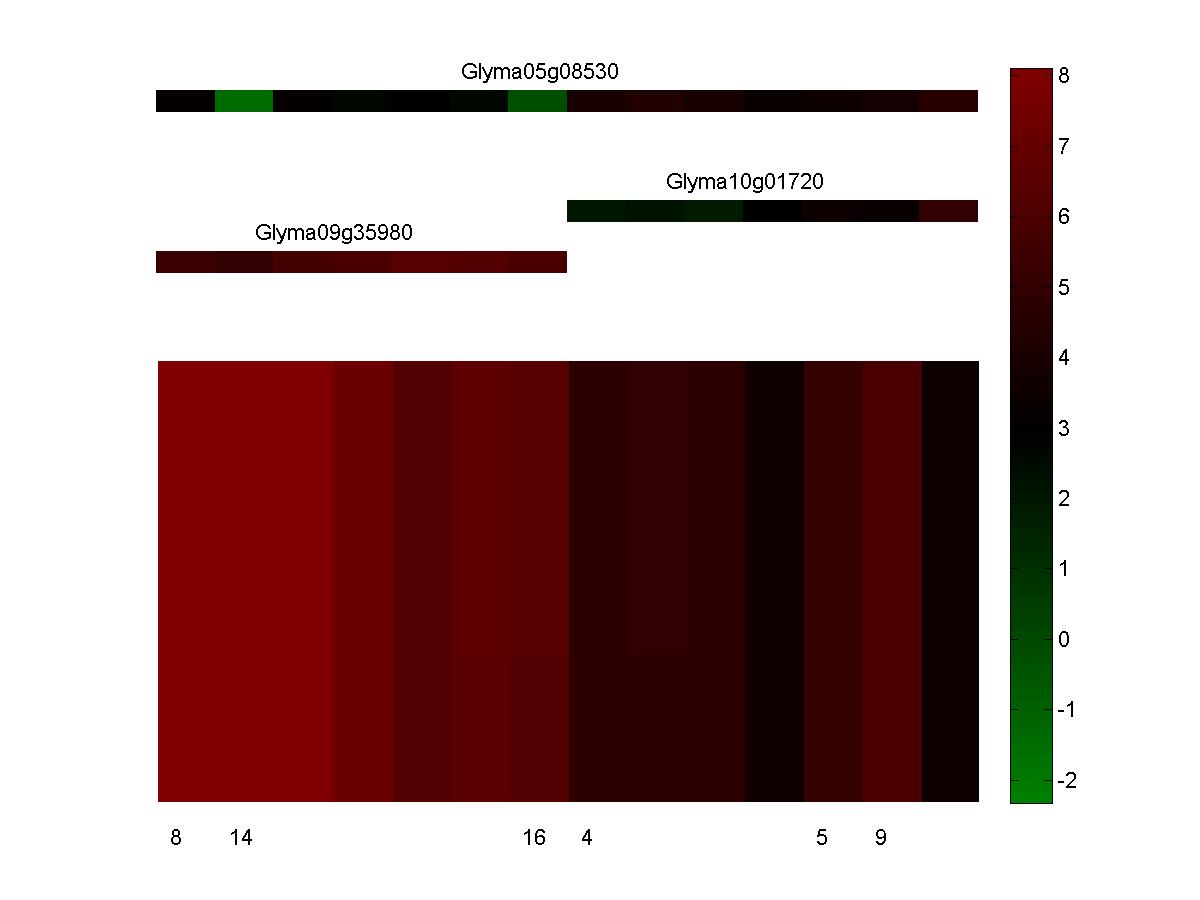


11 Glyma05g08530 TPR

11 Glyma10g01720 TPR

11 Glyma09g35980 C3H-type1(Zn)

Glyma08g36680 Glyma08g36690

12


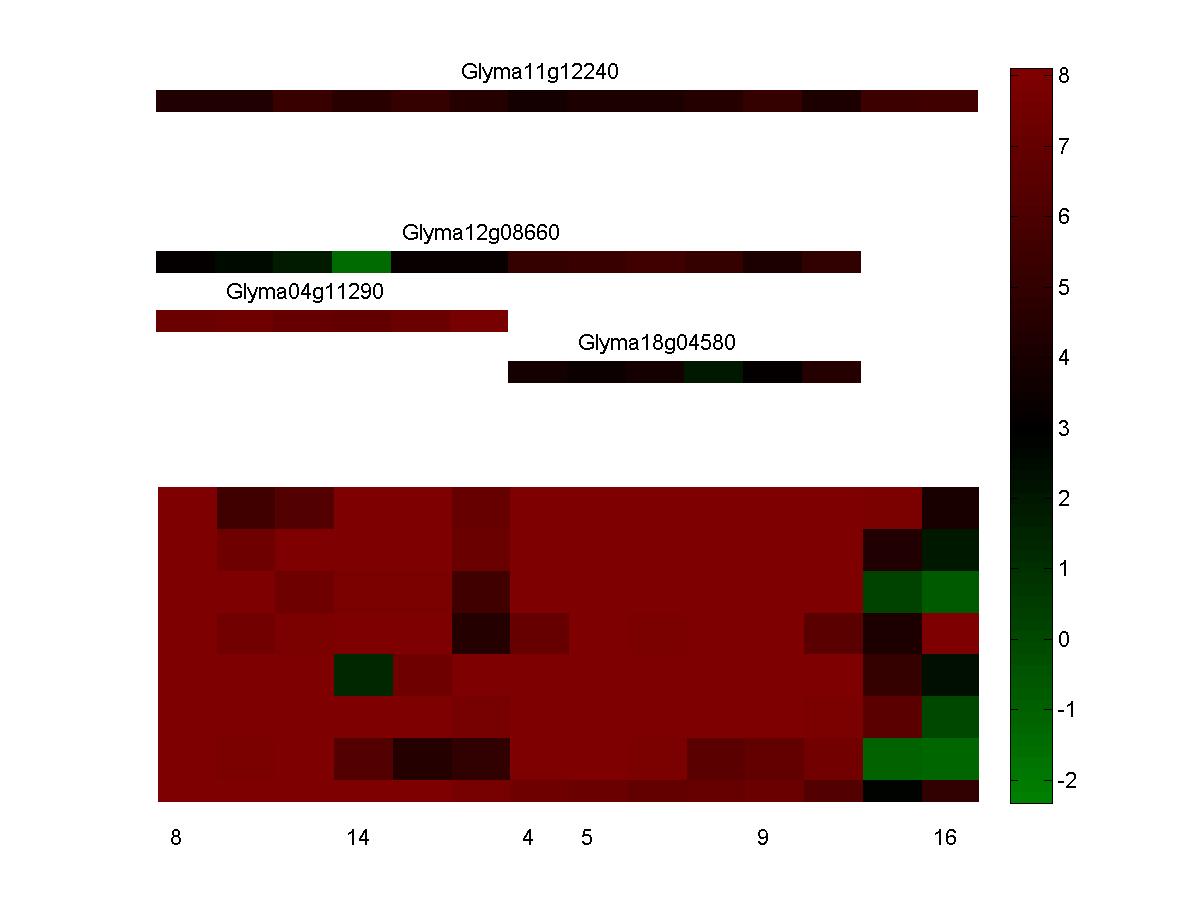


12 Glyma04g11290 AP2-EREBP

12 Glyma18g04580 MYB/HD-like

12 Glyma12g08660 C2H2 (Zn)

12 Glyma11g12240 BZIP

Glyma08g21410 Glyma12g34570 Glyma12g34550 Glyma17g34870 Glyma10g35870

Glyma07g01730 Glyma15g12170 Glyma02g18090

13


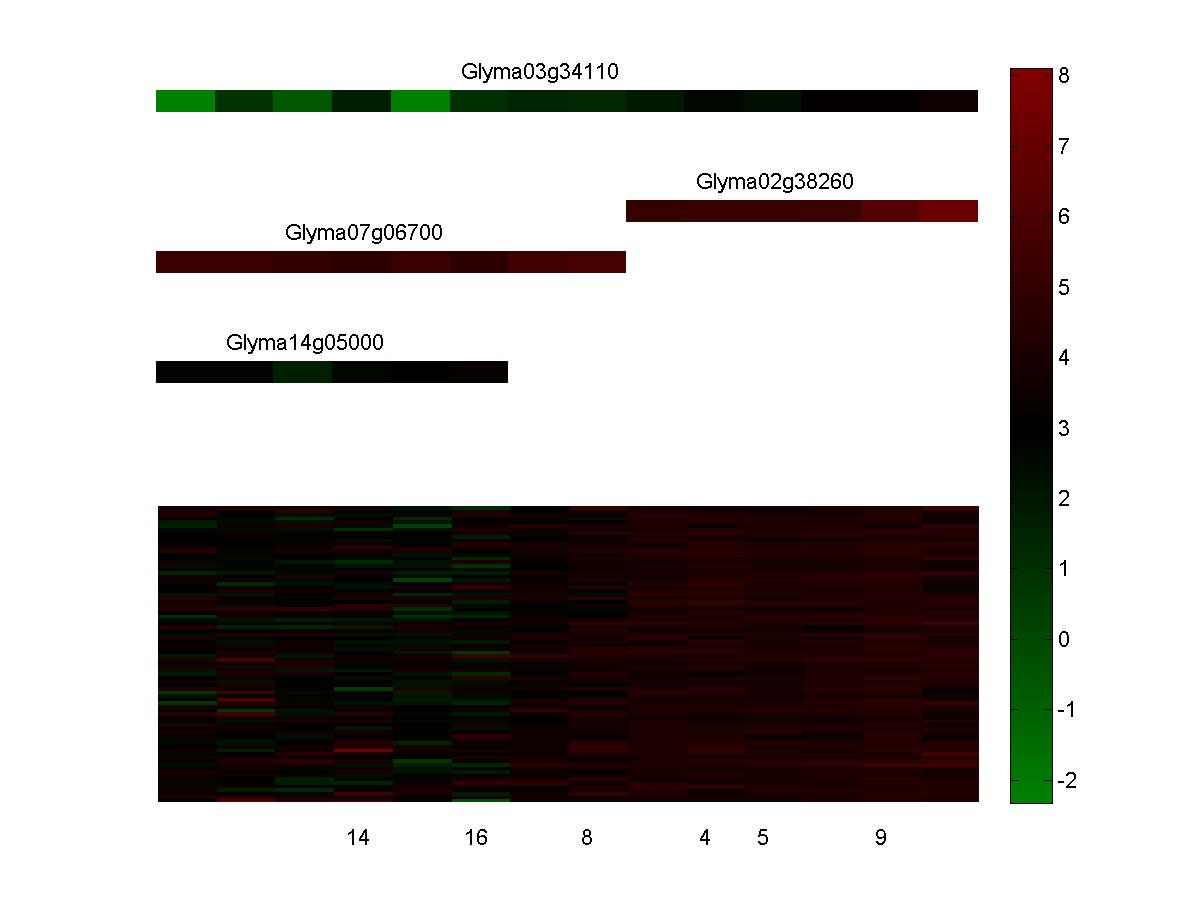


13 Glyma03g34110 MYB/HD-like

13 Glyma02g38260 AUX-IAA-ARF

13 Glyma14g05000 BTB/POZ

13 Glyma07g06700 BTB/POZ

Glyma11g26970 Glyma15g06520 Glyma03g01850 Glyma19g34120 Glyma02g01180

Glyma02g36980 Glyma17g13890 Glyma20g01640 Glyma02g14860 Glyma08g19090

Glyma20g27160 Glyma07g39590 Glyma20g30100 Glyma04g02820 Glyma17g07780

Glyma11g12090 Glyma18g01140 Glyma17g34140 Glyma15g05060 Glyma01g43680

Glyma03g37510 Glyma05g35490 Glyma12g04790 Glyma13g44390 Glyma11g04420

Glyma17g21590 Glyma04g02900 Glyma18g10400 Glyma02g48100 Glyma07g02430

Glyma10g04770 Glyma20g38430 Glyma02g45980 Glyma13g34340 Glyma11g37720

Glyma17g04440 Glyma15g02860 Glyma02g37510 Glyma10g05100 Glyma13g28250

Glyma02g13520 Glyma04g27580 Glyma18g49060 Glyma05g29400 Glyma02g01210

Glyma20g24750 Glyma06g12260 Glyma04g07980 Glyma07g02420 Glyma09g29480

Glyma01g43480 Glyma03g40710 Glyma08g44330 Glyma11g30020 Glyma08g10890

Glyma13g10800 Glyma19g38130 Glyma04g26440 Glyma18g08230 Glyma20g18290

Glyma09g30230 Glyma11g11510 Glyma06g11380 Glyma13g04940 Glyma10g01220

Glyma17g36400 Glyma13g26470 Glyma04g40030 Glyma13g23920 Glyma02g08600

Glyma11g15700 Glyma15g05730 Glyma10g08540 Glyma01g35110 Glyma04g41770

Glyma14g35160 Glyma05g36350 Glyma08g27730 Glyma01g04730 Glyma20g15460

Glyma20g38270 Glyma03g29080

14


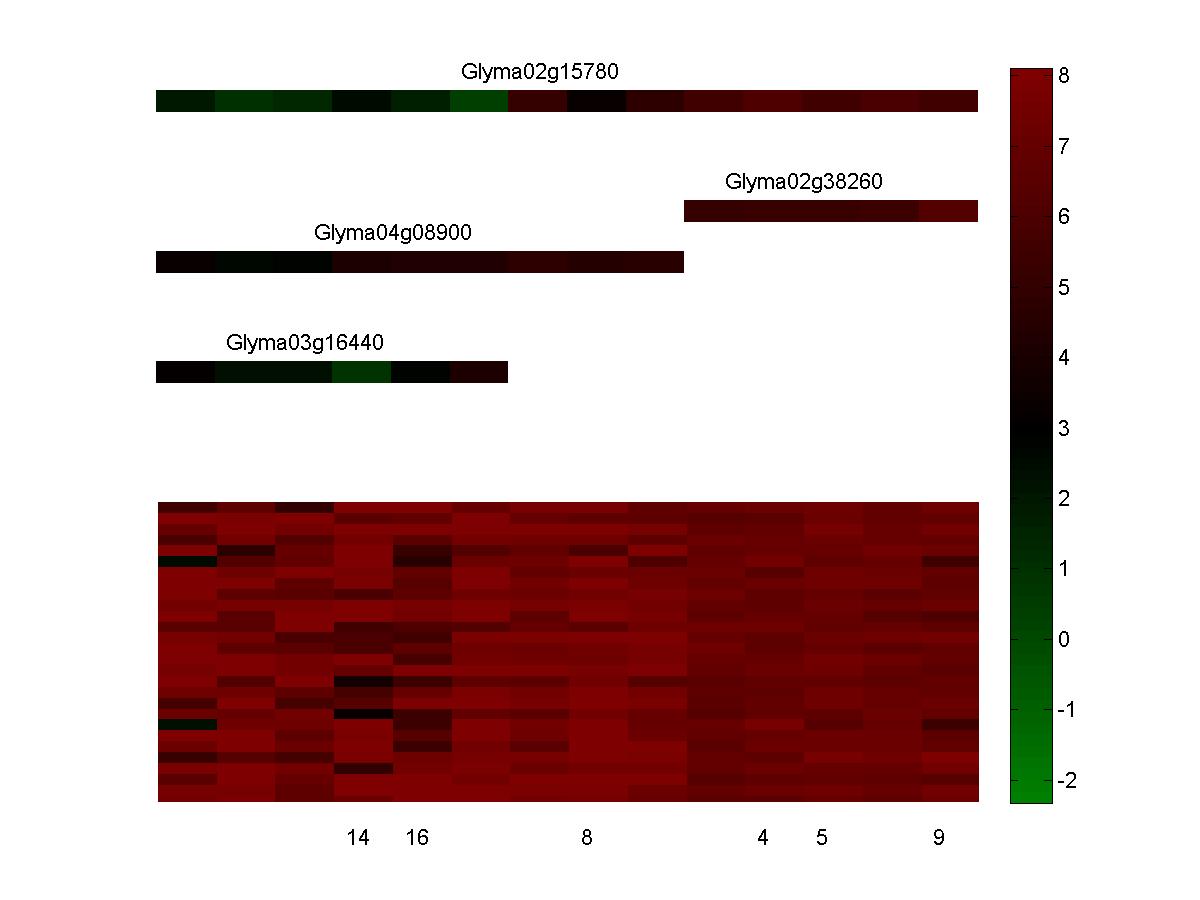


14 Glyma04g08900 AP2-EREBP

14 Glyma02g38260 AUX-IAA-ARF

14 Glyma03g16440 TPR

14 Glyma02g15780 bHLH

Glyma12g19520 Glyma04g39380 Glyma06g00990 Glyma17g24160 Glyma05g13900

Glyma01g45300 Glyma12g08040 Glyma06g01970 Glyma04g00960 Glyma17g14940

Glyma06g09420 Glyma01g02400 Glyma05g27180 Glyma07g39020 Glyma07g38940

Glyma11g12500 Glyma06g44380 Glyma03g40760 Glyma05g27190 Glyma04g03020

Glyma14g05510 Glyma14g10590 Glyma17g34920 Glyma02g11580 Glyma10g40460

Glyma02g43470 Glyma01g45290 Glyma13g23170

15


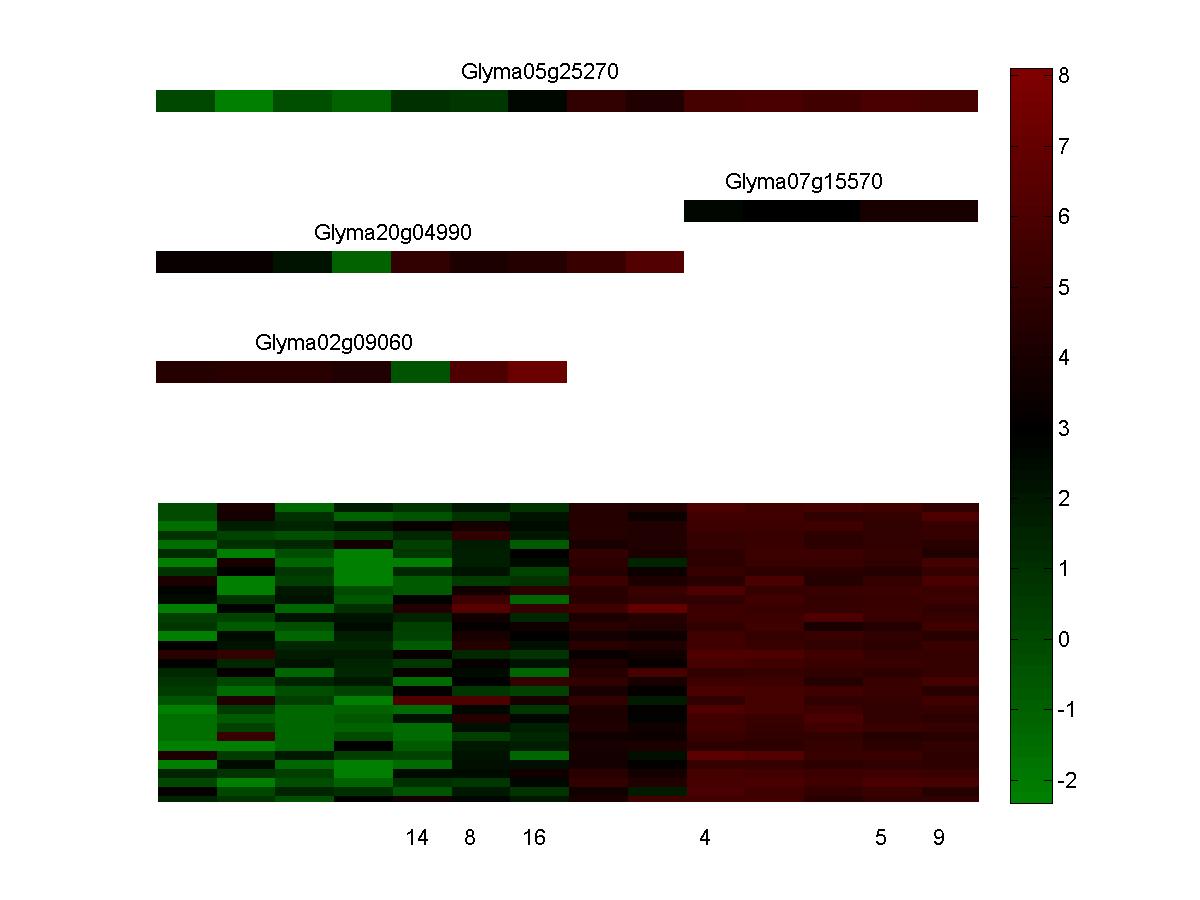


15 Glyma02g09060 MYB/HD-like

15 Glyma07g15570 SSB protein

15 Glyma20g04990 SRS

15 Glyma05g25270 WRKY

Glyma18g50750 Glyma17g34590 Glyma12g03450 Glyma13g41540 Glyma02g46600

Glyma04g38710 Glyma13g36110 Glyma01g04380 Glyma18g44250 Glyma14g26700

Glyma05g00930 Glyma02g34670 Glyma04g33010 Glyma09g12860 Glyma03g29770

Glyma17g13530 Glyma14g35660 Glyma08g14600 Glyma13g01690 Glyma07g38580

Glyma14g17060 Glyma19g32650 Glyma10g31020 Glyma12g11210 Glyma19g42200

Glyma07g05620 Glyma20g31040 Glyma08g46610 Glyma08g47620 Glyma20g28490

Glyma10g08300 Glyma02g07700 Glyma05g25270

16


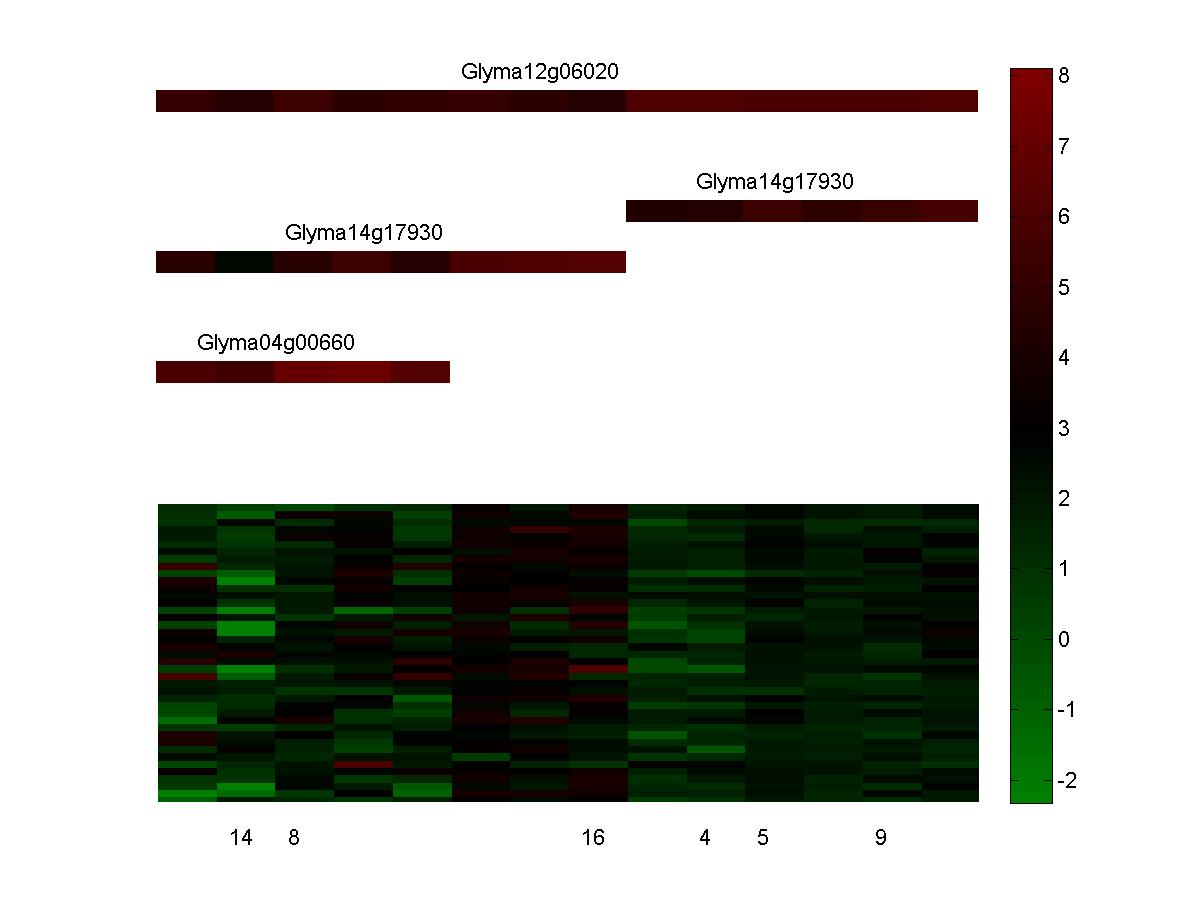


16 Glyma14g17930 CCHC (Zn)

16 Glyma12g06020 zf-A20

16 Glyma04g00660 CSD

Glyma20g35750 Glyma15g11440 Glyma05g25600 Glyma06g18770 Glyma17g04640

Glyma11g36790 Glyma09g04560 Glyma09g38070 Glyma02g04590 Glyma03g37710

Glyma09g34830 Glyma20g01730 Glyma20g01830 Glyma06g00400 Glyma20g01690

Glyma04g42790 Glyma03g28180 Glyma08g46690 Glyma11g04240 Glyma07g00730

Glyma02g29420 Glyma01g26650 Glyma15g07410 Glyma12g35940 Glyma13g12000

Glyma16g05950 Glyma17g15090 Glyma15g18060 Glyma11g07140 Glyma14g39590

Glyma19g44410 Glyma19g40570 Glyma11g35910 Glyma11g27190 Glyma13g23220

Glyma04g37340 Glyma13g44440 Glyma09g21300 Glyma01g09530 Glyma15g12310

Glyma10g07240

17


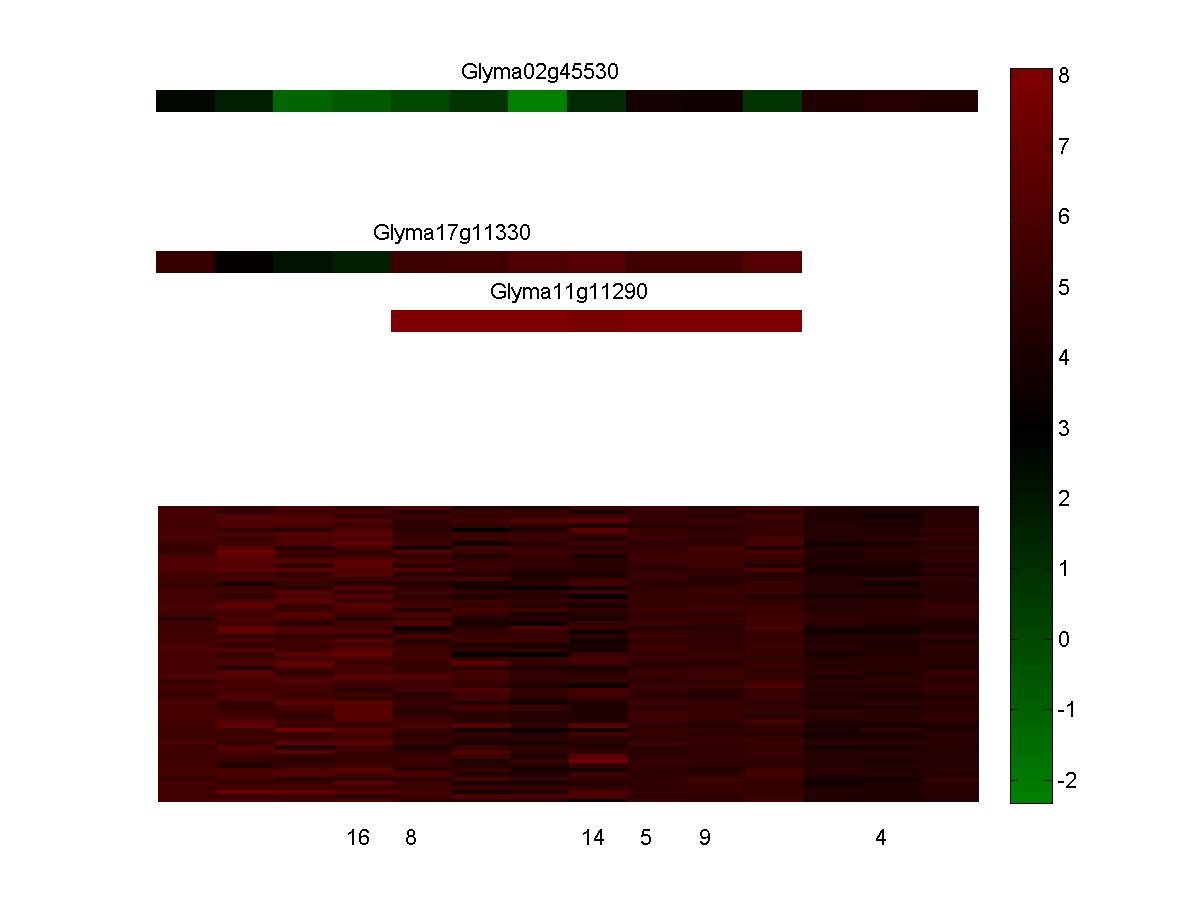


17 Glyma17g11330 Homeodomain/HOMEOBOX

17 Glyma11g11290 CSD

17 Glyma02g45530 WRKY

Glyma05g37790 Glyma16g03020 Glyma20g10260 Glyma11g07980 Glyma02g42890

Glyma02g43640 Glyma20g33270 Glyma15g34720 Glyma13g37600 Glyma16g22530

Glyma05g29580 Glyma07g14460 Glyma09g03560 Glyma02g43500 Glyma12g01340

Glyma17g23520 Glyma06g11680 Glyma03g40250 Glyma20g22850 Glyma07g05550

Glyma09g06930 Glyma04g11160 Glyma03g31460 Glyma09g06940 Glyma15g05410

Glyma15g06890 Glyma01g10070 Glyma02g10470 Glyma08g05930 Glyma14g02940

Glyma07g16970 Glyma18g45420 Glyma04g12680 Glyma06g00430 Glyma04g03390

Glyma08g13320 Glyma14g17930 Glyma16g07660 Glyma05g24550 Glyma06g10930

Glyma04g08570 Glyma03g00870 Glyma07g03180 Glyma15g00350 Glyma01g06170

Glyma20g02270 Glyma11g36580 Glyma16g02090 Glyma19g39250 Glyma01g02660

Glyma20g24850 Glyma07g06700 Glyma17g07130 Glyma15g15900 Glyma04g42830

Glyma13g19870 Glyma20g35120 Glyma08g09090 Glyma03g33080 Glyma15g10020

Glyma12g35730 Glyma06g33950 Glyma09g40420 Glyma07g37430 Glyma04g04570

Glyma13g16570 Glyma13g22800

18


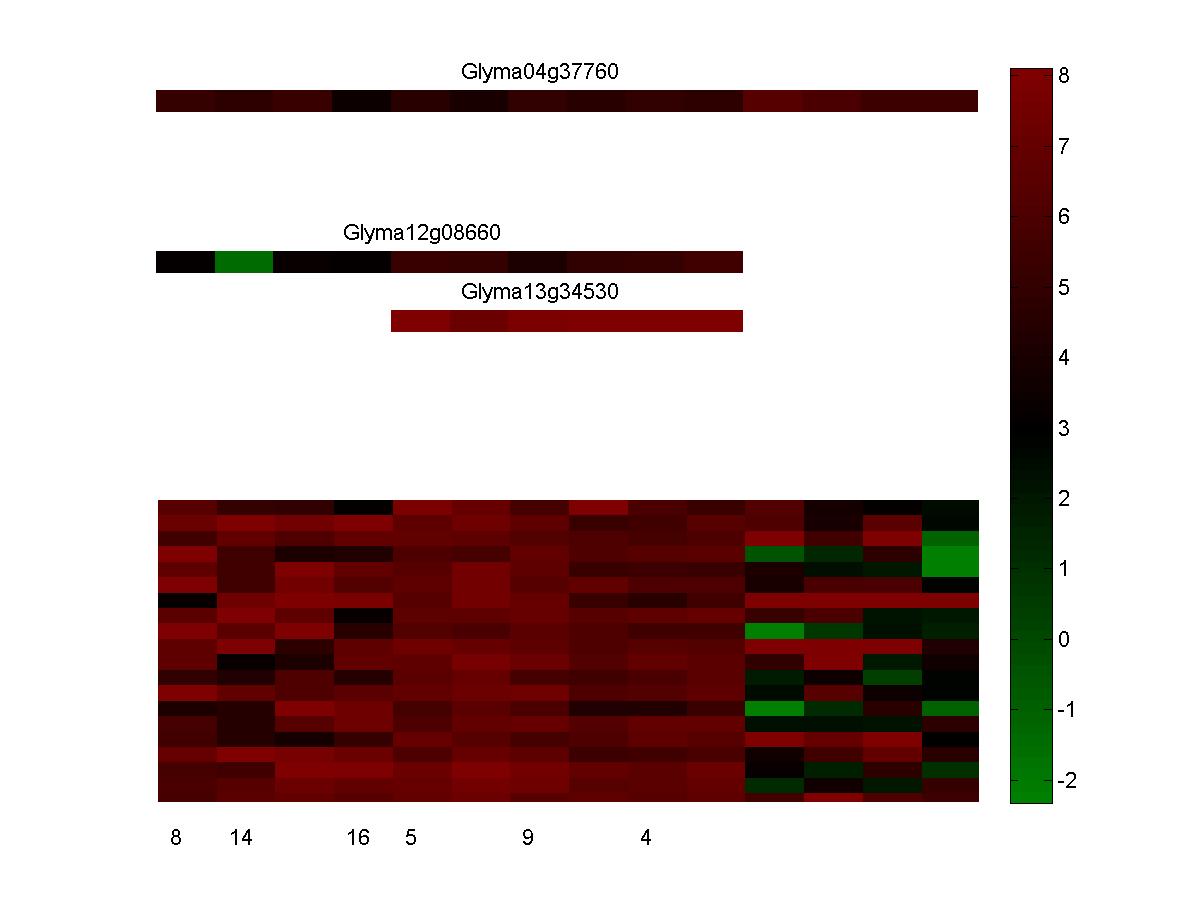


18 Glyma13g34530 C2H2 (Zn)

18 Glyma04g37760 AUX-IAA-ARF

18 Glyma12g08660 C2H2 (Zn)

Glyma09g04630 Glyma16g27880 Glyma11g37360 Glyma09g04340 Glyma04g08070

Glyma10g39450 Glyma17g03040 Glyma20g30910 Glyma07g03910 Glyma18g01330

Glyma17g14850 Glyma13g42340 Glyma09g04530 Glyma03g29440 Glyma13g06230

Glyma12g06950 Glyma16g27440 Glyma02g12980 Glyma08g06420 Glyma14g09510

19


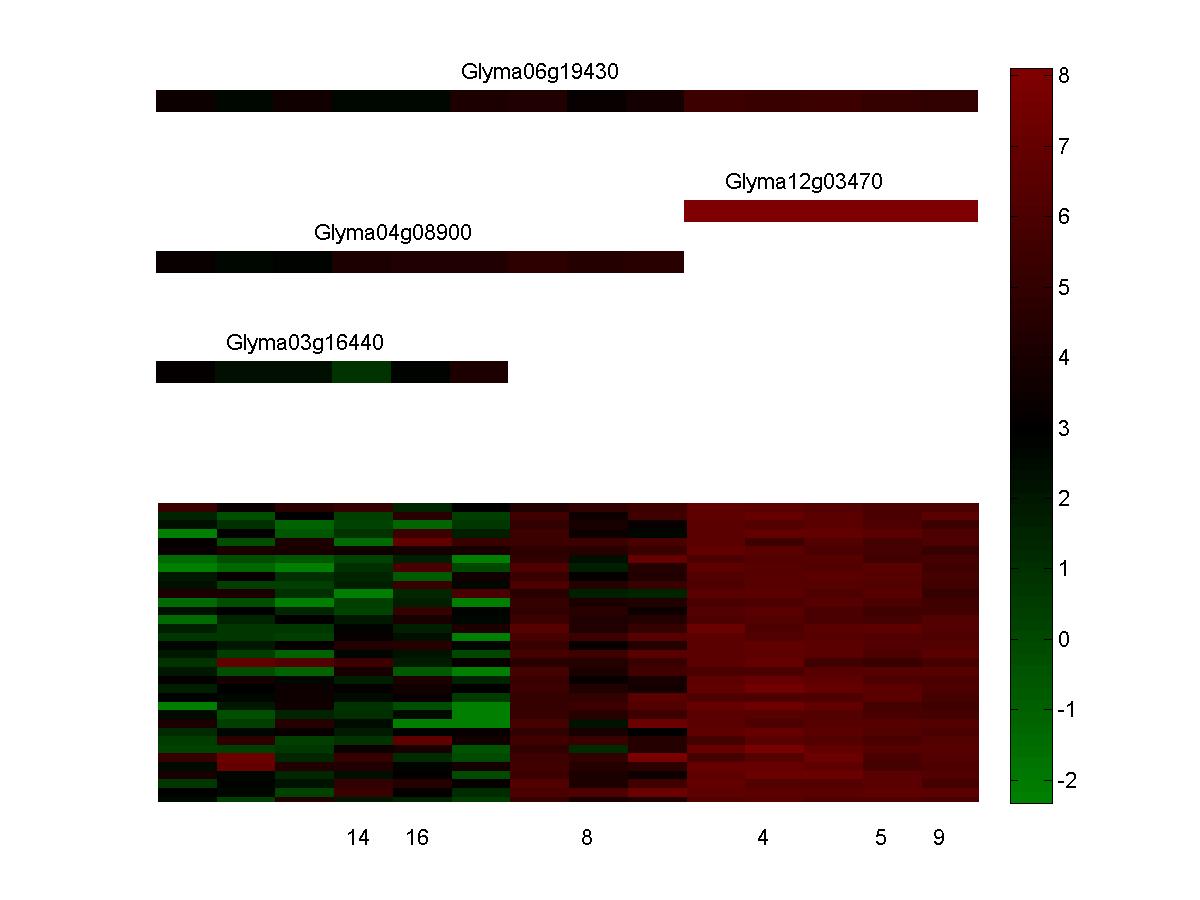


19 Glyma12g03470 CSD

19 Glyma04g08900 AP2-EREBP

19 Glyma06g19430 TPR

19 Glyma03g16440 TPR

Glyma01g37810 Glyma08g20230 Glyma01g05580 Glyma01g00770 Glyma18g45260

Glyma04g03980 Glyma08g17230 Glyma04g42460 Glyma14g35940 Glyma12g08520

Glyma19g25980 Glyma07g16420 Glyma09g40970 Glyma15g27660 Glyma15g00600

Glyma08g45920 Glyma04g40170 Glyma16g01960 Glyma01g36590 Glyma05g09990

Glyma20g39190 Glyma19g33730 Glyma12g04940 Glyma18g20510 Glyma08g00320

Glyma15g30110 Glyma02g15150 Glyma08g42440 Glyma10g28610 Glyma15g41970

Glyma16g29220 Glyma06g41220 Glyma15g13500 Glyma02g12520 Glyma18g05700

20


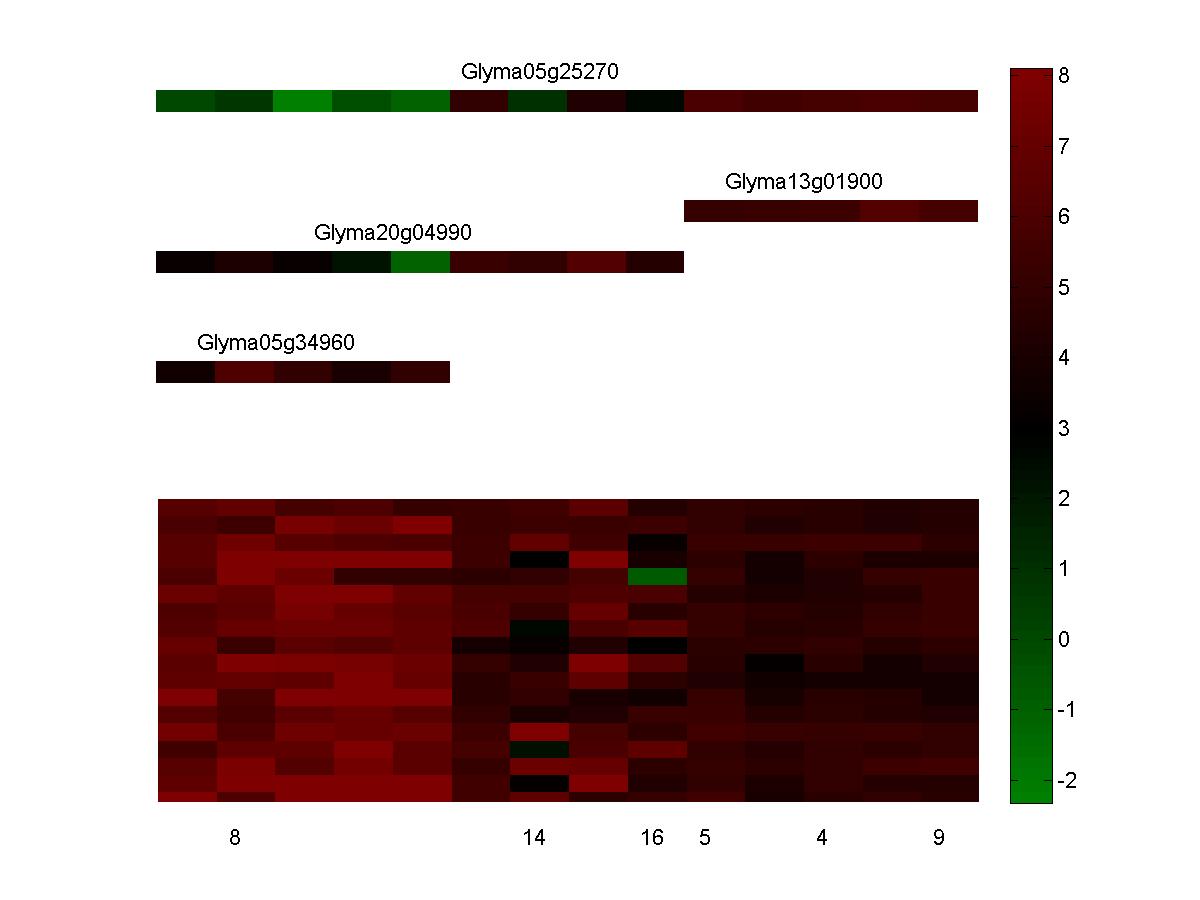


20 Glyma13g01900 TPR

20 Glyma05g25270 WRKY

20 Glyma20g04990 SRS

20 Glyma05g34960 ZIM

Glyma15g41540 Glyma10g32250 Glyma01g39270 Glyma11g13070 Glyma08g03290

Glyma08g17610 Glyma17g37020 Glyma08g02610 Glyma20g08800 Glyma01g02230

Glyma09g33160 Glyma01g02220 Glyma04g04540 Glyma09g33750 Glyma15g01950

Glyma03g40640 Glyma14g00620 Glyma07g15960

21


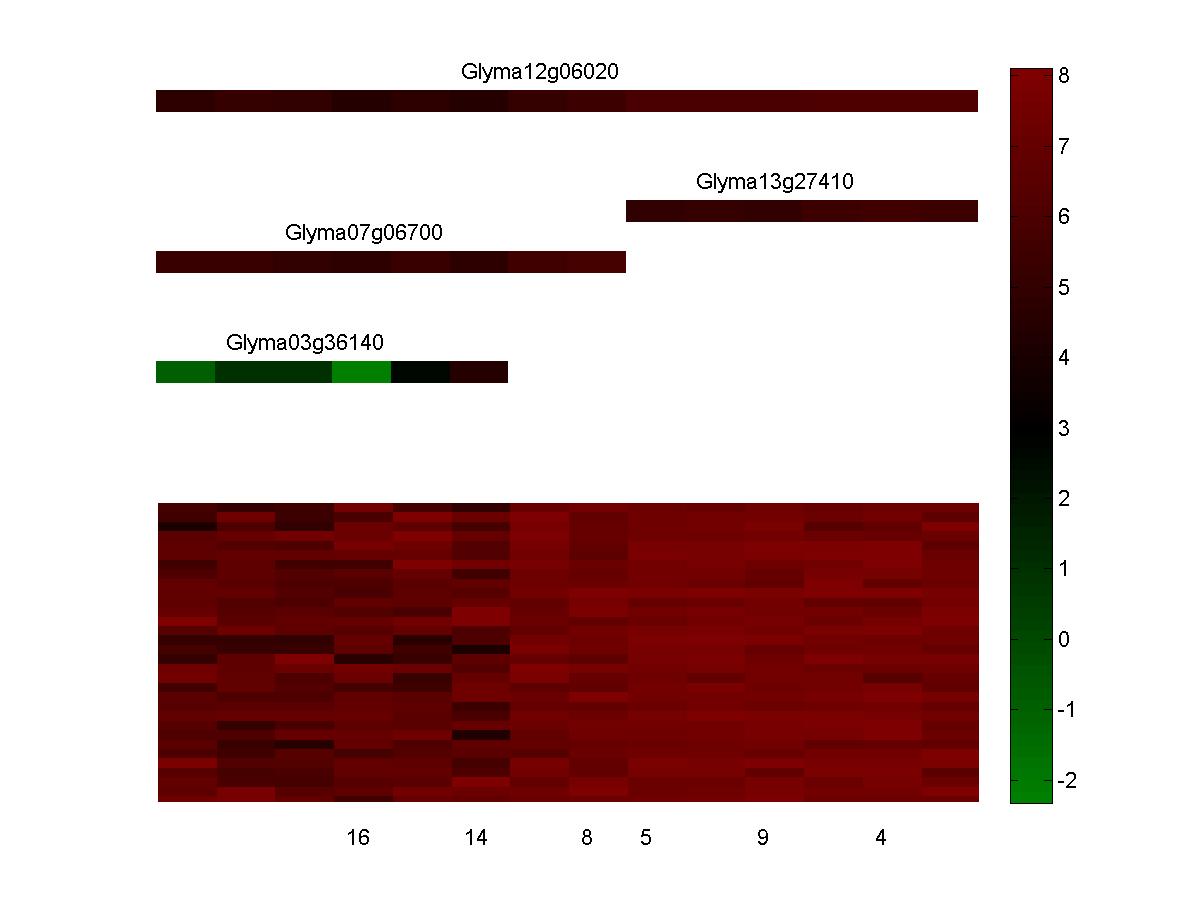


21 Glyma13g27410 zf-A20

21 Glyma12g06020 zf-A20

21 Glyma03g36140 CCAAT

21 Glyma07g06700 BTB/POZ

Glyma11g36950 Glyma06g13020 Glyma20g29660 Glyma16g01500 Glyma19g44160

Glyma03g32880 Glyma08g04740 Glyma05g30140 Glyma06g13760 Glyma15g13970

Glyma09g01450 Glyma07g32020 Glyma12g10160 Glyma08g14130 Glyma13g28240

Glyma20g05560 Glyma13g44170 Glyma10g44370 Glyma08g11000 Glyma16g20780

Glyma17g23860 Glyma02g44350 Glyma20g38020 Glyma04g07220 Glyma18g04500

Glyma15g10710 Glyma07g00760 Glyma0169s00210 Glyma09g24890 Glyma04g11290

Glyma19g01300 Glyma08g11040

22


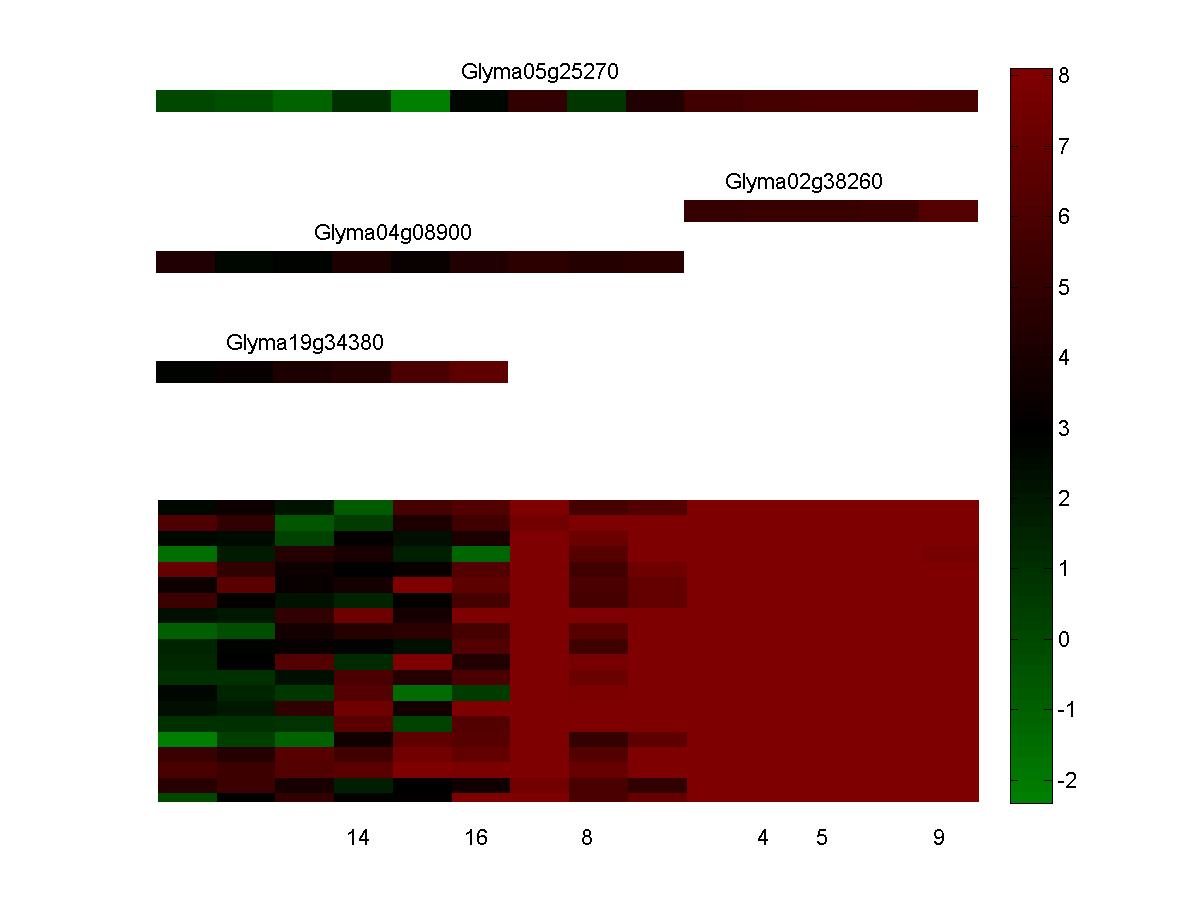


22 Glyma02g38260 AUX-IAA-ARF

22 Glyma19g34380 AUX-IAA-ARF

22 Glyma05g25270 WRKY

22 Glyma04g08900 AP2-EREBP

Glyma08g26140 Glyma11g18320 Glyma07g32340 Glyma17g14620 Glyma04g08830

Glyma07g32330 Glyma12g00400 Glyma17g03350 Glyma02g09220 Glyma07g38110

Glyma08g38740 Glyma20g35630 Glyma01g26840 Glyma12g06100 Glyma11g14140

Glyma09g05440 Glyma13g32300 Glyma17g02600 Glyma09g02600 Glyma01g31750

23


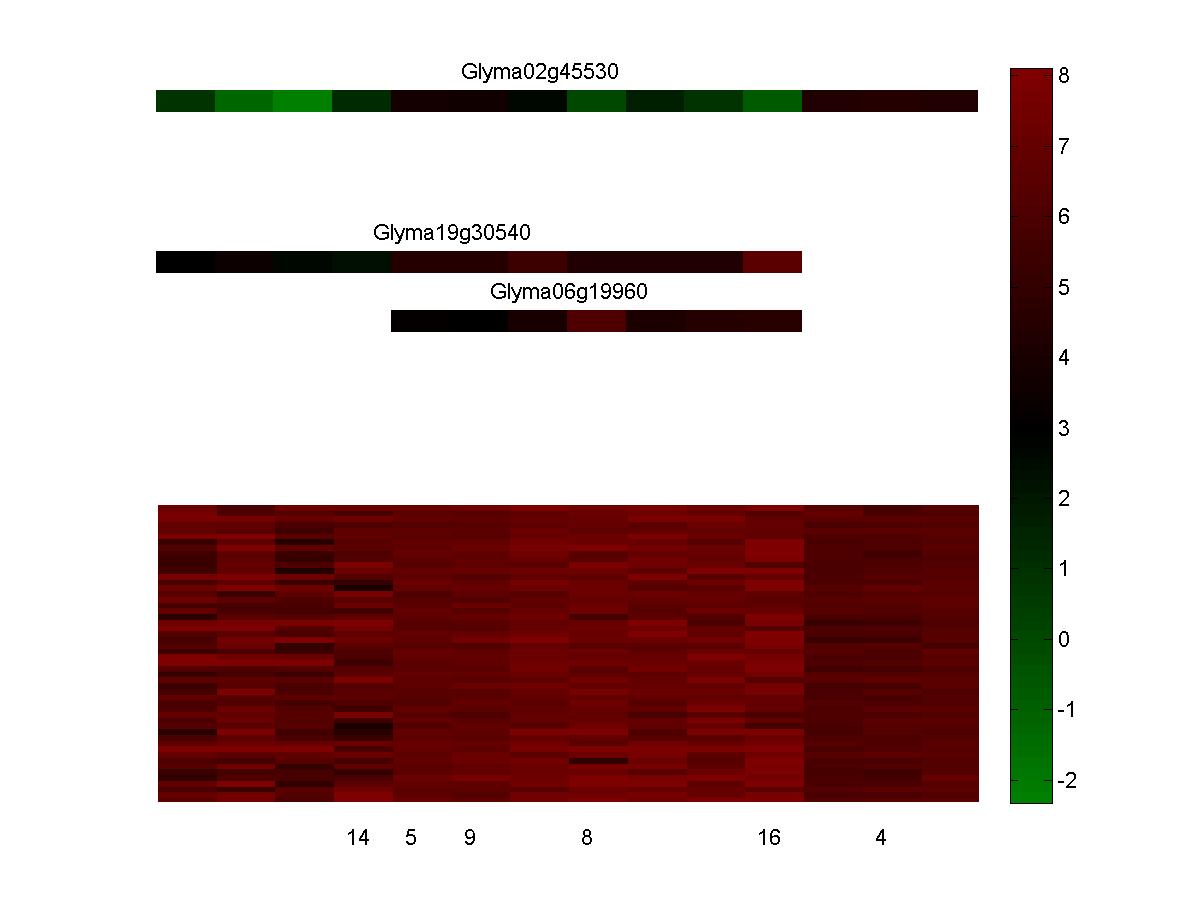


23 Glyma19g30540 NAC

23 Glyma02g45530 WRKY

23 Glyma06g19960 MYB/HD-like

Glyma05g21820 Glyma11g03570 Glyma08g18760 Glyma11g33720 Glyma14g05470

Glyma08g10140 Glyma12g35990 Glyma05g37170 Glyma05g00400 Glyma12g08930

Glyma02g15190 Glyma18g48620 Glyma05g30940 Glyma18g00500 Glyma04g00500

Glyma05g30950 Glyma04g00650 Glyma02g42080 Glyma11g08440 Glyma13g16500

Glyma18g02210 Glyma18g52860 Glyma08g45710 Glyma07g15320 Glyma14g36620

Glyma12g31040 Glyma14g07150 Glyma18g01870 Glyma12g09250 Glyma08g13840

Glyma07g33300 Glyma18g47780 Glyma17g17850 Glyma15g06570 Glyma01g36850

Glyma04g00660 Glyma05g30020 Glyma13g17660 Glyma13g41960 Glyma14g40320

Glyma11g10790 Glyma09g32540 Glyma20g28440 Glyma10g32580 Glyma15g15910

Glyma08g13300 Glyma15g09800 Glyma08g24000 Glyma17g38140 Glyma17g12840

Glyma09g38590 Glyma03g25820

24


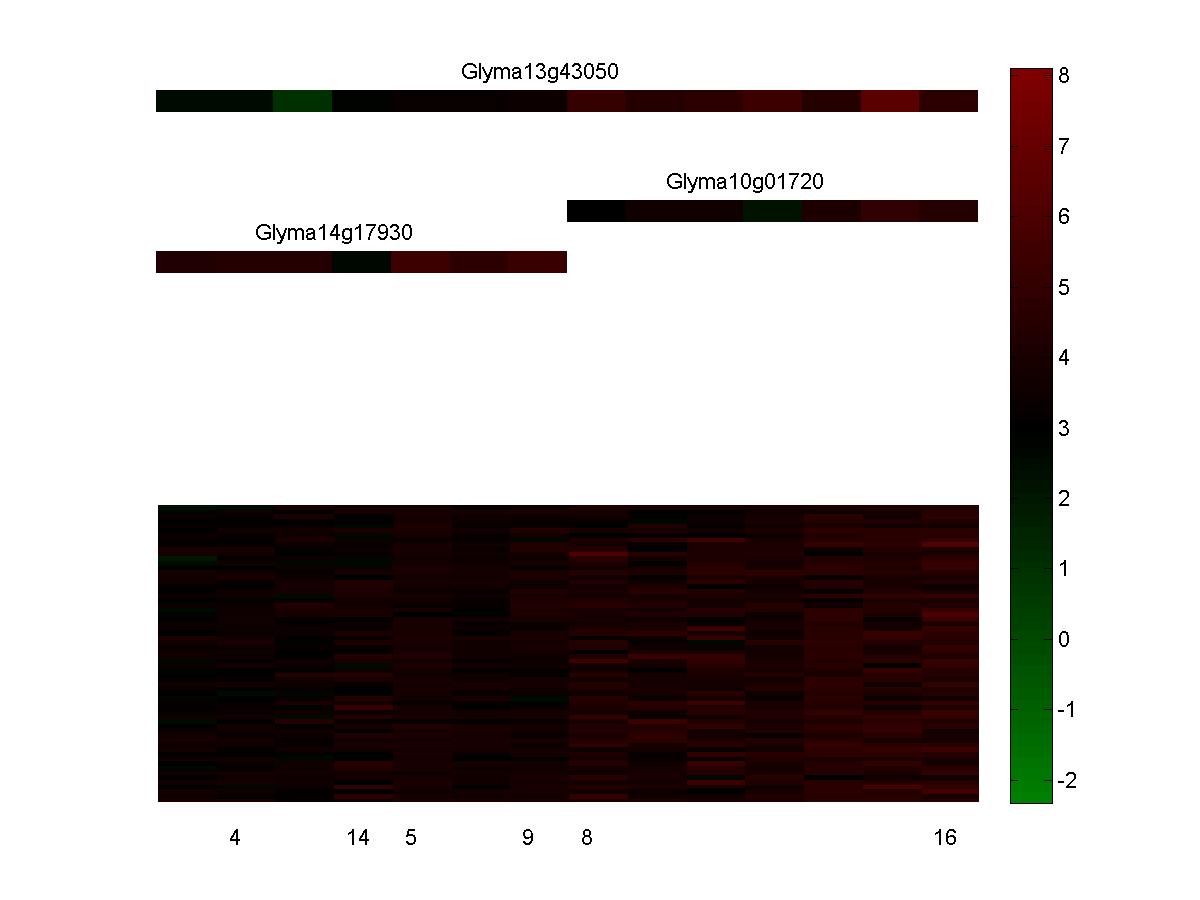


24 Glyma13g43050 AUX-IAA-ARF

24 Glyma14g17930 CCHC (Zn)

24 Glyma10g01720 TPR

Glyma19g28160 Glyma20g25630 Glyma09g15300 Glyma07g37190 Glyma08g17650

Glyma08g00510 Glyma18g48290 Glyma03g34240 Glyma07g12190 Glyma20g24340

Glyma20g24480 Glyma20g24050 Glyma09g18050 Glyma08g47690 Glyma03g36890

Glyma20g33080 Glyma07g37820 Glyma12g10750 Glyma15g12800 Glyma04g34720

Glyma11g14150 Glyma13g02870 Glyma10g40560 Glyma07g08650 Glyma17g33020

Glyma08g19520 Glyma17g35590 Glyma15g14390 Glyma07g15570 Glyma02g43410

Glyma17g35770 Glyma02g10570 Glyma16g01760 Glyma17g35580 Glyma11g18710

Glyma07g30030 Glyma06g12440 Glyma12g02440 Glyma06g13630 Glyma17g05930

Glyma14g06010 Glyma10g34750 Glyma10g41070 Glyma16g00530 Glyma13g42690

Glyma13g08480 Glyma04g08900 Glyma16g27430 Glyma20g38710 Glyma14g04020

Glyma15g40170 Glyma09g08190 Glyma02g05550 Glyma03g02060 Glyma12g00350

Glyma13g29110 Glyma12g01430 Glyma17g12270 Glyma06g48330 Glyma15g11230

Glyma06g45990 Glyma12g23170 Glyma04g02030 Glyma05g03260

25


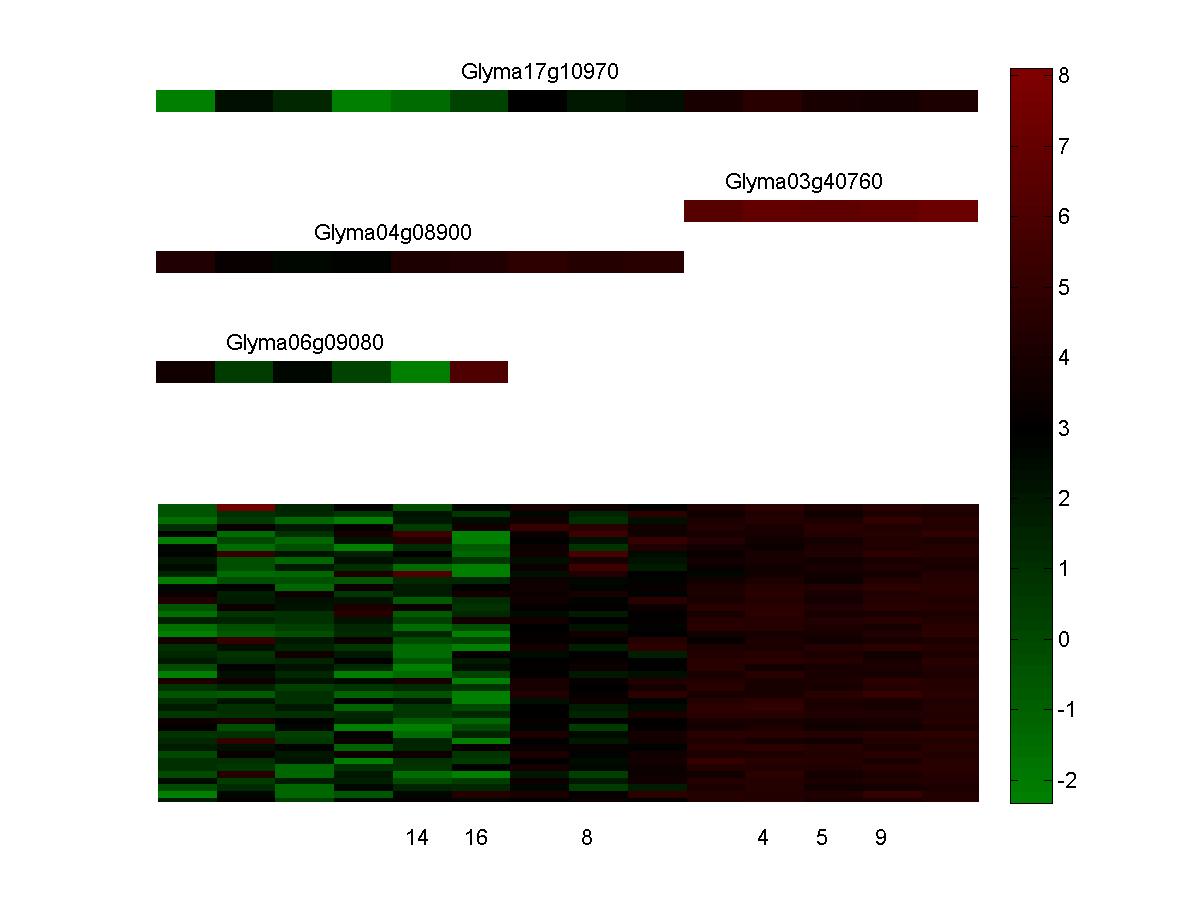


25 Glyma03g40760 AUX-IAA-ARF

25 Glyma06g09080 C2C2 (Zn) GATA

25 Glyma04g08900 AP2-EREBP

25 Glyma17g10970 NAC

Glyma03g03670 Glyma06g13910 Glyma08g19430 Glyma09g41120 Glyma13g10460

Glyma09g27320 Glyma14g38650 Glyma16g08020 Glyma06g10970 Glyma13g37830

Glyma10g38640 Glyma15g07260 Glyma14g09070 Glyma18g53860 Glyma13g00590

Glyma02g26890 Glyma03g05600 Glyma12g35000 Glyma12g34410 Glyma03g04450

Glyma09g34380 Glyma10g43340 Glyma08g25390 Glyma16g26950 Glyma14g01440

Glyma03g39580 Glyma02g15620 Glyma17g10970 Glyma02g15370 Glyma08g20330

Glyma04g05550 Glyma18g05710 Glyma20g25660 Glyma13g27530 Glyma13g01960

Glyma09g39690 Glyma20g34980 Glyma15g42800 Glyma03g40480 Glyma09g41560

Glyma17g36150 Glyma13g42420 Glyma20g22290 Glyma05g28810 Glyma14g09080

26


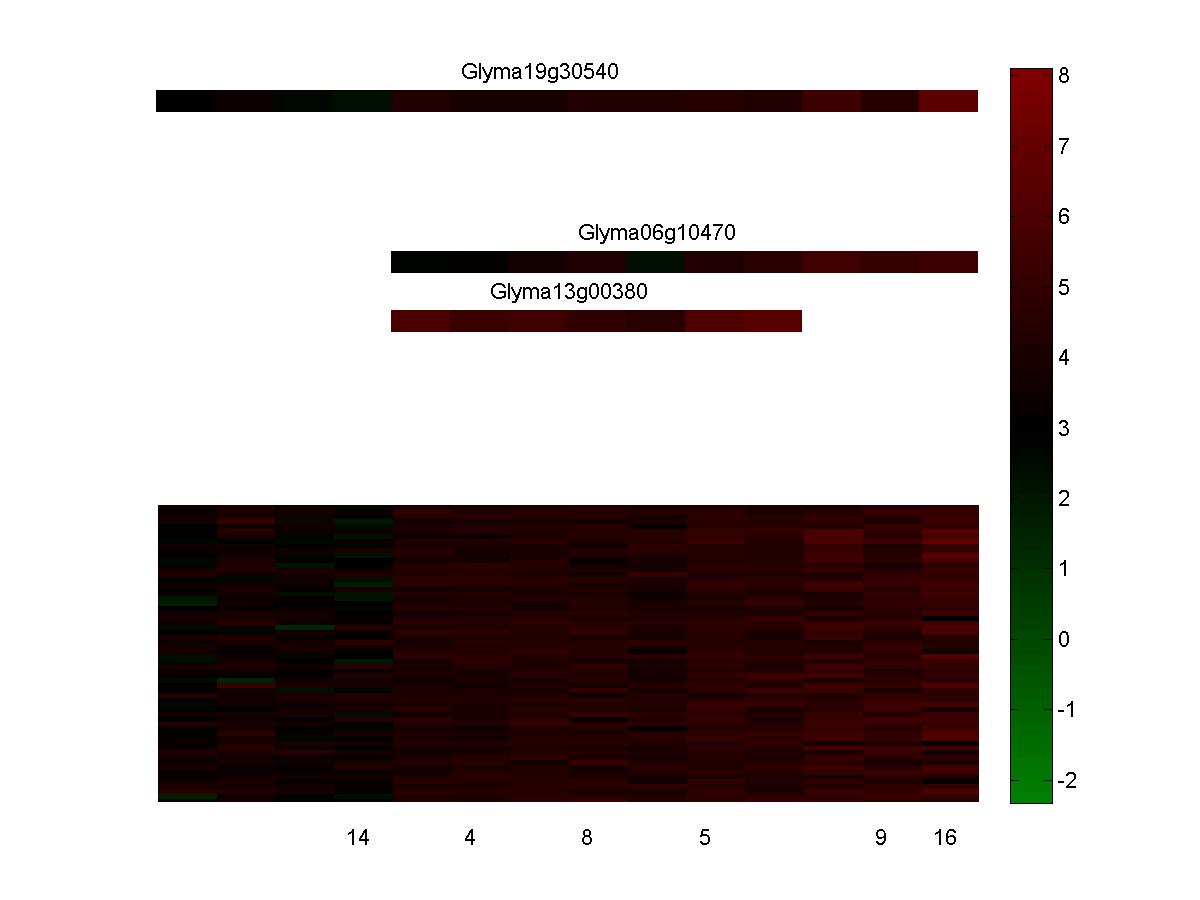


26 Glyma19g30540 NAC

26 Glyma06g10470 bHLH

26 Glyma13g00380 WRKY

Glyma13g35580 Glyma16g04810 Glyma09g00280 Glyma08g12050 Glyma08g03350

Glyma19g32690 Glyma10g04590 Glyma14g00790 Glyma14g07120 Glyma09g04280

Glyma02g47690 Glyma16g08050 Glyma17g31120 Glyma12g01050 Glyma15g37200

Glyma12g03660 Glyma05g01670 Glyma02g1737 Glyma12g09600 Glyma01g01950

Glyma07g08690 Glyma08g08030 Glyma16g25220 Glyma16g13470 Glyma01g03530

Glyma16g08010 Glyma10g17160 Glyma08g11980 Glyma13g22970 Glyma15g01210

Glyma14g08280 Glyma15g11340 Glyma08g29030 Glyma14g24270 Glyma03g32150

Glyma03g39300 Glyma02g32100 Glyma13g20550 Glyma06g08200 Glyma08g18350

Glyma15g03020 Glyma18g49070 Glyma17g11620 Glyma12g07130 Glyma07g37020

Glyma05g09400 Glyma19g30540 Glyma02g43930 Glyma13g28840 Glyma03g29560

Glyma01g39640 Glyma02g05920 Glyma15g24650 Glyma10g02050 Glyma15g15330

Glyma20g31150 Glyma11g14780 Glyma08g02960 Glyma08g03880 Glyma06g47280

Glyma03g24600 Glyma19g22310

27


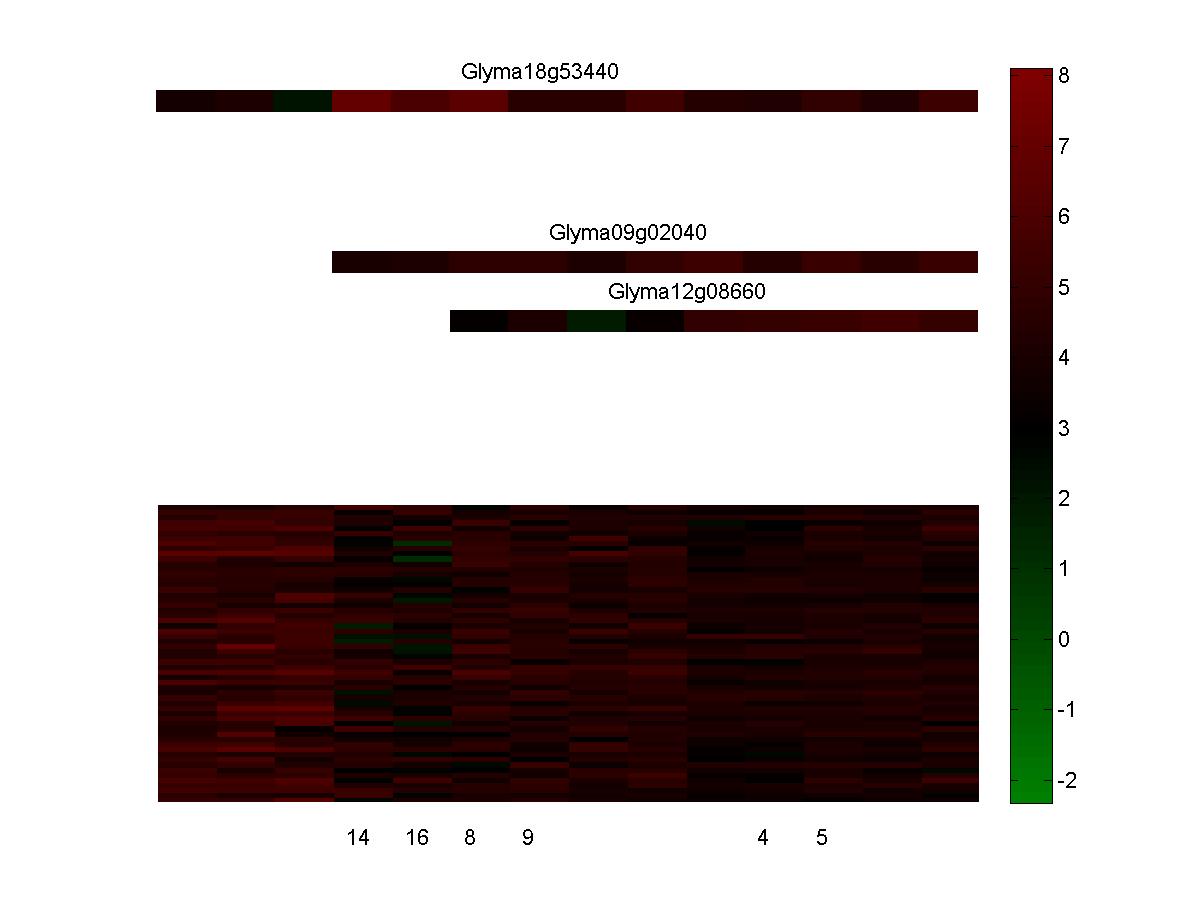


27 Glyma12g08660 C2H2 (Zn)

27 Glyma09g02040 MYB/HD-like

27 Glyma18g53440 AS2

Glyma10g28450 Glyma08g01500 Glyma05g31630 Glyma07g40370 Glyma10g32660

Glyma07g09060 Glyma13g44830 Glyma10g40620 Glyma10g01280 Glyma02g08970

Glyma10g43890 Glyma08g04540 Glyma18g46820 Glyma16g01070 Glyma11g08350

Glyma02g03160 Glyma20g29750 Glyma09g22270 Glyma13g41830 Glyma12g17230

Glyma06g12020 Glyma20g38290 Glyma19g30690 Glyma10g03450 Glyma08g23800

Glyma08g07990 Glyma06g17320 Glyma16g06840 Glyma06g25310 Glyma02g11750

Glyma18g04140 Glyma04g07540 Glyma20g26350 Glyma05g35120 Glyma02g42160

Glyma19g39370 Glyma05g23100 Glyma14g02130 Glyma18g01890 Glyma16g34180

Glyma19g02540 Glyma17g10810 Glyma03g41570 Glyma04g35820 Glyma09g38310

Glyma07g19100 Glyma19g34360 Glyma07g09050 Glyma16g00460 Glyma10g02630

Glyma18g46420 Glyma17g33050 Glyma10g28590 Glyma16g18030 Glyma11g00390

Glyma14g00540 Glyma17g02380 Glyma11g12240

28


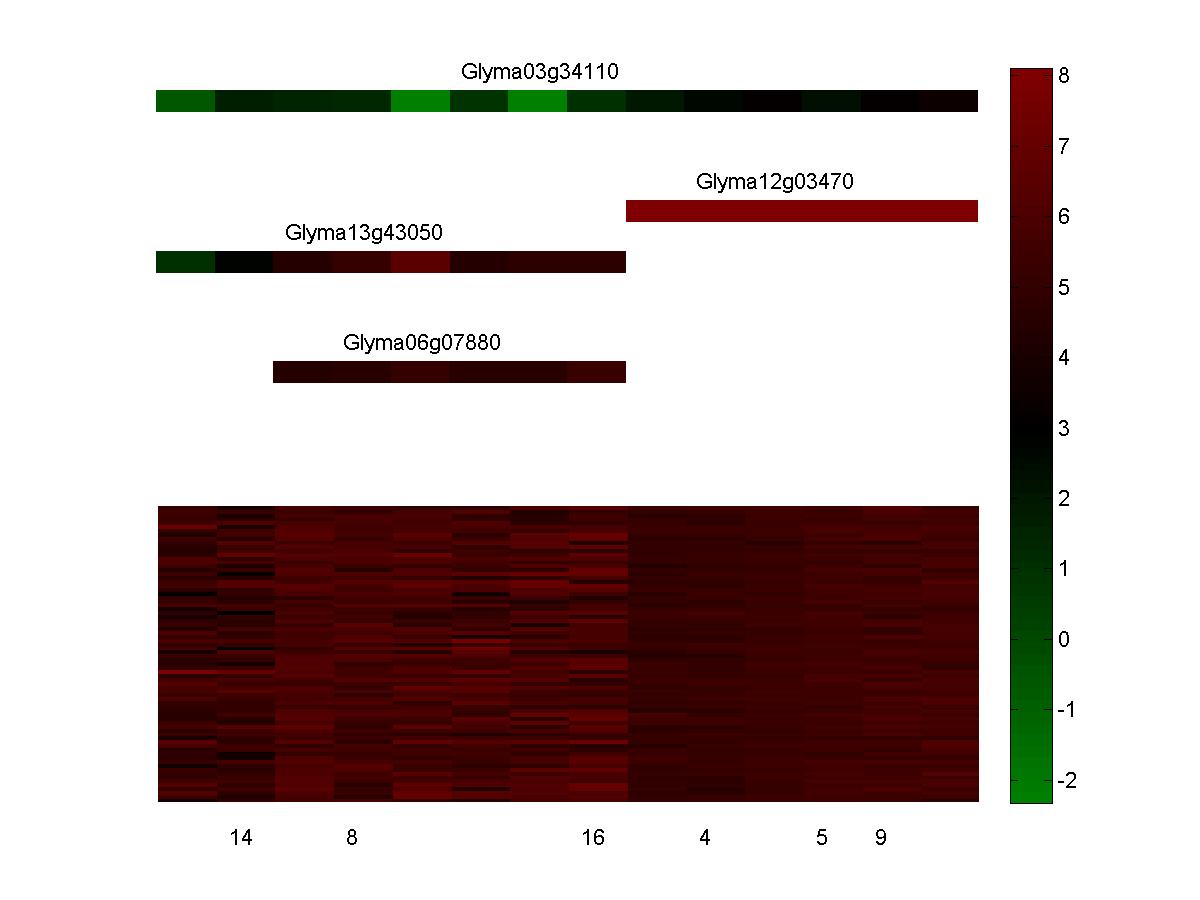


28 Glyma13g43050 AUX-IAA-ARF

28 Glyma06g07880 HTH-ARAC

28 Glyma12g03470 CSD

28 Glyma03g34110 MYB/HD-like

Glyma12g31700 Glyma08g24950 Glyma11g14190 Glyma05g30790 Glyma19g42180

Glyma11g02900 Glyma11g29420 Glyma17g16030 Glyma13g34670 Glyma10g04960

Glyma05g35880 Glyma02g46340 Glyma11g12480 Glyma02g00320 Glyma11g02250

Glyma09g35980 Glyma12g31710 Glyma07g02260 Glyma06g33940 Glyma13g00560

Glyma05g28420 Glyma08g02140 Glyma06g06390 Glyma18g01550 Glyma01g40710

Glyma04g16350 Glyma13g23060 Glyma05g02400 Glyma09g38890 Glyma01g20760

Glyma06g03230 Glyma04g05340 Glyma06g15150 Glyma16g30190 Glyma09g34000

Glyma10g38940 Glyma08g05100 Glyma05g38160 Glyma19g40090 Glyma18g47920

Glyma13g33960 Glyma07g02640 Glyma02g05870 Glyma10g42940 Glyma15g12760

Glyma04g43560 Glyma02g39510 Glyma04g04580 Glyma20g33250 Glyma12g28880

Glyma04g12240 Glyma20g22400 Glyma11g29430 Glyma19g28430 Glyma10g01210

Glyma18g05730 Glyma10g00820 Glyma01g45740 Glyma15g42690 Glyma08g06200

Glyma13g10330 Glyma02g41510 Glyma17g23830 Glyma05g01270 Glyma09g38420

Glyma13g19640 Glyma12g36290 Glyma19g35660 Glyma19g05780 Glyma16g04930

Glyma10g39750 Glyma20g20900 Glyma01g31600 Glyma16g07150 Glyma20g23690

Glyma11g08550

29


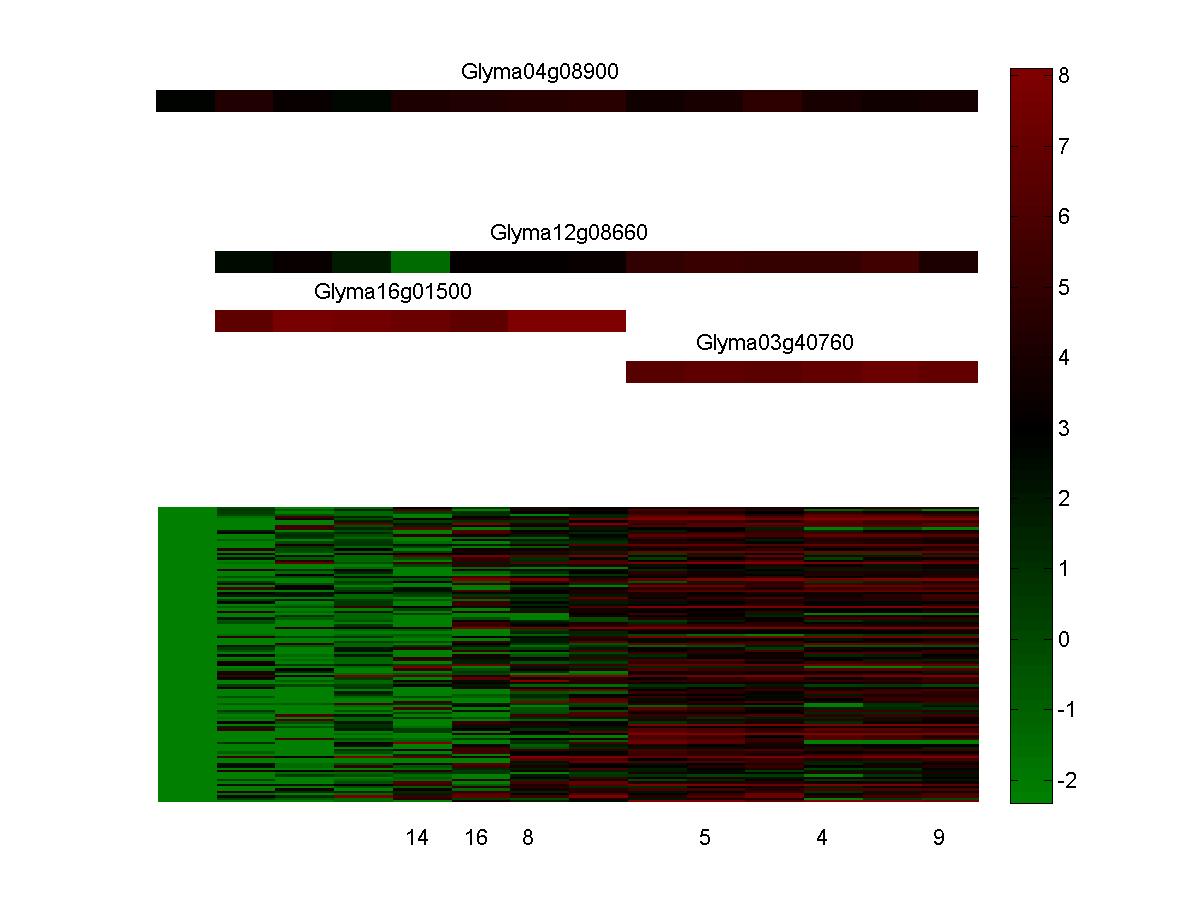


29 Glyma03g40760 AUX-IAA-ARF

29 Glyma04g08900 AP2-EREBP

29 Glyma16g01500 AP2-EREBP

29 Glyma12g08660 C2H2 (Zn)

Glyma19g02440 Glyma02g41120 Glyma17g14230 Glyma06g17640 Glyma19g01200

Glyma16g06520 Glyma06g05540 Glyma09g05340 Glyma16g07830 Glyma02g15020

Glyma08g20190 Glyma03g35700 Glyma07g15690 Glyma06g10750 Glyma18g51880

Glyma19g35190 Glyma09g37910 Glyma07g01960 Glyma06g02290 Glyma05g01300

Glyma02g25950 Glyma12g06110 Glyma05g04400 Glyma10g15130 Glyma09g02190

Glyma14g00470 Glyma07g09710 Glyma06g10740 Glyma12g00990 Glyma16g01780

Glyma08g12660 Glyma15g03110 Glyma19g44060 Glyma16g01490 Glyma09g35840

Glyma08g17300 Glyma09g04860 Glyma05g00530 Glyma01g39460 Glyma13g35320

Glyma01g22750 Glyma14g08070 Glyma06g05530 Glyma01g42660 Glyma12g30050

Glyma11g14060 Glyma08g48240 Glyma08g05490 Glyma12g31780 Glyma05g04440

Glyma04g10900 Glyma10g38550 Glyma06g45910 Glyma16g33720 Glyma04g01230

Glyma18g52410 Glyma10g30120 Glyma13g27440 Glyma11g12790 Glyma09g04850

Glyma03g37420 Glyma03g02580 Glyma10g33700 Glyma14g36700 Glyma03g21540

Glyma04g08990 Glyma06g29670 Glyma13g20260 Glyma15g11700 Glyma12g04850

Glyma19g35270 Glyma16g26940 Glyma08g45520 Glyma01g45110 Glyma12g05940

Glyma13g03600 Glyma20g27020 Glyma01g20900 Glyma16g06500 Glyma03g28080

Glyma07g00870 Glyma05g19620 Glyma13g38130 Glyma09g00700 Glyma12g34310

Glyma02g36580 Glyma11g05800 Glyma02g40890 Glyma04g12030 Glyma14g36690

Glyma02g04180 Glyma13g17750 Glyma07g37260 Glyma05g34170 Glyma10g06710

Glyma10g32820 Glyma13g23740 Glyma08g47200 Glyma13g39710 Glyma13g27130

Glyma15g10630 Glyma08g09870 Glyma17g07350 Glyma11g27720 Glyma16g27900

Glyma18g46560 Glyma19g38390 Glyma09g37780 Glyma05g25690 Glyma03g05530

Glyma10g04620 Glyma20g32470 Glyma17g15680 Glyma08g12650 Glyma17g19790

Glyma19g40460 Glyma06g03180 Glyma20g29710 Glyma11g02770 Glyma04g04470

Glyma01g42800 Glyma11g12810 Glyma05g03750 Glyma10g30110 Glyma17g14860

Glyma15g13880 Glyma02g46670 Glyma04g33940

30


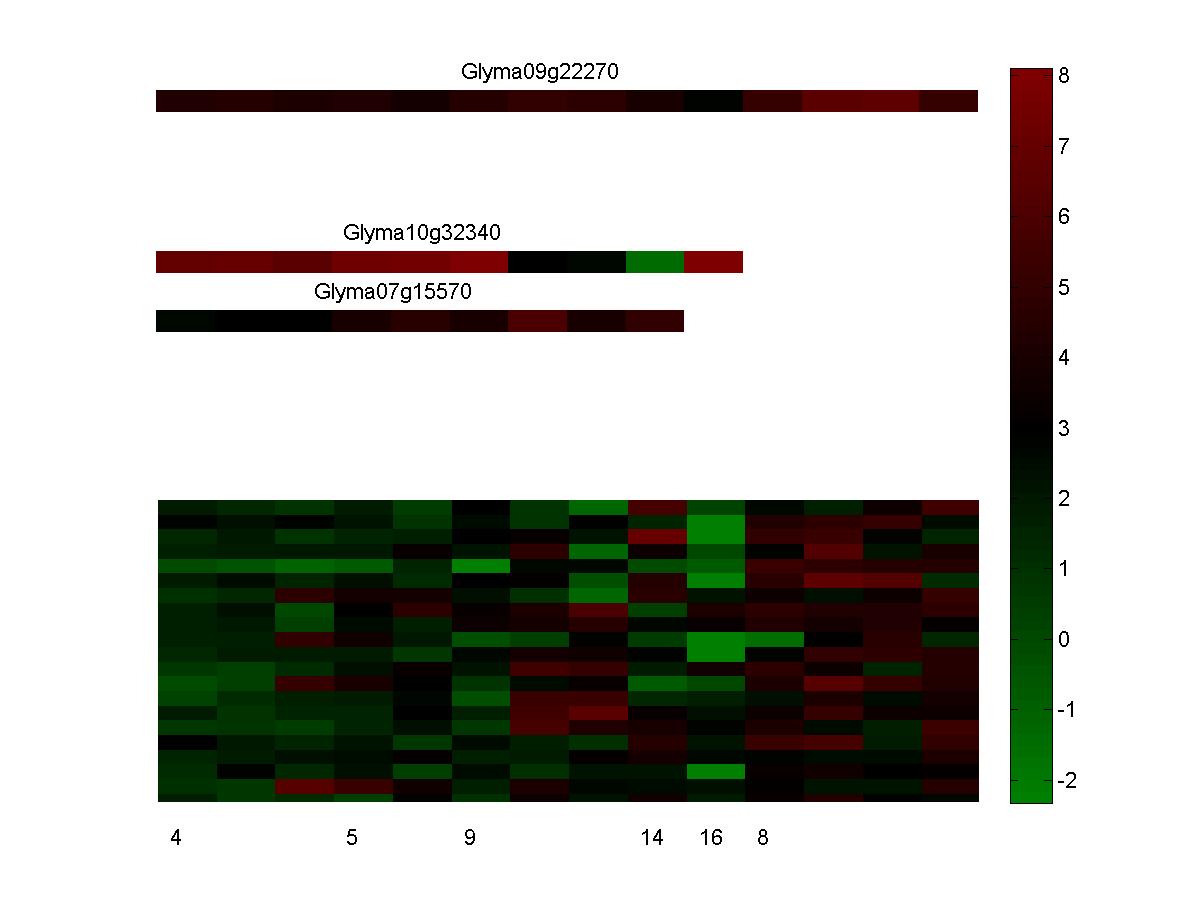


30 Glyma07g15570 SSB protein

30 Glyma10g32340 AUX-IAA-ARF

30 Glyma09g22270 ZIM

Glyma03g42060 Glyma18g05160 Glyma15g40290 Glyma07g36180 Glyma15g40890

Glyma17g17310 Glyma02g42250 Glyma05g14800 Glyma05g22660 Glyma16g04440

Glyma19g39690 Glyma10g01690 Glyma18g51100 Glyma16g06740 Glyma09g03730

Glyma11g13130 Glyma17g21540 Glyma20g29200 Glyma02g13730 Glyma11g12980

Glyma13g39310

31


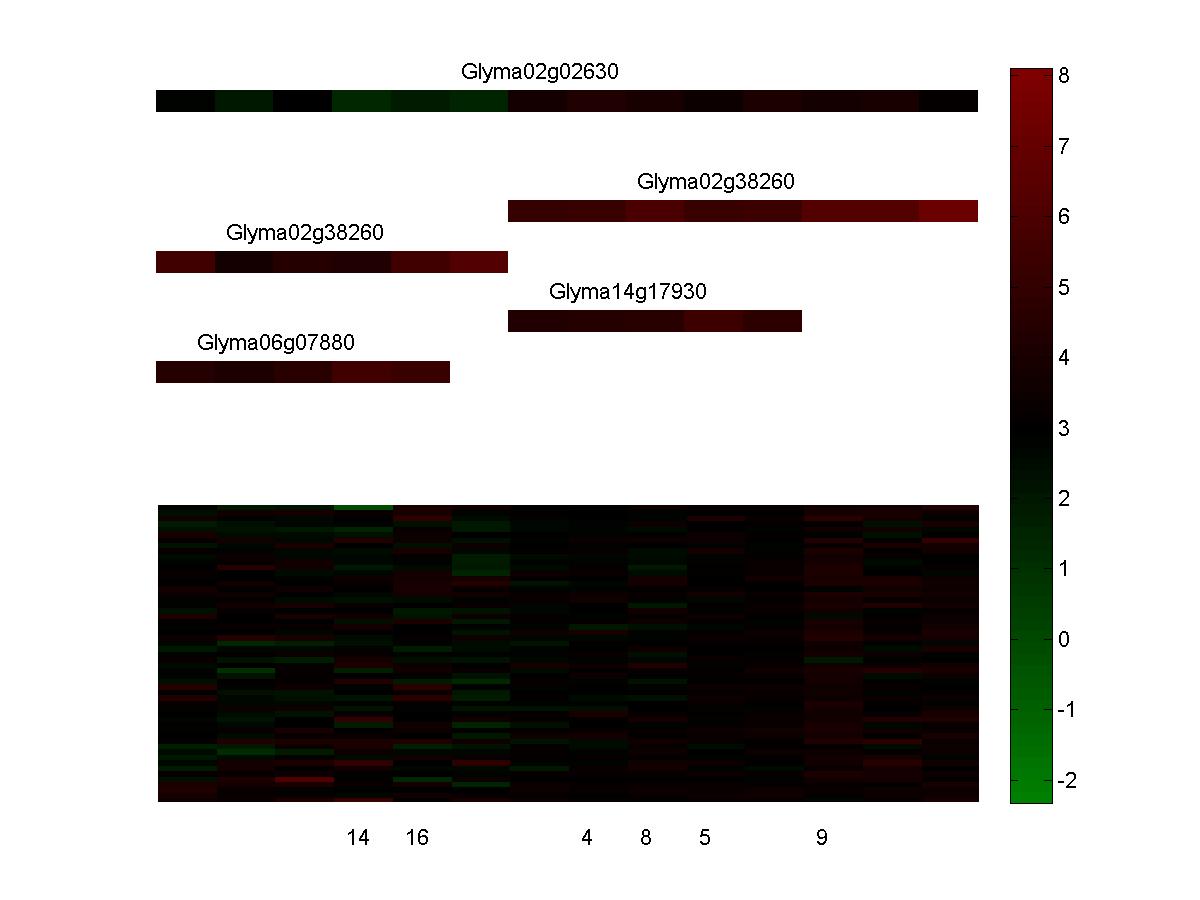


31 Glyma02g02630 Homeodomain/HOMEOBOX

31 Glyma02g38260 AUX-IAA-ARF

31 Glyma14g17930 CCHC (Zn)

31 Glyma06g07880 HTH-ARAC

Glyma11g10150 Glyma15g10650 Glyma04g07930 Glyma11g03510 Glyma01g35130

Glyma14g33330 Glyma10g21950 Glyma08g14430 Glyma08g42380 Glyma17g14250

Glyma20g03900 Glyma05g01690 Glyma15g14320 Glyma19g44940 Glyma14g03780

Glyma19g05550 Glyma03g00350 Glyma08g26480 Glyma01g30910 Glyma03g40660

Glyma15g13480 Glyma01g00690 Glyma01g01930 Glyma11g35110 Glyma03g41400

Glyma04g08800 Glyma14g10520 Glyma10g02400 Glyma08g03130 Glyma11g07910

Glyma05g13110 Glyma10g41100 Glyma20g29960 Glyma14g07830 Glyma09g08580

Glyma10g40140 Glyma08g06860 Glyma03g41110 Glyma08g08730 Glyma15g07490

Glyma04g01590 Glyma09g07540 Glyma14g01170 Glyma12g22360 Glyma05g38070

Glyma16g26110 Glyma08g20900 Glyma05g30430 Glyma08g09940 Glyma07g39240

Glyma09g36540 Glyma08g11930 Glyma14g07140 Glyma05g26960 Glyma02g47520

32


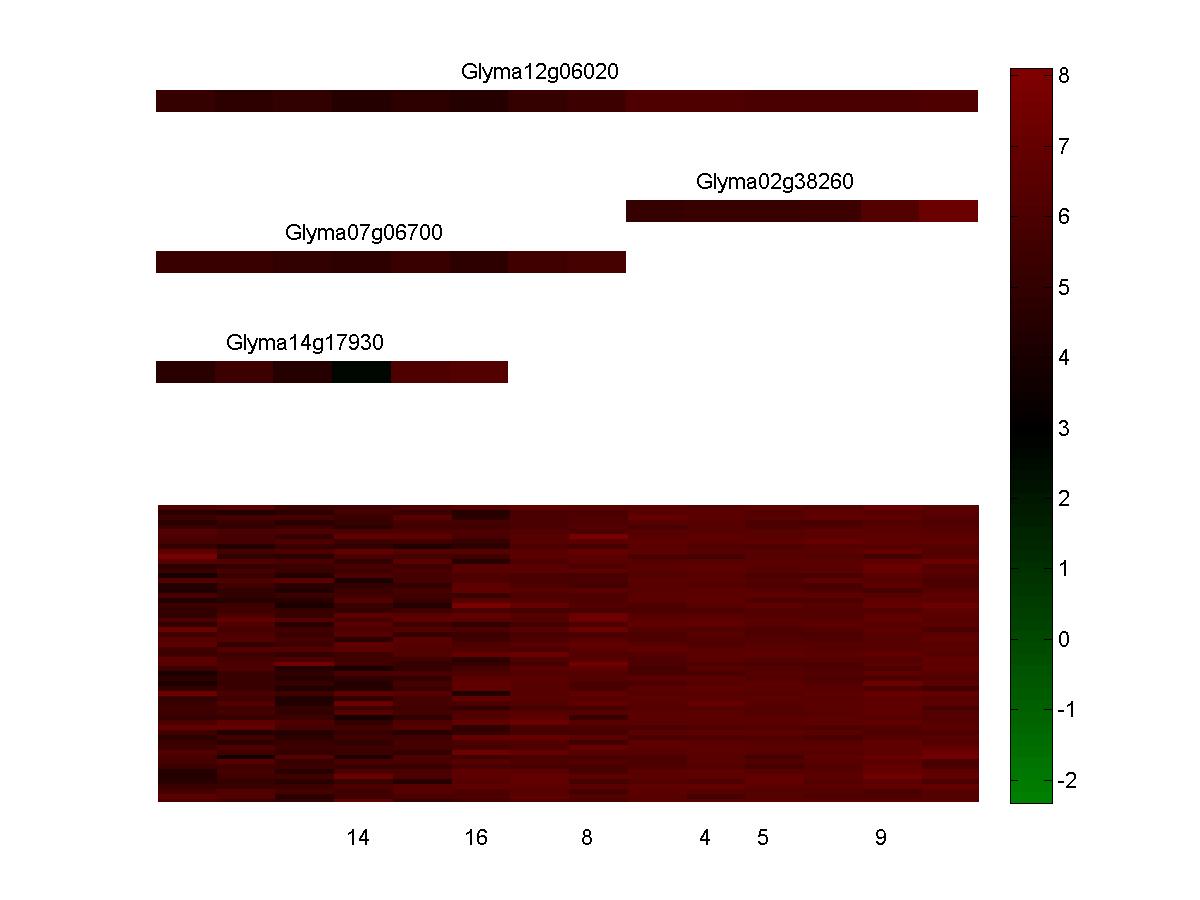


32 Glyma02g38260 AUX-IAA-ARF

32 Glyma14g17930 CCHC (Zn)

32 Glyma07g06700 BTB/POZ

32 Glyma12g06020 zf-A20

Glyma02g35990 Glyma10g35700 Glyma02g40820 Glyma09g42010 Glyma05g26530

Glyma19g37840 Glyma18g19420 Glyma08g06010 Glyma20g31790 Glyma10g02290

Glyma11g34980 Glyma04g42250 Glyma03g40860 Glyma10g44200 Glyma05g37300

Glyma11g12120 Glyma13g02750 Glyma07g30160 Glyma09g03020 Glyma16g23230

Glyma07g00390 Glyma05g23220 Glyma08g01990 Glyma03g25190 Glyma16g27210

Glyma17g13640 Glyma19g36080 Glyma19g37970 Glyma03g37250 Glyma14g33210

Glyma03g38730 Glyma10g22720 Glyma19g38600 Glyma12g31850 Glyma14g39160

Glyma03g41940 Glyma05g28060 Glyma18g03390 Glyma19g39860 Glyma10g24590

Glyma14g38620 Glyma10g35750 Glyma06g11010 Glyma18g47950 Glyma19g25870

Glyma16g04580 Glyma13g28880 Glyma10g28530 Glyma04g37030 Glyma06g47890

Glyma19g44620 Glyma19g41320 Glyma17g15970 Glyma05g28080 Glyma03g28760

Glyma13g33190 Glyma12g11760 Glyma15g10160 Glyma14g36610 Glyma05g28730

Glyma05g08670

33


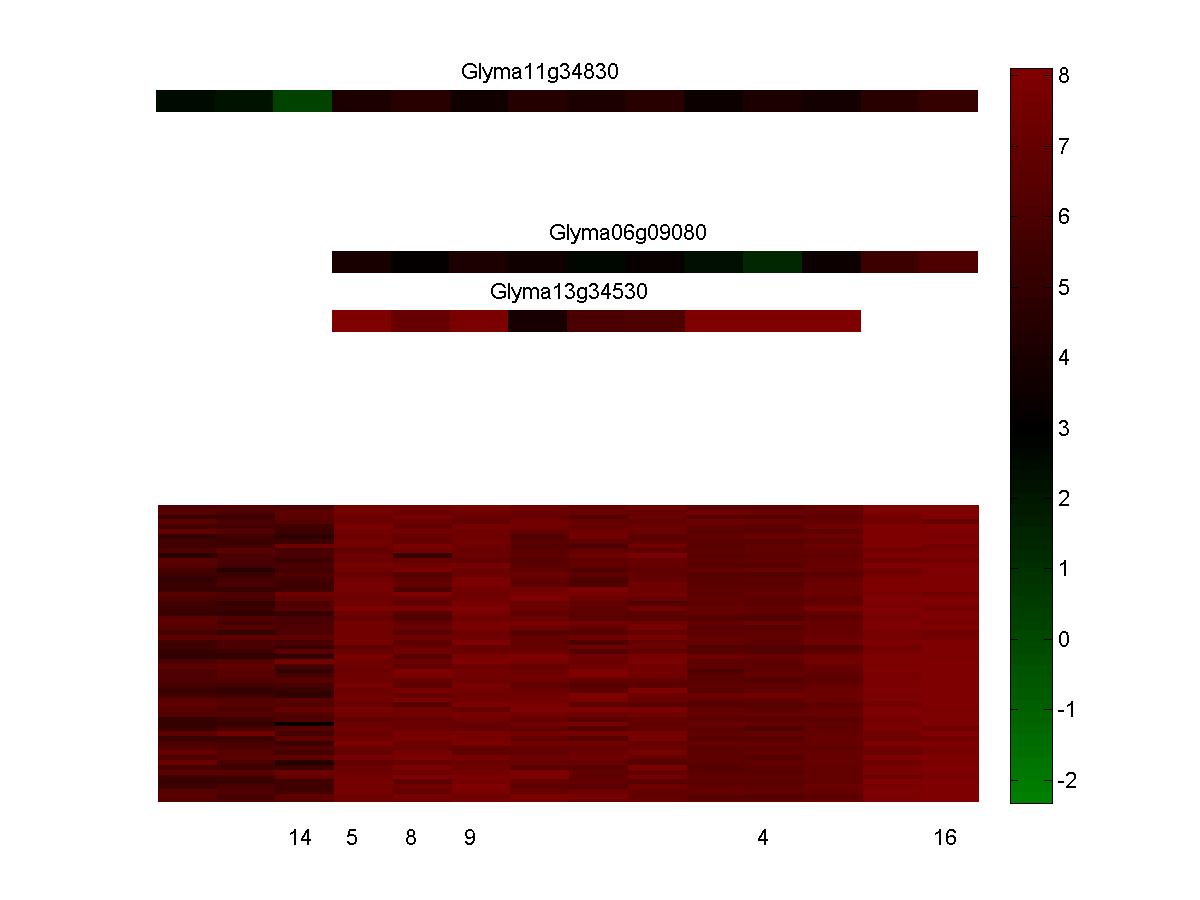


33 Glyma06g09080 C2C2 (Zn) GATA

33 Glyma11g34830 LIM

33 Glyma13g34530 C2H2 (Zn)

Glyma04g36860 Glyma10g40150 Glyma15g15800 Glyma14g09300 Glyma03g36470

Glyma06g18800 Glyma08g09200 Glyma02g00810 Glyma01g02720 Glyma04g40430

Glyma05g03880 Glyma17g14170 Glyma03g32980 Glyma08g03480 Glyma19g37520

Glyma08g05610 Glyma06g05410 Glyma18g53610 Glyma01g26950 Glyma14g35410

Glyma18g32680 Glyma16g23730 Glyma08g02410 Glyma03g26100 Glyma06g18110

Glyma01g43110 Glyma04g06700 Glyma14g06170 Glyma05g04870 Glyma08g03150

Glyma08g16130 Glyma06g07320 Glyma16g03760 Glyma03g25790 Glyma01g00740

Glyma17g10710 Glyma08g46850 Glyma14g36970 Glyma05g26290 Glyma04g40720

Glyma10g41330 Glyma05g3642 Glyma04g16660 Glyma02g43790 Glyma08g46070

Glyma13g33410 Glyma02g38450 Glyma02g10170 Glyma04g42690 Glyma06g14080

Glyma02g07430 Glyma08g22600 Glyma07g02270 Glyma12g08990 Glyma07g04890

Glyma04g09820 Glyma15g13140 Glyma10g37960 Glyma20g25920 Glyma17g02200

Glyma18g14980 Glyma05g34570

34


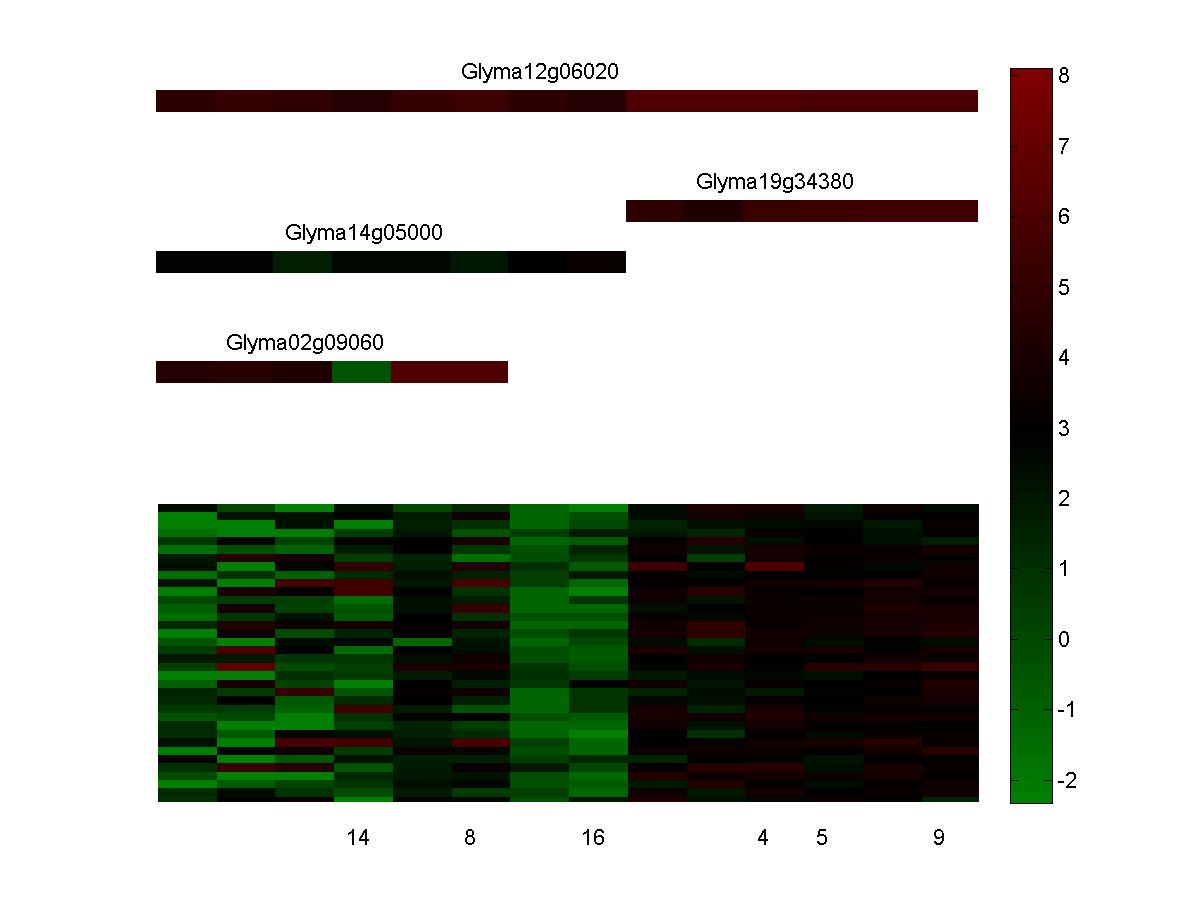


34 Glyma14g05000 BTB/POZ

34 Glyma12g06020 zf-A20

34 Glyma19g34380 AUX-IAA-ARF

34 Glyma02g09060 MYB/HD-like

Glyma06g11580 Glyma18g04580 Glyma06g05620 Glyma10g35410 Glyma14g38730

Glyma18g07140 Glyma06g18390 Glyma12g36360 Glyma13g28710 Glyma05g34760

Glyma08g16580 Glyma17g11340 Glyma15g15680 Glyma06g42960 Glyma20g26420

Glyma08g25600 Glyma05g30960 Glyma10g35380 Glyma18g41420 Glyma14g37980

Glyma16g28970 Glyma11g33450 Glyma13g42610 Glyma18g06380 Glyma08g13900

Glyma18g02430 Glyma04g08090 Glyma20g26600 Glyma03g36760 Glyma08g27720

Glyma08g03240 Glyma04g02750 Glyma13g29450 Glyma13g42620 Glyma15g11400

Glyma03g24020

35


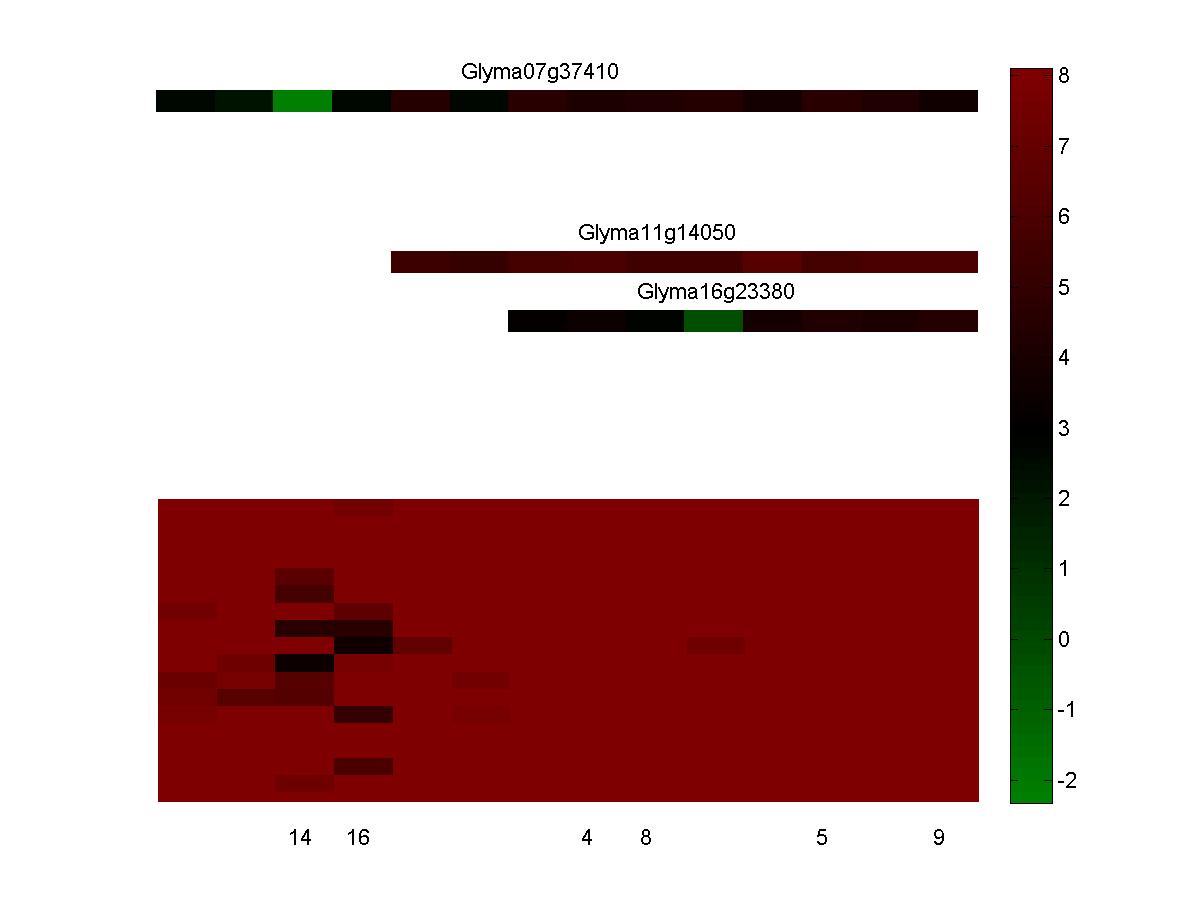


35 Glyma16g23380 TPR

35 Glyma07g37410 AP2-EREBP

35 Glyma11g14050 zf-A20

Glyma19g36440 Glyma14g06680 Glyma09g08100 Glyma10g39780 Glyma20g27950

Glyma19g29210 Glyma15g19580 Glyma20g27940 Glyma17g35720 Glyma16g04190

Glyma13g40100 Glyma14g40670 Glyma17g23900 Glyma03g34310 Glyma02g42220

Glyma19g37000 Glyma04g01130 Glyma17g37400

36


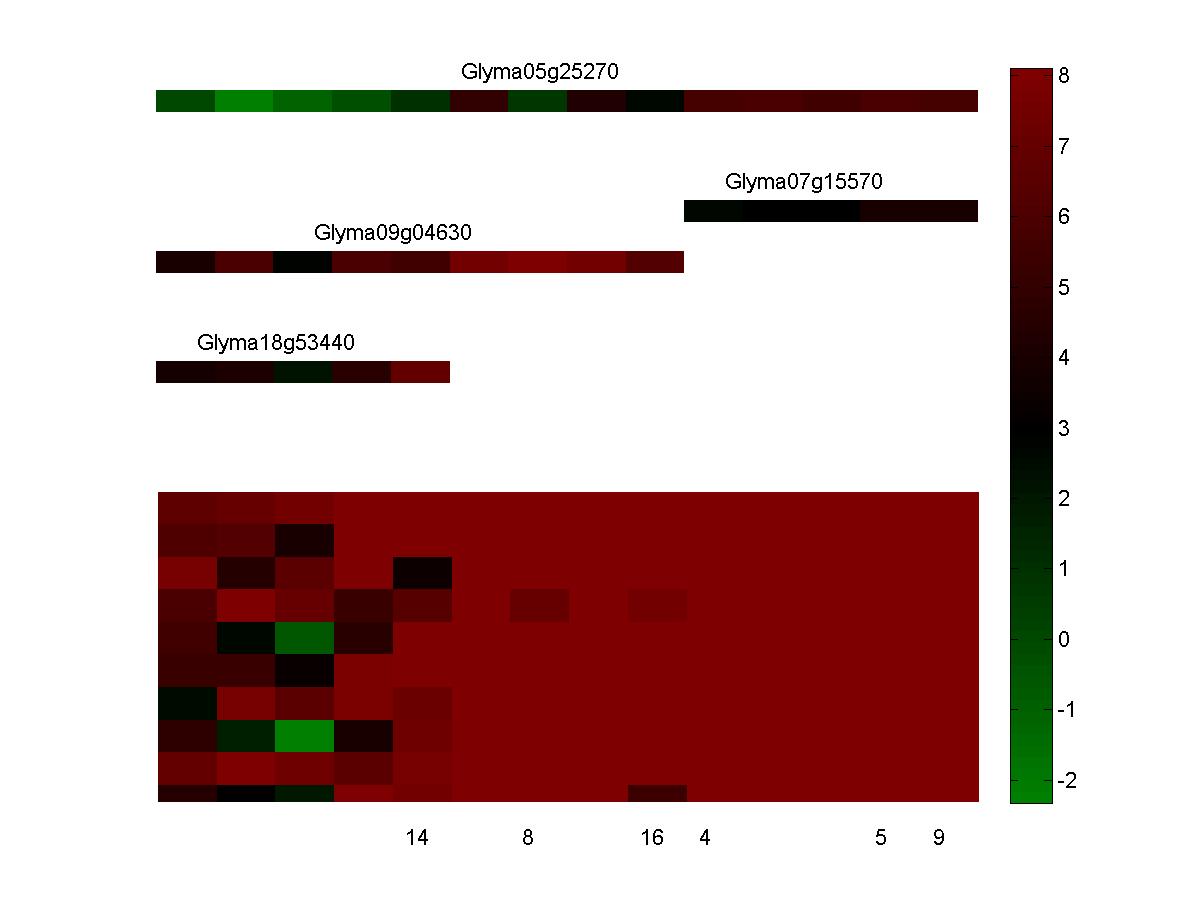


36 Glyma18g53440 AS2

36 Glyma09g04630 AP2-EREBP

36 Glyma07g15570 SSB protein

36 Glyma05g25270 WRKY

Glyma09g12200 Glyma10g35090 Glyma13g42330 Glyma12g00390 Glyma08g26150

Glyma11g03690 Glyma07g00900 Glyma10g35080 Glyma05g37730 Glyma07g00910

37


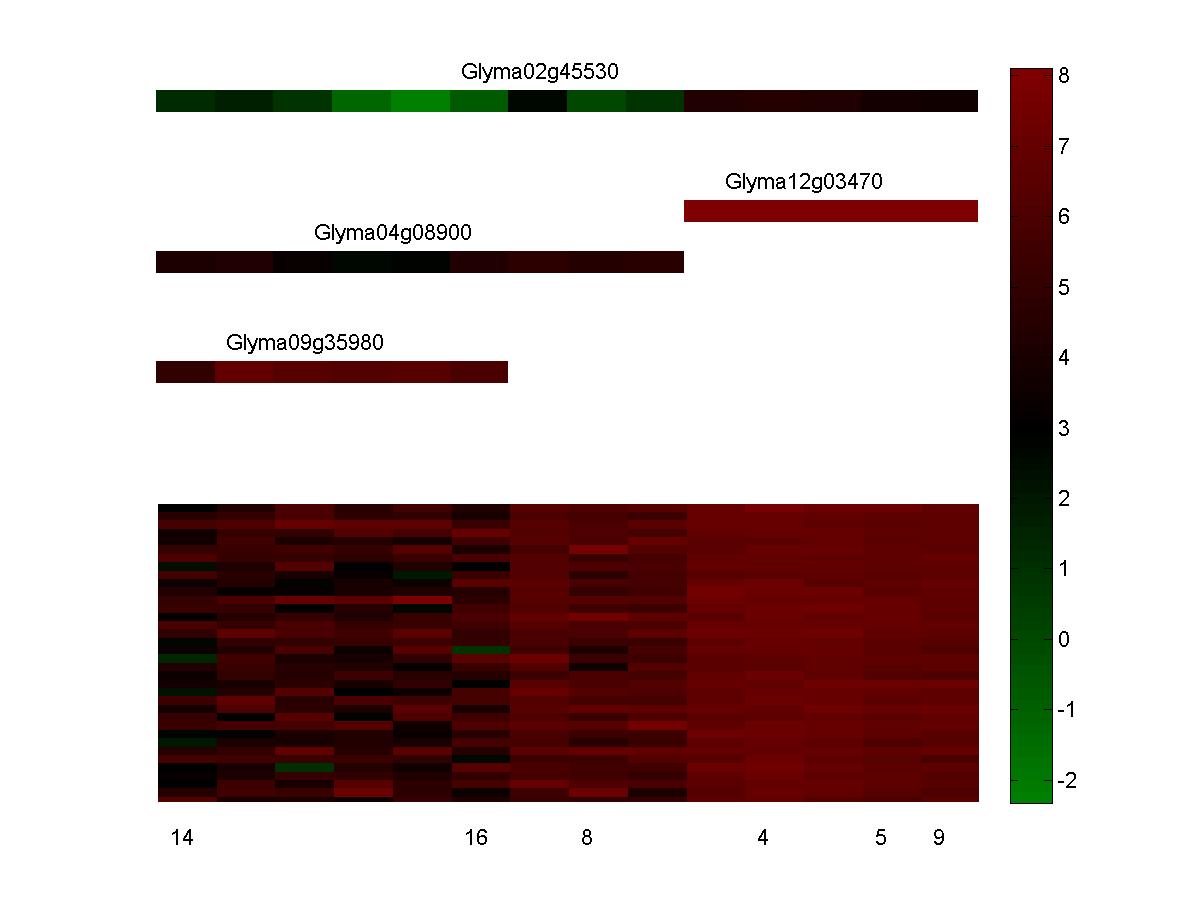


37 Glyma02g45530 WRKY

37 Glyma12g03470 CSD

37 Glyma04g08900 AP2-EREBP

37 Glyma09g35980 C3H-type1(Zn)

Glyma13g06050 Glyma09g11770 Glyma16g01790 Glyma07g30880 Glyma09g14380

Glyma01g07070 Glyma04g09510 Glyma18g50760 Glyma08g47790 Glyma09g12320

Glyma13g19830 Glyma05g01390 Glyma11g37920 Glyma01g44290 Glyma13g01580

Glyma16g22650 Glyma11g29350 Glyma15g02700 Glyma12g10240 Glyma13g39600

Glyma03g16510 Glyma13g17220 Glyma03g05620 Glyma19g30600 Glyma14g02070

Glyma19g02180 Glyma18g17350 Glyma01g44600 Glyma11g09190 Glyma03g40680

Glyma10g35550 Glyma04g03110 Glyma04g41090 Glyma17g07190 Glyma09g33960

Glyma03g01760

38


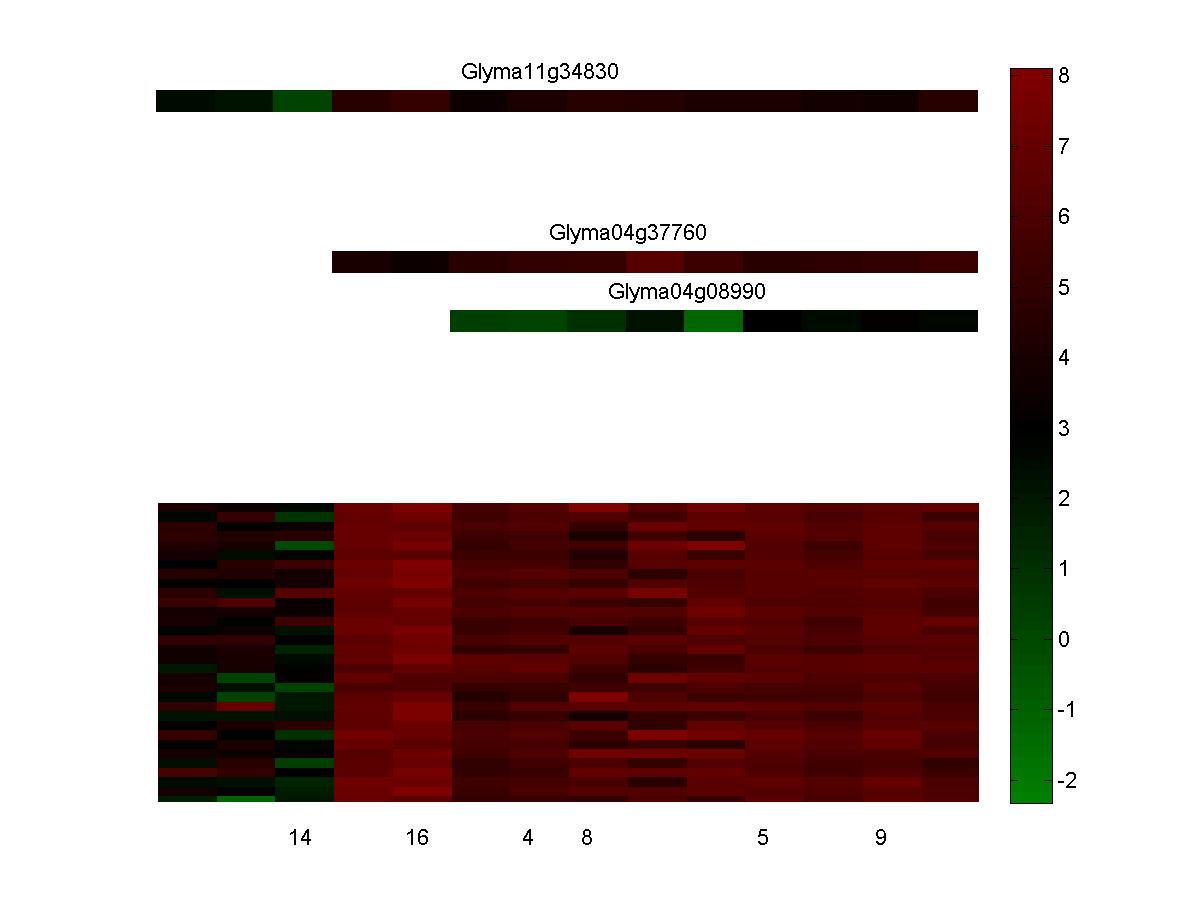


38 Glyma04g37760 AUX-IAA-ARF

38 Glyma04g08990 C2C2 (Zn) GATA

38 Glyma11g34830 LIM

Glyma15g42140 Glyma13g05810 Glyma19g03500 Glyma11g05480 Glyma17g07070

Glyma01g20460 Glyma17g17970 Glyma11g05470 Glyma16g01650 Glyma02g47790

Glyma17g04060 Glyma13g19650 Glyma17g01580 Glyma17g14750 Glyma10g11620

Glyma08g45810 Glyma05g31310 Glyma16g03410 Glyma19g42240 Glyma13g21050

Glyma07g38460 Glyma04g00450 Glyma01g42450 Glyma16g09760 Glyma01g37270

Glyma11g05900 Glyma02g13330 Glyma09g32850 Glyma20g04490 Glyma16g23900

Glyma06g48360 Glyma08g19290

39


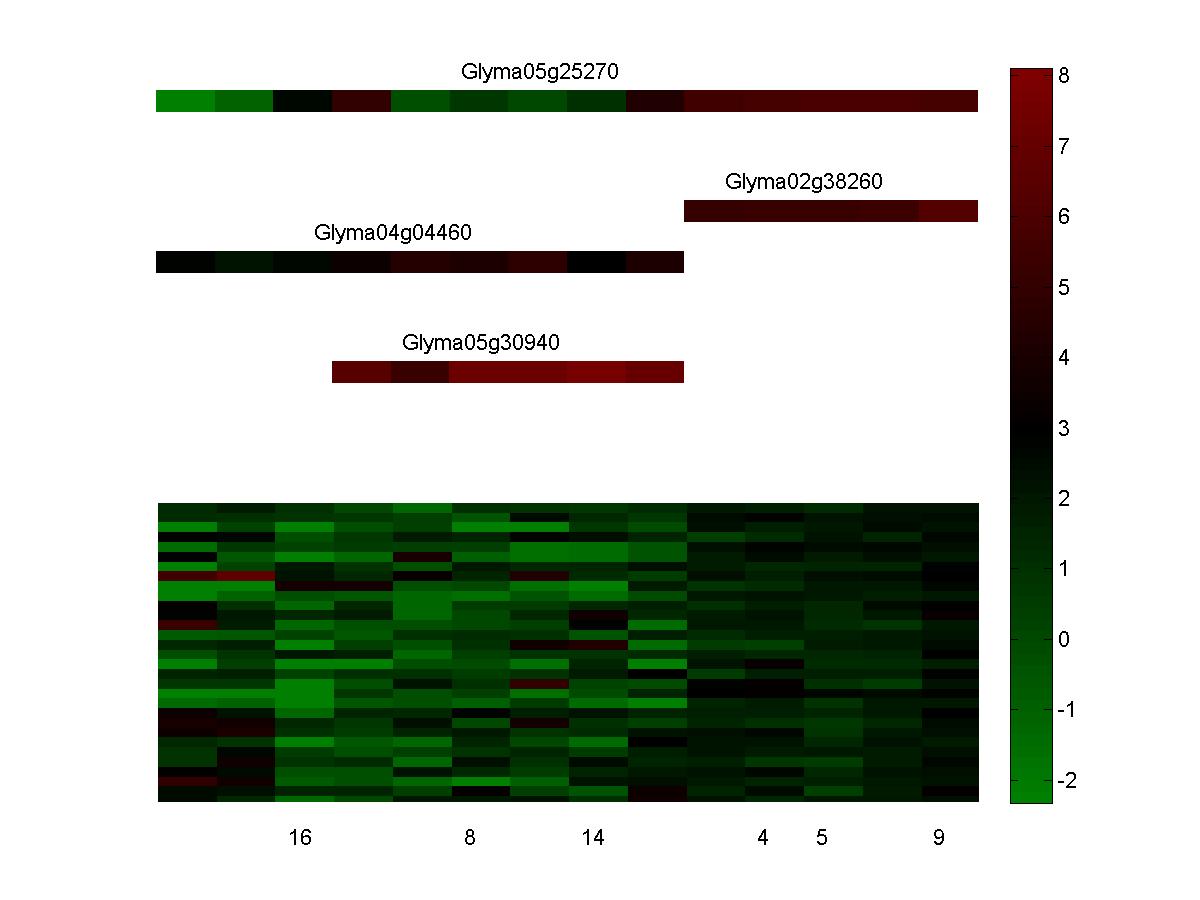


39 Glyma05g30940 Homeodomain/HOMEOBOX

39 Glyma02g38260 AUX-IAA-ARF

39 Glyma05g25270 WRKY

39 Glyma04g04460 HTH-ARAC

Glyma01g23140 Glyma03g39730 Glyma12g31070 Glyma15g02880 Glyma07g35380

Glyma10g10480 Glyma04g41310 Glyma06g47800 Glyma05g03070 Glyma15g17410

Glyma11g04370 Glyma17g11730 Glyma12g34990 Glyma01g34700 Glyma19g28480

Glyma12g34190 Glyma09g10010 Glyma06g16280 Glyma18g05450 Glyma03g01980

Glyma15g08180 Glyma18g19710 Glyma18g02900 Glyma20g03210 Glyma16g07110

Glyma19g03010 Glyma18g49910 Glyma16g06080 Glyma02g02740 Glyma13g30030

Glyma10g37380

40


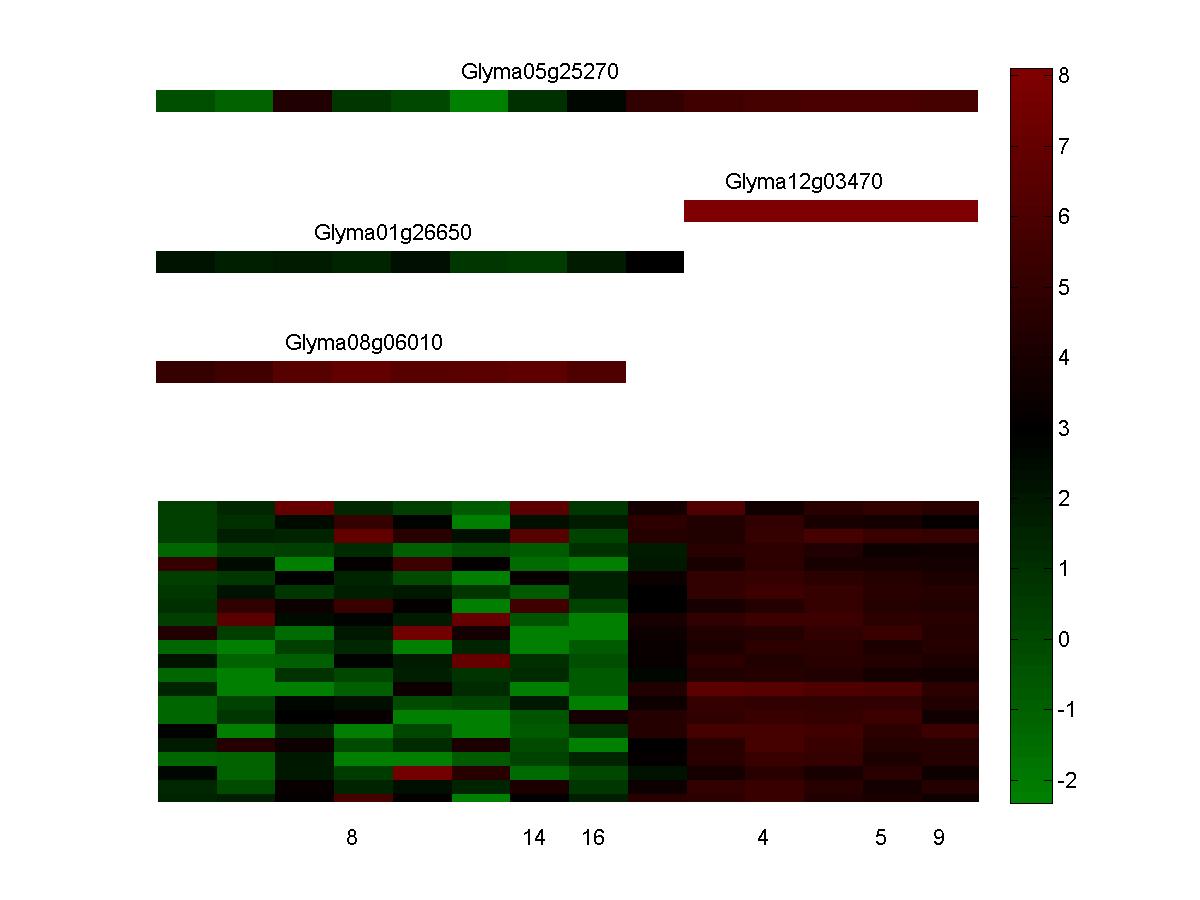


40 Glyma12g03470 CSD

40 Glyma08g06010 TPR

40 Glyma05g25270 WRKY

40 Glyma01g26650 MYB/HD-like

Glyma18g44840 Glyma16g32440 Glyma10g28510 Glyma05g02690 Glyma09g40300

Glyma07g32810 Glyma06g41510 Glyma02g45530 Glyma14g05390 Glyma04g08170

Glyma19g40190 Glyma01g33170 Glyma12g31080 Glyma10g38080 Glyma09g08470

Glyma10g26220 Glyma05g02950 Glyma03g38130 Glyma13g25530 Glyma13g42630

Glyma13g25540 Glyma13g27010

41


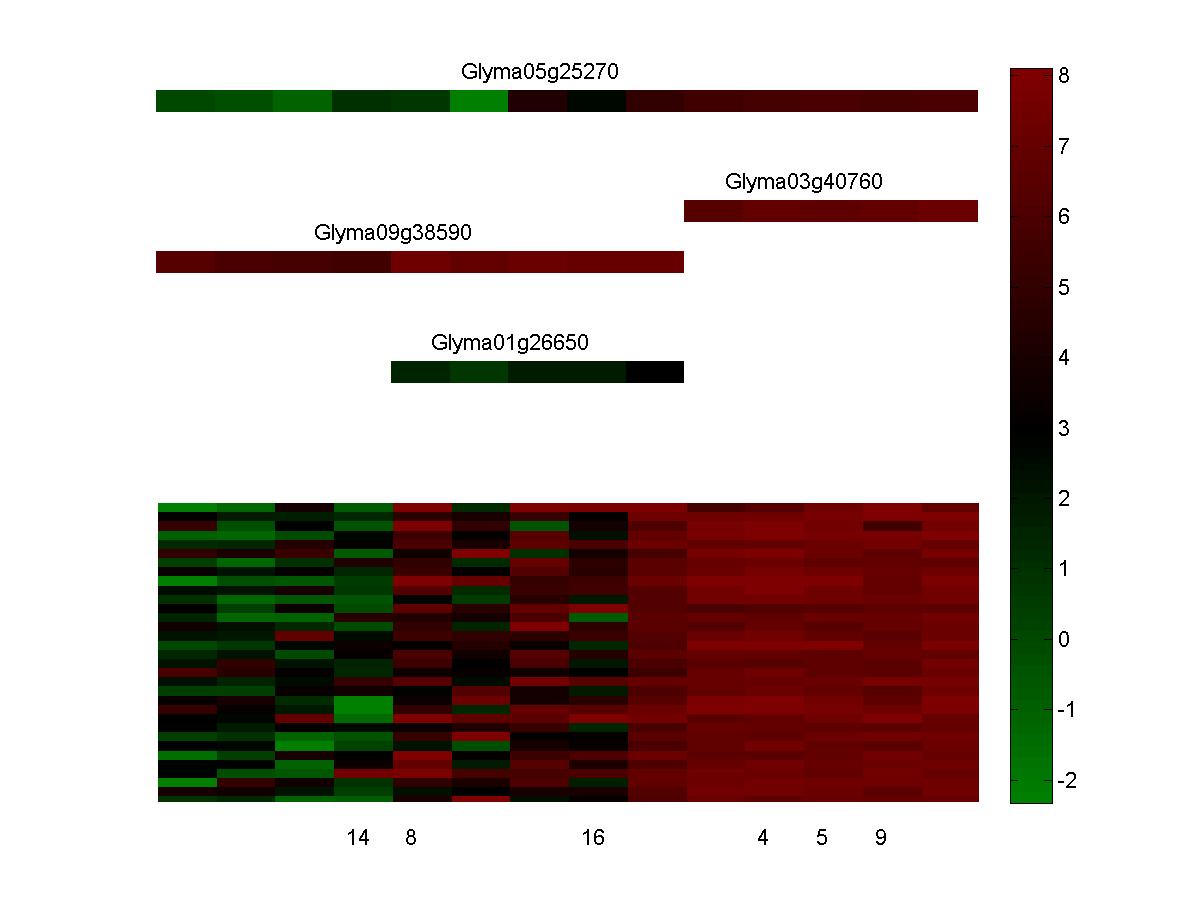


41 Glyma05g25270 WRKY

41 Glyma03g40760 AUX-IAA-ARF

41 Glyma09g38590 C2H2 (Zn)

41 Glyma01g26650 MYB/HD-like

Glyma08g12460 Glyma04g42240 Glyma03g04920 Glyma11g14130 Glyma09g22310

Glyma10g33650 Glyma15g06790 Glyma03g38630 Glyma06g12340 Glyma15g01470

Glyma12g36320 Glyma03g02410 Glyma03g04990 Glyma01g42670 Glyma14g35340

Glyma05g32160 Glyma13g33890 Glyma03g15130 Glyma15g17530 Glyma03g19260

Glyma17g14930 Glyma16g33270 Glyma02g18380 Glyma12g36330 Glyma02g08950

Glyma08g24750 Glyma18g06840 Glyma06g08910 Glyma19g01940 Glyma10g32340

Glyma15g42780 Glyma01g02330 Glyma15g37520

42


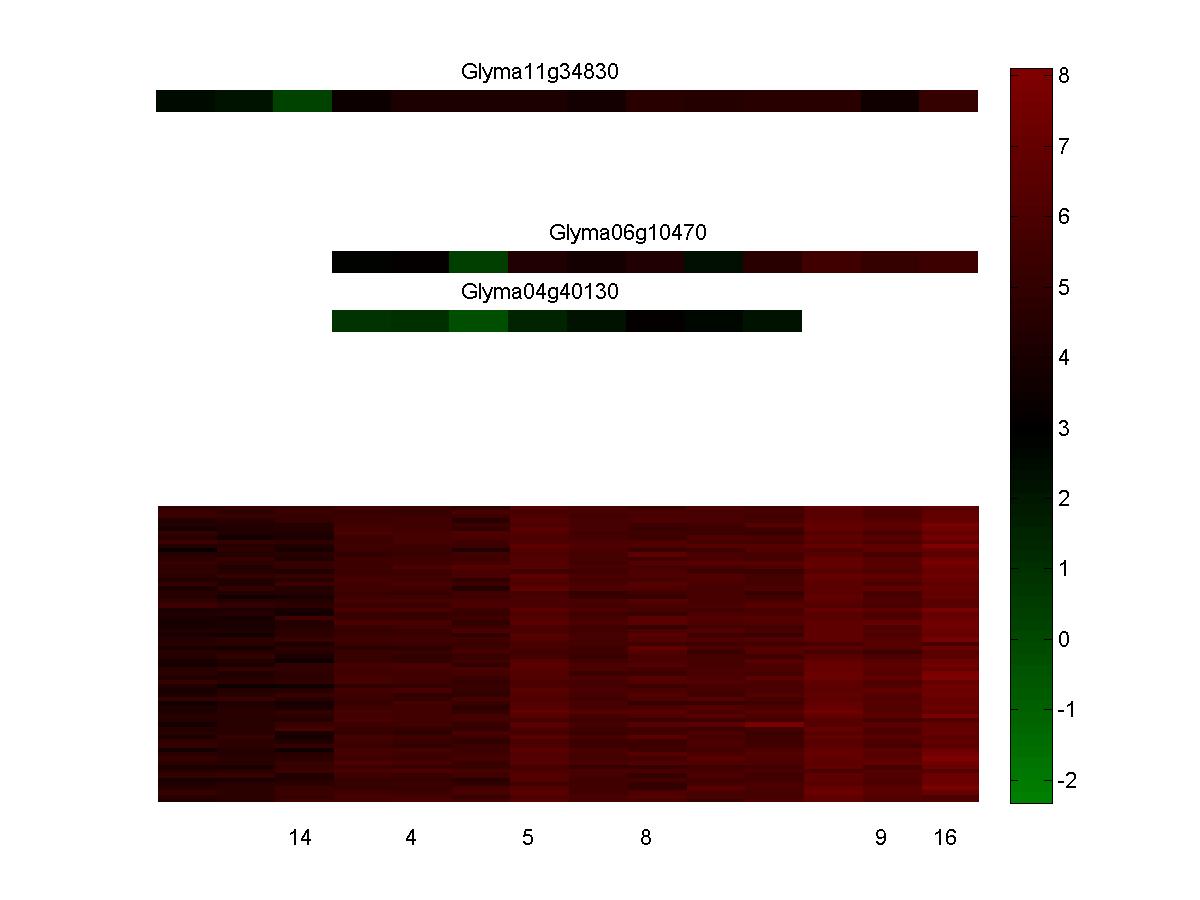


42 Glyma06g10470 bHLH

42 Glyma11g34830 LIM

42 Glyma04g40130 WRKY

Glyma06g14960 Glyma11g00450 Glyma15g00610 Glyma18g44990 Glyma10g37840

Glyma05g27940 Glyma15g17010 Glyma12g29040 Glyma10g08910 Glyma11g20630

Glyma09g34760 Glyma07g05220 Glyma10g06040 Glyma04g40980 Glyma17g11430

Glyma15g05670 Glyma09g34310 Glyma13g42090 Glyma12g03230 Glyma08g40110

Glyma16g01460 Glyma06g32680 Glyma04g14640 Glyma13g31900 Glyma02g02140

Glyma06g46690 Glyma07g13730 Glyma18g18050 Glyma09g38530 Glyma08g05470

Glyma14g06310 Glyma18g01580 Glyma12g02750 Glyma03g34700 Glyma17g03550

Glyma12g29550 Glyma15g23220 Glyma03g34830 Glyma13g37610 Glyma03g37460

Glyma16g33230 Glyma11g19220 Glyma19g03520 Glyma08g22550 Glyma04g40550

Glyma07g39980 Glyma13g31650 Glyma11g11760 Glyma05g34980 Glyma11g36090

Glyma07g01540 Glyma19g42720 Glyma11g33480 Glyma15g15020 Glyma20g37190

Glyma11g27000 Glyma09g35650 Glyma09g34100 Glyma18g01110 Glyma07g02720

Glyma13g21520 Glyma02g44460 Glyma10g34220 Glyma14g40120 Glyma20g24400

Glyma20g09810 Glyma09g01270 Glyma19g43190 Glyma07g33800 Glyma07g37060

43


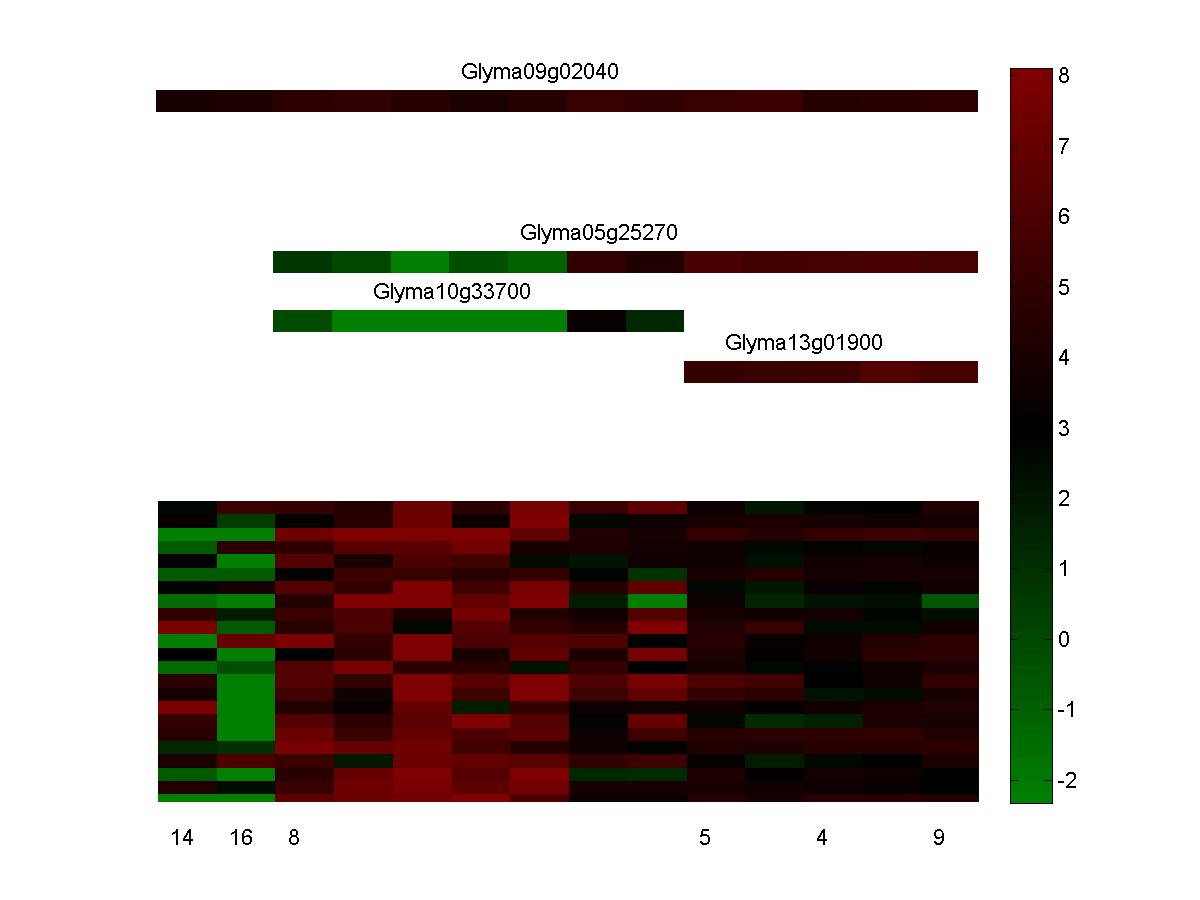


43 Glyma13g01900 TPR

43 Glyma10g33700 AP2-EREBP

43 Glyma09g02040 MYB/HD-like

43 Glyma05g25270 WRKY

Glyma08g44310 Glyma04g08410 Glyma12g09730 Glyma13g41650 Glyma13g24360

Glyma11g21640 Glyma09g36720 Glyma03g30890 Glyma11g30010 Glyma04g02330

Glyma08g18080 Glyma02g08260 Glyma08g43340 Glyma08g43330 Glyma10g36200

Glyma11g21610 Glyma14g10620 Glyma16g27350 Glyma15g40450 Glyma14g10890

Glyma19g28990 Glyma11g21620 Glyma10g29190

44


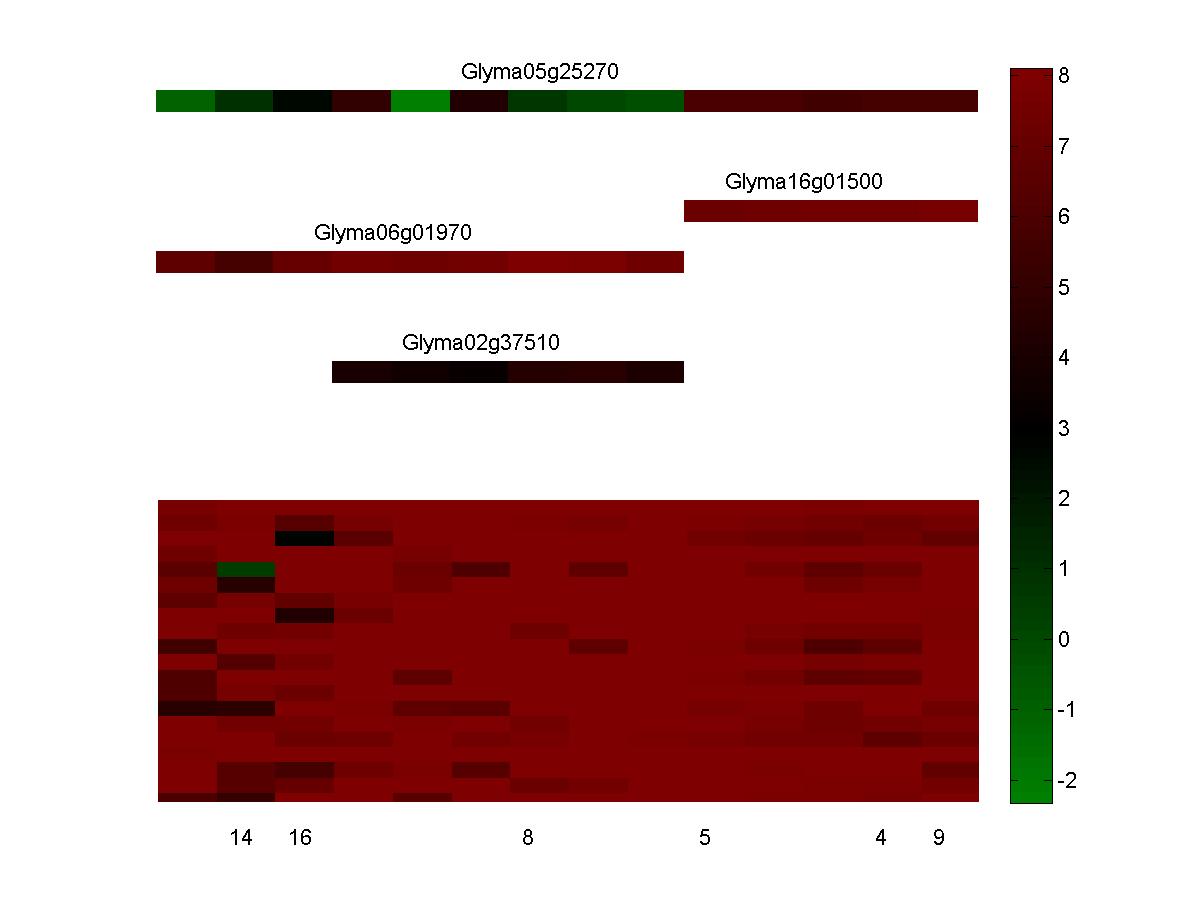


44 Glyma05g25270 WRKY

44 Glyma16g01500 AP2-EREBP

44 Glyma06g01970 HMG

44 Glyma02g37510 bHLH

Glyma17g04340 Glyma04g01380 Glyma03g27970 Glyma06g03050 Glyma09g01320

Glyma09g04950 Glyma13g27570 Glyma05g31610 Glyma20g27280 Glyma18g39690

Glyma15g32800 Glyma11g12510 Glyma17g01720 Glyma03g38990 Glyma17g00600

Glyma07g15800 Glyma18g01860 Glyma18g05340 Glyma08g14550 Glyma13g22940

45


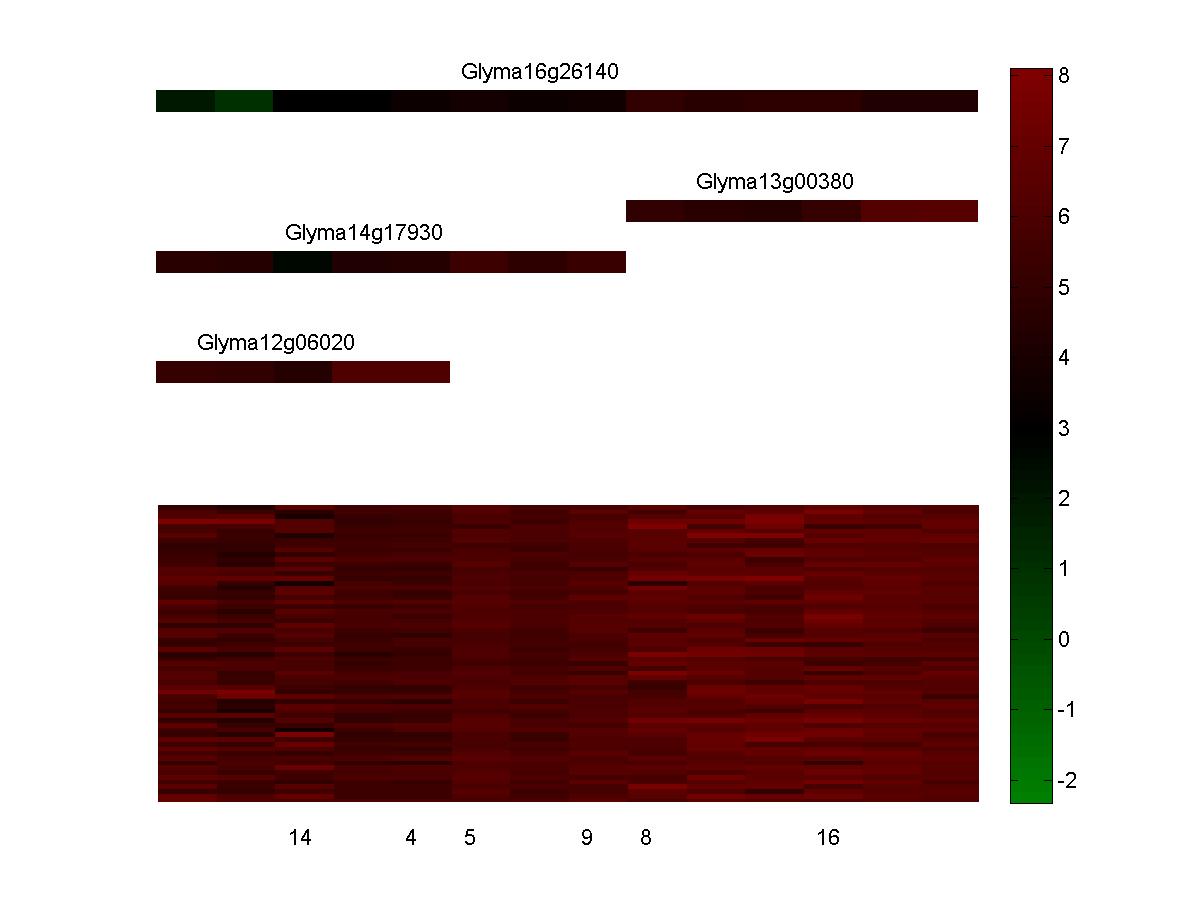


45 Glyma14g17930 CCHC (Zn)

45 Glyma13g00380 WRKY

45 Glyma12g06020 zf-A20

45 Glyma16g26140 DHHC (Zn)

Glyma11g02190 Glyma09g01800 Glyma04g07300 Glyma05g32220 Glyma13g40930

Glyma09g29960 Glyma13g04050 Glyma13g04440 Glyma05g01770 Glyma08g15480

Glyma08g22410 Glyma18g38010 Glyma05g36590 Glyma11g19490 Glyma14g08960

Glyma07g34900 Glyma19g36580 Glyma12g19050 Glyma02g29430 Glyma05g36140

Glyma11g08020 Glyma07g00540 Glyma08g23970 Glyma17g35890 Glyma17g00710

Glyma18g47730 Glyma06g04660 Glyma08g46240 Glyma20g20010 Glyma09g39880

Glyma08g01700 Glyma20g39340 Glyma18g06560 Glyma17g04830 Glyma07g10170

Glyma20g34330 Glyma15g42940 Glyma18g47420 Glyma16g06100 Glyma13g29180

Glyma16g08200 Glyma12g02870 Glyma03g30070 Glyma08g23990 Glyma15g08150

Glyma18g00480 Glyma18g46330 Glyma19g23570 Glyma20g39380 Glyma08g05710

Glyma17g34850 Glyma02g33770 Glyma07g10330 Glyma06g09500 Glyma13g29020

Glyma07g14540 Glyma02g44070 Glyma10g01960 Glyma06g12090 Glyma20g03020

Glyma19g35780 Glyma19g22730 Glyma08g39390

46


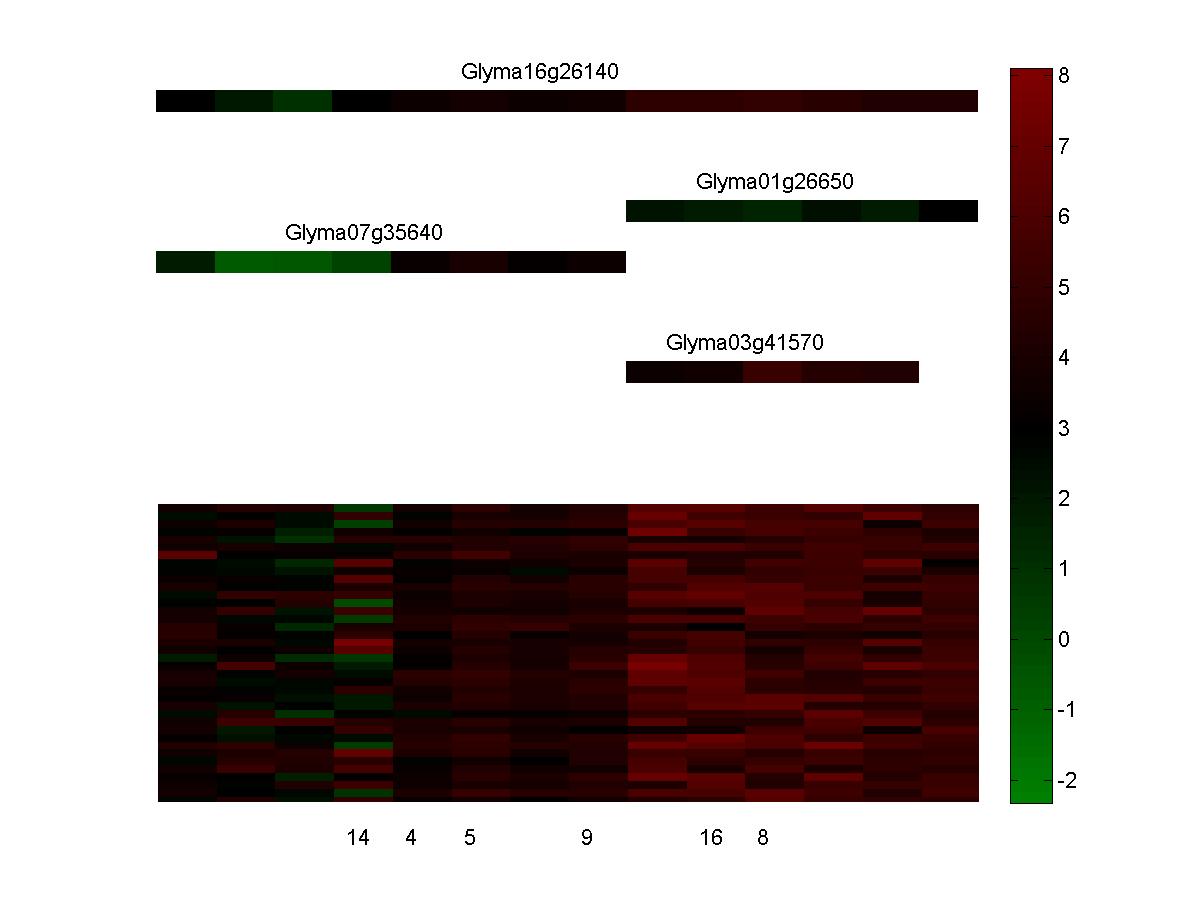


46 Glyma16g26140 DHHC (Zn)

46 Glyma03g41570 BTB/POZ

46 Glyma01g26650 MYB/HD-like

46 Glyma07g35640 TPR

Glyma06g18950 Glyma14g36900 Glyma17g13740 Glyma01g06640 Glyma18g43460

Glyma06g08390 Glyma14g05300 Glyma09g36740 Glyma03g13780 Glyma14g02350

Glyma18g07330 Glyma01g38980 Glyma13g08050 Glyma13g43050 Glyma11g13580

Glyma10g02190 Glyma14g09170 Glyma16g26070 Glyma13g26960 Glyma15g20180

Glyma07g00550 Glyma20g05530 Glyma10g36690 Glyma18g20800 Glyma13g33830

Glyma12g07260 Glyma11g25650 Glyma15g43180 Glyma02g04760 Glyma16g04730

Glyma13g04590 Glyma10g15250 Glyma17g35290 Glyma08g31890 Glyma01g39370

Glyma08g01410 Glyma20g33060 Glyma08g45820

47


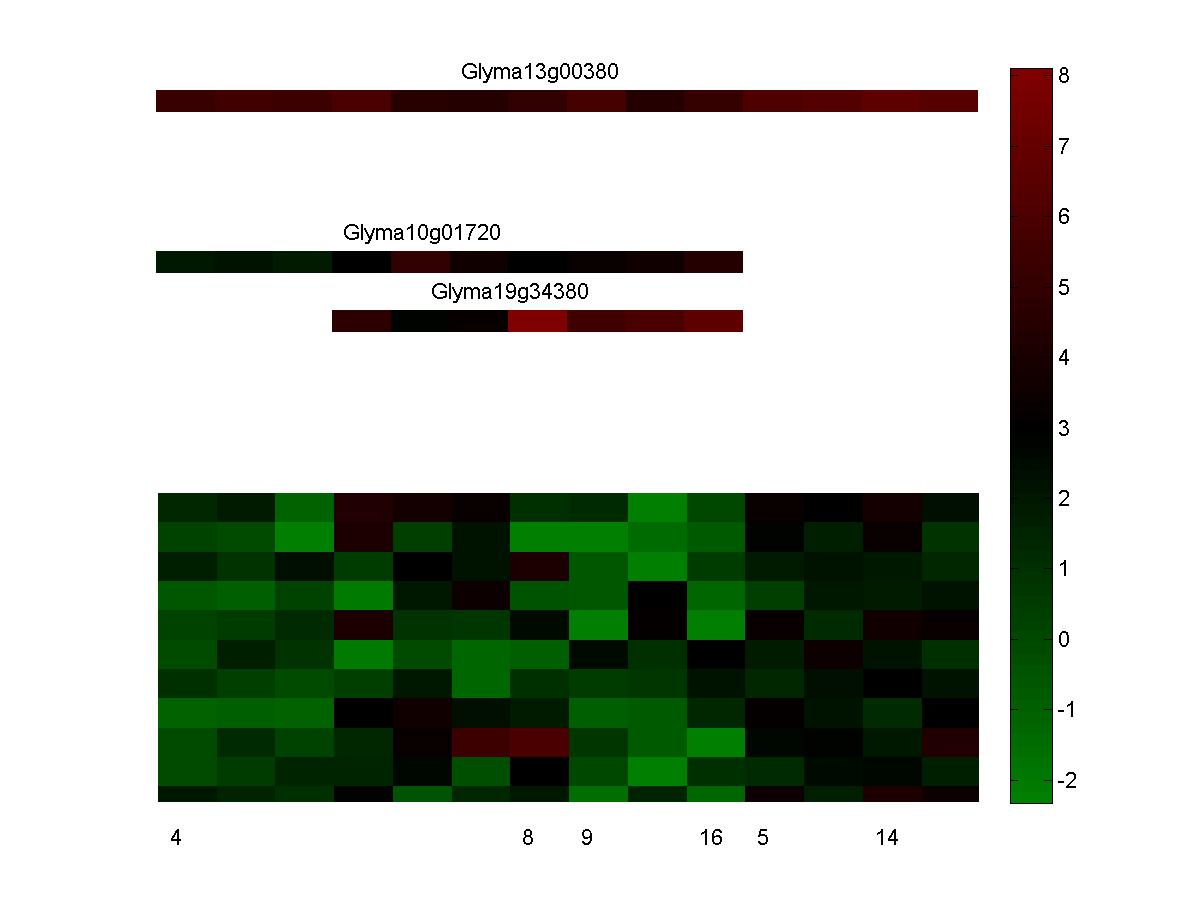


47 Glyma10g01720 TPR

47 Glyma13g00380 WRKY

47 Glyma19g34380 AUX-IAA-ARF

Glyma08g48030 Glyma04g42120 Glyma09g21820 Glyma02g36080 Glyma02g07980

Glyma17g06220 Glyma13g43010 Glyma12g02590 Glyma10g07500 Glyma13g23450

Glyma08g23310

48


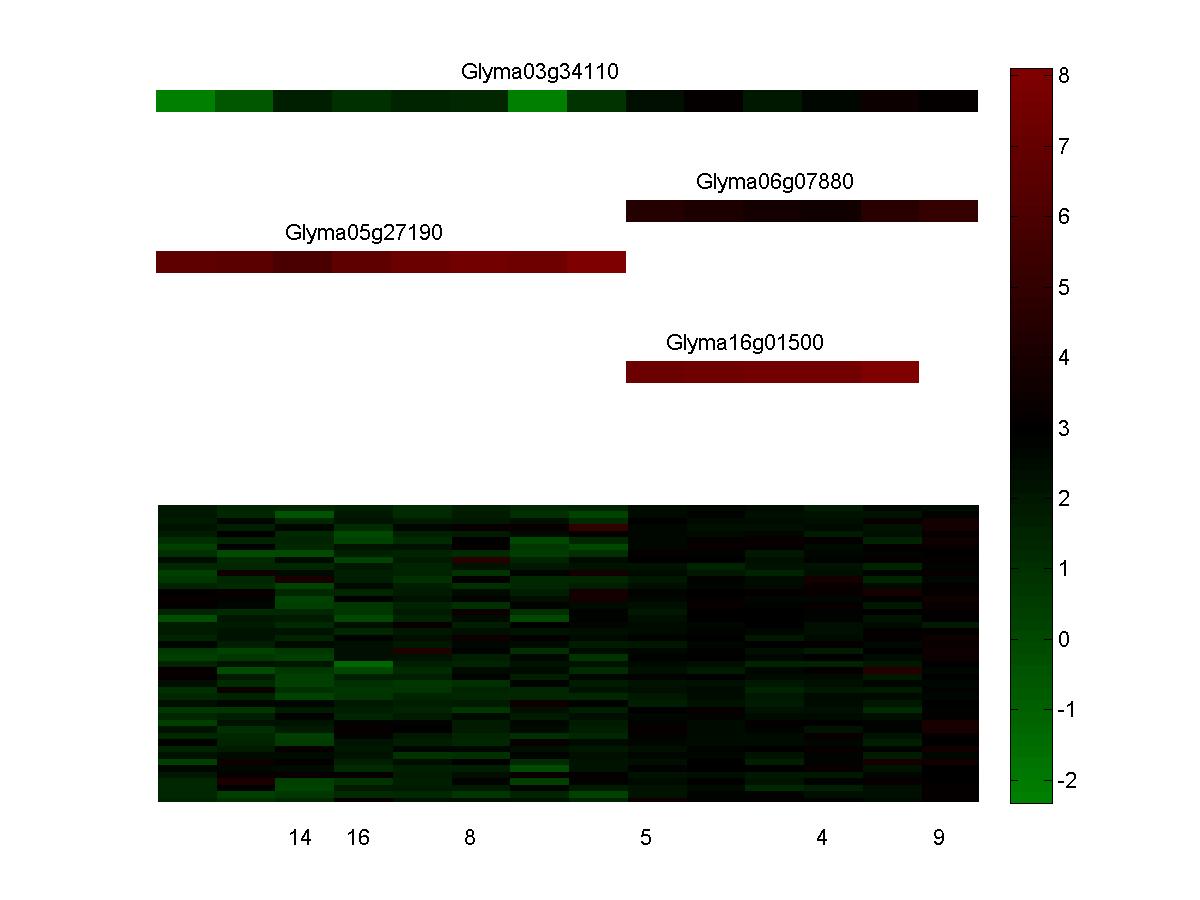


48 Glyma05g27190 GRAS

48 Glyma03g34110 MYB/HD-like

48 Glyma16g01500 AP2-EREBP

48 Glyma06g07880 HTH-ARAC

Glyma06g04510 Glyma08g12570 Glyma18g53370 Glyma06g19590 Glyma18g03550

Glyma08g04710 Glyma06g19630 Glyma03g41740 Glyma18g18210 Glyma03g35640

Glyma13g31870 Glyma05g27360 Glyma18g49540 Glyma16g27060 Glyma20g36200

Glyma18g08200 Glyma10g41230 Glyma12g32810 Glyma11g37100 Glyma20g24860

Glyma07g15420 Glyma15g11810 Glyma12g03740 Glyma06g21350 Glyma07g26040

Glyma20g02800 Glyma18g19360 Glyma13g28900 Glyma18g48060 Glyma01g04630

Glyma01g41530 Glyma14g21160 Glyma05g04530 Glyma09g32740 Glyma10g07930

Glyma01g01170 Glyma03g13310 Glyma09g41340 Glyma08g43130 Glyma04g38520

Glyma09g25200 Glyma04g04220 Glyma16g32480 Glyma15g03720 Glyma18g48180

Glyma06g05560

49


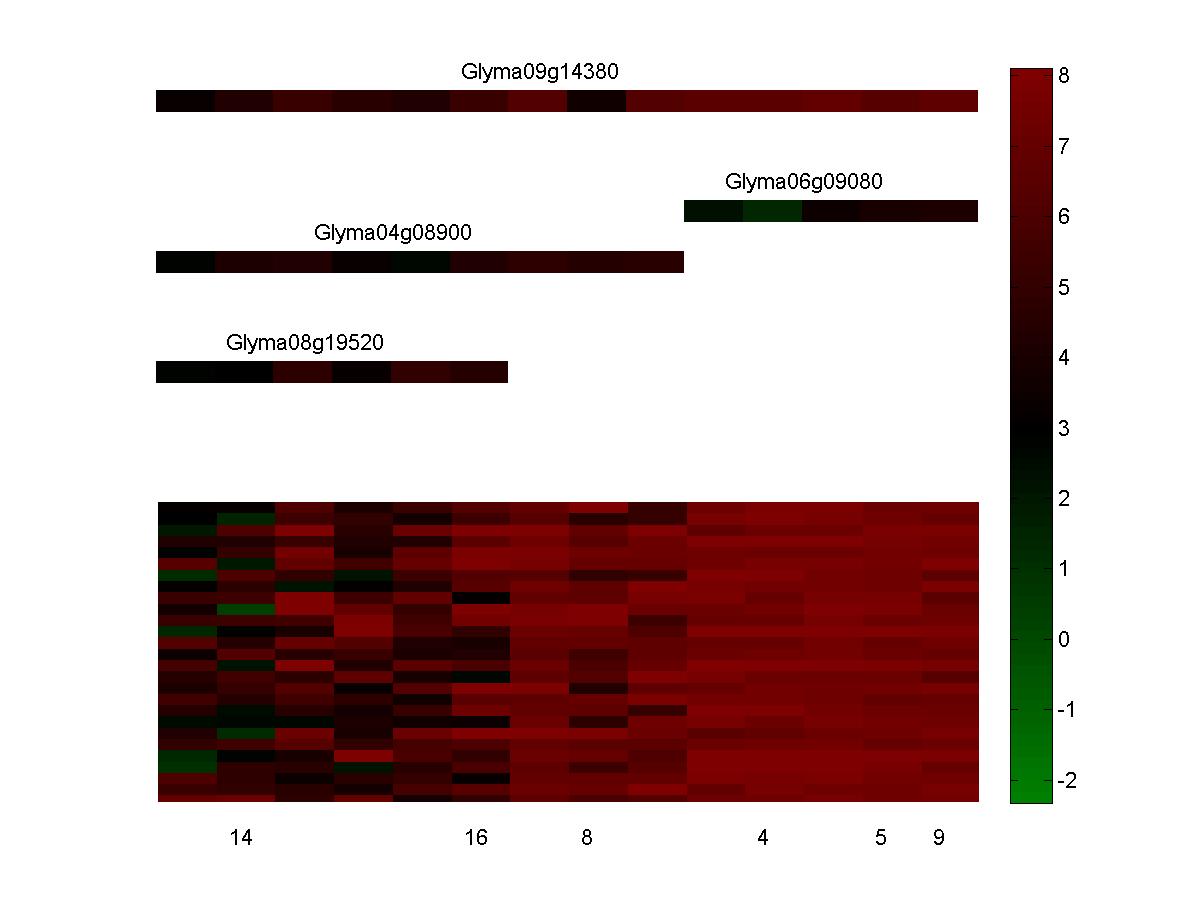


49 Glyma06g09080 C2C2 (Zn) GATA

49 Glyma09g14380 bHLH

49 Glyma08g19520 TUB

49 Glyma04g08900 AP2-EREBP

Glyma13g34530 Glyma06g02650 Glyma04g34840 Glyma07g16910 Glyma08g00790

Glyma18g49400 Glyma20g29840 Glyma01g24950 Glyma19g00550 Glyma18g02220

Glyma19g02370 Glyma03g30110 Glyma13g33590 Glyma11g36620 Glyma13g44950

Glyma02g11640 Glyma05g33190 Glyma13g34520 Glyma13g01870 Glyma08g06470

Glyma18g00590 Glyma17g10490 Glyma15g31750 Glyma01g42440 Glyma04g09350

Glyma13g19500 Glyma09g06250

50


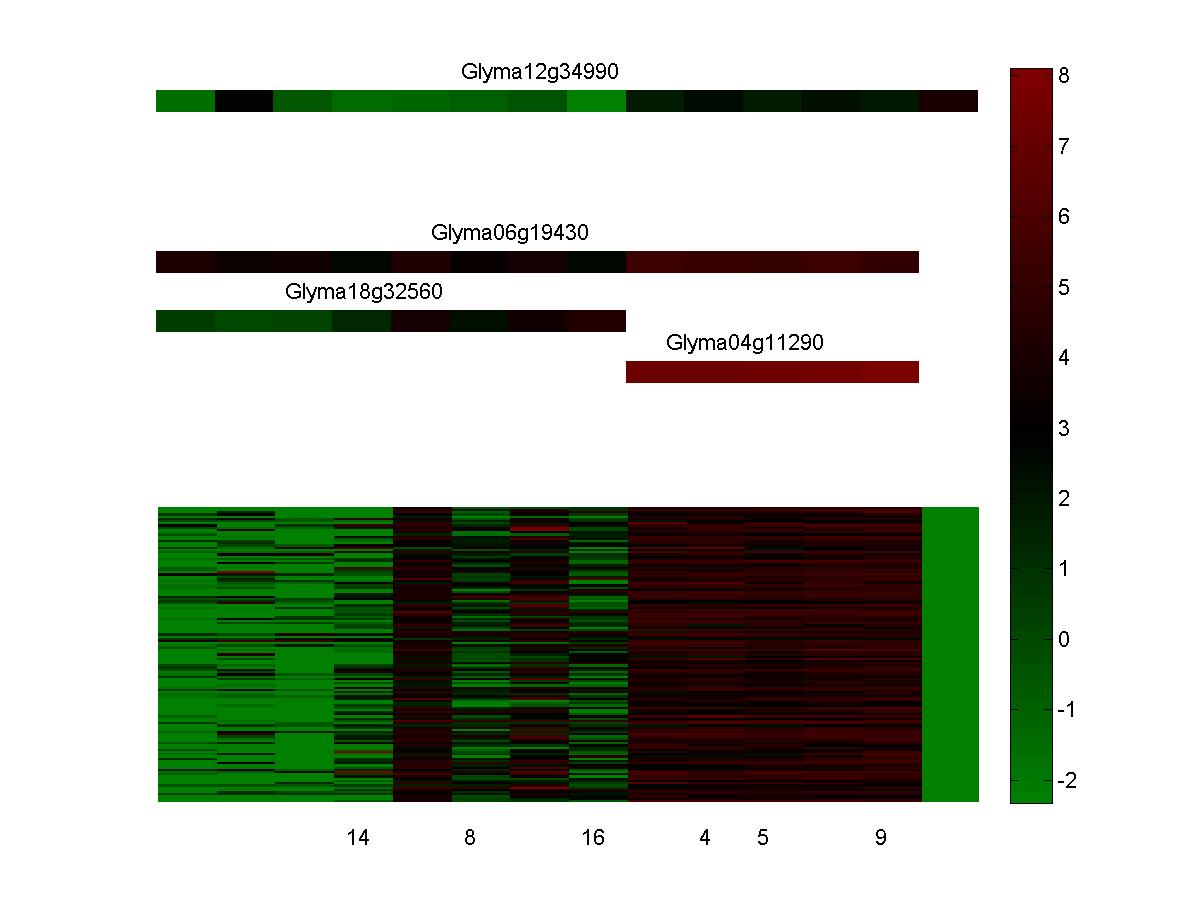


50 Glyma04g11290 AP2-EREBP

50 Glyma06g19430 TPR

50 Glyma12g34990 NAC

50 Glyma18g32560 bHLH

Glyma15g13100 Glyma04g37040 Glyma16g04980 Glyma15g07710 Glyma03g37390

Glyma09g41570 Glyma03g29190 Glyma05g04080 Glyma18g50870 Glyma10g32010

Glyma11g02640 Glyma17g01470 Glyma12g30990 Glyma19g30830 Glyma12g05790

Glyma01g28510 Glyma05g33010 Glyma02g05300 Glyma16g03710 Glyma19g28520

Glyma10g31280 Glyma03g30260 Glyma11g31330 Glyma09g02650 Glyma01g34580

Glyma06g03390 Glyma09g41850 Glyma08g25520 Glyma14g08810 Glyma18g39500

Glyma06g40620 Glyma13g05530 Glyma08g20220 Glyma14g16060 Glyma12g35370

Glyma09g28800 Glyma10g08010 Glyma06g17040 Glyma08g19180 Glyma06g12920

Glyma08g09990 Glyma19g33180 Glyma07g16810 Glyma11g13970 Glyma16g26970

Glyma07g17170 Glyma20g00590 Glyma03g03830 Glyma06g46800 Glyma08g19240

Glyma03g34110 Glyma08g44780 Glyma15g02000 Glyma05g25460 Glyma08g12470

Glyma01g42230 Glyma13g44140 Glyma07g11710 Glyma02g02880 Glyma16g31280

Glyma09g21280 Glyma05g05420 Glyma20g01370 Glyma14g39210 Glyma15g42760

Glyma01g20980 Glyma02g09210 Glyma03g24060 Glyma16g04720 Glyma10g08120

Glyma05g29380 Glyma13g23760 Glyma02g02860 Glyma02g35210 Glyma02g43580

Glyma02g09840 Glyma13g22350 Glyma08g46620 Glyma18g52950 Glyma17g16660

Glyma07g35240 Glyma15g11140 Glyma02g03190 Glyma06g08160 Glyma03g01190

Glyma05g37420 Glyma11g06690 Glyma07g05920 Glyma14g01660 Glyma05g03720

Glyma10g29130 Glyma06g06930 Glyma16g22920 Glyma01g43380 Glyma08g18340

Glyma06g28650 Glyma08g12290 Glyma19g28950 Glyma10g40350 Glyma16g22620

Glyma19g37760 Glyma12g05320 Glyma09g06350 Glyma10g27020 Glyma02g17060

Glyma19g40770 Glyma01g40820 Glyma12g34880 Glyma06g44450 Glyma13g01900

Glyma08g05000 Glyma13g30330 Glyma04g05510 Glyma12g10850 Glyma19g40000

Glyma09g27370 Glyma11g27480 Glyma13g25570 Glyma11g09250 Glyma15g01170

Glyma09g16930 Glyma16g06900 Glyma09g05280 Glyma13g43140 Glyma02g37280

Glyma13g31610 Glyma16g03570 Glyma06g11600 Glyma09g31840 Glyma06g17950

Glyma15g35410 Glyma12g00980 Glyma07g11720

51


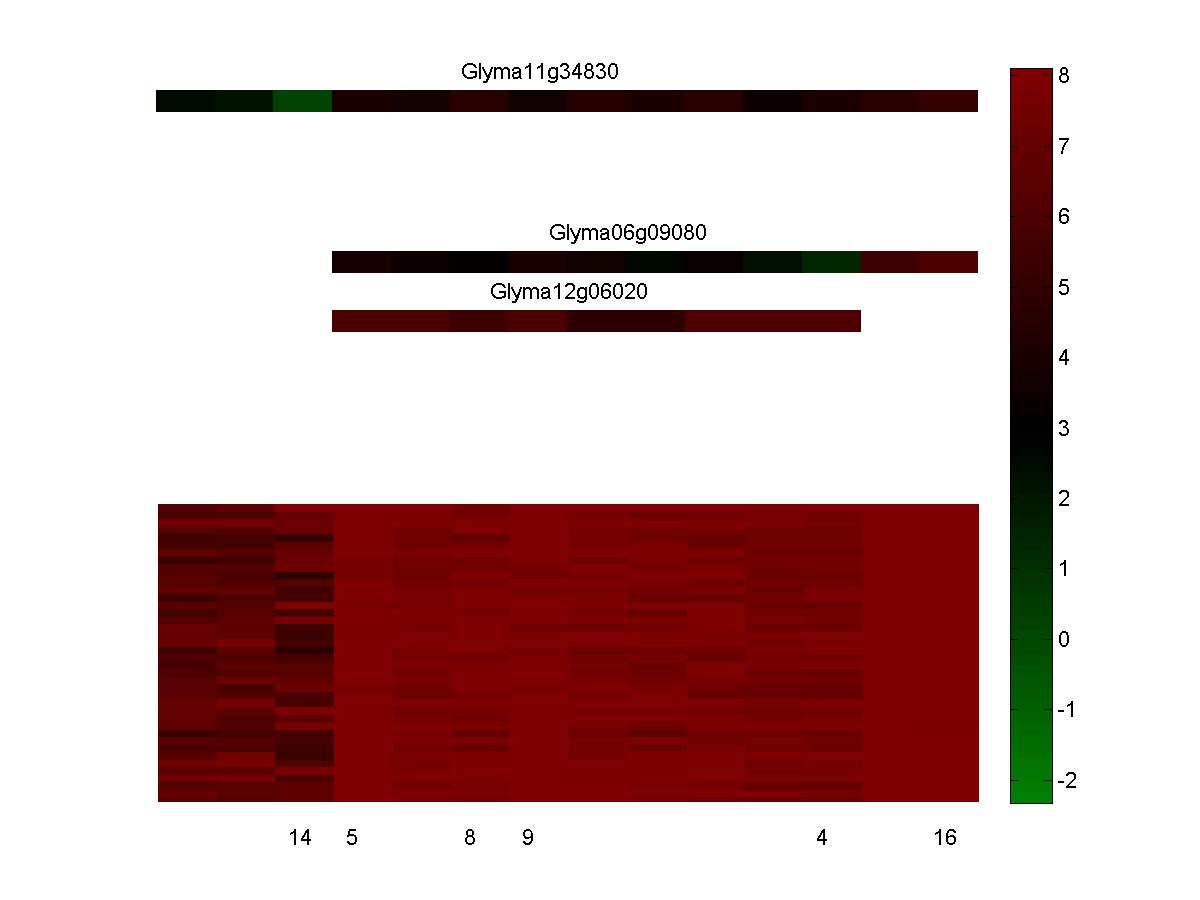


51 Glyma06g09080 C2C2 (Zn) GATA

51 Glyma11g34830 LIM

51 Glyma12g06020 zf-A20

Glyma19g03960 Glyma02g05540 Glyma13g06390 Glyma15g10220 Glyma06g20540

Glyma10g28890 Glyma03g35540 Glyma08g11490 Glyma13g20780 Glyma13g19470

Glyma04g36140 Glyma04g33900 Glyma08g05290 Glyma10g04560 Glyma13g18830

Glyma20g24280 Glyma02g11540 Glyma15g40860 Glyma18g08220 Glyma11g10480

Glyma05g34070 Glyma06g02330 Glyma02g09370 Glyma13g19930 Glyma17g16830

Glyma09g02240 Glyma05g01180 Glyma05g34350 Glyma16g24120 Glyma04g40470

Glyma14g06630 Glyma20g23080 Glyma01g37250 Glyma19g03950 Glyma03g40280

Glyma13g19330 Glyma09g02160 Glyma04g11400 Glyma09g24070 Glyma10g36780

52


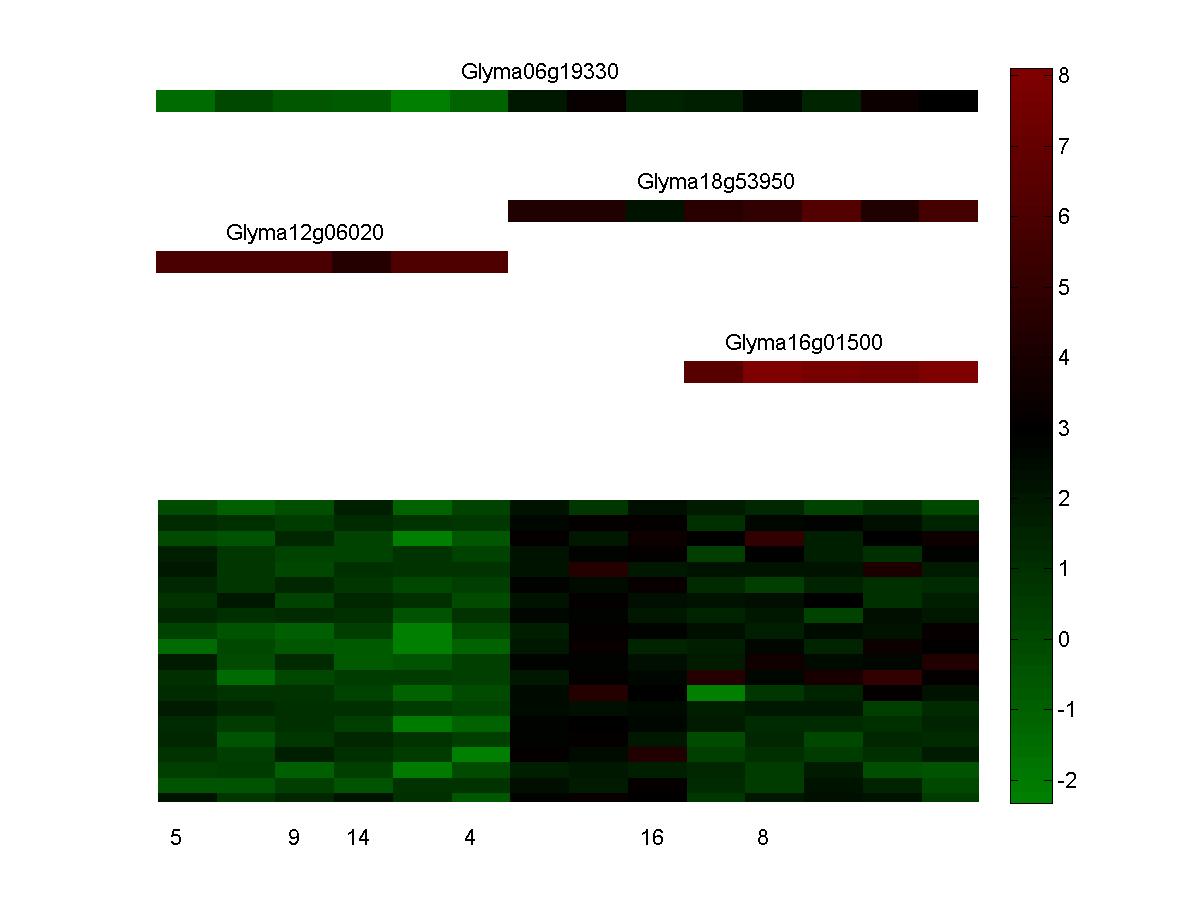


52 Glyma18g53950 NAC

52 Glyma12g06020 zf-A20

52 Glyma16g01500 AP2-EREBP

52 Glyma06g19330 C2C2 (Zn) Dof

Glyma10g00580 Glyma12g29320 Glyma10g01730 Glyma19g32590 Glyma19g40800

Glyma15g23470 Glyma04g06040 Glyma06g19330 Glyma07g07810 Glyma01g23430

Glyma06g05130 Glyma17g14670 Glyma19g27800 Glyma17g01590 Glyma13g41030

Glyma15g22220 Glyma11g02500 Glyma13g04610 Glyma05g28110 Glyma11g30730

53


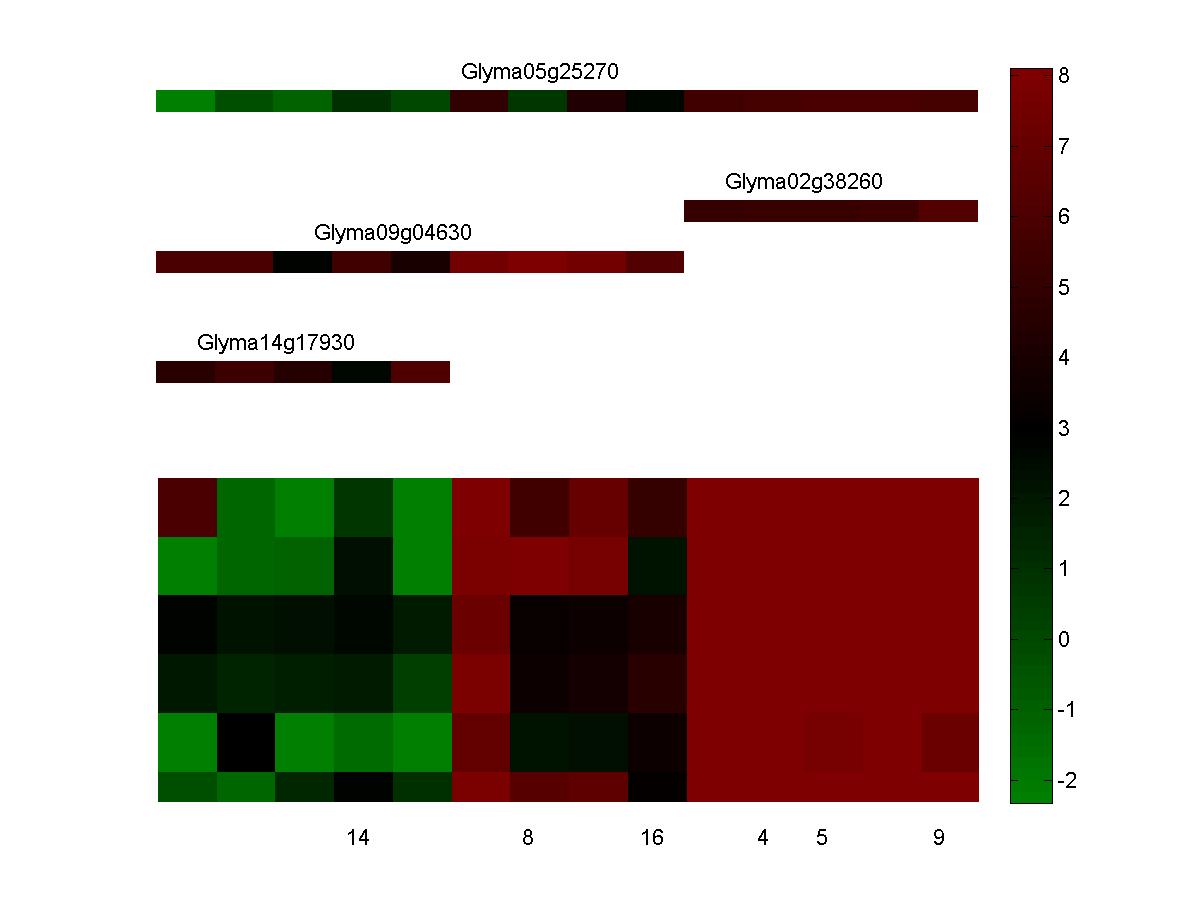


53 Glyma05g25270 WRKY

53 Glyma09g04630 AP2-EREBP

53 Glyma02g38260 AUX-IAA-ARF

53 Glyma14g17930 CCHC (Zn)

Glyma15g07040 Glyma19g37230 Glyma17g08900 Glyma15g31520 Glyma19g37240

Glyma10g05800

54


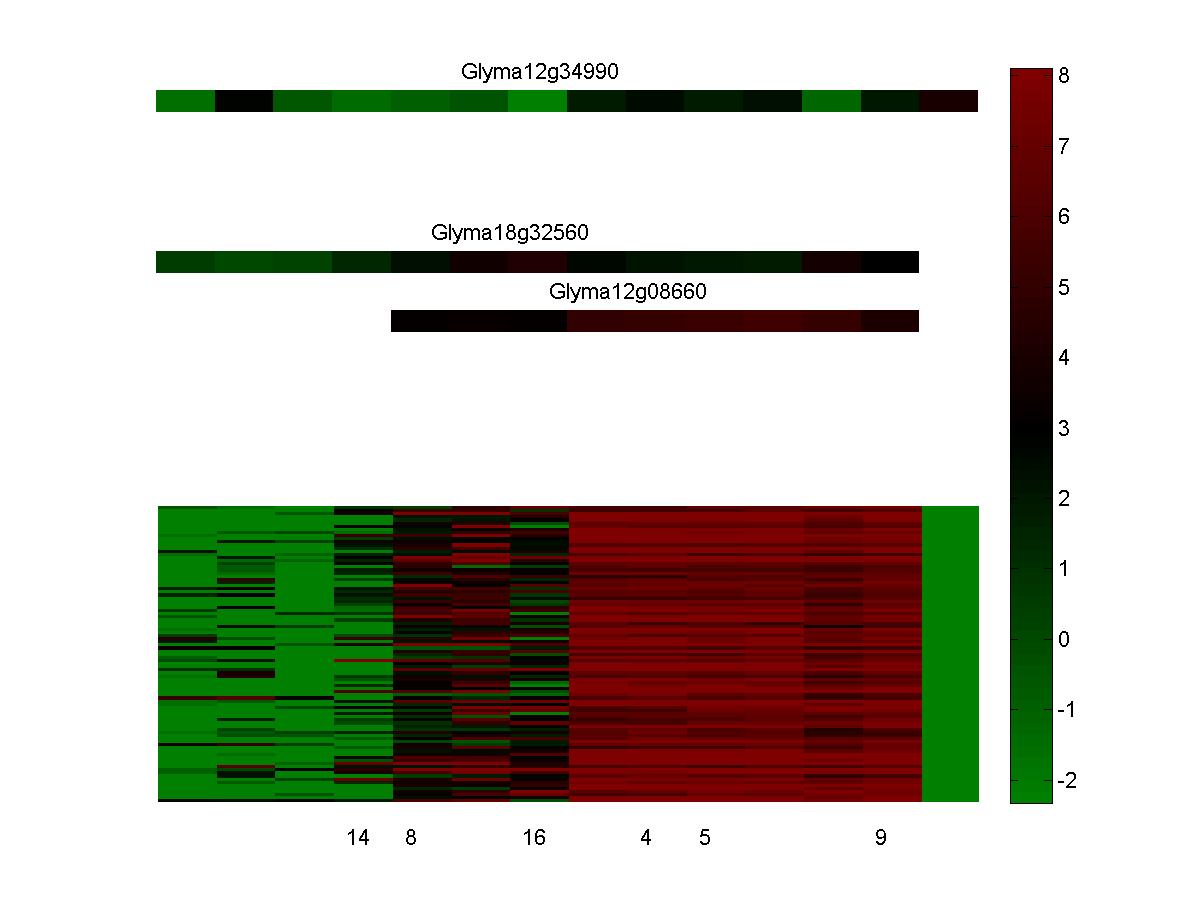


54 Glyma12g34990 NAC

54 Glyma18g32560 bHLH

54 Glyma12g08660 C2H2 (Zn)

Glyma16g05710 Glyma18g12180 Glyma12g09940 Glyma12g02040 Glyma03g37400

Glyma15g16710 Glyma15g05760 Glyma10g02090 Glyma15g13870 Glyma15g07700

Glyma08g04370 Glyma01g33450 Glyma09g21010 Glyma04g35130 Glyma03g37410

Glyma12g06300 Glyma01g42370 Glyma12g03050 Glyma09g23140 Glyma12g07350

Glyma03g29950 Glyma11g20700 Glyma18g06220 Glyma13g42140 Glyma01g31660

Glyma20g11610 Glyma16g33790 Glyma15g25060 Glyma12g05770 Glyma11g06070

Glyma08g24720 Glyma11g10760 Glyma18g53740 Glyma15g13550 Glyma12g00970

Glyma05g25450 Glyma11g12650 Glyma10g03000 Glyma11g35560 Glyma17g15690

Glyma17g03360 Glyma13g23680 Glyma11g35030 Glyma13g25560 Glyma10g00950

Glyma10g02730 Glyma08g27070 Glyma15g14210 Glyma07g37270 Glyma08g24760

Glyma09g31110 Glyma06g47190 Glyma02g16800 Glyma19g45260 Glyma18g02870

Glyma12g00720 Glyma08g24770 Glyma06g12010 Glyma20g38590 Glyma09g00850

Glyma02g40010 Glyma05g30290 Glyma15g35390 Glyma16g28610 Glyma03g15800

Glyma15g02380 Glyma17g12160 Glyma11g03310 Glyma11g29920 Glyma06g01270

Glyma08g43040 Glyma01g33440 Glyma11g14120 Glyma03g34560 Glyma02g01970

Glyma12g02240 Glyma08g43550 Glyma09g32630 Glyma15g06780 Glyma09g37460

Glyma03g04880 Glyma06g18560 Glyma19g40960 Glyma09g02610 Glyma06g12670

Glyma01g37820 Glyma10g29150 Glyma07g04340 Glyma01g34770 Glyma03g03460

Glyma15g15200 Glyma06g38530 Glyma03g36620 Glyma04g02230 Glyma09g09430

55


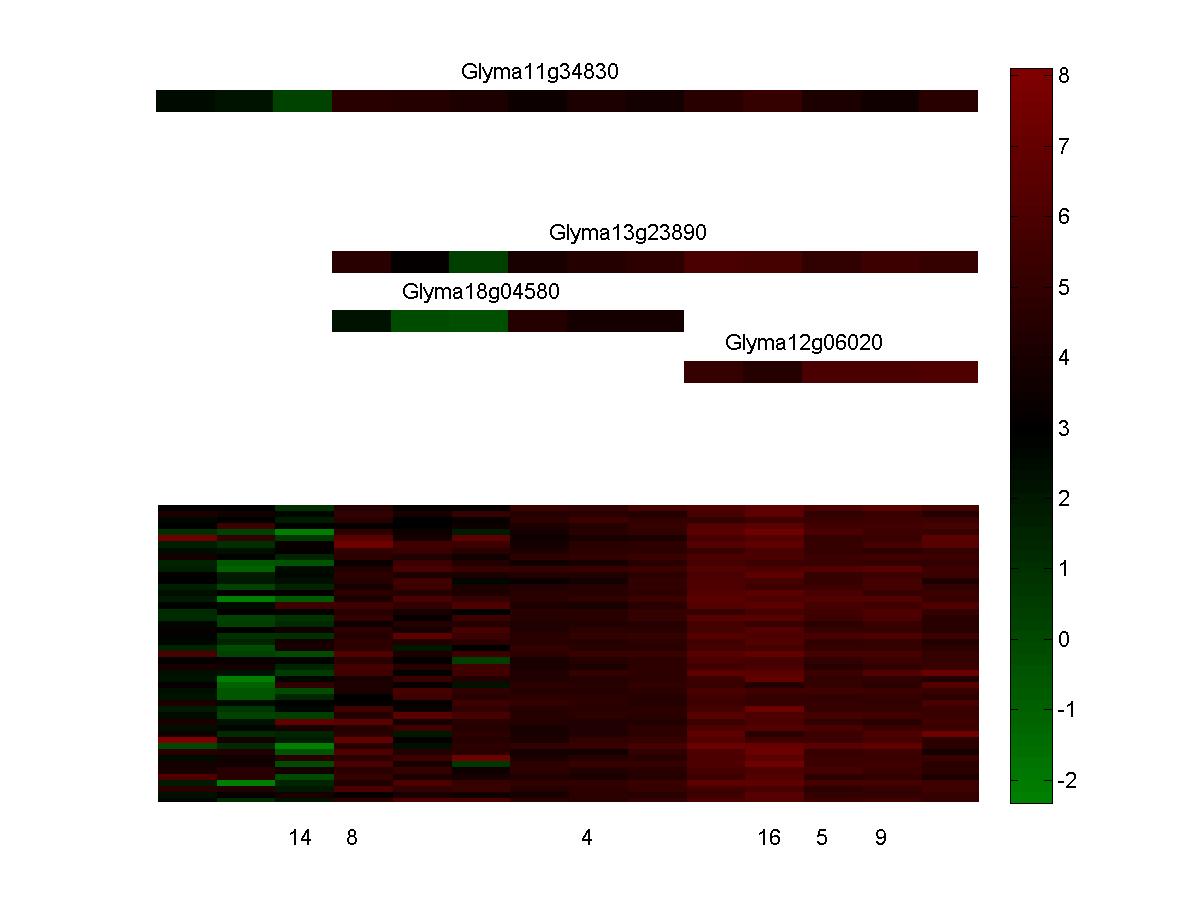


55 Glyma18g04580 MYB/HD-like

55 Glyma12g06020 zf-A20

55 Glyma11g34830 LIM

55 Glyma13g23890 Homeodomain/HOMEOBOX

Glyma20g04990 Glyma16g09770 Glyma13g23890 Glyma11g00210 Glyma11g15500

Glyma20g29990 Glyma05g36320 Glyma10g03620 Glyma08g24380 Glyma03g42070

Glyma10g05720 Glyma04g33570 Glyma02g05480 Glyma07g02400 Glyma18g07150

Glyma08g26880 Glyma15g15370 Glyma15g18220 Glyma15g03430 Glyma10g41450

Glyma19g28750 Glyma20g31120 Glyma18g03950 Glyma13g01950 Glyma19g28220

Glyma01g27810 Glyma12g05910 Glyma16g05450 Glyma16g29340 Glyma12g32160

Glyma15g15990 Glyma13g34410 Glyma03g30440 Glyma18g53440 Glyma11g02530

Glyma03g35080 Glyma06g05320 Glyma08g27590 Glyma02g0906 Glyma13g24180

Glyma17g14910 Glyma18g12660 Glyma08g46110 Glyma03g09100 Glyma17g02000

Glyma11g24400 Glyma14g39880 Glyma17g38120 Glyma18g08530

56


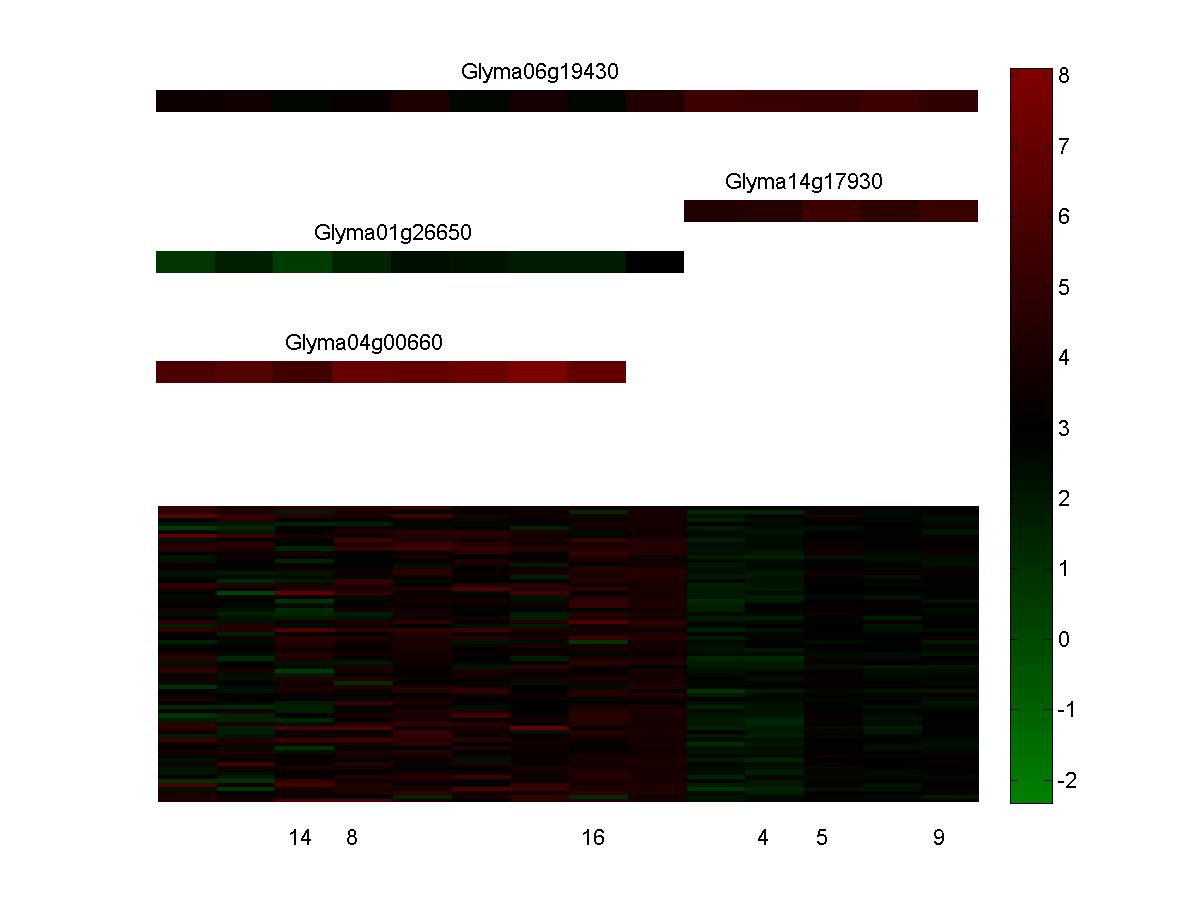


56 Glyma14g17930 CCHC (Zn)

56 Glyma01g26650 MYB/HD-like

56 Glyma04g00660 CSD

56 Glyma06g19430 TPR

Glyma10g01720 Glyma03g33000 Glyma13g00580 Glyma13g30930 Glyma02g14190

Glyma08g47960 Glyma10g02030 Glyma13g17290 Glyma13g42850 Glyma19g34350

Glyma03g35710 Glyma09g24910 Glyma18g45100 Glyma09g38750 Glyma07g31330

Glyma19g29410 Glyma08g07740 Glyma02g02950 Glyma02g12950 Glyma05g05710

Glyma06g19960 Glyma04g36400 Glyma11g15890 Glyma11g17490 Glyma15g18950

Glyma06g18730 Glyma19g30530 Glyma02g42150 Glyma03g41460 Glyma02g40270

Glyma10g41610 Glyma07g40160 Glyma11g13590 Glyma13g44910 Glyma06g19600

Glyma20g34460 Glyma09g37800 Glyma10g34480 Glyma08g13280 Glyma20g26960

Glyma13g32290 Glyma06g06170 Glyma04g22780 Glyma13g04960 Glyma18g47580

Glyma09g05590 Glyma07g38290 Glyma18g01910 Glyma18g49970 Glyma16g04090

Glyma07g35540 Glyma20g38810 Glyma17g36730 Glyma07g19190 Glyma15g11840

Glyma20g30430 Glyma05g14430 Glyma13g24810 Glyma05g24640 Glyma02g12460

Glyma09g03660 Glyma03g00880 Glyma09g03630 Glyma02g40790 Glyma04g04460

Glyma11g00600 Glyma17g14660 Glyma13g30240 Glyma13g03160 Glyma20g11950

Glyma15g16650 Glyma14g39120 Glyma11g35840

57


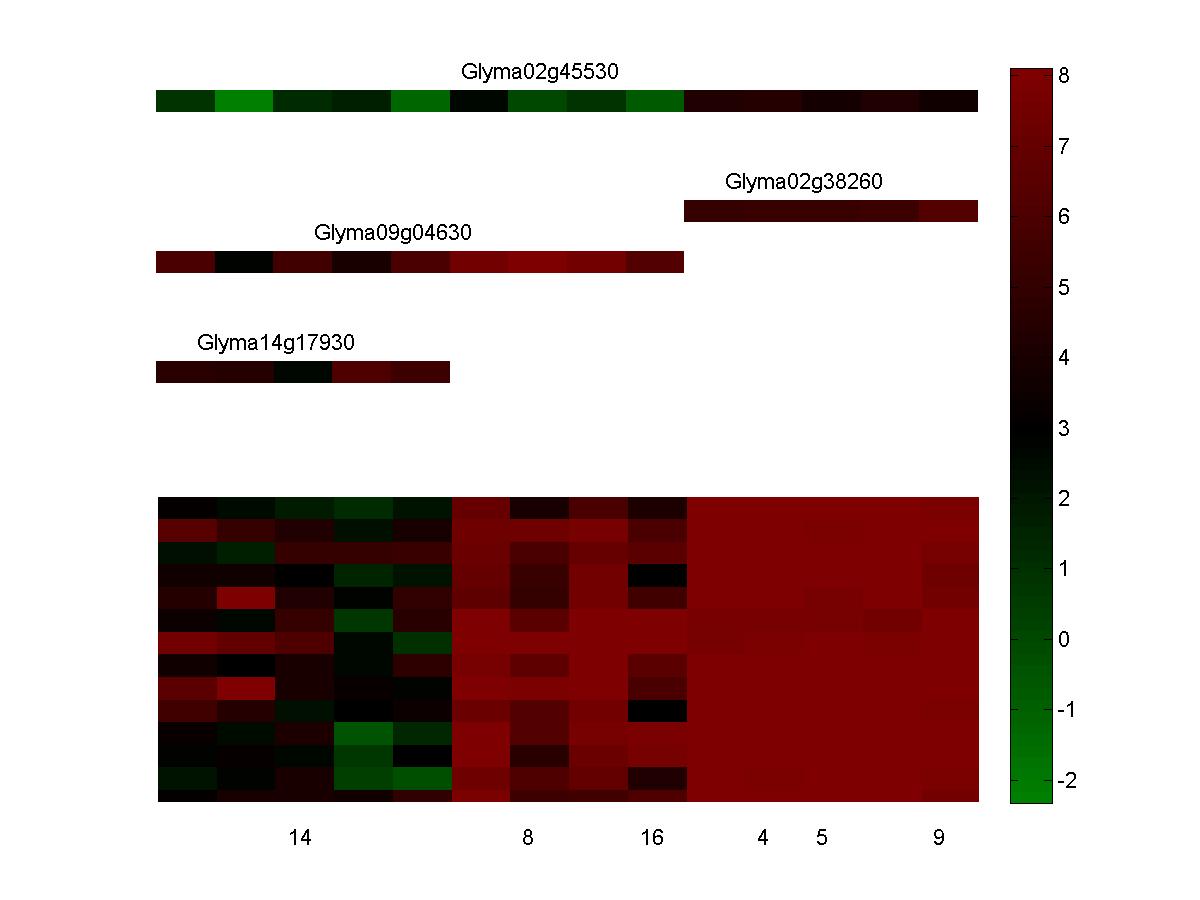


57 Glyma14g17930 CCHC (Zn)

57 Glyma02g38260 AUX-IAA-ARF

57 Glyma09g04630 AP2-EREBP

57 Glyma02g45530 WRKY

Glyma05g22180 Glyma10g29260 Glyma10g38070 Glyma11g07490 Glyma17g03340

Glyma01g43880 Glyma08g00780 Glyma14g00870 Glyma16g28590 Glyma17g03910

Glyma11g00230 Glyma09g28490 Glyma08g47750 Glyma10g44360

58


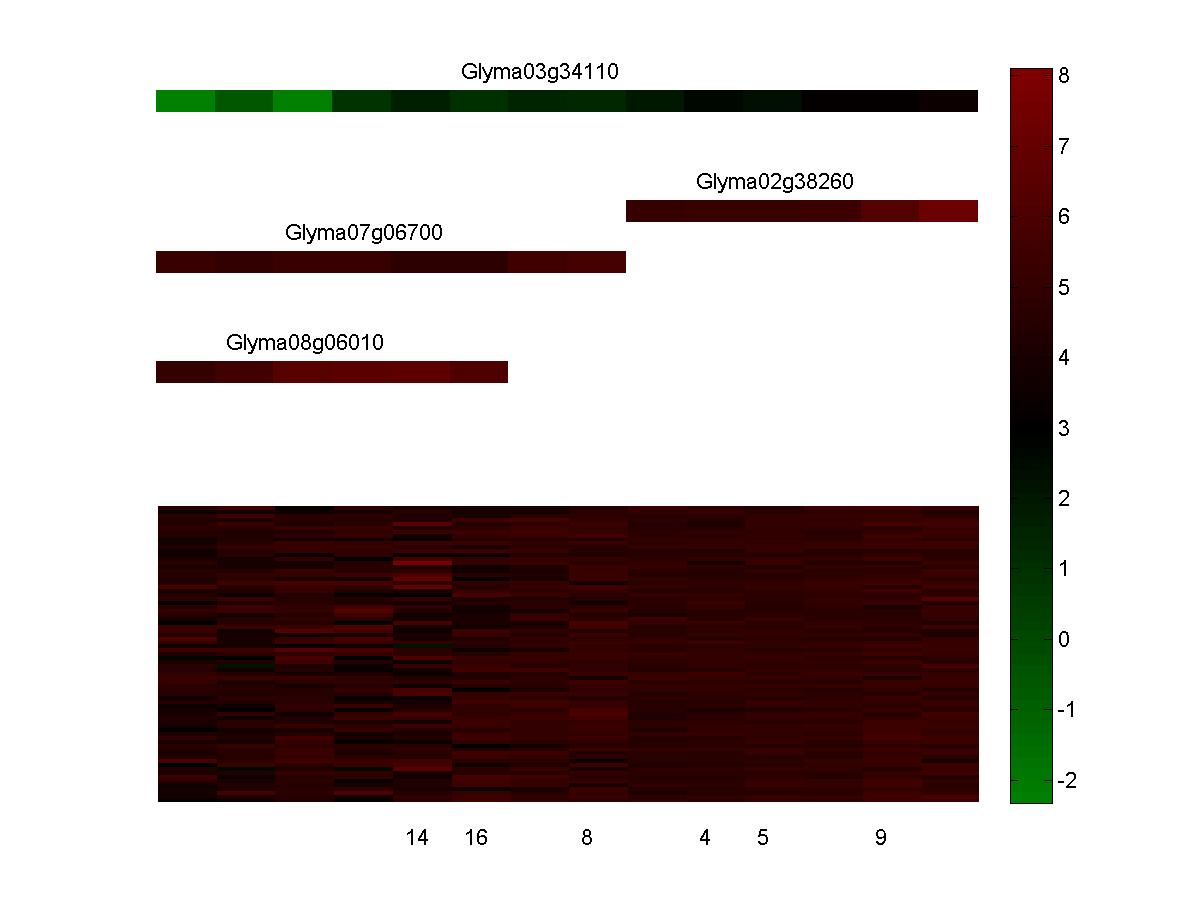


58 Glyma03g34110 MYB/HD-like

58 Glyma02g38260 AUX-IAA-ARF

58 Glyma07g06700 BTB/POZ

58 Glyma08g06010 TPR

Glyma10g07950 Glyma05g27570 Glyma06g45900 Glyma02g46050 Glyma20g24450

Glyma04g00770 Glyma15g13470 Glyma20g38640 Glyma16g05370 Glyma07g16120

Glyma11g37760 Glyma20g08680 Glyma03g06350 Glyma05g37590 Glyma18g53810

Glyma10g43110 Glyma10g09280 Glyma18g07500 Glyma15g13940 Glyma11g05350

Glyma07g06330 Glyma10g40250 Glyma13g39580 Glyma05g34960 Glyma18g54010

Glyma07g15170 Glyma05g33980 Glyma04g09950 Glyma17g05970 Glyma12g07250

Glyma02g46380 Glyma08g13990 Glyma11g14980 Glyma20g30660 Glyma10g44070

Glyma16g26600 Glyma03g24270 Glyma04g38640 Glyma07g30470 Glyma12g33540

Glyma08g11800 Glyma09g05180 Glyma14g23990 Glyma17g35610 Glyma06g15280

Glyma04g43160 Glyma06g04190 Glyma04g37760 Glyma17g05690 Glyma04g08730

Glyma06g01930 Glyma01g34820 Glyma13g04100 Glyma10g36980 Glyma09g02040

Glyma08g17810 Glyma07g34340 Glyma17g20610 Glyma08g46210 Glyma04g08200

Glyma08g46940 Glyma11g19780 Glyma04g41480 Glyma15g36180 Glyma11g31450

Glyma11g17030 Glyma16g33870 Glyma04g06010 Glyma10g43120 Glyma11g34960

Glyma08g23550 Glyma15g41680 Glyma10g35210 Glyma08g24460 Glyma17g05290

59


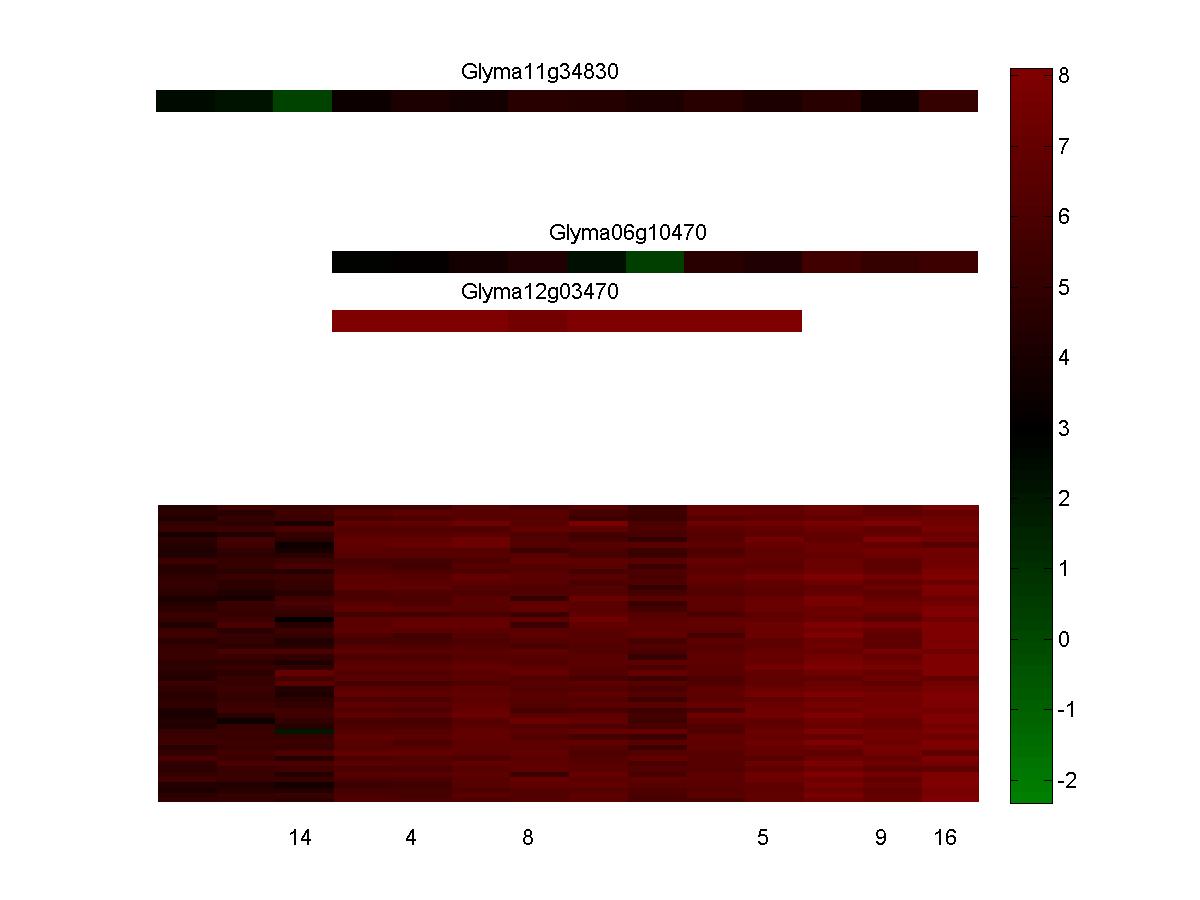


59 Glyma06g10470 bHLH

59 Glyma11g34830 LIM

59 Glyma12g03470 CSD

Glyma06g46530 Glyma01g41810 Glyma06g06790 Glyma19g37370 Glyma17g00820

Glyma06g07410 Glyma07g00700 Glyma02g43080 Glyma20g38130 Glyma07g00330

Glyma05g05670 Glyma15g01860 Glyma19g40080 Glyma12g08050 Glyma15g04810

Glyma16g17370 Glyma02g04090 Glyma11g05160 Glyma01g03570 Glyma08g23750

Glyma05g02570 Glyma11g15230 Glyma02g00540 Glyma03g40530 Glyma11g20570

Glyma03g37340 Glyma09g38550 Glyma10g30200 Glyma08g09230 Glyma08g17600

Glyma20g38970 Glyma03g42180 Glyma01g41620 Glyma12g04020 Glyma10g36610

Glyma09g05720 Glyma04g37120 Glyma07g06590 Glyma10g00890 Glyma04g43540

Glyma11g02410 Glyma08g18860 Glyma19g06460 Glyma08g41280 Glyma08g22880

Glyma14g38220 Glyma05g04670 Glyma13g43450 Glyma19g27380 Glyma20g21230

Glyma0169s00200 Glyma11g11020 Glyma19g28850 Glyma10g07850 Glyma08g21960

Glyma05g30780

60


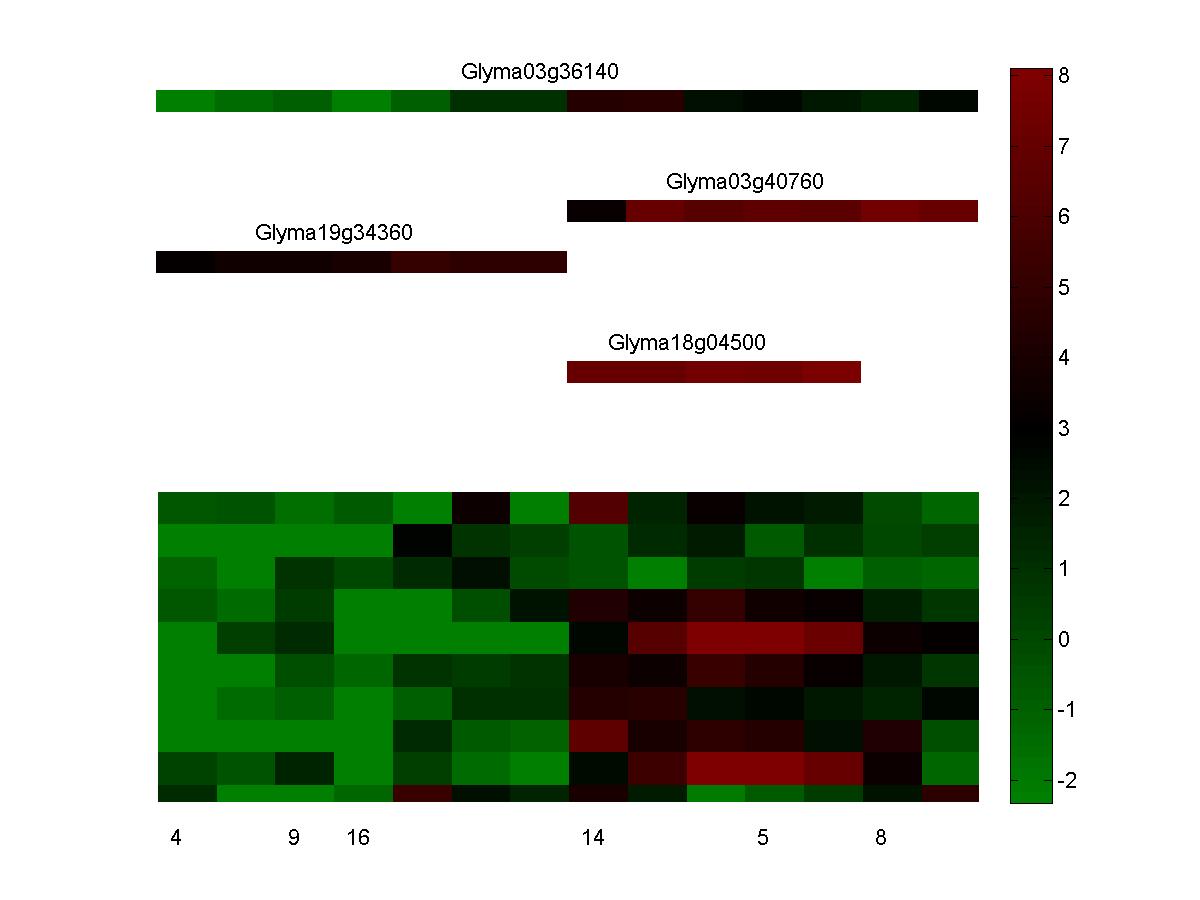


60 Glyma03g40760 AUX-IAA-ARF

60 Glyma19g34360 bHLH

60 Glyma18g04500 GRAS

60 Glyma03g36140 CCAAT

Glyma14g11320 Glyma03g36140 Glyma15g15010 Glyma01g03470 Glyma19g38800

Glyma02g15420 Glyma02g35190 Glyma13g38710 Glyma14g05840 Glyma02g42730

61


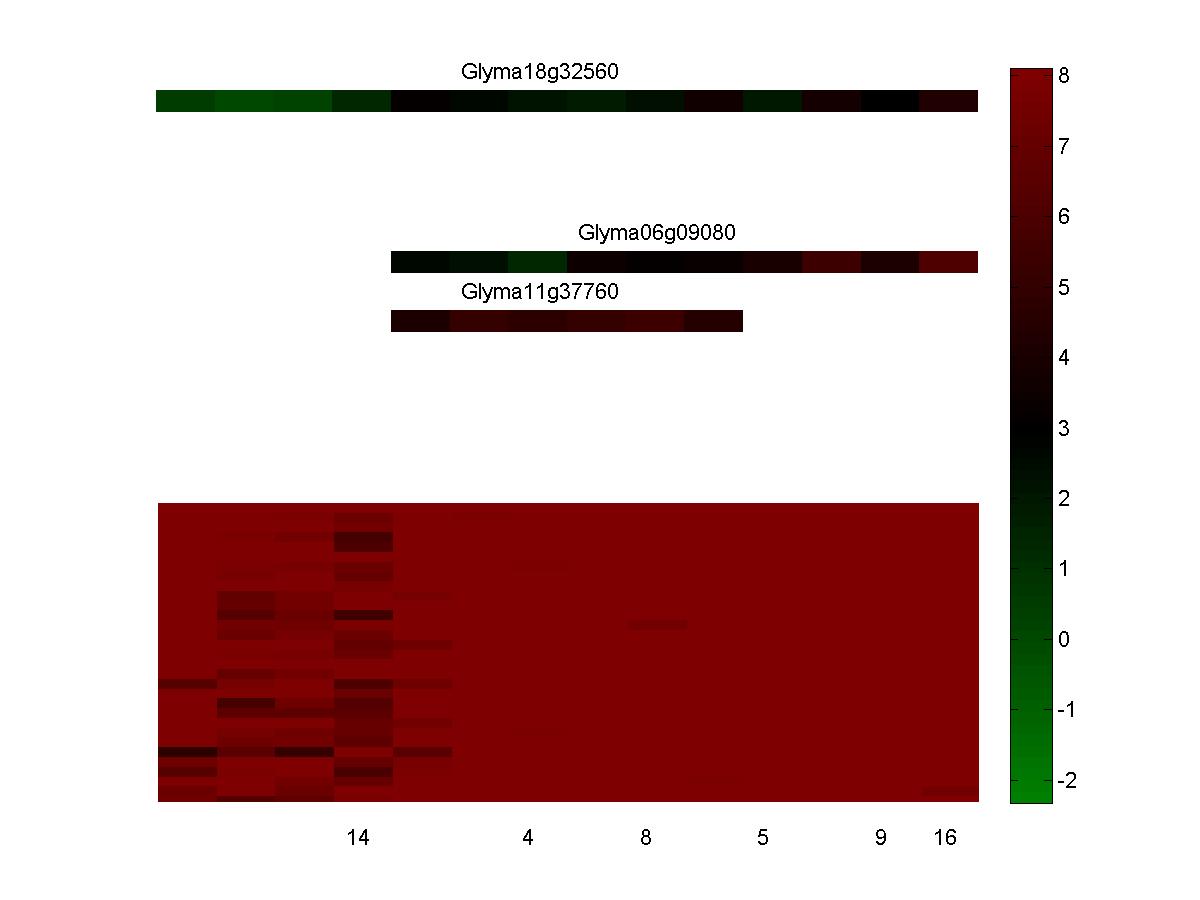


61 Glyma18g32560 bHLH

61 Glyma11g37760 MYB

61 Glyma06g09080 C2C2 (Zn) GATA

Glyma10g05580 Glyma08g46520 Glyma11g11300 Glyma05g23230 Glyma05g11630

Glyma08g11480 Glyma11g11290 Glyma07g34440 Glyma19g29180 Glyma12g07780

Glyma12g04710 Glyma12g03470 Glyma05g24110 Glyma14g09440 Glyma05g29000

Glyma02g04510 Glyma08g29090 Glyma19g35560 Glyma11g12470 Glyma12g04700

Glyma06g19820 Glyma19g35570 Glyma03g32850 Glyma17g23870 Glyma08g12140

Glyma08g18110 Glyma12g02790 Glyma01g03070 Glyma19g07240 Glyma07g15610

Glyma05g28480

62


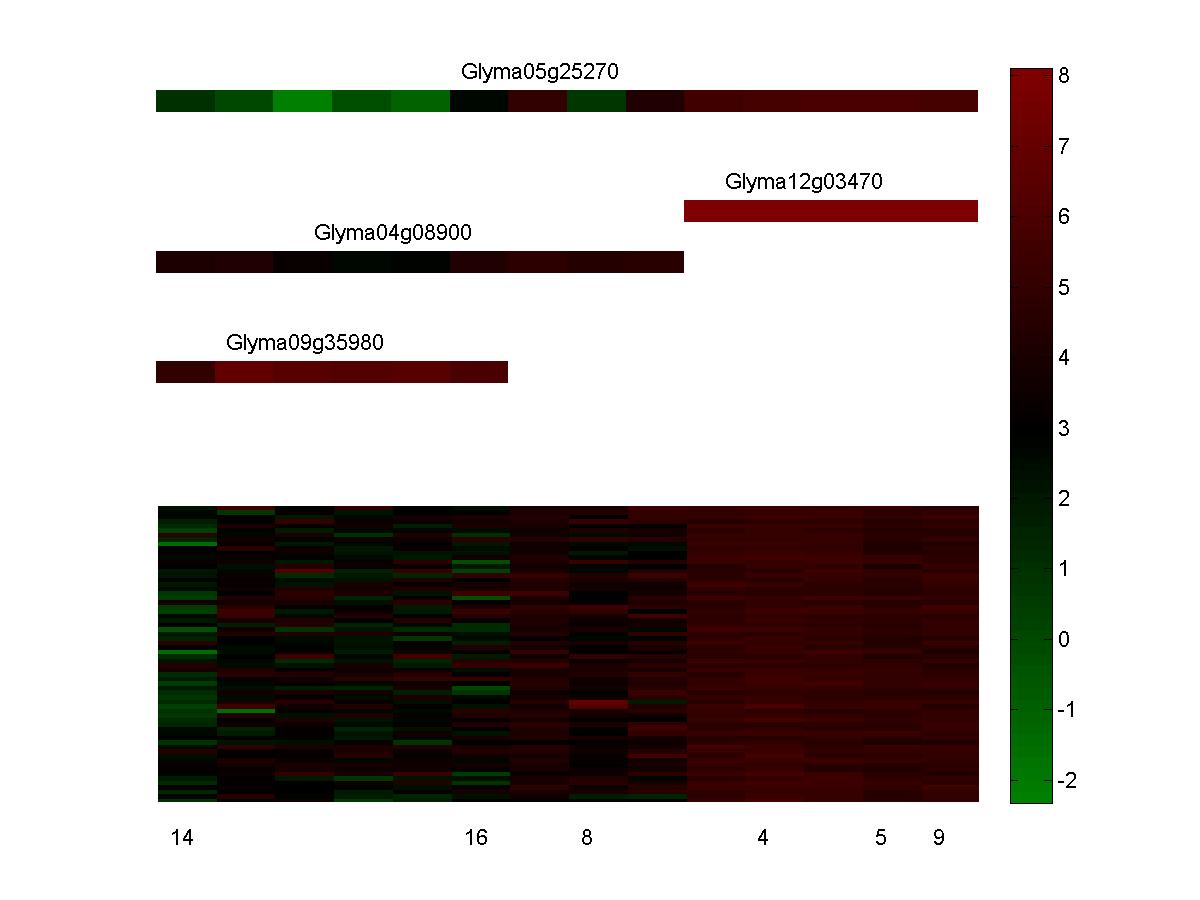


62 Glyma05g25270 WRKY

62 Glyma04g08900 AP2-EREBP

62 Glyma12g03470 CSD

62 Glyma09g35980 C3H-type1(Zn)

Glyma20g02490 Glyma04g35180 Glyma19g43520 Glyma05g00320 Glyma19g30360

Glyma15g08520 Glyma17g05860 Glyma14g04580 Glyma04g43340 Glyma11g30100

Glyma18g50770 Glyma01g45260 Glyma13g37280 Glyma03g13520 Glyma07g01270

Glyma13g05020 Glyma02g06160 Glyma12g35140 Glyma04g34020 Glyma11g33370

Glyma20g02500 Glyma06g19430 Glyma05g28560 Glyma11g20380 Glyma01g42200

Glyma04g05570 Glyma12g12570 Glyma09g03750 Glyma15g02010 Glyma13g01080

Glyma03g34480 Glyma11g37190 Glyma07g10010 Glyma13g09240 Glyma13g24470

Glyma19g32280 Glyma05g01680 Glyma06g20360 Glyma12g08660 Glyma11g02980

Glyma02g02340 Glyma03g00660 Glyma09g37860 Glyma13g29270 Glyma11g30110

Glyma08g11500 Glyma18g06420 Glyma20g01550 Glyma13g31840 Glyma02g07610

Glyma02g35230 Glyma13g41910 Glyma02g15350 Glyma20g32050 Glyma07g39130

Glyma02g04250 Glyma04g32710 Glyma13g28380 Glyma09g33700 Glyma03g29280

Glyma03g35920 Glyma09g32790 Glyma02g00830 Glyma13g05830 Glyma06g05190

Glyma06g03200

63


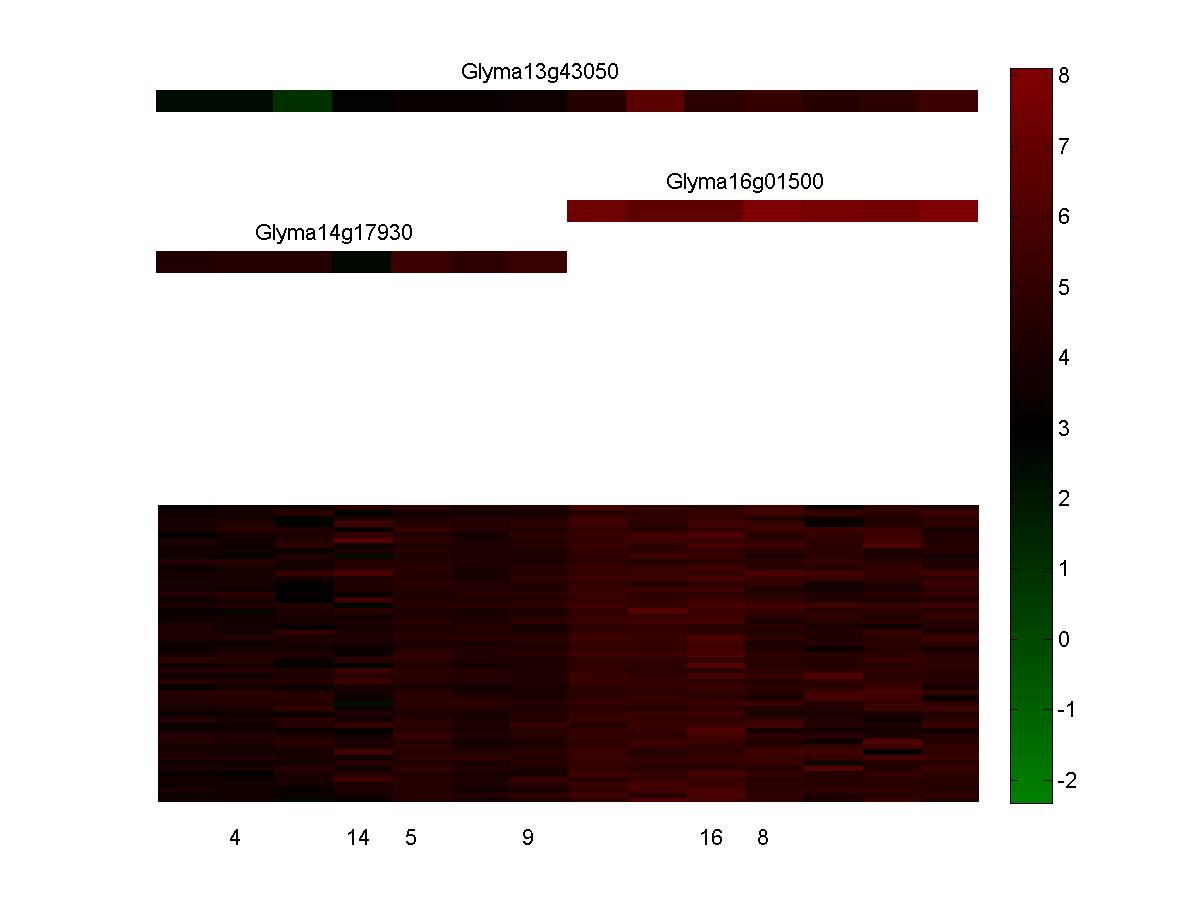


63 Glyma14g17930 CCHC (Zn)

63 Glyma13g43050 AUX-IAA-ARF

63 Glyma16g01500 AP2-EREBP

Glyma13g21870 Glyma08g07860 Glyma07g33130 Glyma06g01030 Glyma15g12050

Glyma04g40740 Glyma18g47760 Glyma03g38830 Glyma10g12260 Glyma07g29720

Glyma20g24040 Glyma07g40330 Glyma13g38730 Glyma12g17950 Glyma13g24750

Glyma19g36750 Glyma17g16350 Glyma05g08480 Glyma08g21290 Glyma05g03940

Glyma03g31440 Glyma01g42520 Glyma19g34900 Glyma04g36090 Glyma13g38420

Glyma08g43720 Glyma15g03560 Glyma07g03000 Glyma09g38560 Glyma18g01390

Glyma08g10330 Glyma01g44090 Glyma17g09250 Glyma06g42130 Glyma04g39680

Glyma19g45180 Glyma03g31800 Glyma08g08590 Glyma01g35650 Glyma06g07880

Glyma10g06610 Glyma10g40810 Glyma08g23250 Glyma19g40390 Glyma08g20940

Glyma03g39850 Glyma10g31660 Glyma02g43050 Glyma08g10160 Glyma13g13260

Glyma02g44710 Glyma07g19170 Glyma12g04040 Glyma05g37260 Glyma08g15470

64


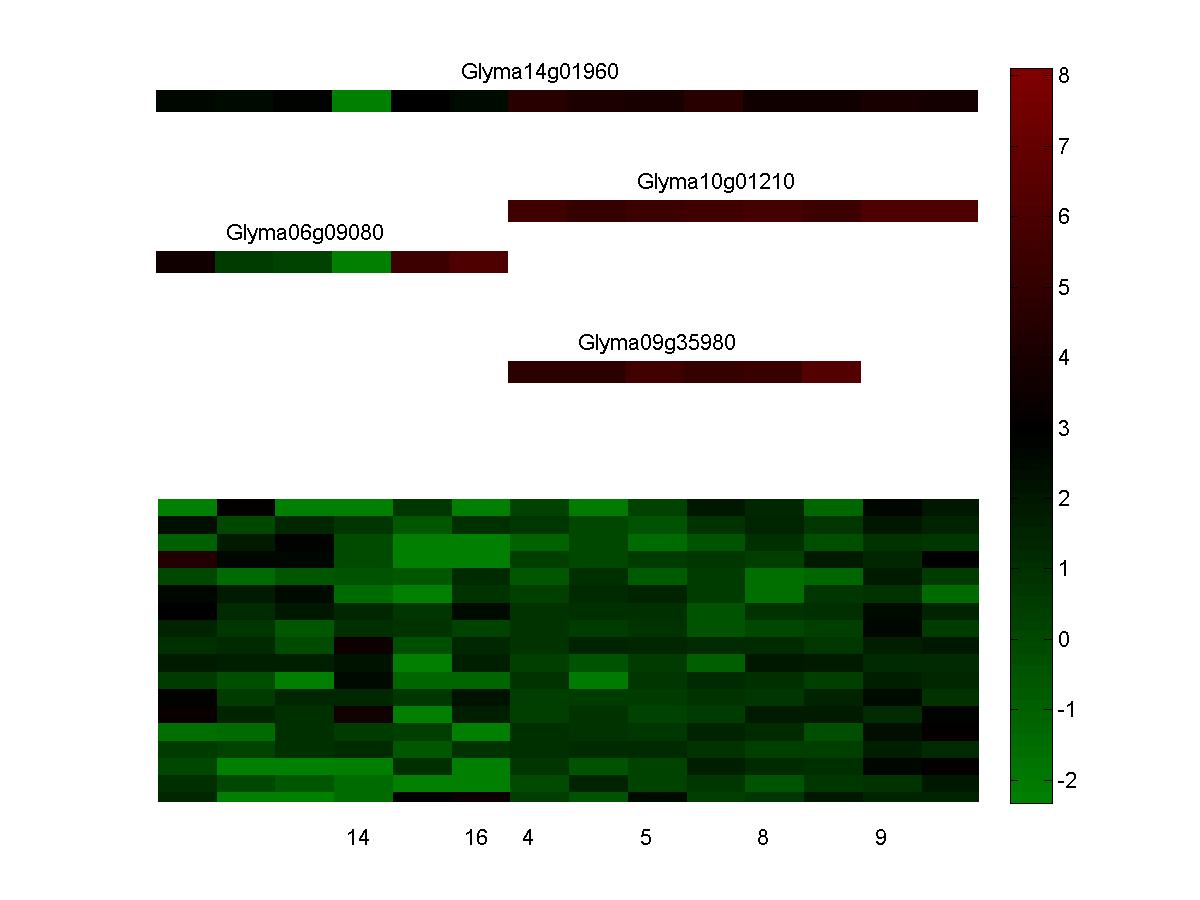


64 Glyma06g09080 C2C2 (Zn) GATA

64 Glyma14g01960 GRAS

64 Glyma10g01210 SBP

64 Glyma09g35980 C3H-type1(Zn)

Glyma04g39700 Glyma02g37090 Glyma17g13000 Glyma15g37870 Glyma15g16370

Glyma05g02790 Glyma20g08860 Glyma01g44060 Glyma03g04760 Glyma16g19270

Glyma19g33400 Glyma08g16380 Glyma10g04350 Glyma13g38490 Glyma10g28140

Glyma04g09200 Glyma06g13470 Glyma10g39980

65


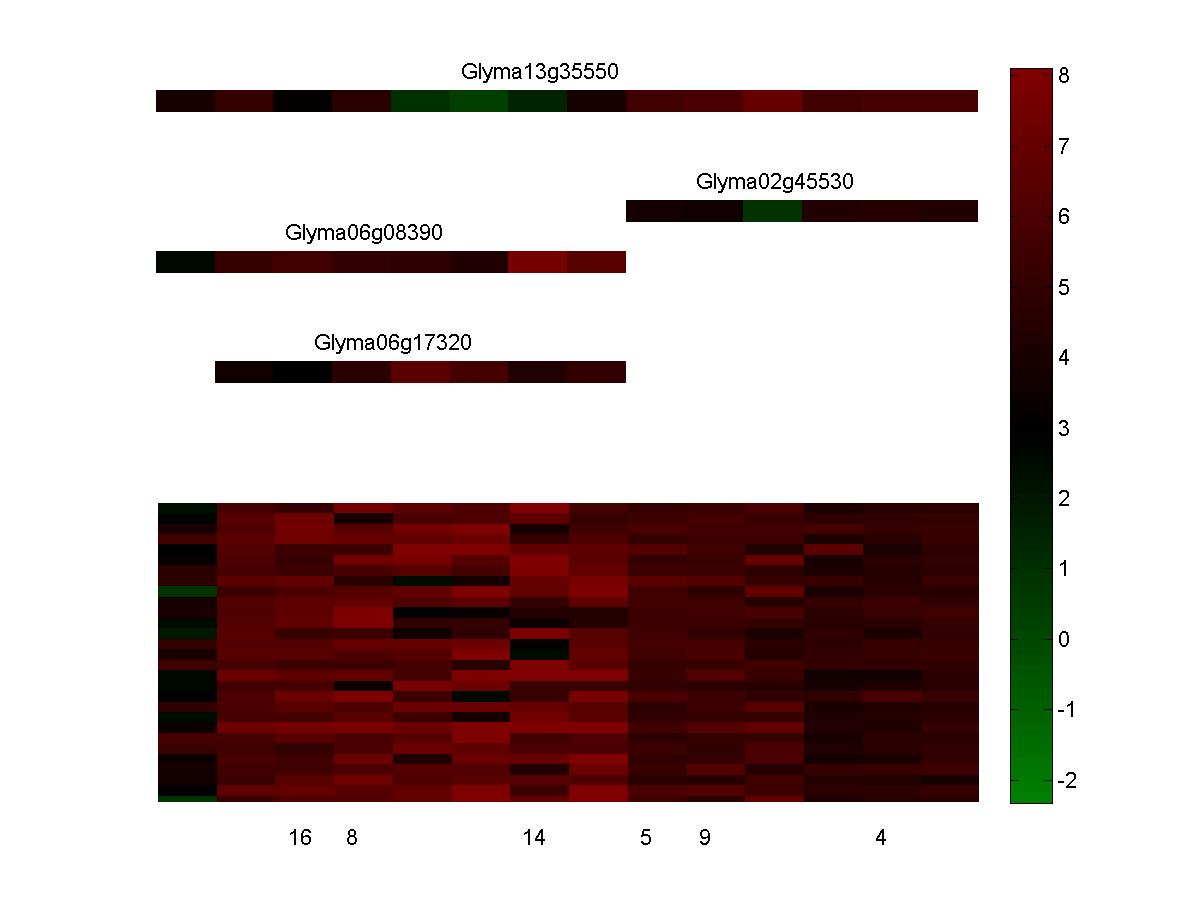


65 Glyma13g35550 NAC

65 Glyma02g45530 WRKY

65 Glyma06g17320 AUX-IAA-ARF

65 Glyma06g08390 BZIP

Glyma12g03570 Glyma19g34380 Glyma01g01310 Glyma16g21620 Glyma07g02500

Glyma16g29370 Glyma05g03100 Glyma09g08120 Glyma10g31630 Glyma01g00950

Glyma05g06050 Glyma02g39090 Glyma13g17420 Glyma20g24670 Glyma07g21100

Glyma14g06160 Glyma06g05460 Glyma13g11100 Glyma09g08550 Glyma02g38260

Glyma12g10960 Glyma05g38150 Glyma09g04940 Glyma13g11090 Glyma17g05060

Glyma08g05820 Glyma16g08590 Glyma03g28850 Glyma08g00370

66


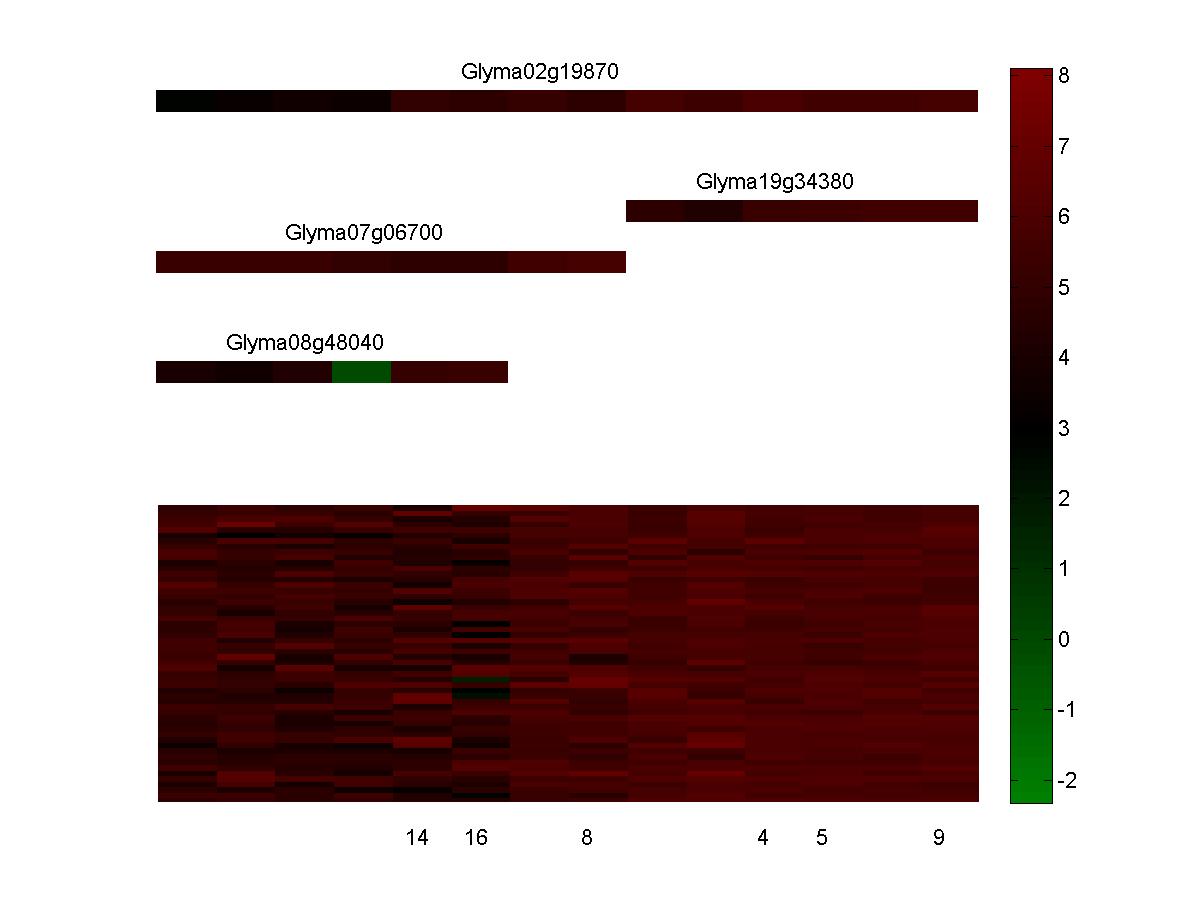


66 Glyma02g19870 BZIP

66 Glyma07g06700 BTB/POZ

66 Glyma08g48040 AS2

66 Glyma19g34380 AUX-IAA-ARF

Glyma05g26420 Glyma16g03250 Glyma07g00580 Glyma11g12720 Glyma07g02460

Glyma08g29130 Glyma04g05250 Glyma17g32910 Glyma20g32850 Glyma06g01410

Glyma20g02570 Glyma11g06830 Glyma07g15510 Glyma12g06020 Glyma18g02590

Glyma09g13840 Glyma10g30440 Glyma04g41750 Glyma18g50240 Glyma03g41630

Glyma13g30990 Glyma13g24500 Glyma19g27360 Glyma14g36540 Glyma06g09150

Glyma11g14050 Glyma03g17450 Glyma14g12570 Glyma08g46830 Glyma18g36750

Glyma06g47830 Glyma19g38910 Glyma18g03340 Glyma15g08390 Glyma04g07360

Glyma20g28860 Glyma10g04920 Glyma16g06850 Glyma12g05510 Glyma07g34730

Glyma03g08270 Glyma17g09830 Glyma12g02030 Glyma03g36230 Glyma05g23240

Glyma04g38140 Glyma09g04440 Glyma01g31360 Glyma07g11890 Glyma07g37100

Glyma13g00380 Glyma10g28370 Glyma11g17920 Glyma08g20060

67


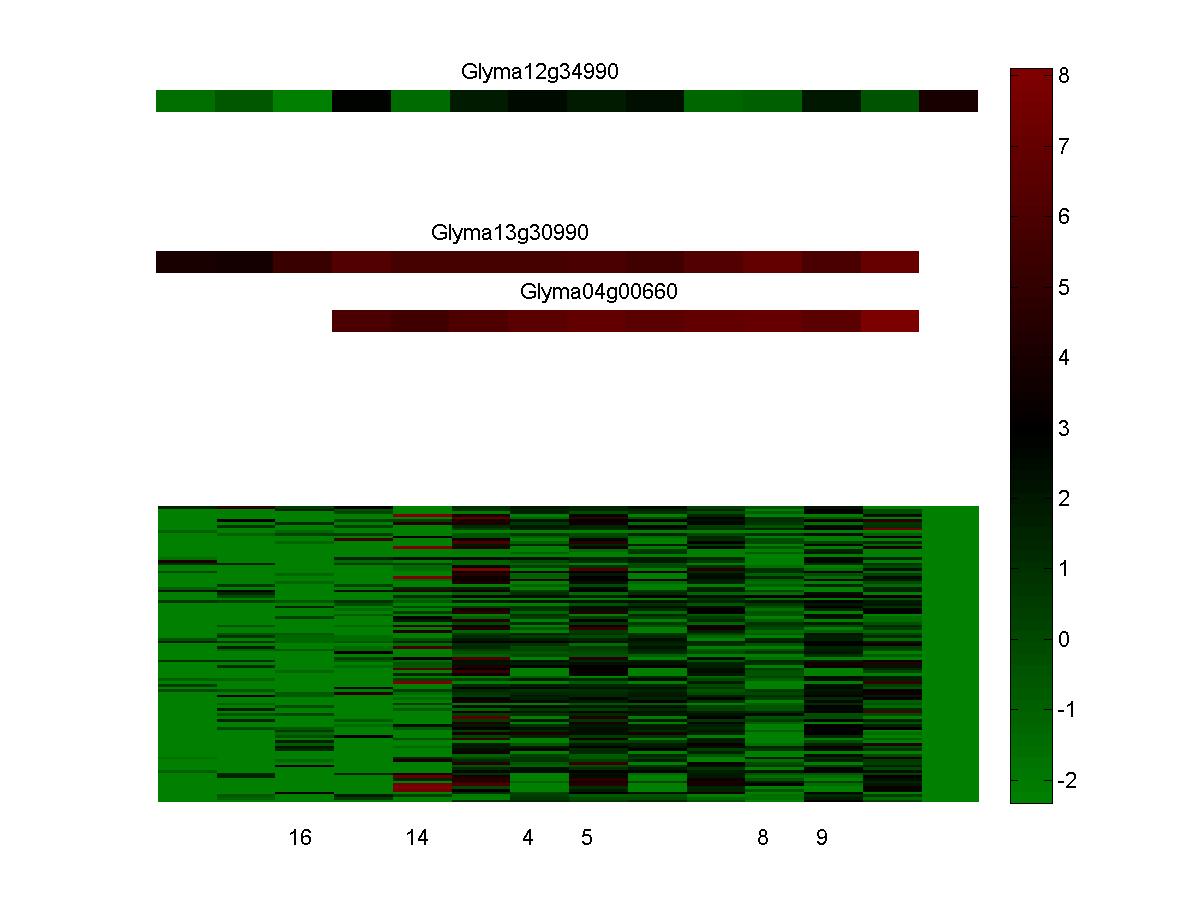


67 Glyma12g34990 NAC

67 Glyma04g00660 CSD

67 Glyma13g30990 AP2-EREBP

Glyma11g06700 Glyma03g01830 Glyma18g48910 Glyma10g42840 Glyma14g02830

Glyma15g17420 Glyma01g04370 Glyma04g01890 Glyma08g09070 Glyma13g35720

Glyma04g14360 Glyma09g27600 Glyma09g30380 Glyma07g11700 Glyma07g09140

Glyma11g13390 Glyma07g08280 Glyma13g37990 Glyma13g23770 Glyma19g43570

Glyma03g40210 Glyma06g40400 Glyma08g44700 Glyma20g33290 Glyma08g24680

Glyma19g43010 Glyma10g05280 Glyma02g48080 Glyma03g40220 Glyma04g00210

Glyma08g08170 Glyma08g18030 Glyma12g06650 Glyma19g42790 Glyma05g12100

Glyma02g34710 Glyma18g47010 Glyma09g15200 Glyma20g29210 Glyma08g17270

Glyma17g02080 Glyma17g07440 Glyma05g25010 Glyma18g06350 Glyma02g14820

Glyma16g33350 Glyma08g03330 Glyma01g32270 Glyma15g32210 Glyma18g16790

Glyma13g39650 Glyma05g01620 Glyma09g31450 Glyma06g03670 Glyma20g03850

Glyma19g03450 Glyma08g23530 Glyma04g13990 Glyma04g11310 Glyma15g17190

Glyma16g02520 Glyma20g30450 Glyma07g38360 Glyma16g17560 Glyma17g12480

Glyma07g08830 Glyma05g36290 Glyma01g38040 Glyma15g06920 Glyma17g06150

Glyma18g53220 Glyma10g32070 Glyma16g063907 Glyma13g39850 Glyma09g29050

Glyma08g25400 Glyma10g25120 Glyma06g47690 Glyma08g05850 Glyma03g01840

Glyma09g31910 Glyma16g32600 Glyma08g19250 Glyma19g25240 Glyma03g02850

Glyma05g12090 Glyma16g26020 Glyma20g06680 Glyma16g04360 Glyma04g02070

Glyma07g08290 Glyma20g22050 Glyma07g38620 Glyma16g06530 Glyma13g27300

Glyma10g25130 Glyma16g01770 Glyma19g29190 Glyma12g33750 Glyma16g32470

Glyma01g45280 Glyma04g42300 Glyma05g00980 Glyma14g03890 Glyma02g27110

Glyma20g34830 Glyma10g10240 Glyma10g37340 Glyma07g11220 Glyma09g33430

68


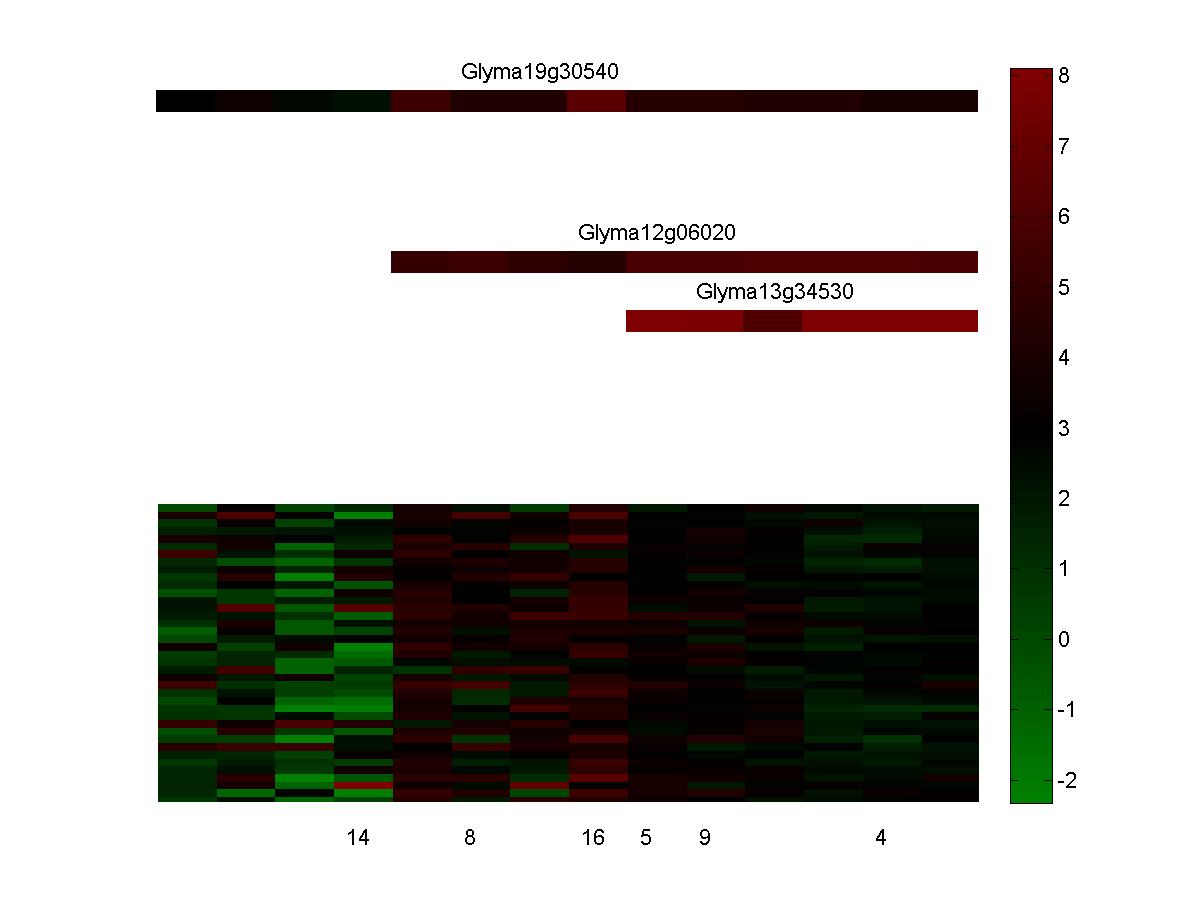


68 Glyma13g34530 C2H2 (Zn)

68 Glyma12g06020 zf-A20

68 Glyma19g30540 NAC

Glyma19g35710 Glyma07g17810 Glyma11g24360 Glyma12g02260 Glyma13g06080

Glyma19g07210 Glyma18g40750 Glyma18g32560 Glyma12g27400 Glyma07g35640

Glyma12g30210 Glyma14g04940 Glyma02g07670 Glyma17g33040 Glyma17g09440

Glyma05g26500 Glyma15g24610 Glyma07g04090 Glyma07g04810 Glyma19g44270

Glyma06g13950 Glyma12g03490 Glyma01g39650 Glyma03g34120 Glyma16g05560

Glyma12g34440 Glyma02g09550 Glyma02g40840 Glyma05g21030 Glyma14g37350

Glyma17g18490 Glyma17g03310 Glyma18g08770 Glyma06g04530 Glyma19g33920

Glyma12g14130 Glyma11g12000 Glyma11g34030 Glyma11g11310

69


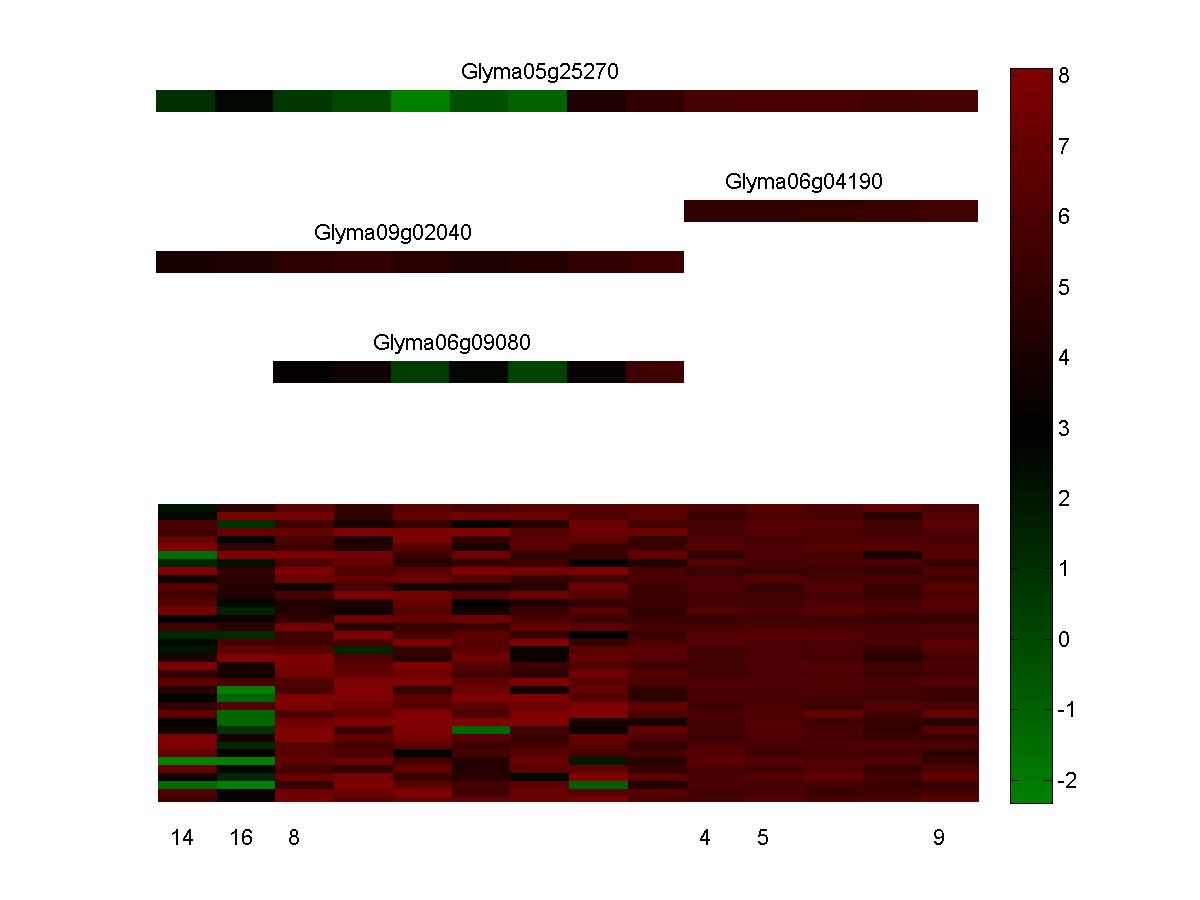


69 Glyma05g25270 WRKY

69 Glyma06g09080 C2C2 (Zn) GATA

69 Glyma06g04190 ABI3/VP1

69 Glyma09g02040 MYB/HD-like

Glyma01g25270 Glyma19g42060 Glyma07g10220 Glyma08g01100 Glyma13g05120

Glyma12g32770 Glyma10g39330 Glyma11g29460 Glyma09g33770 Glyma18g04940

Glyma06g03100 Glyma06g04640 Glyma10g35540 Glyma05g31390 Glyma17g11940

Glyma02g13490 Glyma07g22970 Glyma02g00340 Glyma05g04290 Glyma11g37010

Glyma15g18360 Glyma10g39460 Glyma18g00930 Glyma02g12970 Glyma03g30460

Glyma02g42860 Glyma05g36930 Glyma04g06110 Glyma13g27800 Glyma19g31500

Glyma03g40490 Glyma16g28150 Glyma06g12640 Glyma17g34900 Glyma13g30950

Glyma16g29960 Glyma06g17360 Glyma01g02950

70


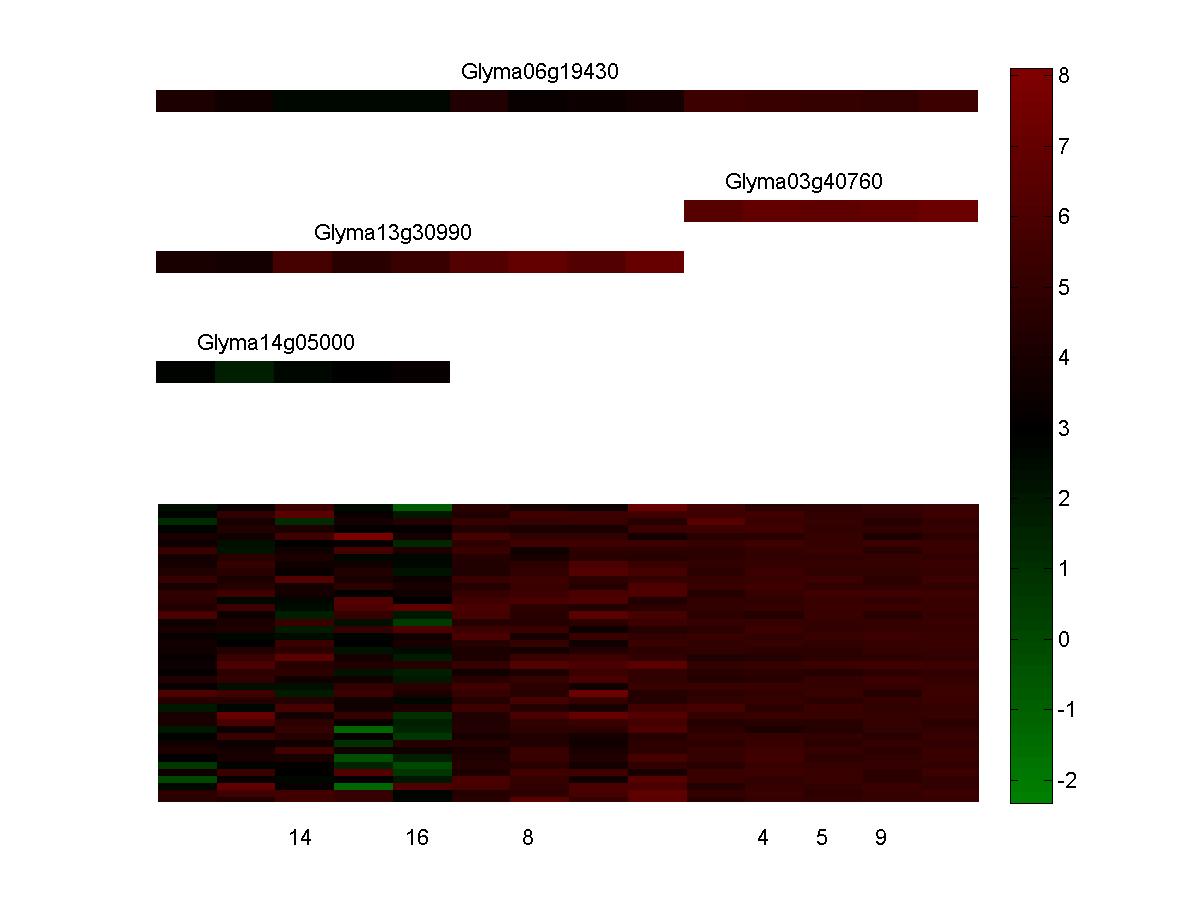


70 Glyma14g05000 BTB/POZ

70 Glyma06g19430 TPR

70 Glyma03g40760 AUX-IAA-ARF

70 Glyma13g30990 AP2-EREBP

Glyma05g37000 Glyma08g07210 Glyma14g08230 Glyma15g40790 Glyma11g05840

Glyma03g23290 Glyma13g27410 Glyma06g44430 Glyma07g16020 Glyma07g04450

Glyma18g00870 Glyma08g39510 Glyma14g40170 Glyma01g07120 Glyma17g02410

Glyma20g16910 Glyma13g17650 Glyma13g06450 Glyma07g17250 Glyma01g36600

Glyma01g37280 Glyma08g22170 Glyma12g22660 Glyma17g16570 Glyma18g02580

Glyma03g30510 Glyma20g02580 Glyma05g24410 Glyma17g13390 Glyma17g36940

Glyma13g23530 Glyma18g10930 Glyma02g10580 Glyma20g16100 Glyma11g10340

Glyma03g15850 Glyma18g11010 Glyma11g36010 Glyma15g16810 Glyma11g08750

Glyma18g53950 Glyma11g12630

DEG gene List

Glyma10g42940 Glyma20g30910 Glyma17g14620 Glyma20g22850 Glyma18g46420 Glyma13g42140 Glyma02g45430 Glyma06g18560 Glyma08g07740 Glyma19g02440 Glyma09g10010 Glyma09g34000 Glyma13g36110 Glyma08g27730 Glyma05g28490 Glyma06g03670 Glyma02g29430 Glyma05g24130 Glyma10g40250 Glyma17g35890 Glyma05g26290 Glyma04g04540 Glyma20g01730 Glyma02g12520 Glyma07g11720 Glyma01g02950 Glyma15g06890 Glyma05g38150 Glyma17g07060 Glyma03g24710 Glyma14g12550 Glyma08g17190 Glyma11g27460 Glyma20g30910 Glyma20g30910 Glyma19g42900 Glyma19g42900 Glyma11g09420 Glyma16g21780 Glyma05g17460 Glyma02g05570 Glyma04g41590 Glyma20g11220 Glyma02g18620 Glyma02g36510 Glyma06g08400 Glyma16g30100 Glyma09g40050 Glyma14g17690 Glyma08g26830 Glyma07g25480 Glyma07g25480 Glyma02g00390 Glyma02g06040 Glyma07g33350 Glyma12g35060 Glyma12g35060 Glyma06g01040 Glyma09g30550 Glyma09g30550 Glyma07g05300 Glyma15g43210 Glyma15g43210 Glyma20g29040 Glyma15g08480 Glyma09g12440 Glyma09g12440 Glyma18g46880 Glyma12g36880 Glyma08g03670 Glyma03g40960 Glyma03g40960 Glyma10g41960 Glyma07g10790 Glyma01g37040 Glyma01g37040 Glyma02g40920 Glyma17g08410 Glyma06g16130 Glyma10g01520 Glyma02g27040 Glyma16g24450 Glyma16g24450 Glyma13g01090 Glyma04g02430 Glyma04g05790 Glyma08g40420 Glyma09g05320 Glyma13g26100 Glyma06g22760 Glyma06g22760 Glyma13g03270 Glyma03g06230 Glyma05g27890 Glyma05g27890 Glyma05g27890 Glyma11g25950 Glyma11g05750 Glyma01g44910 Glyma11g35570 Glyma12g09370 Glyma10g00290 Glyma02g12730 Glyma07g35060 Glyma06g04000 Glyma19g23230 Glyma13g05390 Glyma08g04220 Glyma17g14790 Glyma19g29230 Glyma19g29230 Glyma04g38070 Glyma04g38070 Glyma15g07830 Glyma06g14290 Glyma06g14290 Glyma05g24380 Glyma05g24380 Glyma08g44350 Glyma09g26560 Glyma04g21390 Glyma04g21390 Glyma07g29170 Glyma06g18560 Glyma06g18560 Glyma05g00550 Glyma15g16270 Glyma11g17530 Glyma10g38970 Glyma20g29770 Glyma20g29770 Glyma05g25800 Glyma05g05530 Glyma07g26240 Glyma13g21290 Glyma17g16930 Glyma18g06440 Glyma02g03800 Glyma02g03800 Glyma18g11540 Glyma06g36090 Glyma17g05360 Glyma14g23940 Glyma12g13800 Glyma07g40060 Glyma20g17300 Glyma09g30360 Glyma17g33600 Glyma19g22780 Glyma08g23890 Glyma08g23890 Glyma18g45250 Glyma16g03650 Glyma07g05930 Glyma05g35970 Glyma05g35970 Glyma12g12940 Glyma08g41770 Glyma08g41770 Glyma14g05070 Glyma01g09230 Glyma12g01370 Glyma13g24780 Glyma06g44170 Glyma08g17760 Glyma08g17760 Glyma08g17760 Glyma17g02400 Glyma17g09200 Glyma17g09200 Glyma17g09200 Glyma08g03220 Glyma09g34000 Glyma09g34000 Glyma18g32830 Glyma06g08740 Glyma06g08740 Glyma07g02830 Glyma07g01030 Glyma02g27240 Glyma19g40660 Glyma07g36130 Glyma07g36130 Glyma17g14600 Glyma17g14600 Glyma02g31560 Glyma02g31560 Glyma02g31560 Glyma04g14750 Glyma19g38960 Glyma03g40380 Glyma09g12220 Glyma08g27730 Glyma14g17110 Glyma17g34570 Glyma07g01090 Glyma08g27890 Glyma08g25540 Glyma06g31610 Glyma09g02000 Glyma17g07080 Glyma17g07080 Glyma19g34990 Glyma18g34800 Glyma13g16040 Glyma13g16040 Glyma16g01920 Glyma01g31650 Glyma09g02280 Glyma09g02280 Glyma06g23450 Glyma06g08790 Glyma03g29360 Glyma18g46410 Glyma20g14940 Glyma05g30810 Glyma19g25860 Glyma03g34940 Glyma09g03270 Glyma15g36680 Glyma10g40360 Glyma09g30280 Glyma19g28440 Glyma20g34790 Glyma12g01240 Glyma15g06080 Glyma05g23560 Glyma10g14900 Glyma04g32270 Glyma13g23000 Glyma15g35740 Glyma15g35740 Glyma05g30900 Glyma11g30450 Glyma04g42680 Glyma14g09600 Glyma10g09470 Glyma19g33700 Glyma18g12710 Glyma10g33320 Glyma06g25350 Glyma06g25350 Glyma10g01630 Glyma19g32880 Glyma19g32880 Glyma16g32750 Glyma08g25150 Glyma08g18620 Glyma11g06530 Glyma14g40000 Glyma13g35000 Glyma05g14810 Glyma16g32610 Glyma09g03160 Glyma08g35450 Glyma13g01130 Glyma05g24850 Glyma18g35680 Glyma06g29730 Glyma0021s00420 Glyma18g07290 Glyma11g27120 Glyma11g27120 Glyma08g18730 Glyma15g22280 Glyma05g14350 Glyma07g17280 Glyma08g28790 Glyma12g01690 Glyma04g38730 Glyma04g38730 Glyma04g38730 Glyma04g38730 Glyma07g39450 Glyma10g25700 Glyma03g22000 Glyma01g42810 Glyma08g05500 Glyma13g02250 Glyma12g29760 Glyma10g40960 Glyma18g05650 Glyma04g16120 Glyma14g03960 Glyma11g26480 Glyma03g20890 Glyma20g36390 Glyma08g05480 Glyma01g01610 Glyma20g37420 Glyma14g39100 Glyma03g33060 Glyma06g24400 Glyma06g24400 Glyma10g41430 Glyma10g41430 Glyma08g11120 Glyma08g11120 Glyma15g05990 Glyma14g36660 Glyma18g20560 Glyma06g36230 Glyma15g40050 Glyma09g10520 Glyma17g13470 Glyma10g41310 Glyma18g06140 Glyma01g20450 Glyma01g35830 Glyma01g35830 Glyma12g22240 Glyma03g29830 Glyma13g05540 Glyma16g31270 Glyma04g15380 Glyma05g07760 Glyma06g36210 Glyma06g36210 Glyma16g06660 Glyma18g04800 Glyma18g04800 Glyma17g13120 Glyma13g00670 Glyma13g00670 Glyma03g39880 Glyma04g06520 Glyma04g06520 Glyma04g15400 Glyma04g15400 Glyma14g35960 Glyma12g32540 Glyma12g32540 Glyma04g32450 Glyma11g10910 Glyma05g00510 Glyma04g27570 Glyma04g27570 Glyma16g07210 Glyma16g07210 Glyma16g07210 Glyma10g42710 Glyma10g42710 Glyma12g19010 Glyma06g39260 Glyma17g37340 Glyma19g27410 Glyma09g33870 Glyma12g04160 Glyma16g17210 Glyma06g46200 Glyma09g17250 Glyma0048s00300 Glyma01g04770 Glyma01g04770 Glyma16g00360 Glyma18g14540 Glyma07g07760 Glyma06g14400 Glyma13g38700 Glyma18g44470 Glyma10g28770 Glyma10g41170 Glyma20g31960 Glyma15g07370 Glyma14g38930 Glyma16g07980 Glyma16g07980 Glyma14g32170 Glyma09g32340 Glyma06g06990 Glyma06g06990 Glyma06g06990 Glyma03g42520 Glyma20g17160 Glyma16g22590 Glyma12g07140 Glyma05g15700 Glyma12g17570 Glyma14g28040 Glyma01g30920 Glyma01g30920 Glyma20g01450 Glyma17g16380 Glyma18g10280 Glyma16g22500 Glyma19g41680 Glyma20g13140 Glyma03g42470 Glyma12g10650 Glyma12g07490 Glyma18g32700 Glyma08g17950 Glyma07g37920 Glyma07g08540 Glyma07g08540 Glyma07g08540 Glyma14g37880 Glyma08g07970 Glyma06g41080 Glyma15g18550 Glyma19g42160 Glyma19g42160 Glyma16g04200 Glyma16g27330 Glyma16g27330 Glyma16g27330 Glyma03g29200 Glyma06g37490 Glyma13g32790 Glyma05g05730 Glyma18g40870 Glyma12g14250 Glyma09g31590 Glyma20g29080 Glyma09g38760 Glyma07g08530 Glyma03g31360 Glyma05g09110 Glyma05g09110 Glyma10g39010 Glyma10g39010 Glyma10g39010 Glyma10g39010 Glyma10g39010 Glyma10g39010 Glyma03g24710 Glyma03g24710 Glyma03g24710 Glyma03g24710 Glyma03g24710 Glyma03g24710 Glyma14g12550 Glyma14g12550 Glyma14g12550 Glyma14g12550 Glyma14g12550 Glyma14g12550 Glyma14g12550 Glyma08g46290 Glyma08g46290 Glyma08g46290 Glyma08g46290 Glyma08g46290 Glyma08g46290 Glyma08g46290 Glyma08g46290 Glyma17g22990 Glyma17g22990 Glyma17g22990 Glyma17g22990 Glyma17g22990 Glyma08g17190 Glyma08g17190 Glyma08g17190 Glyma08g17190 Glyma11g27460 Glyma11g27460 Glyma11g27460 Glyma11g27460 Glyma11g27460 Glyma11g27460 Glyma11g27460 Glyma11g27460 Glyma11g27460 Glyma19g31480 Glyma19g31480 Glyma19g31480 Glyma19g31480 Glyma20g18430 Glyma20g18430 Glyma20g18430 Glyma20g18430 Glyma20g18430 Glyma20g18430 Glyma20g30910 Glyma20g30910 Glyma20g30910 Glyma19g42900 Glyma19g42900 Glyma19g42900 Glyma19g42900 Glyma19g42900 Glyma11g09420 Glyma11g09420 Glyma11g09420 Glyma11g09420 Glyma11g09420 Glyma11g09420 Glyma10g04870 Glyma10g04870 Glyma10g04870 Glyma10g04870 Glyma10g04870 Glyma10g04870 Glyma16g21780 Glyma16g21780 Glyma16g21780 Glyma01g30900 Glyma01g30900 Glyma01g30900 Glyma01g30900 Glyma01g30900 Glyma05g17460 Glyma05g17460 Glyma05g17460 Glyma05g17460 Glyma05g17460 Glyma05g17460 Glyma05g17460 Glyma10g23830 Glyma10g23830 Glyma10g23830 Glyma10g23830 Glyma10g23830 Glyma02g05570 Glyma02g05570 Glyma02g05570 Glyma02g05570 Glyma18g36070 Glyma03g41820 Glyma03g41820 Glyma03g41820 Glyma03g41820 Glyma03g41820 Glyma03g41820 Glyma04g41590 Glyma04g41590 Glyma04g41590 Glyma04g41590 Glyma04g41590 Glyma04g41590 Glyma05g38400 Glyma05g38400 Glyma05g38400 Glyma08g43850 Glyma08g43850 Glyma08g43850 Glyma08g43850 Glyma08g43850 Glyma08g43850 Glyma08g43850 Glyma08g43850 Glyma06g17610 Glyma06g17610 Glyma06g17610 Glyma06g17610 Glyma06g17610 Glyma06g17610 Glyma20g16070 Glyma20g16070 Glyma20g16070 Glyma20g16070 Glyma20g16070 Glyma04g32420 Glyma04g32420 Glyma04g32420 Glyma04g32420 Glyma04g32420 Glyma12g09020 Glyma12g09020 Glyma12g09020 Glyma15g03920 Glyma15g03920 Glyma15g03920 Glyma15g03920 Glyma15g03920 Glyma15g03920 Glyma15g03920 Glyma15g03920 Glyma10g04170 Glyma10g04170 Glyma10g04170 Glyma10g04170 Glyma20g11220 Glyma20g11220 Glyma20g11220 Glyma20g11220 Glyma17g14620 Glyma17g14620 Glyma17g14620 Glyma17g14620 Glyma17g14620 Glyma16g08790 Glyma16g08790 Glyma09g29500 Glyma09g29500 Glyma09g29500 Glyma09g29500 Glyma09g29500 Glyma09g29500 Glyma09g29500 Glyma09g29500 Glyma09g29500 Glyma02g18620 Glyma02g18620 Glyma02g18620 Glyma02g18620 Glyma02g18620 Glyma06g07520 Glyma06g07520 Glyma02g36510 Glyma02g36510 Glyma02g36510 Glyma02g36510 Glyma06g08400 Glyma06g08400 Glyma16g30100 Glyma16g30100 Glyma16g30100 Glyma16g30100 Glyma16g30100 Glyma16g30100 Glyma13g31210 Glyma13g31210 Glyma13g31210 Glyma09g40050 Glyma09g40050 Glyma09g40050 Glyma09g40050 Glyma09g40050 Glyma09g40050 Glyma09g40050 Glyma14g17690 Glyma08g26830 Glyma08g26830 Glyma08g26830 Glyma08g26830 Glyma18g36600 Glyma18g36600 Glyma18g36600 Glyma18g36600 Glyma18g36600 Glyma07g25480 Glyma07g25480 Glyma07g25480 Glyma07g25480 Glyma07g25480 Glyma02g00390 Glyma02g00390 Glyma02g00390 Glyma02g00390 Glyma02g00390 Glyma02g00390 Glyma02g00390 Glyma02g00390 Glyma17g07700 Glyma17g07700 Glyma17g07700 Glyma17g07700 Glyma17g07700 Glyma02g06040 Glyma02g06040 Glyma02g06040 Glyma02g06040 Glyma13g26530 Glyma13g26530 Glyma13g26530 Glyma13g26530 Glyma13g26530 Glyma13g26530 Glyma04g09810 Glyma04g09810 Glyma15g24350 Glyma15g24350 Glyma15g24350 Glyma15g24350 Glyma15g24350 Glyma14g38560 Glyma14g38560 Glyma14g38560 Glyma14g38560 Glyma14g38560 Glyma14g38560 Glyma02g33990 Glyma02g33990 Glyma02g33990 Glyma07g33350 Glyma07g33350 Glyma07g33350 Glyma07g33350 Glyma07g33350 Glyma12g35060 Glyma12g35060 Glyma12g35060 Glyma12g35060 Glyma20g18020 Glyma20g18020 Glyma20g18020 Glyma20g18020 Glyma20g18020 Glyma02g40460 Glyma02g40460 Glyma02g40460 Glyma02g40460 Glyma02g40460 Glyma14g11040 Glyma14g11040 Glyma09g30670 Glyma09g30670 Glyma09g30670 Glyma09g30670 Glyma08g11020 Glyma08g11020 Glyma08g11020 Glyma08g11020 Glyma06g01040 Glyma06g01040 Glyma06g01040 Glyma09g30550 Glyma09g30550 Glyma09g30550 Glyma09g30550 Glyma09g30550 Glyma02g43330 Glyma02g43330 Glyma02g43330 Glyma02g43330 Glyma06g06560 Glyma12g11250 Glyma19g37810 Glyma19g37810 Glyma20g22850 Glyma07g05300 Glyma07g05300 Glyma07g05300 Glyma07g05300 Glyma07g05300 Glyma09g32960 Glyma09g32960 Glyma09g32960 Glyma09g32960 Glyma09g32960 Glyma09g32960 Glyma04g32960 Glyma04g32960 Glyma04g32960 Glyma04g32960 Glyma15g43210 Glyma15g43210 Glyma15g43210 Glyma15g43210 Glyma08g26540 Glyma08g26540 Glyma08g26540 Glyma08g26540 Glyma08g26540 Glyma20g28550 Glyma20g28550 Glyma20g28550 Glyma20g28550 Glyma20g28550 Glyma14g01300 Glyma14g01300 Glyma14g01300 Glyma14g01300 Glyma14g01300 Glyma18g41350 Glyma18g41350 Glyma18g41350 Glyma18g41350 Glyma06g21710 Glyma06g21710 Glyma06g21710 Glyma06g21710 Glyma06g21710 Glyma06g21710 Glyma06g21710 Glyma03g23990 Glyma18g52550 Glyma18g52550 Glyma18g52550 Glyma18g52550 Glyma18g52550 Glyma18g52550 Glyma18g52550 Glyma20g29040 Glyma20g29040 Glyma20g29040 Glyma20g29040 Glyma20g29040 Glyma20g29040 Glyma12g18260 Glyma12g18260 Glyma12g18260 Glyma12g18260 Glyma15g08480 Glyma15g08480 Glyma15g08480 Glyma15g08480 Glyma09g12440 Glyma09g12440 Glyma09g12440 Glyma18g46880 Glyma18g46880 Glyma08g08620 Glyma08g08620 Glyma08g08620 Glyma12g36880 Glyma12g36880 Glyma12g36880 Glyma12g36880 Glyma12g36880 Glyma12g36880 Glyma13g20170 Glyma13g20170 Glyma13g20170 Glyma13g20170 Glyma13g20170 Glyma13g20170 Glyma04g06560 Glyma04g06560 Glyma04g06560 Glyma04g06560 Glyma04g06560 Glyma04g06560 Glyma0844s00200 Glyma0844s00200 Glyma0844s00200 Glyma0844s00200 Glyma0844s00200 Glyma0844s00200 Glyma0844s00200 Glyma08g03670 Glyma08g03670 Glyma08g03670 Glyma08g03670 Glyma08g03670 Glyma08g03670 Glyma08g03670 Glyma03g40960 Glyma03g40960 Glyma03g40960 Glyma03g40960 Glyma18g46420 Glyma18g46420 Glyma18g46420 Glyma18g46420 Glyma14g08260 Glyma14g08260 Glyma14g08260 Glyma14g08260 Glyma14g08260 Glyma17g01690 Glyma17g01690 Glyma17g01690 Glyma17g01690 Glyma17g01690 Glyma18g46730 Glyma18g46730 Glyma18g46730 Glyma03g00980 Glyma03g00980 Glyma03g00980 Glyma03g00980 Glyma14g10480 Glyma14g10480 Glyma14g10480 Glyma14g10480 Glyma18g44730 Glyma18g44730 Glyma18g44730 Glyma18g44730 Glyma18g44730 Glyma05g27060 Glyma05g27060 Glyma05g27060 Glyma05g27060 Glyma05g27060 Glyma05g27060 Glyma05g27060 Glyma05g27060 Glyma05g27060 Glyma05g27060 Glyma18g46710 Glyma18g46710 Glyma18g46710 Glyma18g46710 Glyma18g46710 Glyma18g46710 Glyma02g02800 Glyma02g02800 Glyma02g02800 Glyma02g02800 Glyma03g26250 Glyma03g26250 Glyma03g26250 Glyma05g25630 Glyma05g25630 Glyma05g25630 Glyma11g35610 Glyma11g35610 Glyma11g35610 Glyma11g35610 Glyma11g35610 Glyma19g41360 Glyma19g41360 Glyma19g41360 Glyma12g14810 Glyma12g14810 Glyma10g41960 Glyma10g41960 Glyma10g41960 Glyma10g41960 Glyma10g41960 Glyma10g41960 Glyma07g34380 Glyma07g34380 Glyma07g34380 Glyma07g34380 Glyma07g34380 Glyma07g34380 Glyma07g34380 Glyma07g34380 Glyma07g10790 Glyma07g10790 Glyma07g10790 Glyma07g10790 Glyma07g10790 Glyma07g10790 Glyma08g36280 Glyma08g36280 Glyma08g36280 Glyma08g36280 Glyma11g17090 Glyma11g17090 Glyma11g17090 Glyma11g17090 Glyma11g17090 Glyma01g37040 Glyma01g37040 Glyma02g11400 Glyma02g11400 Glyma02g11400 Glyma02g11400 Glyma02g40920 Glyma02g40920 Glyma02g40920 Glyma02g40920 Glyma02g40920 Glyma02g40920 Glyma02g40920 Glyma06g45170 Glyma06g45170 Glyma06g36290 Glyma06g36290 Glyma06g36290 Glyma06g36290 Glyma06g36290 Glyma06g36290 Glyma03g36840 Glyma03g36840 Glyma14g10650 Glyma14g10650 Glyma14g10650 Glyma18g05590 Glyma18g05590 Glyma18g05590 Glyma18g05590 Glyma17g08410 Glyma17g08410 Glyma10g09220 Glyma10g09220 Glyma10g09220 Glyma10g09220 Glyma10g09220 Glyma10g09220 Glyma06g16130 Glyma06g16130 Glyma06g16130 Glyma06g40810 Glyma10g01520 Glyma10g01520 Glyma10g01520 Glyma06g12620 Glyma06g12620 Glyma06g12620 Glyma06g12620 Glyma06g12620 Glyma06g12620 Glyma06g12620 Glyma02g27040 Glyma02g27040 Glyma02g27040 Glyma02g27040 Glyma02g27040 Glyma02g27040 Glyma02g27040 Glyma02g27040 Glyma02g27040 Glyma02g27040 Glyma13g20470 Glyma13g20470 Glyma13g20470 Glyma13g20470 Glyma13g20470 Glyma13g20470 Glyma13g20470 Glyma13g20470 Glyma13g20470 Glyma19g01650 Glyma19g01650 Glyma19g01650 Glyma19g01650 Glyma19g01650 Glyma19g01650 Glyma05g30740 Glyma05g30740 Glyma05g30740 Glyma05g30740 Glyma03g33440 Glyma03g33440 Glyma03g33440 Glyma03g08730 Glyma03g08730 Glyma03g08730 Glyma03g08730 Glyma03g08730 Glyma03g08730 Glyma03g08730 Glyma08g44060 Glyma08g44060 Glyma08g44060 Glyma08g44060 Glyma08g44060 Glyma08g44060 Glyma08g44060 Glyma16g24450 Glyma16g24450 Glyma16g24450 Glyma16g24450 Glyma16g24450 Glyma13g01090 Glyma13g01090 Glyma13g01090 Glyma04g02430 Glyma04g02430 Glyma04g02430 Glyma20g21090 Glyma20g21090 Glyma20g21090 Glyma20g21090 Glyma20g21090 Glyma20g21090 Glyma20g21090 Glyma20g21090 Glyma07g39000 Glyma07g39000 Glyma07g39000 Glyma07g39000 Glyma07g39000 Glyma04g05790 Glyma04g05790 Glyma04g05790 Glyma04g05790 Glyma04g05790 Glyma04g05790 Glyma04g05790 Glyma19g12410 Glyma19g12410 Glyma19g12410 Glyma15g14410 Glyma15g14410 Glyma15g14410 Glyma15g14410 Glyma12g36840 Glyma12g36840 Glyma08g15330 Glyma08g15330 Glyma08g15330 Glyma08g15330 Glyma08g15330 Glyma08g10000 Glyma08g10000 Glyma08g10000 Glyma08g10000 Glyma08g10000 Glyma08g10000 Glyma08g10000 Glyma08g10000 Glyma08g40420 Glyma08g40420 Glyma08g40420 Glyma08g40420 Glyma08g40420 Glyma08g40420 Glyma08g40420 Glyma08g40420 Glyma07g08350 Glyma07g08350 Glyma07g08350 Glyma07g08350 Glyma07g08350 Glyma07g08350 Glyma07g08350 Glyma18g51250 Glyma18g51250 Glyma15g19490 Glyma15g19490 Glyma15g19490 Glyma15g19490 Glyma15g19490 Glyma15g19490 Glyma09g05320 Glyma09g05320 Glyma09g05320 Glyma09g05320 Glyma09g21170 Glyma09g21170 Glyma09g21170 Glyma09g21170 Glyma09g21170 Glyma09g21170 Glyma02g26550 Glyma02g26550 Glyma02g26550 Glyma02g26550 Glyma02g26550 Glyma05g25890 Glyma05g25890 Glyma05g25890 Glyma13g26100 Glyma13g26100 Glyma13g26100 Glyma13g26100 Glyma13g26100 Glyma13g17790 Glyma13g17790 Glyma13g17790 Glyma06g22760 Glyma06g22760 Glyma06g22760 Glyma06g22760 Glyma06g22760 Glyma06g22760 Glyma12g06860 Glyma12g06860 Glyma12g06860 Glyma12g06860 Glyma12g06860 Glyma12g06860 Glyma12g06860 Glyma10g23650 Glyma10g23650 Glyma10g23650 Glyma10g23650 Glyma19g27610 Glyma13g43190 Glyma13g43190 Glyma13g43190 Glyma13g43190 Glyma13g43190 Glyma13g43190 Glyma03g02240 Glyma03g02240 Glyma03g02240 Glyma05g08180 Glyma05g08180 Glyma05g08180 Glyma05g08180 Glyma05g08180 Glyma05g08180 Glyma13g03270 Glyma13g03270 Glyma13g03270 Glyma06g15520 Glyma06g15520 Glyma06g15520 Glyma06g15520 Glyma06g15520 Glyma06g15520 Glyma03g06230 Glyma13g42140 Glyma13g42140 Glyma13g42140 Glyma13g42140 Glyma02g45430 Glyma02g45430 Glyma02g45430 Glyma05g27890 Glyma05g27890 Glyma05g27890 Glyma05g27890 Glyma05g27890 Glyma05g27890 Glyma18g14990 Glyma18g14990 Glyma18g14990 Glyma18g14990 Glyma08g42800 Glyma08g42800 Glyma08g42800 Glyma08g42800 Glyma08g42800 Glyma11g25950 Glyma11g25950 Glyma11g25950 Glyma11g25950 Glyma11g25950 Glyma11g25950 Glyma11g05750 Glyma11g05750 Glyma11g05750 Glyma11g05750 Glyma11g05750 Glyma11g34080 Glyma11g34080 Glyma01g44910 Glyma01g44910 Glyma01g44910 Glyma01g44910 Glyma01g44910 Glyma01g44910 Glyma01g44910 Glyma07g21000 Glyma07g21000 Glyma09g29700 Glyma09g29700 Glyma09g29700 Glyma09g29700 Glyma09g29700 Glyma09g29700 Glyma01g15020 Glyma01g15020 Glyma01g15020 Glyma11g35570 Glyma11g35570 Glyma11g35570 Glyma11g35570 Glyma11g35570 Glyma11g35570 Glyma11g35570 Glyma11g35570 Glyma10g36280 Glyma10g36280 Glyma10g36280 Glyma10g36280 Glyma10g36280 Glyma10g36280 Glyma10g36280 Glyma10g36280 Glyma15g42280 Glyma15g42280 Glyma15g42280 Glyma15g42280 Glyma15g42280 Glyma15g42280 Glyma18g50580 Glyma18g50580 Glyma18g50580 Glyma18g50580 Glyma10g37030 Glyma10g37030 Glyma10g37030 Glyma08g47710 Glyma08g47710 Glyma18g37390 Glyma18g37390 Glyma18g37390 Glyma18g37390 Glyma05g37370 Glyma05g37370 Glyma05g37370 Glyma05g37370 Glyma08g43480 Glyma08g43480 Glyma08g43480 Glyma08g43480 Glyma08g14760 Glyma08g14760 Glyma08g14760 Glyma08g14760 Glyma08g14760 Glyma08g14760 Glyma04g11100 Glyma04g11100 Glyma04g11100 Glyma04g11100 Glyma04g11100 Glyma08g39500 Glyma08g39500 Glyma08g39500 Glyma08g39500 Glyma08g39500 Glyma08g39500 Glyma08g39500 Glyma12g09370 Glyma12g09370 Glyma12g09370 Glyma12g09370 Glyma12g09370 Glyma12g09370 Glyma12g09370 Glyma11g12130 Glyma11g12130 Glyma11g12130 Glyma11g12130 Glyma11g12130 Glyma19g43630 Glyma19g43630 Glyma19g43630 Glyma19g43630 Glyma19g43630 Glyma19g43630 Glyma19g43630 Glyma10g00290 Glyma10g00290 Glyma10g00290 Glyma10g00290 Glyma18g28580 Glyma18g28580 Glyma18g28580 Glyma18g28580 Glyma10g40300 Glyma10g40300 Glyma10g40300 Glyma10g40300 Glyma10g40300 Glyma10g40300 Glyma10g40300 Glyma02g12730 Glyma02g12730 Glyma02g12730 Glyma06g36580 Glyma06g36580 Glyma06g36580 Glyma06g36580 Glyma06g36580 Glyma07g35060 Glyma07g35060 Glyma07g35060 Glyma07g35060 Glyma07g35060 Glyma06g04000 Glyma06g04000 Glyma06g04000 Glyma06g04000 Glyma06g04000 Glyma06g04000 Glyma06g04000 Glyma19g23230 Glyma19g23230 Glyma19g23230 Glyma19g23230 Glyma17g35620 Glyma17g35620 Glyma17g35620 Glyma17g35620 Glyma17g35620 Glyma17g35620 Glyma17g35620 Glyma13g05390 Glyma13g05390 Glyma13g05390 Glyma13g05390 Glyma08g04220 Glyma08g04220 Glyma08g04220 Glyma08g04220 Glyma17g14790 Glyma17g14790 Glyma17g14790 Glyma19g29230 Glyma19g29230 Glyma19g29230 Glyma04g38070 Glyma04g38070 Glyma04g38070 Glyma04g38070 Glyma04g38070 Glyma04g38070 Glyma04g38070 Glyma04g38070 Glyma18g28150 Glyma18g28150 Glyma18g28150 Glyma18g28150 Glyma18g28150 Glyma18g28150 Glyma16g12130 Glyma16g12130 Glyma16g12130 Glyma16g03150 Glyma16g03150 Glyma16g03150 Glyma16g03150 Glyma15g07830 Glyma15g07830 Glyma15g07830 Glyma15g07830 Glyma15g07830 Glyma15g07830 Glyma15g07830 Glyma15g07830 Glyma15g07830 Glyma15g07830 Glyma06g14290 Glyma06g14290 Glyma06g14290 Glyma06g14290 Glyma06g14290 Glyma05g24380 Glyma05g24380 Glyma05g24380 Glyma05g24380 Glyma08g44350 Glyma08g44350 Glyma08g44350 Glyma08g44350 Glyma08g44350 Glyma08g44350 Glyma08g44350 Glyma08g44350 Glyma08g44350 Glyma08g44350 Glyma09g26560 Glyma09g26560 Glyma09g26560 Glyma09g26560 Glyma09g26560 Glyma09g26560 Glyma08g45880 Glyma08g45880 Glyma08g45880 Glyma03g07230 Glyma03g07230 Glyma03g07230 Glyma03g07230 Glyma04g14710 Glyma04g14710 Glyma04g14710 Glyma04g14710 Glyma04g14710 Glyma04g14710 Glyma04g14710 Glyma04g14710 Glyma04g21390 Glyma04g21390 Glyma04g21390 Glyma04g21390 Glyma04g21390 Glyma04g21390 Glyma04g21390 Glyma16g09320 Glyma16g09320 Glyma16g09320 Glyma16g09320 Glyma16g09320 Glyma16g09320 Glyma16g09320 Glyma08g08740 Glyma08g08740 Glyma08g08740 Glyma08g08740 Glyma07g29170 Glyma07g29170 Glyma06g18560 Glyma06g18560 Glyma06g18560 Glyma06g18560 Glyma06g18560 Glyma06g18560 Glyma05g00550 Glyma05g00550 Glyma05g00550 Glyma05g00550 Glyma05g00550 Glyma05g00550 Glyma15g16270 Glyma15g16270 Glyma15g16270 Glyma15g16270 Glyma15g16270 Glyma15g16270 Glyma15g16270 Glyma15g16270 Glyma15g16270 Glyma15g16270 Glyma01g33880 Glyma01g33880 Glyma01g33880 Glyma01g33880 Glyma01g33880 Glyma01g33880 Glyma01g33880 Glyma01g33880 Glyma01g33880 Glyma01g33880 Glyma13g17730 Glyma13g17730 Glyma13g17730 Glyma13g17730 Glyma13g17730 Glyma13g17730 Glyma13g17730 Glyma08g07740 Glyma08g07740 Glyma08g07740 Glyma08g07740 Glyma08g07740 Glyma08g07740 Glyma11g17530 Glyma11g17530 Glyma11g17530 Glyma11g17530 Glyma11g17530 Glyma11g17530 Glyma11g17530 Glyma11g17530 Glyma10g38970 Glyma10g38970 Glyma10g38970 Glyma10g38970 Glyma10g38970 Glyma10g38970 Glyma10g38970 Glyma10g38970 Glyma10g38970 Glyma10g38970 Glyma10g38970 Glyma20g29770 Glyma20g29770 Glyma20g29770 Glyma05g25800 Glyma05g25800 Glyma05g25800 Glyma05g25800 Glyma05g25800 Glyma05g05530 Glyma05g05530 Glyma05g05530 Glyma05g05530 Glyma05g05530 Glyma05g05530 Glyma05g05530 Glyma02g22320 Glyma02g22320 Glyma02g22320 Glyma02g22320 Glyma02g22320 Glyma02g22320 Glyma02g22320 Glyma02g02720 Glyma02g02720 Glyma02g02720 Glyma02g02720 Glyma02g02720 Glyma02g02720 Glyma02g02720 Glyma19g30640 Glyma19g30640 Glyma19g30640 Glyma19g30640 Glyma07g26240 Glyma07g26240 Glyma07g26240 Glyma07g26240 Glyma07g26240 Glyma07g26240 Glyma02g11520 Glyma02g11520 Glyma02g11520 Glyma02g11520 Glyma02g11520 Glyma02g11520 Glyma02g11520 Glyma02g11520 Glyma02g11520 Glyma02g11520 Glyma02g11520 Glyma13g21290 Glyma13g21290 Glyma13g21290 Glyma03g29750 Glyma03g29750 Glyma03g29750 Glyma03g29750 Glyma19g28840 Glyma19g28840 Glyma19g28840 Glyma17g16930 Glyma17g16930 Glyma17g16930 Glyma08g29120 Glyma08g29120 Glyma08g29120 Glyma08g29120 Glyma08g29120 Glyma18g06440 Glyma18g06440 Glyma18g06440 Glyma18g06440 Glyma17g07880 Glyma17g07880 Glyma17g07880 Glyma17g07880 Glyma17g07880 Glyma17g07880 Glyma05g29860 Glyma05g29860 Glyma05g29860 Glyma05g29860 Glyma18g18840 Glyma18g18840 Glyma18g18840 Glyma18g18840 Glyma18g18840 Glyma09g26340 Glyma09g26340 Glyma09g26340 Glyma09g26340 Glyma02g03800 Glyma02g03800 Glyma02g03800 Glyma02g03800 Glyma18g34340 Glyma18g34340 Glyma18g34340 Glyma18g34340 Glyma18g34340 Glyma18g34340 Glyma18g34340 Glyma18g34340 Glyma18g34340 Glyma18g34340 Glyma03g03970 Glyma03g03970 Glyma03g03970 Glyma03g03970 Glyma03g03970 Glyma03g03970 Glyma18g11540 Glyma18g11540 Glyma18g11540 Glyma18g11540 Glyma18g11540 Glyma05g31600 Glyma05g31600 Glyma05g31600 Glyma05g31600 Glyma05g31600 Glyma19g31620 Glyma19g31620 Glyma19g31620 Glyma19g31620 Glyma19g31620 Glyma19g31620 Glyma19g31620 Glyma20g25160 Glyma20g25160 Glyma20g25160 Glyma20g25160 Glyma05g23570 Glyma05g23570 Glyma05g23570 Glyma04g03530 Glyma04g03530 Glyma04g03530 Glyma04g03530 Glyma01g16360 Glyma01g16360 Glyma01g16360 Glyma18g51070 Glyma18g51070 Glyma18g51070 Glyma18g51070 Glyma18g51070 Glyma18g51070 Glyma06g36090 Glyma06g36090 Glyma06g36090 Glyma06g36090 Glyma06g36090 Glyma06g36090 Glyma19g02440 Glyma19g02440 Glyma19g02440 Glyma19g02440 Glyma19g02440 Glyma17g05360 Glyma17g05360 Glyma17g05360 Glyma17g05360 Glyma17g05360 Glyma17g05360 Glyma07g07010 Glyma07g07010 Glyma07g07010 Glyma07g07010 Glyma19g02890 Glyma19g02890 Glyma19g02890 Glyma19g02890 Glyma19g02890 Glyma04g11940 Glyma04g11940 Glyma04g11940 Glyma04g11940 Glyma04g11940 Glyma04g11940 Glyma15g05510 Glyma15g05510 Glyma15g05510 Glyma20g22510 Glyma20g22510 Glyma20g22510 Glyma20g22510 Glyma20g22510 Glyma20g22510 Glyma20g22510 Glyma20g22510 Glyma04g09980 Glyma04g09980 Glyma04g09980 Glyma04g09980 Glyma14g23940 Glyma14g23940 Glyma14g23940 Glyma12g13800 Glyma12g13800 Glyma12g13800 Glyma12g13800 Glyma12g13800 Glyma07g40060 Glyma07g40060 Glyma07g40060 Glyma07g40060 Glyma07g40060 Glyma07g40060 Glyma07g40060 Glyma20g26370 Glyma20g26370 Glyma20g26370 Glyma20g26370 Glyma20g26370 Glyma20g26370 Glyma20g26370 Glyma03g20900 Glyma03g20900 Glyma03g20900 Glyma03g20900 Glyma15g12080 Glyma15g12080 Glyma15g12080 Glyma15g12080 Glyma12g16450 Glyma12g16450 Glyma12g16450 Glyma16g27300 Glyma16g27300 Glyma16g27300 Glyma16g27300 Glyma20g17300 Glyma20g17300 Glyma08g28140 Glyma08g28140 Glyma15g28320 Glyma15g28320 Glyma15g28320 Glyma15g28320 Glyma07g08880 Glyma07g08880 Glyma07g08880 Glyma07g08880 Glyma07g08880 Glyma17g33600 Glyma17g33600 Glyma17g33600 Glyma17g33600 Glyma17g33600 Glyma17g33600 Glyma17g33600 Glyma02g00550 Glyma02g00550 Glyma02g00550 Glyma02g00550 Glyma02g00550 Glyma19g22780 Glyma19g22780 Glyma19g22780 Glyma19g22780 Glyma14g05860 Glyma14g05860 Glyma14g05860 Glyma20g25880 Glyma20g25880 Glyma20g25880 Glyma20g25880 Glyma20g25880 Glyma20g25880 Glyma20g25880 Glyma06g18660 Glyma06g18660 Glyma06g18660 Glyma06g18660 Glyma08g23890 Glyma08g23890 Glyma08g23890 Glyma08g23890 Glyma08g23890 Glyma06g43210 Glyma06g43210 Glyma06g43210 Glyma15g12010 Glyma15g12010 Glyma15g12010 Glyma18g45250 Glyma18g45250 Glyma18g45250 Glyma18g45250 Glyma18g45250 Glyma09g10010 Glyma09g10010 Glyma09g10010 Glyma09g10010 Glyma09g10010 Glyma16g03650 Glyma16g03650 Glyma16g03650 Glyma15g16090 Glyma15g16090 Glyma15g16090 Glyma15g16090 Glyma04g05100 Glyma04g05100 Glyma04g05100 Glyma04g05100 Glyma04g05100 Glyma06g11830 Glyma06g11830 Glyma06g11830 Glyma06g11830 Glyma06g11830 Glyma06g11830 Glyma20g21470 Glyma20g21470 Glyma20g21470 Glyma20g21470 Glyma20g21470 Glyma20g21470 Glyma07g05930 Glyma07g05930 Glyma07g05930 Glyma09g29490 Glyma09g29490 Glyma09g29490 Glyma05g35970 Glyma05g35970 Glyma05g35970 Glyma05g35970 Glyma05g35970 Glyma05g35690 Glyma03g36270 Glyma03g36270 Glyma03g36270 Glyma07g17930 Glyma07g17930 Glyma14g32700 Glyma14g32700 Glyma14g32700 Glyma14g32700 Glyma14g32700 Glyma14g32700 Glyma12g12940 Glyma12g12940 Glyma12g12940 Glyma12g12940 Glyma12g12940 Glyma05g25110 Glyma05g25110 Glyma05g25110 Glyma05g25110 Glyma05g25110 Glyma05g25110 Glyma08g41770 Glyma08g41770 Glyma08g41770 Glyma08g41770 Glyma08g41770 Glyma05g34920 Glyma05g34920 Glyma05g34920 Glyma09g03470 Glyma09g03470 Glyma09g03470 Glyma14g05070 Glyma14g05070 Glyma14g05070 Glyma14g05070 Glyma01g38310 Glyma19g09030 Glyma19g09030 Glyma19g09030 Glyma19g09030 Glyma19g09030 Glyma19g09030 Glyma19g09030 Glyma19g09030 Glyma19g09030 Glyma19g09030 Glyma19g09030 Glyma19g09030 Glyma01g09230 Glyma01g09230 Glyma01g09230 Glyma04g41950 Glyma04g41950 Glyma04g41950 Glyma04g41950 Glyma09g12510 Glyma09g12510 Glyma09g12510 Glyma09g12510 Glyma09g12510 Glyma09g12510 Glyma09g12510 Glyma09g12510 Glyma09g12510 Glyma09g12510 Glyma11g21200 Glyma11g21200 Glyma11g21200 Glyma11g21200 Glyma11g21200 Glyma04g01900 Glyma04g01900 Glyma04g01900 Glyma04g01900 Glyma04g01900 Glyma04g01900 Glyma04g01900 Glyma12g01370 Glyma12g01370 Glyma12g01370 Glyma12g01370 Glyma13g43640 Glyma13g43640 Glyma13g43640 Glyma13g43640 Glyma13g43640 Glyma13g43640 Glyma13g43640 Glyma13g43640 Glyma20g17500 Glyma20g17500 Glyma20g17500 Glyma20g17500 Glyma02g46090 Glyma02g46090 Glyma02g46090 Glyma02g46090 Glyma02g46090 Glyma02g46090 Glyma14g39970 Glyma14g39970 Glyma14g39970 Glyma14g39970 Glyma14g39970 Glyma14g39970 Glyma14g39970 Glyma13g24780 Glyma13g24780 Glyma13g24780 Glyma13g24780 Glyma13g24780 Glyma11g30040 Glyma11g30040 Glyma11g30040 Glyma11g30040 Glyma11g30040 Glyma11g30040 Glyma11g30040 Glyma11g30040 Glyma11g30040 Glyma11g30040 Glyma08g25580 Glyma08g25580 Glyma08g25580 Glyma08g25580 Glyma08g25580 Glyma08g25580 Glyma08g25580 Glyma18g10570 Glyma18g10570 Glyma18g10570 Glyma18g10570 Glyma18g10570 Glyma06g44170 Glyma06g44170 Glyma06g44170 Glyma06g44170 Glyma06g44170 Glyma06g44170 Glyma08g17760 Glyma08g17760 Glyma08g17760 Glyma13g26640 Glyma13g26640 Glyma17g02400 Glyma17g02400 Glyma17g02400 Glyma17g02400 Glyma17g02400 Glyma17g02400 Glyma17g09200 Glyma17g09200 Glyma17g09200 Glyma08g03220 Glyma08g03220 Glyma08g03220 Glyma11g10850 Glyma11g10850 Glyma11g10850 Glyma11g10850 Glyma11g10850 Glyma12g23390 Glyma12g23390 Glyma12g23390 Glyma12g23390 Glyma12g23390 Glyma09g34000 Glyma09g34000 Glyma09g34000 Glyma09g34000 Glyma09g34000 Glyma09g34000 Glyma16g03390 Glyma16g03390 Glyma16g03390 Glyma16g03390 Glyma16g03390 Glyma16g03390 Glyma18g25870 Glyma18g25870 Glyma18g25870 Glyma18g25870 Glyma08g45420 Glyma08g45420 Glyma08g45420 Glyma08g45420 Glyma18g32830 Glyma18g32830 Glyma18g32830 Glyma18g32830 Glyma19g32470 Glyma19g32470 Glyma19g32470 Glyma19g32470 Glyma19g32470 Glyma19g32470 Glyma19g32470 Glyma06g08740 Glyma07g02830 Glyma07g02830 Glyma07g02830 Glyma07g01030 Glyma07g01030 Glyma07g01030 Glyma07g01030 Glyma02g27240 Glyma02g27240 Glyma02g27240 Glyma02g27240 Glyma02g03560 Glyma02g03560 Glyma02g03560 Glyma02g03560 Glyma02g03560 Glyma15g20010 Glyma15g20010 Glyma19g25690 Glyma19g25690 Glyma19g25690 Glyma19g25690 Glyma19g25690 Glyma19g25690 Glyma19g25690 Glyma05g14670 Glyma05g14670 Glyma05g14670 Glyma19g40660 Glyma19g40660 Glyma19g40660 Glyma19g40660 Glyma19g40660 Glyma07g33060 Glyma07g33060 Glyma07g33060 Glyma07g33060 Glyma07g33060 Glyma18g02640 Glyma18g02640 Glyma18g02640 Glyma07g36130 Glyma07g36130 Glyma07g36130 Glyma07g36130 Glyma07g36130 Glyma17g14600 Glyma17g14600 Glyma17g14600 Glyma17g14600 Glyma02g45030 Glyma02g45030 Glyma02g45030 Glyma02g45030 Glyma02g45030 Glyma02g45030 Glyma11g17010 Glyma02g31560 Glyma02g31560 Glyma02g31560 Glyma02g31560 Glyma02g31560 Glyma02g31560 Glyma02g31560 Glyma13g36110 Glyma13g36110 Glyma13g36110 Glyma13g36110 Glyma13g36110 Glyma13g36110 Glyma01g45090 Glyma01g45090 Glyma01g45090 Glyma01g45090 Glyma01g45090 Glyma01g45090 Glyma01g45090 Glyma01g45090 Glyma04g14750 Glyma04g14750 Glyma04g14750 Glyma04g14750 Glyma04g14750 Glyma19g38960 Glyma19g38960 Glyma19g38960 Glyma17g32270 Glyma17g32270 Glyma17g32270 Glyma15g23680 Glyma15g23680 Glyma15g23680 Glyma15g23680 Glyma03g40380 Glyma03g40380 Glyma03g40380 Glyma09g01180 Glyma09g01180 Glyma09g01180 Glyma09g01180 Glyma09g01180 Glyma09g01180 Glyma09g01180 Glyma09g12220 Glyma09g12220 Glyma09g12220 Glyma09g12220 Glyma09g12220 Glyma18g29680 Glyma18g29680 Glyma18g29680 Glyma18g29680 Glyma18g29680 Glyma18g29680 Glyma18g29680 Glyma08g27730 Glyma08g27730 Glyma08g27730 Glyma08g27730 Glyma08g27730 Glyma08g27730 Glyma14g17110 Glyma14g17110 Glyma14g17110 Glyma14g17110 Glyma14g17110 Glyma14g17110 Glyma14g17110 Glyma14g17110 Glyma14g17110 Glyma14g17110 Glyma14g39710 Glyma14g39710 Glyma14g39710 Glyma14g39710 Glyma19g22860 Glyma19g22860 Glyma19g22860 Glyma19g22860 Glyma19g22860 Glyma17g34570 Glyma17g34570 Glyma17g34570 Glyma17g34570 Glyma06g18470 Glyma06g18470 Glyma06g18470 Glyma06g18470 Glyma06g18470 Glyma06g18470 Glyma06g18470 Glyma15g41300 Glyma15g41300 Glyma15g41300 Glyma15g41300 Glyma15g41300 Glyma07g01090 Glyma07g01090 Glyma07g01090 Glyma08g27890 Glyma08g27890 Glyma08g27890 Glyma08g25540 Glyma08g25540 Glyma08g25540 Glyma08g25540 Glyma08g25540 Glyma08g25540 Glyma08g25540 Glyma06g31610 Glyma06g31610 Glyma06g31610 Glyma06g31610 Glyma09g02000 Glyma09g02000 Glyma10g15260 Glyma10g15260 Glyma10g15260 Glyma10g15260 Glyma10g15260 Glyma10g15260 Glyma10g15260 Glyma10g15260 Glyma10g15260 Glyma17g07080 Glyma17g07080 Glyma17g07080 Glyma17g07080 Glyma17g07080 Glyma05g33560 Glyma05g33560 Glyma05g33560 Glyma05g33560 Glyma05g33560 Glyma05g33560 Glyma05g33560 Glyma05g33560 Glyma06g00670 Glyma06g00670 Glyma06g00670 Glyma06g00670 Glyma19g34990 Glyma19g34990 Glyma19g34990 Glyma19g34990 Glyma19g34990 Glyma19g34990 Glyma19g34990 Glyma07g05210 Glyma07g05210 Glyma07g05210 Glyma07g05210 Glyma18g34800 Glyma18g34800 Glyma18g34800 Glyma18g34800 Glyma18g34800 Glyma18g34800 Glyma18g34800 Glyma18g34800 Glyma06g19160 Glyma06g19160 Glyma06g19160 Glyma06g19160 Glyma13g16040 Glyma13g16040 Glyma13g16040 Glyma13g16040 Glyma05g34300 Glyma05g34300 Glyma05g34300 Glyma05g34300 Glyma05g34300 Glyma05g34300 Glyma05g34300 Glyma05g34300 Glyma05g34300 Glyma16g01920 Glyma16g01920 Glyma16g01920 Glyma16g01920 Glyma16g01920 Glyma16g01920 Glyma16g01920 Glyma01g31650 Glyma01g31650 Glyma01g31650 Glyma01g31650 Glyma18g15940 Glyma18g15940 Glyma18g15940 Glyma18g15940 Glyma18g15940 Glyma17g37430 Glyma17g37430 Glyma15g09610 Glyma15g09610 Glyma15g09610 Glyma15g09610 Glyma15g09610 Glyma15g09610 Glyma15g09610 Glyma04g11260 Glyma04g11260 Glyma04g11260 #number reads mapped uniquely = 4693603 #number reads mapped uniquely = 4693603 #number reads mapped uniquely = 4693603 #number reads mapped uniquely = 4693603 Glyma07g19710 Glyma07g19710 Glyma07g19710 Glyma09g02280 Glyma09g02280 Glyma09g02280 Glyma09g02280 Glyma09g02280 Glyma09g02280 Glyma09g02280 Glyma09g02280 Glyma09g02280 Glyma06g23450 Glyma06g23450 Glyma06g23450 Glyma20g36760 Glyma20g36760 Glyma20g36760 Glyma20g36760 Glyma20g36760 Glyma05g05130 Glyma05g05130 Glyma05g05130 Glyma05g05130 Glyma12g29380 Glyma12g29380 Glyma12g29380 Glyma12g29380 Glyma20g25440 Glyma20g25440 Glyma20g25440 Glyma20g25440 Glyma07g01400 Glyma07g01400 Glyma07g01400 Glyma07g01400 Glyma06g08790 Glyma06g08790 Glyma17g31960 Glyma17g31960 Glyma17g31960 Glyma17g31960 Glyma13g20760 Glyma13g20760 Glyma13g20760 Glyma13g20760 Glyma13g20760 Glyma07g09420 Glyma07g09420 Glyma07g09420 Glyma07g09420 Glyma07g09420 Glyma07g09420 Glyma05g28800 Glyma05g28800 Glyma05g28800 Glyma05g28800 Glyma05g28800 Glyma03g29360 Glyma03g29360 Glyma03g29360 Glyma03g29360 Glyma03g29360 Glyma03g29360 Glyma03g29360 Glyma05g28490 Glyma05g28490 Glyma05g28490 Glyma18g46410 Glyma18g46410 Glyma18g46410 Glyma18g46410 Glyma20g14940 Glyma20g14940 Glyma20g14940 Glyma20g14940 Glyma20g14940 Glyma20g14940 Glyma08g08850 Glyma08g08850 Glyma08g08850 Glyma08g08850 Glyma05g30810 Glyma05g30810 Glyma05g30810 Glyma05g30810 Glyma05g30810 Glyma05g30810 Glyma05g30810 Glyma05g30810 Glyma02g11150 Glyma02g11150 Glyma02g11150 Glyma02g11150 Glyma02g11150 Glyma18g33800 Glyma18g33800 Glyma18g33800 Glyma18g33800 Glyma18g33800 Glyma18g33800 Glyma18g33800 Glyma06g38420 Glyma06g38420 Glyma06g38420 Glyma06g38420 Glyma06g38420 Glyma06g38420 Glyma11g36800 Glyma11g36800 Glyma11g36800 Glyma11g36800 Glyma11g36800 Glyma15g07150 Glyma15g07150 Glyma15g07150 Glyma15g07150 Glyma15g07150 Glyma15g07150 Glyma19g25860 Glyma19g25860 Glyma03g34940 Glyma03g34940 Glyma03g34940 Glyma03g34940 Glyma03g34940 Glyma09g03270 Glyma15g36680 Glyma15g36680 Glyma15g36680 Glyma15g36680 Glyma15g36680 Glyma15g36680 Glyma03g01620 Glyma03g01620 Glyma03g01620 Glyma03g01620 Glyma03g01620 Glyma03g35370 Glyma03g35370 Glyma03g35370 Glyma03g35370 Glyma03g35370 Glyma03g35370 Glyma10g40360 Glyma10g40360 Glyma10g40360 Glyma10g40360 Glyma10g40360 Glyma10g40360 Glyma10g40360 Glyma09g30280 Glyma09g30280 Glyma09g30280 Glyma19g28440 Glyma19g28440 Glyma19g28440 Glyma19g28440 Glyma19g28440 Glyma19g28440 Glyma20g34790 Glyma20g34790 Glyma20g34790 Glyma20g34790 Glyma20g34790 Glyma20g34790 Glyma20g34790 Glyma20g34790 Glyma20g34790 Glyma12g01240 Glyma12g01240 Glyma12g01240 Glyma12g01240 Glyma12g01240 Glyma12g01240 Glyma15g06080 Glyma15g06080 Glyma17g23180 Glyma05g23560 Glyma05g23560 Glyma05g23560 Glyma05g23560 Glyma05g23560 Glyma05g23560 Glyma05g23560 Glyma05g23560 Glyma01g32460 Glyma01g32460 Glyma01g32460 Glyma01g32460 Glyma01g32460 Glyma10g14900 Glyma10g14900 Glyma10g14900 Glyma10g14900 Glyma10g14900 Glyma10g14900 Glyma10g14900 Glyma10g14900 Glyma04g09020 Glyma04g09020 Glyma18g08170 Glyma04g32270 Glyma04g32270 Glyma04g32270 Glyma02g07380 Glyma02g07380 Glyma02g07380 Glyma02g07380 Glyma02g07380 Glyma11g37590 Glyma11g37590 Glyma11g37590 Glyma11g37590 Glyma03g41770 Glyma03g41770 Glyma03g41770 Glyma03g41770 Glyma14g08050 Glyma0859s00200 Glyma0859s00200 Glyma0859s00200 Glyma0859s00200 Glyma0859s00200 Glyma20g01620 Glyma20g01620 Glyma20g01620 Glyma20g01620 Glyma09g21310 Glyma09g21310 Glyma09g21310 Glyma09g21310 Glyma09g21310 Glyma09g21310 Glyma12g29800 Glyma12g29800 Glyma12g29800 Glyma12g29800 Glyma13g23000 Glyma13g23000 Glyma13g23000 Glyma15g35740 Glyma15g35740 Glyma15g35740 Glyma15g35740 Glyma05g30900 Glyma05g30900 Glyma05g30900 Glyma05g30900 Glyma05g30900 Glyma05g30900 Glyma05g30900 Glyma11g30450 Glyma11g30450 Glyma11g30450 Glyma11g30450 Glyma11g30450 Glyma11g30450 Glyma19g27600 Glyma19g27600 Glyma19g27600 Glyma19g27600 Glyma19g27600 Glyma04g42680 Glyma04g42680 Glyma04g42680 Glyma04g42680 Glyma04g42680 Glyma04g42680 Glyma04g42680 Glyma14g09600 Glyma14g09600 Glyma12g30740 Glyma12g30740 Glyma17g27150 Glyma17g27150 Glyma17g27150 Glyma17g27150 Glyma17g27150 Glyma07g03020 Glyma07g03020 Glyma07g03020 Glyma07g03020 Glyma19g24490 Glyma19g24490 Glyma10g09470 Glyma10g09470 Glyma04g05610 Glyma04g05610 Glyma04g05610 Glyma04g05610 Glyma04g05610 Glyma04g05610 Glyma19g33700 Glyma19g33700 Glyma19g33700 Glyma07g19090 Glyma07g19090 Glyma07g19090 Glyma07g19090 Glyma07g00740 Glyma07g00740 Glyma07g00740 Glyma07g00740 Glyma07g00740 Glyma07g00740 Glyma07g00740 Glyma18g12710 Glyma18g12710 Glyma10g33320 Glyma10g33320 Glyma10g33320 Glyma10g33320 Glyma10g33320 Glyma20g13340 Glyma20g13340 Glyma20g13340 Glyma20g13340 Glyma20g13340 Glyma20g13340 Glyma20g13340 Glyma20g13340 Glyma20g13340 Glyma20g13340 Glyma20g13340 Glyma20g13340 Glyma06g25350 Glyma16g34170 Glyma16g34170 Glyma16g34170 Glyma16g34170 Glyma16g34170 Glyma02g39870 Glyma02g39870 Glyma05g28050 Glyma05g28050 Glyma05g28050 Glyma05g28050 Glyma05g28050 Glyma05g28050 Glyma05g28050 Glyma05g28050 Glyma05g28050 Glyma02g42540 Glyma02g42540 Glyma02g42540 Glyma02g42540 Glyma02g42540 Glyma02g42540 Glyma02g42540 Glyma10g43070 Glyma10g43070 Glyma10g43070 Glyma10g43070 Glyma10g01630 Glyma10g01630 Glyma10g01630 Glyma10g01630 Glyma10g01630 Glyma10g01630 Glyma12g22200 Glyma12g22200 Glyma12g22200 Glyma12g22200 Glyma19g32880 Glyma19g32880 Glyma19g32880 Glyma19g32880 Glyma19g37550 Glyma19g37550 Glyma19g37550 Glyma19g37550 Glyma19g37550 Glyma13g31010 Glyma13g31010 Glyma13g31010 Glyma13g31010 Glyma13g31010 Glyma13g31010 Glyma16g32750 Glyma16g32750 Glyma16g32750 Glyma03g38650 Glyma03g38650 Glyma03g38650 Glyma05g29600 Glyma05g29600 Glyma05g29600 Glyma05g29600 Glyma05g29600 Glyma05g29600 Glyma05g29600 Glyma05g29600 Glyma05g29600 Glyma05g29600 Glyma05g29600 Glyma18g38550 Glyma18g38550 Glyma18g38550 Glyma18g38550 Glyma18g38550 Glyma08g25150 Glyma08g25150 Glyma08g25150 Glyma08g25150 Glyma08g25150 Glyma08g25150 Glyma13g00410 Glyma13g00410 Glyma12g33650 Glyma12g33650 Glyma08g18620 Glyma08g18620 Glyma08g18620 Glyma20g12270 Glyma20g12270 Glyma20g12270 Glyma20g12270 Glyma20g12270 Glyma07g06140 Glyma07g06140 Glyma07g06140 Glyma18g34400 Glyma18g34400 Glyma18g34400 Glyma18g34400 Glyma18g34400 Glyma18g34400 Glyma18g34400 Glyma18g34400 Glyma18g34400 Glyma11g06530 Glyma11g06530 Glyma11g06530 Glyma14g40000 Glyma14g40000 Glyma14g40000 Glyma14g40000 Glyma13g31490 Glyma13g31490 Glyma13g31490 Glyma13g31490 Glyma13g31490 Glyma13g35000 Glyma13g35000 Glyma13g35000 Glyma13g35000 Glyma13g35000 Glyma13g35000 Glyma05g17080 Glyma05g17080 Glyma05g17080 Glyma05g17080 Glyma05g17080 Glyma05g17080 Glyma04g04650 Glyma04g04650 Glyma04g04650 Glyma05g14810 Glyma05g14810 Glyma05g14810 Glyma05g14810 Glyma10g30710 Glyma10g30710 Glyma10g30710 Glyma10g30710 Glyma10g30710 Glyma04g34390 Glyma04g34390 Glyma04g34390 Glyma16g32610 Glyma16g32610 Glyma16g32610 Glyma16g32610 Glyma16g32610 Glyma16g32610 Glyma16g32610 Glyma14g19000 Glyma14g19000 Glyma14g19000 Glyma14g19000 Glyma09g03160 Glyma09g03160 Glyma09g03160 Glyma09g03160 Glyma09g03160 Glyma09g03160 Glyma09g03160 Glyma09g03160 Glyma01g24970 Glyma01g24970 Glyma01g24970 Glyma01g24970 Glyma01g24970 Glyma01g24970 Glyma01g24970 Glyma14g40100 Glyma14g40100 Glyma14g40100 Glyma14g40100 Glyma14g40100 Glyma13g01130 Glyma13g01130 Glyma13g01130 Glyma13g01130 Glyma13g01130 Glyma03g02620 Glyma03g02620 Glyma03g02620 Glyma03g02620 Glyma03g02620 Glyma03g02620 Glyma03g04180 Glyma03g04180 Glyma06g22250 Glyma06g22250 Glyma06g22250 Glyma06g22250 Glyma06g22250 Glyma06g22250 Glyma05g24850 Glyma05g24850 Glyma17g10090 Glyma17g10090 Glyma17g10090 Glyma17g10090 Glyma17g10090 Glyma17g10090 Glyma17g10090 Glyma17g10090 Glyma17g10090 Glyma17g10090 Glyma17g10090 Glyma17g10090 Glyma17g10090 Glyma17g10090 Glyma18g35680 Glyma18g35680 Glyma18g35680 Glyma18g35680 Glyma18g35680 Glyma18g35680 Glyma18g35680 Glyma18g35680 Glyma18g35680 Glyma06g29730 Glyma06g29730 Glyma06g29730 Glyma18g01670 Glyma18g01670 Glyma18g01670 Glyma06g45690 Glyma06g45690 Glyma06g45690 Glyma06g45690 Glyma06g45690 Glyma06g45690 Glyma06g45690 Glyma06g45690 Glyma06g45690 Glyma14g34060 Glyma14g34060 Glyma14g34060 Glyma14g34060 Glyma19g39800 Glyma19g39800 Glyma19g39800 Glyma19g39800 Glyma09g36920 Glyma09g36920 Glyma09g36920 Glyma09g36920 Glyma09g36920 Glyma09g36920 Glyma09g36920 Glyma09g36920 Glyma09g36920 Glyma09g36920 Glyma09g36920 Glyma09g36920 Glyma14g00970 Glyma14g00970 Glyma14g00970 Glyma06g03670 Glyma06g03670 Glyma06g03670 Glyma06g03670 Glyma06g03670 Glyma02g29430 Glyma02g29430 Glyma02g29430 Glyma02g29430 Glyma02g29430 Glyma02g29430 Glyma02g29430 Glyma02g29430 Glyma02g29430 Glyma12g04800 Glyma12g04800 Glyma12g04800 Glyma06g36690 Glyma06g36690 Glyma06g36690 Glyma06g36690 Glyma05g16420 Glyma05g16420 Glyma05g16420 Glyma05g16420 Glyma10g42810 Glyma10g42810 Glyma10g42810 Glyma10g42810 Glyma10g42810 Glyma0021s00420 Glyma0021s00420 Glyma18g07290 Glyma18g07290 Glyma18g07290 Glyma18g07290 Glyma18g07290 Glyma18g07290 Glyma18g07290 Glyma18g07290 Glyma11g27120 Glyma11g27120 Glyma11g27120 Glyma11g27120 Glyma11g27120 Glyma11g27120 Glyma11g27120 Glyma11g27120 Glyma15g38140 Glyma15g38140 Glyma15g38140 Glyma15g38140 Glyma15g38140 Glyma01g08010 Glyma01g08010 Glyma01g08010 Glyma01g08010 Glyma01g08010 Glyma01g08010 Glyma03g15910 Glyma03g15910 Glyma03g15910 Glyma03g15910 Glyma03g15910 Glyma03g37540 Glyma03g37540 Glyma03g37540 Glyma16g29020 Glyma16g29020 Glyma16g29020 Glyma02g43710 Glyma02g43710 Glyma08g18730 Glyma08g18730 Glyma08g18730 Glyma08g18730 Glyma08g18730 Glyma08g18730 Glyma08g18730 Glyma13g25000 Glyma13g25000 Glyma13g25000 Glyma13g25000 Glyma13g25000 Glyma13g25000 Glyma13g25000 Glyma13g25000 Glyma13g25000 Glyma15g22280 Glyma15g22280 Glyma15g22280 Glyma15g05750 Glyma15g05750 Glyma15g05750 Glyma15g05750 Glyma15g05750 Glyma05g14350 Glyma05g14350 Glyma05g14350 Glyma05g14350 Glyma05g14350 Glyma05g14350 Glyma05g14350 Glyma05g14350 Glyma07g17280 Glyma07g17280 Glyma07g17280 Glyma07g17280 Glyma07g17280 Glyma07g17280 Glyma07g17280 Glyma07g17280 Glyma07g17280 Glyma07g17280 Glyma07g17280 Glyma04g12130 Glyma04g12130 Glyma04g12130 Glyma04g12130 Glyma08g28790 Glyma08g28790 Glyma08g28790 Glyma08g28790 Glyma12g01690 Glyma12g01690 Glyma12g01690 Glyma12g01690 Glyma12g01690 Glyma12g01690 Glyma04g38730 Glyma16g21450 Glyma16g21450 Glyma16g21450 Glyma18g14610 Glyma18g14610 Glyma18g14610 Glyma03g35300 Glyma03g35300 Glyma03g35300 Glyma07g39450 Glyma07g39450 Glyma07g39450 Glyma07g39450 Glyma10g25700 Glyma10g25700 Glyma10g25700 Glyma10g25700 Glyma03g22000 Glyma03g22000 Glyma03g22000 Glyma03g22000 Glyma15g17790 Glyma15g17790 Glyma15g17790 Glyma15g17790 Glyma15g17790 Glyma15g17790 Glyma02g19330 Glyma02g19330 Glyma02g19330 Glyma09g35000 Glyma09g35000 Glyma09g35000 Glyma09g35000 Glyma09g35000 Glyma09g35000 Glyma09g35000 Glyma09g35000 Glyma09g35000 Glyma09g35000 Glyma01g38420 Glyma01g38420 Glyma01g38420 Glyma07g09210 Glyma07g09210 Glyma07g09210 Glyma07g09210 Glyma07g09210

Genes with sequence AAAGAT

Module1

Glyma02g19870

Glyma06g09600

Glyma06g21850

Glyma07g07970

Glyma08g17240

Glyma11g36960

Glyma15g01520

Glyma17g13760

Glyma20g37370

Module2

Glyma02g45430

Glyma03g40130

Glyma05g24930

Glyma05g34680

Glyma05g36600

Glyma06g13870

Glyma10g29200

Glyma11g15090

Glyma12g04510

Glyma12g30800

Glyma12g32000

Glyma15g03120

Glyma16g05530

Glyma19g39940

Glyma20g22090

Glyma20g29190

Module3

Glyma02g40290

Glyma03g38190

Glyma06g04760

Glyma08g02940

Glyma08g11070

Glyma10g35520

Glyma11g33560

Glyma12g29510

Glyma19g32990

Module4

Glyma01g04420

Glyma01g32140

Glyma02g12020

Glyma03g29330

Glyma04g02380

Glyma04g02780

Glyma05g04500

Glyma06g02540

Glyma06g09970

Glyma06g39810

Glyma06g47560

Glyma07g18080

Glyma08g45610

Glyma09g30420

Glyma09g41460

Glyma10g42100

Glyma13g23250

Glyma14g05680

Glyma15g01100

Glyma15g02790

Glyma16g06640

Glyma18g06060

Module5

Glyma01g08540

Glyma02g02630

Glyma03g16440

Glyma05g36430

Glyma06g23570

Glyma08g22820

Glyma09g05200

Glyma10g29980

Glyma10g34710

Glyma11g04150

Glyma11g11430

Glyma14g05000

Glyma15g13700

Glyma18g50410

Module6

Glyma08g17010

Glyma15g43170

Glyma16g26630

Glyma20g02110

Module7

Glyma01g38520

Glyma03g29860

Glyma03g37280

Glyma09g38100

Glyma10g40670

Glyma11g34830

Glyma17g00230

Glyma18g52050

Module8

Glyma04g27810

Glyma07g05230

Glyma08g41120

Glyma09g36000

Glyma09g38990

Glyma09g39230

Glyma10g26690

Module9

Glyma04g05690

Glyma06g14640

Glyma06g43060

Glyma07g05820

Glyma09g17130

Glyma12g04890

Glyma12g15170

Glyma13g44870

Glyma14g06650

Glyma16g06410

Glyma20g30140

Module10

Glyma01g03820

Glyma02g15780

Glyma04g04240

Glyma04g37220

Glyma05g30690

Glyma05g37680

Glyma07g25390

Glyma08g41650

Glyma13g27060

Glyma13g35550

Glyma15g18070

Glyma15g24130

Glyma17g11330

Module12

Glyma12g34550

Glyma15g12170

Glyma17g34870

Module13

Glyma02g13520

Glyma02g37510

Glyma03g01850

Glyma03g37510

Glyma04g40030

Glyma05g29400

Glyma05g36350

Glyma06g11380

Glyma06g12260

Glyma08g19090

Glyma10g01220

Glyma11g04420

Glyma11g11510

Glyma12g04790

Glyma13g04940

Glyma13g23920

Glyma13g26470

Glyma13g34340

Glyma13g44390

Glyma15g02860

Glyma17g21590

Glyma18g01140

Glyma20g15460

Glyma20g18290

Glyma20g30100

Module14

Glyma01g45290

Glyma06g09420

Glyma07g38940

Glyma07g39020

Glyma10g40460

Glyma12g08040

Glyma13g23170

Module15

Glyma02g34670

Glyma04g33010

Glyma08g46610

Glyma08g47620

Glyma12g11210

Glyma14g26700

Module16

Glyma06g18770

Glyma08g46690

Glyma09g34830

Glyma11g07140

Glyma12g35940

Glyma16g05950

Glyma17g04640

Glyma17g15090

Glyma20g01690

Glyma20g01830

Module17

Glyma01g02660

Glyma02g10470

Glyma02g43500

Glyma03g31460

Glyma04g42830

Glyma05g29580

Glyma06g10930

Glyma06g11680

Glyma07g14460

Glyma08g05930

Glyma08g09090

Glyma09g06930

Glyma12g35730

Glyma13g22800

Glyma14g02940

Glyma15g05410

Glyma15g34720

Glyma16g03020

Glyma16g22530

Glyma18g45420

Glyma20g24850

Module18

Glyma02g12980

Glyma04g08070

Glyma09g04340

Glyma09g04630

Glyma11g37360

Glyma14g09510

Glyma18g01330

Glyma20g30910

Module19

Glyma04g03980

Glyma04g40170

Glyma06g41220

Glyma08g00320

Glyma08g17230

Glyma10g28610

Glyma15g30110

Glyma19g25980

Module20

Glyma07g15960

Glyma09g33160

Glyma15g01950

Glyma20g08800

Module21

Glyma02g44350

Glyma04g11290

Glyma05g30140

Glyma06g13020

Glyma07g32020

Glyma13g44170

Glyma19g01300

Glyma19g44160

Module22

Glyma01g26840

Glyma07g32330

Glyma07g32340

Glyma07g38110

Glyma12g06100

Module23

Glyma02g15190

Glyma02g42080

Glyma04g00660

Glyma05g00400

Glyma05g21820

Glyma08g24000

Glyma13g41960

Glyma14g07150

Glyma17g17850

Glyma17g38140

Glyma18g02210

Glyma18g47780

Glyma18g48620

Glyma18g52860

Module24

Glyma02g10570

Glyma03g02060

Glyma03g36890

Glyma04g08900

Glyma06g12440

Glyma06g13630

Glyma06g45990

Glyma06g48330

Glyma07g12190

Glyma07g37820

Glyma08g00510

Glyma08g17650

Glyma08g47690

Glyma10g41070

Glyma11g18710

Glyma12g00350

Glyma12g02440

Glyma12g10750

Glyma13g08480

Glyma14g04020

Glyma14g06010

Glyma16g00530

Glyma17g35770

Glyma18g48290

Glyma20g24480

Glyma20g25630

Module25

Glyma02g15370

Glyma03g03670

Glyma03g04450

Glyma03g39580

Glyma04g05550

Glyma05g28810

Glyma06g13910

Glyma08g19430

Glyma08g20330

Glyma12g34410

Glyma12g35000

Glyma13g27530

Glyma13g37830

Glyma16g08020

Glyma17g36150

Glyma18g05710

Glyma20g22290

Glyma20g34980

Module26

Glyma01g03530

Glyma02g32100

Glyma03g39300

Glyma05g09400

Glyma06g08200

Glyma06g47280

Glyma08g02960

Glyma08g12050

Glyma09g00280

Glyma09g04280

Glyma12g01050

Glyma12g07130

Glyma12g09600

Glyma13g22970

Glyma15g03020

Glyma16g04810

Glyma17g11620

Glyma18g49070

Glyma19g30540

Module27

Glyma04g07540

Glyma05g23100

Glyma06g25310

Glyma08g04540

Glyma08g07990

Glyma09g22270

Glyma10g32660

Glyma10g40620

Glyma11g08350

Glyma13g44830

Glyma14g02130

Glyma17g33050

Glyma19g30690

Glyma20g26350

Glyma20g29750

Module28

Glyma01g45740

Glyma05g01270

Glyma05g28420

Glyma05g30790

Glyma05g35880

Glyma06g15150

Glyma07g02260

Glyma07g02640

Glyma08g02140

Glyma08g06200

Glyma09g38420

Glyma10g42940

Glyma11g02900

Glyma11g08550

Glyma11g29430

Glyma13g00560

Glyma13g19640

Glyma13g23060

Glyma13g33960

Glyma13g34670

Glyma16g07150

Glyma17g16030

Glyma17g23830

Glyma19g05780

Glyma20g22400

Module29

Glyma01g42800

Glyma02g46670

Glyma03g05530

Glyma03g28080

Glyma06g05530

Glyma06g10740

Glyma06g45910

Glyma07g01960

Glyma07g15690

Glyma08g12650

Glyma08g20190

Glyma08g47200

Glyma08g48240

Glyma10g15130

Glyma10g32820

Glyma11g12790

Glyma12g34310

Glyma13g17750

Glyma13g20260

Glyma13g27130

Glyma13g39710

Glyma16g01490

Glyma16g33720

Glyma18g52410

Glyma19g01200

Glyma19g40460

Module30

Glyma10g01690

Glyma15g40890

Glyma17g21540

Glyma18g05160

Glyma18g51100

Glyma19g39690

Module31

Glyma03g40660

Glyma03g41110

Glyma04g01590

Glyma05g26960

Glyma05g38070

Glyma07g39240

Glyma08g20900

Glyma09g36540

Glyma10g40140

Glyma11g03510

Glyma11g35110

Glyma12g22360

Glyma14g03780

Glyma14g33330

Glyma15g10650

Module32

Glyma03g37250

Glyma03g40860

Glyma04g37030

Glyma05g28060

Glyma05g28080

Glyma08g06010

Glyma10g22720

Glyma13g33190

Glyma14g33210

Glyma14g36610

Glyma14g38620

Glyma14g39160

Glyma16g04580

Glyma16g23230

Glyma18g03390

Glyma18g19420

Glyma18g47950

Glyma19g38600

Module33

Glyma03g25790

Glyma03g32980

Glyma03g36470

Glyma04g09820

Glyma04g40430

Glyma05g03880

Glyma05g36420

Glyma06g05410

Glyma06g14080

Glyma08g03150

Glyma08g09200

Glyma08g22600

Glyma10g40150

Glyma13g33410

Glyma16g23730

Module34

Glyma03g24020

Glyma06g11580

Glyma06g18390

Glyma08g27720

Glyma13g42620

Glyma14g37980

Glyma20g26420

Glyma20g26600

Module35

Glyma09g08100

Glyma13g40100

Glyma16g04190

Glyma17g35720

Glyma20g27950

Module36

Glyma07g00900

Module37

Glyma01g44290

Glyma03g16510

Glyma03g40680

Glyma04g41090

Glyma07g30880

Glyma09g12320

Glyma09g14380

Glyma11g29350

Glyma13g19830

Glyma17g07190

Glyma18g17350

Module38

Glyma01g42450

Glyma04g00450

Glyma07g38460

Glyma08g45810

Glyma09g32850

Glyma11g05900

Glyma13g21050

Module39

Glyma01g34700

Glyma04g41310

Glyma15g17410

Glyma16g07110

Glyma19g28480

Module40

Glyma02g45530

Glyma03g38130

Glyma05g02950

Glyma10g28510

Glyma16g32440

Module41

Glyma03g04990

Glyma03g15130

Glyma03g19260

Glyma05g32160

Glyma09g22310

Glyma14g35340

Glyma15g06790

Module42

Glyma02g44460

Glyma03g34700

Glyma03g34830

Glyma04g40980

Glyma07g05220

Glyma07g33800

Glyma08g22550

Glyma09g34310

Glyma09g34760

Glyma09g38530

Glyma13g31650

Glyma13g31900

Glyma14g40120

Glyma19g42720

Module43

Glyma08g18080

Glyma12g09730

Glyma13g24360

Glyma14g10890

Glyma16g27350

Module44

Glyma03g38990

Glyma04g01380

Glyma09g01320

Glyma20g27280

Module45

Glyma03g30070

Glyma05g32220

Glyma06g09500

Glyma06g12090

Glyma08g05710

Glyma08g15480

Glyma08g23990

Glyma09g01800

Glyma09g29960

Glyma13g04050

Glyma13g29020

Glyma15g42940

Glyma17g00710

Glyma17g04830

Glyma18g47730

Glyma19g22730

Glyma19g35780

Module46

Glyma08g01410

Glyma12g07260

Glyma14g36900

Glyma16g04730

Glyma16g26070

Glyma17g35290

Glyma18g07330

Glyma20g05530

Module47

Glyma09g21820

Glyma10g07500

Glyma17g06220

Module48

Glyma01g04630

Glyma03g13310

Glyma05g04530

Glyma05g27360

Glyma06g19630

Glyma07g15420

Glyma10g41230

Glyma12g03740

Glyma16g27060

Glyma18g19360

Module49

Glyma08g06470

Glyma09g06250

Glyma13g19500

Glyma13g34520

Glyma17g10490

Glyma19g02370

Module50

Glyma01g20980

Glyma01g42230

Glyma02g17060

Glyma03g29190

Glyma04g05510

Glyma05g05420

Glyma05g25460

Glyma05g29380

Glyma05g33010

Glyma06g44450

Glyma07g11720

Glyma07g16810

Glyma08g05000

Glyma08g12290

Glyma08g19180

Glyma08g44780

Glyma10g08120

Glyma10g31280

Glyma11g02640

Glyma11g09250

Glyma11g31330

Glyma12g05790

Glyma12g10850

Glyma12g35370

Glyma13g22350

Glyma13g30330

Glyma13g31610

Glyma13g43140

Glyma15g35410

Glyma16g03570

Glyma16g31280

Glyma18g52950

Glyma19g28950

Module51

Glyma02g11540

Glyma03g40280

Glyma04g11400

Glyma04g40470

Glyma06g02330

Glyma09g02240

Glyma10g04560

Glyma13g06390

Glyma14g06630

Glyma16g24120

Glyma18g08220

Module52

Glyma15g23470

Module53

Glyma19g37230

Module54

Glyma01g42370

Glyma03g29950

Glyma04g02230

Glyma05g30290

Glyma07g37270

Glyma08g24720

Glyma09g21010

Glyma09g32630

Glyma10g02090

Glyma11g03310

Glyma12g02040

Glyma12g02240

Glyma12g06300

Glyma13g42140

Glyma15g06780

Glyma15g35390

Glyma16g33790

Glyma17g15690

Glyma19g45260

Glyma20g38590

Module55

Glyma04g33570

Glyma07g02400

Glyma08g46110

Glyma11g02530

Glyma15g15370

Glyma15g15990

Glyma17g02000

Glyma19g28220

Glyma20g29990

Module56

Glyma02g12950

Glyma02g40270

Glyma03g41460

Glyma06g06170

Glyma06g19600

Glyma06g19960

Glyma07g31330

Glyma07g35540

Glyma08g47960

Glyma09g24910

Glyma09g38750

Glyma10g02030

Glyma10g41610

Glyma11g17490

Glyma13g03160

Glyma13g17290

Glyma13g32290

Glyma15g16650

Glyma20g26960

Glyma20g30430

Glyma20g34460

Module57

Glyma01g43880

Glyma05g22180

Glyma08g47750

Glyma09g28490

Glyma10g38070

Glyma11g07490

Glyma16g28590

Module58

Glyma02g46380

Glyma04g08200

Glyma04g38640

Glyma04g41480

Glyma06g01930

Glyma07g15170

Glyma07g30470

Glyma07g34340

Glyma08g11800

Glyma08g23550

Glyma09g02040

Glyma09g05180

Glyma10g40250

Glyma11g14980

Glyma11g31450

Glyma15g13470

Glyma15g41680

Glyma16g26600

Glyma17g05290

Glyma17g35610

Glyma20g30660

Module59

Glyma01g03570

Glyma01g41810

Glyma03g37340

Glyma05g02570

Glyma06g06790

Glyma07g00330

Glyma08g17600

Glyma08g18860

Glyma10g00890

Glyma17g00820

Glyma19g27380

Glyma19g28850

Glyma20g38970

Module60

Glyma01g03470

Glyma02g15420

Glyma19g38800

Module61

Glyma05g24110

Glyma05g29000

Glyma06g19820

Glyma08g12140

Glyma08g18110

Glyma08g29090

Glyma11g11290

Glyma12g03470

Glyma19g29180

Module62

Glyma03g13520

Glyma04g43340

Glyma05g01680

Glyma06g03200

Glyma08g11500

Glyma09g32790

Glyma11g37190

Glyma18g06420

Glyma20g01550

Glyma20g02490

Module63

Glyma01g44090

Glyma02g44710

Glyma03g31800

Glyma04g36090

Glyma04g39680

Glyma04g40740

Glyma06g01030

Glyma07g03000

Glyma07g29720

Glyma07g33130

Glyma07g40330

Glyma08g07860

Glyma08g10160

Glyma08g10330

Glyma08g23250

Glyma09g38560

Glyma13g21870

Glyma13g38420

Glyma17g09250

Glyma18g47760

Module64

Glyma08g16380

Glyma10g39980

Glyma13g38490

Module65

Glyma01g01310

Glyma02g39090

Glyma03g28850

Glyma07g02500

Glyma09g04940

Glyma10g31630

Glyma12g03570

Glyma12g10960

Glyma13g17420

Glyma14g06160

Module66

Glyma04g41750

Glyma07g37100

Glyma10g30440

Glyma11g12720

Glyma11g14050

Glyma14g36540

Glyma16g06850

Glyma18g03340

Glyma18g36750

Glyma18g50240

Glyma20g28860

Module67

Glyma01g38040

Glyma03g40210

Glyma03g40220

Glyma04g02070

Glyma05g00980

Glyma06g03670

Glyma07g11220

Glyma08g08170

Glyma08g17270

Glyma08g24680

Glyma08g44700

Glyma09g15200

Glyma09g30380

Glyma09g31450

Glyma13g23770

Glyma14g03890

Glyma15g17420

Glyma16g01770

Glyma16g06530

Glyma17g02080

Glyma17g07440

Glyma18g48910

Glyma19g03450

Glyma20g03850

Glyma20g33290

Glyma20g34830

Module68

Glyma05g21030

Glyma07g04810

Glyma07g35640

Glyma11g12000

Glyma12g02260

Glyma12g27400

Glyma12g34440

Glyma14g04940

Glyma14g37350

Glyma16g05560

Glyma17g33040

Glyma18g40750

Glyma19g44270

Module69

Glyma01g02950

Glyma02g00340

Glyma06g03100

Glyma06g04640

Glyma06g12640

Glyma07g22970

Glyma09g33770

Glyma10g39460

Glyma12g32770

Glyma13g05120

Glyma15g18360

Glyma17g11940

Glyma18g00930

Module70

Glyma02g10580

Glyma03g30510

Glyma07g04450

Glyma08g07210

Glyma08g22170

Glyma08g39510

Glyma13g17650

Glyma13g27410

Glyma14g40170

Glyma15g16810

Glyma15g40790

Glyma17g16570

Glyma18g10930

Genes with sequence CTCTT

Module1

Glyma01g40830

Glyma02g11960

Glyma02g19870

Glyma05g07480

Glyma05g24230

Glyma06g48350

Glyma08g17240

Glyma08g26670

Glyma10g30020

Glyma10g35890

Glyma10g42480

Glyma11g14840

Glyma12g04300

Glyma14g03970

Glyma14g04520

Glyma15g14970

Glyma17g08970

Glyma18g52590

Glyma20g24540

Glyma20g38980

Module2

Glyma03g27570

Glyma03g29810

Glyma03g40130

Glyma04g37770

Glyma06g17310

Glyma06g46180

Glyma06g47520

Glyma07g06830

Glyma08g02630

Glyma08g04990

Glyma10g01060

Glyma11g12080

Glyma11g37250

Glyma13g20310

Glyma17g07060

Glyma18g02820

Glyma18g26190

Glyma19g25930

Glyma19g39940

Glyma19g42090

Glyma20g29190

Module3

Glyma02g40290

Glyma03g38190

Glyma06g11080

Glyma08g02940

Glyma09g02790

Glyma10g06600

Glyma11g15680

Glyma12g29510

Glyma14g00720

Glyma16g33710

Module4

Glyma01g04420

Glyma01g32140

Glyma01g32750

Glyma02g01990

Glyma02g11720

Glyma03g29330

Glyma04g02380

Glyma04g02780

Glyma05g03580

Glyma06g01990

Glyma06g03290

Glyma06g08170

Glyma06g09080

Glyma06g09970

Glyma06g47560

Glyma07g09840

Glyma08g11100

Glyma08g25090

Glyma08g45610

Glyma09g01750

Glyma09g30420

Glyma09g41460

Glyma10g31780

Glyma11g15020

Glyma11g18810

Glyma11g37620

Glyma12g06310

Glyma12g08160

Glyma12g09660

Glyma13g39230

Glyma13g43970

Glyma14g01960

Glyma15g01370

Glyma15g01500

Glyma15g13460

Glyma15g15690

Glyma16g06640

Glyma19g07080

Glyma19g41730

Glyma20g24930

Glyma20g32990

Module5

Glyma01g08540

Glyma01g32450

Glyma03g00410

Glyma03g16440

Glyma04g35580

Glyma04g43270

Glyma05g08530

Glyma05g08970

Glyma05g24130

Glyma06g23570

Glyma09g05200

Glyma10g29980

Glyma10g34710

Glyma11g04150

Glyma12g30430

Glyma15g24060

Glyma16g05700

Glyma16g26640

Glyma18g50410

Glyma19g31420

Glyma20g20280

Glyma20g26770

Glyma20g34920

Module6

Glyma01g03650

Glyma07g39160

Glyma11g00710

Glyma11g21480

Glyma13g41700

Glyma13g41710

Glyma13g42150

Glyma15g03710

Module7

Glyma01g33650

Glyma03g25520

Glyma03g29860

Glyma03g37280

Glyma08g48040

Glyma10g26790

Glyma10g40670

Glyma12g29340

Glyma12g32390

Glyma15g16630

Glyma17g00230

Glyma17g26600

Glyma18g13270

Glyma18g52050

Glyma20g04230

Module8

Glyma08g11960

Glyma08g41120

Glyma09g38990

Glyma09g39230

Glyma10g26690

Glyma10g43760

Glyma12g06540

Glyma16g29330

Glyma18g44830

Glyma20g00760

Module9

Glyma03g28490

Glyma04g05690

Glyma04g28560

Glyma04g42740

Glyma06g36590

Glyma07g05820

Glyma12g04890

Glyma14g05850

Glyma14g06640

Glyma14g06650

Glyma14g26660

Glyma14g39050

Glyma15g43200

Glyma16g06410

Glyma16g29790

Glyma18g50180

Module10

Glyma03g24180

Glyma04g35710

Glyma04g37220

Glyma05g25700

Glyma05g30690

Glyma06g21560

Glyma07g05390

Glyma07g16850

Glyma07g37790

Glyma08g05200

Glyma08g08550

Glyma08g36660

Glyma09g02590

Glyma13g40470

Glyma15g24130

Glyma16g02490

Glyma16g03960

Glyma17g11330

Glyma17g15860

Glyma17g36950

Glyma20g07060

Glyma20g32140

Module12

Glyma10g35870

Glyma15g12170

Module13

Glyma01g35110

Glyma01g43480

Glyma02g01210

Glyma02g48100

Glyma03g01850

Glyma03g29080

Glyma03g40710

Glyma04g02820

Glyma04g02900

Glyma04g07980

Glyma04g26440

Glyma04g40030

Glyma06g11380

Glyma06g12260

Glyma07g02430

Glyma08g10890

Glyma09g29480

Glyma09g30230

Glyma10g04770

Glyma11g12090

Glyma11g37720

Glyma13g04940

Glyma13g23920

Glyma13g26470

Glyma14g35160

Glyma15g02860

Glyma15g05060

Glyma15g05730

Glyma17g36400

Glyma18g10400

Glyma18g49060

Glyma19g34120

Glyma19g38130

Glyma20g01640

Glyma20g38430

Module14

Glyma01g02400

Glyma01g45290

Glyma03g40760

Glyma04g00960

Glyma04g03020

Glyma04g39380

Glyma05g27190

Glyma06g09420

Glyma07g39020

Glyma13g23170

Module15

Glyma01g04380

Glyma02g07700

Glyma02g46600

Glyma04g38710

Glyma05g00930

Glyma07g05620

Glyma07g38580

Glyma08g46610

Glyma08g47620

Glyma09g12860

Glyma10g08300

Glyma10g31020

Glyma13g41540

Glyma14g17060

Glyma14g35660

Glyma17g13530

Glyma18g44250

Glyma19g42200

Glyma20g31040

Module16

Glyma01g26650

Glyma02g04590

Glyma03g37710

Glyma06g00400

Glyma06g18770

Glyma07g00730

Glyma09g04560

Glyma09g21300

Glyma09g34830

Glyma09g38070

Glyma10g07240

Glyma11g04240

Glyma11g36790

Glyma12g35940

Glyma14g39590

Glyma20g01690

Glyma20g01730

Module17

Glyma01g02660

Glyma02g43640

Glyma03g31460

Glyma03g40250

Glyma04g08570

Glyma05g29580

Glyma05g37790

Glyma06g11680

Glyma07g14460

Glyma07g16970

Glyma07g37430

Glyma09g03560

Glyma09g06930

Glyma09g40420

Glyma12g35730

Glyma14g17930

Glyma15g06890

Glyma15g10020

Glyma16g02090

Glyma16g03020

Glyma18g45420

Glyma20g10260

Glyma20g22850

Glyma20g33270

Module18

Glyma03g29440

Glyma04g08070

Glyma07g03910

Glyma08g06420

Glyma09g04340

Glyma09g04530

Glyma09g04630

Glyma10g39450

Glyma12g06950

Glyma13g06230

Glyma13g42340

Glyma16g27880

Glyma17g14850

Glyma20g30910

Module19

Glyma01g00770

Glyma02g15150

Glyma04g40170

Glyma06g41220

Glyma08g00320

Glyma12g04940

Glyma12g08520

Glyma15g13500

Glyma15g41970

Glyma16g01960

Glyma18g45260

Glyma19g33730

Module20

Glyma04g04540

Glyma07g15960

Glyma08g03290

Glyma09g33160

Glyma11g13070

Glyma15g01950

Module21

Glyma04g07220

Glyma06g13020

Glyma08g14130

Glyma09g24890

Glyma13g28240

Glyma15g10710

Glyma15g13970

Glyma16g20780

Glyma17g23860

Glyma18g04500

Glyma20g29660

Glyma0169s00210

Module22

Glyma07g32330

Glyma07g32340

Glyma08g38740

Glyma11g14140

Glyma17g14620

Glyma20g35630

Module23

Glyma01g36850

Glyma02g42080

Glyma04g00660

Glyma05g00400

Glyma07g15320

Glyma08g24000

Glyma09g32540

Glyma09g38590

Glyma11g08440

Glyma11g33720

Glyma13g16500

Glyma14g07150

Glyma18g47780

Glyma18g48620

Glyma18g52860

Glyma20g28440

Module24

Glyma02g05550

Glyma02g43410

Glyma03g02060

Glyma03g36890

Glyma04g34720

Glyma06g12440

Glyma06g13630

Glyma07g08650

Glyma07g12190

Glyma07g30030

Glyma07g37820

Glyma08g19520

Glyma08g47690

Glyma09g18050

Glyma10g34750

Glyma11g14150

Glyma12g00350

Glyma12g01430

Glyma13g02870

Glyma13g08480

Glyma14g04020

Glyma15g40170

Glyma16g00530

Glyma16g27430

Glyma17g33020

Glyma17g35580

Glyma18g48290

Glyma20g24480

Glyma20g38710

Module25

Glyma02g26890

Glyma03g03670

Glyma03g04450

Glyma06g10970

Glyma06g13910

Glyma08g19430

Glyma08g20330

Glyma09g27320

Glyma09g41120

Glyma12g34410

Glyma12g35000

Glyma13g27530

Glyma13g42420

Glyma14g01440

Glyma14g09070

Glyma14g09080

Glyma16g08020

Glyma17g36150

Glyma18g53860

Module26

Glyma01g01950

Glyma01g03530

Glyma02g47690

Glyma03g32150

Glyma06g47280

Glyma07g08690

Glyma08g11980

Glyma09g04280

Glyma10g04590

Glyma12g07130

Glyma13g22970

Glyma13g28840

Glyma14g24270

Glyma15g01210

Glyma15g24650

Glyma15g37200

Glyma19g22310

Glyma20g31150

Module27

Glyma02g03160

Glyma02g08970

Glyma02g11750

Glyma04g07540

Glyma05g35120

Glyma06g17320

Glyma06g25310

Glyma08g01500

Glyma08g07990

Glyma09g38310

Glyma10g32660

Glyma10g43890

Glyma11g08350

Glyma13g41830

Glyma13g44830

Glyma16g18030

Glyma16g34180

Glyma17g02380

Glyma17g10810

Glyma18g46420

Glyma19g02540

Glyma19g39370

Glyma20g38290

Module28

Glyma01g20760

Glyma02g46340

Glyma05g01270

Glyma05g35880

Glyma06g06390

Glyma07g02260

Glyma07g02640

Glyma08g24950

Glyma09g34000

Glyma09g38890

Glyma10g42940

Glyma11g12480

Glyma11g14190

Glyma12g28880

Glyma13g00560

Glyma15g12760

Glyma16g04930

Glyma16g30190

Glyma17g23830

Glyma18g05730

Glyma19g05780

Glyma19g28430

Glyma20g20900

Glyma20g23690

Module29

Glyma01g39460

Glyma01g42660

Glyma01g45110

Glyma02g15020

Glyma02g36580

Glyma02g40890

Glyma03g02580

Glyma03g21540

Glyma03g28080

Glyma03g35700

Glyma04g08990

Glyma04g10900

Glyma04g33940

Glyma05g04400

Glyma05g19620

Glyma06g02290

Glyma06g03180

Glyma06g05530

Glyma06g10740

Glyma06g10750

Glyma06g45910

Glyma07g00870

Glyma07g09710

Glyma08g09870

Glyma08g12650

Glyma08g20190

Glyma08g45520

Glyma08g47200

Glyma09g02190

Glyma09g04850

Glyma09g05340

Glyma09g35840

Glyma09g37910

Glyma10g30110

Glyma10g32820

Glyma10g38550

Glyma11g12790

Glyma11g27720

Glyma12g04850

Glyma12g30050

Glyma13g03600

Glyma13g20260

Glyma13g35320

Glyma13g38130

Glyma13g39710

Glyma14g08070

Glyma15g10630

Glyma15g11700

Glyma15g13880

Glyma16g01780

Glyma16g06500

Glyma16g26940

Glyma16g27900

Glyma17g19790

Glyma18g46560

Glyma19g01200

Glyma19g35190

Glyma19g35270

Glyma19g38390

Glyma19g44060

Module30

Glyma02g42250

Glyma03g42060

Glyma10g01690

Glyma16g04440

Glyma16g06740

Glyma17g21540

Glyma19g39690

Glyma20g29200

Module31

Glyma01g35130

Glyma04g01590

Glyma05g01690

Glyma05g13110

Glyma05g26960

Glyma05g30430

Glyma05g38070

Glyma08g06860

Glyma08g20900

Glyma08g42380

Glyma09g07540

Glyma09g36540

Glyma10g02400

Glyma10g21950

Glyma10g40140

Glyma11g10150

Glyma14g01170

Glyma14g03780

Glyma14g10520

Glyma14g33330

Glyma15g10650

Glyma19g05550

Glyma19g44940

Glyma20g29960

Module32

Glyma02g40820

Glyma03g28760

Glyma03g38730

Glyma05g26530

Glyma05g28730

Glyma06g47890

Glyma08g01990

Glyma09g03020

Glyma10g02290

Glyma10g24590

Glyma11g12120

Glyma11g34980

Glyma12g31850

Glyma14g33210

Glyma14g39160

Glyma16g04580

Glyma16g27210

Glyma18g03390

Glyma19g25870

Glyma19g37840

Glyma19g39860

Module33

Glyma01g00740

Glyma01g26950

Glyma02g10170

Glyma02g38450

Glyma02g43790

Glyma03g25790

Glyma03g32980

Glyma04g06700

Glyma04g36860

Glyma04g42690

Glyma05g34570

Glyma06g07320

Glyma08g03150

Glyma08g03480

Glyma08g05610

Glyma08g09200

Glyma10g40150

Glyma12g08990

Glyma14g09300

Glyma14g35410

Glyma14g36970

Glyma16g23730

Glyma18g14980

Glyma19g37520

Module34

Glyma03g24020

Glyma05g34760

Glyma06g11580

Glyma06g42960

Glyma08g03240

Glyma08g13900

Glyma08g16580

Glyma10g35380

Glyma13g42620

Glyma14g37980

Glyma17g11340

Glyma20g26600

Module35

Glyma02g42220

Glyma04g01130

Glyma09g08100

Glyma13g40100

Glyma15g19580

Glyma17g37400

Glyma20g27940

Module36

Glyma05g37730

Glyma11g03690

Glyma12g00390

Module37

Glyma01g07070

Glyma04g03110

Glyma05g01390

Glyma08g47790

Glyma09g12320

Glyma12g10240

Glyma13g06050

Glyma13g39600

Glyma15g02700

Glyma17g07190

Glyma18g50760

Glyma19g30600

Module38

Glyma02g47790

Glyma06g48360

Glyma07g38460

Glyma08g19290

Glyma08g45810

Glyma10g11620

Glyma16g01650

Glyma16g03410

Glyma16g09760

Glyma17g07070

Glyma17g17970

Glyma20g04490

Module39

Glyma03g39730

Glyma04g41310

Glyma05g03070

Glyma06g16280

Glyma07g35380

Glyma09g10010

Glyma12g34990

Glyma16g06080

Glyma18g02900

Glyma18g19710

Glyma18g49910

Glyma19g03010

Glyma19g28480

Module40

Glyma01g33170

Glyma03g38130

Glyma04g08170

Glyma09g08470

Glyma10g28510

Glyma12g31080

Glyma13g27010

Glyma19g40190

Module41

Glyma01g42670

Glyma02g08950

Glyma03g15130

Glyma08g12460

Glyma08g24750

Glyma10g32340

Glyma13g33890

Glyma14g35340

Glyma15g01470

Glyma15g06790

Glyma15g17530

Glyma16g33270

Glyma19g01940

Module42

Glyma02g44460

Glyma03g34700

Glyma07g01540

Glyma07g02720

Glyma07g13730

Glyma07g39980

Glyma09g01270

Glyma09g35650

Glyma10g34220

Glyma11g20630

Glyma11g36090

Glyma12g03230

Glyma13g21520

Glyma15g23220

Glyma16g01460

Glyma16g33230

Glyma17g03550

Glyma17g11430

Glyma18g01110

Glyma18g18050

Glyma20g09810

Module43

Glyma02g08260

Glyma08g18080

Glyma08g43330

Glyma08g44310

Glyma09g36720

Glyma10g29190

Glyma12g09730

Glyma13g24360

Glyma14g10620

Glyma15g40450

Glyma16g27350

Glyma19g28990

Module44

Glyma04g01380

Glyma05g31610

Glyma06g03050

Glyma09g01320

Glyma09g04950

Glyma13g22940

Glyma17g01720

Glyma18g05340

Glyma20g27280

Module45

Glyma02g33770

Glyma04g07300

Glyma06g12090

Glyma07g00540

Glyma07g10170

Glyma07g34900

Glyma08g05710

Glyma08g15480

Glyma08g39390

Glyma09g01800

Glyma11g02190

Glyma11g08020

Glyma11g19490

Glyma12g02870

Glyma12g19050

Glyma13g04440

Glyma13g29020

Glyma16g06100

Glyma17g00710

Glyma17g04830

Glyma18g06560

Glyma18g38010

Glyma18g46330

Glyma18g47420

Glyma18g47730

Glyma20g03020

Glyma20g34330

Module46

Glyma01g06640

Glyma01g38980

Glyma02g04760

Glyma03g13780

Glyma07g00550

Glyma08g01410

Glyma10g36690

Glyma11g25650

Glyma12g07260

Glyma13g08050

Glyma13g33830

Glyma13g43050

Glyma14g02350

Glyma14g09170

Glyma14g36900

Glyma15g20180

Glyma15g43180

Glyma16g04730

Glyma16g26070

Glyma18g07330

Glyma18g20800

Glyma20g33060

Module47

Glyma04g42120

Glyma08g48030

Glyma09g21820

Glyma10g07500

Glyma12g02590

Glyma17g06220

Module48

Glyma01g04630

Glyma03g13310

Glyma04g38520

Glyma05g04530

Glyma06g04510

Glyma06g19590

Glyma09g25200

Glyma09g32740

Glyma10g07930

Glyma13g28900

Glyma15g03720

Glyma18g03550

Glyma18g48060

Glyma18g53370

Glyma20g02800

Glyma20g24860

Module49

Glyma01g24950

Glyma02g11640

Glyma04g09350

Glyma04g34840

Glyma05g33190

Glyma07g16910

Glyma08g00790

Glyma09g06250

Glyma18g00590

Glyma18g02220

Glyma18g49400

Glyma19g02370

Module50

Glyma01g20980

Glyma01g34580

Glyma01g40820

Glyma01g42230

Glyma02g03190

Glyma02g05300

Glyma02g35210

Glyma02g37280

Glyma02g43580

Glyma03g37390

Glyma04g05510

Glyma05g25460

Glyma05g33010

Glyma05g37420

Glyma06g06930

Glyma06g11600

Glyma06g12920

Glyma06g17950

Glyma06g40620

Glyma06g44450

Glyma07g05920

Glyma07g11720

Glyma07g16810

Glyma07g17170

Glyma08g09990

Glyma08g12290

Glyma08g19180

Glyma08g20220

Glyma08g25520

Glyma09g02650

Glyma09g06350

Glyma09g31840

Glyma09g41570

Glyma10g08010

Glyma10g08120

Glyma10g31280

Glyma10g40350

Glyma11g27480

Glyma11g31330

Glyma12g00980

Glyma12g05320

Glyma12g10850

Glyma13g01900

Glyma13g22350

Glyma13g23760

Glyma13g30330

Glyma14g39210

Glyma15g01170

Glyma15g07710

Glyma15g13100

Glyma16g04980

Glyma16g06900

Glyma16g22620

Glyma16g22920

Glyma16g26970

Glyma16g31280

Glyma17g01470

Glyma17g16660

Glyma18g39500

Glyma18g52950

Glyma19g28520

Glyma19g40000

Glyma20g00590

Glyma20g01370

Module51

Glyma02g05540

Glyma02g09370

Glyma02g11540

Glyma03g40280

Glyma04g11400

Glyma05g01180

Glyma05g34070

Glyma09g24070

Glyma11g10480

Glyma13g19330

Glyma13g19470

Glyma17g16830

Module52

Glyma06g19330

Glyma07g07810

Glyma10g00580

Glyma11g02500

Glyma13g04610

Glyma13g41030

Glyma15g22220

Glyma15g23470

Glyma17g01590

Glyma19g40800

Module53

Glyma10g05800

Glyma15g07040

Glyma17g08900

Glyma19g37230

Glyma19g37240

Module54

Glyma01g31660

Glyma02g16800

Glyma02g40010

Glyma03g15800

Glyma03g29950

Glyma03g34560

Glyma03g36620

Glyma03g37400

Glyma05g30290

Glyma06g01270

Glyma06g12010

Glyma06g18560

Glyma06g47190

Glyma07g04340

Glyma07g37270

Glyma08g04370

Glyma08g43040

Glyma08g43550

Glyma09g00850

Glyma09g09430

Glyma09g23140

Glyma09g32630

Glyma10g03000

Glyma11g03310

Glyma11g10760

Glyma11g20700

Glyma11g29920

Glyma11g35560

Glyma12g02040

Glyma12g03050

Glyma12g06300

Glyma12g07350

Glyma13g25560

Glyma13g42140

Glyma15g02380

Glyma15g07700

Glyma15g13550

Glyma15g13870

Glyma15g14210

Glyma15g16710

Glyma15g25060

Glyma16g05710

Glyma17g03360

Glyma17g12160

Glyma17g15690

Glyma18g02870

Glyma18g06220

Glyma19g40960

Module55

Glyma01g27810

Glyma03g09100

Glyma03g30440

Glyma03g42070

Glyma04g33570

Glyma08g24380

Glyma08g26880

Glyma08g46110

Glyma10g05720

Glyma11g00210

Glyma11g02530

Glyma13g01950

Glyma13g24180

Glyma14g39880

Glyma15g03430

Glyma15g15990

Glyma16g29340

Glyma18g08530

Glyma18g53440

Glyma19g28220

Glyma20g29990

Module56

Glyma02g12460

Glyma02g40790

Glyma02g42150

Glyma03g41460

Glyma06g06170

Glyma06g19600

Glyma06g19960

Glyma07g19190

Glyma07g35540

Glyma08g07740

Glyma08g47960

Glyma09g03630

Glyma09g05590

Glyma09g24910

Glyma09g37800

Glyma10g01720

Glyma10g02030

Glyma10g34480

Glyma11g15890

Glyma13g00580

Glyma13g17290

Glyma13g44910

Glyma15g11840

Glyma15g16650

Glyma16g04090

Glyma17g36730

Glyma18g01910

Glyma18g49970

Glyma19g30530

Glyma20g30430

Glyma20g34460

Glyma20g38810

Module57

Glyma08g00780

Glyma09g28490

Glyma17g03910

Module58

Glyma02g46380

Glyma04g00770

Glyma04g06010

Glyma04g08730

Glyma04g09950

Glyma04g41480

Glyma06g15280

Glyma07g15170

Glyma07g30470

Glyma07g34340

Glyma08g11800

Glyma08g23550

Glyma09g02040

Glyma10g07950

Glyma10g35210

Glyma10g43110

Glyma10g43120

Glyma10g44070

Glyma11g17030

Glyma11g19780

Glyma11g37760

Glyma14g23990

Glyma17g05290

Glyma18g53810

Module59

Glyma01g41620

Glyma02g00540

Glyma03g37340

Glyma04g43540

Glyma05g30780

Glyma06g06790

Glyma06g46530

Glyma07g00700

Glyma07g06590

Glyma08g17600

Glyma08g18860

Glyma08g21960

Glyma10g36610

Glyma11g11020

Glyma11g15230

Glyma11g20570

Glyma12g04020

Glyma12g08050

Glyma19g06460

Glyma19g28850

Glyma19g40080

Module60

Glyma02g35190

Glyma03g36140

Glyma13g38710

Glyma14g05840

Glyma15g15010

Module61

Glyma06g19820

Glyma08g18110

Glyma12g03470

Glyma12g07780

Glyma17g23870

Glyma19g29180

Glyma19g35560

Module62

Glyma01g42200

Glyma02g02340

Glyma02g07610

Glyma02g35230

Glyma03g29280

Glyma03g34480

Glyma03g35920

Glyma04g05570

Glyma04g34020

Glyma04g35180

Glyma04g43340

Glyma05g01680

Glyma05g28560

Glyma06g03200

Glyma06g19430

Glyma06g20360

Glyma09g03750

Glyma09g32790

Glyma09g33700

Glyma09g37860

Glyma13g01080

Glyma13g05020

Glyma13g05830

Glyma13g09240

Glyma13g28380

Glyma15g02010

Glyma15g08520

Glyma17g05860

Glyma18g06420

Glyma18g50770

Glyma19g30360

Glyma19g32280

Glyma20g01550

Glyma20g02490

Glyma20g02500

Glyma20g32050

Module63

Glyma01g35650

Glyma02g44710

Glyma03g31800

Glyma03g39850

Glyma05g03940

Glyma05g37260

Glyma07g03000

Glyma07g29720

Glyma08g10330

Glyma08g20940

Glyma08g23250

Glyma09g38560

Glyma13g13260

Glyma13g38730

Glyma17g09250

Glyma18g01390

Glyma19g40390

Glyma19g45180

Glyma20g24040

Module64

Glyma01g44060

Glyma02g37090

Glyma03g04760

Glyma04g09200

Glyma05g02790

Glyma06g13470

Glyma08g16380

Glyma10g04350

Glyma13g38490

Module65

Glyma01g01310

Glyma06g05460

Glyma09g08120

Glyma09g08550

Glyma12g03570

Glyma12g10960

Glyma13g11090

Glyma13g17420

Glyma14g06160

Glyma16g29370

Glyma19g34380

Glyma20g24670

Module66

Glyma01g31360

Glyma03g41630

Glyma07g00580

Glyma07g02460

Glyma07g15510

Glyma08g20060

Glyma08g29130

Glyma10g28370

Glyma12g05510

Glyma13g00380

Glyma13g30990

Glyma14g12570

Glyma15g08390

Glyma18g50240

Glyma19g38910

Module67

Glyma01g04370

Glyma01g32270

Glyma01g38040

Glyma03g01830

Glyma03g40210

Glyma03g40220

Glyma04g00210

Glyma04g01890

Glyma04g13990

Glyma04g14360

Glyma04g42300

Glyma05g01620

Glyma05g25010

Glyma05g36290

Glyma06g40400

Glyma06g47690

Glyma07g08280

Glyma07g08290

Glyma07g08830

Glyma07g11220

Glyma07g38620

Glyma08g08170

Glyma08g25400

Glyma08g44700

Glyma09g15200

Glyma09g31450

Glyma10g10240

Glyma10g32070

Glyma12g33750

Glyma13g23770

Glyma13g27300

Glyma13g37990

Glyma13g39650

Glyma13g39850

Glyma14g03890

Glyma15g17420

Glyma16g02520

Glyma16g06530

Glyma17g07440

Glyma18g06350

Glyma18g47010

Glyma18g53220

Glyma19g03450

Glyma19g29190

Glyma19g42790

Glyma19g43570

Glyma20g03850

Glyma20g29210

Glyma20g30450

Glyma20g33290

Module68

Glyma01g39650

Glyma02g07670

Glyma02g09550

Glyma02g40840

Glyma05g21030

Glyma05g26500

Glyma07g04810

Glyma07g17810

Glyma07g35640

Glyma11g11310

Glyma11g12000

Glyma12g30210

Glyma13g06080

Glyma15g24610

Glyma16g05560

Glyma17g03310

Glyma17g09440

Glyma19g33920

Module69

Glyma02g12970

Glyma03g30460

Glyma05g04290

Glyma06g03100

Glyma06g12640

Glyma06g17360

Glyma07g10220

Glyma08g01100

Glyma10g39330

Glyma11g37010

Glyma13g05120

Glyma13g27800

Glyma15g18360

Module70

Glyma01g07120

Glyma03g15850

Glyma06g44430

Glyma07g17250

Glyma08g22170

Glyma08g39510

Glyma11g05840

Glyma11g10340

Glyma11g12630

Glyma12g22660

Glyma13g17650

Glyma14g08230

Glyma17g02410

Glyma17g36940

Glyma18g02580

Glyma18g10930

Glyma18g11010

Glyma20g02580

Glyma20g16910
